# Catalytic Asymmetric β‐Oxygen Elimination**

**DOI:** 10.1002/anie.202114044

**Published:** 2022-03-25

**Authors:** Christof Matt, Andreas Orthaber, Jan Streuff

**Affiliations:** ^1^ Department of Chemistry—BMC Uppsala University Husargatan 3 75237 Uppsala Sweden; ^2^ Institut für Organische Chemie Albert-Ludwigs-Universität Freiburg Albertstr. 21 79104 Freiburg im Breisgau Germany; ^3^ Department of Chemistry—Ångström Laboratory Uppsala University Lägerhyddsvägen 1 75237 Uppsala Sweden

**Keywords:** Asymmetric Catalysis, Reduction, Regiodivergent Reaction, Zirconium, β-Elimination

## Abstract

A catalytic enantioselective β‐O‐elimination reaction is reported in the form of a zirconium‐catalyzed asymmetric opening of *meso*‐ketene acetals. Furthermore, a regiodivergent β‐O‐elimination is demonstrated. The reaction proceeds under mild conditions, at low catalyst loadings, and produces chiral monoprotected *cis‐*1,2‐diols in good yield and enantiomeric excess. The combination with a Mitsunobu reaction or a one‐pot hydroboration/Suzuki reaction sequence then gives access to additional diol and aminoalcohol building blocks. A stereochemical analysis supported by DFT calculations reveals that a high selectivity in the hydrozirconation step is also important for achieving high enantioselectivity, although it does not constitute the asymmetric step. This insight is crucial for the future development of related asymmetric β‐elimination reactions.

## Graphical abstract

A catalytic enantioselective β‐O‐elimination is reported. The reaction design utilizes the symmetry of *meso*‐ketene acetal substrates and proceeds in the presence of a chiral zirconocene catalyst. Furthermore, a regiodivergent β‐O‐elimination, the application in diol and amino alcohol synthesis, and a DFT‐supported stereochemical analysis are presented.

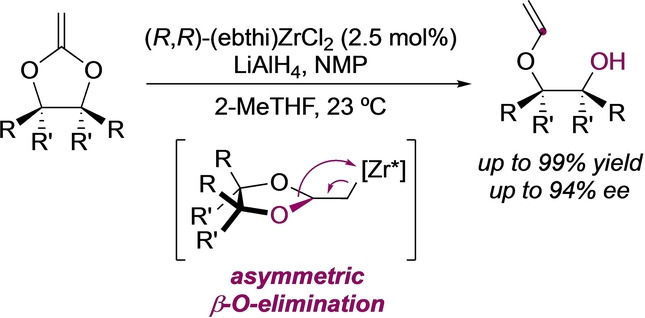

The development of new types of catalytic asymmetric bond formations and activations is at the heart of modern stereoselective synthesis.^[1]^ One particularly intriguing case is β‐elimination reactions that occur as a mechanistic key step in numerous important transition metal catalyzed processes.[^2^, ^3^] In the past, catalytic enantioselective β‐carbon eliminations have been developed and applied as an exquisite tool for the construction of quaternary stereocenters (Scheme 1).[^2c^, ^2d^, ^4^] In contrast, catalytic enantioselective reactions involving a β‐heteroatom elimination event usually have a preceding asymmetric hydro‐, carbo‐, or nucleometalation step.[^2b^, ^5^] A direct enantioselective β‐heteroatom elimination reaction in which the β‐Het cleavage itself is stereodiscriminating has remained elusive with exception of an asymmetric β‐F‐elimination that was published very recently.^[6]^ In this work, we now report a zirconium‐catalyzed enantioselective β‐oxygen elimination reaction in the form of an opening of cyclic ketene acetals. The reaction gives access to enantioenriched mono‐vinylated diols that are versatile precursors of numerous chiral building blocks but difficult to prepare by other means.^[7]^

**Scheme 1:**
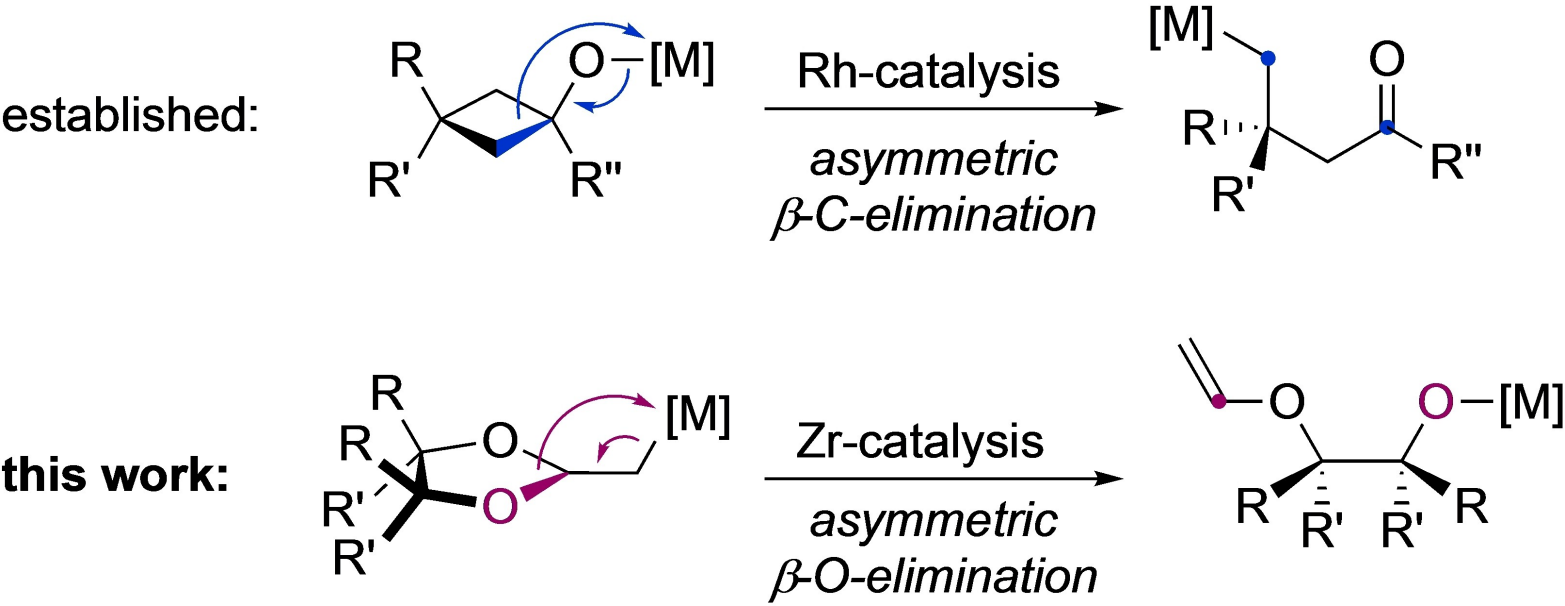
Concept of a catalytic asymmetric β‐O‐elimination.

Our group has been exploring zirconium‐catalyzed β‐elimination reactions as a tool for the selective cleavage of unreactive carbon‐heteroatom bonds.[^8^, ^9^] The challenging nature of the targeted bonds, the abundant and non‐toxic nature of zirconium and the fact that catalytic β‐Het‐elimination has mostly been reported with late transition metals render this approach attractive.^[3]^

We reasoned that an asymmetric β‐O‐elimination could be realized with substrates having two enantiotopic C−O bonds and, therefore, *meso*‐ketene acetals were chosen as precursors. After an initial hydrozirconation with an in situ generated chiral zirconium hydride catalyst, the following ring opening by β‐elimination would be the desymmetrizing step. The asymmetric opening would lead to selectively monoprotected enantioenriched 1,2‐diols, rendering the reaction a counterpart to common *meso*‐diol‐desymmetrizing acylation and sulfonylation reactions.^[10]^

Starting with *meso*‐compound **1 a** as substrate, it was quickly discovered that a highly enantioselective ring‐opening to **2 a** could be achieved in presence of 5 mol% (*R*,*R*)‐(ebthi)ZrCl_2_ (**cat‐1**) as precatalyst (Scheme 2, entry 1). A combination of LiAlH_4_ and *N*‐methylpyrrolidine (NMP) as previously established by us for Zr‐catalyzed β‐eliminations was found ideal for achieving turnover.^[8]^ Additional experiments showed that the yield in β‐vinyloxy alcohol **2 a** varied over time, reaching a maximum after 2 h. At this point, a precipitate had formed which was concluded to be a weakly soluble aluminium alkoxide of **2 a**, hampering the hydrozirconation and cleavage of the vinyl group.^[8]^ Extending the reaction time led to overreduction, which resulted in significant amounts of achiral *cis*‐cyclohexanediol. The enantioselectivity remained high, regardless of the reaction time (92–96 % ee). An optimum in yield while maintaining 92 % ee could be achieved at a lower catalyst loading of 2.5 mol% and 4 h reaction time (entry 2). A further reduction in catalyst amount and the absence of NMP reduced the yield, but the stereoselectivity remained unaffected (entries 3, 4). Switching the solvent to the more polar THF, led again to overreduction, diminishing the yield in **2 a** to 46 % (entry 5). Using the optimized conditions, we briefly investigated other catalysts.[^11^, ^12^] Ebthi‐titanocene **cat‐2** did not catalyze the reaction. Other literature‐known bridged and unbridged zirconocene dichlorides resulted in inferior stereodiscrimination (<30 % ee) and reduced yields. An in situ formed Zr‐TADDOL catalyst (**cat‐6**), on the other hand, gave respectable 79 % *ee*, but no turnover could be achieved. Overall, the ethylene‐bridged catalyst **cat‐1**, having a well‐defined and conformationally locked structure, was a superior catalyst for this transformation. The absolute configuration of **2 a** was established by X‐ray analysis of a *para*‐bromobenzoate derivative as (*S*,*R*).^[13]^

**Scheme 2:**
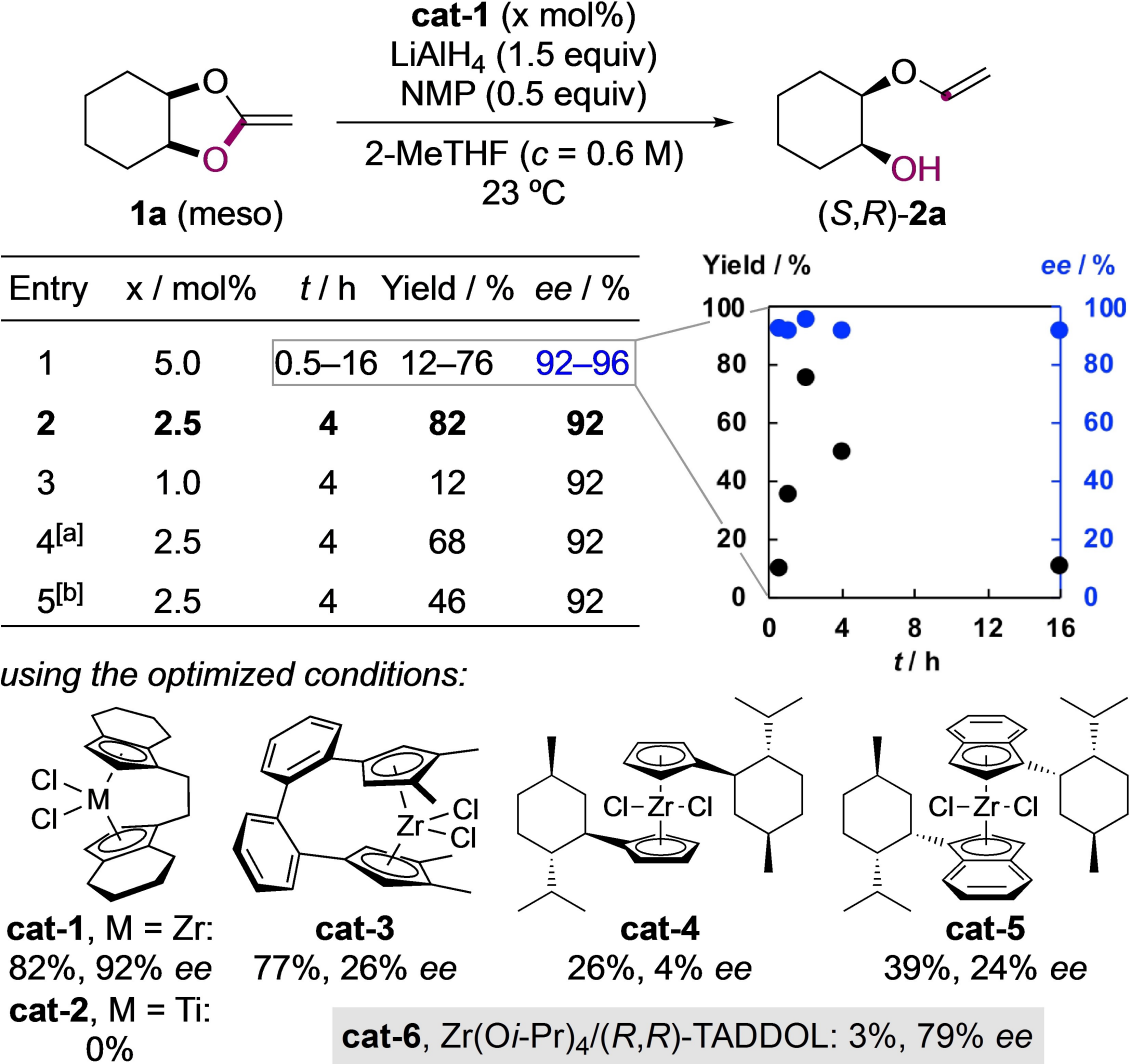
Reaction optimization. [a] Without NMP. [b] In THF as solvent.

We were delighted to find that the reaction could be carried out on larger scale (2.6 mmol) with similar yield and enantioselectivity (Table 1, entry 1b). A reaction with the (*S*,*S*)‐catalyst gave (*R*,*S*)‐**2 a** in 80 % yield and 92 % *ee*. Using the optimized conditions, we then investigated a number of structurally modified ketene acetals that were prepared from the corresponding diols by transacetalization with chloroacetaldehyde dimethyl acetal followed by an elimination step.^[14]^ The water sensitive compounds were purified by distillation and could be stored for 24 h at −20 °C. Ketene acetals with annulated five‐ and seven‐membered rings worked well (entries 2 and 3) giving 90 % and 88 % *ee* and very high yields (99 % and 96 %, respectively). An eight‐membered ring, showing higher conformational flexibility, still led to a good stereodiscrimination (83 % *ee*, entry 4). A mono‐vinylated dihydroxytetrahydrofuran was produced in 85 % yield and 90 % *ee* (entry 5). Exchanging the THF oxygen by a *trans*‐configured phenyl‐substituted carbon center led to 67 % yield and 78 % *ee* (entry 6). A ketene acetal derived from 2‐benzyl‐propane‐1,3‐diol (**1 g**), having only a remotely located prochiral center, led to a diminished enantioselectivity.^[15]^ This could be rationalized by a reduced facial preference for the hydrozirconation of the ketene acetal double bond,^[11]^ which in turn was essential for achieving an asymmetric C−O bond cleavage (see below). Increasing the concentration to *c*=1.0 M improved the yield in **2 g** from 25 % to 74 %, a possible explanation being the prevention of overreduction through increased precipitation. Substrates that were derived from secondary alcohols and featured distinct concave and convex faces, on the other hand, gave excellent results. This was also true for substrate **1 h** derived from a linear *meso*‐diol, which afforded the ring‐opening product **2 h** in 71 % yield and 84 % *ee* (entry 8). Norbornene‐ and norbornadiene‐based tricyclic ketene acetals gave good yields and high enantiomeric excess as well (entries 9, 10). Importantly, the internal alkene function of **1 j** was retained and only a small quantity of **2 i** was observed (19 : 1 ratio).

**Table 1:**
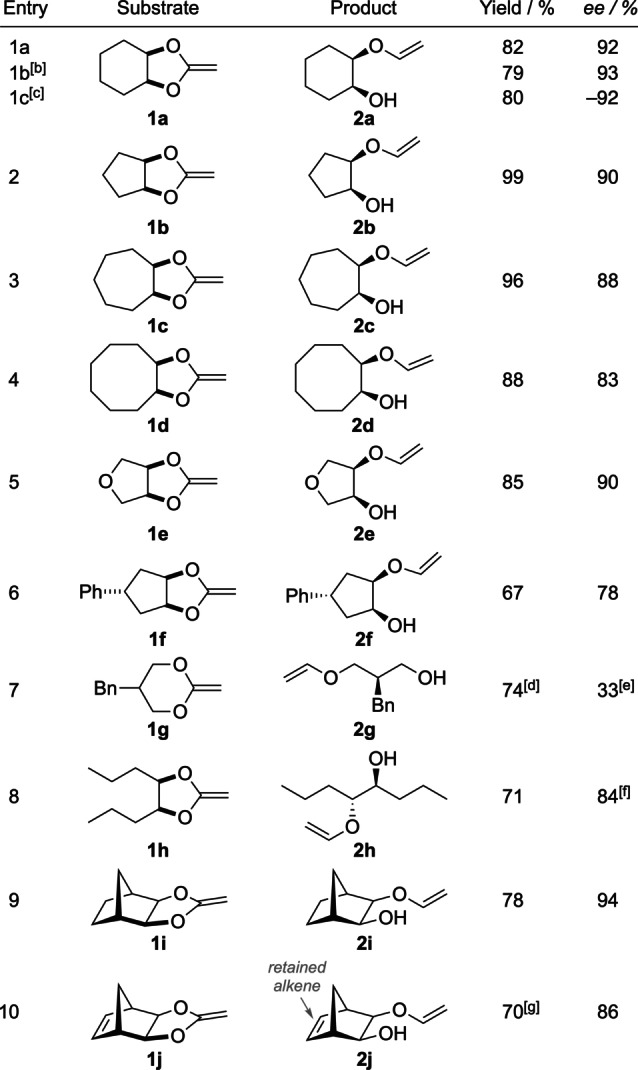
Scope of the enantioselective β‐O‐elimination reaction.^[a]^

[a] Reactions on the 0.2 mmol scale. Conditions: Scheme 2, table entry 2. [b] 2.6 mmol scale reaction. [c] Reaction with 2.5 mol% (*S*,*S*)‐(ebthi)ZrCl_2_, giving (*R*,*S*)‐**2 a** as product. [d] Reaction run at *c*=1.0 M. [e] The absolute configuration of **2 g** was not determined. [f] Reaction run at 18 °C for 9 h. [g] Contained 5 % of **2 i** as determined by GC‐MS.

The concept of asymmetric β‐elimination was then taken to the next level by attempting the regiodivergent opening of pseudo‐*meso* substrate *rac*‐**3** (Scheme 3),^[16]^ which was prepared from *cis*‐1‐phenylhex‐3‐ene by dihydroxylation.^[11]^ Ideally, the remote structural divergence between the phenyl group and methyl termination of the backbone would not influence the selectivity of the catalysis. The two substrate enantiomers would then lead to one enantioenriched regioisomer each (**4** and **5**) in a ratio of 1 : 1. We were pleased to find that the reaction gave the desired regioisomers in 83 % and 78 % *ee* in a 1.1 : 1 mixture and 64 % combined yield. The products were further oxidized and hydrolyzed to the free regioisomeric α‐hydroxyketones in 91 % overall yield and without significant loss (3–4 %) in *ee*.^[11]^

**Scheme 3:**
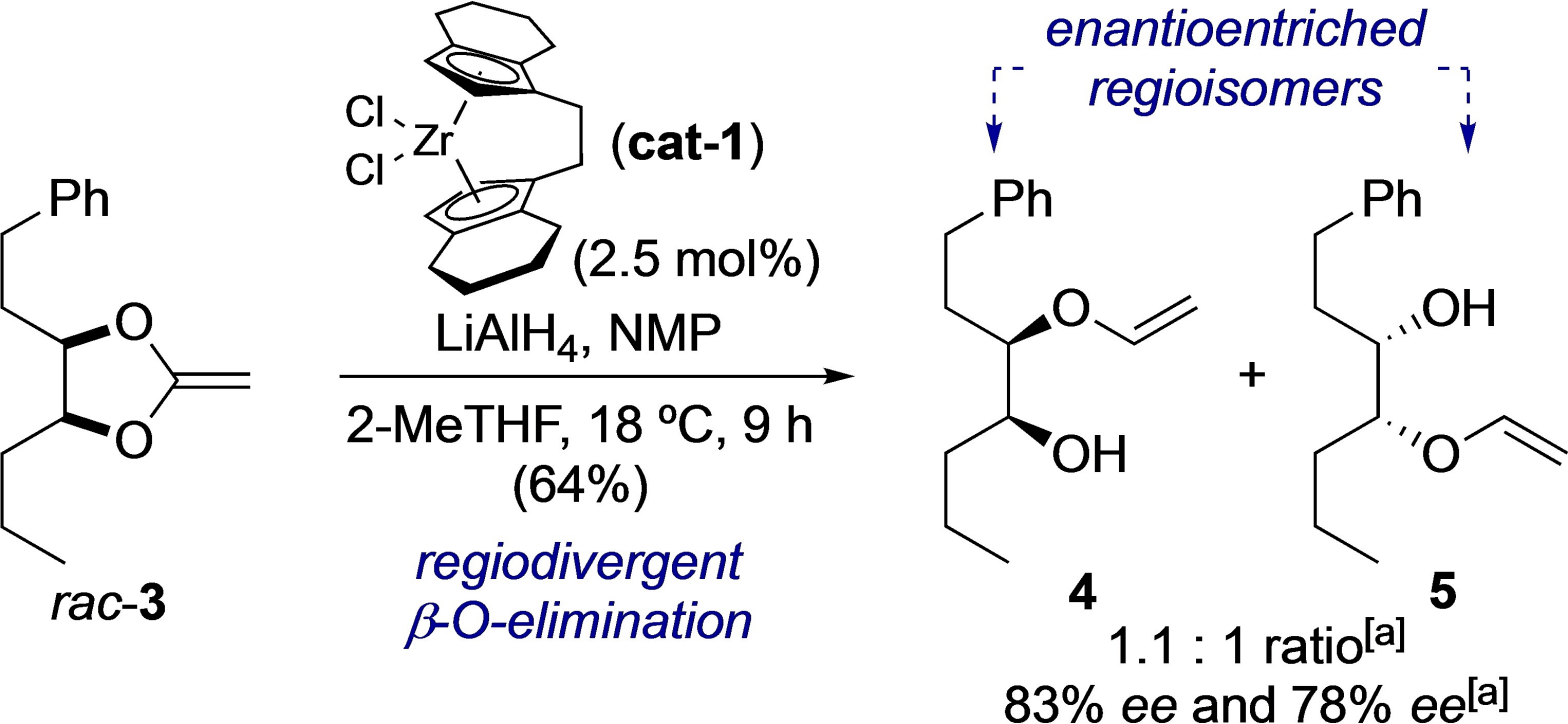
Regiodivergent β‐O‐elimination. [a] Determined by chiral HPLC.

To show a potential application of the asymmetric β‐O‐elimination approach in the stereoselective synthesis of enantioenriched 1,2‐diols and ‐aminoalcohols, the asymmetric ketene acetal opening was combined with a Mitsunobu reaction (Scheme 4, top). Using *para*‐nitrobenzoic acid as nucleophile and conditions optimized for Mitsunobu inversions of cyclohexanols,^[17]^ the *cis*‐diol (*S*,*R*)‐**2 a** was converted into the orthogonally protected *trans*‐diol (*R*,*R*)‐**6** in 83 % yield and with full conservation of enantiopurity (92 % *ee*). The enantioenriched *trans*‐aminoalcohol (*R*,*R*)‐**7** could be accessed in 77 % yield and 91 % *ee* by an analogous reaction with phthalimide. Likewise, (*R*,*S*)‐**2 a**, prepared with (*S*,*S*)‐**cat‐1**, was converted into the opposite enantiomers of **6** and **7** in high yield and *ee*.^[11]^ Overall, all 1,2‐diol diastereo‐ and enantiomers as well as the *trans*‐1,2‐aminoalcohol enantiomers were prepared in good yield and high enantiomeric excess. In a separate experiment, we converted the vinyl ether **2 a** into arylethyl ether **8** in a one‐pot hydroboration/Suzuki coupling in 79 % yield and without erosion of the enantiopurity (92 % *ee*, Scheme 4, bottom).^[18]^

**Scheme 4:**
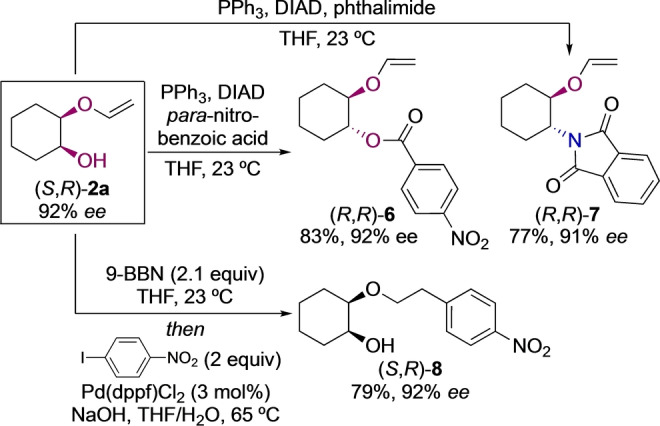
Top: accessing enantioenriched, orthogonally protected diols and aminoalcohols. Bottom: further elaboration of the vinyl ether group.

A stereochemical analysis of the reaction course provided a rationale for the observed stereoselectivity (Scheme 5). The reaction involved two potentially stereoselectivity‐determining events: first, the hydrozirconation that either occurred from the concave or the less hindered convex side of the molecule. The two corresponding transition states would then give the *anti*‐intermediate **9** and kinetically favored *syn*‐intermediate **10**, respectively. Secondly, the β‐elimination step followed, which could occur from two rotational conformers of the individual intermediates, one of which would lead to a significantly lower transition state due to minimization of steric repulsion. In the case of **9**, the favored transition state was **TS**‐**11** showing minimal steric interactions between the substrate and the ebthi ligand. It transitioned into **11** and ultimately gave (*R*,*S*)‐**2 a**. The bowl‐shaped *syn*‐intermediate **10**, on the other hand, led to transition state **TS**‐**12**, with the two stereocenters at the ring junction being inverted, allowing the cyclohexyl ring to point away from the tetrahydroindenyl group of the ligand. The favored product **12** was the direct precursor to (*S*,*R*)‐**2 a**. As a direct consequence of this scenario, the enantioselectivity of the reaction was dependent on the selectivity of the hydrozirconation step. This rationale was confirmed by a computational analysis of the reaction paths (Figure 1). The calculations were performed on the PW6B95‐D4‐CPCM/def2‐QZVP//PBEh‐3c‐CPCM level,[^19^, ^20^] using the ORCA 4.2.1^[21]^ program package. Several transition states including coordination isomers, conformers and rotamers were computed for the hydrozirconation and β‐O‐elimination steps each. In detail, it was found that the hydrozirconation from the convex side (leading to **10**) was favored by 2.6 kcal mol^−1^. The corresponding transition states **TS**‐**9** and **TS**‐**10** were at 20.1 and 17.5 kcal mol^−1^, which were in agreement with the rapid reaction at room temperature. The barriers for the β‐O‐elimination (**TS**‐**11** and **TS**‐**12**) were significantly lower (10.6 and 12.1 kcal mol^−1^, respectively). For comparison, the lowest β‐O‐elimination transition states leading to the respective opposite enantiomers (**TS**‐**11′** and **TS**‐**12′**) were calculated and added to Figure 1. However, these were significantly higher in energy (ΔΔ*G* ^≠^=7.0 and 4.4 kcal mol^−1^, respectively). Hence, the favored convex pathway would exclusively give (*S*,*R*)‐**2 a** and the disfavored concave pathway only (*R*,*S*)‐**2 a**. We further calculated the reaction pathways starting from (*R*,*R*)‐(ebthi)Zr(H)Cl as alternative hydrozirconation precursor, but this led to significantly higher activation barriers.[^11^, ^22^] Moreover, zirconocene dichlorides typically give the corresponding dihydrides if treated with an excess of LiAlH_4_ or similar hydride reagents.^[23]^ Overall, the calculations indicated that the hydrozirconation was selectivity‐determining and slower than the β‐O‐elimination. This was further supported by a kinetic isotope effect of *k*_H_/*k*_D_=1.5 observed at the vinylic position in a stoichiometric experiment with a 1 : 1 mixture of pre‐generated Zr−H and Zr−D species.^[11]^ The enantiomeric excess calculated from the difference in transition state energies corresponded to 97.5 % *ee*, which was in good agreement with the experimentally observed range of 92–96 % *ee*.

**Scheme 5:**
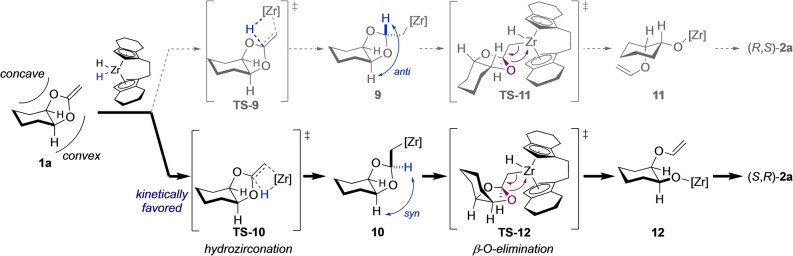
Stereochemical analysis for the observed selectivity.

**Figure 1:**
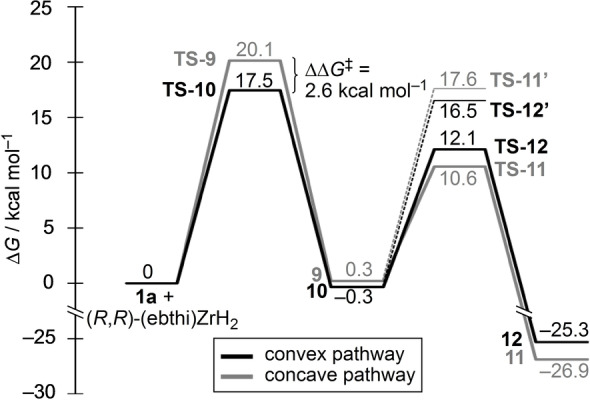
Calculation of the paths in Scheme 5 (PW6B95‐D4‐CPCM(2‐MeTHF)/def2‐QZVP//PBEh‐3c). Values are the Gibbs free Energy in kcal mol^−1^.

In conclusion, a zirconium‐catalyzed asymmetric β‐O‐elimination has been developed on the example of an enantioselective opening of *meso*‐ketene acetals. The reaction proceeds at mild temperatures and it gives enantioenriched, monoprotected diols in high yield and *ee*. As a proof‐of‐principle, a regiodivergent β‐O‐elimination has been demonstrated. If desired, the products can be converted further into *trans*‐diols and ‐aminoalcohols as well as mono‐arylethyl ethers without loss in enantiopurity. A stereochemical analysis supported by DFT calculations revealed that an efficient site differentiation in the non‐symmetry‐breaking hydrozirconation step is already important for achieving high enantioselectivity. This insight will greatly facilitate the development of other asymmetric β‐elimination reactions using chiral zirconocene‐based catalysts. Since chiral diols and vinyl ethers are frequently employed as precursors in organic synthesis and polymer chemistry, the enantioselective β‐O‐elimination presented herein could be of broader relevance to these areas.^[24]^

## Conflict of interest

The authors declare no conflict of interest.

## Supporting information

As a service to our authors and readers, this journal provides supporting information supplied by the authors. Such materials are peer reviewed and may be re‐organized for online delivery, but are not copy‐edited or typeset. Technical support issues arising from supporting information (other than missing files) should be addressed to the authors.

Supporting Information

Supporting Information

Supporting Information
