# Supplementary material for: Catalytic Asymmetric β‐Oxygen Elimination
Source: Angew Chem Int Ed Engl. 2022 Mar 25;61(22):e202114044. doi: 10.1002/anie.202114044 (PMC9314826; doi:10.1002/anie.202114044)
Supplement: Supplementary file 2 — Supporting Information [file ANIE-61-0-s003.pdf]

Angewandte Chemie
Eine Zeitschrift der Gesellschaft Deutscher Chemiker

Supporting Information

Catalytic Asymmetric β-Oxygen Elimination

*C. Matt, A. Orthaber, J. Streuff**

Table of Contents

Materials and Methods **S2**

Substrate Synthesis **S3**

Zr-Catalyzed Desymmetrization of Cyclic Ketene Acetals **S16**
- General Procedure S16
- Purification and Characterization Products S16

Product Derivatization **S21**
- Determination of the Absolute Configuration of **2a** S21
- Oxidation and Deprotection of Regioisomers **4** and **5** S22
- Synthesis of Enantioenriched *trans*-Diol and *trans*-Aminoalcohol Derivatives S24
- One-Pot Hydroboration/Suzuki Coupling S26

Additional Experiments with Unsymmetric Substrates **S26**

Kinetic Isotope Effect Experiments **S32**

Initial Reaction Optimization and Experiments with other Catalysts **S36**
- Screening Results and Discussion of Quenching Byproducts S36
- Characterization of Hydrolysis Byproducts **S44–S46** S37
- Synthesis of Zirconocene Catalysts **cat-4** and **cat-5** S39

Conditions for the Determination of Enantiomeric Excess **S42**

Computational Studies **S46**
- General remarks S46
- Reaction with the Dihydride Catalyst (as in Scheme 5/Figure 1) S47
- Alternative Zirconium Chloride-Hydride Pathways (not shown in the manuscript) S49
- Coordinates S53

References **S103**

NMR Spectra **S105**

HPLC and GC reports **S186**

X-Ray Analysis Report for Compound S12 .... **S247**

X-Ray Analysis Report for Compound S27 .... **S251**

Materials and Methods

All reactions have been carried out in flame-dried Schlenk tubes or Schlenk flasks under an argon atmosphere and using absolute solvents unless noticed otherwise. Absolute THF was dried over sodium and benzophenone under an argon atmosphere and freshly distilled prior to use. 2-Methyltetrahydrofuran (2-Me-THF) was dried using a column of basic Al_2O_3. Dichloromethane was purchased in p.a. quality from Fisher. Diethyl ether was purchased in p.a. quality from VWR. *rac*-**cat-1**, (*R,R*)-**cat-1**, (*S,S*)-**cat-1**, (*R,R*)-**cat-2**, and (*R*)-**cat-3** were purchased from MCAT GmbH, Germany (www.mcat.de). All remaining chemicals were purchased from Merck, VWR, TCI, ABCR or Acros. An IKAmag temperature modulator in combination with an oil bath was used to control the reaction temperatures. Thin-layer chromatography (TLC) was performed using E. Merck silica gel 60 F254 precoated plates (0.25 mm) and visualized by UV fluorescence quenching or staining ($KMnO_4$). In general, Macherey-Nagel Silica gel 60 (particle size 0.04–0.063 mm) was used for flash chromatography. ^1H, ^{13}C NMR spectra were recorded on a Bruker DRX 500 (500 MHz and 125 MHz, ^1H, ^{13}C, 2D-spectra), a Bruker Avance II 400 (400 MHz and 100 MHz, ^1H, ^{13}C, 2D-spectra), a Bruker Avance III 300 (300 MHz), spectrometer and reported to $CDCl_3$ [δ(1H) = 7.26 ppm and δ (13C) = 77.16 ppm] or C_6D_6 [$\delta(^1H)$ = 7.16 ppm and $\delta(^{13}C)$ = 128.06 ppm] The following abbreviations were used: s = singlet, d = doublet, t = triplet, q = quartet, m = multiplett. NMR spectra were recorded at room temperature (298–300 K) unless noted otherwise. IR spectra were recorded on a Thermo Scientific Nicolet iS10 FT-IR spectrometer equipped with a diamond ATR unit and are reported in frequency of absorption. Low- and high-resolution mass analyses were performed by the service department at the Institute for Organic Chemistry, University of Freiburg using a Thermo Finnigan TSQ 700 for electron impact ionization (EI) at 70 eV, 200 °C. High resolution mass analyses (HRMS) were carried out on a Thermo Exactive with Orbitrap-Analyzer using atmospheric pressure chemical ionization (APCI). Chiral GC analyses were carried out on an Agilent 6890N with a Macherey-Nagel Hydrodex-β-TBDAc column (25 m × 250 mm ID). Chiral HPLC-analyses were carried out on a Shimadzu LC-2010C HT Liquid Chromatography system and the reported columns (Chiralpak IA, particle size 5 µm; Chiralcel OD-H, particle size 5µm; ChiralPAK AD-H, particle size 5 µm).

Substrate Synthesis

The ketene acetal substrates were prepared from the corresponding alkenes via the following sequence and purified by distillation. The substrates were of acceptable purity as judged by NMR.

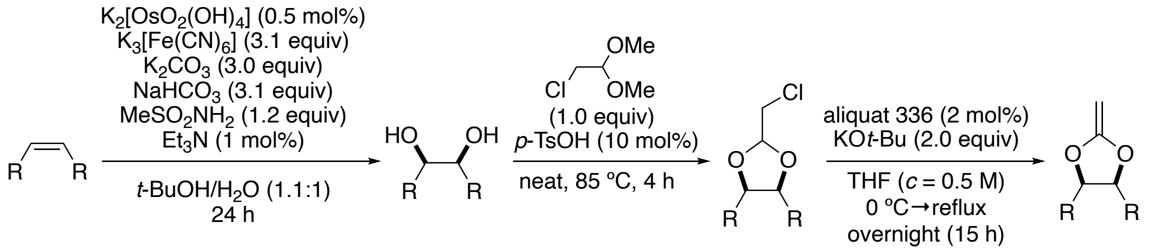

General procedure for dihydroxylation of alkenes. Dihydroxylation of alkenes was performed following a modified literature procedure.[1] The given alkene (1 equiv) and NEt_3 (1 mol-%) were dissolved in *t*-BuOH (9.4 ml / mmol alkene). $K_3[Fe(CN)_6]$ (3.1 equiv), K_2CO_3 (3 equiv), $NaHCO_3$ (3.1 equiv), and $MeSO_2NH_2$ (1.2 equiv) dissolved in H_2O (8.3 ml / mmol alkene) were added and the two-layered reaction mixture was stirred vigorously. Then, $K_2OsO_2(OH)_4$ (0.5 mol-%) was added to the reaction mixture and stirring was continued at room temperature (23 °C) for 1 d. Afterwards, the reaction mixture was quenched by addition of Na_2SO_3 (7 equiv) as a solid. The reaction mixture was transferred into a separatory funnel and extracted with EtOAc (6 x 100 ml). The combined organic layers were washed with brine (300 ml), dried over Na_2SO_4, filtered, and the solvent was removed under reduced pressure. The crude products were purified as described individually.

General procedure for the synthesis of chloromethyldioxolanes. Chloromethyldioxolanes were synthesized following a modified literature.[2] A 10 ml Schlenk flask equipped with a magnetic stirring bar was charged with 2-chloro-1,1-dimethoxyethane (1 equiv) *p*-toluene sulfonic acid (10 mol%), and the given diol (1.0 equiv). The reaction mixture became homogenous upon warming and was stirred at 85°C for 4 h. Afterwards, it was cooled down to room temperature, the volatiles were removed under reduced pressure and the crude product was purified as described individually.

General procedure for the synthesis of cyclic ketene acetals. Cyclic ketene acetals were synthesized following a modified literature procedure.[3] All glassware (including NMR tubes) was put into a KOH/iPrOH bath for at least 30 min, then rinsed with demineralized water and finally dried at 110°C prior to use. Each reaction and each purification step were performed under an argon atmosphere. A 10 ml Schlenk flask equipped with a magnetic stirring bar was charged with Aliquat 336 (2 mol-%). The given 2-halomethyl-1,3-dioxolanes (1 equiv) dissolved in THF (c = 0.5M) was added dropwise. The reaction mixture was cooled down to 0°C (ice bath) and KO*t*-Bu (2 equiv) was added in one portion. The ice bath was removed and the reaction mixture was stirred under reflux overnight. Afterwards, it was cooled down to room temperature, the solvent was removed under reduced pressure (300 mbar) and the residue was purified by distillation as described individually. After distillation, the products were directly used in the next step.

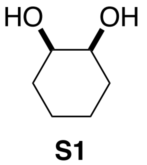

S1

1,2-*cis*-Cyclohexaneanediol. Synthesized from cyclohexene (1.6 ml, 1.2 g, 18.0 mmol, 1.0 equiv) following the general procedure for dihydroxylation of alkenes. The product was purified by column chromatography (EtOAc, R_f = 0.4). and obtained as a white solid in 89% yield (1.865

g, 16.06 mmol). The NMR data matched the literature values.[4] ^{1}H NMR (400 MHz, $CDCl_3$): δ = 1.24-1.33 (m, 2H), 1.48-1.65 (m, 4H), 1.69-1.78 (m, 2H), 2.82 (s, 2H), 3.73-3.75 (m, 2H). ^{13}C NMR (100 MHz, $CDCl_3$): δ = 21.59, 30.01, 70.80.

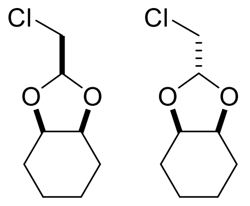

S2

***cis,cis*-2-(Chloromethyl)hexahydrobenzo[d][1,3]dioxole and *cis,trans*-2-(chloromethyl)hexahydrobenzo[d][1,3]dioxole.** Synthesized according to the general procedure for the synthesis of chloromethyldioxolanes from 1,2-*cis*-cyclohexaneanediol. (**S1**, 800 mg, 6.89 mmol, 1.0 equiv). The crude product was purified by distillation under reduced pressure (0.05 mbar, bp 85°C) and obtained as a colorless oil in 92% yield as a mixture of diastereomers (1.118 g, 6.327 mmol). ^{1}H NMR (400 MHz, $CDCl_3$): δ = 1.25-1.36 (m, 2H), 1.51-1.61 (m, 2H), 1.69-1.83 (m, 4H), 3.49 (d, J = 4.3 Hz, 1H), 3.58 (d, J = 4.4 Hz, 0.9H), 4.06-4.10 (m, 0.88H), 4.13-4.18 (m, 1H), 5.13 (t, J = 4.4 Hz, 0.42H), 5.38 (t, J = 4.3 Hz, 0.48H). ^{13}C NMR (100 MHz, $CDCl_3$): δ = 20.91, 21.10, 27.23, 28.49, 45.26, 45.58, 74.81, 75.17, 101.16, 102.35. HRMS (pos. APCI): calcd for $C_8H_{13}ClO_2$ $[M+NH_4]^+$: 194.0942, found: 194.0942. MS (CI, 70 eV): m/z (%) = 196.1 (7), 194.1 $[M+NH_4]^+$ (17), 127.1 (100), 181.1 (49). IR (ATR): ν [cm^{-1}] = 613, 648, 726, 758, 809, 905, 1008, 1036, 1099, 1113, 1135, 1195, 1303, 1338, 1364, 1384, 1447, 1619, 2941.

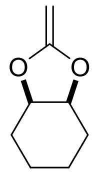

1a

***cis*-2-Methylenehexahydrobenzo[d][1,3]dioxole.** Synthesized according to the general procedure for the synthesis of cyclic ketene acetals from *cis,cis*-2-(chloromethyl)hexahydrobenzo[d][1,3]dioxole and *cis,trans*-2-(chloromethyl)-hexahydrobenzo[d][1,3]dioxole (**S2**, 529.8 mg, 3.0 mmol, 1.0 equiv) The crude product was purified by distillation under reduced pressure (0.05 mbar, bp 85°C) and obtained as a colorless oil in 54% yield (228.3 mg, 1.628 mmol). ^{1}H NMR (300 MHz, C_6D_6): δ = 0.71-0.84 (m, 2H), 1.13-1.28 (m, 4H), 1.51-1.63 (m, 2H), 3.69 (d, 2H), 3.74-3.80 (m, 2H). ^{13}C NMR (100 MHz, C_6D_6): δ = 20.39, 26.80, 55.41, 75.16, 164.03. HRMS (pos. APCI): calcd for $C_8H_{12}O_2$ $[M+H_3O]^+$: 159.1016, found: 159.1016. MS (CI, 70 eV): m/z (%) = 142.1 (9), 141.1 $[M+H]^+$ (100), 140.1 (8), 98.1 (2), 81.1 (4). IR (ATR): ν [cm^{-1}] = 668, 789, 890, 1110, 1256, 1382, 1641, 1675, 1710, 2340, 2360, 2937, 3423.

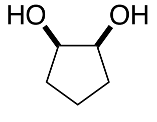

S3

1,2-*cis*-Cyclopentanediol. Synthesized from cyclopentene (1.6 ml, 1.2 g, 18.0 mmol, 1.0 equiv) following the general procedure for dihydroxylation of alkenes. The product was purified by column chromatography (cyclohexane/acetone, 3:1, Rf = 0.3) and obtained as a white solid in 62% yield (1.138 g, 11.14 mmol). The NMR data matched the literature values.[5] ^{1}H NMR (400 MHz, $CDCl_3$): δ = 1.44-1.54 (m, 1H), 1.62-1.71 (m, 2H), 1.79-1.92 (m, 3H), 2.26 (s, 2H), 4.01-4.06 (m, 2H). ^{13}C NMR (100 MHz, $CDCl_3$): δ = 19.90, 31.32, 74.06.

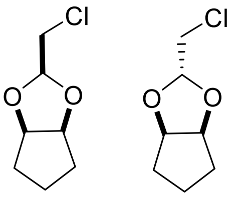

S4

***cis,cis*-Chloromethyltetrahydro-4H-cyclopenta[d][1,3]dioxole and *cis,trans*-2-(chloromethyl)tetrahydro-4H-cyclopenta[d][1,3]dioxole.** Synthesized according to the general procedure for the synthesis of chloromethyldioxolanes from 1,2-*cis*-cyclopentanediol. (**S3**, 1.021g, 10.0 mmol, 1.0 equiv). The crude product was purified by distillation under reduced pressure (0.5 mbar, bp 74°C) and obtained as a colorless oil in 94% yield as a mixture of diastereomers (1.526 g, 9.387 mmol). ^{1}H NMR (500 MHz, C_6D_6): δ = 0.88-1.04 (m, 2H), 1.14-1.23 (m, 1H), 1.43-1.53 (m, 0.17H), 1.67-1.81 (m, 2.84H), 3.13 (d, *J* = 4.0 Hz, 0.32H), 3.30 (d, *J* = 3.7 Hz, 1.62H), 4.09-4.12 (m, 1.57H), 4.28-4.31 (m, 0.31H), 4.69 (t, *J* = 3.7 Hz, 0.77H), 5.16 (t, *J* = 4.0 Hz, 0.15H). ^{13}C NMR (125 MHz, C_6D_6): δ = 22.39, 23.31, 33.02, 33.40, 43.95, 45.25, 81.98, 82.15, 102.13, 103.91. HRMS (pos. APCI): calcd for $C_{10}H_{17}ClO_2$ $[M+H]^+$: 163.0520, found: 163.0520. MS (CI, 70 eV): m/z (%) = 180.1 (6) $[M+NH]^+$, 161.1 (2), 126.1 (3), 114.1 (6), 113.1 (100), 85.1 (2), 84.1 (2), 83.1 (3), 67.0 (13). IR (ATR): ν [cm^{-1}] = 664, 757, 812, 863, 963, 1028, 1140, 1214, 1260, 1330, 1419, 1742, 2280, 2964, 3123.

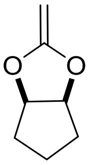

1b

***cis*-2-Methylenetetrahydro-4H-cyclopenta[d][1,3]dioxole.** Synthesized according to the general procedure for the synthesis of cyclic ketene acetals from *cis,cis*-chloromethyltetrahydro-4H-cyclopenta[d][1,3]dioxole *and* *cis,trans*-2-(chloromethyl)tetrahydro-4H-cyclopenta[d][1,3]dioxole (**S4**, 500 mg, 3.08 mmol, 1 equiv**).** The crude product was purified by distillation under reduced pressure (0.5 mbar, bp 62°C) and obtained as a colorless oil in 20% yield (78.3 mg, 0.621 mmol). ^{1}H NMR (500 MHz, C_6D_6): δ = 0.77-0.85 (m, 2H), 1.06-1.12 (m, 1H), 1.44-1.54 (m, 1H), 1.65-1.70 (m, 2H), 3.51 (s, 2H), 4.22-4.25 (m, 2H). ^{13}C NMR (125 MHz, C_6D_6): δ = 22.24, 33.40, 52.04, 82.66, 128.00, 165.82. HRMS (pos. APCI): calcd for $C_7H_{10}O_2$ $[M+H]^+$: 127.0754, found: 127.0755. MS (CI, 70 eV): m/z (%) = 127.1 (100) $[M+H]^+$, 126.1 (6), 118.1 (1), 102.1 (1), 84.0 (1), 77.2 (1). IR (ATR): ν [cm^{-1}] = 606, 609, 704, 812, 1033, 1102, 1262, 1330, 1389, 1453, 1618, 2269, 2280.

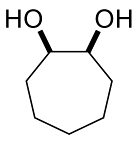

S5

1,2-*cis*-Cycloheptanediol*.* Synthesized from cycloheptene (2.1 ml, 1.7 g, 18.0 mmol, 1.0 equiv) following the general procedure for dihydroxylation of alkenes. The product was purified by column chromatography (cyclohexane/acetone, 2:1, Rf = 0.3) and obtained as a white solid in 59% yield (1.377 g, 10.58 mmol). The NMR data matched the literature values.[6] ^{1}H NMR (400 MHz, C_6D_6): δ = 1.32-1.42 (m, 2H), 1.45-1.55 (m, 1H), 1.56-1.84 (m, 7H), 2.20 (s, 2H), 3.84-3.88 (m, 2H). ^{13}C NMR (100 MHz, C_6D_6): δ = 22.04, 28.00, 31.15, 73.97.

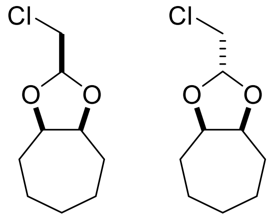

S6

***cis,cis*-2-(Chloromethyl)hexahydro-4H-cyclohepta[d][1,3]dioxole and *cis,trans*-2-(chloromethyl)hexahydro-4H-cyclohepta[d][1,3]dioxole.** Synthesized according to the general procedure for the synthesis of chloromethyldioxolanes from 1,2-*cis*-cyclohexanediol. (**S5**, 2.9 g, 22.0 mmol, 1 equiv). The crude product was purified by by column chromatography (cyclohexane/acetone, 9:1, Rf = 0.6) and obtained as a colorless oil in 73% yield as a mixture of diastereomers (3.06 g, 16.1 mmol). ^{1}H NMR (400 MHz, C_6D_6): δ = 0.81-0.95 (m, 2H), 1.03-1.19 (m, 1H), 1.25-1.35 (m, 1H), 1.43-1.65 (m, 6H), 3.20 (d, *J* = 4.2 Hz, 0.57H), 3.38 (d, *J* = 4.0 Hz, 1.36H), 3.79-3.85 (m, 1.33H), 3.98-4.04 (m, 0.55H), 4.88 (t, *J* = 3.9 Hz, 0.65H), 5.29 (t, *J* = 4.2 Hz, 0.26H). ^{13}C NMR (100 MHz, C_6D_6): δ = 24.03, 24.15, 30.50, 30.73, 30.88, 31.11, 44.46, 45.32, 79.77, 80.06, 101.63, 102.22. HRMS (pos. APCI): calcd for $C_9H_{15}ClO_2$ $[M+NH_4]^+$: 208.1099 found: 208.1101. MS (CI): m/z (%) = 210.1 (8), 208.2 (21) $[M+NH_4]^+$, 189.1 (16), 155.2 (13), 142.2 (8) 141.1 (100), 95.1 (20). IR (ATR): ν [cm^{-1}] = 570, 581, 767, 942, 993, 1042, 1094, 1136, 1362, 1430, 1450, 2857, 2931.

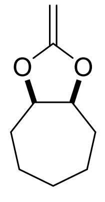

1c

***cis*-2-Methylenehexahydro-4H-cyclohepta[d][1,3]dioxole.** Synthesized according to the general procedure for the synthesis of cyclic ketene acetals from *cis,cis*-2-(chloromethyl)hexahydro-4H-cyclohepta[d][1,3]dioxole and *cis,trans*-2-(chloromethyl)hexahydro-4H-cyclohepta[d][1,3]dioxole (**S6**, 762.7 mg, 4.0 mmol, 1.0 equiv)**.** The crude product was purified by distillation under reduced pressure (0.5 mbar, bp 78°C) and obtained as a colorless oil in 38% yield (233.9 mg, 1.517 mmol). ^{1}H NMR (300 MHz, C_6D_6): δ = 0.71-0.85 (m, 2H), 0.95-1.17 (m, 2H), 1.32-1.60 (m, 6H), 3.59 (s, 2H), 3.98-4.06 (m, 2H). ^{13}C NMR (100 MHz, C_6D_6): δ = 23.57, 30.13, 30.64, 52.54, 80.27, 128.00, 164.45. HRMS (pos. APCI): calcd for $C_9H_{14}O_2$ $[M+H]^+$: 155.1067 found: 155.1066. MS (CI): m/z (%) = 190.1 (19), 173.1 (100), 171.1 (25), 155.1 (39) $[M+H]^+$, 130.1 (7), 112.1 (3). IR (ATR): ν [cm^{-1}] = 558, 565, 571, 580, 587, 670, 822, 1035, 1186, 1478, 1634, 1815, 1961, 2932, 3036, 3071, 3091.

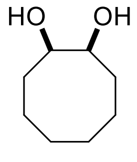

S7

1,2-*cis*-Cyclooctanediol. Synthesized from cyclooctene (2.3 ml, 1.96 g, 17.8 mmol, 1.0 equiv) following the general procedure for dihydroxylation of alkenes. The product was purified by column chromatography (cyclohexane/cetone, 2:1, Rf = 0.3) and obtained as a white solid in 81% yield (2.103 g, 14.58 mmol). The NMR data matched the literature values.[7] ^{1}H NMR (400 MHz, $CDCl_3$): δ = 1.45-1.58 (m, 6H), 1.63-1.74 (m, 4H), 1.86-1.96 (m, 2H), 2.08 (s, 2H), 3.88-3.92 (m, 2H). ^{13}C NMR (100 MHz, $CDCl_3$): δ = 23.89, 26.41, 30.32, 73.32.

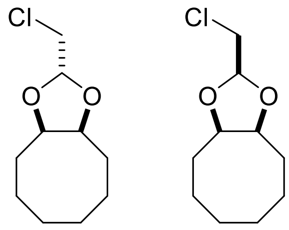

S8

***cis,cis*-2-(Chloromethyl)octahydrocycloocta[d][1,3]dioxole and *cis,trans*-2-(chloromethyl)octahydrocycloocta[d][1,3]dioxole.** Synthesized according to the general procedure for the synthesis of chloromethyldioxolanes 1,2-*cis*-cyclooctanediol (**S7**, 498.2 mg, 3.45 mmol, 1.0 equiv)**.** The crude product was purified by distillation under reduced pressure (0.05 mbar, bp 118°C) and obtained as a colorless oil in 88% yield as a mixture of diastereomers. (621 mg, 3.04 mmol). ^{1}H NMR (500 MHz, $CDCl_3$): δ = 1.25-1.51 (m, 6H), 1.60-1.68 (m, 2H), 1.77-1.89 (m, 4H), 3.45 (d, *J* = 4.4 Hz, 0.18H), 3.50 (d, *J* = 4.1 Hz, 1.72H), 4.05-4.11 (m, 1.71H), 4.17-4.22 (m, 0.16H), 4.97 (t, *J* = 4.1 Hz, 0.80H), 5.23 (t, *J* = 4.3 Hz, 0.08H). ^{13}C NMR (125 MHz, $CDCl_3$): δ = 25.32, 25.51, 26.65, 26.98, 27.82, 28.44, 44.87, 45.29, 80.12, 80.70, 99.85, 99.97. HRMS (pos. APCI): calcd for $C_{10}H_{17}ClO_2$ $[M+NH_4]^+$: 222.1255, found: 222.1252. MS (CI, 70 eV): m/z (%) = 224.1 (24), 222.1 (79) $[M+NH_4]^+$, 155.1 (100), 125.1 (5), 109.1 (50), 81.1 (8), 67.0 (8). IR (ATR): ν [cm^{-1}] = 814, 1013, 1419, 2930, 3123.

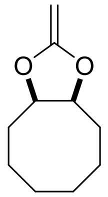

1d

***cis*-2-Methyleneoctahydrocycloocta[d][1,3]dioxole.** Synthesized according to the general procedure for the synthesis of cyclic ketene acetals from *cis,cis*-2-(chloromethyl)octahydrocycloocta[d][1,3]dioxole and *cis,trans*-2-(chloromethyl)octahydrocycloocta[d][1,3]dioxole (**S8**, 614.1 mg, 3.0 mmol, 1.0 equiv)**.** The crude product was purified by distillation under reduced pressure (0.5 mbar, bp 125°C) and obtained as a colorless solid in 58% yield (291.7 mg, 1.733 mmol). ^{1}H NMR (400 MHz, C_6D_6): δ = 0.73-0.82 (m, 2H), 0.84-1.03 (m, 4H), 1.14-1.22 (m, 2H), 1.57-1.9 (m, 4H), 3.60 (s, 2H), 3.89-3.94 (m, 2H). ^{13}C NMR (100 MHz, C_6D_6): δ = 25.28, 26.18, 27.25, 52.85, 80.85, 162.98. HRMS (pos. APCI): calcd for $C_{10}H_{16}O_2$ $[M+H]^+$: 169.1223, found: 169.1229. MS (CI, 70 eV): m/z (%) = 169.2 (100) $[M+H]^+$, 144.2 (6), 126.2 (2), 109.1 (4), 98.1 (1). IR (ATR): ν [cm^{-1}] = 817, 891, 1013, 1061, 1114, 1261, 1419, 1728, 2929, 3123.

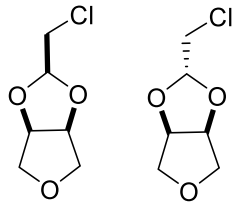

S9

***cis,cis*-2-(Chloromethyl)tetrahydrofuro[3,4-d][1,3]dioxole and *cis,trans*-2-(chloromethyl)tetrahydrofuro[3,4-d][1,3]dioxole.** Synthesized according to the general procedure for the synthesis of chloromethyldioxolanes from *meso*-3,4-dihydroxytetrahydrofuran (754 µL, 1.04 g, 10.0 mmol, 1.0 equiv). The crude product was purified by distillation under reduced pressure (0.5 mbar, bp 74°C) and obtained as a colorless oil in 87% yield as a mixture of diastereomers (1.437 g, 8.731 mmol). ^{1}H NMR (500 MHz,

C_6D_6): δ = 2.71-2.76 (m, 1.74H), 2.77-2.80 (m, 0.27H), 3.16 (d, *J* = 3.5 Hz, 0.26H), 3.36 (d, *J* = 4.3 Hz, 1.71H), 3.79-3.82 (m, 2H), 3.95-3.98 (m, 1.63H), 4.15-4.18 (m, 0.25H), 4.91 (t, *J* = 4.6 Hz, 0.8H), 5.26 (t, *J* = 3.5 Hz, 0.12H). ^{13}C NMR (125 MHz, C_6D_6): δ = 44.26, 44.53, 73.11, 73.96, 81.34, 81.85, 104.39, 105.48. HRMS (pos. APCI): calcd for $C_6H_9ClO_3$ $[M+NH_4]^+$: 182.0578 found: 182.0578. MS (CI): m/z (%) = 184.0 (33), 182.0 (100) $[M+NH_4]^+$, 115.0 (52), 69.0 (14). IR (ATR): ν [cm^{-1}] = 551, 534, 571, 574, 577, 581, 588, 595, 606, 611, 617, 620, 662, 730, 761, 839, 858, 907, 927, 983, 1017, 107, 1067, 1100, 1142, 1190, 1235, 1263, 1430, 1461, 2856, 2961.

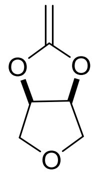

1e

***cis*-2-Methylenetetrahydrofuro[3,4-d][1,3]dioxole.** Synthesized according to the general procedure for the synthesis of cyclic ketene acetals from *cis,cis*-2-(chloromethyl)tetrahydrofuro[3,4-d][1,3]dioxole and *cis,trans*-2-(chloromethyl)tetrahydrofuro[3,4-d][1,3]dioxole (**S5**, 601.4 mg, 3.65 mmol, 1.0 equiv)**.** The crude product was purified by distillation under reduced pressure (0.5 mbar, bp 78°C) and obtained as a white solid in 39% yield (182.3 mg, 1.423 mmol.) ^{1}H NMR (400 MHz, C_6D_6): δ = 2.60-2.64 (m, 2H), 3.51 (s, 2H), 3.75-3.78 (m, 2H), 4.03-4.06 (m, 2H). ^{13}C NMR (100 MHz, C_6D_6): δ = 53.05, 73.17, 81.64, 165.39. HRMS (pos. APCI): calcd for $C_6H_8O_3$ $[M+H]^+$: 129.054 found: 129.0547. MS (CI): m/z (%) = 164.1 (78), 147.1 (100), 129.1 (27) $[M+H]^+$. IR (ATR): ν [cm^{-1}] = 562, 602, 674, 721, 867, 900, 938, 1036, 1098, 1166, 1238, 1374, 1479, 1739, 2855, 3036.

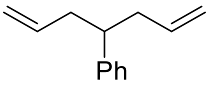

S10

4-Phenyl-1,6-heptadiene. Synthesized according to a literature procedure from benzaldehyde (2.653 g, 25.0 mmol, 1.0 equiv).[8] The product was purified by column chromatography (cyclohexane, R_f = 0.8) and obtained as a colorless oil in 51% yield (2.2057 g, 12.80 mmol). The NMR data matched the literature values. ^{1}H NMR (500 MHz, $CDCl_3$): δ = 2.33-2.46 (m, 4H), 2.69-2.75 (m, 1H), 4.92-4.99 (m, 4H), 5.63-5.71 (m, 2H), 7.15-7.21 (m, 3H), 7.28-7.31 (m, 2H). ^{13}C NMR (125 MHz, $CDCl_3$): δ = 40.44, 45.75, 116.21, 126.24, 127.88, 128.38, 136.93, 144.78.

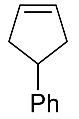

S11

Cyclopent-3-en-1-ylbenzene. Synthesized according to a modified literature procedure.[8] A 250 ml two necked flask was charged with Grubbs I catalyst (197.5 mg, 0.240 mmol, 2 mol%). A solution of 4-phenyl-1,6-heptadiene (**S10**, 2.067 g, 12.0 mmol, 1.0 equiv) in degassed CH_2Cl_2 (120 ml) was added and the mixture was stirred under reflux overnight. Afterwards, it was allowed to cool to room temperature and the solvent was removed under reduced pressure. The residue was purified by column chromatography (cyclohexane, R_f = 0.9) and obtained as a colorless oil in 65% yield (1.122 g, 7.780 mmol). The NMR data matched the literature values. ^{1}H NMR (300 MHz, $CDCl_3$): δ = 2.41-2.51 (m, 2H), 2.78-2.90 (m, 2H), 3.43-3.53 (m, 1H), 5.78-5.82 (m, 2H), 7.14-7.20 (m, 1H), 7.22-7.31 (m, 4H). ^{13}C NMR (100 MHz, $CDCl_3$): δ = 41.47, 43.35, 125.95, 127.08, 128.52, 130.01, 147.63.

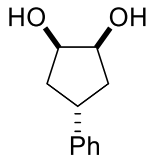

S12

***cis,trans*-4-Phenylcyclopentane-1,2-diol.** Synthesized from cyclopent-3-en-1-ylbenzene (**S11**, 810.0 mg, 5.616 mmol, 1.0 equiv) following the general procedure for dihydroxylation of alkenes. The product was purified by column chromatography (cyclohexane/EtOAc, 9:1, Rf = 0.3) and obtained as white crystals in 85% yield (850.3 mg, 4.769 mmol). The relative configuration was determined by X-ray structure analysis. ^{1}H NMR (400 MHz, $CDCl_3$): δ = 1.89-1.99 (m, 2H), 2.16-2.25 (m, 2H), 2.59 (s, 2H), 3.61 (tt, *J* = 8.8, 8.8 Hz, 1H), 4.33-4.37 (m, 2H), 7.17-7.21 (m, 3H), 7.28-7.33 (m, 2H). ^{13}C NMR (100 MHz, $CDCl_3$): δ = 40.64, 40.68, 74.10, 126.18, 127.03, 128.64, 145.93. HRMS (pos. APCI): calcd for $C_{11}H_{14}O_2$ $[M+NH_4]^+$: 196.1332 found: 196.1322. MS (CI): m/z (%) = 196.1 (100) $[M+NH_4]^+$, 194.1 (18), 177.2 (2), 160.1 (13), 142.1 (11), 133.1 (3) 104.1 (2), 104.1 (2), 78.1 (2). Melting point: 91°C. IR (ATR): ν [cm^{-1}] = 559, 572, 575, 578, 581, 588, 674, 697, 754, 793, 868, 901, 915, 948, 958, 1002, 1014, 1025, 1043, 1070, 1086, 1107, 1121, 1163, 1180, 1210, 1277, 1326, 1345, 1433, 1444, 1455, 1479, 1490, 2943, 2965, 3029, 3294, 3417.

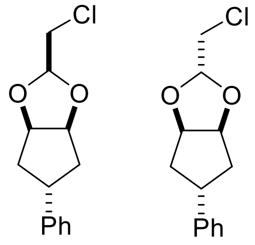

S13

***cis,cis,trans*-2-(Chloromethyl)-5-phenyltetrahydro-4H-cyclopenta[d][1,3]-dioxole and *cis,trans,trans*-2-(chloromethyl)-5-phenyltetrahydro-4H-cyclopenta[d][1,3]-dioxole.** Synthesized from *cis,trans*-4-phenylcyclopentane-1,2-diol (**S12**, 750 mg, 4.21 mmol, 1.0 equiv) following the general procedure for the synthesis of chloromethyldioxolanes. The product was purified by column chromatography (cyclohexane/EtOAc, 7:3, Rf = 0.7) and obtained as a yellow oil in 98% yield (981.5 mg, 4.112 mmol) as a mixture of diastereomers. ^{1}H NMR (500 MHz, C_6D_6): δ = 1.16-1.24 (m, 2H), 2.07-2.11 (m, 0.16H), 2.14-2.18 (m, 1.77H), 3.13 (d, *J* = 4.1 Hz, 0.15H), 3.18-3.25 (m, 0.08H), 3.36 (d, *J* = 3.7 Hz, 1.75H), 3.43-3.51 (m, 0.86H), 4.15-4.18 (m, 1.74H), 4.36-4.39 (m, 0.14H), 4.70 (t, *J* = 3.8 Hz, 0.83H), 5.27 (t, *J* = 4.0 Hz, 0.07H), 6.93-7.00 (m, 2H), 7.04-7.08 (m, 1H), 7.11-7.15 (m, 2H). ^{13}C NMR (125 MHz, C_6D_6): δ = 39.25, 40.54, 41.14, 42.08, 43.86, 45.38, 81.54, 81.71, 101.98, 104.16, 126.51, 126.55, 127.26, 127.40, 128.29, 128.61, 142.95, 143.03. HRMS (pos. APCI): calcd for $C_{13}H_{15}ClO_2$ $[M+NH_4]^+$: 256.1099 found: 256.1099. MS (CI): m/z (%) = 256.1 (2) $[M+NH_4]^+$, 237 (1), 203.1 (24), 189.1 (100), 160.1 (15), 143.1 (70), 142.1 (38), 128.1 (6), 104.1 (4), 78.1 (4). IR (ATR): ν [cm^{-1}] = 553, 562, 573, 578, 584, 588, 598, 667, 1035, 1478, 1815, 1961, 3036, 3071, 3091.

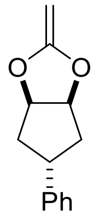

1f

***cis,trans*-2-Methylene-5-phenyltetrahydro-4H-cyclopenta[d][1,3]dioxole.** Synthesized from *cis,cis,trans*-2-(chloromethyl)-5-phenyltetrahydro-4H-cyclopenta[d][1,3]-dioxole and *cis,trans,trans*-2-(chloromethyl)-5-phenyltetrahydro-4H-cyclopenta[d][1,3]-dioxole (**S13**, 944.6 mg, 3.957 mmol, 1.0 equiv) following the general procedure for the synthesis of cyclic ketene acetals. The product was purified by distillation under reduced pressure (0.5 mbar, bp = 121°C) and obtained as a white solid in 43% yield (344.6 mg, 1.704 mmol). The material contained about 5% impurities, but was suitable for the catalytic β-O-elimination reaction. ^{1}H NMR (500 MHz, C_6D_6): δ = 1.06-1.13 (m, 2H), 2.04-2.08 (m, 2H), 3.20-3.28 (m, 1H), 3.58 (s, 2H), 4.32-4.35 (m, 2H), 6.78-6.80 (m, 2H), 7.01-7.14 (m, 3H). ^{13}C NMR (125 MHz, C_6D_6): δ = 40.98, 41.09, 52.49, 82.40, 126.68, 127.18, 128.60, 142.03, 165.75. HRMS (pos. APCI): calcd for $C_{13}H_{14}O_2$ $[M+H]^+$: 203.1067 found: 203.1069. MS (CI): m/z (%) = 203.1 (2) $[M+H]^+$, 202.1 (5), 184.2 (94), 157.2 (12), 156.2 (100), 142.2 (7). IR (ATR): ν [cm^{-1}] = 558, 562, 564, 571, 578, 581, 588, 596, 604, 755, 833, 898, 923, 940, 1034, 1088, 1115, 1136, 1173, 1195, 1211, 1241, 1373, 1389, 1448, 1495, 1603, 1735, 2960, 3027.

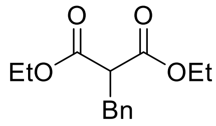

S14

Diethyl 2-benzylmalonate.[9] Synthesized according to the literature procedure from diethylmalonate (10.0 g, 9.5 ml, 62.5 mmol, 1.0 equiv). The crude product was purified by column chromatography (cyclohexane/EtOAc, 19:1, R_f = 0.3) and obtained as a colorless oil in 83% yield (13.0 g, 51.9 mmol). The NMR data matched the literature values. ^{1}H NMR (400MHz, $CDCl_3$): δ = 1.21 (t, *J* = 7.2 Hz, 6H), 3.22 (d, *J* = 7.8 Hz, 2H), 3.64 (t, *J* = 7.7 Hz, 1H), 4.10-4.21 (m, 4H), 7.18-7.23 (m, 3H), 7.25-7.30 (m, 2H). ^{13}C NMR (100 MHz, $CDCl_3$): δ = 14.15, 34.86, 54.02, 61.57, 126.86, 128.63, 128.99, 138.09, 169.00.

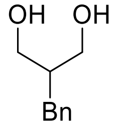

S15

2-Benzylpropane-1,3-diol.[10] Synthesized according to the literature procedure from diethyl 2-benzylmalonate (**S14**, 13.0 g, 51.9 mmol, 1.0 equiv). The crude product was purified by column chromatography (EtOAc, R_f = 0.3) and obtained as a white solid in 67% yield (5.81 g, 34.9 mmol). The NMR data matched the literature values. ^{1}H NMR (400MHz, $CDCl_3$): δ = 2.02-2.11 (m, 1H), 2.34 (s, 2H), 2.63 (d, *J* = 7.6 Hz, 2H), 3.68 (dd, *J* = 7.0, 10.6 Hz, 2H), 3.80 (dd, *J* = 3.9, 10.7 Hz, 2H), 7.17-7.23 (m, 3H), 7.27-7.31 (m, 2H). ^{13}C NMR (100 MHz, $CDCl_3$): δ = 34.44, 44.05, 65.69, 126.30, 128.60, 129.13, 140.00.

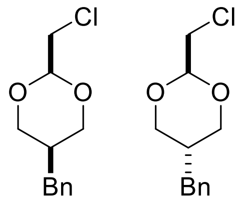

S16

***cis*-5-Benzyl-2-(chloromethyl)-1,3-dioxane and *trans*-5-benzyl-2-(chloromethyl)-1,3-dioxane.** Synthesized according to the general procedure for the synthesis of chloromethyldioxolanes from 2-benzylpropane-1,3-diol. (**S15**, 1.5 g, 9.0 mmol, 1.0 equiv) The product was purified by distillation under reduced pressure (0.5 mbar, bp 170°C) and obtained as a colorless oil in 83% yield as a mixture of diastereomers (1.694 g, 7.472 mmol). ^{1}H NMR (400MHz, C_6D_6): δ = 0.93-0.98 (m, 0.46H), 1.79 (d, *J* = 7.5 Hz, 1.17H), 2.00-2.12 (m, 0.56H), 2.87 (d, *J* = 8.1 Hz, 0.92H), 2.92-2.98 (m, 1.19H), 3.28-3.29 (m, 0.42H), 3.31-3.32 (m, 0.51H), 3.37-3.38 (m, 2.03H), 3.57-3.58 (m, 0.49H), 3.60-3.61 (m, 0.41H), 3.75-3.79 (m, 1.18H), 4.38 (t, *J* = 4.6 Hz, 0.56 H), 4.41 (t, *J* = 4.6 Hz, 0.44 H), 6.73-6.76 (m, 1.10H), 7.00-7.09 (m, 3.05H), 7.11-7.14 (m, 0.76H). ^{13}C NMR (100 MHz, C_6D_6): δ = 34.56, 35.56, 35.74, 36.27, 44.67, 44.80, 69.29, 71.63, 100.57, 100.87, 126.37, 126.54, 128.69, 128.74, 129.58, 138.34, 140.60. HRMS (pos. APCI): calcd for $C_{12}H_{15}ClO_2$ $[M+H]^+$: 227.0833, found: 227.0837. MS (CI, 70 eV): m/z (%) = 246.1 (32), 244.1 $[M+NH_4]^+$ (100), 177.1 (36), 131.1 (33), 91.1 (19). IR (ATR): ν [cm^{-1}] = 606, 614, 1037, 1046, 1056, 1141, 1241, 1367, 1384, 1670, 1738, 2928.

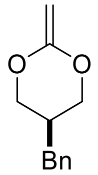

1g

5-Benzyl-2-methylene-1,3-dioxane. Synthesized according to the general procedure for the synthesis of cyclic ketene acetals from *cis*-5-benzyl-2-(chloromethyl)-1,3-dioxane and *trans*-5-benzyl-2-(chloromethyl)-1,3-dioxane (**S16**, 1.0 g, 4.41 mmol, 1.0 equiv) The product was purified by distillation under reduced pressure (0.5 mbar, bp 160°C) and obtained as a colorless oil in 73% yield (612.3 mg, 3.219 mmol). ^{1}H NMR (500 MHz, C_6D_6): δ = 1.80-1.88 (m, 1H), 2.14 (d, *J* = 7.8 Hz, 2H), 3.36 (dd, *J* = 7.2, 10.8 Hz, 2H), 3.65 (dd, *J* = 5.2, 10.8 Hz, 2H), 3.86 (s, 2H), 6.75-6.77 (m, 2H), 7.00-7.08 (m, 3H).^{13}C NMR (125 MHz, C_6D_6): δ = 35.54, 67.93, 69.10, 126.60, 128.29, 128.73, 128.97, 138.60, 161.40. HRMS (pos. APCI): calcd for $C_{12}H_{14}O_2$ $[M+H]^+$: 191.1067, found: 191.1066. MS (CI, 70 eV): m/z (%) = 223.1 (16), 192.1 (13), 191.1 (100), 131.1 (5), 118.1 (8), 91.1 (4). IR (ATR): ν [cm^{-1}] = 606, 617, 622, 701, 742, 1037, 1141, 1086, 1242, 1367, 1385, 1670, 1738, 2923.

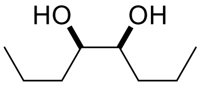

S17

1,2-*cis*-Octanediol. Synthesized from (*Z*)-oct-4-ene (2.8 ml 2.0 g, 18.0 mmol, 1.0 equiv) following the general procedure for dihydroxylation of alkenes. The product was purified by column chromatography (cyclohexane/EtOAc, 4:1, R_f = 0.2) and obtained as a white solid in 62% yield (1.638 g, 11.20 mmol). The NMR data matched the literature values.[11] ^{1}H NMR (400 MHz, $CDCl_3$): δ = 0.95 (t, *J* = 7.2 Hz, 6H), 1.30-1.47 (m, 6H), 1.49-1.60 (m, 2H), 1.87 (s, 2H), 3.60-3.64 (m, 2H). ^{13}C NMR (100 MHz, $CDCl_3$): δ = 14.22, 19.33, 33.57, 74.60.

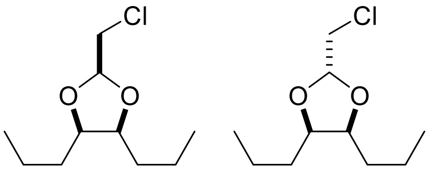

S18

***cis,cis*-2-(Chloromethyl)-4,5-dipropyl-1,3-dioxolane and *cis*,*trans*-(chloromethyl)-4,5-dipropyl-1,3-dioxolane.** Synthesized according to the general procedure for the synthesis of chloromethyldioxolanes from 1,2-*cis*-octanediol (**S17**, 731.3 mg, 5.0 mmol, 1.0 equiv). The product was purified by distillation under reduced pressure (0.5 mbar, bp 83°C) and obtained as a colorless oil in 92% yield as a mixture of diastereomers (945.9 mg, 4.579 mmol). ^{1}H NMR (400 MHz, C_6D_6): δ = 0.85 and 0.86 (2 x t, *J* = 7.3 Hz and *J* = 7.3 Hz, 6H), 0.96-1.08 (m, 2H), 1.19-1.30 (m, 2H), 1.32-1.60 (m, 4H), 3.23 (d, *J* = 3.9 Hz, 0.64H), 3.30 (d, *J* = 3.9 Hz, 1.28H), 3.64-3.70 (m, 1.25H), 3.83-3.88 (m, 0.65H), 4.94 (t, *J* = 3.9 Hz, 0.60H), 5.14 (t, *J* = 3.9 Hz, 0.32H). ^{13}C NMR (100 MHz, C_6D_6): δ = 14.11, 14.12, 19.71, 19.75, 30.92, 32.01, 45.12, 45.71, 78.77, 78.99, 101.02, 101.87. HRMS (pos. APCI): calcd for $C_{10}H_{19}ClO_2$ $[M+NH_4]^+$: 224.1412 found: 224.1411. MS (CI): m/z (%) = 224.1 (15) $[M+NH_4]^+$, 205.1 (33), 171.1 (19), 157.1 (100), 99.1 (5). IR (ATR): ν [cm^{-1}] = 555, 574, 677, 761, 841, 1012, 1131, 1378, 1429, 1467, 1694, 2213, 2873, 2959.

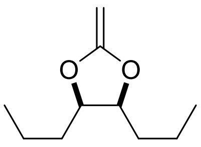

1h

***cis*-2-Methylene-4,5-dipropyl-1,3-dioxolane.** Synthesized according to the general procedure for the synthesis of cyclic ketene acetals from *cis,cis*-2-(chloromethyl)-4,5-dipropyl-1,3-dioxolane and *cis*,*trans*-(chloromethyl)-4,5-dipropyl-1,3-dioxolane (**S18**, 826.8 mg, 4.0 mmol, 1.0 equiv) The product was purified by distillation under reduced pressure (0.5 mbar, bp 71°C) and obtained as a colorless oil in 46% yield (313.3 mg, 1.840 mmol). ^{1}H NMR (400 MHz, C_6D_6): δ = 0.77 (t, *J* = 7.3 Hz, 6H), 0.86-0.94 (m, 2H), 1.10-1.23 (m, 2H), 1.30-1.50 (m, 4H), 3.62 (s, 2H), 3.81-3.87 (m, 2H). ^{13}C NMR (100 MHz, C_6D_6): δ = 13.90, 19.40, 30.57, 54.03, 79.48, 163.75. HRMS (pos. APCI): calcd for $C_{10}H_{18}O_2$ $[M+H]^+$: 171.1380 found: 171.1381. MS (CI): m/z (%) = 206.2 (9), 204.2 (9), 189.2 (38), 187.2 (24), 172.2 (12), 171.2 (100) $[M+H]^+$, 146.2 (9), 127.2 (3). IR (ATR): ν [cm^{-1}] = 553, 559, 565, 569, 572, 577, 584, 588, 595, 604, 609, 614, 617, 1027, 1242, 1378, 1736, 2874, 2936, 2959.

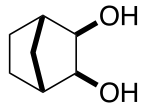

S19

exo,exo-Bicyclo[2.2.1]heptane-2,3-diol. Synthesized from norbornene (1.694 g, 18.0 mmol, 1.0 equiv) following the general procedure for dihydroxylation of alkenes. The product was purified by column chromatography (cyclohexane/EtOAc, 4:1, R_f = 0.2) and obtained as a white solid in 91% yield (2.096 g, 16.35 mmol). The NMR data matched the literature values.[12] ^{1}H NMR (400 MHz, $CDCl_3$): δ = 1.01-1.11 (m, 3H), 1.42-1.49 (m, 2H), 1.73-1.77 (m, 1H), 2.12-2.14 (m, 2H), 2.94 (s, 2H), 3.67 (d, *J* = 1.6 Hz, 1H). ^{13}C NMR (100 MHz, $CDCl_3$): δ = 24.67, 31.72, 43.24, 75.00.

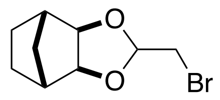

S20

***exo*-2-(Bromomethyl)hexahydro-4,7-methanobenzo[d][1,3]-dioxole.** Synthesized according to the general procedure for the synthesis of chloromethyldioxolanes from 2-exo-3-exo- bicyclo[2.2.1]heptane-2,3-diol (**S19**, 2.08 g, 16.2 mmol, 1.0 equiv) with the following exception: 2-bromo-1,1-dimethoxyethane (1.9 ml, 2.7 g, 16.2 mmol, 1.0 equiv) was used instead of 2-chloro-1,1-dimethoxyethane. The product was purified by column chromatography (cyclohexane/EtOAc, 4:1, R_f = 0.9) and obtained as a yellow oil in 99% yield (3.7 g, 16.0 mmol). ^{1}H NMR (500 MHz, C_6D_6): δ = 0.45-0.50 (m, 2H), 0.75-0.78 (m, 1H), 1.01-1.07 (m, 2H), 1.79-1.83 (m, 1H), 2.13-2.14 (m, 2H), 3.15 (d, *J* = 4.1 Hz, 2H), 3.50 (d, *J* = 1.5 Hz, 2H), 4.62 (t, *J* = 4.1 Hz, 1H). ^{13}C NMR (125 MHz, C_6D_6): δ = 22.92, 30.85, 31.81, 39.95, 83.29, 101.22. HRMS (pos. APCI): calcd for $C_9H_{13}O_2Br$ $[M+H]^+$: 233.0172 found: 233.0174. MS (CI): m/z (%) = 233.1 (1) $[M+H]^+$, 230.9 (1), 153.1 (6), 140.1 (8), 139.1 (100), 110.1 (3), 93.1 (9), 81.1 (3), 67.1 (2).). IR (ATR): ν [cm^{-1}] = 551, 555, 559, 563, 567, 570, 575, 579, 593, 596, 600, 602, 607, 617, 621, 634, 676, 703, 730, 768, 833, 872, 886, 918, 1005, 1028, 1140, 1171, 1223, 1284, 1300, 1394, 1425, 1450, 1477, 1736, 2162, 2876, 2966, 3525.

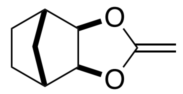

1i

***exo*-Methylenehexahydro-4,7-methanobenzo[d][1,3]dioxole.** Synthesized according to the general procedure for the synthesis of cyclic ketene acetals from *exo*-2-(bromomethyl)hexahydro-4,7-methanobenzo[d][1,3]-dioxole (**S20**, 932.4 mg, 4.0 mmol, 1.0 equiv) The product was purified by distillation under reduced pressure (0.5 mbar, bp 84°C) and obtained as a colorless oil in 63% yield (383.3 mg, 2.517 mmol). ^{1}H NMR (400 MHz, C_6D_6): δ = 0.33-0.39 (m, 2H), 0.71-0.76 (dp, *J* = 1.5, 11.0 Hz, 1H), 0.85-0.92 (m, 2H), 1.63-1.68 (m, 1H), 2.05-2.07 (m, 2H), 3.51 (s, 2H), 3.77 (d, *J* = 1.6 Hz, 2H). ^{13}C NMR (100 MHz, C_6D_6): δ = 22.59, 31.11, 41.02, 51.82, 83.68, 166.08. HRMS (pos. APCI): calcd for $C_9H_{12}O_2$ $[M+H]^+$: 153.0910 found: 153.0912 MS (CI): m/z (%) = 153.1 (100) $[M+H]^+$, 152.1 (7), 110.0 (1), 93.1 (1). IR (ATR): ν [cm^{-1}] = 555, 559, 561, 567, 570, 574, 579, 583, 587, 593, 596, 599, 604, 607, 804, 966, 1054, 1081, 1141, 1188, 1261, 1378, 1462, 2854, 2924.

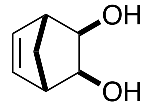

S21

***exo,exo*-Bicyclo[2.2.1]hept-5-ene-2,3-diol.** Synthesized from norbornadiene (1.659 g, 18.0 mmol, 1.0 equiv) following the general procedure for dihydroxylation of alkenes. The product was purified by column chromatography (cyclohexane/EtOAc, 1:1, R_f = 0.6) and obtained as a white solid in 91% yield (2.065 g, 16.37 mmol). The NMR data matched the literature values.[13] ^{1}H NMR (400 MHz, $CDCl_3$): δ = 1.51-1.55 (m, 1H), 1.77-1.80 (m, 1H), 2.58-2.60 (m, 2H), 2.82 (s, 2H), 3.60-3.61 (t, *J* = 1.8 Hz, 2H). ^{13}C NMR (100 MHz, $CDCl_3$): δ = 93.14, 98.95, 119.89, 187.33.

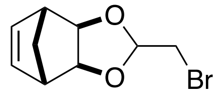

S22

***exo*-2-(Bromomethyl)-3a,4,7,7a-tetrahydro-4,7-methanobenzo[d][1,3]dioxole.**
Synthesized according to the general procedure for the synthesis of chloromethyldioxolanes

from exo,exo-bicyclo[2.2.1]hept-5-ene-2,3-diol (**S21**, 2.06 g, 16.3 mmol, 1.0 equiv) with the following exception: 2-bromo-1,1-dimethoxyethane (1.9 ml, 2.8 g, 16.3 mmol, 1.0 equiv) was used instead of 2-chloro-1,1-dimethoxyethane. The product was purified by column chromatography (cyclohexane/EtOAc, 4:1, R_f = 0.9) and obtained as a yellow oil in 96% yield (3.60 g, 15.6 mmol). ^{1}H NMR (400 MHz, C_6D_6): δ = 1.55-1.59 (m, 1H), 2.11-2.14 (m, 1H), 2.61-2.62 (m, 2H), 3.14 (d, *J* = 4.0 Hz, 2H), 3.83 (d, *J* = 1.5 Hz, 1H), 4.92 (t, *J* = 4.0 Hz, 1H), 5.63 (t, *J* = 1.9 Hz, 2H). ^{13}C NMR (100 MHz, C_6D_6): δ = 30.99, 43.35, 45.37, 81.67, 105.71, 136.89. HRMS (pos. APCI): calcd for $C_9H_{11}O_2Br$ $[M+NH_4]^+$: 248.0281 found: 248.0278. MS (CI): m/z (%) = 248.0 (4) $[M+NH_4]^+$, 203.0 (63), 201.0 (64), 166.0 (100), 163.9 (99), 151.1 (51), 137.1 (10), 108.1 (21), 84.0 (30), 71.0 (31). IR (ATR): ν [cm^{-1}] = 551, 555, 559, 566, 576, 579, 582, 586, 591, 595, 600, 604, 607, 684, 697, 800, 1087, 1262, 1496, 1725, 2158, 2882

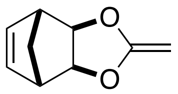

1j

***exo*-2-Methylene-3a,4,7,7a-tetrahydro-4-exo,7-exo-methanobenzo[d]-[1,3]dioxole.** Synthesized according to the general procedure for the synthesis of cyclic ketene acetals from *exo*-2-(bromomethyl)-3a,4,7,7a-tetrahydro-4,7-methanobenzo-[d][1,3]dioxole (**S22**, 693.3 mg, 4.0 mmol, 1.0 equiv) The product was purified by distillation under reduced pressure (0.5 mbar, bp 77°C) and obtained as a colorless oil in 78% yield (349.5 mg, 2.327 mmol). ^{1}H NMR (500 MHz, C_6D_6): δ = 1.42-1.46 (dp, *J* = 1.7, 9.9 Hz, 1H), 1.87-1.89 (m, 1H), 2.56-2.57 (m, 2H), 3.51 (s, 2H), 4.00 (d, *J* = 1.7 Hz, 2H), 5.42 (m, 2H). ^{13}C NMR (125 MHz, C_6D_6): δ = 41.91, 46.66, 52.59, 81.79, 136.41, 168.72. HRMS (pos. APCI): calcd for $C_9H_{10}O_2$ $[M+H]^+$: 151.0754 found: 151.0755. MS (CI): m/z (%) = 151.1 (100) $[M+H]^+$, 150.1 (6), 109.1 (1), 84.0 (1). IR (ATR): ν [cm^{-1}] = 559, 567, 574, 579, 600, 715, 785, 804, 905, 929, 1019, 1036, 1077, 1134, 1161, 1251, 1331, 1373, 1479, 1738, 1815, 2985, 3036, 3091, 3071.

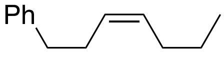

S23

(*Z*)-Hept-3-en-1-ylbenzene. Butyltriphenylphosphonium bromide (11.98 g, 30.0 mmol, 1.2 equiv) was suspended in THF (50 ml). The solution was cooled to 0°C (ice bath) and KO*t*-Bu (3.37 g, 30.0 mmol, 1.2 equiv) was added portion wise. The resulting orange solution was stirred at 0°C for another hour before 3-phenylpropanal (3.1 ml, 3.4 g, 25.0 mmol, 1.0 equiv) was added. Afterwards, the solution was allowed to stir at room temperature overnight. Then, the reaction mixture was quenched with sat. NH_4Cl solution (50 ml). The layers were separated and the aqueous layer was extracted with Et_2O (3 x 50 ml). The combined organic layers were washed with brine (50 ml), dried over Na_2SO_4, filtered and the solvent was removed under reduced pressure. The product was purified by column chromatography (cyclohexane, R_f = 0.9) and obtained as a colorless oil in 87% yield (3.80 g, 21.79 mmol) as a 1:16 E/Z mixture. The NMR data matched the literature values.[14] ^{1}H NMR (400 MHz, $CDCl_3$): δ = 0.88 (t, *J* = 7.5 Hz, 3H), 1.28-1.39 (m, 2H), 1.94-2.01 (m, 2H), 2.30-2.39 (m, 2H), 2.65-2.69 (m, 2H), 5.36-5.46 (m, 2H), 7.16-7.21 (m, 3H), 7.27-7.30 (m, 2H). ^{13}C NMR (100 MHz, $CDCl_3$): δ = 13.76, 13.89, 22.82, 22.91, 29.33, 29.45, 34.59, 34.82, 36.21, 36.35, 125.83, 125.90, 128.37, 128.40, 128.60, 128.63, 129.01, 129.66, 130.64, 131.10, 142.34.

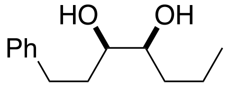

S24

1-Phenylheptane-*cis*-3,4-diol. Synthesized from (*Z*)-hept-3-en-1-ylbenzene (**S23**, 3.137 g, 18.0 mmol, 1.0 equiv) following the general procedure for dihydroxylation of alkenes. The product was purified by column chromatography (cyclohexane, R_f = 0.6) followed by recrystallization from toluene and obtained as a white solid in 59% yield (2.228 g, 10.70 mmol).

^{1}H NMR (400 MHz, $CDCl_3$): δ = 0.93 (t, *J* = 7.2 Hz, 3H), 1.26-1.58 (m, 4H), 1.75-1.81 (m, 2H), 1.81-1.88 (m, 2H), 2.64-2.71 (m, 1H), 2.85-2.92 (m, 1H), 3.59-3.65 (m, 2H), 7.17-7.23 (m, 3H), 7.27-7.31 (m, 2H). ^{13}C NMR (100 MHz, $CDCl_3$): δ = 14.18, 19.29, 32.43, 33.04, 33.70, 74.13, 74.65, 126.07, 128.59, 142.11. HRMS (pos. APCI): calcd for $C_{13}H_{20}O_2$ $[M+NH_4]^+$: 226.1802 found: 226.1803. MS (CI): m/z (%) = 226.1 (100) $[M+NH_4]^+$, 209.2 (62), 208.2 (38), 207.1 (65), 191.1 (68), 189.1 (38), 173.1 (65), 152.1 (7), 134.1 (6), 104.1 (8), 91.1 (19), 78.1 (4). IR (ATR): ν [cm^{-1}] = 565, 573, 578, 581, 583, 587, 603, 606, 676, 696, 744, 849, 920, 947, 1010, 1030, 1055, 1121, 1433, 1454, 1464, 1496, 2872, 2908, 2935, 2955, 3028, 3302.

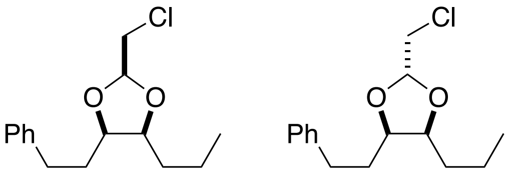

S25

***cis,cis*-2-(Chloromethyl)-4-phenethyl-5-propyl-1,3-dioxolane and *cis,trans*-2-(chloromethyl)-4-phenethyl-5-propyl-1,3-dioxolane.** Synthesized according to the general procedure for the synthesis of chloromethyldioxolanes from 1-Phenylheptane-*cis*-3,4-diol (**S24**, 2.7 g, 13.0 mmol, 1.0 equiv). The product was purified by column chromatography (cyclohexane/EtOAc, 9:1, R_f = 0.8) and obtained as a yellow oil in 96% yield (3.34 g, 12.4 mmol) as a mixture of diastereomers (945.9 mg, 4.579 mmol). ^{1}H NMR (400 MHz, C_6D_6): δ = 0.81 (t, *J* = 7.3 Hz, 3H), 0.87-1.00 (m, 1H), 1.14-1.53 (m, 4H), 1.60-1.77 (m, 1H), 2.50-2.60 (m, 1H), 2.74-2.87 (m, 1H), 3.22 (d, *J* = 3.9 Hz, 0.64H), 3.31 (d, *J* = 3.8 Hz, 1.35H), 3.57-3.69 (m, 1.31H), 3.76-3.87 (m, 0.65H), 4.92 (t, *J* = 3.8 Hz, 0.63H), 5.16 (t, *J* = 3.9 Hz, 0.31H). ^{13}C NMR (100 MHz, C_6D_6): δ = 14.06, 19.72, 19.83, 30.81, 30.93, 31.73, 32.12, 32.59, 45.04, 45.61, 77.93, 77.95, 78.64, 79.10, 101.09, 101.82, 126.24, 126.28, 127.88, 128.70, 128.85, 128.88, 141.95, 142.08. HRMS (pos. APCI): calcd for $C_{15}H_{21}O_2Cl$ $[M+NH_4]^+$: 268.1568 found: 286.1567. MS (CI): m/z (%) = 288.1 (31), 286.1 (100), 219.1 (64), 208.1 (22), 191.1 (29), 173.1 (70), 152.0 (5), 117.1 (12), 104.0 (21), 91.1 (31), 65.0 (5). IR (ATR): ν [cm^{-1}] = 565, 583, 588, 314, 617, 664, 700, 752, 908, 1029, 1132, 1351, 1376, 1428, 1455, 1496, 2872, 2958, 3027.

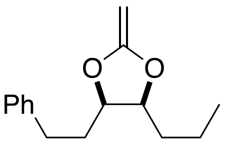

rac-**3**

***cis*-2-Methylenehexahydro-4,7-methanobenzo[d][1,3]dioxole.** Synthesized according to the general procedure for the synthesis of cyclic ketene acetals from *cis,cis*-2-(chloromethyl)-4-phenethyl-5-propyl-1,3-dioxolane and *cis,trans*-2-(chloromethyl)-4-phenethyl-5-propyl-1,3-dioxolane (**S25**, 1.08 g, 4.0 mmol, 1.0 equiv) The product was purified by distillation under reduced pressure (0.5 mbar, bp 131°C) and obtained as a colorless solid in 69% yield (640.7 mg, 2.757 mmol). ^{1}H NMR (400 MHz, C_6D_6): δ = 0.73 (t, *J* = 7.5 Hz, 3H), 0.76-0.83 (m, 1H), 1.07-1.17 (m, 1H), 1.19-1.44 (m, 3H), 1.62-1.72 (m, 1H), 2.44-2.52 (m, 1H), 2.70-2.77 (m, 1H), 3.64-3.67 (m, 2H), 3.74-3.79 (m, 1H), 3.83-3.88 (m, 1H), 7.01-7.09 (m, 3H), 7.13-7.15 (m, 2H). ^{13}C NMR (100 MHz, C_6D_6): δ = 13.86, 19.44, 30.53, 32.20, 54.24, 78.56, 79.45, 126.38, 128.74, 128.82, 141.44, 163.64. HRMS (pos. APCI): calcd for $C_{15}H_{20}O_2$ $[M+H]^+$: 233.1536 found: 233.1537. MS (CI): m/z (%) = 233.1 (3) $[M+H]^+$, 209.2 (13), 208.1 (100), 206.2 (3), 191.1 (68), 189.1 (9), 173.1 (9), 148.1 (2), 133.2 (1), 104.1 (4), 91.1 (3). IR (ATR): ν [cm^{-1}] = 554, 566, 571, 583, 587, 606, 677, 699, 748, 812, 950, 1029, 1095, 1238, 1378, 1454, 1480, 1496, 1604, 1682, 1734, 2280, 2872, 2958, 3028, 3478.

Zr-Catalyzed Desymmetrization of Cyclic Ketene Acetals

General Procedure

A flame dried and argon filled 10 ml Young tube equipped with a magnetic stir bar was charged with (*R,R*)-(ebthi)$ZrCl_2$ (2.1 mg, 5 µmol, 2.5 mol%). A solution of $LiAlH_4$ in 2-MeTHF (2.3 M, 0.13 ml, 0.3 mmol, 1.5 equiv) was added dropwise. Afterwards, the desired substrate (0.2 mmol, dissolved in 0.2 ml of 2-MeTHF) and *N*-methylpyrrolidine (10.0 µl, 8.5 mg, 0.1 mmol, 0.5 equiv) were added dropwise. The tube was sealed and the reaction mixture was stirred for 4h at room temperature. The reaction was quenched by dropwise addition of 10% (w/w) aq. Rochelle salt solution (2 ml) and CH_2Cl_2 (2 ml). The reaction mixture was transferred into a separatory funnel, the layers were separated and the aqueous layer was extracted with CH_2Cl_2 (3 × 5 ml). The combined organic layers were dried over Na_2SO_4, filtered, and the solvent was removed under reduced pressure. The crude product was purified as described individually.

Racemic products were synthesized using *rac*-(ebthi)$ZrCl_2$ as catalyst precursor.

Purification and Characterization Products

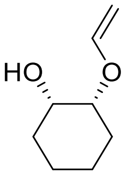

(*S,R*)-**2a**

(1*S*,2*R*)-2-(Vinyloxy)cyclohexan-1-ol. Synthesized from *cis*-2-methylenehexahydrobenzo[d][1,3]dioxole (**1a**) following the general catalytic desymmetrization procedure. The product was purified by column chromatography (*n*-pentane/CH_2Cl_2 3:1 containing 1% (v/v) NEt_3, R_f = 0.3) and obtained as a colorless liquid in 82% yield (23.4 mg, 0.165 mmol) and 92% ee. $[\alpha]_D^{23} = -12.8°$ (*c* 0.05, C_6H_6). ^1H NMR (400 MHz, C_6D_6): δ = 0.92-1.09 (m, 2H), 1.22-1.32 (m, 2H), 1.38-1.47 (m, 1H), 1.53-1.63 (m, 1H), 1.68-179 (m, 3H), 3.50 (dt, *J* = 3.2, 8.1 Hz, 1H), 3.61-3.62 (m, 1H), 3.99 (dd, *J* = 1.5, 6.6 Hz, 1H), 4.37 (dd, *J* = 1.5, 14.0 Hz, 1H), 6.12 (dd, *J* = 6.6, 14.1 Hz, 1H). ^{13}C NMR (100 MHz, C_6D_6): δ = 24.42, 22.12, 26.96, 30.72, 68.93, 79.44, 89.02, 150.79. HRMS (pos. APCI): calcd for $C_8H_{14}O_2$ $[M+H]^+$: 143.1067, found: 143.1066. MS (CI, 70 eV): m/z (%) = 143.1 $[M+H]^+$ (100), 127.1 (4), 99.1 (14), 81.1 (23). IR (ATR): ν [cm^{-1}] = 603, 607, 616, 623, 627, 759, 807, 987, 1009, 1036, 1045, 1099, 1113, 1135, 1261, 1364, 1447, 2855, 2929, 3735.

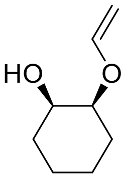

(*R,S*)-**2a**

(1*R*,2*S*)-2-(Vinyloxy)cyclohexan-1-ol. Synthesized from *cis*-2-methylenehexahydrobenzo[d][1,3]dioxole (**1a**, 316.2 mg, 2.255 mmol, 1.0 equiv) following the general catalytic desymmetrization procedure with the following modification: (*S,S*)-(ebthi)$ZrCl_2$ (24.0 mg, 56 µmol, 2.5 mol-%) was used as catalyst. The product was purified by column chromatography (*n*-pentane/CH_2Cl_2 3:1 containing 1% (v/v) NEt_3, R_f = 0.3) and obtained as a colorless liquid in 80% yield (257 mg, 1.81 mmol) and 92% ee. $[\alpha]_D^{23} = +19.3°$ (*c* 0.09, C_6H_6).

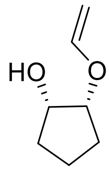

2b

(1*S*,2*R*)-2-(Vinyloxy)cyclopentan-1-ol. Synthesized from *cis*-2-methylenetetrahydro-4H-cyclopenta[d][1,3]dioxole (**1b**) following the general catalytic desymmetrization procedure. The product was purified by column chromatography (cyclohexane/EtOAc 9:1 R_f = 0.2) and obtained as a colorless liquid in 99% yield (25.4 mg, 0.198 mmol) and 90%ee. $[\alpha]_D^{23} = +21.9°$ (*c* 0.12, C_6H_6) ^{1}H NMR (300 MHz, C_6D_6): δ = 1.04-1.20 (m, 1H), 1.41-1.55 (m, 2H), 1.57-1.73 (m, 3H), 1.99 (d, *J* = 5.3 Hz, 1H), 3.69-3.74 (m, 1H), 3.84-3.90 (m, 1H), 3.97 (dd, *J* =1.7, 6.6 Hz, 1H), 4.23 (dd, *J* = 1.8, 14.3 Hz, 1H), 6.16 (dd, *J* = 6.4, 14.3 Hz, 1H). ^{13}C NMR (100 MHz, C_6D_6): δ = 19.80, 28.05, 31.31, 73.03, 80.96, 88.68, 150.75. HRMS (pos. APCI): calcd for $C_{10}H_{18}O_2$ $[M+H]^+$: 129.0910, found: 129.0909. MS (CI, 70 eV): m/z (%) = 129.1 (41) $[M+H]^+$, 127.1 (61), 113.0 (100), 85.1 (37), 84.1 (18), 67 (30). ν [cm^{-1}] = 555, 587, 594, 598, 607, 619, 625, 678, 1055, 1109, 1388, 1518, 1587, 2070, 3405.

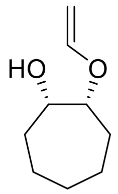

2c

(1*S*,2*R*)-2-(Vinyloxy)cycloheptan-1-ol. Synthesized from *cis*-2-methylenehexahydro-4H-cyclohepta[d][1,3]dioxole (**1c**) following the general catalytic desymmetrization procedure. The product was purified by column chromatography (cyclohexane/EtOAc 9:1 containing 1% (v/v) NEt_3, R_f = 0.5) and obtained as a colorless liquid in 96% yield (30.0 mg, 0.192 mmol) and 88% ee. $[\alpha]_D^{23} = +83.6°$ (*c* 0.13, C_6H_6). ^{1}H NMR (500 MHz, C_6D_6): δ = 1.07-1.18 (m, 2H), 1.26-1.56 (m, 5H), 1.61-1.69 (m, 1H), 1.72-1.78 (m, 1H), 1.83-1.90 (m, 2H), 3.58 (dt, *J* = 2.8, 8.9 Hz, 1H), 3.75-3.79 (m, 1H), 4.00 (dd, *J* = 1.5, 6.6 Hz, 1H), 4.33 (dd, *J* = 1.5, 14.2 Hz, 1H), 6.13 (dd, *J* = 6.6, 14.0 Hz, 1H). ^{13}C NMR (125 MHz, C_6D_6): δ = 21.63, 22.67, 27.08, 27.59, 31.68, 71.89, 82.85, 88.73, 150.95. HRMS (pos. APCI): calcd for $C_9H_{16}O_2$ $[M+NH_4]^+$: 174.1489 found: 174.1489. MS (CI): m/z (%) = 174.2 (9) $[M+NH_4]^+$, 157.1 (19), 155.1 (100), 141.1 (40), 130.1 (8), 112.2 (7), 95.1 (9), 75.1 (5). IR (ATR): ν [cm^{-1}] = 973, 1024, 1156, 1369, 1457, 1734, 2861, 3482.

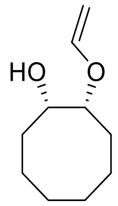

2d

(1*S*,2*R*)-2-(Vinyloxy)cyclooctan-1-ol. Synthesized from *cis*-2-methyleneoctahydrocyclo-octa[d][1,3]dioxole (**1d**) following the general catalytic desymmetrization procedure. The product was purified by column chromatography (cyclohexane/EtOAc 9:1 R_f = 0.2) and obtained as a colorless liquid in 88% yield (29.8 mg, 0.175 mmol) and 83% ee $[\alpha]_D^{23} = +61.5°$ (*c* 0.11, C_6H_6). ^{1}H NMR (500 MHz, C_6D_6): δ = 1.11-1.30 (m, 5H), 1.35-1.42 (m, 1H), 1.45-1.64 (m, 4H), 1.77-1.85 (m, 1H), 1.99-2.09 (m, 2H), 3.75 (dt, *J* = 2.4, 9.3 Hz, 1H), 3.80-3.83 (m, 1H), 4.01 (dd, *J* = 1.5, 6.6 Hz, 1H), 4.31 (dd, *J* = 1.5, 14.2 Hz, 1H), 6.15 (dd, *J* = 6.7, 14.2 Hz, 1H). ^{13}C NMR (125 MHz, C_6D_6): δ = 21.87, 25.33, 25.44, 26.77, 27.06, 29.84, 71.47, 81.68, 88.66,

150.75. HRMS (neg. APCI): calcd for $C_{10}H_{18}O_2$ [M - H]$^-$: 169.1234, found: 169.1237. MS (CI, 70 eV): m/z (%) = 188.2 (20) [M+NH$_4$]$^+$, 171.2 (42), 169.2 (45), 155.1 (100), 144.2 (28). 127.2 (57), 126.2 (26), 109.1 (99), 81.1 (8). IR (ATR): ν [cm^{-1}] = 812, 891, 1013, 1141, 1261, 1419, 1738, 2929, 3122.

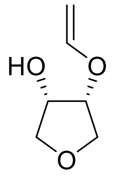

2e

(3*S*,4*R*)-4-(Vinyloxy)tetrahydrofuran-3-ol. Synthesized from *cis*-2-methylenetetrahydrofuro[3,4-d][1,3]dioxole (**1e**) following the general catalytic desymmetrization procedure. The product was purified by column chromatography (cyclohexane/EtOAc 9:1 containing 1% (v/v) NEt_3, R_f = 0.3) and obtained as a colorless liquid in 85% yield (22.2 mg, 0.171 mmol) and 90% ee. $[\alpha]_D^{23}$ = +29.7° (*c* 0.14, C_6H_6). ^{1}H NMR (400 MHz, C_6D_6): δ = 2.08 (s, 1H), 3.56-3.74 (m, 5H), 3.87-3.91 (m, 2H), 4.04 (dd, *J* = 2.3, 14.3 Hz, 1H), 6.03 (dd, *J* = 6.7, 14.2 Hz, 1H). ^{13}C NMR (100 MHz, C_6D_6): δ = 70.10, 71.04, 72.75, 77.39, 89.16, 150.21. HRMS (pos. APCI): calcd for $C_6H_{10}O_3$ [M+NH$_4$]$^+$: 148.0968 found: 148.0968. MS (CI): m/z (%) = 148.1 (68) [M+NH$_4$]$^+$, 131.1 (65), 129.0 (25), 115.0 (34), 87.1 (36), 86.0 (22), 72.1 (4), 70.1 (100), 69.1 (41), 68.1 (15), 60.2 (4). IR (ATR): ν [cm^{-1}] = 555, 559, 565, 571, 576, 588, 667, 1035, 1478, 1815, 3036, 3071, 3091.

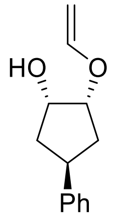

2f

(1*S*,2*R*,4*S*)-4-Phenyl-2-(vinyloxy)cyclopentan-1-ol. Synthesized from *cis,trans*-2-methylene-5-phenyltetrahydro-4H-cyclopenta[d][1,3]dioxole (**1f**) following the general catalytic desymmetrization procedure. The product was purified by column chromatography (cyclohexane/EtOAc 6:1 containing 1% (v/v) NEt_3, R_f = 0.3) and obtained as a colorless liquid in 67% yield (27.3 mg, 0.134 mmol) and 78% ee. $[\alpha]_D^{23}$ = +6.41° (*c* 0.14, C_6D_6). ^{1}H NMR (400 MHz, C_6H_6): δ = 1.54-1.67 (m, 2H), 2.10-2.17 (m, 3H), 3.50 (tt, *J* = 8.7, 8.8 Hz, 1H), 3.95-4.00 (m, 2H), 4.00-4.11 (m, 1H), 4.24 (dd, *J* = 1.9, 14.3 Hz, 1H), 6.17 (dd, *J* = 6.7, 14.2 Hz, 1H), 6.92-6.96 (m, 2H), 7.03-7.07 (m, 1H), 7.11-7.15 (m, 2H). ^{13}C NMR (100 MHz, C_6D_6): δ = 37.46, 40.47, 40.61, 73.15, 80.92, 88.99, 126.21, 127.18, 128.70, 146.14, 150.66. HRMS (pos. APCI): calcd for $C_{13}H_{16}O_2$ [M+NH$_4$]$^+$: 222.1486 found: 222.1486. MS (CI): m/z (%) = 205.1 (17), [M+H]$^+$, 203.1 (29), 189.1 (35), 160.1 (78), 143.1 (71), 142.1 (100), 128.1 (6), 115.1 (5), 104.1 (8), 91.1 (5), 78.1 (6). IR (ATR): ν [cm^{-1}] = 551, 554, 558, 562, 566, 571, 578, 580, 583, 587, 598, 601, 606, 614, 622, 1035, 1105, 1478, 1815, 1961, 3036, 3071, 3091.

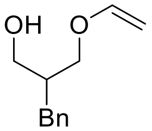

2g

2-Benzyl-3-(vinyloxy)propan-1-ol. Synthesized from 5-benzyl-2-methylene-1,3-dioxane (**1g**) following the general catalytic desymmetrization procedure but applying a concentration of *c* = 1.0 M. The product was purified by column chromatography (*n*-pentane/CH_2Cl_2 3:1 containing 1% (v/v) NEt_3, R_f = 0.3) and obtained as a colorless liquid in 74% yield (28.5 mg,

0.051 mmol) and 33% ee. $[\alpha]_D^{23} = -2.3°$ (*c* 0.007, C_6H_6). ^1H NMR (500 MHz, C_6D_6): δ = 1.36 (s, 1H), 1.92 (m, 1H), 2.56 (d, *J* = 7.5 Hz, 2H), 3.34-3.41 (m, 2H), 3.51 (d, *J* = 5.5 Hz, 2H), 3.92 (dd, *J* = 1.8, 6.9 Hz, 1H), 4.14 (dd, *J* = 1.8, 14.3 Hz, 1H), 6.37 (dd, *J* = 6.7, 14.3 Hz, 1H), 7.03-7.07 (m, 3H), 7.11-7.15 (m, 2H). ^{13}C NMR (125 MHz, C_6D_6): δ = 34.39, 42.90, 62.61, 68.07, 86.59, 126.29, 128.29, 128.62, 129.44, 140.29, 152.07. HRMS (pos. APCI): calcd for $C_{12}H_{16}O_2$ $[M+NH_4]^+$: 210.1489, found: 210.1489. MS (CI, 70 eV): m/z (%) = 210.1 $[M+NH_4]^+$ (100), 193.2 (81), 175.1 (35), 164.1 (79), 147.1 (49), 131.1 (83), 117.1 (34), 108.1 (20), 104.1 (13), 91.0 (56), 61.1 (23). IR (ATR): ν [cm^{-1}] = 606, 615, 700, 743, 812, 910, 945, 963, 990, 1030, 1101, 1200, 1261, 1320, 1330, 1377, 1454, 1467, 1495, 1615, 2280, 2925, 3423.

The reduced enantioselectivity in the β-O-elimination reaction from **1g** to **2g** is presumably connected to a) the lack of further substitution at the former primary diol carbons and/or b) the six-membered structure of the ketene acetal. The result is a significant "flatter" structure of **1g** in comparison to **1a** and thus the facial discrimination by the catalyst is reduced. Figure S1 shows side-view 3D structures of **1a** and **1g**, illustrating this rationale (created with Avogadro).[15]

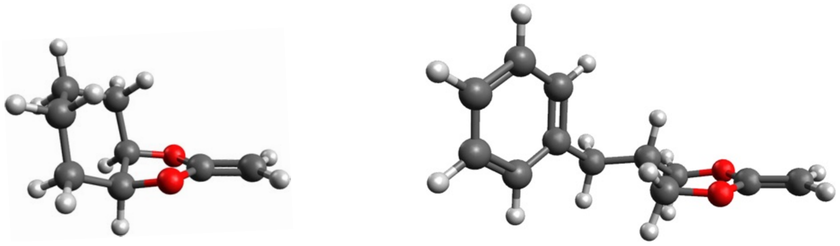

1a **1g**

Figure S1. 3D representations of substrates **1a** and **1g** (side view).

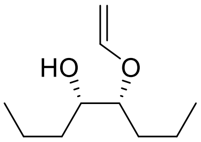

2h

(4*S*,5*R*)-5-(Vinyloxy)octan-4-ol. Synthesized from *cis*-2-methylene-4,5-dipropyl-1,3-dioxolane (**1h**) following the general catalytic desymmetrization procedure with the following exception: the reaction was stirred for 9h at 18°C. The product was purified by column chromatography (cyclohexane/EtOAc 9:1 containing 1% (v/v) NEt_3 R_f = 0.5) and obtained as a colorless liquid in 71% yield (24.5 mg, 0.142 mmol) and 84% ee. $[\alpha]_D^{23} = +34.7°$ (*c* 0.08, C_6H_6). ^1H NMR (400 MHz, C_6D_6): δ = 0.84 and 0.87 (2 x t, *J* = 7.3 Hz and *J* = 7.3 Hz, 6H), 1.18-1.40 (m, 7H), 1.42-1.54 (m, 2H), 1.56-1.65 (m, 1H), 3.45 (dt, *J* = 3.8, 7.6 Hz, 1H), 3.51-3.56 (m, 1H), 3.96 (dd, *J* = 1.4, 6.4 Hz, 1H), 4.41 (dd, *J* = 1.4, 13.9 Hz, 1H), 6.13 (dd, *J* = 6.4, 14.0 Hz, 1H). ^{13}C NMR (100 MHz, C_6D_6): δ = 14.21, 19.13, 19.43, 31.91, 34.71, 72.58, 83.96, 88.29, 152.47. HRMS (pos. APCI): calcd for $C_{10}H_{20}O_2$ $[M+NH_4]^+$: 190.1802 found: 190.1800. MS (CI): m/z (%) = 190.2 (9), $[M+NH_4]^+$, 173.1 (100), 146.2 (10), 129.2 (15), 128.2 (18), 111.2 (7). IR (ATR): ν [cm^{-1}] = 552, 557, 564, 576, 582, 586, 594, 597, 611, 616, 620, 623, 631, 634, 642, 667, 819, 958, 1027, 1065, 1117, 1189, 1244, 1376, 1466, 1632, 1719, 2873, 2934, 2959, 3421.

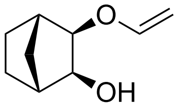

2i

(1*R*,2*S*,3*R*,4*S*)-3-(Vinyloxy)bicyclo[2.2.1]heptan-2-ol. Synthesized from *exo*-methylenehexahydro-4,7-methanobenzo[d][1,3]dioxole. (**1i**) following the general catalytic desymmetrization procedure. The product was purified by column chromatography (*n*-pentane/Et_2O 5:1 containing 1% (v/v) NEt_3, R_f = 0.4) and obtained as a colorless liquid in 78%

yield (24.0 mg, 0.156 mmol) and 94% ee. $[\alpha]_D^{23} = -5.9°$ (*c* 0.11, C_6D_6). ^1H NMR (400 MHz, C_6H_6): δ = 0.61-0.76 (m, 2H), 0.86-0.90 (dpent, *J* = 1.5, 10.1 Hz, 1H), 1.06-1.19 (m, 2H), 1.86-1.90 (dp, *J* = 1.5, 10.1 Hz, 1H), 2.08-2.09 (m, 1H), 2.18-2.19 (m, 1H), 2.60-2.61 (d, *J* = 4.6 Hz, 1H), 3.40 (dd, *J* = 1.7, 5.9 Hz, 1H), 3.62-3.64 (m, 1H), 3.95 (dd, *J* = 1.9, 6.7 Hz, 1H), 4.21 (dd, *J* = 2.0, 14.3 Hz, 1H), 6.15 (ddd, *J* = 0.5, 6.7, 10.7 Hz, 1H). ^{13}C NMR (100 MHz, C_6D_6): δ = 24.23, 24.68, 32.46, 40.30, 43.66, 75.66, 81.84, 88.89, 151.08. HRMS (pos. APCI): calcd for $C_9H_{14}O_2$ $[M+H]^+$: 155.1067 found: 155.1067 MS (CI): m/z (%) = 155.1 (50) $[M+H]^+$, 153.1 (69), 139.1 (100), 137.1 (22), 111.1 (27), 93.1 (15), 81.1 (6), 67.1 (5). IR (ATR): ν [cm^{-1}] = 551, 554, 559, 567, 570, 574, 580, 587, 659, 779, 806, 936, 998, 1016, 1038, 1095, 1136, 1213, 1263, 1331, 1395, 1426, 1457, 2909, 2973, 3388.

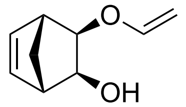

2j

(19:1 mixture with **2i**)

(1*S*,2*S*,3*R*,4*R*)-3-(Vinyloxy)bicyclo[2.2.1]hept-5-en-2-ol. Synthesized from *exo*-2-methylene-3a,4,7,7a-tetrahydro-4,7-methanobenzo[d]-[1,3]dioxole (**1j**) following the general catalytic desymmetrization procedure. The product was purified by column chromatography (*n*-pentane/Et_2O 5:1 containing 1% (v/v) NEt_3, R_f = 0.4) and obtained as a colorless liquid in 70% yield (21.4 mg, 0.141 mmol) and 86% ee as a 19:1 mixture with **2i**. $[\alpha]_D^{23} = -9.3°$ (*c* = 0.12, C_6H_6). ^1H NMR (500 MHz, C_6D_6): δ = 1.53-1.56 (m, 1H), 1.98-2.01 (m, 1H), 2.59-2.71 (m, 3H), 3.47-3.48 (m, 1H), 3.70-3.72 (m, 1H), 3.95 (dd, *J* = 2.0, 6.6 Hz, 1H), 4.23 (dd, *J* = 2.0, 14.3 Hz. 1H), 5.62 (dd, *J* = 3.1, 5.8 Hz, 1H), 5.75 (dd, *J* = 3.1, 6.0 Hz, 1H), 6.11 (dd, *J* = 6.7, 14. 2Hz, 1H). ^{13}C NMR (125 MHz, C_6D_6): δ = 43.18, 45.48, 48.74, 69.56, 76.25, 89.18, 135.26, 137.71, 150.86. HRMS (pos. APCI): calcd for $C_9H_{12}O_2$ $[M+NH_4]^+$: 188.1281 found: 188.1279. MS (CI): m/z (%) = 170.1 (5), 153.1 (7) $[M+H]^+$: 151.1 (41), 135.1 (4), 126.1 (14), 109.1 (100), 86.0 (38), 71.0 (26), 65.1 (4). IR (ATR): ν [cm^{-1}] = 555, 567, 575, 579, 585 587, 587, 600, 605, 611, 619, 698, 746, 968, 1030, 1054, 1455, 1496, 1603, 2873, 2963, 3026, 3368.

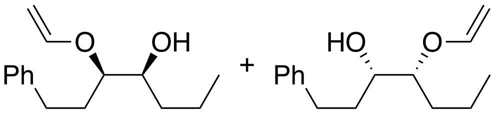

4 **5**

(3*R*,4*S*)-1-Phenyl-3-(vinyloxy)heptan-4-ol and **(3*S*,4*R*)-1-phenyl-4-(vinyloxy)heptan-3-ol**. Synthesized from *cis*-2-methylenehexahydro-4,7-methanobenzo[d][1,3]dioxole (*rac*-**3**, 1.3 mmol, 1.0 equiv) following the general catalytic desymmetrization procedure. The product was purified by column chromatography (*n*-pentane/Et_2O 5:1 containing 1% (v/v) NEt_3, R_f = 0.4) and obtained as a mixture of regioisomers a colorless liquid in 66% yield (145.6 mg, 0.621 mmol) and 83%ee and 78%ee. $[\alpha]_D^{23} = -16.7°$ (*c* 0.11, C_6H_6). ^1H NMR (400 MHz, C_6D_6): δ = 0.79 and 0.82 (2 x t, *J* = 7.2 and *J* = 7.3 Hz, 3H), 1.12-1.47 (m, 6H), 1.51-1.72 (m, 2.47H), 1.91-1.98 (m, 0.53H), 2.50-2.58 (m, 1.08H), 2.73-2.84 (m, 1.28H), 3.38-3.42 (m, 1.03H), 3.49-3.53 (m, 1H), 3.93 and 3.98 (2 x dd, *J* = 1.4, 6.4 Hz and *J* = 1.5, 6.6 Hz, 1H), 4.38 and 4.42 (2 x dd, *J* = 1.4, 13.9 Hz, and *J* = 1.4, 13.9 Hz, 1H), 6.11 and 6.14 (2 x dd, *J* = 6.4, 14.0 Hz, and *J* = 6.5, 14.0 Hz, 1H), 7.04-7.13 (m, 3H), 7.14-7.17 (m, 2H). ^{13}C NMR (100 MHz, C_6D_6): δ = 14.18, 19.07, 19.41, 31.38, 31.91, 32.50, 34.31, 34.70, 72.04, 72.37, 83.02, 83.89, 88.40, 88.66, 126.14, 126.18, 128.29, 128.68, 128.81, 128.82, 142.17, 142.41, 152.29, 152.44. HRMS (pos. APCI): calcd for $C_{15}H_{22}O_2$ $[M+NH_4]^+$: 252.1958 found: 252.1959. MS (CI): m/z (%) = 252.2 (9) $[M+NH_4]^+$, 235.2 (24), 208.1 (55), 191.1 (87), 173.1 (100), 161.1 (7), 117.1 (5), 104.1 (9), 91.1 (14) IR (ATR): ν [cm^{-1}] = 555, 559, 563, 570, 575, 579, 587, 593, 699, 748, 821, 946, 967, 1030, 1065, 1139, 1187, 1324, 1380, 1454, 1496, 1631, 2872, 2957, 3027, 3430.

Product Derivatization

Determination of the Absolute Configuration of 2a

Enantiomerically enriched compound **2a** prepared with (*R*,*R*)-(ebthi)$ZrCl_2$ as catalyst was converted into crystalline bromobenzoate derivative **S27** by Steglich esterification and vinyl ether hydrolysis as follows. Compound **S27** was then crystallized and the absolute configuration was determined as (*S*,*R*) by X-ray analysis of the obtained single crystals.

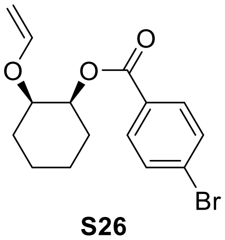

S26

(1*S*,2*R*)-2-(Vinyloxy)cyclohexyl 4-bromobenzoate. Synthesized according to a modified literature procedure for Steglich esterification. [16] To a stirred solution of (1*S*,2*R*)-2-(vinyloxy)cyclohexan-1-ol (**2a**, 14.3 mg, 0.1 mmol, 1.0 equiv) in CH_2Cl_2 (0.5 ml) DMAP (1.1 mg, 9 µmol, 9 mol%) and 4-bromobenzoic acid (40.2 mg, 0.2 mmol, 2.0 equiv) was added. The reaction mixture was cooled to 0°C (ice bath) and DCC (41.2 mg, 0.2 mmol, 2.0 equiv) was added. The reaction mixture was stirred at 0°C for further 30 min. Then, the ice bath was removed and the resulting white suspension was stirred at room temperature overnight. Afterwards, it was quenched by addition of H_2O (5 ml) and CH_2Cl_2 (2 ml). The layers were separated and the organic layer was extracted with CH_2Cl_2 (3 x 2 ml). The combined organic layers were dried over Na_2SO_4, filtered and the solvent was removed under reduced pressure. The product was purified by column chromatography (*n*-pentane/Et_2O 5:1 containing 1% (v/v) NEt_3, R_f = 0.6) and obtained as a colorless liquid in 91% yield (29.4 mg, 0.091 mmol) and 90%ee $[\alpha]_D^{23} = +2.14°$ (*c* 0.93, $CHCl_3$). ^1H NMR (400 MHz, C_6D_6): δ = 1.00-1.09 (m, 2H), 1.21-1.27 (m, 1H), 1.37-1.54 (m, 3H), 1.73-1.80 (m, 1H), 1.90-2.00 (m, 1H), 3.76-3.79 (m, 1H), 3.96 (dd, *J* = Hz, 1H), 4.40 (dd, *J* = Hz, 1H), 5.11-5.15 (m, 1H), 6.15 (dd, *J* = Hz, 1H), 7.13-7.14 (m, 2H), 7.84-7.88 (m, 2H). ^{13}C NMR (100 MHz, C_6D_6): δ = 20.92, 22.66, 27.27, 28.45, 73.27, 76.52, 88.53, 129.91, 131.52, 131.85, 151.40, 164.98. MS (EI): m/z (%) = 325.8 (1) $[M]^+$, 323.9 (1) $[M]^+$, 285.2 (2), 284.1 (9), 283.2 (3), 281.9 (10), 154.0 (2), 152.2 (2), 131.5 (8), 130.7 (100), 128.8 (12), 127.7 (24), 126.7 (9), 125.7 (4), 124.2 (4), 120.3 (1), 118.3 (1), 108.3 (1), 106.8 (1). HRMS (pos. APCI): calcd for $C_{15}H_{17}BrO_3$ $[M+H]^+$: 327.0420 found: 327.0400. IR (ATR): ν $[cm^{-1}]$ = 2940, 2862, 1713, 1591, 1485, 1450, 1398, 1344, 1311, 1270, 1184, 1173, 1133, 1112, 1103, 1069, 1040, 1023, 1012, 992, 947, 892, 847, 821, 756, 683, 627, 495, 467.

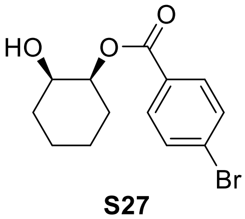

S27

(1*S*,2*R*)-2-Hydroxycyclohexyl 4-bromobenzoate. (1*S*,2*R*)-2-(vinyloxy)cyclohexyl 4-bromobenzoate (**S26**, 32.4 mg, 0.1 mmol, 1.0 equiv.) was dissolved in THF (1.0 ml). Hydrochloric acid (1.0 M, 0.5 ml) was added and the resulting clear solution was stirred at room temperature overnight. Afterwards, it was quenched by the addition of sat. aq. $NaHCO_3$ solution (2 ml) and transferred to a separatory funnel. The aqueous layer was extracted with ethyl acetate (3 x 5 ml), the combined organic layers were dried over Na_2SO_4, filtered and the solvent was removed under reduced pressure. The product was purified by column chromatography (*n*-pentane/EtOAc 5:1 R_f = 0.4) and obtained as a white solid in 85% yield (25.3 mg, 0.085 mmol) and 92% ee. $[\alpha]_D^{23} = +5.41°$ (*c* 0.86, $CHCl_3$). The product was dissolved in 200 µL CH_2Cl_2 (200 µL) and recrystallized from pentane by diffusion crystallization. Single

crystals were received that were suitable for X-ray crystallography, which confirmed the absolute configuration as (*S*,*R*). See below for the X-ray analysis report. The reported analytical data matched the literature values.[17] Melting point: 82°C. ^{1}H NMR (400 MHz, $CDCl_3$): δ = 1.38-1.51 (m, 2H), 1.61-1.78 (m, 4H), 1.80-1.87 (m, 1H), 1.92 (s, 1H), 1.96-2.05 (m, 1H), 3.96-3.98 (m, 1H), 5.19-5.23 (m, 1H), 7.57-7.61 (m, 2H), 7.90-7.92 (m, 2H). ^{13}C NMR (100 MHz, $CDCl_3$): δ = 21.64, 21.95, 27.51, 30.57, 69.73, 75.09, 128.34, 129.47, 131.29, 131.92, 165.69. MS (EI): m/z (%) = 297.3 (1) $[M]^+$, 299.5 (1) $[M]^+$, 186.8 (57), 184.9 (58), 159.7 (6), 157.7 (14), 100.6 (21), 98.5 (100), 83.5 (9), 81.5 (16), 41.7 (4), 40.8 (4). HRMS (pos. APCI): calcd for $C_{15}H_{22}O_2$ $[M+NH_4]^+$: 299.0283 found: 299.0279. IR (ATR): ν [cm^{-1}] = 3443, 2937, 2861, 1716, 1590, 1484, 1449, 1398, 1344, 1272, 1173, 1103, 1069, 1012, 983, 941, 917, 889, 847, 815, 756, 683, 627, 496, 467.

Oxidation and Deprotection of Regioisomers 4 and 5

The enantioenriched regioisomers **4** and **5** were successfully oxidized and then hydrolyzed to give the corresponding α-hydroxyketones. Attempts to separate the regioisomers on each stage by achiral preparative HPLC were not successful. However, chiral HPLC led to a separation of all four product regioisomers and enantiomers on the analytical scale.

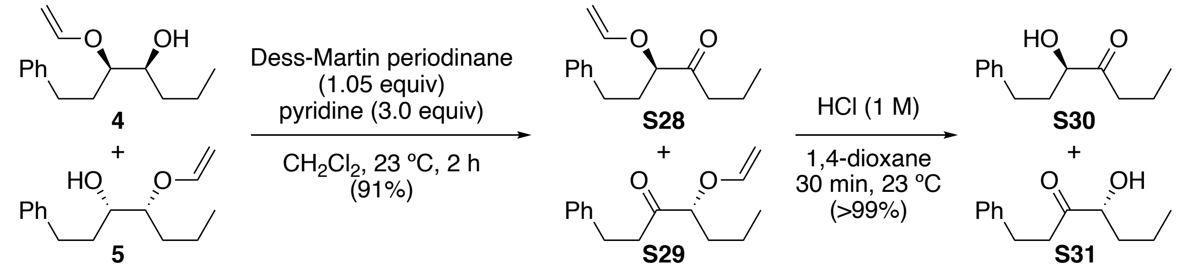

(82% *ee* and 79% *ee*) (79% *ee* and 75% *ee*) (78% *ee* and 75% *ee*)

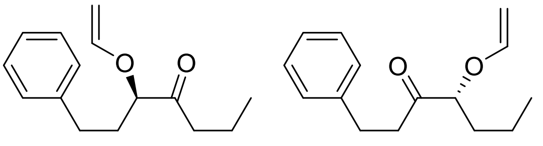

S28 **S29**

(*R*)-1-Phenyl-4-(vinyloxy)heptan-3-one and (*R*)-1-phenyl-3-(vinyloxy)heptan-4-one. Synthesized according to a modified literature procedure for Dess-Martin oxidation.[18] To a solution of (3*R*,4*S*)-1-phenyl-3-(vinyloxy)heptan-4-ol and (3*S*,4*R*)-1-phenyl-4-(vinyloxy)-heptan-3-ol (**4** and **5**, 70.3 mg, 0.3 mmol, 1.0 equiv) and pyridine (130 µL, 71.2 mg, 0.9 mmol, 3.0 equiv) in CH_2Cl_2 (2.5 ml) Dess-Martin periodinane (133.6 mg, 0.315 mmol, 1.05 equiv) was added in portions. The reaction was monitored by TLC control once in 15 min. After 2h the substrate was fully consumed. The solvent was removed under reduced pressure and the residue was directly purified by column chromatography (cyclohexane/EtOAc 9 : 1 containing 1% (v/v) NEt_3, R_f = 0.6) The product was obtained as mixture of regioisomers as a colorless liquid in 91% yield (63.6 mg, 0.274 mmol) and 79%ee and 75%ee. $[\alpha]_D^{23}$ = +13.8° (*c* 0.06, C_6H_6). ^{1}H NMR (500 MHz, C_6D_6): δ = 0.70 and 0.78 (2 x t, *J* = 7.3 and *J* = 7.5 Hz, 3H), 1.13-1.31 (m, 1.32H), 1.36-1.43 (m, 0.74H), 1.45-1.52 (m, 0.58H), 1.53-1.59 (m, 0.92H) 1.80-1.93 (m, 1H), 2.27 (t, *J* = 7.2 Hz, 1H), 2.51-2.57 (m, 0.47H), 2.60-2.69 (m, 1.39H), 2.80-2.92 (m, 1H), 3.86-3.91 (m, 1H), 3.94-3.96 (m, 1H), 4.13-4.18 (m, 1H), 6.08-6.15 (m, 1H), 7.00-7.07 (m, 3H), 7.10-7.15 (m, 2H). ^{13}C NMR (125 MHz, C_6D_6): δ = 13.65, 13.93, 17.04, 18.48, 29.71, 31.56, 35.86, 35.89, 39.20, 39.39, 75.47, 76.35, 126.32, 126.45, 128.00, 128.61, 128.71, 128.96, 141.13, 141.70, 210.88, 211.41. HRMS (pos. APCI): calcd for $C_{15}H_{20}O_2$ $[M+H]^+$: 233.1536 found: 233.1538. MS (CI): m/z (%) = 233.1 (100) $[M+H]^+$, 215.1 (88), 208.1 (37), 191.1 (27), 189.1 (44), 187.1 (9), 145.1 (3), 128.1 (9), 117.1 (7), 91.1 (10). IR (ATR): ν [cm^{-1}]

= 551, 555, 559, 565, 570, 574, 588, 596, 699, 748, 1031, 1074, 1401, 1454, 1469, 1604, 1710, 1776, 1977, 2158, 2964, 3027, 3486.

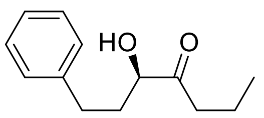

S30

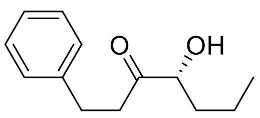

S31

(*R*)-4-Hydroxy-1-phenylheptan-3-one and **(*R*)-3-hydroxy-1-phenylheptan-4-one**. (*R*)-1-phenyl-4-(vinyloxy)heptan-3-one and (*R*)-1-phenyl-3-(vinyloxy)heptan-4-one (**S28** and **S29**, 11.6 mg, 0.056 mmol, 1.0 equiv) was dissolved in 1,4-dioxane (2 ml). Hydrochloric acid (1.0M, 1.0 ml) was added and the resulting clear solution was stirred for 30 min at room temperature. Afterwards, it was quenched by the addition of sat. aq. $NaHCO_3$ solution (2 ml) and transferred to a separatory funnel. The aqueous layer was extracted with ethyl acetate (3 x 5 ml), the combined organic layers were dried over Na_2SO_4, filtered and the solvent was removed under reduced pressure. The products were purified by column chromatography (cyclohexane/EtOAc 3:1 R_f = 0.6) and obtained as a mixture of regioisomers as a colorless oil in >99% yield (11.4 mg, 0.056 mmol) and 78% and 75% ee. $[\alpha]_D^{23}$ = +41.6° (*c* 0.12, C_6H_6). ^{1}H NMR (500 MHz, C_6D_6): δ = 0.66 and 0.77 (2 x t, *J* = 7.3 Hz and *J* = 7.2 Hz, 3H), 1.09-1.17 (m, 0.61), 1.24-1.52 (m, 3.77H), 1.62-1.68 (m, 0.54H), 1.75-1.87 (m, 1.03H), 2.09-2.16 (m, 0.53H), 2.26-2.33 (m, 0.5H), 2.65-2.81 (m, 2H), 3.37-3.38 (m, 0.44H), 3.58-3.59 (m, 0.46H), 3.73-3.80 (m, 1H), 6.95-6.98 (m, 1H), 7.03-7.18 (m, 4H). ^{13}C NMR (125 MHz, C_6D_6): δ = 13.65, 13.94, 17.04, 18.48, 29.72, 31.57, 35.86, 35.89, 39.21, 39.39, 75.47, 76.36, 126.32, 126.45, 128.29, 128.61, 128.71, 128.96, 141.14, 141.70, 210.88, 211.41. HRMS (pos. APCI): calcd for $C_{13}H_{18}O_2$ $[M+H]^+$: 207.1380 found: 207.1380. MS (CI): m/z (%) = 224.3 (21), 207.1 (100) $[M+H]^+$, 189.1 (47), 134.1 (4), 91.0 (6). IR (ATR): ν [cm^{-1}] = 562, 572, 700, 749, 1031, 1065, 1141, 1267, 1362, 1403, 1454, 1497, 1064, 1710, 2874, 2933, 2961, 3028, 3063, 3473.

Synthesis of Enantioenriched *trans*-Diol and *trans*-Aminoalcohol Derivatives

Starting from (*S*,*R*)-**2a** and its enantiomer (*R*,*S*)-**2a** [prepared with (*S*,*S*)-**cat-1**], Mitsunobu reactions allowed the selective synthesis of each enantiomer of the orthogonally protected *trans*-diol **6** and *trans*-aminoalcohol **7**. Overall, our approach thus allowed the regio-, diastereo- and enantioselective preparation of the cyclic *cis*- and *trans*-cyclohexanediols as well as the corresponding *trans*-aminoalcohols.

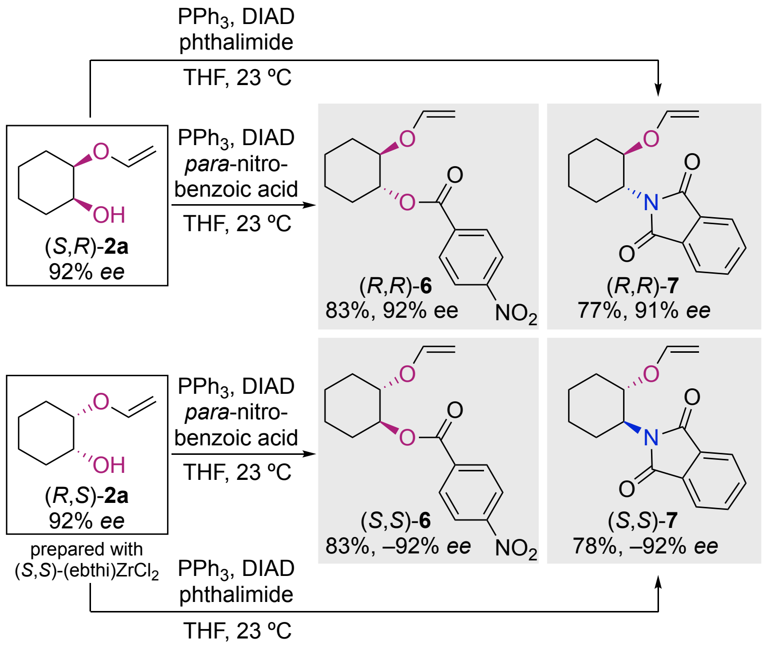

Scheme S1. Accessing all enantioenriched, orthogonally protected cyclohexane diol stereoisomers as well as the orthogonally protected *trans*-cyclohexane aminoalcohol enantiomers.

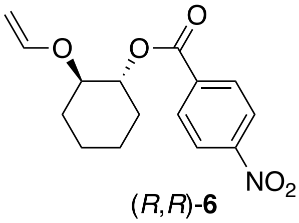

(*R*,*R*)-**6**

(1*R*,2*R*)-2-(Vinyloxy)cyclohexyl 4-nitrobenzoate. Synthesized according to a modified literature procedure for Mitsunobu inversion.[19] A flame dried 10 ml Schlenk tube equipped with a magnetic stirring bar was backfilled with argon and charged with 4-nitrobenzoic acid (323.5 mg, 1.6 mmol, 4.0 equiv), triphenylphosphine (419.7 mg, 1.6 mmol, 4.0 equiv), (1*S*,2*R*)-2-(vinyloxy)cyclohexan-1-ol (**2a**, 56.9 mg, 0.4 mmol, 1.0 equiv) and THF (3 ml). The reaction mixture was cooled to 0°C (ice bath) and diisopropyl azodicarboxylate (320 µL, 1.6 mmol, 4.0 eqiuv) was added dropwise. The resulting yellow solution was stirred at 0°C for further 10 min. Afterwards, the ice bath was removed, the reaction mixture was allowed to warm to room temperature and stirred overnight. Then, the solvent was removed under reduced pressure and the residue was directly purified by column chromatography (*n*-pentane/EtOAc = 4:1 containing 1% (v/v) NEt_3, R_f = 0.4) and obtained as a colorless liquid in 83% yield (96.6 mg, 0.332 mmol) and 92%ee $[\alpha]_D^{23}$ = –51.5° (*c* 0.74, $CHCl_3$). ^{1}H NMR (400 MHz, C_6D_6): δ = 0.85-1.03 (m, 2H), 1.14-1.39 (m, 4H), 1.76-1.83 (m, 1H), 1.92-1.99 (m, 1H), 3.58-3.64 (m, 1H), 3.94 (dd, *J* = 1.7, 6.6 Hz, 1H), 4.42 (dd, *J* = 1.7, 4.1 Hz, 1H), 5.13-5.19 (m, 1H), 6.20 (dd, *J* = 6.6, 14.1 Hz, 1H), 7.65-7.70 (m, 2H), 7.77-7.80 (m, 2H). ^{13}C NMR (100 MHz, C_6D_6): δ = 23.02, 23.08, 29.68, 29.90, 75.73, 78.97, 88.56, 123.38, 130.50, 135.66, 150.55, 151.30, 163.90. MS (EI): m/z (%) = 290.7 (1) [M]$^+$, 230.5 (2), 229.5 (16), 228.6 (100), 227.7 (10), 226.8 (3), 162.6 (4), 150.9 (4), 133.4 (5), 127.3 (3), 39.7 (2), 41.6 (13). HRMS (pos. APCI): calcd for $C_{15}H_{17}NO_5$

[M+H]$^+$: 292.1179 found: 292.2231. IR (ATR): ν [cm^{-1}] = 465, 508, 719, 784, 831, 873, 915, 949, 1014, 1048, 1082, 1103, 1116, 1136, 1159, 1187, 1218, 1269, 1321, 1348, 1378, 1410, 1454, 1526, 1608, 1635, 1722, 2865, 2941.

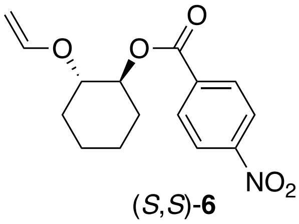

(*S*,*S*)-**6**

(1*S*,2*S*)-2-(Vinyloxy)cyclohexyl 4-nitrobenzoate. Synthesized in analogy to (1*R*,2*R*)-2-(vinyloxy)cyclohexyl 4-nitrobenzoate using (1*R*,2*S*)-2-(vinyloxy)cyclohexan-1-ol (***ent*-2a**, 56.9 mg, 0.4 mmol, 1.0 equiv) as substrate. The product was obtained as a colorless liquid in 83% yield (96.3 mg, 0.322 mmol) and 92%ee $[\alpha]_D^{23}$ = +57.9° (*c* 0.81, $CHCl_3$).

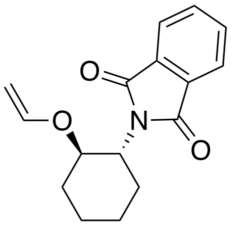

(*R*,*R*)-**7**

2-((1*R*,2*R*)-2-(Vinyloxy)cyclohexyl)isoindoline-1,3-dione. Synthesized according to a modified literature procedure for Mitsunobu inversion.[20] A flame dried 10 ml Schlenk tube equipped with a magnetic stirring bar was backfilled with argon and charged with phtalimide (117.7 mg, 0.8 mmol, 4.0 equiv), triphenylphosphine (209.8 mg, 0.8 mmol, 4.0 equiv) (1*S*,2*R*)-2-(vinyloxy)cyclohexan-1-ol (**2a**, 28.4 mg, 0.2 mmol, 1.0 equiv) and THF (1.5 ml) . The reaction mixture was cooled to 0°C (ice bath) and diisopropyl azodicyrboxylate (160 µL, 0.8 mmol, 4.0 equiv.) was added dropwise. The resulting yellow solution was stirred at 0°C for further 10 min. Afterwards, the ice bath was removed the reaction mixture was allowed to warm to room temperature and stirred overnight. Then, the solvent was removed under reduced pressure and the residue was directly purified by column chromatography (*n*-pentane/EtOAc = 4:1 containing 1% (v/v) NEt_3, R_f = 0.4) and obtained as a colorless liquid in 77% yield (41.8 mg, 0.154 mmol) and 91%ee $[\alpha]_D^{23}$ = +15.5° (*c* 0.85, $CHCl_3$). ^{1}H NMR (400 MHz, C_6D_6): δ = 0.82-1.06 (m, 2H), 1.12-1.22 (m, 1H), 1.29-1.39 (m, 2H), 1.55-1.61 (m, 1H), 1.94-2.01 (m, 1H), 2.17-2.27 (m, 1H), 3.74-3.74 (dd, *J* = 1.5, 6.5 Hz, 1H), 4.34-4.41 (m, 2H), 4.60-4.66 (m, 1H), 6.13-6.18 (dd, *J* = 6.5, 14.0 Hz, 1H), 6.81-6.86 (m, 2H), 7.43-7.48 (m, 2H). ^{13}C NMR (100 MHz, C_6D_6): δ = 24.08, 25.16, 29.30, 32.10, 54.99, 77.36, 88.70, 122.97, 132.39, 133.44, 151.12, 168.38. MS (EI): m/z (%) = 271.0 (1) [M]$^+$, 2320.5 (2), 229.5 (16), 228.6 (100), 227.7 (11), 226.8 (4), 225.1 (1), 224.3 (1), 127.5 (2), 105.9 (1). HRMS (pos. APCI): calcd for $C_{15}H_{22}O_2$ [M+NH$_4$]$^+$: 272.1287 found: 252.1319. IR (ATR): ν [cm^{-1}] = 531, 640, 700, 718, 794, 828, 842, 870, 905, 951, 1003, 1022, 1070, 1086, 1150, 1187, 1218, 1252, 1331, 1358, 1379, 1392, 1454, 1467, 1615, 1634, 1704, 1769, 2861, 2937.

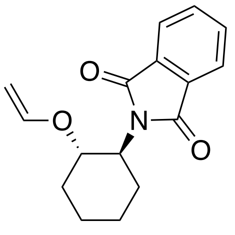

(*S*,*S*)-**7**

2-((1*S*,2*S*)-2-(Vinyloxy)cyclohexyl)isoindoline-1,3-dione. Synthesized in analogy to 2-((1*R*,2*R*)-2-(vinyloxy)cyclohexyl)isoindoline-1,3-dione using (1*R*,2*S*)-2-(vinyloxy)cyclohexan-1-ol (**2a**, 28.4 mg, 0.2 mmol, 1.0 equiv) as substrate. The product was obtained as a colorless liquid in 78% yield (42.2 mg, 0.156 mmol) and 92%ee $[\alpha]_D^{23} = -23.8°$ (*c* 0.75, $CHCl_3$).

One-Pot Hydroboration/Suzuki Coupling

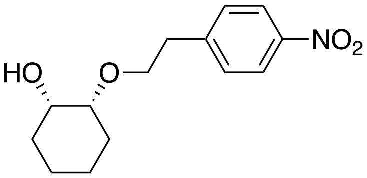

(*S*,*R*)-**8**

(1*S*,2*R*)-2-(4-Nitrophenethoxy)cyclohexan-1-ol. This compound was synthesized according to a modified literature procedure.[21] To a solution of (1*S*,2*R*)-2-(vinyloxy)cyclohexan-1-ol (**2a**, 28.4 mg, 0.2 mmol, 1.0 equiv) in THF (0.2 ml) was added 9-BBN in THF (0.5M, 2.1 ml, 0.42 mmol, 2.1 equiv) dropwise at 0°C. The resulting clear solution was warmed to room temperature and stirred for further 3 h. Then, Pd(dppf)Cl_2 (4.4 mg, 6 µmol, 3 mol%), water (400 µl) and solid NaOH (32.0 mg, 0.8 mmol, 4.0 equiv) were added subsequently and the solution turned black. Afterwards, 1-iodo-4-nitrobenzene (99.6 mg, 0.4 mmol, 2.0 equiv) was added and the reaction mixture was stirred for 14 h at 65°C. Then, water (5 ml) and Et_2O (5 ml) were added, the reaction mixture was transferred to a separatory funnel, the layers were separated and the aqueous layer was extracted with Et_2O (3 × 5ml). The combined organic layers were dried over Na_2SO_4, filtered and the solvent was removed under reduced pressure. The product was purified by column chromatography (*n*-pentane/EtOAc 1:1 R_f = 0.2) and obtained as a yellow oil in 79% yield (42.1 mg, 0.159 mmol) and 92% ee. $[\alpha]_D^{23} = -12.57°$ (*c* 0.07, $CHCl_3$). ^{1}H NMR (400 MHz, $CDCl_3$): δ = 1.18-1.31 (m, 2H), 1.44-1.60 (m, 4H), 1.64-1.78 (m, 2H), 1.98 (s, 1H), 2.99 (t, *J* = 6.4 Hz, 2H), 3.36-3.40 (m, 1H), 3.65-3.82 (m, 3H), 7.39-7.41 (m, 2H), 8.15-8.17 (m, 2H). ^{13}C NMR (100 MHz, $CDCl_3$): δ = 21.53, 21.88, 26.72, 30.54, 36.69, 68.27, 69.05, 78.99, 123.73, 129.87, 146.82, 147.41. HRMS (ESI): calcd for $C_{14}H_{19}NO_4$ $[M + H]^+$: 266.1387 found: 266.1392. MS (EI): m/z (%) = 266.5 (1) $[M]^+$, 248.5 (16), 247.5 (100), 190.5 (16), 150.5 (14), 133.5 (10), 132.5 (35), 121.0 (26), 106.5 (31), 103.5 (13), 90.6 (14), 414.7 (32). IR (ATR): ν [cm^{-1}] = 699, 749, 772, 856, 912, 982, 1094, 1344, 1447, 1517, 1600, 2860, 2933, 3447.

Additional Experiments with Unsymmetric Substrates

In addition to the regiodivergent opening of pseudo-meso substrates, we tested unsymmetric ketene acetals for which a substrate-induced regioselectivity was expected (Scheme S2). In the case of **S33**, a good conversion and 8.3:1 regioselectivity was obtained, but the enantioselectivity was insufficient. Moreover, the major regioisomer (**S34**) was formed in significantly lower enantiomeric excess (6% *ee* vs 23% *ee*). For substrate **S36**, containing a tertiary carbon center, the conversion to the desired products was low (30% overall) and the vinyl ethers were received in a 2.8:1 ratio. Again, the minor regioisomer **S37** showed a higher

enantiopurity, which in this case was 89% *ee*. Depending on the substrate either good regio- or enantioselectivity can be achieved, but it is in mismatch with the observed enantioselectivity. The absolute configuration of the major enantiomer of the products was not determined.

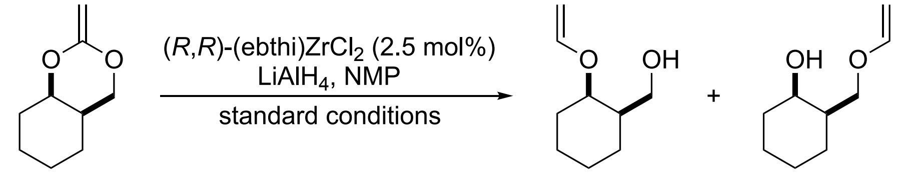

rac-**S32** | **S33** | **S34**

	S33	S34	
	66%, 6% *ee*	8%, 23% *ee*	(8.3:1 ratio)
with *rac*-(ebthi)$ZrCl_2$:	68%	9%	(7.6:1 ratio)

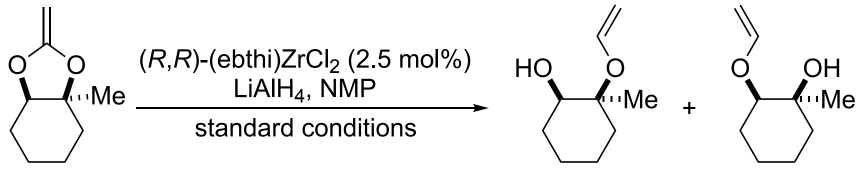

rac-**S35** | **S36** | **S37**

	S36	S37	
	8%, 89% *ee*	22%, 49% *ee*	(1:2.8 ratio)
with *rac*-(ebthi)$ZrCl_2$:	8%	22%	(1:2.8 ratio)

Scheme S2. Regio- and enantioselective opening of further unsymmetric substrates.

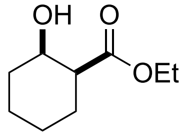

S38

***cis*-Ethyl-2-hydroxycyclohexane-1-carboxylate.** Synthesized according to the literature procedure[22] from ethyl 2-oxocyclohexane-1-carboxylate (19.2 mmol, 1.0 equiv). The crude product was purified by column chromatography (*n*-hexane/Et_2O, 5:4, R_f = 0.2) and obtained as a yellow oil in 42% yield (8.1 mmol). The NMR data matched the literature values. ^1H NMR (500 MHz, $CDCl_3$): δ = 1.27 (t, *J* = 7.2 Hz), 1.29-1.35 (m, 1H), 1.39-1.50 (m, 2H), 1.65-1.73 (m, 3H), 1.84-1.94 (m, 2H), 2.45-2.49 (m, 1H), 3.17-3.18 (m 1H), 4.11f-4.20 (m, 3H). ^{13}C NMR (125 MHz, $CDCl_3$): δ = 14.32, 20.26, 24.15, 24.95, 31.89, 46.83, 60.71, 66.84, 176.08.

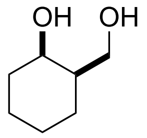

S39

***cis*-2-(Hydroxymethyl)cyclohexan-1-ol.** Synthesized according to the literature procedure[22] from cis-ethyl-2-hydroxycyclohexane-1-carboxylate (1.78 g, 10.4 mmol, 1.0 equiv). The crude product was obtained in analytically pure form in 92% yield (1.24 g, 9.5 mmol) and was used in the next step without further purification. The NMR data match the literature values. ^1H NMR (500 MHz, $CDCl_3$): δ = 1.25-1.34 (m, 1H), 1.40-1.48 (m, 2H), 1.50-1.55 (m, 1H), 1.60-1.71 (m, 4H), 1.76-1.81 (m, 1H), 2.27 (sf, 2H), 3.72-3.78 (m, 2H), 4.14-4.16 (m, 1H). ^{13}C NMR (125 MHz, $CDCl_3$): δ = 20.57, 23.68, 25.11, 33.16, 42.60, 66.56, 70.18.

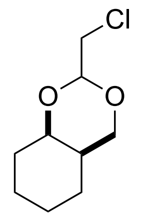

S40

***cis*-2-(Chloromethyl)hexahydro-4H-benzo[d][1,3]dioxine.** Synthesized according to the general procedure for the synthesis of chloromethyldioxolanes from cis-2-(hydroxymethyl)cyclohexan-1-ol (1.24 g, 9.5 mmol, 1.0 equiv). The product was purified by distillation under reduced pressure (0.5 mbar, bp 140°C) and obtained as a colorless oil in 78% yield. (1.42 g, 7.45 mmol). ^{1}H NMR (500 MHz, C_6D_6): δ = 0.64-0.69 (m, 1H), 0.94-1.11 (m, 2H), 1.14-1.19 (m, 1H), 1.25-1.31 (m, 1H), 1.56-1.66 (m, 2H), 1.80-1.85 (m, 1H), 1.97-2.06 (m, 1H), 3.34-3.37 (m, 1H), 3.39-3.43 (m, 3H), 3.46-3.49 (m, 1H), 4.49 (t, *J* = 4.6 Hz, 1H). ^{13}C NMR (125 MHz, C_6D_6): δ = 20.25, 24.64, 25.67, 31.63, 35.33, 44.98, 71.71, 74.44, 100.66. HRMS (pos. APCI): calcd for $C_9H_{15}O_2Cl$ $[M + H]^+$: 191.0833 found: 191.0833. MS (CI): m/z (%) = 208.1 (60) $[M + NH_4]^+$, 191.1 (13), 189.0 (7), 149.2 (7), 142.1 (9), 141.1 (100), 112.1 (3), 95.1 (23), 78.0 (6). IR (ATR): ν [cm^{-1}] = 582, 654, 731, 761, 815, 912, 935, 959, 1019, 1031, 1046, 1100, 1178, 1138, 1191, 1204, 1240, 1338, 1401, 1431, 1448, 1461, 2854, 2934.

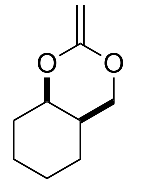

S32

***cis*-2-Methylenehexahydro-4H-benzo[d][1,3]dioxine.** Synthesized according to the general procedure for the synthesis of cyclic ketene acetals from cis-2-(chloromethyl)hexahydro-4H-benzo[d][1,3]dioxine (762.4 mg, 4.0 mmol, 1.0 equiv). The product was purified by distillation under reduced pressure (0.5 mbar, bp 160°C) and obtained as a colorless oil in 61% yield (373.5 mg, 2.42 mmol). ^{1}H NMR (500 MHz, C_6D_6): δ = 0.84-0.93 (m, 2H), 0.98-1.11 (m, 2H), 1.17-1.23 (m, 1H), 1.42-1.48 (m, 1H), 1.50-1.59 (m, 1H), 1.76-1.85 (m, 2H), 3.44 (dd, *J* = 2.3, 10.8 Hz, 1H), 3.62 (dd, *J* = 4.1, 10.7 Hz, 1H), 3.74-3.76 (m, 1H), 3.88-3.89 (m, 2H). ^{13}C NMR (125 MHz, C_6D_6): δ = 19.90, 24.48, 24.81, 30.75, 34.30, 68.57, 70.96, 73.60, 161.54. HRMS (pos. APCI): calcd for $C_9H_{14}O_2$ $[M + H]^+$: 155.1067 found: 155.1071. MS (CI): m/z (%) = 155.1 (100) $[M + H]^+$, 128.2 (3), 112.1 (7), 95.1 (21), 94.1 (11), 81.1 (2). IR (ATR): ν [cm^{-1}] = 567, 606, 733, 812, 873, 904, 920, 951, 973, 998, 1037, 1052, 1072, 1100, 1148, 1170, 1114, 1133, 1207, 1241, 1291, 1291, 1363, 1381, 1447, 1736, 2861, 2931.

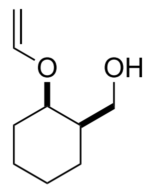

S33

***cis*-2-(Vinyloxy)cyclohexyl)methanol.** Synthesized from *cis*-2-methylenehexahydro-4H-benzo[d][1,3]dioxine (154.2 mg, 1.0 mmol, 1.0 equiv) following the general catalytic desymmetrization procedure. The product was purified by column chromatography (*n*-pentane/Et_2O 4:1 containing 1% (v/v) NEt_3, R_f = 0.2) and obtained as a colorless liquid in 66% yield (102.8 mg, 0.658 mmol) and 6% ee. $[\alpha]_D^{20} = +18.9°$ (*c* 1.2, C_6H_6). ^{1}H NMR (500 MHz, C_6D_6): δ = 1.01-1.11 (m, 2H), 1.23-1.31 (m, 3H), 1.38-1.45 (m, 1H), 1.47-1.61 (m, 3H), 1.80-1.85 (m, 1H), 3.33 (dd, *J* = 5.3, 10.4 Hz, 1H), 3.55 (dd, *J* = 7.6, 10.2 Hz, 1H), 3-93-3.95 (m, 1H), 3.98 (dd, *J* = 1.4, 6.6 Hz, 1H), 4.40 (dd, *J* = 1.4, 14.0 Hz, 1H), 6.19 (dd, *J* = 6.6, 14.0 Hz, 1H). ^{13}C NMR (125 MHz, C_6D_6): δ = 20.78, 24.01, 25.23, 29.49, 43.50, 64.27, 75.53, 88.37,

151.49. HRMS (pos. APCI): calcd for $C_9H_{16}O_2$ $[M + H]^+$: 157.1223 found: 157.1221. MS (CI): m/z (%) = 157.1 (100) $[M + H]^+$, 141.1 (24), 113.1 (4), 112.1 (3), 95.1 (13), 61.1 (4). IR (ATR): ν [cm^{-1}] = 562, 576, 588, 602, 622, 722, 811, 881, 945, 975, 1000, 1079, 1134, 1204, 1320, 1377, 1464, 1610, 2853, 2923, 3430.

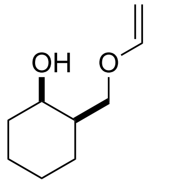

S34

***cis*-2-((Vinyloxy)methyl)cyclohexan-1-ol.** Synthesized from *cis*-2-methylenehexahydro-4H-benzo[d][1,3]dioxine (154.2 mg, 1.0 mmol, 1.0 equiv) following the general catalytic desymmetrization procedure. The product was purified by column chromatography (n-pentane/Et_2O 4:1 containing 1% (v/v) NEt_3, R_f = 0.3) and obtained as a colorless liquid in 8% yield (13.2 mg, 0.08 mmol) and 23% ee. $[\alpha]_D^{20} = -13.2°$ (*c* 0.9, C_6H_6). ^1H NMR (500 MHz, C_6D_6): δ = 1.02-1.21 (m, 3H), 1.24-1.28 (m, 2H), 1.43-1.64 (m, 5H), 3.45 (dd, *J* = 5.6, 9.6 Hz, 1H), 3.65 (dd, *J* = 7.2, 9.8 Hz, 1H), 3.81-3.83 (m, 1H), 3.96 (dd, *J* = 1.8, 6.9 Hz, 1H), 4.23 (dd, *J* = 1.8, 14.3 Hz, 1H), 6.41 (dd, *J* = 6.7, 14.3 Hz, 1H). ^{13}C NMR (125 MHz, C_6D_6): δ = 20.37, 23.53, 25.52, 33.35, 41.46, 66.80, 70.49, 86.40, 152.34. HRMS (pos. APCI): calcd for $C_9H_{16}O_2$ $[M + H]^+$: 157.1223 found: 157.1226. MS (CI): m/z (%) = 157.1 (100) $[M + H]^+$, 141.1 (11), 128.1 (2), 112.1 (3), 95.1 (6), 61.1 (2)., IR (ATR): ν [cm^{-1}] = 561, 568, 575, 587, 602, 611, 625, 659, 720, 809, 976, 1000, 1079, 1204, 1320, 1378, 1464, 1609, 2852, 2923, 3423.

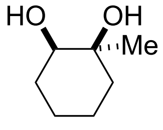

S41

1-Methylcyclohexane-*cis*-1,2-diol. Synthesized from 1-methylcyclohex-1-ene (2.1 ml, 1.7 g, 18.0 mmol, 1.0 equiv) following the general procedure for dihydroxylation of alkenes. The product was purified by column chromatography (cyclohexane/acetone, 2:1, R_f = 0.3) and obtained as a white solid in 67% yield (1.58 g, 12.14 mmol).The NMR data matched the literature values.[23] ^1H NMR (400 MHz, $CDCl_3$): δ = 1.22 (s, 3H), 1.23-1.29 (m, 1H), 1.30-1.41 (m, 2H), 1.46-1.69 (m, 4H), 1.71-1.77 (m, 1H), 2.33 (s, 2H), 3.35-3.38 (m, 1H). ^{13}C NMR (100 MHz, $CDCl_3$): δ = 21.69, 23.25, 26.64, 30.48, 36.98, 71.69, 74.91.

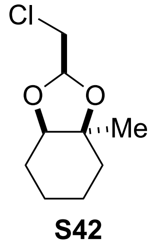

\+

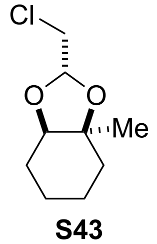

S42 **S43**

***cis,cis*-2-(Chloromethyl)-3a-methylhexahydrobenzo[d][1,3]dioxole** and ***cis,trans*-2-(chloromethyl)-3a-methylhexahydrobenzo[d][1,3]dioxole.** Synthesized according to the general procedure for the synthesis of chloromethyldioxolanes from 1-methylcyclohexane-cis-1,2-diol (2.16 g, 16.5 mmol, 1.0 equiv). The crude product was purified by distillation under reduced pressure (0.05 mbar, bp 130°C) and obtained as a colorless oil in 57% yield as a mixture of diastereomers. (1.79 g, 9.4 mmol).^1H NMR (400 MHz, C_6D_6): δ = 0.74-0.92 (m, 1.84H), 1.01 (d, *J* = 0.9 Hz, 1.78H), 1.06 (s, 3H), 1.08-1.23 (m, 3.01H), 1.30-1.64 (m, 6.95H), 1.81-1.95 (m, 1.67H), 3.22-3.29 (m, 2.07H), 3.35-3.38 (m, 1.75H), 3.53 (t, *J* = 3.0 Hz, 1.01H), 5.07 (t, *J* = 4.3 Hz, 0.57H), 5.18 (t, *J* = 3.8 Hz, 1H). ^{13}C NMR (100 MHz, C_6D_6): δ = 20.01, 20.10, 21.65, 22.24, 22.69, 23.01, 26.65, 26.77, 33.13, 36.05, 45.48, 46.16, 78.90, 79.11,

79.71, 80.77, 100.30, 101.50. HRMS (pos. APCI): calcd for $C_9H_{15}O_2Cl$ [M + NH_4]$^+$: 208.1099 found: 208.1101. MS (CI): m/z (%) = 208.1 (52) [M + NH_4]$^+$, 189.1 (6), 155.1 (11), 141.1 (100), 95.1 (20). IR (ATR): ν [cm^{-1}] = 571, 592, 639, 676, 735, 761, 795, 844, 896, 967, 1003, 1037, 1050, 1086, 1130, 1151, 1207, 1246, 1339, 1378, 1430, 1448, 1734, 2863, 2936.

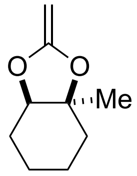

S35

***cis*-3a-Methyl-2-methylenehexahydrobenzo[d][1,3]dioxole.** Synthesized according to the general procedure for the synthesis of cyclic ketene acetals from *cis*,*cis*-2-(chloromethyl)-3a-methylhexahydrobenzo[d][1,3]dioxole and cis,trans-2-(chloromethyl)-3a-methylhexahydrobenzo[d][1,3]dioxole (572.0 mg, 3.0 mmol, 1.0 equiv). The crude product was purified by distillation under reduced pressure (0.5 mbar, bp 74°C) and obtained as a colorless solid in 52% yield (238.9 mg, 1.549 mmol). ^{1}H NMR (400 MHz, C_6D_6): δ = 0.66-0.77 (m, 1H), 0.91-0.97 (m, 1H), 0.98 (d, *J* = 0.8 Hz, 3H), 1.00-1.11 (m, 1H), 1.19-1.33 (m, 3H), 1.64-1.77 (m, 2H), 3.57 (t, *J* = 3.8 Hz, 1H), 3.68-3.71 (m, 2H). ^{13}C NMR (100 MHz, C_6D_6): δ = 19.95, 21.73, 21.89, 26.39, 33.08, 55.64, 80.24, 80.56, 163.40. HRMS (pos. APCI): calcd for $C_9H_{14}O_2$ [M + H]$^+$: 155.1067 found: 155.1067. MS (CI): m/z (%) = 173.1 (100), 171.1 (7), 156.2 (8), 155.1 (75) [M + H]$^+$, 146.2 (2), 130.2 (6). IR (ATR): ν [cm^{-1}] = 563, 567, 575, 567, 579, 582, 586, 589, 593, 596, 599, 602, 606, 613, 619, 625, 687, 697, 796, 926, 1005, 1037, 1054, 1117, 1151, 1186, 1371, 1496, 1813, 1978, 2034, 2157, 2180.

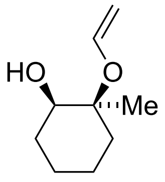

S36

***cis*-2-Methyl-2-(vinyloxy)cyclohexan-1-ol.** Synthesized from *cis*-3a-methyl-2-methylenehexahydrobenzo[d][1,3]dioxole (77.1 mg, 0.5 mmol, 1.0 equiv) following the general catalytic desymmetrization procedure. The product was purified by column chromatography (n-pentane/Et_2O 5:1 containing 1% (v/v) NEt_3, Rf= 0.1) and obtained as a colorless liquid in 8% yield (6.6 mg, 0.04 mmol) and 89% ee (abs. conf. not determined). $[\alpha]_D^{20} = +4.1°$ (*c* 0.03, C_6H_6). ^{1}H NMR (500 MHz, C_6D_6): δ = 0.86-0.97 (m, 3H), 1.02-1.08 (m, 4H), 1.51-1.59 (m, 2H), 1.62-1.70 (m, 2H), 1.78 (s, 1H), 4.09 (dd, *J* = 0.6, 6.1 Hz, 1H), 4.60 (dd, *J* = 0.6, 13.6 Hz, 1H), 6.26 (dd, *J* = 6.3, 13.6 Hz, 1H). ^{13}C NMR (125 MHz, C_6D_6): δ = 21.32, 21.91, 30.17, 30.65, 74.92, 79.00, 91.73, 145.65. HRMS (pos. APCI): calcd for $C_9H_{16}O_2$ [M + NH_4]$^+$: 174.1489 found: 174.1489. MS (CI): m/z (%) = 157.1 (91) [M + H]$^+$, 155.1 (100), 141.1 (42), 130.1 (17), 113.1 (24), 95.1 (17). IR (ATR): ν [cm^{-1}] = 555, 559, 564, 567, 575, 579, 582, 587, 596, 607, 635, 785, 846, 938, 1044, 1097, 1234, 1372, 1447, 1737, 2985.

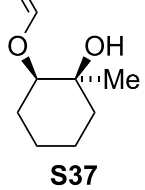

S37

***cis*-1-Methyl-2-(vinyloxy)cyclohexan-1-ol.** Synthesized from *cis*-3a-methyl-2-methylenehexahydrobenzo[d][1,3]dioxole (77.1 mg, 0.5 mmol, 1.0 equiv) following the general catalytic desymmetrization procedure. The product was purified by column chromatography (n-pentane/Et_2O 5:1 containing 1% (v/v) NEt_3, Rf= 0.2) and obtained as a colorless liquid in

22% yield (16.8 mg, 0.11 mmol) and 49% ee (abs. conf. not determined). $[\alpha]_D^{20} = -2.8°$ (*c* 0.07, C_6H_6). ^{1}H NMR (500 MHz, C_6D_6): δ = 0.91-0.99 (m, 1H), 1.05-1.12 (m, 4H), 1.15-1.22 (m, 1H), 1.45-1.55 (m, 2H), 1.59-1.74 (m, 3H), 1.84 (s, 1H), 3.18-3.20 (m, 1H), 3.98 (dd, *J* = 1.4, 6.4 Hz, 1H), 4.37 (dd, *J* = 1.5, 14.0 Hz, 1H), 6.11 (dd, *J* = 6.4, 14.0 Hz, 1H). ^{13}C NMR (125 MHz, C_6D_6): δ = 21.70, 26.79, 27.12, 37.54, 70.67, 83.51, 88.46, 151.44. HRMS (pos. APCI): calcd for $C_9H_{16}O_2$ $[M + H]^+$: 157.1223 found: 157.1225. MS (CI): m/z (%) = 174.2 (10), 157.1 (100) $[M + H]^+$, 155.1 (62), 141.1 (35), 130.1 (30), 113.1 (40), 112.2 (16), 95.1 (30). IR (ATR): ν [cm^{-1}] = 551, 555, 559, 564, 567, 570, 575, 580, 588, 593, 596, 601, 607, 611, 617, 630, 635, 666, 685, 692, 809, 926, 1005, 1075, 1158, 1194, 1375, 1461, 1634, 2012, 2031, 2162, 2857, 2927, 3538.

Kinetic Isotope Effect Experiments

We carried out kinetic isotope effect experiments to support the presence of a higher activation barrier for the hydrozirconation than for the β-O-elimination step (Figure S2). The ^{1}H NMR spectrum of the product received from the standard catalysis with $LiAlH_4$ was used as a reference (Figure S2a, an integral value of 0.96 corresponded to 100% H at the vinylic position).

First, a stoichiometric experiment was carried out, in which a 1:1 mixture of pre-generated Zr-H and Zr-D species, formed by mixing *rac*-(ebthi)$ZrCl_2$ with a 1:1 mixture of $LiAlH_4$ and $LiAlD_4$ and allowing for equilibration for 15 min, was added to a mixture of **1a** and NMP. Here, a positive kinetic isotope effect in form of only 40% deuterium incorporation at the vinylic position, corresponding to k_H/k_D = 1.5 was observed (see Figure S2b). Keeping in mind that the hydrozirconation is usually a reversible event, this indicated that a slow hydrozirconation was followed by a rapid β-O-elimination.
In this experiment, we kept the overall amount of $LiAlH_4$ and $LiAlD_4$ identical to the catalytic conditions, but used 2.0 equiv of (ebthi)$ZrCl_2$. We used this excess in zirconocene to ensure that the substrate would be consumed before catalyst turnover would occur. This also accounted for small amounts being consumed in a potential overreduction of **2a**. Furthermore, the reaction was quenched after 3 min based on the observation that the catalysis with 2.5 mol% was complete after 4 h. This would also avoid a slow overreaction of the precipitated intermediate. The experiment was not further optimized and **2a** was received in 21% yield.

We carried out two additional experiments:
1) A catalytic reaction with a 1:1 mixture of $LiAlH_4$/$LiAlD_4$ and 2.5 mol% *rac*-(ebthi)$ZrCl_2$, which showed an almost identical KIE of k_H/k_D = 1.6:1 (Figure S2c). Since this experiment was run under turnover conditions, the catalyst regeneration could in principle have contributed to this value as well. The fact that it is close to the value from the stoichiometric experiment, however, supports an overall rate-limiting hydrozirconation.
2) A control experiment consisting of a catalysis run with $LiAlD_4$ only (Figure S2d), which led to 100% D incorporation and spectra for comparison were recorded.

In conclusion, the results were in agreement with the scenario in Scheme 5 and Figure 1 of the manuscript.

Stoichiometric competition experiment with pre-generated Zr-H and Zr-D species

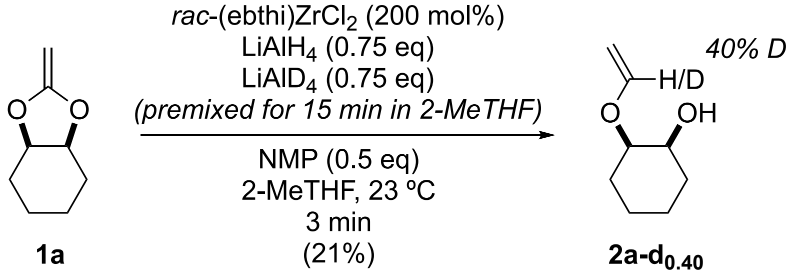

A flame dried 10 ml Schlenk tube equipped with a magnetic stir bar was charged with *rac*-(ebthi)$ZrCl_2$ (85.3 mg, 0.2 mmol, 2.0 equiv). 2-MeTHF (870 µl) was added. Then, a solution of $LiAlH_4$ in 2-MeTHF (2.3 M, 33 µl, 2.8 mg, 0.075 mmol, 0.75 equiv) and a solution of $LiAlD_4$ in 2-MeTHF (2.3 M, 33 µl, 3.1 mg, 0.075 mmol, 0.75 equiv) were added and the reaction mixture was stirred at room temperature for 15 min. A separate flame dried 10 ml Schlenk flask equipped with a magnetic stir bar was charged with *cis*-2-methylenehexahydro-benzo[d][1,3]dioxole (**1a**, 14.0 mg, 0.1 mmol, 1.0 equiv) in 2-MeTHF (1 ml) and *N*-methyl-pyrrolidine (5 µl, 4.3 mg, 0.05 mmol, 0.5 equiv). This mixture was vigorously stirred. Then, the contents of the Schlenk tube containing the pre-formed Zr-H/Zr-D species were added to the Schlenk flask containing the substrate via syringe. The resulting reaction mixture was stirred

for 3 min at room temperature. Afterwards, it was quenched by addition of 10% (w/w) aq. Rochelle salt solution (5 ml). The layers were separated and the aqueous layer was extracted with CH_2Cl_2 (3 × 10 ml). The combined organic layers were dried over Na_2SO_4, filtered, and the solvent was removed under reduced pressure. The product was purified by column chromatography (*n*-pentane/EtOAc 4:1 containing 1% (v/v) NEt_3, R_f = 0.3) and obtained as a colorless oil in 21% yield (3.0 mg, 0.021 mmol). The reaction product showed a deuterium incorporation of 40%. ^{1}H NMR (400 MHz, C_6D_6): δ = 0.90-1.07 (m, 2H), 1.20-1.31 (m, 2H), 1.37-1.46 (m, 1H), 1.52-1.62 (m, 1H), 1.67-1.78 (m, 3H), 3.46-3.49 (m, 1H), 3.56-3.62 (m, 1H), 3.97-3.99 (m, 1H), 4.34-4.38 (m, 1H), 6.10 (d, *J* = 6.5, 14.1 Hz, 0.61H). ^{2}H NMR (77 MHz, C_6H_6): δ = 6.09 (s).

Catalytic experiment with a 1:1 mixture of $LiAlH_4$ and $LiAlD_4$

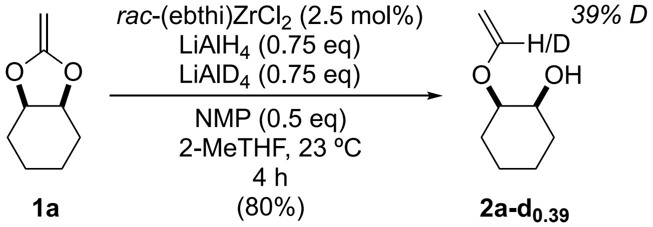

1a **2a-d$_{0.39}$**

A flame dried and argon filled 10 ml Young tube equipped with a magnetic stir bar was charged with *rac*-(ebthi)$ZrCl_2$ (5.3 mg, 12.5 µmol, 2.5 mol%). A solution of $LiAlH_4$ in 2-MeTHF (2.3 M, 0.16 ml, 0.375 mmol, 0.75 equiv) and a solution of $LiAlD_4$ in 2-MeTHF (2.3 M, 0.16 ml, 0.375 mmol, 0.75 equiv) were added dropwise simultaneously. Afterwards, *cis*-2-methylenehexahydrobenzo[d][1,3]dioxole (**1a**, 0.5 mmol, dissolved in 0.5 ml of 2-MeTHF) and *N*-methylpyrrolidine (26 µl, 21.3 mg, 0.25 mmol, 0.5 equiv) were added dropwise. The tube was sealed and the reaction mixture was stirred for 4h at room temperature. The reaction was quenched by dropwise addition of 10% (w/w) aq. Rochelle salt solution (2 ml) and CH_2Cl_2 (2 ml). The reaction mixture was transferred into a separatory funnel, the layers were separated and the aqueous layer was extracted with CH_2Cl_2 (3 × 5 ml). The combined organic layers were dried over Na_2SO_4, filtered, and the solvent was removed under reduced pressure. The product was purified by column chromatography (*n*-pentane/CH_2Cl_2 3:1 containing 1% (v/v) NEt_3, Rf = 0.3) and obtained as a colorless liquid in 80%yield (56.5 mg, 0.397 mmol). The reaction product showed a deuterium incorporation of 39%. ^{1}H NMR (400 MHz, C_6D_6): δ = 0.92-1.09 (2H), 1.21-1.32 (m, 2H), 1.38-1.47 (m, 1H), 1.53-1.63 (m, 1H), 1.68-1.81 (m, 3H), 3.47-3.51 (m, 1H), 3.61 (s, 1H), 3.98-4.01 (m, 1H), 4.35-4.39 (m, 1H), 6.08-6.14 (m, 0.59H). ^{13}C NMR (100 MHz, C_6D_6): δ = 21.41, 22.10, 26.94, 30.69, 69.28, 79.42, 89.00, 150.79. ^{2}H NMR (77 MHz, C_6D_6): δ = 1.30 (s), 6.18 (m). IR (ATR): ν [cm^{-1}] = 499, 597, 668, 813, 848, 881, 974, 1017, 1038, 1078, 1113, 1178, 1260, 1321, 1367, 1448, 1612, 1635, 1732, 2858, 2935, 3433.

Control experiment with $LiAlD_4$

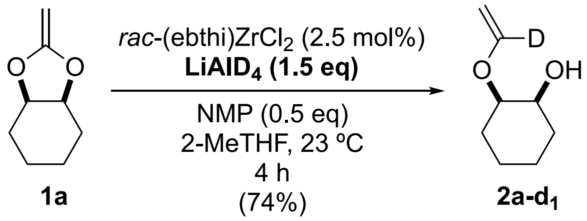

1a **2a-d$_1$**

***cis*-2-((Vinyl-1-d)oxy)cyclohexan-1-ol.** Synthesized from *cis*-2-methylenehexahydrobenzo[d][1,3]dioxole (**1a**) following the general catalytic desymmetrization procedure with the following modifications: *rac*-(ebthi)$ZrCl_2$ (2.1 mg, 5 µmol, 2.5 mol%) was used as catalyst precursor and $LiAlD_4$ in 2-MeTHF (2.3 M 0.13 ml, 0.3 mmol, 1.5 equiv) was used as reducing agent. The product was purified by column chromatography (*n*-pentane/CH2Cl2 3:1 containing 1% (v/v) NEt_3, Rf= 0.3) and obtained as a colorless liquid in 74% yield (21.1 mg, 0.148 mmol).

^{1}H NMR (400 MHz, C_6D_6): δ = 0.87-1.09 (m, 2H), 1.22-1.23 (m, 2H), 1.38-1.47 (m, 1H), 1.54-1.64 (m, 1H), 1.69-1.80 (m, 2H), 1.88 (s, 1H), 3.48-3.52 (m, 1H), 3.61-3.63 (m, 1H), 3.99 (s, 1H), 4.35-4.37 (m, 1H). ^{2}H NMR (77 MHz, C_6D_6): δ = 1.29 (s), 6.08-6.12 (m). ^{13}C NMR (500 MHz, C_6D_6): δ = 21.42, 22.10, 26.92, 30.70, 68.91, 79.33, 88.73- 88.76 (m), 150.25, 150.52-150.79 (m). IR (ATR): ν [cm^{-1}] = 691, 732, 802, 917, 1019, 1048, 1081, 1155, 1185, 1223, 1260, 1296, 1321, 1347, 1373, 1427, 1449, 1559, 1631, 2160, 2885, 2935, 3153, 3437.

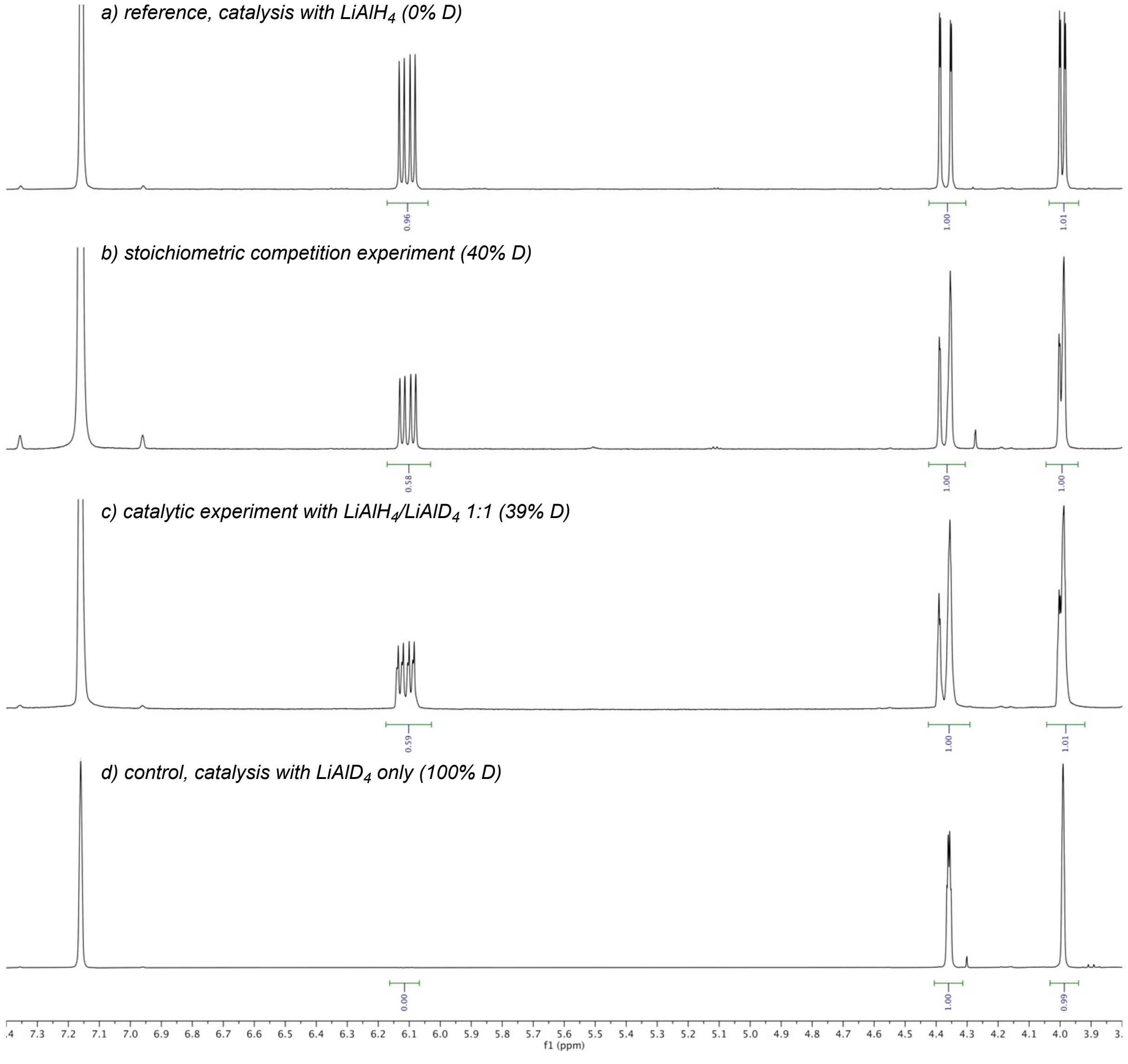

Figure S2. Comparison of ^{1}H NMR spectra in C_6D_6 (400 MHz). a) product from the standard reaction with $LiAlH_4$ only. The integration value of 0.96 was used as 100% reference. b) Product from the stoichiometric competition experiment with pre-formed Zr-H/Zr-D species. c) Catalytic experiment with a 1:1 mixture of $LiAlH_4$ and $LiAlD_4$. d) Product from the catalysis with $LiAlD_4$ only.

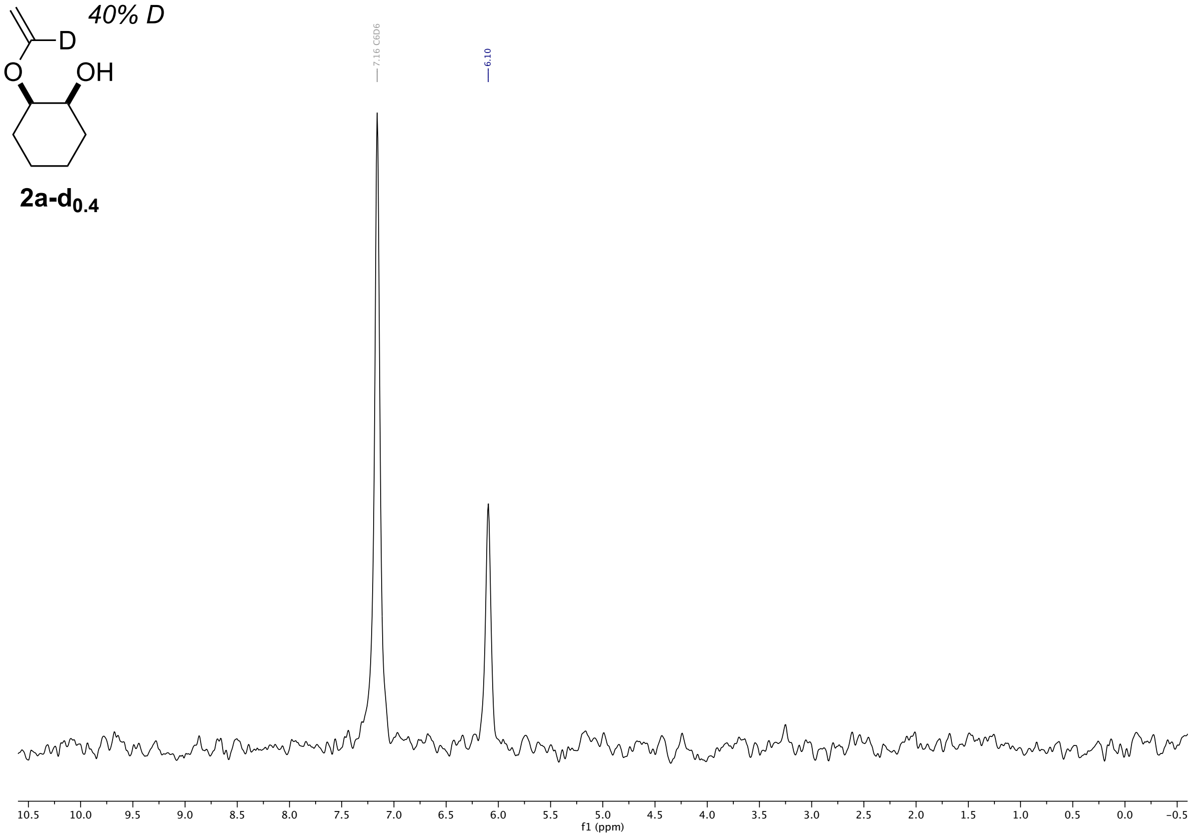

Figure S3. ^{2}H NMR (61 MHz, C_6H_6, 1000 scans) spectrum of the product obtained from the stoichiometric experiment (Figure 2b).

Initial Reaction Optimization and Experiments with other Catalysts

Screening Results and Discussion of Quenching Byproducts

The experiments for the reaction optimization followed the above general procedure but with the changes noted in Table S1. The initial conditions were adapted from our previous works.[24] Table S1 also notes the formation of byproducts as determined by ^{1}H NMR analysis of the crude reaction mixture using the isolated yield of **2a** as reference. The formation of **S1** results from an overreaction in which product **2a** undergoes an additional hydrozirconation/β-O-elimination event. Byproducts **S44**, **S45**, and **S46** are formed during the quench of the reaction. Ketene acetal **1a** was not observed in the crude NMR and the byproducts **S1**, and **S44**–**S46** usually accounted for the remaining mass balance. However, none of the byproducts were observed under the optimized conditions.

Table S1. Expanded Screening Table.

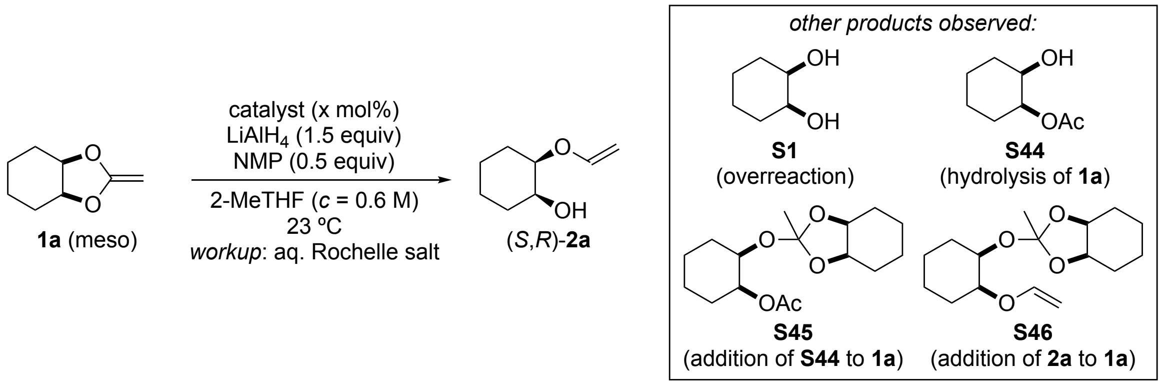

Entry	Catalyst	x / mol%	*t* / h	Yield (2a) / %	*ee* / %	Sideproducts / % conversion[a]
1	**cat-1**	5.0	0.5	10	93	**S44**: 17%, **S45**: 17%, **S46**: 15%
2	**cat-1**	5.0	1	36	92	**S44:** 17%, **S45:** 6%, **S46:** 18%
3	**cat-1**	5.0	2	76	96	**S44:** 8%, **S45:** 3%, **S46:** 14%
4	**cat-1**	5.0	4	50	92	**S1**: 52%
5	**cat-1**	5.0	16	11	92	**S1**: 33%
**6**	**cat-1**	**2.5**	**4**	**82**	**92**	**none**
7	**cat-1**	1.0	4	12	92	**S44:** 28%, **S45:** 17%, **S46:** 14%
8[b]	**cat-1**	2.5	4	68	92	**S45:** 3%, **S46:** 14%
9[c]	**cat-1**	2.5	4	46	92	**S1**: 51%
10	**cat-2**	2.5	4	0	--	**S44:** 10%, **S45:** 78%, **S46:** 9%[d]
11	**cat-3**	2.5	4	77	26	**S1**: 16%
12	**cat-4**	2.5	4	26	4	**S44:** 16%, **S45:** 19%, **S46:** 19%
13	**cat-5**	2.5	4	39	24	**S44:** 38%, **S45:** 18%, **S46:** 11%
14	**cat-6**	2.5	4	3	79	**S44:** 73%, **S45:** 2%

[a] Determined from the ratio observed in the crude NMR mixture using the yield of isolated **2a** as reference. [b] Reaction without NMP. [c] Reaction in THF as solvent. [d] The yield of isolated **S45** was used as reference for the determination of the conversion.

S44 originates from the hydrolysis of unreacted **1a** during aqueous workup with Rochelle salt solution. The formation of the addition products **S45** and **S46** are also formed during the *slow addition* of the Rochelle salt solution. Here, if deprotonated **S44** or **2a** (the excess of $LiAlH_4$ used in the reaction leads to deprotonation of **S44** and **2a**) and larger amounts of unreacted **1a** are present, these alcoholates outcompete water in the addition to the acetal carbon center and, resulting in the formation of **S45** or **S46**, respectively. Byproduct **S44** was first isolated in the first optimization experiments with **cat-1**. Byproducts **S45** and **S46** were isolated from the separate experiments with Cp_2ZrCl_2 and $Cp^*{}_2ZrCl_2$ as catalysts as follows.
*In conclusion, all three byproducts **S44–S46** are formed from **1a** and **2a**. Hence, in these cases, only the remaining substrate **1a** was present in the reaction mixture next to product **2a** prior to the quench.*

We tested the racemic catalysts Cp_2ZrCl_2 and $Cp^*{}_2ZrCl_2$, a non-bulky/less electron-rich and a particularly bulky/more electron-rich zirconocene dichloride, respectively, in the ring-opening reaction. The reaction with Cp_2ZrCl_2 as catalyst resulted in a very low yield with the hydrolysis

products of **1a** (**S44** and **S45**) constituting the major material. This indicated that the remaining material (prior to workup) was unreacted substrate, which had not undergone hydrozirconation (Scheme S3). In earlier stoichiometric studies, it was proposed that hydrozirconation of simple alkenes with $Cp_2Zr(H)Cl$ was slower in comparison to $MeCp_2Zr(H)Cl$ due to a lower solubility of the former.[25] However, under our catalytic conditions all catalyst was dissolved. In comparison, the well-soluble precatalyst **cat-4**, possessing one alkyl substituent at each Cp, gave a slightly higher yield than Cp_2ZrCl_2. **cat-3**, having overall 4 alkyl Cp substituents showed a good reactivity and **cat-1** (6 alkyl substituents) the highest yield. We therefore propose an positive electronic influence of the alkyl substituents on the hydrozirconation step.
The bulkiness of $Cp^*{}_2ZrCl_2$, however, appears to hinder its approach to **1a**, increasing the barrier for the hydrozirconation step and slowing the reaction (30% yield). Here, the remaining material also consisted only of the substrate hydrolysis products **S44** and **S45**, as well as a small quantity of **S46** (2% conversion), which were formed during the workup. Again, this showed that the remaining material was **1a** (before workup), which had not yet undergone hydrozirconation.

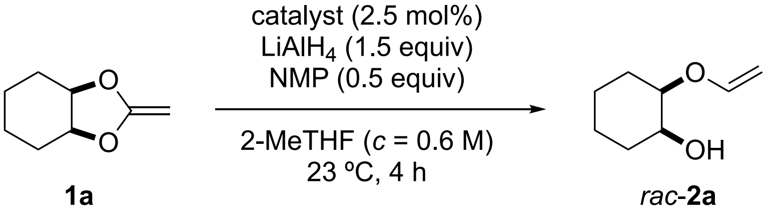

1a *rac*-**2a**

Cp_2ZrCl_2 : 8% (**S44**: 82%, **S45**: 4%)[a]
$Cp^*{}_2ZrCl_2$: 30% (**S44**: 63%, **S45**: 2%, **S46**: 2%)[a]

Scheme S3. Reaction outcome with Cp_2ZrCl_2 and $Cp^*{}_2ZrCl_2$ as catalyst under the optimized conditions. Yields of isolated **2a**. [a] Conversions determined from the ratio observed in the crude NMR mixture using the yield of **2a** as reference.

Overall, this shows that the water-sensitive **1a** was the remaining material before the aqueous workup. As no other byproducts were found, this is in an agreement with a scenario of a rate-limiting hydrozirconation. The hypothetical occurrence of formally hydrogenated **1a** (the acetaldehyde acetal), for example, would have indicated a rate-limiting β-O-elimination, but such products were never observed or isolated. A detailed mechanistic investigation will be needed to give further insight on this point and how the individual steps of the catalytic cycle are influenced by the various catalyst motifs.
Furthermore, we propose that the (ebthi)$ZrCl_2$ precatalyst is having the right balance of electron-richness to ensure a rapid hydrozirconation and the right sterical bulk paired with a bridge-locked conformation to give a high asymmetric induction. In comparison, **cat-3**, which contains a longer biphenylene instead of an ethylene bridge, also undergoes rapid hydrozirconation. We thus propose that the high reactivity originates from the presence of overall four methyl substituents at the cyclopentadienyl ligands rather than a bridging unit. However, **cat-3** does not have the correct chiral pocket for a high stereodiscrimination in the following β-O-elimination and, thus, it leads only to a low enantioselectivity (26% *ee*).

Characterization of Hydrolysis Byproducts S44–S46

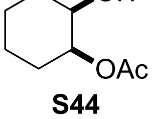

S44

***cis*-2-Hydroxycyclohexyl acetate.** The product was observed in the crude reaction mixture of entry 1,3,4,5,7,8, was purified by column chromatography (*n*-pentane/EtOAc 4:1, R_f =0.2) and obtained as a colorless oil. ^{1}H NMR (400 MHz, C_6D_6): δ = 0.83-1.00 (m, 2H), 1.05-1.19 (m, 2H), 1.27-1.34 (m, 2H), 1.70 (s, 3H), 1.80-1.85 (m, 1H), 1.87-1.93 (m, 1H), 2.00 (s, 1H), 3.36-3.42 (m, 1H), 4.64-4.70 (m, 1H). ^{13}C NMR (100 MHz, C_6D_6): δ = 20.81, 23.79, 23.91, 30.14,

33.35, 72.62, 77.96, 170.57. HRMS (pos. APCI): calcd for $C_8H_{14}O_3$ [M + H]$^+$: 159.1016 found: 159.1017. MS (CI): m/z (%) = 159.1 (100) [M + H]$^+$, 157.1 (8). 98.1 (1). IR (ATR): ν [cm^{-1}] = 555, 559, 567, 575, 578, 587, 607, 675, 839, 851, 892, 909, 997, 1035, 1075, 1162, 1241, 1370, 1453, 1479, 1716, 1735, 2864, 2940, 3036, 3442.

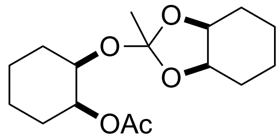

S45

***cis*-2-((*cis*-2-Methylhexahydrobenzo[d][1,3]dioxol-2-yl)oxy)cyclohexyl acetate**. The product was observed in the crude reaction mixture of entry 1,3,4,5,7,8, was purified by column chromatography (*n*-pentane/Et_2O 4:1, R_f =0.2) and obtained as a yellow oil.^{1}H NMR (400 MHz, C_6D_6): δ = 0.86-1.02 (m, 2H), 1.08-1.18 (m, 2H), 1.28-1.51 (m, 9H), 1.58-1.65 (m, 2H), 1.67 (s, 3H), 1.78 (s, 3H), 1.83-1.99 (m, 2H), 3.98-4.00 (m, 1H), 4.14-4.18 (m, 1H), 5.08-5.10 (m,1H). ^{13}C NMR (100 MHz, C_6D_6): δ = 20.00, 20.11, 21.04, 22.37, 24.43, 27.50, 27.71, 27.94, 30.18, 30.43, 69.94, 73.13, 74.00, 74.22, 120.97, 169.92. MS (ESI): calcd for $C_{16}H_{26}O_5$ [M + NH_4]$^+$: 316.2 found: 316.3. IR (ATR): ν [cm^{-1}] = 835, 862, 888, 934, 962, 995, 1015, 1047, 1072, 1111, 1143, 1199, 1238, 1377, 1449, 1734, 2860, 2937.

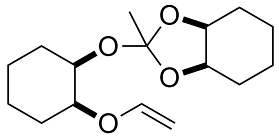

S46

***cis*-2-Methyl-2-((*cis*-2-(vinyloxy)cyclohexyl)oxy)hexahydrobenzo[d][1,3]dioxole.** The product was observed in the crude reaction mixture of entry 1,3,4,8, was purified by column chromatography (*n*-pentane/EtOAc 4:1, R_f =0.6) and obtained as a colorless oil as a mixture of diastereomers. ^{1}H NMR (500 MHz, C_6D_6): δ = 0.79-0.92 (m, 2H), 1.10-1.38 (m, 6H), 1.51-1.59 (m, 2H), 1.62-1.69 (m, 2H), 1.72 and 1.73 (2 x s, 3H), 1.82-1.95 (m, 3H), 2.02-2.12 (m, 1H), 3.12-3.18 (m, 1H), 3.47-3.54 (m, 1H), 3.89-3.92 (m, 1H), 4.01-4.03 (m, 1H), 4.14-4.21 (m, 1H), 4.51-4.55 (m, 1H), 6.36-6.42 (m, 1H). ^{13}C NMR (125 MHz, C_6D_6): δ = 14.28, 21.90, 21.97, 22.05, 22.14, 22.99, 23.70, 23.73, 25.27, 25.32, 27.19, 28.29, 28.38, 28.84, 29.39, 30.15, 30.36, 31.91, 70.41, 71.04, 78.73, 78.88, 80.89, 81.15, 81.64, 81.69, 87.81, 87.88, 122.36, 122.43, 128.00, 151.94, 152.07. HRMS (pos. APCI): calcd for $C_{16}H_{26}O_4$ [M + H]$^+$: 283.1904 found: 283.1910. MS (CI): m/z (%) = 283.1 (1) [M + H]$^+$, 160.2 (6), 143.1 (41), 142.2 (9), 141.1 (100), 139.9 (2), 116.1 (1), 98.1 (1), 81.1 (3), 60.2 (1). IR (ATR): ν [cm^{-1}] = 555, 567, 574, 582, 670, 833, 1035, 1134, 1190, 1380, 1478, 1815, 1960, 3036, 3071, 3091.

Synthesis of Zirconocene Catalysts cat-4 and cat-5

cat-4 was prepared following an optimized procedure by Gansäuer et al.[26]

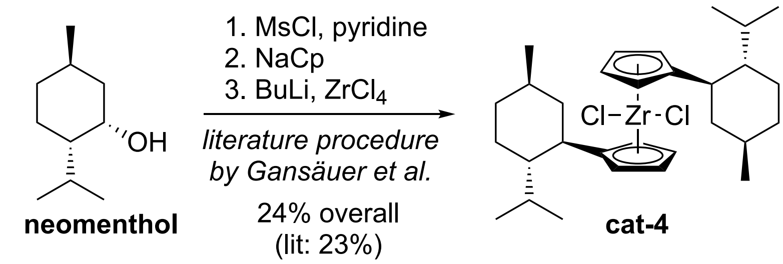

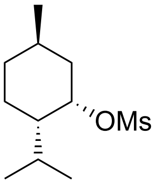

S47

Neomenthyl mesylate. Neomenthol (7.81 g, 50.0 mmol) was reacted with mesyl chloride following the literature procedure.[26] Neomenthyl mesylate was obtained as a yellow oil in analytically pure form in 88% yield (10.29 g, 43.93 mmol) and was used in the next step without further purification. ^{1}H NMR (400 MHz, $CDCl_3$): δ =0.87-0.95 (m, 7H), 0.98-1.06 (m, 4H), 1.11-1.18 (m, 1H), 1.27-1.38 (m, 1H), 1.52-1.61 (m, 1H), 1.71-1.83 (m, 2H), 2.19-2.25 (m, 1H), 3.00 (s, 3H), 5.15 (s, 1H). ^{13}C NMR (100 MHz, $CDCl_3$): δ = 20.77, 20.87, 22.16, 24.42, 26.17, 28.98, 34.63, 39.34, 40.37, 47.68, 52.70, 81.47.

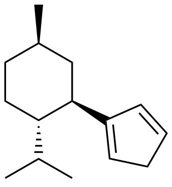

S48

(1*S*,2*R*,4*R*)-2-(Cyclopentadienyl)-1-isopropyl-4-methylcyclohexane. Neomenthyl mesylate (**S47**, 7.03 g, 30.0 mmol) was reacted with sodium cyclopentadienide according to the literature procedure but using commercial NaCp solution (2.4 M in THF).[26] The crude product was filtered through a short pad of silica (*n*-pentane, R_f = 0.8). The colorless oil obtained after concentration under reduced pressure (5.26 g, 25.7 mmol, 86%) was directly used in the next step. NMR analysis showed a mixture of double bond isomers of (1*S*,2*R*,4*R*)-2-(cyclopentadienyl)-1-isopropyl-4-methylcyclohexane. Furthermore, the crude material contained 15% of menthene. ^{1}H NMR (400 MHz, $CDCl_3$): δ = 0.70 (d, *J* = 6.9 Hz, 3H), 0.83 (d, *J* = 7.0 Hz, 3H), 0.88 (d, *J* = 6.5 Hz, 3H), 0.92-0.99 (m, 2H), 1.03-1.12 (m, 2H), 1.22-1.31 (m, 2H), 1.36-1.48 (m, 1H), 1.64-1.71 (m, 2H), 1.71-1.79 (m, 1H), 2.33-2.44 (m, 1H), 2.93-2.95 (m, 1H), 5.93-5.99 (m, 0.71H), 6.14-6.16 (m, 0.34H), 6.24-6.26 (m, 0.36H), 6.39-6.43 (m, 0.86H), 6.47-6.49 (m, 0.53H).

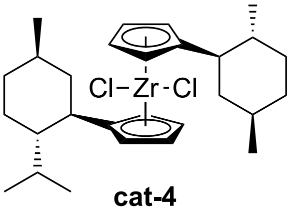

cat-4

Bis{η^5[(*1R,2S,5R*)-5-methyl-2-(prop-2-yl)-cyclohex-1-yl]cyclopentadienyl}-zirconium dichloride. The crude substituted cyclopendatiene **S48** prepared in the previous step (495 mg, 2.06 mmol, 2.06 equiv) was reacted with zirconocene tetrachloride according to the literature procedure. The product was washed with *n*-pentane (3 × 5 ml), recrystallized from

CH_2Cl_2 and obtained as a white solid in 32% yield (180 mg, 0.317 mmol). ^1H NMR (400 MHz, $CDCl_3$): δ = 0.78 (d, *J* = 6.9 Hz, 6H), 0.81 (d, *J* = 6.8 Hz, 6H), 0.86-0.99 (m, 10H), 1.06-1.20 (m, 4H), 1.40-1.51 (m, 4H), 1.66-1.72 (m, 2H), 1.75-1.81 (m, 2H), 1.83-1.88 (m, 2H), 2.61-2.67 (m, 2H), 6.08-6.10 (m, 4H), 6.32-6.34 (m, 2H), 6.54-6.56 (m, 2H). ^{13}C NMR (100 MHz, $CDCl_3$): δ = 15.64, 21.75, 22.90, 24.79, 27.38, 32.75, 35.33, 41.43, 41.66, 50.94, 106.54, 114.24, 116.28, 119.17, 139.97.

cat-5 was prepared following the literature procedure by Erker et al.[27]

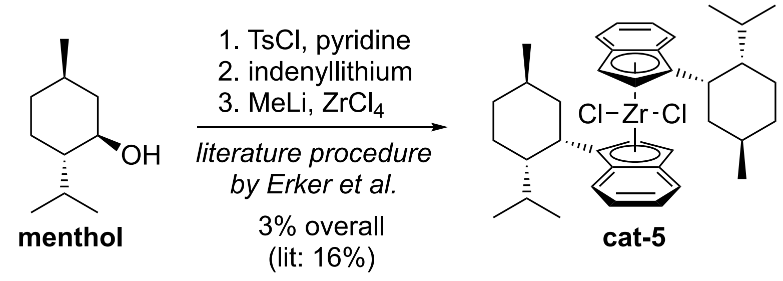

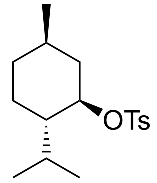

S49

Menthyl tosylate. Synthesized from (–)-menthol (12.0 g, 76.8 mmol) according to the literature procedure.[27] The crude product was obtained as a white solid in analytically pure form in 77% yield (18.2 g, 58.7 mmol) and it was used in the next step without further purification. The NMR data matched the literature values. ^1H NMR (400 MHz, $CDCl_3$): δ = 0.52 (d, *J* = 8.1 Hz, 3H), 0.80-0.87 (m, 4H), 0.90 (d, *J* = 6.6 Hz, 3H), 0.91-1.02 (m, 1H), 1.13-1.22 (m, 1H), 1.33-1.46 (m, 2H), 1.61-1.68 (m, 2H), 1.86-1.93 (m, 1H), 2.11-2.15 (m, 1H), 2.44 (s, 3H), 4.37-4.43 (m, 1H), 7.31-7.33 (m. 2H), 7.79-7.81 (m, 2H). ^{13}C NMR (100 MHz, $CDCl_3$): δ = 15.38, 20.97, 21.74, 21.99, 23.10, 25.61, 31.79, 33.90, 42.10, 47.72, 83.84, 126.14, 127.80, 129.04, 129.79, 134.92, 144.45.

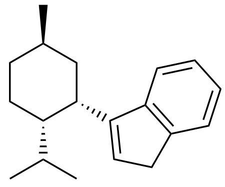

S50

3-((1*S*,2*S*,5*R*)-2-Isopropyl-5-methylcyclohexyl)-1*H*-indene. Synthesized according to the literature procedure from **S49** (11.5 g, 37.1 mmol).[27] The crude product was purified by column chromatography (*n*-pentane, Rf = 0.8) and obtained as a colorless oil in 34% yield (3.62 g, 14.2 mmol). The NMR data matched the literature values. ^1H NMR (400 MHz, $CDCl_3$): δ = 0.72 (d, *J* = 6.6 Hz, 3H), 0.79 (d, *J* = 6.6 Hz, 3H), 0.92 (d, *J* = 6.7 Hz, 3H), 0.96-1.03 (m, 1H), 1.20-1.28 (m, 2H), 1.44-1.56 (m, 1H), 1.58-1.69 (m, 2H), 1.78-1.82 (m, 2H), 1.89-1.94 (m, 1H), 3.34-3.37 (m, 3H), 6.36-6.38 (m, 1H), 7.18-7.22 (m, 1H), 7.28-7.32 (m, 1H), 7.37-7.39 (m, 1H), 7.46-7.48 (m, 1H). ^{13}C NMR (100 MHz, $CDCl_3$): δ = 21.54, 21.69, 22.54, 26.90, 27.36, 30.16, 34.22, 35.72, 38.48, 39.67, 47.73, 118.93, 123.74, 124.41, 126.09, 129.59, 143.91, 144.68, 146.74.

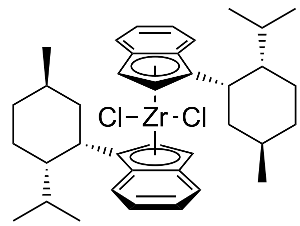

cat-5

Bis{η^5[(*1S,2S,5R*)-5-methyl-2-(prop-2-yl)-cyclohex-1-yl]indenyl}-zirconium dichloride. Synthesized according to the literature procedure from **S50** (495 mg, 2.06 mmol, 2.06 equiv).[27] The crude product was washed with *n*-pentane (3 × 5 ml). Crystallization from CH_2Cl_2 gave **cat-5** as a yellow solid in 11% yield (71.0 mg, 0.11 mmol). The NMR data matched the literature values. ^{1}H NMR (400MHz, $CDCl_3$): δ = –0.03 (d, *J* = 6.7Hz, 6H), 0.62 (d, *J* = 6.7Hz, 6H), 0.97 (d, *J* = 6.5 Hz, 6H), 1.01-1.07 (m, 2H), 1.16-1.36 (m, 6H), 1.46-1.56 (m, 4H), 1.81-1.84 (m, 2H), 2.06-2.09 (m, 2H), 2.15-2.18 (m, 2H), 3.68-3.72 (m, 2H), 5.55 (m, 2H), 6.60-6.61 (m, 2H), 7.14-7.18 (m, 2H), 7.32-7.35 (m, 2H), 7.67 (d, *J* = 8.5Hz, 2H), 7.75 (d, *J* = 8.7Hz, 2H). ^{13}C NMR (100 MHz, $CDCl_3$): δ = 18.41, 22.39, 23.76, 29.02, 31.09, 34.35, 36.25, 39.19, 48.27, 98.15, 122.63, 124.66, 125.31, 125.98, 126.28, 126.83, 128.59.

Conditions for the Determination of Enantiomeric Excess

Table S2. Conditions for Determination of enantiomeric excess.

product	assay conditions	t_{major} (min)	t_{minor} (min)	ee (%)	Sign of $[\alpha]_D$
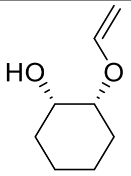 (*S*,*R*)-**2a**	GC<br>Hydrodex-β-TBDAc<br>RAMP from 60°C to 200°C, 5°C/min	18.4	18.0	92%	–
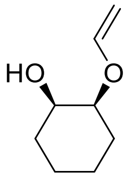 (*R*,*S*)-**2a**	HPLC: IA<br>*n*-heptane/iPrOH 98:2<br>210nm,<br>0.5 ml/min	15.5	16.9	92%	+
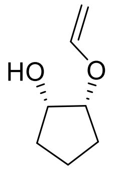 **2b**	GC<br>Hydrodex-β-TBDAc<br>RAMP from 60°C to 200°C, 5°C/min	17.4	17.2	90%	+
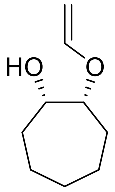 **2c**	HPLC: OD-H<br>*n*-heptane/iPrOH 99:1<br>210 nm,<br>0.5 ml/min	28.0	33.0	88%	+
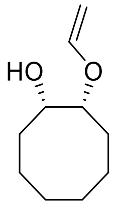 **2d**	HPLC: OD-H<br>n-heptane/iPrOH 98:2<br>210 nm,<br>0.5 ml/min	10.9	12.2	83%	+
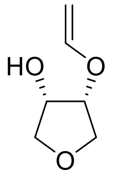 **2e**	GC<br>Hydrodex-β-TBDAc<br>RAMP from 80°C to 120°C, 1°C/min	47.1	46.6	90%	+

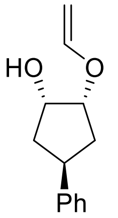 **2f**	HPLC: OD-H<br>*n*-heptane/iPrOH 98:2<br>210 nm,<br>0.5 ml/min	23.0	24.9	78%	+
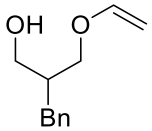 **2g**	HPLC: IA<br>*n*-heptane/iPrOH 95:5<br>254 nm,<br>0.7 ml/min	11.8	10.8	33%	−
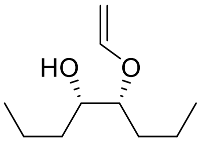 **2h**	HPLC: AD-3<br>*n*-heptane/iPrOH<br>99.5:0.5<br>212 nm,<br>0.5 ml/min	10.5	9.2	84%	+
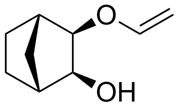 **2i**	HPLC: OD-H<br>*n*-heptane/iPrOH 99:1<br>210nm,<br>0.5 ml/min	14.5	15.9	94%	−
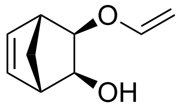 **2j**	HPLC: OD-H<br>*n*-heptane/iPrOH<br>99.5:0.5<br>210 nm,<br>0.5 ml/min	16.3	17.3	86%	−
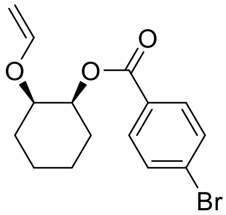 **S26**	HPLC: IA<br>*n*-heptane/iPrOH<br>99.5:0.5<br>210nm,<br>0.5 ml/min	14.2	9.4	91%	+
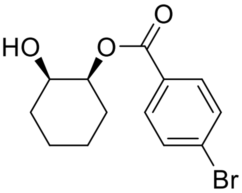 **S27**	HPLC: IA<br>*n*-heptane/iPrOH 95:5<br>210nm,<br>0.5 ml/min	29.1	31.1	91%	+
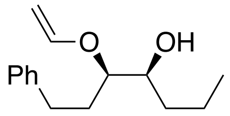 **4** + 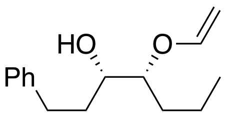 **5**	HPLC: OD-3<br>*n*-heptane/iPrOH<br>99.5:0.5<br>212nm,<br>0.5 ml/min	13.9<br>15.0	11.1<br>20.5	83%<br>78%	−
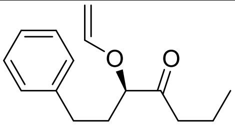 **S28** 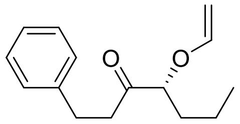 **S29**	HPLC: AD-3<br>*n*-heptane/iPrOH<br>99.7:0.3<br>212nm,<br>0.5 ml/min	15.9<br>24.9	18.1<br>21.5	79%<br>75%	+

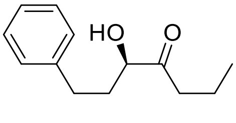 **S30** 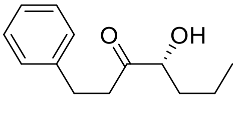 **S31**	HPLC: AD-3<br>*n*-heptane/iPrOH<br>99.5:0.5<br>212nm,<br>0.5 ml/min	13.0<br>15.9	14.0<br>15.2	78%<br>75%	+
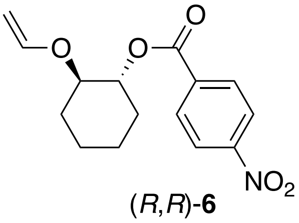 (*R*,*R*)-**6**	HPLC: IA<br>*n*-heptane/iPrOH<br>90:10<br>254nm,<br>0.5 ml/min	13.8	12.0	92%	–
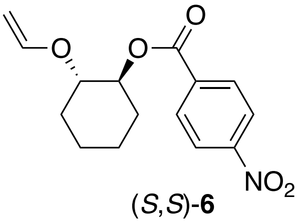 (*S*,*S*)-**6**	HPLC: IA<br>*n*-heptane/iPrOH<br>90:10<br>254nm,<br>0.5 ml/min	12.0	13.9	93%	+
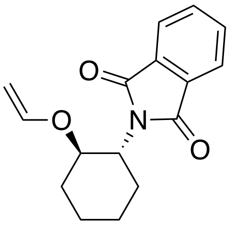 (*R*,*R*)-**7**	HPLC: IA<br>*n*-heptane/iPrOH<br>90:10<br>254nm,<br>0.5 ml/min	11.6	14.2	91%	–
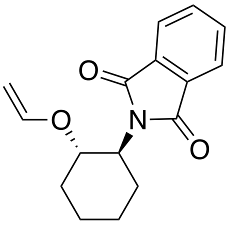 (*S*,*S*)-**7**	HPLC: IA<br>*n*-heptane/iPrOH<br>90:10<br>254nm,<br>0.5 ml/min	14.2	11.6	92%	+
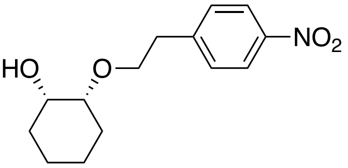 (*S*,*R*)-**8**	HPLC: IA<br>*n*-heptane/iPrOH 95:5<br>254 nm,<br>0.5 mL/min	41.6	38.1	92%	–
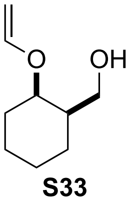 **S33**	HPLC: OD-H<br>*n*-heptane/iPrOH 99:1<br>210 nm,<br>0.5 mL/min	36.9	35.3	6%	+
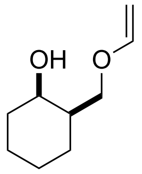 **S34**	GC<br>Hydrodex-β-TBDAc<br>ISO 120°C	11.0	10.6	23%	–

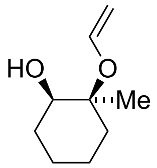 **S36**	HPLC: OD-3<br>*n*-heptane/iPrOH 99:1<br>212 nm,<br>0.5 mL/min	6.9	6.1	89%	+
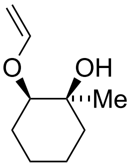 **S37**	HPLC: AD-3<br>*n*-heptane/iPrOH<br>99.5:0.5<br>212 nm,<br>0.5 mL/min	11.7	14.2	49%	–

Computational Studies

General remarks

Methods
The Orca 4.2.1 program package was used for the DFT calculations.[28] Initial conformer searches were carried out using the CREST 2.11 program package using the GFN2-xTB level of theory with implicit solvation (THF).[29] Afterwards, a manual search for potentially lower conformers was carried out. The structure optimizations were finalized using the pheh-3c method.[30,31] The conductor-like polarizable continuum model (CPCM)[32] was applied for the optimizations using 2-MeTHF as solvent (eps = 6.97; refract = 1.408, value at 20 ºC).[33] Frequency analyses were carried out analytically. Stationary points (minimum structures) were characterized by the absence of imaginary frequencies, transition states were characterized by the presence of one imaginary frequency that matched the transition state trajectory. The correction to the Gibbs Free Energy was obtained from the Orca output of the frequency calculation. Single-point calculations were carried out using the PW6B95[34] functional together with the def2-QZVP basis set[35] and matching auxiliary basis sets.[36] The single point calculations were carried out with the *Grid4 FinalGrid5* option and CPCM(2-MeTHF) solvation. The RI-J approximation for Coulomb integrals and the COSX numerical integration for HF exchange (RIJCOSX) were applied.[37,38] Furthermore, the D4 dispersion correction was applied.[39] The correction $\Delta G^{*\rightarrow o}_{solv}$ (= 1.90 kcal mol^{-1}, see Born-Haber cycle in Scheme S4) was added to obtain the energy in solution.
The computed structures shown herein were visualized using CYLview 2.0.[40]

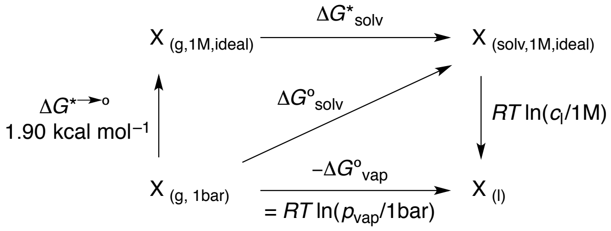

Scheme S4. Interconversion Scheme for the calculation of solvation energies for a compound X.

Chloride-Hydride vs Pseudo-Cationic Chloride-Hydride vs Dihydride Pathways
Initially, we calculated the catalytic cycle and potential transition states using the chloride complex (*R*,*R*)-(ebthi)Zr(H)Cl. It was found that the transition states were for hydrozirconation and β-O-elimination were energetically too high to be reasonable. Alternative pseudo-cationic pathways could be identified that were more favorable. Here, the chloride ligand dissociated from the titanium center to about 3.5 Å distance and was kept in the periphery through hydrogen bonds with the ebthi ligand and the C-H hydrogens of the substrate. This gave more space at the zirconium center and allowed an energetically more favorable orientation of the ebthi ligand, which resulted in about 10 kcal mol^{-1} lower transition state energy in the β-O-elimination.
A zirconium dihydride catalyst, however, lowered again the energy of the transition states and intermediates, rendering the reaction feasible at room temperature as discussed in the manuscript. Considering that the formation of zirconocene dihydrides from the corresponding dichloride in presence of an excess of $LiAlH_4$ is well known in the literature,[41] this was concluded to be the most plausible scenario.

Conformers and Isomers Considered
The structures of all intermediates were subject to a conformer search using automated (CREST) and manual searches. Different conformers of the tetrahydroindenyl and cyclohexyl motifs as well as rotamers of the substrate-catalyst complexes were considered.
For the intermediates and transition states, H-C-H (methylene carbon in the center between the two hydrides) and a H-H-C (a hydride in the center) arrangements at the zirconium center were considered for the hydrozirconation step. However, the H-H-C arrangement resulted in

the formation of H_2. Likewise, H-C-O and H-O-C were considered for the β-O-elimination event. In analogy, the corresponding chloride-hydride and the pseudo-cationic pathways were investigated (*vide infra*). In general, the X-C-Y isomer (methylene carbon in the center) was energetically favored. This was in agreement with an earlier calculation of the hydrozirconation of propylene with $Cp_2Zr(H)Cl$, which also showed a preference for the H-C-Cl arrangement.[42]

Reaction with the Dihydride Catalyst (as in Scheme 5/Figure 1)

Hydrozirconation transition states (rel. energies of the barriers in kcal mol^{-1}):

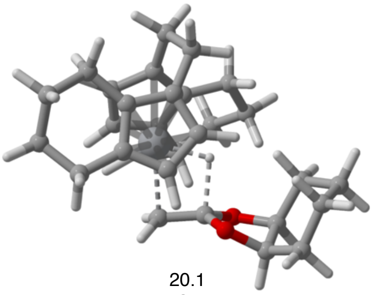

20.1
TS-9
(concave)

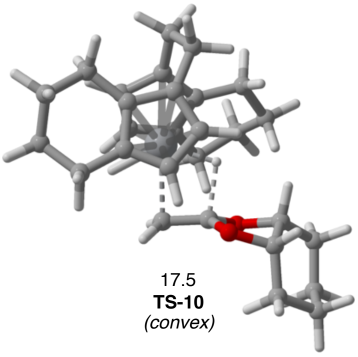

17.5
TS-10
(convex)

β-O-Elimination transition states (concave pathway, rel. energies of the barriers in kcal mol^{-1}):

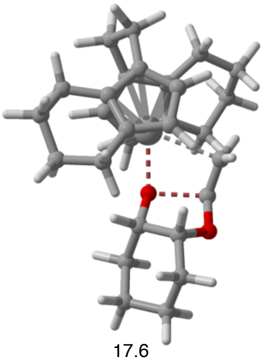

17.6
TS-11a (*S*,*R*)
(= TS-11' in manuscript)

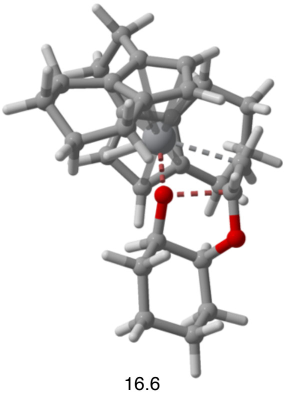

16.6
TS11b (*R*,*S*)

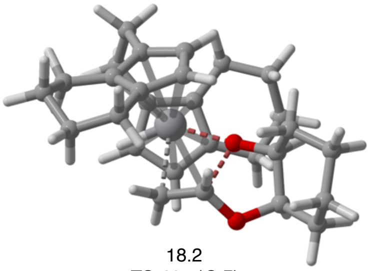

18.2
TS-11c (*S*,*R*)

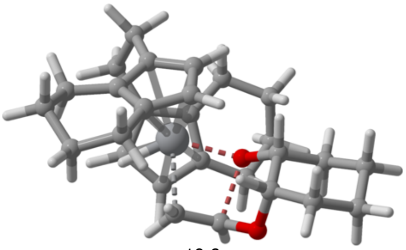

10.6
TS11d (*R*,*S*)
(= TS-11 in manuscript)

β-O-Elimination transition states (convex pathway, rel. energies of the barriers in kcal mol^{-1}):

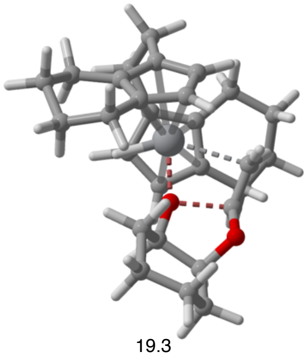

19.3
TS-12a (*R*,*S*)

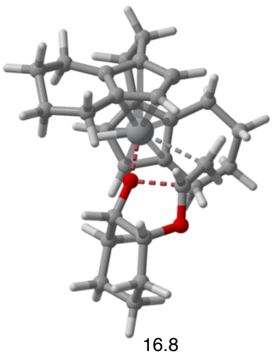

16.8
TS12b (*S*,*R*)

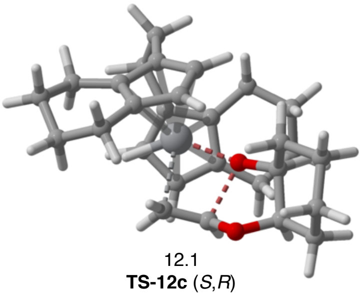

12.1
TS-12c (*S*,*R*)
(= TS-12 in manuscript)

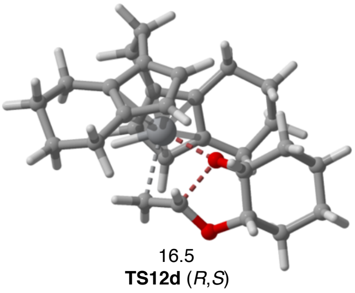

16.5
TS12d (*R*,*S*)
(= TS-12' in manuscript)

Table S3. Data for the structures calculated along the concave dihydride pathway.

structure	pheh-3c energy / Hartree	PW6B95-D4-CPCM/def2-QZVP / Hartree	correction to *G* / kcal mol^{-1}	G_{solv} / kcal mol^{-1}	ν_{imag} / cm^{-1}
**1a**	–461.523908	–463.309765	103.46	–290625.91	none
(*R*,*R*)-(ebthi)ZrH_2	–1624.994974	–1629.273631	235.15	–1022147.59	none
**TS-9**	–2086.503442	–2092.577308	356.82	–1312753.37	–590.45
**9**	–2086.548547	–2092.611181	358.21	–1312773.23	none
**TS-11a** (= **TS-11'**)	–2086.508686	–2092.582723	357.70	–1312755.88	–482.10
**TS-11b**	–2086.51188	–2092.584467	357.81	–1312756.87	–457.01
**TS-11c**	–2086.510021	–2092.582436	358.09	–1312755.31	–422.02
**TS-11d** (= **TS-11**)	–2086.523488	–2092.594885	358.31	–1312762.91	–406.89
**11**	–2086.587094	–2092.652153	356.77	–1312800.38	none

Table S4. Data for the additional structures calculated along the convex dihydride pathway.

structure	pheh-3c energy / Hartree	PW6B95-D4-CPCM/def2-QZVP / Hartree	correction to *G* / kcal mol^{-1}	G_{solv} / kcal mol^{-1}	ν_{imag} / cm^{-1}
**TS-10**	–2086.507411	–2092.581235	356.60	–1312756.05	–557.07
**10**	–2086.548925	–2092.611642	357.94	–1312773.79	none
**TS-12a**	–2086.50554	–2092.579883	357.64	–1312754.16	–501.45
**TS-12b**	–2086.509897	–2092.583829	357.57	–1312756.71	–504.57
**TS-12c** (= **TS-12**)	–2086.519655	–2092.592495	358.29	–1312761.43	–394.77
**TS-12d** (= **TS-12'**)	–2086.512145	–2092.586591	359.05	–1312756.96	–430.07
**12**	–2086.586405	–2092.650228	357.14	–1312798.80	none

Alternative Zirconium Chloride-Hydride Pathways (not shown in the manuscript)

Starting from (*R*,*R*)-(ebthi)Zr(H)Cl, it was found that the hydrozirconation was again favored via an attack from the convex side of the ketene acetal (Scheme S5). In addition to the Cl-C-H arrangement (**TS-A** and **TS-B**), the isomers having a Cl-H-C arrangement were calculated (**TS-iso-A** and **TS-iso-B**). As already mentioned, these were significantly higher in energy ($\Delta\Delta G$= 8 and 9.5 kcal mol^{-1}, respectively). The corresponding barriers for the β-O-elimination towards the product complexes **C** and **D** (**TS-C** and **TS-D**, respectively), however, were found to be slightly higher than for the hydrozirconation. Here, the one leading to the *R*,*S*-product complex **D** via the concave pathway was favored by 1.0 kcal. This small difference and preference for the opening towards the *R*,*S*-enantiomer of the product was not in agreement with the observed selectivity.

It was found that displacing the chloride ligand from the zirconium center into the ligand periphery was possible, leading to Zr-Cl bond lengths of 4.3–4.6 Å. The chloride was held in place by several hydrogen bonds and the space made available around the now cationic zirconium center lowered the barrier for the β-O-elimination to 21.1 kcal mol^{-1} (convex, **TS-C$_{cat}$**) and 19.3 kcal mol^{-1} (concave, **TS-D$_{cat}$**). Again, the transition state leading to the *R*,*S*-product was lower in energy. However, since the hydrozirconation was rate- and selectivity-determining (as in the dihydride scenario shown in the manuscript), the *S*,*R*-product arising from TS-C' would be favored. The energy difference in the two now selectivity-determining transition states **TS-A** and **TS-B** corresponded to $\Delta\Delta G$ = 2.3 kcal mol^{-1} (corresponds to 96% ee). Nevertheless, the barriers via the dihydride scenario were even lower and, thus, in better agreement with our experiments.

The snapshots below show the four transition states that were evaluated for the hydrozirconation step and the conformers and isomers that were calculated for the chloride and pseudo-cationic β-elimination via the concave and convex pathways (4 each, 16 overall).

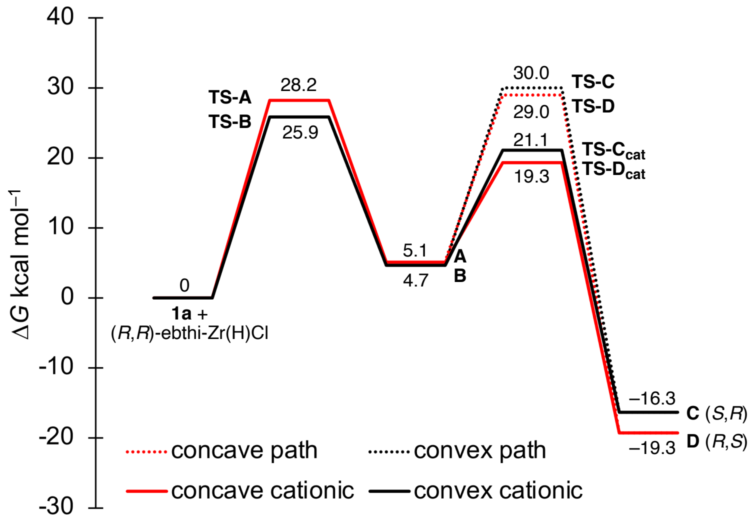

Scheme S5. Energy profile for the reaction with (*R*,*R*)-(ebthi)Zr(H)Cl. The β-O-elimination was calculated with the chloride coordinated to the zirconium center (dots) and with a cationic Zr center, the chloride being coordinated only via hydrogen bonds. Gibbs free energies in kcal mol^{-1}.

Hydrozirconation transition states (relative barrier energies in kcal mol^{-1}):

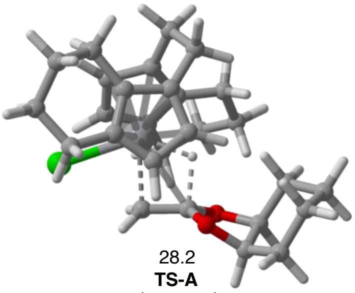

28.2
TS-A
(concave)

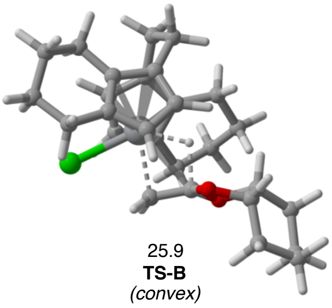

25.9
TS-B
(convex)

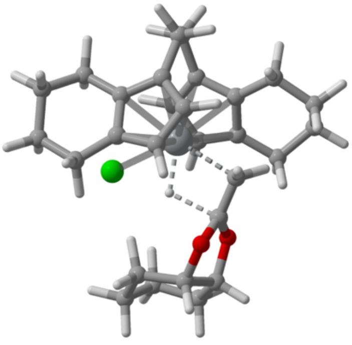

36.2
TS-iso-A
(concave)

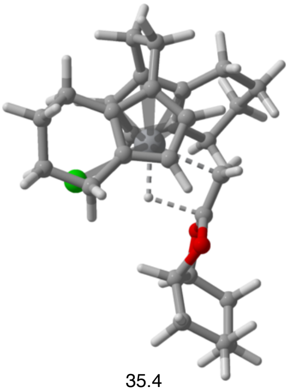

35.4
TS-iso-B
(convex)

β-O-Elimination transition states **TS-Ca–e** (convex pathway, relative barrier energies in kcal mol^{-1}):

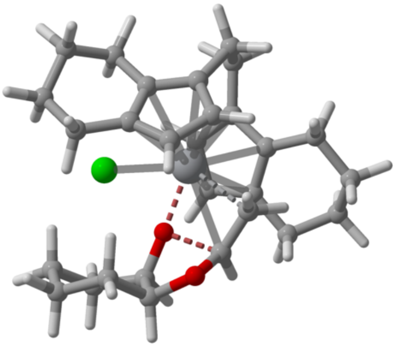

37.1
TS-Ca (*R*,*S*)
(convex)

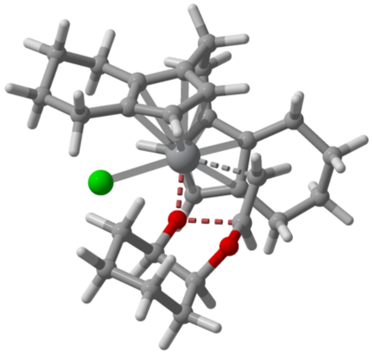

31.8
TS-Cb (*R*,*S*)
*(as **TS-Ca**, but other chair conformer)*

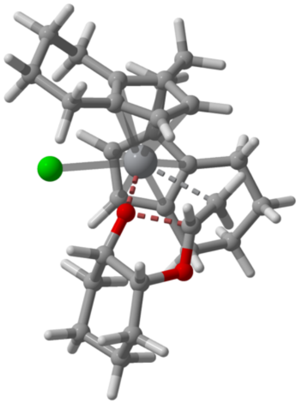

30.0
TS-Cc (*S*,*R*)
(convex)

designated TS-C

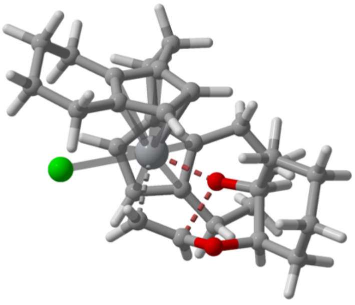

33.0
TS-Cd (*S*,*R*)
(convex)

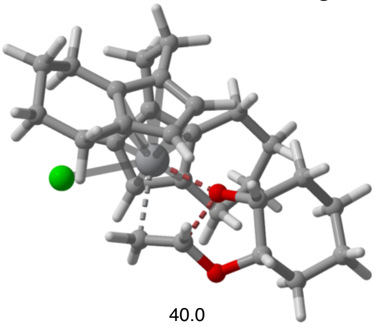

40.0
TS-Ce (*R*,*S*)
(convex)

β-O-Elimination transition states **TS-Da–e** (concave pathway, relative barrier energies in kcal mol^{-1}):

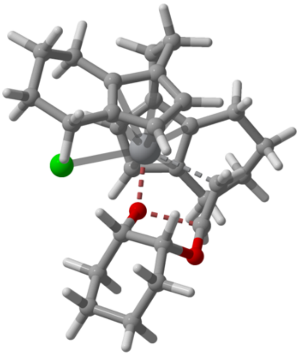

32.0
TS-Da (*S*,*R*)
(concave)

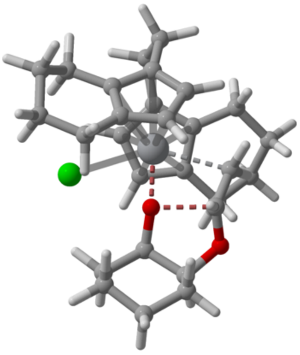

30.5
TS-Db (*R*,*S*)
(concave)

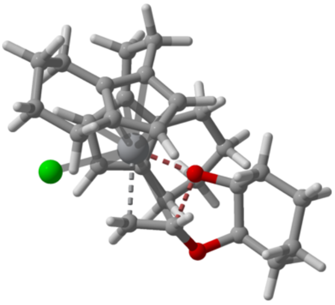

37.9
TS-Dc (*S*,*R*)
(concave)

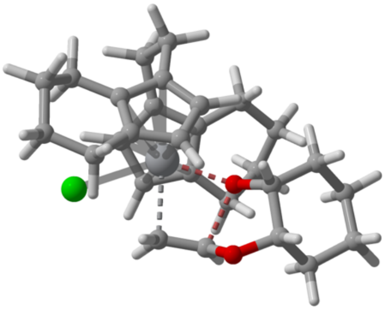

29.0
TS-Dd (*R*,*S*)
(concave)

designated TS-D

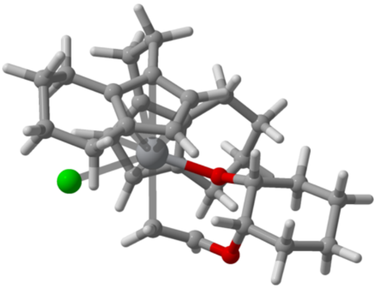

29.8
TS-De (*R*,*S*)
*(as **TS-Dd**, but other chair conformer)*

β-O-Elimination transition states **TS-C$_{cat}$a–e** (convex pathway, pseudo-cationic, relative barrier energies in kcal mol^{-1}):

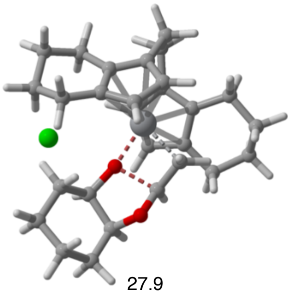

27.9
TS-C$_{cat}$a (*R*,*S*)
(convex)

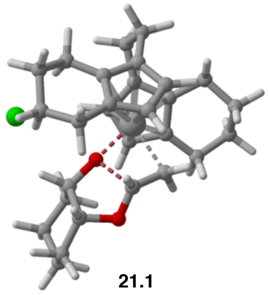

21.1
TS-C$_{cat}$b (*S*,*R*)
(convex)

designated TS-C$_{cat}$

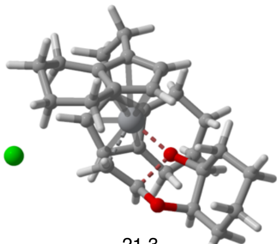

21.3
TS-C$_{cat}$c (*S*,*R*)
(convex)

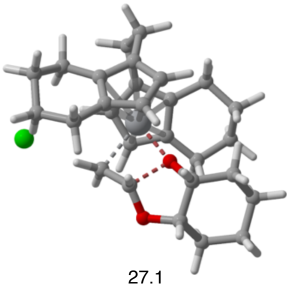

27.1
TS-C$_{cat}$d (*R*,*S*)
(convex)

β-O-Elimination transition states **TS-D$_{cat}$a–e** (concave pathway, pseudo-cationic, relative barrier energies in kcal mol^{-1}):

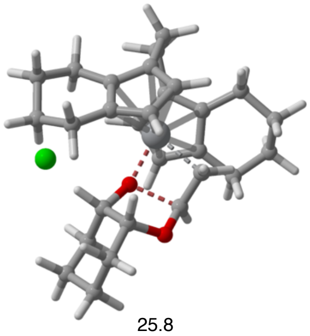

25.8
TS-D$_{cat}$a (*S*,*R*)
(concave)

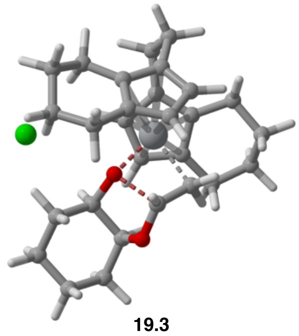

19.3
TS-D$_{cat}$b (*R*,*S*)
(concave)
designated TS-D$_{cat}$

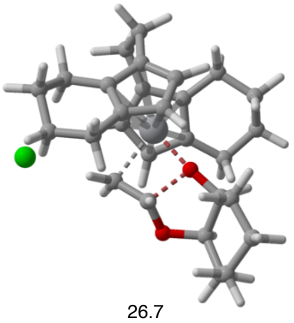

26.7
TS-D$_{cat}$c (*S*,*R*)
(concave)

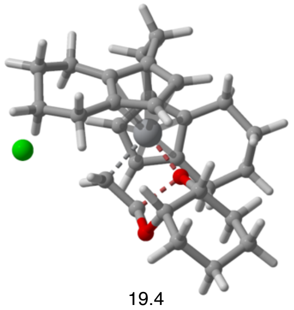

19.4
TS-D$_{cat}$d (*R*,*S*)
(concave)

Table S5. Data for the structures calculated along the convex chloride-hydride pathway (for **1a**, see Table S3).

structure	pheh-3c energy / Hartree	PW6B95-D4-CPCM/def2-QZVP / Hartree	correction to G / kcal mol^{-1}	G_{solv} / kcal mol^{-1}	ν_{imag} / cm^{-1}
(*R*,*R*)-ebthi-Zr(H)Cl	-2084.204498	-2089.352416	232.50	-1310854.04	none
**TS-B**	-2545.693949	-2552.643010	351.68	-1601454.09	–708.86
**TS-iso-B**	-2545.676907	-2552.627040	351.17	-1601444.58	–310.58
**B**	-2545.745952	-2552.680864	354.28	-1601475.25	none
**TS-Ca**	-2545.677023	-2552.627811	353.38	-1601442.85	–490.49
**TS-Cb**	-2545.686408	-2552.635127	352.65	-1601448.18	–469.95
**TS-Cc (= TS-C)**	-2545.689447	-2552.638820	353.21	-1601449.93	–456.12
**TS-Cd**	-2545.682565	-2552.633291	352.74	-1601446.93	–376.90
**TS-Ce**	-2545.669352	-2552.622876	352.94	-1601440.20	–438.48
**C**	-2545.776392	-2552.711318	352.38	-1601496.26	none

Table S6. Data for the additional structures calculated along the concave chloride-hydride pathway.

structure	pheh-3c energy / Hartree	PW6B95-D4-CPCM/def2-QZVP / Hartree	correction to G / kcal mol^{-1}	G_{solv} / kcal mol^{-1}	ν_{imag} / cm^{-1}
**TS-A**	-2545.689988	-2552.639643	351.94	-1601451.72	–755.78
**TS-iso-A**	-2545.678104	-2552.626462	351.6	-1601443.79	–341.97
**A**	-2545.744546	-2552.680567	354.51	-1601474.83	none
**TS-Da**	-2545.686627	-2552.634282	352.37	-1601447.93	–471.79
**TS-Db**	-2545.689052	-2552.638019	353.15	-1601449.49	–393.06
**TS-Dc**	-2545.673745	-2552.625523	352.78	-1601442.02	–418.02
**TS-Dd (=TS-D)**	-2545.689205	-2552.641501	353.87	-1601450.96	–330.14
**TS-De**	-2545.689701	-2552.639374	353.36	-1601450.13	–402.67
**D**	-2545.779716	-2552.716159	352.47	-1601499.20	none

Table S7. Data for the additional pseudo-cationic transition states calculated along the convex chloride-hydride pathway.

structure	pheh-3c energy / Hartree	PW6B95-D4-CPCM/def2-QZVP / Hartree	correction to *G* / kcal mol^{-1}	G_{solv} / kcal mol^{-1}	ν_{imag} / cm^{-1}
**TS-C$_{cat}$a**	-2545.689631	-2552.642861	353.67	-1601452.01	–410.86
**TS-C$_{cat}$b** (= **TS-C$_{cat}$**)	-2545.702433	-2552.654149	353.94	-1601458.82	–387.48
**TS-C$_{cat}$c**	-2545.701612	-2552.653093	353.49	-1601458.61	–398.37
**TS-C$_{cat}$d**	-2545.690253	-2552.644221	353.67	-1601452.86	–419.96

Table S8. Data for the additional pseudo-cationic transition states calculated along the concave chloride-hydride pathway.

structure	pheh-3c energy / Hartree	PW6B95-D4-CPCM/def2-QZVP / Hartree	correction to *G* / kcal mol^{-1}	G_{solv} / kcal mol^{-1}	ν_{imag} / cm^{-1}
**TS-D$_{cat}$a**	-2545.693856	-2552.645364	353.15	-1601454.10	–412.53
**TS-D$_{cat}$b (= TS-D$_{cat}$)**	-2545.685926	-2552.656355	353.47	-1601460.68	–393.40
**TS-D$_{cat}$c**	-2545.69206	-2552.644148	353.19	-1601453.30	–437.88
**TS-D$_{cat}$d**	-2545.705455	-2552.656234	353.57	-1601460.50	–411.39

Coordinates

In the order of appearance as in Table S3–Table S8.

Dihydride Scenario

1a

```
22
Coordinates from ORCA-job sp_pw6b95
  C   1.08088555999447     -2.19567443129143     -1.00713257777001
  C   0.93613617035558     -3.24567730768371     -0.20278032026225
  O   1.72962928875789     -4.33525808149104     -0.21925700869232
  O   -0.02115444738688     -3.36734997159344      0.73440482269607
  C   1.45384794109382     -5.08399123934314      0.97061268979104
  C   -0.00977099305422     -4.72558621977462      1.19553637635479
  C   -0.51773527116327     -4.84332324231442      2.61573178337526
  H   -0.64637393652312     -5.32044154939123      0.52924928462256
  C   2.38924103712860     -4.65531459257311      2.09699221897527
  H   1.57886447528590     -6.14303378555271      0.74086401536102
  C   1.82636465626049     -5.01754787773190      3.46654426087965
  H   2.53781803960688     -3.57141453945741      2.06032537810598
  H   3.37049985983942     -5.10762495982785      1.93985391458049
  C   0.47042650156197     -4.35232736009972      3.66602246454405
  H   -0.71687645090039     -5.90535279473807      2.78733058917950
  H   -1.48229981852081     -4.33753960789394      2.69870447444216
  H   1.72204115036939     -6.10372685585259      3.56302755912976
  H   2.52780776299490     -4.70400849630393      4.24172468656285
  H   0.07609452509438     -4.57311120745235      4.65988110483518
  H   0.58221660372170     -3.26573150035285      3.60834782788884
  H   1.90851700187732     -2.14641329453027     -1.69713491754073
  H   0.37510434360595     -1.38054478475023     -0.97686902705915
```

(*R*,*R*)-(ebthi)ZrH_2

```
47
Coordinates from ORCA-job sp_pw6b95
  H   1.00936295483829     -1.22598045469025      0.96669516635460
  H   -0.61169427776163     -1.78554377155196     -0.42539322495015
  Ti  -0.02911681708767     -0.26751063949468      0.06604823683579
  C   -1.17369489727772     -0.43597488586137      2.10566900653205
  C   -0.40852923193093      0.75110369973540      2.12772537239648
  H   0.43942532375328      0.93906536725767      2.77253912628639
```

C	-0.88932217575156	1.61935493999643	1.12805211336303
C	-0.29535275461156	2.94155443533843	0.74448100939120
C	-1.97210004211603	0.96356097031825	0.48511411487523
C	-2.90348199178824	1.50644866955011	-0.56177285867156
C	-2.14938424976622	-0.30302064758009	1.09799048207531
H	-1.03954076383335	-1.29037228854006	2.75182542564079
C	-3.26178306721592	-1.23145485980151	0.72329639698792
C	0.94269427090739	-0.50460857010373	-2.03325790433847
C	2.01785236419361	-0.34006194864285	-1.13578128898980
C	1.89517130297644	0.94084942037561	-0.54223062356775
C	0.76471238059035	1.58752340019346	-1.09466309177797
C	0.17737293572205	0.68626788742075	-2.01003854456552
H	-0.71412811803838	0.87117427917572	-2.59427158668987
H	0.76578097817340	-1.36269190776548	-2.66353187187330
C	3.17729800285481	-1.25962346441006	-0.88911886998551
C	2.88636126819741	1.46143298864748	0.45382778449885
C	0.20925504324267	2.92843375811125	-0.70858136610364
C	3.77689112914355	0.33567763015613	0.97700957962964
H	2.39148598646464	1.97122643986774	1.28347078292342
H	3.51003090477915	2.21829521446236	-0.03540629387636
C	4.30636602123092	-0.52199674249373	-0.16829669352899
H	3.53901019553170	-1.65062779053973	-1.84329027241422
H	2.85996714890198	-2.12332031359913	-0.30300178330019
H	3.20725360880053	-0.29299253221100	1.66790932479645
H	4.60573397121080	0.76262783890305	1.54485122998465
H	4.83630360556868	0.12153934090394	-0.87825586871471
H	5.03626244919921	-1.24358840141900	0.20306450705601
C	-4.15952683222533	0.64114321601823	-0.68455530706915
H	-2.41485871610545	1.56509904532174	-1.53644251101271
H	-3.17796263897369	2.53380858648944	-0.30694081846797
C	-3.82637818266625	-0.84761411104547	-0.64135429720821
H	-4.05702163770124	-1.15516785326511	1.47277952140341
H	-2.92076230644147	-2.26722761599999	0.73036184303652
H	-4.67942296744689	0.89133747475579	-1.61106929993582
H	-4.85047748671465	0.87422914469425	0.13186582173360
H	-4.71624849397836	-1.44131698487759	-0.85839434087212
H	-3.08982645623392	-1.08317681609118	-1.41764337158787
H	-0.61056952160403	3.17403421416185	-1.38648906525684
H	0.96070861106441	3.71010031761796	-0.84793969901812
H	0.52629695191817	3.16769732681736	1.42672369218692
H	-1.02645348199290	3.74477139369352	0.87483091578854

TS-9

69

Coordinates from ORCA-job sp

C	0.77338791614178	-3.24231585690643	0.71461170578037
H	-0.70842611751877	-2.21330360691011	-0.56148477238146
Ti	0.10192456420612	-1.04289849141539	0.28128606952727
C	-1.36335816424693	-1.33874175685460	2.08646992783842
C	-0.63886783072936	-0.14050785898479	2.28978478936281
H	0.06874914846285	0.05203589286628	3.08188552094298
C	-0.99950827473074	0.76767101095732	1.27583925813005
C	-0.38678669994001	2.11182168792742	1.02590624261000
C	-1.99513437373043	0.14449373811701	0.47448588140061
C	-2.89823199704233	0.77262268107092	-0.54900969680447
C	-2.20196606767501	-1.15994781661379	0.96853906554502
H	-1.33048741865450	-2.21728741588142	2.70985133271922
C	-3.26239932034881	-2.07785699984656	0.44900361308322
C	0.91275883519313	-1.10730148746988	-1.97290949766589
C	2.06032342752296	-0.90098859492074	-1.19470853012472
C	1.91169593919026	0.33464748246418	-0.51066938541920
C	0.68198874691112	0.90641103265126	-0.90156566396631

C	0.06231935544766	0.00139994016790	-1.79426885433392
H	-0.88609300924943	0.13770465501367	-2.28912635258537
H	0.73244654283781	-1.94913299217397	-2.62486643738443
C	3.35466888608486	-1.65252085573326	-1.23574235115296
C	3.03259662425440	0.99922751858911	0.22972424319065
C	0.11382422424341	2.21127771595232	-0.42404862558673
C	4.28787471073547	0.12166689993962	0.27339186370682
H	2.73537106598154	1.27767620623221	1.24432951880553
H	3.26811921086993	1.93884278776341	-0.28303883213425
C	4.50285098462764	-0.66723083894771	-1.01407566750008
H	3.45653790103747	-2.14775095990346	-2.20446886303023
H	3.40923813960135	-2.44012239218820	-0.48163454258135
H	4.20337236803830	-0.59057579191749	1.09275889808343
H	5.15734839122676	0.74735456735615	0.48741144802501
H	4.57203388368650	0.01598850139337	-1.86722856013070
H	5.45240771950843	-1.20539744688267	-0.96597592999282
C	-4.14856431159705	-0.08171890037390	-0.77242921629955
H	-2.40557505119122	0.92925853063726	-1.50885829299883
H	-3.18855853762718	1.77007876839768	-0.20565227478349
C	-3.80323544535576	-1.56413875058822	-0.88036060446876
H	-4.08172120753121	-2.12145361111097	1.17609963755629
H	-2.88139580965224	-3.09628916411890	0.35269531636434
H	-4.66393463078969	0.26080714191367	-1.67217472856921
H	-4.84853650149351	0.06036293834417	0.05791485466032
H	-4.68381435008066	-2.13951917097574	-1.17410145121147
H	-3.04909907706505	-1.71082128357289	-1.66061618050200
H	-0.70927345045565	2.50028139344723	-1.08056110037941
H	0.85881253315554	3.00811886172911	-0.50817659246173
H	0.44683060814588	2.25327190539622	1.71787745043133
H	-1.09893692931465	2.91825119725268	1.23085945215105
C	1.48748326518432	-2.76169890436190	1.80910522770359
O	2.84072703617263	-2.76649060172483	1.88352192915187
H	1.33764213849212	-3.53772768952812	-0.16008234762176
H	-0.09737199054099	-3.84451207993135	0.93003227657779
O	0.96496947413287	-2.84301818294397	3.05588854790662
H	1.33038575619741	-1.05359572889442	1.50588384341185
C	3.20951081483504	-2.51386817844170	3.24811555981530
C	2.02713422010417	-3.15107184008605	3.96271511130015
C	1.72783000202620	-2.65828548474821	5.36001164893987
H	2.15381463908730	-4.24100089476289	3.98208980368506
C	3.40164079750877	-1.02564722854808	3.53327563411695
H	4.13079847854382	-3.06563810741454	3.44077457445654
C	3.22747482928031	-0.69746674639692	5.01265097549055
H	2.68394014961340	-0.44424695146158	2.94616842139923
H	4.39440251083689	-0.72256555095844	3.19696049396142
C	1.86308326323843	-1.15036468215458	5.51185452518685
H	2.44241097211158	-3.14869413406402	6.02782950086539
H	0.73940670965881	-3.01080009502086	5.66309698953604
H	4.01166353030939	-1.18501206467245	5.60239176761949
H	3.35773676072204	0.37651046897790	5.15732303305736
H	1.72526434707146	-0.87794663430218	6.55996842377422
H	1.07433514432279	-0.64022969984954	4.95200097419981

9

69

Coordinates from ORCA-job sp

C	1.36290250933962	-2.22041566545953	0.45604297885364
H	-0.43962165873422	-1.31838131137339	-1.09475382978239
Ti	0.38982023235596	-0.33333961519518	-0.03959555025778
C	-0.72924300114457	-0.69734271146973	1.96840644159918
C	-0.20914504775127	0.61722944417013	1.96793881844670
H	0.56023171255297	0.99960308143198	2.62605796612503

C	-0.89072065554136	1.36935416531737	0.98488349197364
C	-0.61275560865856	2.79247664721363	0.61173338699960
C	-1.80788687663712	0.50385810142974	0.35430282053632
C	-2.78778358813349	0.81816343474488	-0.73589152541329
C	-1.71616208280155	-0.77273748154784	0.96530831423392
H	-0.42436913933501	-1.49569256684798	2.62286719810316
C	-2.64890409711877	-1.89373154736575	0.62944310630274
C	1.75318345147168	-0.19590319798380	-1.99981874982481
C	2.59032692216813	0.17176003452397	-0.93344709852163
C	2.04077703414659	1.33268583330292	-0.33149287731319
C	0.89322439483584	1.71540976767566	-1.07569176465765
C	0.71639840178565	0.75878291741417	-2.09247773545041
H	-0.09131766986366	0.74374843658465	-2.81004441217837
H	1.87196153863407	-1.05972976594433	-2.63763202468311
C	3.89432832842307	-0.43646692900995	-0.51145021791842
C	2.76020920759825	2.08465427896289	0.74696869097952
C	-0.01300319373688	2.87692376135000	-0.79916785343331
C	3.82504990417076	1.20844567566651	1.40165331981102
H	2.07962011435202	2.48312675186755	1.50003285120099
H	3.24620291835920	2.95846067313120	0.29751350315045
C	4.70006466193337	0.53713993530978	0.34809208709810
H	4.46552325659713	-0.72126909418338	-1.39831654661378
H	3.73186315856492	-1.36316200651133	0.04506488162405
H	3.34370038655377	0.44412155871117	2.02221447159555
H	4.43685722247498	1.81516237025433	2.07198217707154
H	5.14712139965161	1.30886120174432	-0.28645550989463
H	5.52762444630045	0.00506454478550	0.82084139097104
C	-3.92421466793602	-0.20294491312823	-0.73977622358673
H	-2.29513256005453	0.81767256263651	-1.71282154940860
H	-3.18475749844422	1.82802673344080	-0.59999771580983
C	-3.38835511485098	-1.63003335319679	-0.67993416225476
H	-3.37901544040798	-1.98747709062782	1.44153667642023
H	-2.11789234521693	-2.84368852348401	0.58863472401822
H	-4.54084133588097	-0.06481107278356	-1.63031601787114
H	-4.57788325073448	-0.02647414120884	0.12113202828802
H	-4.20493084318548	-2.34670229059714	-0.78834536504393
H	-2.70564503131077	-1.79261108809807	-1.51867411072994
H	-0.81386284053179	2.87422667936984	-1.54151455823872
H	0.51837410094676	3.82412265023756	-0.92636761128474
H	0.07241308920364	3.22465630711431	1.34221939318253
H	-1.52620945638100	3.39099876018169	0.66537462808375
C	0.62482125756427	-3.51638844065565	0.66846219276485
O	0.01754314992883	-3.59978817658750	1.93744518546766
H	2.04765506519757	-2.04551717876797	1.30246132967766
H	2.00140768730717	-2.39758021308373	-0.41508289187895
O	1.49345395045575	-4.65544511995795	0.59296150599101
H	-0.12535319686201	-3.65296590868162	-0.12510816172367
C	0.04549276047056	-4.95965921878777	2.32769324388667
C	1.41756475542020	-5.37403765816899	1.80746839217820
C	1.61762999894367	-6.86384934484217	1.59714396615576
H	2.18704239657354	-5.01285219115194	2.50643430554312
C	-1.11896916322427	-5.74861864018247	1.72883360478986
H	0.00226982555971	-5.00170332118833	3.41977226677721
C	-0.84981337424574	-7.24900115962280	1.73184955672517
H	-1.29205885722931	-5.43142741926118	0.69526532531703
H	-2.03178661244001	-5.51149194951270	2.28055492170755
C	0.42290100253104	-7.55413280327408	0.95158296593854
H	1.79409768031972	-7.30872060699660	2.58189841442159
H	2.53248472051618	-7.03121186745448	1.02302674501733
H	-0.74494156870335	-7.61210267795977	2.76041404759935
H	-1.70337460502307	-7.77853867146943	1.30339149649838
H	0.60153264692196	-8.63096613597759	0.91340130951016

H 0.30546929198877 -7.21945373897102 -0.08340085886249

TS-11a
69
Coordinates from ORCA-job sp

C	1.48537315026838	-2.03785742066038	2.04902768872625
H	-0.92418633339201	-1.31979940816670	-0.98481929175857
Ti	0.14101302190364	-0.90120924533012	0.16678789189509
C	-1.21277174866403	-0.77037797568905	2.13131779972438
C	-0.44152184819370	0.41375052464638	1.98573979157908
H	0.34347836542880	0.75163491060220	2.64663449293028
C	-0.95551715132937	1.12461889467631	0.87996721383028
C	-0.47756749537097	2.43700154850245	0.34577997467217
C	-1.97251560551780	0.34465272652048	0.29861496467015
C	-2.86109757665679	0.67484735639081	-0.86392393809637
C	-2.15257803753573	-0.81473608650093	1.10022335879912
H	-1.11922770375004	-1.49993364805189	2.92015484541497
C	-3.30006298898513	-1.75386133020028	0.90568316983981
C	1.64321179089686	-1.11143883319317	-1.72776107202408
C	2.47268215109649	-0.63306720178809	-0.70584171397751
C	1.96762516376075	0.61797530211559	-0.27778345567362
C	0.85938904300549	0.94099209937942	-1.09927116938563
C	0.64314316574427	-0.14353831911771	-1.97835641132973
H	-0.09099503036258	-0.17566566599655	-2.76932745836662
H	1.73992779316919	-2.06254944990649	-2.22787290550888
C	3.80028822794355	-1.16403830600850	-0.25826964132220
C	2.72341471741609	1.52800541070983	0.64464292500288
C	0.09019233350597	2.22330231832943	-1.05811571256116
C	3.99005340961194	0.87260334761590	1.20166294076981
H	2.11125322586460	1.88198317840704	1.47601678450000
H	3.00264065422941	2.42609234637815	0.08169626428508
C	4.69647971047535	-0.00286157162116	0.17297246753504
H	4.26160923728767	-1.72282895436763	-1.07558355853655
H	3.71481806247987	-1.86477673250279	0.57398246079430
H	3.73689069709532	0.26355178704693	2.07395241560516
H	4.66199691403209	1.65233478940696	1.56583044008358
H	4.96879376302632	0.59646795979141	-0.70179876398193
H	5.62874734076730	-0.39079817323921	0.58863135822047
C	-4.17288295172450	-0.10740596780388	-0.78049063250455
H	-2.36476533745233	0.43740139088782	-1.80887723972110
H	-3.06559960895044	1.74908005802278	-0.88788956618696
C	-3.93149958785244	-1.58270522820473	-0.47243271209199
H	-4.05284056954813	-1.52483504817644	1.66882205256446
H	-3.01262397497337	-2.79134866186582	1.08436639568860
H	-4.72166003357895	-0.00126578575110	-1.71845142915316
H	-4.80851667050075	0.32253854625306	0.00049648212681
H	-4.87144096779470	-2.13672622439211	-0.51346469969736
H	-3.27784163913425	-2.01395174054237	-1.23676425716958
H	-0.72207064011476	2.17417054665321	-1.78554517215628
H	0.72027097060455	3.06648214441483	-1.35760263948975
H	0.28320887696210	2.85125877307029	1.00806053381051
H	-1.29540239629238	3.16302122552393	0.32588583658100
C	1.54311034797052	-3.22823967874291	1.34083132958600
O	0.28378385330278	-2.97978994311701	-0.13326634662990
H	0.89309743009812	-2.01033029605222	2.95241528793909
H	2.37443667057301	-1.42350940153639	2.06498885652736
O	0.93698852748589	-4.37487234526780	1.73807311570558
H	2.40220122240518	-3.42198031936518	0.70283037116891
C	-0.62245543731380	-3.99488117195886	0.06910044056337
C	-0.47684130707595	-4.37138136084513	1.55152927924542
C	-1.06120796633674	-5.71178584391378	1.94715341963908
H	-0.92801154035699	-3.58873661536994	2.17737926790430

C	-0.31761207118914	-5.17919558844457	-0.85218771453378
H	-1.65893010958647	-3.67183930707778	-0.11541727487353
C	-1.06009123177442	-6.44700125887527	-0.44518729503627
H	0.75882591126894	-5.37587696064071	-0.83418785776921
H	-0.56516937060137	-4.89760250101251	-1.87935591284861
C	-0.70419056932809	-6.83714301413579	0.98402845244881
H	-2.14908589637836	-5.58892848697506	1.96142484901194
H	-0.77107162253828	-5.95442430892612	2.97278117868582
H	-2.14229009320594	-6.28953886536553	-0.52181685048859
H	-0.81971216762126	-7.25970588853279	-1.13477349704661
H	-1.23108029678424	-7.74733932738464	1.27967724555710
H	0.36418982808583	-7.06312772272786	1.04652254628796

TS-11b

69

Coordinates from ORCA-job sp

C	1.74591627594616	-2.04776057530108	1.91918319054181
H	-0.64047802338729	-1.21773557360548	-1.04233993816077
Ti	0.33088481207369	-0.85786266375392	0.19595189731252
C	-0.95104143570347	-0.74518470054411	2.20776321951791
C	-0.32815024142577	0.50076966479411	1.95857705263018
H	0.42073721543743	0.97511475926110	2.57661501884650
C	-0.92296167537455	1.06024652267493	0.80640937088308
C	-0.59182823432524	2.38523678650501	0.19521654589457
C	-1.86618523478022	0.13118347880948	0.31333266095012
C	-2.83123064325870	0.29507769085315	-0.82536263615354
C	-1.89185866682507	-0.97647976250479	1.19885418034300
H	-0.73821868542160	-1.40906879040505	3.03222991534289
C	-2.81774826276452	-2.13350239488566	1.01946145789234
C	1.92016092780634	-1.05189383272146	-1.62734558849678
C	2.70747515341529	-0.45061653708042	-0.63188781461055
C	2.09023158416250	0.75837863614429	-0.25053438285861
C	0.96641225108800	0.95728631078721	-1.10479825180012
C	0.86609892934169	-0.16507396795955	-1.95458830925954
H	0.18265736989090	-0.26290278493445	-2.78465944790154
H	2.13522792888426	-1.98825328351817	-2.12118755466142
C	4.08488832078329	-0.85808840550869	-0.20592144032231
C	2.74397166520268	1.72174391524949	0.69358304960786
C	0.08029488002728	2.16467878010430	-1.15913729595377
C	3.89591521304240	1.05253867787087	1.44046582278940
H	2.04170208138930	2.14592574438854	1.41298416633346
H	3.13161576565563	2.57376958602414	0.12209885749573
C	4.81163534182748	0.29827418631969	0.48340752167613
H	4.64080894877543	-1.16910810490141	-1.09434753088115
H	4.08297036414730	-1.73376953116536	0.44570691742373
H	3.49300114142556	0.36027709305743	2.18559674022637
H	4.46317174594990	1.80772041829158	1.98836902308540
H	5.18876886605941	0.99507839085797	-0.27181593314125
H	5.68668358322442	-0.08352684091150	1.01314068470205
C	-3.94171968860361	-0.75586731624939	-0.75885286156604
H	-2.31184964145923	0.22446686917292	-1.78425774152396
H	-3.26816994041078	1.29811287400406	-0.79560626565522
C	-3.40617348509351	-2.13669852557066	-0.38878898785850
H	-3.63026517766740	-2.05474537897803	1.75111667206905
H	-2.30047743338475	-3.07203984118134	1.22160125601672
H	-4.46486553628127	-0.79109964437406	-1.71682592120089
H	-4.68588878226895	-0.45470266928308	-0.01396268166053
H	-4.20482462637302	-2.87888206477818	-0.45484801658469
H	-2.62768439401465	-2.43779740394238	-1.09524778339046
H	-0.67796887048913	2.00196535369720	-1.92742013253802
H	0.64112983313143	3.05206494373733	-1.46869696426991
H	0.07172594931566	2.93334571894000	0.86497506046551

H	-1.48920624255923	3.00163019299421	0.08983359127206
C	1.02580807220079	-3.22811704197048	1.80678803494236
O	1.51892918411345	-4.38746644011529	1.32123295799383
H	1.55247098555920	-1.43837815292772	2.79196044856877
H	2.76703047159134	-2.04304497773740	1.57525986936216
O	-0.03354010123578	-2.92913811744600	0.15636619396497
C	1.79317782843140	-4.34294391895117	-0.07961864577411
C	0.43733823850674	-3.91870727886521	-0.67210023585762
C	-0.55250611398139	-5.08511743290183	-0.72464392199101
H	0.61112640570378	-3.56405199916128	-1.70285037226142
C	2.32632189724023	-5.67618297664730	-0.56189883280560
H	2.54594781009651	-3.56726013460088	-0.27449363145753
C	1.27939786076332	-6.78348618794167	-0.58032236574947
H	3.20111914388755	-5.96311088835963	0.02761872982367
H	2.69185790155276	-5.51570203597378	-1.58154605480798
C	0.04330934456094	-6.33783939490178	-1.35302235613689
H	-1.44330203418938	-4.76074333346704	-1.26965493523945
H	-0.88277782776041	-5.31655424384534	0.29288645989055
H	1.70917094474777	-7.68362569472082	-1.02565146654867
H	0.99286876488233	-7.04927909467434	0.44120588251622
H	0.31518356315003	-6.14311692203505	-2.39683101384165
H	-0.70118008061048	-7.13717136280483	-1.37494037356529
H	0.17905651465767	-3.39082837043184	2.47065523610529

TS-11c

69

Coordinates from ORCA-job sp

C	0.68776548763664	-2.55675461943897	0.03768093529318
H	-0.24199469017213	-0.72976346600017	-0.81563463209873
Ti	1.03325937366333	-0.15625610712986	-0.02357649504133
C	0.42288469274620	0.40036220471426	2.26010084147445
C	0.80601319561793	1.55274489862597	1.54952007075136
H	1.65728557099227	2.18101498549458	1.77080968007770
C	-0.17331762783429	1.79422802465221	0.55010677177174
C	-0.22222676275748	2.94432050995829	-0.40698816697858
C	-1.11588377074850	0.74949251360508	0.61444760402583
C	-2.42642071394639	0.63604290734841	-0.10949477217675
C	-0.74191758522336	-0.11472182143632	1.68019345812385
H	0.96100837112506	-0.03988442667943	3.08390631951527
C	-1.61249697813597	-1.23825800947891	2.15070735676969
C	2.11392262789218	-0.66244982221429	-2.09805121003460
C	3.04184354669438	0.20862232261350	-1.49779772398452
C	2.40606070811345	1.43541059917827	-1.25902887449669
C	1.08614884469721	1.35635191247089	-1.79402071907038
C	0.91778695994794	0.06113724610260	-2.32964512424400
H	0.06386907752778	-0.27847299146701	-2.89633783576880
H	2.31099267275754	-1.68182496686479	-2.39519826854308
C	4.48414308822523	-0.04916310323021	-1.20967001970015
C	3.14118528221285	2.61982003319922	-0.71050749373064
C	0.05987643190141	2.44648541280984	-1.82585544578513
C	4.42308967915493	2.17553436984439	-0.01183303798406
H	2.52340923002091	3.20449426165896	-0.02765019893834
H	3.39258515564051	3.29934928575953	-1.53396552838009
C	5.23623045664563	1.24835790514394	-0.90775049302783
H	4.94015590872727	-0.55194799531185	-2.06700405593674
H	4.58500234499907	-0.73677811957315	-0.37240668146557
H	4.16508405996770	1.65570288409903	0.91645647989652
H	5.01379497752713	3.05111359050871	0.26612465645180
H	5.46585216729417	1.76930612410625	-1.84298931046241
H	6.19607687776868	1.01307283239530	-0.44312160405225
C	-3.40285347510178	-0.21575417539952	0.70061109755445
H	-2.29050525827595	0.19523917211448	-1.10020038362662

```
  H   -2.84658896466512      1.63277492441689     -0.27515757717197
  C   -2.74964602754739     -1.51301633154428      1.16693039134316
  H   -2.04502770327609     -0.95654256659252      3.11708339735112
  H   -1.03759609697528     -2.14801404494653      2.34244243563058
  H   -4.29041576981018     -0.43127061438837      0.10239390725007
  H   -3.74458300193971      0.34930752121560      1.57409806667467
  H   -3.48931823065497     -2.16187855316536      1.64021942910883
  H   -2.37372411914470     -2.05547505787085      0.29328446706416
  H   -0.85796755765840      2.04680903193983     -2.26350412807915
  H   0.37440278935983      3.27095068964046     -2.47348797563064
  H   0.50836609731856      3.69779015455861     -0.11169171075564
  H   -1.19736178029878      3.43874894321399     -0.36288469301569
  C   1.63525750613266     -2.65296930253358      1.04408631895829
  O   2.60460697698608     -0.93949385155988      1.11727446687066
  H   0.93515673526125     -2.94472965110093     -0.94348270532454
  H   -0.34533330365479     -2.67097873725066      0.33360161325720
  O   2.77144203615138     -3.34702183473375      0.86104712677619
  H   1.28326455321478     -2.55422328395174      2.07155102275519
  C   3.63755684849416     -1.37509977901008      1.90372169779159
  C   3.98320817511992     -2.78337814265563      1.37556433324594
  C   4.69875025362437     -3.70896169716185      2.34675426883519
  H   4.62558130489152     -2.67125683237202      0.49567487675195
  C   3.31067701920408     -1.32916735595489      3.40599835466995
  H   4.54499315274040     -0.76114126796291      1.76608987137137
  C   4.25649781112168     -2.17111648992757      4.25153811914328
  H   2.29565372254687     -1.69447316112155      3.58622566572339
  H   3.31844912163727     -0.28421209650547      3.72805300370279
  C   4.20793044429031     -3.62002864195612      3.78405653799198
  H   5.75479561921505     -3.41994217701956      2.33109501463596
  H   4.66462925193011     -4.73412852433785      1.96931675068891
  H   5.28318676110741     -1.79605210580769      4.17120173293811
  H   3.98070505525781     -2.09186125579503      5.30551211430476
  H   4.82649280056273     -4.25697767120155      4.42006853713919
  H   3.18355859215456     -3.99749460873618      3.86902807182404
```

TS-11d

```
69
Coordinates from ORCA-job sp
  C   0.71230913780628     -2.61950357564159      0.30498887990758
  H   -0.37678754288265     -0.93040973855104     -0.68403042412174
  Ti  0.79619306019335     -0.24736262834624      0.19276950396919
  C   -0.16765625345214      0.13333900977285      2.36727953989780
  C   0.37307105558943      1.33414496759298      1.85514319353950
  H   1.20646428840537      1.88416761388539      2.26799838582589
  C   -0.41687259250415      1.72220898443597      0.74717348736369
  C   -0.24661076413503      2.94510290849140     -0.09868854503886
  C   -1.40231860941754      0.73268156007301      0.55254535417773
  C   -2.53679949915139      0.71947093341130     -0.42766456462854
  C   -1.25218606984874     -0.24335333761269      1.57003667082900
  H   0.17886245809115     -0.40381006402125      3.23674976327962
  C   -2.22203296390423     -1.36328922595247      1.76725980911175
  C   1.95732334003005     -0.71102284635209     -1.87615084416025
  C   2.90355819308738      0.05207668751460     -1.17210459975452
  C   2.32961121190055      1.30144014973376     -0.87675769204425
  C   1.04745649619709      1.35119569269363     -1.49424364141360
  C   0.82002765476227      0.10404245863836     -2.11018150717514
  H   -0.02718381311723     -0.14661668743921     -2.73048727335595
  H   2.09863169708085     -1.71687783507887     -2.24607389381620
  C   4.30652887236652     -0.31482099174348     -0.81947961612379
  C   3.10747743841874      2.41425894319489     -0.24091540294159
  C   0.12474433338189      2.53160274022665     -1.52428602558123
  C   4.40217037444510      1.89836885199076      0.38267306423199
```

```
H   2.52471997094761     2.94274006434631     0.51595547430419
H   3.34020359362179     3.16287995499533    -1.00796325589411
C   5.13734156303788     0.93995559814684    -0.54760528327083
H   4.75191934783009    -0.89291794924845    -1.63383803187005
H   4.31556902654139    -0.95361837581928     0.06580684352224
H   4.16581145920082     1.37763324637296     1.31354979759643
H   5.04267380795160     2.74409969700103     0.64299885272074
H   5.36386092106448     1.44840695004323    -1.49090101915169
H   6.09759925021892     0.65532868302521    -0.11182614343874
C  -3.65158461184529    -0.21109577158083     0.05258741100075
H  -2.19062529309548     0.39048506338915    -1.41010867589242
H  -2.92881244104993     1.73214640777148    -0.56228305522997
C  -3.10238826359720    -1.55115355850196     0.53492891617204
H  -2.86059891557823    -1.12310251393987     2.62500184430210
H  -1.71540423797217    -2.29389583930069     2.03292026498477
H  -4.37341299670361    -0.36208870186690    -0.75276545176111
H  -4.19947904064747     0.26570909766821     0.87249030216125
H  -3.92186347111964    -2.23321987620541     0.77093086087218
H  -2.52022395817158    -2.01822776616202    -0.26405918413135
H  -0.77562126579631     2.25749258976729    -2.07699055292518
H   0.57512401104197     3.36837389932034    -2.06761796130111
H   0.52772928043246     3.57944143587270     0.33346336855500
H  -1.16130022997481     3.54548740898325    -0.09651056672945
C   2.03882524566812    -2.76796398350342     0.71661390238105
O   2.45729495158251    -3.29911261716662     1.87330299497509
H   0.52384858805611    -2.92767086641179    -0.71578989692827
H  -0.06843178277330    -2.88140966833509     1.01067866289112
O   2.40532505191611    -0.90267832385592     1.39428566355446
H   2.80443666419731    -2.84221249313520    -0.05451267750678
C   2.06149468921933    -2.54837542661377     3.01949723664851
C   2.67124174173583    -1.16704571083977     2.71575837137691
C   4.18160949010718    -1.15772370335640     2.97425289431948
H   2.20899286298648    -0.43300965636448     3.39625123538878
C   2.54804249507050    -3.21736683524646     4.28846772896538
H   0.96573945218644    -2.47877088293966     3.05071394342737
C   4.05890553063732    -3.15192337371385     4.47717562462796
H   2.18477572824239    -4.24758826506143     4.32760789818976
H   2.06479981871789    -2.69480744031748     5.11988712172399
C   4.54935677529156    -1.71588875110717     4.34283287575390
H   4.54568960268527    -0.13380690472356     2.85926591615626
H   4.68174532416610    -1.75073811433087     2.20197328169513
H   4.31807709089859    -3.55907243215749     5.45718080291691
H   4.56229713011974    -3.78187514438338     3.73789205622232
H   4.10247975764346    -1.10214843976321     5.13355103354001
H   5.63092478196445    -1.66560528166740     4.48901495310667
```

11

```
69
Coordinates from ORCA-job sp
C  -1.97040351224479     2.93380358400918    -3.21211258839732
H   1.15412467245802     0.34438828046993    -1.23232376639984
Ti  0.73528555761301    -0.61225382650476     0.05088831435310
C   1.44421265713290     0.50023170968049     2.03952267898593
C   1.81634963820004    -0.86376034373518     2.07474366228272
H   1.46490412966968    -1.59348958016035     2.79002626583755
C   2.81615483126037    -1.07047692749056     1.09121608119656
C   3.46756155739475    -2.38495060135116     0.79363137998560
C   2.98810207394373     0.14288252272371     0.40123904708951
C   3.96736951693192     0.47345402168618    -0.68176718323897
C   2.14506886945389     1.12123698852076     1.00115226530952
H   0.71443794779906     0.96818764027840     2.68522842006268
C   2.11799525957412     2.55080515984451     0.55178205343823
```

C	0.22516072677265	-1.81600973506017	-1.94216238177858
C	-0.75073911820170	-2.21302347496024	-1.01591519174188
C	-0.08933729828295	-2.83942793148225	0.06860930451966
C	1.29329550922025	-2.91429507408541	-0.23871777312570
C	1.48580770462041	-2.26051108135737	-1.46654951097354
H	2.43440608164530	-2.10408855265094	-1.96049273373608
H	0.04623094718593	-1.29837202078152	-2.87279677579269
C	-2.23403181524767	-2.04536544261154	-1.08305684859419
C	-0.82259833279810	-3.42674646834897	1.23570003585462
C	2.38440231736252	-3.45553398529426	0.63150855183317
C	-2.25780188745733	-2.90508005435151	1.29119013031702
H	-0.30829722352271	-3.21495645024670	2.17652777131040
H	-0.83575891903402	-4.51814479628191	1.13803052992387
C	-2.92615109332485	-2.96907480192383	-0.07948595320905
H	-2.58489156640733	-2.25935781604211	-2.09601935388228
H	-2.49991099569899	-1.00752326353452	-0.87327541481152
H	-2.25472555097311	-1.86913097981200	1.64003228329180
H	-2.82773775282280	-3.48572547331486	2.01963369690099
H	-2.90184567146625	-4.00164584668801	-0.44358494563130
H	-3.97975039282649	-2.69369151394117	0.00405542941182
C	3.67465000818522	1.83941422844511	-1.30034388676077
H	3.98187170511176	-0.29605158908032	-1.45754334417714
H	4.97348561205854	0.48611711538358	-0.24772227639051
C	3.38936540931903	2.88671291584495	-0.22771532864473
H	2.01211506108700	3.21670871556689	1.41144020308982
H	1.25240204496418	2.73201153384754	-0.09157248814426
H	2.80950688754442	1.75857856334426	-1.96426468067826
H	4.52187498561310	2.14863215445625	-1.91560808518342
H	4.23991593507521	2.94373672454593	0.46006681531880
H	3.28975019915065	3.87523924104057	-0.68084039117524
H	2.80562100241994	-4.37317341801024	0.21233314226370
H	1.97937809809076	-3.72562225453347	1.60961739615553
H	4.17144478763521	-2.66202108213127	1.58302093629749
H	4.05526464978336	-2.30583819107063	-0.12368185476575
C	-2.63677755834960	1.97699098933434	-2.56737610822431
O	-2.59084685297118	1.63375568869633	-1.27460165406953
O	-0.82819112064225	0.24366111782940	0.37218739393447
C	-1.70965973553496	2.32961453355270	-0.40543589441396
C	-1.42123361501771	1.41975312350567	0.78951275785653
C	-2.68005145767762	1.16512399942784	1.61600193297504
H	-0.73139427474808	1.99766270468983	1.42931807651161
C	-2.32759540110742	3.64343584465561	0.06516122368601
H	-0.76039544479257	2.51253073159857	-0.92473787726727
C	-3.58569878770898	3.40840655421594	0.89548256586283
H	-2.54021387211383	4.29581082633903	-0.78463889437499
H	-1.58179474959844	4.16603372617602	0.67293230703859
C	-3.30243809275944	2.48260895693591	2.07428675746131
H	-2.41479278620370	0.55084047594900	2.48081362545388
H	-3.40066494251595	0.58823645352958	1.02903204681877
H	-3.97386536621348	4.36526601282555	1.25146542206601
H	-4.36789072192082	2.97162863799360	0.26841502348290
H	-2.61866626363077	2.98369828865971	2.76849814615955
H	-4.22059957445008	2.28639892933725	2.63256714972849
H	-2.13555831598831	3.04576170298357	-4.27334997555428
H	-1.27028743582533	3.61447169221766	-2.74851167815981
H	-3.33274331019726	1.33923706579586	-3.10231596176895

TS-10

69

Coordinates from ORCA-job sp

C	0.67381171001942	-3.23203242870426	0.75397363555371
H	-0.70582973492163	-2.15265714010367	-0.52480891843698

Ti	0.14179768922916	-1.00191816635926	0.29781471138731
C	-1.27911122632562	-1.23010855271090	2.14644553410631
C	-0.56573044640174	-0.01322904102713	2.27446051213124
H	0.16154637989247	0.22173341459766	3.03691135222934
C	-0.97344367856332	0.84195048878783	1.23175966764354
C	-0.41231351213695	2.19168164798978	0.90870688921524
C	-1.96835863710829	0.16249188281867	0.47993413615073
C	-2.88397107404882	0.71076446884380	-0.57711934556056
C	-2.14377795891373	-1.11937367497555	1.04074201798398
H	-1.21371622067678	-2.07910496597310	2.80685989133214
C	-3.19257725709084	-2.08274291353700	0.58337383507960
C	1.00789575029173	-1.12323439519235	-1.93949289586036
C	2.13181441974029	-0.84675619257166	-1.15014630744509
C	1.91983269974902	0.40432987537850	-0.51071057651119
C	0.68600317910904	0.92212394818133	-0.95691737704954
C	0.11406085782213	-0.03771829466323	-1.82142453552873
H	-0.82347781028349	0.05064248771358	-2.34817211101876
H	0.86820438039621	-1.99586648954107	-2.56067605659250
C	3.45407784977441	-1.55115832938978	-1.13416766607023
C	2.98491425061404	1.12456586706445	0.25768493875368
C	0.07927099798859	2.23148451890786	-0.54643535390077
C	4.23690008658499	0.26374596477334	0.43131519551019
H	2.62262156926234	1.45456867858692	1.23369515802868
H	3.24911200116856	2.03639442322097	-0.29060715418537
C	4.56185572385480	-0.54300260141963	-0.82191247119544
H	3.63014445759048	-2.00967211184886	-2.11037827524499
H	3.48748506872212	-2.35927959914078	-0.40353506925625
H	4.08889936533118	-0.42778252441741	1.26170711309425
H	5.08069593726474	0.90181014618179	0.70254605419869
H	4.68763332817184	0.13800618485494	-1.67040112274370
H	5.51293015032064	-1.06528888260614	-0.69573158368726
C	-4.12510666331896	-0.17038459782991	-0.73727166522855
H	-2.39234937107476	0.80149843665827	-1.54603846525034
H	-3.18506647559311	1.72662696612048	-0.30407951973168
C	-3.76126206704119	-1.65193073194435	-0.76413962117364
H	-4.00112940709279	-2.10549660914907	1.32321609627636
H	-2.79233903017155	-3.09748135687073	0.53365319287610
H	-4.65390626067701	0.11299844317002	-1.64964535943284
H	-4.81829107619427	0.01066288908525	0.09120132964222
H	-4.63768900404754	-2.25479232548573	-1.01143177121000
H	-3.01639695373097	-1.83224285482601	-1.54569958662835
H	-0.75156969848472	2.46323173406249	-1.21556029664758
H	0.80207281619080	3.04271969801639	-0.67470840513803
H	0.41427748291050	2.40693200480666	1.58995362589098
H	-1.15661647713158	2.97806506179011	1.07363751177814
C	1.47903630997410	-2.76573159521238	1.78899303806494
O	1.05411632080266	-2.77924592576597	3.07755835500810
O	2.82521458782583	-2.83947882974197	1.75946928841389
H	1.40773693478010	-1.05194604175522	1.49574625461105
C	2.20045471914067	-2.81114742290137	3.93567630595234
C	3.28310909057742	-2.29514590998797	2.99787242488705
C	4.70498495075956	-2.70861291763776	3.30254078890212
H	3.21456839071130	-1.20150206918837	2.93611129459406
C	2.43281080294015	-4.23142759187742	4.43972469699372
H	2.03371089425687	-2.12252631296311	4.76622909343328
C	3.87007608789323	-4.43587574041295	4.90566203707134
H	2.22539138500341	-4.94224689120772	3.63405555628668
H	1.72427763637782	-4.45289171937459	5.24047870471260
C	4.83794433776109	-4.15457740147399	3.76281015750901
H	5.06125752759277	-2.04877312258954	4.09877103734571
H	5.33533969728246	-2.50257242336842	2.43415206525967
H	4.09780243994873	-3.77691000689929	5.75068466029958

H	3.99182427421654	-5.45749051826013	5.26990967745854
H	5.86743255266288	-4.33750130630037	4.07687309796465
H	4.63869089145542	-4.84217024073176	2.93575415755800
H	-0.22219402899811	-3.77048238457444	1.02844893657024
H	1.18370608606477	-3.59287807909829	-0.13016251703063

10

69

Coordinates from ORCA-job sp

C	1.45452461928085	-2.17403065937013	0.12344330275116
H	-0.48545673510887	-1.07963968613214	-1.30089315680426
Ti	0.41277073028145	-0.27609750528471	-0.14603243026142
C	-0.62785513497717	-0.86625443548003	1.85516213356603
C	-0.14244495794456	0.45837107515422	1.95827603026656
H	0.63726254529687	0.80063047292180	2.62601802550121
C	-0.88839761834927	1.27783066993163	1.07899032909016
C	-0.68246052641081	2.73922605293549	0.82604070648237
C	-1.80489066151566	0.44699749524197	0.40635629396069
C	-2.83799452130524	0.83183040511164	-0.60912113278079
C	-1.65088800976880	-0.87773229013849	0.88761278911770
H	-0.27817538893447	-1.71454674832853	2.41919514821068
C	-2.57463541132021	-1.98651272920331	0.48999153831252
C	1.75967499709908	0.10391754998802	-2.08208613655419
C	2.58461991147180	0.40838499491057	-0.98709725451459
C	1.98473119796283	1.47713151070527	-0.27498765036380
C	0.81688671233374	1.87515245053169	-0.98015895313236
C	0.67953770751324	1.01406878768075	-2.08511199168875
H	-0.12621936757598	1.03590683170916	-2.80421780097098
H	1.91986158782839	-0.68651378851161	-2.80140065931510
C	3.92019162785543	-0.17154359484963	-0.63153423977933
C	2.66872291652208	2.15190996730192	0.87585981892986
C	-0.13498799629734	2.96586815135655	-0.59014793838900
C	3.79089524833052	1.27741674888570	1.42973742800832
H	1.97173025080918	2.41547630509188	1.67130833543401
H	3.09391036200476	3.09954075715356	0.52535052193358
C	4.68491618284849	0.76190296271899	0.30694681671175
H	4.49481161004315	-0.34070016268272	-1.54498419217762
H	3.81168649872915	-1.15362691205320	-0.16366409992072
H	3.36348403740313	0.42978783071866	1.97748329040409
H	4.37811972049633	1.85055118073580	2.14988119420622
H	5.07226556615373	1.61586693619474	-0.25753681817836
H	5.55123506105395	0.23827589596458	0.71518885749324
C	-3.95458264692225	-0.21019335390044	-0.64966733826740
H	-2.38940102785868	0.92065660675734	-1.60276927869548
H	-3.24497814816007	1.81867892812501	-0.37208014907700
C	-3.38727485284791	-1.62326185078642	-0.75081368188145
H	-3.26421035607383	-2.16523468686851	1.32291366893393
H	-2.03131881627602	-2.91829855937163	0.34190752306203
H	-4.62029163653815	-0.00584160454919	-1.49074065271815
H	-4.56350070848102	-0.13006175346617	0.25697361334020
H	-4.19468563400052	-2.34702651604528	-0.87979929105098
H	-2.75232980399428	-1.69248657018417	-1.63862734053628
H	-0.95828316405491	2.97941897411509	-1.30719818061725
H	0.34589538796682	3.94573516210391	-0.65833371244720
H	0.00460821182367	3.14140047436344	1.57127140768591
H	-1.61990148495789	3.28646630570240	0.95716291664865
C	0.75814559166831	-3.50353541565939	0.21405173829979
O	1.67933309438753	-4.60210065379197	0.05347369667397
H	2.12768753547579	-2.06249429521924	0.98917147605645
H	2.10559072829343	-2.25237553706387	-0.75340805622623
O	0.14152245995367	-3.72818981927984	1.45012559504485
H	0.01618345597994	-3.60051331705355	-0.59598080001005

C	1.45969924571159	-5.53194632101439	1.09670835665760
C	0.07131586285850	-5.13123164186582	1.59068676376027
C	-0.28925261821484	-5.54477255623346	3.00315399803082
H	-0.68829963397265	-5.53442911717203	0.90121292870142
C	2.54340282878220	-5.41710643819803	2.16820701236830
H	1.44882538684229	-6.54849405217922	0.68720699774627
C	2.11390883072032	-6.04913430824175	3.48699458879967
H	2.76467028775882	-4.36042652366934	2.34333074017241
H	3.46724423222949	-5.87093214084688	1.80046209194413
C	0.84759090618977	-5.37636613152310	4.00290376213448
H	-0.57423158643000	-6.60046351767598	2.96581121962569
H	-1.18351495455960	-5.00141447201012	3.31888046145769
H	1.93250448074435	-7.12178365022726	3.35364682174943
H	2.91989488823657	-5.96303169930943	4.21879632085942
H	0.55025794163485	-5.79916905254612	4.96506344339040
H	1.04496295427485	-4.31444741612453	4.17597323283469

TS-12a

69

Coordinates from ORCA-job sp

C	2.66011613485355	-1.58699012101964	1.85071740345313
H	-0.57886233022099	-1.38136590560110	-0.53354805178676
Ti	0.65846147533733	-0.79201899641110	0.34246822713576
C	-0.15019953871728	-0.73766220515945	2.58309051769331
C	0.34879395877082	0.53790066251992	2.20369217458622
H	1.20865298517454	1.03444560101727	2.62844476813679
C	-0.54572797182438	1.08342186452486	1.25534588181908
C	-0.47344271521964	2.41268087474857	0.57511303393007
C	-1.53242941003466	0.11691573276137	0.99010228963119
C	-2.73239895977599	0.23296453799974	0.09691401423722
C	-1.30763283326189	-0.99271896296912	1.84691483362325
H	0.27791653713152	-1.39450993121931	3.32351190425766
C	-2.31146269317301	-2.08776424381951	2.00226159978302
C	1.75069449878877	-0.85109214597137	-1.83015155355911
C	2.68126085067746	-0.14882180769974	-1.05061677314332
C	2.04699203379624	1.01227625944266	-0.55120713188244
C	0.74341026572291	1.06568387764047	-1.10145301561601
C	0.55125042743733	-0.10122278111621	-1.87534043369580
H	-0.31338613480262	-0.32011208108168	-2.48279037252047
H	1.92324045435184	-1.79839991847712	-2.31937832202345
C	4.16025882231642	-0.37244192408588	-0.93366409789491
C	2.79614897377601	2.11232840505186	0.14325787536635
C	-0.22120334873323	2.19323468925510	-0.91821066443840
C	4.25307118922717	1.73624535238217	0.41884690614877
H	2.32326077660928	2.41286808554092	1.07868282457881
H	2.76957576533411	2.99940283032724	-0.50050078593258
C	4.87211421733682	0.96595786501991	-0.74044933576549
H	4.52076263223144	-0.87190174215314	-1.83586467887916
H	4.43266278232180	-1.02459902438564	-0.10068267435647
H	4.30962842749262	1.12528977247986	1.32483420818048
H	4.82387177304028	2.64321065254717	0.62709326788958
H	4.80593071863138	1.56164399864505	-1.65669745498254
H	5.93403944628321	0.79192944879429	-0.55539515008345
C	-3.82847324509447	-0.74688477116639	0.51922509067174
H	-2.45410627140411	0.03436848950289	-0.94163744115196
H	-3.11865853334041	1.25603202525951	0.11954129431676
C	-3.26603127661053	-2.13486594883115	0.81389792857682
H	-2.89229175829497	-1.88039218516198	2.90868267040264
H	-1.83607241076696	-3.05105258279046	2.17058835405672
H	-4.58840197395296	-0.80128630318208	-0.26289578657998
H	-4.33214672656667	-0.36958128806999	1.41514285922607
H	-4.07722231955633	-2.83582514129755	1.02172623881333

H	-2.73753519095750	-2.51113239000980	-0.06717447977994
H	-1.15680470077703	1.94859439943538	-1.42372824054226
H	0.15435630264173	3.10777516306728	-1.38862840209382
H	0.31735102371508	3.01714960553074	1.01830012432580
H	-1.40077559303144	2.97191753120236	0.72907277788824
C	2.71481071254669	-2.68548918121550	1.00961571435565
O	2.65782611741516	-3.96557582760465	1.43623330074137
O	1.01902062801607	-2.81390388503820	0.20494298054960
C	2.18075268293804	-4.78036420951643	0.37275083095772
C	0.79809012074499	-4.16214189776083	0.04743543484458
C	-0.28746521791543	-4.69320389047192	0.98108369517560
H	0.53531127583484	-4.43226233340926	-0.98994001104184
C	2.19444172457171	-6.24879138313235	0.74409850767787
H	2.84277512360645	-4.63708248139569	-0.49501707684874
C	1.07693724240259	-6.66070757034038	1.69196387249765
H	3.17746403468669	-6.51390788946242	1.14194325615208
H	2.08760607336015	-6.81147774816207	-0.18846437032091
C	-0.26765167529189	-6.20810042554938	1.14019088582831
H	-1.26126145202371	-4.35754020568940	0.61791249622146
H	-0.14276889642469	-4.23889344537740	1.96554556186392
H	1.09702736686548	-7.74424527031309	1.82843205810312
H	1.23529535899207	-6.21957627559762	2.68066987640715
H	-0.45416053671537	-6.69665415390774	0.17687398536756
H	-1.07851939015387	-6.51817080162903	1.80343335067924
H	3.25118601070444	-2.57000358270336	0.06829687806394
H	3.35126351357650	-0.77527915823875	1.67342509031311
H	2.35276264538018	-1.72771370650118	2.87808946039089

TS-12b

69

Coordinates from ORCA-job sp

C	2.85698776402102	-1.57574226890223	1.49315860574893
H	-0.21110920454746	-1.30691776262365	-1.11364087897709
Ti	0.82917516485408	-0.72195134831258	-0.01404016826087
C	-0.10588510318000	-1.24944129652390	2.11336671718240
C	0.18863682150533	0.13430289971123	2.07283035063274
H	0.91967449259750	0.65481819085072	2.67561932980250
C	-0.69584037600153	0.74061095509844	1.16010311089939
C	-0.74370999914416	2.19815460652287	0.83623046788386
C	-1.47847111773084	-0.27721812561350	0.57789435247588
C	-2.61546636031406	-0.15463359564038	-0.39238464802369
C	-1.12185645672169	-1.50836533087471	1.18907998788095
H	0.36083081621586	-1.97602791209094	2.76010370379520
C	-1.80038646292340	-2.80240177831982	0.88028556801132
C	1.90580813081805	-0.18097043537703	-2.12545403001074
C	2.77025474448328	0.35515120611619	-1.16702664224578
C	2.04931314552711	1.30951569874136	-0.40793682841640
C	0.75772315679792	1.43036010809245	-0.98659917021683
C	0.66064461304244	0.48144406497438	-2.02102486193640
H	-0.18202614095087	0.35665984211540	-2.68417036478141
H	2.15335510119281	-0.93271836727890	-2.85580723300794
C	4.24819428743384	0.14948198292884	-1.05867996425925
C	2.71229678851687	2.20601593416532	0.59467331628656
C	-0.30217974340471	2.41770217066934	-0.61066650723876
C	4.11702384471636	1.71220656620886	0.94092990077378
H	2.12231210614858	2.30421984917864	1.50771115493150
H	2.78098189793494	3.21500032005682	0.17056084830407
C	4.89916801020782	1.31453222424025	-0.30827875930856
H	4.66378934770631	0.08438179796893	-2.06725596325128
H	4.49395095046825	-0.80181109870847	-0.58502820871917
H	4.04785810875501	0.86107152829329	1.62047814068804
H	4.65274646688909	2.49692621981331	1.47951412412221

H	4.96077878394742	2.18073679307629	-0.97524286677269
H	5.92594111396937	1.05335021953316	-0.04445071395556
C	-3.50535524779029	-1.39818117830342	-0.33304891140704
H	-2.24510827479605	-0.02345932416723	-1.41266015227465
H	-3.20471547687490	0.73918180676343	-0.16775318300906
C	-2.67923083569717	-2.68238694125524	-0.36300083495340
H	-2.42363233954718	-3.08656028770027	1.73545167691984
H	-1.06275205208802	-3.59776611808824	0.75854456103179
H	-4.21134686650656	-1.38052546068800	-1.16565404939619
H	-4.10346904949700	-1.37822599580657	0.58404151665465
H	-3.33570465947984	-3.55232747171119	-0.43275271081323
H	-2.04634799444979	-2.68646020927803	-1.25539009345719
H	-1.15043938098732	2.30144813009138	-1.28815549329266
H	0.05966808549699	3.44040586675457	-0.75375580254920
H	-0.08279065750939	2.73448720673380	1.51911388295946
H	-1.74707885728462	2.59986564094628	1.00366594226847
C	2.41870490295733	-2.86313736291687	1.22066258529057
O	1.26587916514853	-2.72776180884318	-0.26497980938080
H	2.60361180596970	-1.15664760692382	2.45724673937753
H	3.80206032606043	-1.26574693448012	1.07566531086095
O	3.18713514596695	-3.79089832267195	0.61074123457447
H	1.65231046050012	-3.28987543165246	1.86773459249052
C	1.54521131809936	-3.83256838187254	-1.03674164511857
C	2.35284452738541	-4.72468692853587	-0.06613986652623
C	3.15340733504341	-5.84665338161487	-0.69483378641817
H	1.65766959410621	-5.16271793122646	0.66665961937532
C	2.34264614975242	-3.52143049254981	-2.30345877083901
H	0.63265957515140	-4.37967157180298	-1.33135769425967
C	2.94607280065002	-4.76873047066200	-2.93746588010734
H	3.15016032964670	-2.82462387884240	-2.05872188219839
H	1.69035973777348	-3.01156686225000	-3.01677221063535
C	3.89672525200815	-5.44973603150190	-1.96300209798851
H	2.43718118228842	-6.63712971428083	-0.94040961915532
H	3.82927820466778	-6.27842357614132	0.04795319504437
H	2.15111023429192	-5.46550847544080	-3.22703431717053
H	3.46914992852442	-4.49927205410299	-3.85788647842965
H	4.34386223382124	-6.33826403567446	-2.41430483203383
H	4.72274870436669	-4.77297826839301	-1.72537260547082

TS-12c
69
Coordinates from ORCA-job sp

C	0.10159768281592	-2.84262060594057	-0.53751434799106
H	-0.59223794367775	-0.80574339116685	-1.08175605399058
Ti	0.32846320538613	-0.55472915037311	0.21055984775309
C	-1.19635959474276	-0.77894514904170	2.04451627284658
C	-0.49477192663485	0.44504138870819	2.14257651744698
H	0.20550291229772	0.71910195710308	2.91891571119200
C	-0.93245081871273	1.27435694068943	1.08219670044513
C	-0.49477300845903	2.67390983477554	0.78900165397297
C	-1.85555356963365	0.53983633780916	0.31099022428544
C	-2.66856629957439	0.99395275959301	-0.86609041308440
C	-2.02668474342628	-0.72813687775369	0.92191992863044
H	-1.11811951173521	-1.61419416280865	2.71710679564399
C	-3.03878319826118	-1.72148401648463	0.44840416001669
C	1.95763473727185	-0.45274490291280	-1.57931333716220
C	2.72818461801726	-0.07188042467027	-0.46955206054965
C	2.13857732719153	1.06794168768493	0.10523390810728
C	1.05486391694118	1.46694191734384	-0.72709646322429
C	0.93746316480847	0.51910243783650	-1.76270961702292
H	0.25992102470598	0.58082454238283	-2.60117784014728
H	2.14739802999609	-1.29423763770133	-2.23095813302569

```
  C   3.99095496308289     -0.68693242497667      0.03825163209029
  C   2.76561777556451      1.80942679422765      1.24756456975470
  C   0.23390906522098      2.70723077515619     -0.55604951353998
  C   3.84549302691492      0.97039233656018      1.92811285324837
  H   2.02785535762982      2.12066047481216      1.98799198101556
  H   3.21478053543620      2.73598561180322      0.86842880861208
  C   4.76283492116426      0.30354634649703      0.90891331563503
  H   4.60592065068002     -1.01446328154613     -0.80430767718847
  H   3.76628572363670     -1.57880094588710      0.62516654843238
  H   3.36944538273265      0.20234607054497      2.54363442826488
  H   4.42535190629012      1.60297486829265      2.60393024072500
  H   5.22231330217873      1.07199067031458      0.27857816426982
  H   5.58126843833252     -0.21316836333551      1.41477623794724
  C   -3.88218944435740      0.08592229036084     -1.07384537556007
  H   -2.06475724050915      0.99999044433791     -1.77700693629388
  H   -3.00215598899586      2.02506855014325     -0.71593168190484
  C   -3.51077394666709     -1.38996852044832     -0.96431828538698
  H   -3.89969518686977     -1.68741995783132      1.12576318510693
  H   -2.65846661737311     -2.74313488354834      0.50488073082870
  H   -4.33002891161304      0.29596737895743     -2.04724937604010
  H   -4.64543053274074      0.31684270971396     -0.32337026538903
  H   -4.36702594964581     -2.01587563653089     -1.22425371149514
  H   -2.71788914924701     -1.62170426129539     -1.68206905743573
  H   -0.48924862831911      2.77287060027056     -1.37184937686563
  H   0.86176025453046      3.60026169580226     -0.63287767977033
  H   0.15584981670205      3.03006066983142      1.58851401477676
  H   -1.35529750116357      3.34948844536341      0.77659204024781
  C   1.28010084733162     -3.18952398922861      0.11268438833444
  O   1.55712265928098     -1.75471954448043      1.45706946971273
  H   0.16719249495812     -2.77054878407945     -1.61554675870342
  H   -0.82772722778773     -3.24655436880639     -0.15301122031693
  O   1.35258545624210     -4.13005803950982      1.05787403874043
  H   2.21844135051280     -2.98648667130637     -0.40557419138660
  C   2.02643465750965     -2.47222312680346      2.52952391943876
  C   2.43087391677638     -3.84382862179157      1.94500266606095
  C   2.66222700337464     -4.96573137231844      2.93651355393657
  H   3.34403067004024     -3.72037435622943      1.34402481214512
  C   1.02430837290541     -2.64586067577011      3.67275435036666
  H   2.93987134803560     -2.01467232001716      2.95412386018060
  C   1.49368096066353     -3.65621151948774      4.71101937172574
  H   0.07698463069309     -3.00522870864569      3.26353144645994
  H   0.82494833047476     -1.67192394287033      4.12794140663264
  C   1.63772798944409     -5.02633752676126      4.06110213762118
  H   3.65029773581323     -4.79000708439199      3.37320113251192
  H   2.73528348240627     -5.91769291217572      2.40421565778664
  H   2.45285322226686     -3.34743937408370      5.14266238366101
  H   0.78176082796709     -3.69861077865028      5.53843855840524
  H   1.94390059487802     -5.77691151233577      4.79320161458691
  H   0.66500264904314     -5.34533671291852      3.67426413387076
```

TS-12d

```
69
Coordinates from ORCA-job sp
  C   0.51304473784080     -2.66013915679865     -1.56812976595712
  H   -0.38553978890441     -0.64020056094294     -1.29885008871984
  Ti  0.74698313531553     -0.71539985848879     -0.16151294780583
  C   -0.30661499063506     -1.57240444428884      1.83903662132853
  C   0.26181357119682     -0.30586073845298      2.08834922275088
  H   1.07635416467174     -0.10045809193618      2.76855497069455
  C   -0.47469221705373      0.65144708870563      1.34793567864580
  C   -0.26714457921546      2.13373860983206      1.33022335168270
  C   -1.45015510676989     -0.03887866799901      0.60252769020569
```

C	-2.54989057554397	0.52816590402748	-0.24629515100751
C	-1.34414345562045	-1.42106669220851	0.91133048895826
H	0.00994003324112	-2.50736522912446	2.27884350899225
C	-2.31495401551920	-2.43939588301999	0.40050506627393
C	2.06461078287373	-0.00559107306611	-2.02967695651387
C	2.93913541390388	0.18844849708112	-0.94946405786461
C	2.34482189102235	1.09385490081605	-0.05311149582865
C	1.12001697991086	1.53589131714231	-0.63152049473037
C	0.95018991330405	0.85381398748989	-1.85422158317736
H	0.16850459805605	1.03480167474642	-2.57676167584749
H	2.23788686614991	-0.65458203343789	-2.87507384710273
C	4.29897846470061	-0.38725088314734	-0.75714130950530
C	3.06982770939658	1.63809420667363	1.14039401057939
C	0.19086974339034	2.56453661168564	-0.06392435083659
C	4.31125186618708	0.80756684484051	1.45878844484433
H	2.43090802679048	1.68955161444388	2.02376014649921
H	3.37006933953016	2.67134926003736	0.92811629566508
C	5.11187812664226	0.47902087262339	0.20333816692986
H	4.80858557057557	-0.46957254992326	-1.72051406614008
H	4.20598323127304	-1.40104605467944	-0.36525864071519
H	4.00465073544549	-0.12442895636551	1.93968108187306
H	4.93419708035882	1.34453295776870	2.17766550123263
H	5.40354459170021	1.40945029067018	-0.29478284492848
H	6.03912795159649	-0.03357805300943	0.47088318974546
C	-3.68861922452365	-0.48188173807825	-0.39427641514426
H	-2.17136074768487	0.79786309849655	-1.23544914887981
H	-2.92695727981480	1.45345577600971	0.19963846946275
C	-3.16576252184116	-1.87425746104067	-0.73645830917915
H	-2.97384153806151	-2.73497657866204	1.22437073445973
H	-1.81000733928089	-3.35696204821957	0.08686051382169
H	-4.38341309277786	-0.13912631502029	-1.16379462021532
H	-4.26030325887910	-0.53094528049726	0.53838924016208
H	-3.99706060820731	-2.55221264367452	-0.94001329907910
H	-2.57318012632302	-1.82098921231506	-1.65563630017939
H	-0.67021684244330	2.66183396515780	-0.72798854792166
H	0.66509484604348	3.55040306377762	-0.03175401020156
H	0.47934567652995	2.40382286887537	2.07772934144339
H	-1.18565529101814	2.65497655520146	1.61533852921291
C	1.11972335713389	-3.40746852237051	-0.56373349498215
O	2.20521392169164	-4.17015296774659	-0.75670508623912
H	1.03569268120437	-2.54100605267524	-2.50948110357565
H	-0.55870876969473	-2.78381529460925	-1.65510606888426
O	2.12905046637412	-2.19550261396333	0.54790931394505
H	0.46439613674264	-3.73700501858355	0.24629182669669
C	2.95732589490605	-4.36277669998255	0.45048666745632
C	2.50779800451391	-3.21343489312923	1.39396239257727
C	3.54291056038386	-2.88416060310860	2.47247835073801
H	1.63448547816704	-3.59125392500594	1.95845777976546
C	4.43027135187640	-4.35238934212924	0.07516922486925
H	2.69671000599422	-5.33224847636348	0.89205998522423
C	5.32093384276224	-4.20126133468409	1.30206260586396
H	4.62139279209218	-3.52266712696046	-0.61091911334395
H	4.67575351175090	-5.26908727513478	-0.46702592671408
C	5.00095005815659	-2.90385920908459	2.03331406302078
H	3.42345613768472	-3.63672179365965	3.25994638431497
H	3.27501706610434	-1.92776964729052	2.92836453715723
H	5.17614126035707	-5.04960359757208	1.98003647296488
H	6.36962572631029	-4.22431722937090	0.99893202068818
H	5.64297806153110	-2.79443919478503	2.91065537260307
H	5.22436000642702	-2.05447894349562	1.38470345787089

12

```
69
Coordinates from ORCA-job sp
  C   -4.32652611980722      0.03145384615884     -2.24960898345564
  H    1.64815973390338     -0.37232197279228      1.55649156035489
  Ti   0.84265700099187      0.33213055008501      0.29668979526505
  C    0.91956021746932     -1.20706859450905     -1.53241117724203
  C    1.24371739975787      0.09130020964872     -1.98249173887633
  H    0.68182546662774      0.66956827118167     -2.70226238641483
  C    2.48701244910322      0.45925061255595     -1.41405110029077
  C    3.20085067783665      1.75364251165680     -1.65917543757336
  C    2.88073575416446     -0.58254954287150     -0.55293235638575
  C    4.15464618081974     -0.72475203419066      0.21935413074805
  C    1.91057962957156     -1.62031151386443     -0.63528332621738
  H    0.04640680427303     -1.77040502469861     -1.82763497688133
  C    2.03443117238462     -2.91151207399450      0.11568915554612
  C    0.43145362495666      1.92465738776232      2.03329454820211
  C   -0.33851819231384      2.35809529726712      0.94911970155649
  C    0.54047042349308      2.64455073928786     -0.12519332989381
  C    1.87390306458112      2.47479300248582      0.34180732218375
  C    1.79840906483443      2.00587367764526      1.66190632834781
  H    2.64317438467440      1.76549418078945      2.29029155303891
  H    0.05585268667432      1.58144518232880      2.98664292057720
  C   -1.82400488260095      2.49995334689941      0.86581045183711
  C    0.04901537813744      3.22189353203653     -1.41732688065726
  C    3.14027824916955      2.67359088091969     -0.43142084601574
  C   -1.43692311578356      2.92055639171556     -1.60065669064611
  H    0.61936236567021      2.84983437555700     -2.27035014620743
  H    0.19915835957852      4.30775830907881     -1.40631818176178
  C   -2.23379517928080      3.31609340494246     -0.36133347863366
  H   -2.19740754532589      2.97930321051139      1.77452830890902
  H   -2.28124428634776      1.51105743969203      0.83040526757340
  H   -1.57763500257822      1.85182012910468     -1.79358723315537
  H   -1.81590332786410      3.45061230505986     -2.47718137220540
  H   -2.07897469169352      4.38287179881196     -0.16709936336013
  H   -3.30243646703168      3.18525271561577     -0.54371648717072
  C    4.08758181830614     -1.89804201067323      1.19543867447251
  H    4.40149797449173      0.19562547489964      0.75302619816403
  H    4.97208539890696     -0.88927018949323     -0.49259195733669
  C    3.48329660943040     -3.14078153144547      0.54780358774345
  H    1.69209311669930     -3.74102476295879     -0.50770713655594
  H    1.38453431338273     -2.90470278554140      0.99641004167656
  H    3.48438948520846     -1.60812594691221      2.06010204404660
  H    5.09001905194736     -2.11895712922827      1.56780068548359
  H    4.08310040851393     -3.42245749160443     -0.32429015464724
  H    3.52837223891732     -3.98484364333699      1.23928086475113
  H    3.98126139699154      2.46857073609480      0.23353108030326
  H    3.25074694652944      3.71708182627047     -0.73926263551437
  H    2.74288133380937      2.24819808957991     -2.51792134794803
  H    4.24145581400917      1.57120142471118     -1.93930118559809
  C   -4.24911831966875      0.17365709591162     -0.92764228115506
  O   -3.49248720028457     -0.49263610414722     -0.04452276801595
  O   -0.75583652671340     -0.46195616896292      0.63472738561666
  C   -2.60535067421520     -1.49754228652547     -0.51476332424174
  C   -1.46576986552709     -1.64192429829521      0.49622931712507
  C   -1.97348083645688     -2.15248140490570      1.84117108661855
  H   -0.82473116623307     -2.43558795295693      0.07684302632651
  C   -3.33809454188647     -2.82685453982665     -0.68173426167045
  H   -2.17581987179642     -1.17976491158752     -1.47385526964540
  C   -3.84191569626813     -3.36854646128726      0.65273038462831
  H   -4.16121996344309     -2.71898107253544     -1.39186973896148
  H   -2.64272223248882     -3.54275470002553     -1.13203664323368
```

C	-2.71030948623145	-3.47830130457539	1.66889205114962
H	-2.63037259555307	-1.40890138768316	2.30123882666706
H	-1.12111013766905	-2.27884351905047	2.51424605181123
H	-4.62665622912540	-2.71503399177049	1.04400619334049
H	-4.30582228219052	-4.34541069919576	0.49857993667485
H	-3.09617416120943	-3.81930744291124	2.63210797862267
H	-1.99911028708504	-4.24197513354991	1.33456090913752
H	-4.99097087548618	0.68286561390266	-2.79718103529394
H	-3.76799212148693	-0.69495885303987	-2.82229260696963
H	-4.85076501407052	0.92006122047818	-0.41967972566704

Chloride-Hydride Scenario

(*R*,*R*)-(ebthi)Zr(H)Cl
47
Coordinates from ORCA-job sp_pw6b95

H	1.25117809928225	-0.46862351208335	1.30312167678636
Cl	-0.27495374263755	-2.63856926427093	-0.04130562731457
Ti	-0.00220906294237	-0.32708427062932	0.17497282717698
C	-1.33632595260286	-0.47308131154733	2.12921540421685
C	-0.53303228117615	0.69693402890230	2.21105463561186
H	0.24601423456363	0.88786555138585	2.93553493318262
C	-0.92347805976276	1.57983530665765	1.16851782522326
C	-0.29134175472100	2.90611749965534	0.83942646870078
C	-1.99447181975807	0.95177009251936	0.44930384040443
C	-2.89374111875451	1.54650905388321	-0.60660952180462
C	-2.25014707306788	-0.30846549447865	1.06014610904508
H	-1.24127090922915	-1.35381890032727	2.75059940340092
C	-3.34261033289904	-1.23142028109294	0.60442872230880
C	0.91935674696573	-0.52475729375499	-2.00021106138036
C	2.02400432375020	-0.35181652408326	-1.13872112367179
C	1.90606167505573	0.93295415011056	-0.52202245890601
C	0.72680261641353	1.55738470305882	-1.01695562089161
C	0.10423420587375	0.63586053586737	-1.91383033118817
H	-0.80553278022642	0.81324148721053	-2.47118450722259
H	0.70140048800394	-1.41061251589980	-2.58111024836476
C	3.16402632493385	-1.28998130299397	-0.85606329993417
C	2.97065675296646	1.50381541062066	0.37303949535954
C	0.18371919399343	2.91104331461981	-0.63100436133725
C	3.94352184817507	0.41497830563988	0.85327269094070
H	2.52931677300877	2.02485064457490	1.22863985537318
H	3.52534953295588	2.26167885403274	-0.20043862278749
C	4.36687519359711	-0.51237929562868	-0.29515160114947
H	3.43951260970039	-1.82622860731289	-1.77137859079968
H	2.84375586009698	-2.04869536011417	-0.13044580275427
H	3.45252965560988	-0.18226531011083	1.63185756599925
H	4.82322113601046	0.88750522061898	1.30422460125773
H	4.81922023504922	0.08717460745821	-1.09722515537819
H	5.13049206427015	-1.21782746203221	0.05034115774898
C	-4.17805073168553	0.71161513071526	-0.76472204849544
H	-2.38783522976095	1.61416859752269	-1.57493973964532
H	-3.14452942070234	2.57667003392163	-0.32362934922219
C	-3.87291127689285	-0.79323983538270	-0.76813099209995
H	-4.15980957537761	-1.19162627800450	1.33919024068710
H	-2.98237782970498	-2.26484229356888	0.58213281593444
H	-4.68925762383648	1.00560557815694	-1.68757191815409
H	-4.86077105861509	0.93000397997620	0.06733805289515
H	-4.77275453815715	-1.36512278011183	-1.01831077032872
H	-3.12047214782066	-1.01490878605465	-1.53779345488738
H	-0.65503547229734	3.14920395925180	-1.29128522446650
H	0.94139618634172	3.68878932455952	-0.78344168581908

```
  H   0.55874972680295      3.06366131290208      1.50999718622900
  H   -0.99451539079233      3.73061439566083      1.01553820952065
```

TS-B

```
69
Coordinates from ORCA-job sp
  C   0.85742008135175     -3.28574203796034      0.63211969283349
  Cl  -1.16087295924948     -2.81849023716621     -1.40776311903017
  Ti  0.09953890086518     -1.21811987385075      0.09581779692914
  C   -1.34588874723125     -1.51910966398016      1.96113051794362
  C   -0.53750073360521     -0.37114011967946      2.16152776368605
  H   0.19651560205357     -0.23038786233792      2.93989461582580
  C   -0.90360423000734      0.59069501594603      1.20266671417218
  C   -0.30992288062198      1.95105954361213      1.02450065300999
  C   -1.97049477471476      0.04868716162360      0.43106384713237
  C   -2.85425612367050      0.73880535723919     -0.56471940169289
  C   -2.26408344790189     -1.22826362861824      0.93674449534726
  H   -1.35003033307270     -2.41201394615136      2.56569538763258
  C   -3.47079317082691     -2.01524282017977      0.54735447488274
  C   1.16327030800499     -1.09194439139193     -2.08695589471863
  C   2.23109597072116     -0.91257995959751     -1.20713859452946
  C   1.97552386703951      0.25988982700286     -0.44318551109305
  C   0.76673071566989      0.82347796398834     -0.90353980774910
  C   0.24303673524610     -0.03864507317441     -1.89290774537482
  H   -0.67440359111516      0.10738232463511     -2.44298124127457
  H   1.05662703753091     -1.90857533149564     -2.78245103736099
  C   3.56187616726959     -1.59564442460221     -1.20906252550265
  C   3.01231674923227      0.92433138288119      0.41131583922401
  C   0.16738801044800      2.10676632700721     -0.42108417101235
  C   4.33832409836249      0.15326534569258      0.43468981177273
  H   2.65600877497250      1.08876177951008      1.43111544375224
  H   3.18863853770772      1.92301394314616     -0.00319748639298
  C   4.64012430008401     -0.56005488869986     -0.87975590925613
  H   3.74319248261214     -2.03353604212807     -2.19278396706269
  H   3.61666646228060     -2.41395172889482     -0.48982824744906
  H   4.32130897333139     -0.59108843442470      1.22957475846793
  H   5.14608450318340      0.84531306618200      0.68068931944513
  H   4.70666979982695      0.17017861461841     -1.69309444081570
  H   5.61597267039162     -1.04691457666941     -0.81866198480152
  C   -4.25686367095776      0.13009907943535     -0.52548450913554
  H   -2.46250378567750      0.65630284443268     -1.58156574827479
  H   -2.90477732525351      1.80860812530060     -0.34232520034654
  C   -4.20000608362150     -1.38936873086755     -0.63953434265867
  H   -4.13866418782103     -2.02918202459977      1.41649957762137
  H   -3.20850174392912     -3.05205108922468      0.33712247224938
  H   -4.85851571050311      0.54877393839541     -1.33519109739269
  H   -4.75016940231790      0.41302130713724      0.41034865040289
  H   -5.20962516395155     -1.80062463211558     -0.70642702842446
  H   -3.68096178018066     -1.66248072235481     -1.56029374302234
  H   -0.67079155195219      2.37752481994482     -1.06533898023865
  H   0.89217080504572      2.92227719063689     -0.50343418357024
  H   0.52230778793409      2.07855833060874      1.72001592536699
  H   -1.04184477709051      2.72644039186825      1.27141580386549
  C   1.57289882576405     -2.73811074872672      1.70983939098138
  O   1.07630747956985     -2.78623331335992      2.96673839550500
  O   2.91656114510441     -2.70270053445427      1.75606276801927
  H   1.31332882385775     -1.20987928021404      1.34414182353306
  C   2.16697810063060     -2.72508960456623      3.89720483931660
  C   3.26386848638129     -2.13096814771154      3.02446121878308
  C   4.69020225835063     -2.44608896231978      3.41640763256096
  H   3.13226508711631     -1.04261858950802      2.95313431917292
  C   2.46886697223690     -4.12230978142350      4.42680626651292
```

```
  H   1.89230158196744     -2.04878573940439      4.70858780521532
  C   3.88373166608366     -4.21395008776621      4.98698812973782
  H   2.36625153827655     -4.85040556029655      3.61668799376968
  H   1.72798724220176     -4.38903948384038      5.18305598194943
  C   4.89666601802543     -3.87498958027768      3.90089999756024
  H   4.95037701179021     -1.75532287321054      4.22359747125367
  H   5.35733458867998     -2.20134690416557      2.58642183055372
  H   4.00407559650725     -3.52756552421134      5.83206098413524
  H   4.05593332377240     -5.21886500326914      5.37579366672257
  H   5.91622906332121     -3.98049439873940      4.27602835539633
  H   4.79423123349964     -4.58001873789743      3.07114520374862
  H   0.00823357671051     -3.90989270128880      0.87370245503233
  H   1.44275721425995     -3.61876588402812     -0.21662417284296
```

TS-iso-B

```
69
Coordinates from ORCA-job sp
  C   1.62011797206801     -1.34858532875209      2.16734649455801
  Cl  -1.33596569359276     -2.56217684658306     -1.06047016637793
  Ti  0.09473992919938     -0.91078215610160      0.33482373660490
  C   -1.23001979789885     -0.56365361401997      2.28068604723668
  C   -0.49034222394099      0.61108726347465      1.98651799998237
  H   0.29874400397729      1.04403749253783      2.58312715043482
  C   -0.98728083438440      1.15735841640264      0.79224677641599
  C   -0.49658428310437      2.40084623544245      0.12067921475437
  C   -2.01607310796325      0.30230891981929      0.32806966758250
  C   -2.96589389631755      0.49289200285238     -0.81018109237735
  C   -2.19469946723996     -0.72732768766763      1.27878470708919
  H   -1.10966250378219     -1.20222491661732      3.14304019875752
  C   -3.33675156454571     -1.69163522994519      1.25710801455619
  C   1.47506579567471     -1.38941568664112     -1.57216846666490
  C   2.41191044766742     -0.80790088861237     -0.71072509997785
  C   1.97887384737484      0.50798835230699     -0.40974606707257
  C   0.83456337478386      0.77706588099523     -1.20009378393077
  C   0.51017209498165     -0.39869407134228     -1.89239820675785
  H   -0.32531383663407     -0.51288238670587     -2.56469784334945
  H   1.51694410919374     -2.39079121508374     -1.97159794256542
  C   3.78024418320486     -1.30152995055830     -0.35023944192714
  C   2.79523806715223      1.48123868237751      0.38873045397563
  C   0.06796505287425      2.05932289419676     -1.25824445062126
  C   4.15103395704777      0.89960195904280      0.81773371957927
  H   2.25782521197940      1.82766433452075      1.27565527352721
  H   2.96207850293912      2.37400266749712     -0.22282861752077
  C   4.71367113963186     -0.10029579477957     -0.18669274343622
  H   4.14272313600130     -1.95934749746379     -1.14302478650409
  H   3.80227450079251     -1.90232327290927      0.56107582482985
  H   4.06048484222358      0.40827352059412      1.79043349947475
  H   4.85772996716666      1.71828306961130      0.96607916242950
  H   4.85780980982993      0.38664446211338     -1.15623879367868
  H   5.69828537719548     -0.44006337861675      0.14150169144228
  C   -4.34660739217918     -0.01533059732234     -0.38806703444765
  H   -2.63259299635566     -0.06025622003904     -1.69301655174906
  H   -3.01883921550644      1.54760340554977     -1.09198988699729
  C   -4.28655505940566     -1.47037277173260      0.07087095433403
  H   -3.88813504523720     -1.55984527493765      2.19397310441389
  H   -2.97108531524803     -2.71934084272337      1.25592548020291
  H   -5.04553733718562      0.07623278737979     -1.22261135647266
  H   -4.73359015247225      0.61967774615057      0.41597802839231
  H   -5.28687776978234     -1.81353183230563      0.34428187956380
  H   -3.95712246706979     -2.08214318524583     -0.76865470431107
  H   -0.74612081315512      1.94524129557506     -1.97686568504626
  H   0.69502461488827      2.87385002312605     -1.63298197663245
```

H	0.26911222761105	2.86650609893361	0.74322936102435
H	-1.30361812245271	3.13420273252508	0.03269811418389
C	1.33570483355886	-2.70904609292680	2.10114359767294
O	2.07936947590924	-3.58575911027773	1.42934937620889
O	0.50861290964041	-3.31893619856067	2.94120536854059
H	0.05961803452091	-2.50568113214180	0.80493173000986
C	1.67924073630136	-4.91416841816227	1.82518042135246
C	0.29696091804063	-4.63936678174450	2.40529559290592
C	-0.20094634324199	-5.60447016581148	3.45439420350942
H	-0.43473562920373	-4.54219689049413	1.59365786392738
C	2.69212933109378	-5.46852005635269	2.82159531521594
H	1.62050965244251	-5.53469178703194	0.93049229118092
C	2.09208650900591	-6.57121196473625	3.68649197906946
H	3.03947558256427	-4.66366582931947	3.47588570026442
H	3.56873367421965	-5.82955783207422	2.28043827474845
C	0.87303211498262	-6.05131416050347	4.43713359002619
H	-0.57934507374329	-6.47795124053818	2.91615293814672
H	-1.06226212197601	-5.16792177024101	3.96477884964251
H	1.80580931950806	-7.42864324293149	3.06825976947200
H	2.84902126476101	-6.93345944738993	4.38404583956870
H	0.46180551489526	-6.82654038601070	5.08611993315680
H	1.16443991444710	-5.22184379149659	5.08798007763667
H	1.25993371675584	-0.85212457099945	3.05592294686432
H	2.61805239551251	-1.06401872657381	1.88458248395184

B

69

Coordinates from ORCA-job sp_pw6b95

C	1.42623413683635	-2.16231678784341	0.03797987230225
Cl	-0.74966128218966	-1.45721812448283	-2.16139505953895
Ti	0.33575724734734	-0.33363252327944	-0.41634400593123
C	-0.63584957113654	-0.96408841016537	1.63820814469580
C	-0.09827488095165	0.33560889169483	1.74077128517105
H	0.71829056414799	0.62418071890828	2.38659262497359
C	-0.86400444850967	1.20573569641109	0.92931008448382
C	-0.65238677767138	2.67643248492937	0.75053297543554
C	-1.83940442521038	0.42039347377771	0.28590803299216
C	-2.95041726735362	0.85989057670736	-0.61171510648751
C	-1.71233583626117	-0.91684880800139	0.73634872899762
H	-0.29691509870955	-1.82873391643261	2.18089413474555
C	-2.70275025761575	-1.98923721800196	0.40863070010513
C	1.92936850693884	0.15684565271183	-2.13315800594060
C	2.65346335590372	0.33953195929921	-0.94108863422081
C	2.02614267301176	1.37637661581424	-0.20962219936254
C	0.94511577626937	1.87398507672982	-0.98686255765045
C	0.89829576326377	1.11778116851451	-2.17007459463466
H	0.16603580299612	1.23635509769149	-2.95553998735015
H	2.13608281247017	-0.58044974652516	-2.89500182615723
C	3.94928466999350	-0.29315903683877	-0.53691828205919
C	2.63806493645840	1.94514034043835	1.03350693845614
C	-0.01234085602929	2.96259574151884	-0.61212949603337
C	3.62810988947684	0.95529952723369	1.63955246225532
H	1.88918610287557	2.24252931293187	1.76649472391390
H	3.16966301910368	2.86461949899130	0.76299568481219
C	4.62885117548350	0.49301184582050	0.58611965605464
H	4.60619211108260	-0.34174439587689	-1.40840147969884
H	3.80285503198611	-1.32943127429715	-0.22314048343306
H	3.08387455292974	0.09289138592405	2.04026518022238
H	4.14864827233139	1.42081888133449	2.47833369401224
H	5.13050913401233	1.37293880539999	0.17107691640255
H	5.40735049524800	-0.12322119783664	1.03890582088981
C	-4.16151571056453	-0.04355564742182	-0.37277244092470

```
H   -2.65229403188391    0.80014511986462    -1.66219364504819
H   -3.20275446899456    1.90505547652516    -0.41455282953435
C   -3.79408763784662   -1.51277102495343    -0.55844316475546
H   -3.16220870629314   -2.30285185459467     1.35129568446388
H   -2.20473884238450   -2.87310546788394     0.00914186108740
H   -4.96596338889375    0.22681875865907    -1.05993103057657
H   -4.54252503290823    0.12569826899049     0.63956449467590
H   -4.68008761889798   -2.13780763747849    -0.42847473070195
H   -3.45830459448852   -1.65807068755251    -1.58603396577329
H   -0.78946751690052    3.01514740614583    -1.37807492059093
H    0.48386325350374    3.93717197401837    -0.60829610756785
H   -0.02601188601368    3.05230965646245     1.55984048802179
H   -1.60196406218823    3.20996921291196     0.83847643013231
C    0.76026554757819   -3.51851421923222     0.11004284565022
O    1.73597648090879   -4.56948401891873    -0.01052591689440
H    2.05859106009978   -2.02068772833633     0.92433317377877
H    2.10310814867461   -2.24311831790460    -0.81743737978028
O    0.10161582595759   -3.76291889732055     1.32268895198600
H    0.05902404467323   -3.64301853855525    -0.72827708733686
C    1.61019820555568   -5.43361760974231     1.10215216434153
C    0.17882136455280   -5.15566235557163     1.54849222617753
C   -0.16058215402384   -5.50929612246146     2.98228663745881
H   -0.51836160642921   -5.68043056174017     0.87632701423636
C    2.65714592982597   -5.12208983660145     2.17169337611707
H    1.72155032823149   -6.46970979605719     0.76506088927452
C    2.27307546597134   -5.69543950549762     3.53085201780599
H    2.76884784093213   -4.03844765470120     2.26837103716680
H    3.62842119629025   -5.50374355942792     1.84694658981783
C    0.92920345204338   -5.13354090064448     3.97832699628361
H   -0.31498709336274   -6.59195716692889     3.01902610321812
H   -1.11909639004422   -5.05691235085082     3.24858616310331
H    2.21340753003326   -6.78853281188705     3.47916525303893
H    3.05017573571958   -5.46577756001225     4.26315210500133
H    0.66265813809643   -5.51385821462555     4.96687917037512
H    1.00058586494152   -4.04583713987653     4.07181560384855
```

TS-Ca

```
69
Coordinates from ORCA-job sp
C    2.55838360310180   -0.98450544767246     1.99580768353979
Cl  -1.20671494432093   -2.34312673198438    -0.87904710661324
Ti   0.57739297990256   -0.90549204877916     0.42797678698174
C   -0.30772232625085   -0.76170737227761     2.60796026003216
C    0.21815288050299    0.50800447556555     2.23444003904464
H    1.07124500993178    1.00490645293742     2.66725494552505
C   -0.64370802909817    1.08079275871031     1.28780481595080
C   -0.46512792831749    2.41714496692931     0.65009733068214
C   -1.65673784363779    0.14486107668005     1.01301949711272
C   -2.89449112379112    0.32019892332154     0.19712527096721
C   -1.47588586455357   -0.97000006946792     1.87486777509258
H    0.10131269912094   -1.42639937282791     3.35475299377304
C   -2.50706292115235   -2.03232767656873     2.07295094315857
C    1.50195807403462   -0.97741915272144    -1.76513186264972
C    2.52944103279005   -0.36761925233656    -1.03898766973446
C    2.03988975612643    0.86675290818088    -0.53253646685460
C    0.73598804971482    1.05224513196485    -1.03515785897925
C    0.39114234061523   -0.09773619169810    -1.76037245789308
H   -0.54439574413687   -0.24995870392137    -2.27053918420436
H    1.55068068321204   -1.93748031791515    -2.25599155251157
C    3.98791119836634   -0.70102446645463    -1.01176678676888
C    2.91426656801089    1.89059361681849     0.12608615231073
C   -0.13586724592488    2.24551277692418    -0.83330952433119
```

C	4.40915650623163	1.52224581932120	0.07592198305011
H	2.62213554345266	2.07105285398682	1.16410325238370
H	2.75932046945549	2.84272185429599	-0.39106585634690
C	4.77400188459643	0.61035828719073	-1.09090640227234
H	4.22972092120094	-1.35685303769452	-1.84985213045579
H	4.30400310314642	-1.23057651903254	-0.11025802119099
H	4.70326248889688	1.02590812700163	1.00445609671700
H	4.99984070557714	2.43891724735910	0.03367754854143
H	4.56368274390107	1.10697004592924	-2.04259699564176
H	5.84553410849190	0.40166868179762	-1.07222676990908
C	-4.06121254161278	-0.37010352828788	0.90853658467238
H	-2.76573386059796	-0.11249566271695	-0.79749274304014
H	-3.11014412253251	1.38312248971792	0.06048935382678
C	-3.75545055842243	-1.83648768801550	1.20254627213050
H	-2.79339473064741	-2.00096488380615	3.12931884672029
H	-2.08373609025559	-3.02239511275683	1.90297113667262
H	-4.96042317081009	-0.30127968897603	0.29196704216210
H	-4.28121067032037	0.16173659145194	1.84040535525976
H	-4.61069602527431	-2.29723998898313	1.70202334618978
H	-3.61857489942340	-2.36122041406493	0.25825308283144
H	-1.05227232942881	2.11881059828567	-1.41230759295487
H	0.35111190399427	3.14400303361230	-1.22272836191087
H	0.33814661012961	2.95179750738810	1.15891719898989
H	-1.36403016713851	3.02683092182646	0.77208591839930
C	2.66267900647871	-2.32993285968517	1.66387324676060
O	2.42259645167299	-3.28217000105952	2.57420564413066
H	2.26747975018608	-0.75703186506068	3.01171918079635
H	3.29519880220647	-0.31589819163425	1.57943348424452
O	1.11293684729528	-2.78503849076100	0.63845881712453
H	3.31627325936106	-2.60998492072710	0.83655642378637
C	1.80199260307497	-4.46517535594985	2.03446571914122
C	1.45401857153642	-4.12440698797604	0.57596830336873
C	0.46068251797383	-5.11355475643325	-0.02438388744189
H	2.36814436427441	-4.22491637734507	-0.03502198040378
C	0.57022555598926	-4.75284837329851	2.87918302777611
H	2.50730812531407	-5.30164576144273	2.08432700252719
C	-0.28873647415286	-5.84683618520118	2.25378732271735
H	-0.02129975336346	-3.83638679052028	2.95734457688330
H	0.87333423075100	-5.02721948008907	3.89276709349538
C	-0.73457845123748	-5.44565518512631	0.85283508350889
H	1.03075494571048	-6.03291912825321	-0.20453705741949
H	0.14320741451092	-4.74313917314958	-0.99809946881536
H	0.27667093242699	-6.78455413870808	2.20698057288378
H	-1.15299071022661	-6.04255538655613	2.89233732294972
H	-1.30664129626295	-6.25529990182805	0.39253279829338
H	-1.39772342037643	-4.58113550743185	0.89226260523709

TS-Cb

69

Coordinates from ORCA-job sp

C	2.57743839381513	-1.34526719754872	1.77642212725889
Cl	-1.09001802739615	-2.24074716840605	-1.35176201762733
Ti	0.57484627314045	-0.98905635727011	0.14012938185772
C	-0.40428474370848	-1.25694736119257	2.27331028900233
C	0.18266449909302	0.03871926404361	2.18657177505348
H	1.02522035809374	0.40671381507364	2.74999589401216
C	-0.60632676644112	0.82085922178028	1.33484312471493
C	-0.37536052555246	2.25143704797752	0.98425859795857
C	-1.63477065829240	0.00154900856913	0.82944249000212
C	-2.80906979954735	0.38842680617830	-0.00762330277863
C	-1.55275972933920	-1.25866494300469	1.47524686007588
H	-0.06229806743937	-2.06739408755974	2.89911035243372

C	-2.66657666033035	-2.25603198468607	1.44983264037363
C	1.54813865305134	-0.63899752777054	-2.03441595794112
C	2.58206574490023	-0.16319790344123	-1.22463360728516
C	2.08369217363471	0.93879394042327	-0.47965006031431
C	0.77763122375102	1.21205121761153	-0.93210663763074
C	0.42731626803566	0.21164627257909	-1.85197128804904
H	-0.50376981919411	0.15621651030591	-2.39159010852793
H	1.59689092607142	-1.48491305070183	-2.70233169176257
C	4.05169069802956	-0.44406359696568	-1.31677278366639
C	2.94788053565600	1.82102141266759	0.36875732033598
C	-0.07748198473377	2.35875776726855	-0.50934826418518
C	4.44069900314673	1.46735999412511	0.26308460662277
H	2.63813808923594	1.80305402018761	1.41720984938934
H	2.79871769361872	2.85412419485667	0.03752648098806
C	4.81175738849730	0.86401149721805	-1.08708514417414
H	4.27786052280266	-0.85656193416265	-2.30179777568157
H	4.41711423934431	-1.17676958646610	-0.59567655053815
H	4.71585603379614	0.75353585026850	1.04474007770001
H	5.03347239838617	2.36300382759946	0.45585244271164
H	4.58702578897041	1.57097678226393	-1.89187729540793
H	5.88644076163003	0.67546716734799	-1.12549704621978
C	-4.05462174193514	-0.32670374787397	0.51981518723024
H	-2.64834374918555	0.11070408326465	-1.05240205159367
H	-2.95436521445772	1.47104849444468	0.01840780015774
C	-3.84382814991575	-1.83637818246663	0.56142428430384
H	-3.01235329279556	-2.35955598611936	2.48382770738544
H	-2.31177345876366	-3.23916211744569	1.14932733539292
H	-4.91131525937756	-0.09036798326903	-0.11528052291565
H	-4.29124221450903	0.05070241557135	1.52003327168676
H	-4.75025110065692	-2.32895934949774	0.92068260225350
H	-3.67138748110328	-2.19294683165591	-0.45427296475656
H	-1.00282938568455	2.34236692832577	-1.08641401610598
H	0.41260464151030	3.30868969055727	-0.74122653245137
H	0.46063606121327	2.63631866589895	1.56991163865663
H	-1.24486193815159	2.85740915626947	1.25268383948829
C	2.85642565014309	-2.52129556930116	1.09781015774220
O	2.76588128192519	-3.72615553776470	1.66071613815627
O	1.26756902252882	-2.84770104285088	0.01694499928221
C	2.46798472423263	-4.70030506813138	0.65881199471840
C	1.10798459458304	-4.21977652974827	0.10133714416633
C	-0.05180729075210	-4.66237007754182	0.98917896399606
H	0.96723263563057	-4.66253364764948	-0.89584511416861
C	2.50722364089005	-6.09908591896670	1.23409019497569
H	3.22362360437845	-4.61856835675400	-0.13573647664909
C	1.31723697990307	-6.42193142018682	2.12662950189274
H	3.45819669303345	-6.25474419862271	1.74983145260507
H	2.50708723623942	-6.78819341871494	0.38393863389455
C	0.02379026806857	-6.13405099450284	1.37726688217229
H	-0.98210425152458	-4.45074449544273	0.46473464220758
H	-0.05430396966648	-4.06019742520523	1.90181034901917
H	1.36549399172805	-7.46852099729246	2.43509293898518
H	1.35290680789056	-5.82339755184264	3.04204705573503
H	-0.02523073807792	-6.76533879770071	0.48293329559761
H	-0.84290187189433	-6.39594281269451	1.98820019892608
H	3.49112461663084	-2.48861829793148	0.21705632276579
H	3.24032663435605	-0.50897174457659	1.61201391266643
H	2.20965113884029	-1.44332425175137	2.78939445387963

TS-Cc

69

Coordinates from ORCA-job sp

C	2.68539227012303	-1.32867246713758	1.49468107211480

Cl	-0.66450900053460	-2.11539034510742	-2.07440655591968
Ti	0.69497679446521	-0.96229352218303	-0.13977808547946
C	-0.44444078418212	-1.62445178735877	1.86316864393111
C	0.00083517554351	-0.28999333951539	2.02586569253367
H	0.72077978272061	0.06837614430792	2.74321587215139
C	-0.74117332949151	0.53587973152183	1.17261935192073
C	-0.59375493345706	2.01812903987426	1.08580906078468
C	-1.60528749613198	-0.29328584310129	0.42483351700884
C	-2.73110550221026	0.08815113216462	-0.47989428915660
C	-1.45090785884144	-1.62456730430365	0.89438608129091
H	-0.11111934030014	-2.48386702272085	2.42498051232079
C	-2.36836473660363	-2.74690028123592	0.53168308850459
C	1.70656039336116	-0.23121605295424	-2.18567995262756
C	2.67202137797924	0.11934721075924	-1.24276929099803
C	2.09795368707013	1.08182191894862	-0.36308446478138
C	0.81102617340884	1.39046414187179	-0.84710121925662
C	0.54530804058956	0.53814696136634	-1.92777580861248
H	-0.35675517537542	0.54209096628887	-2.51731557912063
H	1.83018749574651	-0.93759079001059	-2.98916606341147
C	4.13967322806020	-0.16664201681365	-1.26229755490070
C	2.88451314176503	1.83158259395584	0.66810641779129
C	-0.14277789680716	2.40900059009785	-0.31923989030790
C	4.39319371669740	1.52411131218144	0.61365022614095
H	2.51018711380448	1.64023366069456	1.67700808203952
H	2.72771328183544	2.90097197418932	0.49351042187538
C	4.87766948939797	1.08118020726847	-0.76482044590221
H	4.44505734238859	-0.40225339193155	-2.28399470564270
H	4.41771006048696	-1.03604656308529	-0.66032175059083
H	4.64537132861214	0.75249426168456	1.34339302864902
H	4.94499067392481	2.41265337672129	0.92620694956394
H	4.72987914142741	1.89065636829608	-1.48622067038512
H	5.95120347666358	0.88564862838819	-0.72923958297996
C	-3.91448130450430	-0.84357464894669	-0.20146351814945
H	-2.44182168478328	-0.00150566403226	-1.52973221350092
H	-3.02128804271560	1.12792352451330	-0.30865281928510
C	-3.52447332204971	-2.30817773419346	-0.38093071134480
H	-2.77239201484184	-3.14231344482002	1.46886432954979
H	-1.81021323873171	-3.56007217158160	0.06503875380335
H	-4.74041478863592	-0.59971384478686	-0.87355937008799
H	-4.27763930683423	-0.66908323817118	0.81687056558295
H	-4.39012635791732	-2.94655105063219	-0.19058050798314
H	-3.23659993443180	-2.46132206193002	-1.42010225041757
H	-1.00080367073257	2.45720291245259	-0.99150042224903
H	0.31146831708818	3.40356634682066	-0.32044716840035
H	0.14438567861993	2.33896617274212	1.82291151791256
H	-1.53013453634421	2.51838617915368	1.34761851034176
C	2.49655765161204	-2.69661699555359	1.33796180895184
O	1.27824076026660	-2.84252857378195	-0.19375364820379
H	2.41216288252624	-0.92443789989469	2.45721775744260
H	3.58854473196764	-0.91764845739279	1.07705671541517
O	3.39148787456315	-3.48442647430930	0.74454850696233
H	1.79919474266956	-3.21083074360030	1.99864803143053
C	1.80653577958618	-3.90279931866364	-0.90901389394655
C	2.73632922843470	-4.58914704469724	0.11521770656004
C	3.73830306144614	-5.57511293711575	-0.44576995996312
H	2.11823248250037	-5.09570054052118	0.87051750326286
C	2.56248013471139	-3.50056347748488	-2.17153806176040
H	1.01577080400621	-4.61433963256746	-1.18713685829177
C	3.40320120368703	-4.63902455058474	-2.73565992546717
H	3.23036381583237	-2.66564058304821	-1.94350276449125
H	1.84087656713817	-3.14059983916008	-2.90466019309635
C	4.43440326822195	-5.09356276074983	-1.71167885267743

```
  H   3.17893062384663     -6.48592364035233     -0.67993981620456
  H   4.45589266786133     -5.85104903811832      0.33078236870860
  H   2.76654862245640     -5.48553269201950     -3.01735928245803
  H   3.89622371041598     -4.30946598351359     -3.65315781989864
  H   5.05061537370302     -5.90034539588028     -2.11475313552282
  H   5.11463108722457     -4.26629419070003     -1.48669299107241
```

TS-Cd

```
69
Coordinates from ORCA-job sp
  C   0.41343325502400     -3.10355932369515     -0.53273979952936
  Cl  -0.98180973855594     -1.30998032934721     -2.32982723505348
  Ti  0.32090576544838     -0.78944985206492     -0.02892910841832
  C   -1.32811505010261     -1.28804822952154      1.56709038104876
  C   -0.62220829387309     -0.12036117926235      1.95420736069048
  H   0.05159047964246     -0.00982899152584      2.78658780720346
  C   -0.98375566549656      0.92106725773381      1.08526704270111
  C   -0.48145185591562      2.32724783459462      1.14366349121688
  C   -1.89145976001557      0.39475885997668      0.14219936584584
  C   -2.71223282377601      1.11812998379649     -0.87229302169186
  C   -2.13503345870702     -0.95870280360710      0.47143126985384
  H   -1.30471663790119     -2.25079007385011      2.05094237197871
  C   -3.22080082071202     -1.78256974589634     -0.14065945905145
  C   1.93698463356493     -0.31531503676029     -1.72808726655288
  C   2.72648076138406     -0.16746694098761     -0.57916959277883
  C   2.17747709814217      0.86959156216154      0.20473650456086
  C   1.11548074877725      1.45066071513437     -0.52698815832776
  C   0.95925361277467      0.71428530593472     -1.70739543395209
  H   0.23741142168429      0.92831066279939     -2.47637076609647
  H   2.09115279038007     -1.03130755309381     -2.52217867703254
  C   4.02221012705994     -0.81291675531123     -0.22626887507318
  C   2.81187090929007      1.38693914794146      1.45649566362366
  C   0.30443974166789      2.64035480053836     -0.12824650163244
  C   4.14509642484362      0.69494432151405      1.78015522266949
  H   2.13581784324186      1.29964421427985      2.31058090097001
  H   2.98423316492606      2.46088422636005      1.32672578046029
  C   4.87931413429934      0.19436964659181      0.54094866245332
  H   4.53067892964930     -1.15599333701788     -1.12985585126624
  H   3.87209187411294     -1.68911457948848      0.40650963174569
  H   3.97355124323814     -0.15388584516035      2.44482510868919
  H   4.77980216669611      1.38662468753796      2.33752620170038
  H   5.13534258788675      1.03606361676827     -0.11038268425925
  H   5.82182534546242     -0.27223388579432      0.83506868685425
  C   -4.11250377126226      0.49791041967745     -0.89531782758084
  H   -2.25981266502575      1.05174663968232     -1.86430869384322
  H   -2.78085752567637      2.17911864656696     -0.61669589738728
  C   -4.06970797519636     -1.00477308519749     -1.15780583794662
  H   -3.85357208687102     -2.13159747960308      0.68137506328877
  H   -2.81080791921554     -2.67831412373300     -0.61087503272080
  H   -4.71876679043839      0.98500393447633     -1.66236236111730
  H   -4.60329527084470      0.69827814908491      0.06277117999689
  H   -5.08651730639460     -1.40353975182860     -1.15194475417769
  H   -3.67018916699783     -1.17305949241885     -2.15665673431853
  H   -0.37569499838166      2.89342596830889     -0.94290932556846
  H   0.95065489314980      3.51047601145849      0.01955194891257
  H   0.15622186886708      2.44222146580537      2.02119919357472
  H   -1.31159988899874      3.02754993212998      1.26981083417679
  C   1.54536861594827     -3.31155687635091      0.24398393765371
  O   1.58432183536473     -1.67330356011422      1.40771699503128
  H   0.59125626181731     -3.11036885281494     -1.59768869008448
  H   -0.52684319708953     -3.54238143583203     -0.21348300340607
  O   1.59104440636856     -4.10276944935918      1.30667492268090
```

H	2.51062692997628	-3.08732570904639	-0.21093538252313
C	1.99620832635554	-2.23072077528629	2.60767934833098
C	2.56933339261850	-3.61382005918161	2.22492585120357
C	2.82932407110498	-4.59515084727362	3.34868662448819
H	3.51132808080792	-3.47043978829605	1.67597836309168
C	0.93981308463743	-2.37082640397903	3.70920918440884
H	2.82719937447402	-1.64494254056155	3.03468397637388
C	1.44081891290947	-3.20896341439579	4.88004229949283
H	0.04411833382074	-2.85614262264427	3.31932262744655
H	0.63917231712323	-1.38612156338727	4.06860886142637
C	1.74763656118975	-4.62541647309297	4.41732205583224
H	3.77243561813800	-4.28873431243905	3.81049281300180
H	3.01260995836357	-5.58672455617616	2.92719566251609
H	2.33993634882607	-2.75815434746272	5.31560511387962
H	0.68700295653934	-3.21814976587680	5.67006668999567
H	2.07953817975693	-5.24681874348402	5.25207006192838
H	0.83522728009366	-5.08839751863321	4.02868090839143

TS-Ce

69

Coordinates from ORCA-job sp

C	0.94242597159406	-2.91839700920911	-1.72165863713301
Cl	-1.01249887345680	-0.95525717321540	-2.49392877361080
Ti	0.67330510795431	-0.91450773981265	-0.44142820795277
C	-0.53535801230716	-2.01170293761448	1.30717763700825
C	0.15181538973878	-0.88626543589452	1.82753675667663
H	0.98274301796582	-0.89765432966875	2.51558781595008
C	-0.47188377366164	0.27127426475799	1.33479914531703
C	-0.11203069614006	1.68833212316015	1.64275558781357
C	-1.51354980784834	-0.13551870606581	0.47418826466042
C	-2.59412804961767	0.69834044890286	-0.13091074638770
C	-1.57181390067581	-1.54996021297349	0.48703247875181
H	-0.35851706410150	-3.04496281240266	1.56707039143331
C	-2.69864613462116	-2.33702770701227	-0.10255281604851
C	1.92842785175786	0.13950122505455	-2.16168274484819
C	2.86320424830346	0.08478972225913	-1.11676433161079
C	2.36531636586196	0.84641847060298	-0.04159994714071
C	1.15516899301755	1.45386219650198	-0.45903451241103
C	0.89377673438993	1.02464084867359	-1.76351600379245
H	0.05324515006209	1.33742249171875	-2.35751902147030
H	2.02094135500172	-0.33429733047549	-3.12681446549460
C	4.23714687073298	-0.48306953323130	-1.09440225071720
C	3.17166866662860	1.21505391086970	1.16263797047253
C	0.32255668345311	2.38868518943335	0.35503003018270
C	4.65068598726754	0.80168737190254	1.05138902860131
H	2.75216146943423	0.79624857835569	2.08076399900112
H	3.09952600789878	2.30033580777388	1.28339424679997
C	5.13776522389502	0.52175643120084	-0.36864106298957
H	4.59367966360468	-0.65957449231793	-2.11129507286674
H	4.24759991919098	-1.44468233318969	-0.57792558574592
H	4.80549033188886	-0.09283736157798	1.64809976167304
H	5.27331834598678	1.57522930749184	1.50477483345680
H	5.17253768166611	1.45064441055107	-0.94567401805616
H	6.16155974566934	0.14340401796617	-0.32456184121853
C	-3.90838859986396	-0.08511110008165	-0.07774918009039
H	-2.35807258559591	0.95488081338188	-1.16567535447094
H	-2.69393501189728	1.63813626306211	0.41890742309829
C	-3.78027866771113	-1.45366553857666	-0.74148122897705
H	-3.13920837255030	-2.92031532381286	0.71259293731694
H	-2.33256129563958	-3.06413758199198	-0.82943007398452
H	-4.69737833896023	0.48687866629948	-0.57115596196417
H	-4.21541137939368	-0.20105976865112	0.96721191445908

```
H   -4.73837390096883     -1.97609005798654     -0.69469642496036
H   -3.54872254373515     -1.30886629654824     -1.79548241120875
H   -0.55194278277529      2.68922929858106     -0.22515123249847
H   0.87773586438475      3.30411755850889      0.58156805766375
H   0.68831839699295      1.70849307199823      2.38346716134994
H   -0.96253475849318      2.20598020312877      2.09570500007996
C   1.49602244400700     -3.57372348476791     -0.63138056388541
O   2.68678231463270     -4.15559090139579     -0.60066039764256
H   1.56783136794327     -2.71782017027395     -2.58402220315005
H   -0.07895205854112     -3.19326116690397     -1.93718324044461
O   2.19988505949495     -2.10122959272466      0.45877402193287
H   0.78865660150845     -3.95548595244471      0.10623143879556
C   3.17563509043686     -4.23033437964186      0.74257056899369
C   2.58057759484166     -2.96876118271759      1.46807597052168
C   3.51229102023005     -2.40835724757332      2.55520622075173
H   1.69070636599196     -3.32315835073459      2.01558683411147
C   4.68661164066093     -4.34795834228645      0.65365136021944
H   2.77617534631760     -5.12980366018725      1.22750794487861
C   5.35219062372530     -4.01345381200085      1.97795735957451
H   5.06155167097602     -3.65638512548890     -0.10611006332096
H   4.94713653275811     -5.35512706411882      0.31938783620812
C   5.01094616400604     -2.57702512227380      2.33506250139194
H   3.25926931274268     -2.92849328284301      3.48443697372802
H   3.25747449712483     -1.36048729407241      2.72902993208780
H   5.01709855346169     -4.69500218399975      2.76729670755783
H   6.43239716169956     -4.14390310060842      1.89040258023096
H   5.54076844090892     -2.25628394895483      3.23520677769387
H   5.36563776074447     -1.93610654381362      1.52452290564860
```

C

```
69
Coordinates from ORCA-job sp
C   4.44625488990082     -0.39129706954217     -2.50951394621989
Cl  -1.40924170446231      1.25700480410790      2.05580405719606
Ti  -0.74977292202585     -0.27972409801882      0.36980370514491
C   -0.70631789121730      0.94707739953089     -1.71113781689107
C   -0.98478029051695     -0.42147143545681     -1.92229264192245
H   -0.35780083184354     -1.11315836079715     -2.46699627710226
C   -2.28269023102388     -0.68952765577873     -1.42034035544589
C   -3.01531378447537     -1.99070453508996     -1.51361644604718
C   -2.74364879588622      0.48592706978361     -0.80374911695881
C   -4.09711901896913      0.74711253029808     -0.21746514340894
C   -1.77837679522894      1.51117214141020     -1.01996597319627
H   0.18284585281351      1.46363809102000     -2.04026543534396
C   -2.03265502686249      2.94186636941066     -0.67749855359830
C   -0.42169198056838     -1.67202139865462      2.28681093143443
C   0.37168420176690     -2.27189454795507      1.30368591682141
C   -0.47537060357756     -2.66702974219992      0.24040110258386
C   -1.82039026515118     -2.40413108624751      0.63056411601112
C   -1.77605859294883     -1.76985249634477      1.87752859802214
H   -2.63514301325369     -1.41072007412454      2.42563526744547
H   -0.06601544379914     -1.21945163191373      3.19994062572865
C   1.84897971910091     -2.47697829380958      1.30094978759465
C   0.03914107933291     -3.44935565129793     -0.92924639000753
C   -3.06326881555814     -2.68687347962980     -0.14952998668013
C   1.55137072687778     -3.28637878853924     -1.06664243807993
H   -0.45348941910214     -3.17073051406945     -1.86168766312162
H   -0.20183032566211     -4.50719771667908     -0.77276343751435
C   2.25451361267966     -3.52590918618132      0.26518465052604
H   2.18091837403494     -2.77351090210118      2.29868315837071
H   2.34281782672556     -1.53031514675090      1.07343752726159
H   1.78289082956772     -2.27533884243214     -1.41809183789161
```

```
H   1.92418541946569     -3.97679302723816     -1.82587481690242
H   2.00539372036631     -4.52875136916164      0.62762596572252
H   3.33781947521664     -3.50370736710191      0.12938104122223
C   -4.25426282269012     2.19141729759162      0.27046308987373
H   -4.32184040848699     0.05187267762921      0.59526550466930
H   -4.84152616081403     0.54712076415377     -0.99664164871739
C   -3.54138697290445     3.19446472463044     -0.63280387014650
H   -1.56128946886711     3.58571724498431     -1.42389915770589
H   -1.58647387358768     3.19323366219245      0.28729527312318
H   -3.85521907231827     2.28014778386348      1.28136801197851
H   -5.31774752550571     2.43181758475075      0.32960033514513
H   -3.94856845034378     3.13301385711769     -1.64723711731524
H   -3.73336318916005     4.21052580255272     -0.28201058196182
H   -3.91966613869511    -2.32404997322709      0.42147914113836
H   -3.20995574216860    -3.76314049824679     -0.27388537418905
H   -2.52562685289111    -2.62651810507815     -2.25152148494937
H   -4.02843483557186    -1.82272274419075     -1.88790105297798
C   4.36531621702460     -0.53066939602111     -1.18740356809438
O   3.65896712440026      0.18249222038678     -0.29991965320767
O   0.96637609169449      0.25882586845206      0.42765518270214
C   2.81947974783635      1.22994312986392     -0.76339816284566
C   1.70373907479739      1.42558641051687      0.26488787023887
C   2.25457448521708      1.90970345663977      1.60267030336292
H   1.07326754632024      2.23430325997136     -0.13913149086578
C   3.60925018656962      2.52491907483860     -0.93848973862021
H   2.36839899333884      0.93245556794133     -1.71878893330495
C   4.15672392291008      3.04504459312701      0.38707369522737
H   4.41573414382823      2.38041420266068     -1.66069322357648
H   2.93999387196931      3.27037779677153     -1.37989456274958
C   3.04818084234386      3.20047984108317      1.42227488081758
H   2.88619240631745      1.13536873757898      2.04732499964277
H   1.41968325010259      2.06881730338516      2.28788609169254
H   4.92163415434488      2.36144996331660      0.76606879737154
H   4.65667872155796      4.00237117597293      0.22353657586301
H   3.46629204269090      3.51522465827909      2.38109693381043
H   2.36840973973512      4.00013607613042      1.10766058202151
H   5.06417804201914     -1.08415176002682     -3.06064952589523
H   3.93687154434958      0.37306794646775     -3.07885127165709
H   4.91728992492068     -1.31677127040492     -0.68303816105179
```

TS-A

```
69
Coordinates from ORCA-job sp
C   0.98494561781273     -3.24589721972231      0.63467784717140
Cl  -1.14009635903365    -2.89382425157300     -1.33565714257402
Ti  0.09930631492574     -1.22703145942644      0.11228200427849
C   -1.36482857143717    -1.53372876044762      1.95627112187874
C   -0.56034415696998    -0.38693075316457      2.17247812497697
H   0.16703482943766     -0.25214899302705      2.95713315732510
C   -0.91169790773783     0.58152723431168      1.21570604397703
C   -0.29867820876867     1.93456481782709      1.04365454440189
C   -1.98260844204118     0.05172286358584      0.43938843811226
C   -2.86343011523902     0.76073542636401     -0.54595088414430
C   -2.28093859944443    -1.22787235728199      0.93202956416304
H   -1.37429262000103    -2.43033715545417      2.55515908217418
C   -3.48882902453270    -2.00563557178516      0.53006733446357
C   1.10478790969213     -1.11030042797733     -2.09733914683566
C   2.19499467771806     -0.94088747913000     -1.24260356277082
C   1.97468005899704      0.23849805934379     -0.48005058946928
C   0.75302867931853      0.80625287093320     -0.90101546320473
C   0.19870729140157     -0.05212843211730     -1.87887236622611
H   -0.73149174895577     0.10155743675940     -2.40495172733639
```

H	0.97407803417175	-1.92722455525944	-2.78816928660104
C	3.51187171097100	-1.64865528473915	-1.26875038773391
C	3.05939925815981	0.91312155216464	0.30445571887784
C	0.17253630369890	2.09388192080344	-0.40417237051215
C	4.36896585485307	0.11514518106978	0.30687093269738
H	2.75058720598495	1.13434944644886	1.32782525655175
H	3.23876854833972	1.88743213414014	-0.16400087577579
C	4.61848944765515	-0.62605957392038	-1.00265712269395
H	3.65106820396227	-2.12231198213805	-2.24267421745725
H	3.57612022286617	-2.44723763419246	-0.52631212775348
H	4.35589381563117	-0.61636974169179	1.11379954341260
H	5.19656977193333	0.79620599340219	0.51666528748306
H	4.66891405359866	0.08434749959774	-1.83444688808033
H	5.58729617248502	-1.12884933789516	-0.96280394513986
C	-4.26229828171731	0.14317584602725	-0.53661374515752
H	-2.46405258928030	0.70903790443514	-1.56158967020520
H	-2.92229822035238	1.82411642180815	-0.29586026912845
C	-4.19237800957831	-1.37418742076875	-0.66800120615940
H	-4.16941247493217	-2.01167265230852	1.38951773597201
H	-3.23233772195859	-3.04477027631489	0.32517795949493
H	-4.85377746621592	0.56937741285129	-1.34979636087777
H	-4.77244108476703	0.40962221662548	0.39499671204927
H	-5.19678269263405	-1.79193261505918	-0.76598645418886
H	-3.64689906314452	-1.63524386089979	-1.57763447537287
H	-0.66635632676801	2.38020500788637	-1.04080337440568
H	0.90598174993637	2.90166923287453	-0.48471846005345
H	0.54187297527843	2.04212644068956	1.73275438789052
H	-1.01489647765466	2.72069199453125	1.30235984836680
C	1.61539068115634	-2.68114120155458	1.75709226747406
O	2.95738029385528	-2.57841365799077	1.88795067976878
H	1.61556559305563	-3.51403689270636	-0.20366149377585
H	0.17215497703482	-3.93084696434892	0.83219668317963
O	1.04849004088391	-2.80877044509062	2.97879765315617
H	1.29737228704697	-1.16471085952012	1.37595566645591
C	3.24369639101693	-2.31027708009637	3.27199736438875
C	2.09713678906219	-3.06128845442142	3.92584225333798
C	1.71575937970969	-2.64070291658623	5.32711355730989
H	2.30465476075148	-4.13799473031632	3.91631910027307
C	3.25957416486333	-0.81799408445004	3.58308871671897
H	4.20854778714260	-2.76606246056695	3.49741244513818
C	3.06614408283631	-0.55562242969892	5.07312801416815
H	2.45972507481857	-0.31623608415170	3.03038829982605
H	4.19707206449721	-0.38060664407759	3.23753855946240
C	1.74256851558429	-1.13524815157224	5.55267635792146
H	2.44227140895567	-3.11097914504937	5.99624244281232
H	0.74605416201071	-3.07333019405789	5.58237913447381
H	3.88989835121841	-0.99936232394768	5.64290536984670
H	3.10773337922594	0.51927090906138	5.25618771702975
H	1.59316518883168	-0.92958341871826	6.61406088044034
H	0.91277208077731	-0.65410988832518	5.02709980473268

TS-iso-A

```
69
Coordinates from ORCA-job sp
  C   1.63473604787127     -1.41630509930119      2.13594051179272
  Cl  -1.21789110728474     -2.59787542218083     -1.14058807064744
  Ti  0.14197937580783     -0.94158167652549      0.33672613385824
  C   -1.22232570872622     -0.69040997464280      2.26644219995675
  C   -0.50038443026949      0.50721191687995      2.02748092844702
  H   0.27280806458009      0.93126399049881      2.65101064701644
  C   -0.98691005813150      1.08487308347571      0.84338867221657
  C   -0.51772286814684      2.36221359249112      0.22233925130339
  C   -1.99134122925284      0.22789653681898      0.33122693511493
  C   -2.92213134526368      0.44187653379404     -0.81870656351965
  C   -2.16763717251959     -0.83613970422498      1.24208421039419
  H   -1.10112575260514     -1.36010320169606      3.10524815488312
  C   -3.29728547190308     -1.81271831631819      1.17030549189346
  C   1.56466552820835     -1.31727972166347     -1.57110726700263
  C   2.47811258334316     -0.73558129137058     -0.68513749735725
  C   2.00653803649783      0.55843523317254     -0.34969094151235
  C   0.86231100113506      0.81927735218746     -1.14132742788234
  C   0.57418414740473     -0.34406015113496     -1.87000139661024
  H   -0.24958509965049     -0.45949762576215     -2.55647597802086
  H   1.63636346684835     -2.30707438816437     -1.99457489470236
  C   3.85160138991566     -1.20745123145870     -0.31517992195715
  C   2.78967716254701      1.53139229688636      0.48128054805248
  C   0.06541973833992      2.08378172241512     -1.16357564077637
  C   4.14020833113217      0.96073297948008      0.93695406713397
  H   2.22671247228135      1.86453488294737      1.35759468052578
  H   2.96394869547714      2.43265908887278     -0.11615721971715
  C   4.75325523593676      0.00668234259418     -0.08284799870560
  H   4.24835095763852     -1.82490839380087     -1.12401200465552
  H   3.86626619556856     -1.84223725146192      0.57302882496348
  H   4.02643967500082      0.43457213597163      1.88880595710276
  H   4.82564621462754      1.78652122302782      1.13721003849709
  H   4.91601646124868      0.52966373012132     -1.03045332241776
  H   5.73480171022227     -0.32433196544125      0.26313161735068
  C   -4.30196472274216     -0.10259834249574     -0.44177839665123
  H   -2.56433839630784     -0.07588308275304     -1.71324649502904
  H   -2.98702697136050      1.50437090615038     -1.06639216027033
  C   -4.22384715079110     -1.57054240880929     -0.02929329400688
  H   -3.86979821991519     -1.70991682273505      2.09811742865697
  H   -2.92366223989007     -2.83739697275895      1.15171181030520
  H   -4.98531361856069      0.00380401692157     -1.28737153972656
  H   -4.71636998290305      0.49948238930789      0.37373949942468
  H   -5.22296358172385     -1.94142367186277      0.21041102088591
  H   -3.86482440835858     -2.14969859608446     -0.88008760709661
  H   -0.73998398761574      1.97636084963958     -1.89279576671546
  H   0.67620267036045      2.92593084346924     -1.50231981507504
  H   0.23251175121041      2.82203904792653      0.86746366256024
  H   -1.34084089837445      3.07974303812395      0.15418428372799
  C   1.41360240915901     -2.79415471622258      2.03454120574019
  O   0.68985286448463     -3.46973580376446      2.91738310166010
  H   1.27685080146921     -0.97813419488502      3.05661783933347
  H   2.62601198362676     -1.09218001518274      1.86938520687400
  O   2.15485405523711     -3.60160914613772      1.29076817554804
  H   0.09459191603817     -2.53621216199357      0.81043536408811
  C   0.61167964551211     -4.84235153305733      2.47174531125802
  C   1.93294535868934     -4.96265303237496      1.71231405322172
  C   1.95369657468865     -5.91851226587646      0.54147992911111
  H   2.74079717245025     -5.21130138068126      2.40800477146906
  C   -0.66191954282021     -5.06238697521162      1.66126246966479
  H   0.62366379296339     -5.46974056941268      3.36287417521815
```

C	-0.52130703194918	-6.17456319605566	0.62802651138340
H	-0.91827643508579	-4.14671702374063	1.12297185047707
H	-1.48413233240052	-5.26777156109113	2.35003200704085
C	0.67154243958299	-5.90084560786389	-0.27771732344267
H	2.10250075640502	-6.92048023870121	0.95534328592474
H	2.83413266357281	-5.71406117671920	-0.07192811438440
H	-0.40389724955805	-7.14928646422597	1.11401597570198
H	-1.44294884242728	-6.22650884206357	0.04592958835428
H	0.73814795020153	-6.65305796246987	-1.06622911709176
H	0.53732275925282	-4.93167105279449	-0.76723042315889

A

69

Coordinates from ORCA-job sp_pw6b95

C	1.31900030617780	-2.20389596784410	0.35165199335507
Cl	-0.78946471079557	-1.64925984177367	-1.96411924101158
Ti	0.31683822941094	-0.39070952105209	-0.32922053100657
C	-0.69721668352512	-0.73761752730196	1.76602408369476
C	-0.11743989484957	0.54684512842313	1.72042871909941
H	0.70139322096972	0.89082674904707	2.33566625186350
C	-0.84246904238178	1.33652734620053	0.79742556627295
C	-0.56585383273136	2.76500274285625	0.44557729588159
C	-1.83928983156541	0.51327429028642	0.23928686839603
C	-2.92571758985038	0.88078048963399	-0.71950065646995
C	-1.76182687749231	-0.76410314523771	0.84800447760872
H	-0.39333250859467	-1.54002738833819	2.41450839529556
C	-2.78645135437437	-1.83416603515934	0.63814404436319
C	1.97518107528756	-0.17743379518611	-2.04758476057177
C	2.67531691152614	0.11932776225465	-0.86421396655858
C	2.07678015264190	1.26196073223675	-0.28253229276785
C	1.03218903009971	1.69949107298744	-1.14225142833137
C	0.98545311541372	0.80982054455096	-2.22892660709937
H	0.27766562602121	0.86047648062552	-3.04337054501731
H	2.16880299890286	-1.00998903880982	-2.70786077071654
C	3.92838107523667	-0.51503574122652	-0.34321331550475
C	2.71094351160722	1.97975212325752	0.86975632406529
C	0.10830555759174	2.85810947832579	-0.92743379673751
C	3.70678349753208	1.08198741864941	1.59824184880758
H	1.98279845015408	2.39230641113426	1.56823186282035
H	3.24638562759183	2.84782330417464	0.46739581839075
C	4.66513283070428	0.42528746193571	0.61059319639880
H	4.57136247621122	-0.78310273804998	-1.18456390205083
H	3.70796865875412	-1.45326384174679	0.17065741082763
H	3.17127196376954	0.30986164291229	2.16263859142970
H	4.26189151547895	1.67383014939113	2.32838700972811
H	5.17533150727075	1.20619006638326	0.03815697131966
H	5.44110424315212	-0.12991254250417	1.14056797285313
C	-4.16731383102955	0.04412958758391	-0.40494075237315
H	-2.61623286067288	0.69856671764831	-1.75249715630374
H	-3.14868536437990	1.94806464809082	-0.64260576724618
C	-3.84118929827702	-1.44691481741463	-0.40594057029847
H	-3.27529475314915	-2.00556379129658	1.60239360849115
H	-2.31894254429961	-2.78176835875503	0.36740077069192
H	-4.94818453559773	0.25210810576185	-1.13943702187564
H	-4.56621772120130	0.34213145190715	0.57015918785289
H	-4.74879254984564	-2.02673331159044	-0.22541367846095
H	-3.48561561727423	-1.72070108106327	-1.39961178237205
H	-0.65432614967484	2.83898429206405	-1.70941322059454
H	0.63783303195047	3.80918021392844	-1.03174743629133
H	0.06730461318183	3.20728624742034	1.21500655685161
H	-1.49138403011413	3.34602276160568	0.45020002508685
C	0.58567352776994	-3.51287003830716	0.55622242638910

O	-0.05058251409351	-3.59825763338762	1.81219371090323
H	1.94337820785194	-1.99218389541102	1.22976085295621
H	2.00058667680017	-2.39906436856034	-0.48088622235137
O	1.48749653722186	-4.62248965281015	0.51106162782078
H	-0.13923416101854	-3.67221142216108	-0.25303025595064
C	0.07071603965539	-4.93418441301528	2.26286482378452
C	1.46982885706618	-5.27515369618754	1.76473169075146
C	1.77615405939614	-6.75518849143479	1.62977391098146
H	2.21133934196047	-4.82055009360419	2.43927235089235
C	-1.03153412424195	-5.82667987845944	1.69344615438802
H	0.02328670746891	-4.93099821908659	3.35554688614353
C	-0.65957250180211	-7.30342190590595	1.76231322320348
H	-1.21647694256039	-5.56522775795017	0.64649709582995
H	-1.96290727316619	-5.62996581373050	2.22956459967276
C	0.63895260515952	-7.55381718280491	1.00505219072961
H	1.96743129601470	-7.13921942107317	2.63700357244128
H	2.70827304738297	-6.88583028913478	1.07430798900130
H	-0.54066227544001	-7.61653981527202	2.80589581633144
H	-1.47073435742317	-7.90750155451010	1.35022455986997
H	0.89117203579024	-8.61643262655620	1.01499744755487
H	0.51043176524548	-7.27159926756331	-0.04418890312968

TS-Da

69

Coordinates from ORCA-job sp

C	1.39932646375934	-1.80779862359317	2.03448725975177
Cl	-1.40497966219453	-2.03881480118692	-1.98182373847242
Ti	0.02171928442611	-1.04858531848031	-0.01375877200408
C	-1.54198169159800	-1.08523012591051	1.77017880689576
C	-0.72090640987011	0.07013579823920	1.89061503957469
H	-0.00991382333253	0.27706700233492	2.67521384995350
C	-1.06507568362836	0.95902740966291	0.86575524841695
C	-0.47017920355006	2.30734994462206	0.63063507103961
C	-2.04953120067955	0.33499641969721	0.06840058557579
C	-2.85709613470377	0.91575086259360	-1.04564144170380
C	-2.38927358578325	-0.90016796877850	0.67383563266105
H	-1.56824713842316	-1.91846526364081	2.45389004559655
C	-3.60020307822141	-1.69390551069180	0.30188829584497
C	1.54610979656809	-0.97488403854053	-1.86903390357530
C	2.41011417529392	-0.68872283379553	-0.80977209003434
C	1.95974988690981	0.50718209412144	-0.18714276649076
C	0.89952536069286	1.02308343181398	-0.96162243388296
C	0.60935954351388	0.08663567112260	-1.96360401277633
H	-0.15770274420763	0.19580446961234	-2.71231357788494
H	1.59497960411290	-1.83373012851997	-2.52005930198881
C	3.77340347343868	-1.24768686750608	-0.53435797714466
C	2.72756044096557	1.21747137915420	0.88562644033140
C	0.19838788011981	2.32186848101788	-0.74195797514117
C	4.10061668043030	0.57740338406558	1.15008894684001
H	2.16200717802636	1.26717772984932	1.81991743559919
H	2.86712939284098	2.25620995379402	0.56917200073355
C	4.68255803881240	-0.10272110292726	-0.08341143063176
H	4.15738995698516	-1.72039991775148	-1.44025906753461
H	3.79849665045232	-2.01724315061766	0.23786175024683
H	4.01978907333035	-0.16757562734038	1.94650101794195
H	4.78307660032410	1.34228903397091	1.52429259384737
H	4.79767656241486	0.62195304057154	-0.89531809749063
H	5.67843213824684	-0.49006754821087	0.13984831394463
C	-4.30656494006220	0.44617931142106	-0.90058213003846
H	-2.46571300952633	0.59648004805461	-2.01548735152102
H	-2.80764678247125	2.00695118039154	-1.02116735229940
C	-4.39592176062366	-1.07622442235999	-0.85531326533736

H	-4.23092268531127	-1.73610266479037	1.19594027300272
H	-3.34096080294953	-2.72561967121868	0.06085551631044
H	-4.90125420485122	0.82358888309525	-1.73549234166634
H	-4.73562532621994	0.87780618646990	0.00985736567865
H	-5.44064885961773	-1.38344191264219	-0.77002181548847
H	-4.02034272907644	-1.47514050625513	-1.79676672133327
H	-0.54633112391690	2.46289802168241	-1.52719886084780
H	0.90279124996634	3.15501232764286	-0.82372772617036
H	0.26207294022113	2.51938110775699	1.41092031307669
H	-1.23702952032568	3.08350609423179	0.70129513935238
C	1.67396934066556	-3.04614753913533	1.46057880556710
O	0.29311271964161	-3.01034302048338	-0.00480297308112
H	0.74896912248008	-1.79956880859871	2.89987229907520
H	2.23229732518627	-1.12131911905867	2.09624212042513
O	1.23227391902040	-4.23980540213234	1.86948193367551
H	2.56086580706965	-3.15757758284418	0.84744724843824
C	-0.51905314243885	-4.07004388434477	0.35154356601141
C	-0.18612037584783	-4.39071874625836	1.82232084240316
C	-0.59077678065114	-5.77317868696480	2.29295560070195
H	-0.65062125915286	-3.65488440667342	2.48818393691920
C	-0.24805050135829	-5.24910143708910	-0.58122218305096
H	-1.58672487909852	-3.81720752756380	0.29718796123345
C	-0.82814504060407	-6.55967426709660	-0.06806337747836
H	0.83394416631542	-5.36287953597795	-0.70400982270693
H	-0.64908526968415	-4.99112319623918	-1.56334070745750
C	-0.26138786464398	-6.88713015314397	1.30750244809812
H	-1.67468822113240	-5.74357614767422	2.44157585168823
H	-0.15801605520635	-5.96679140209012	3.27778051193748
H	-1.92004085338218	-6.49068356970547	-0.00558772796813
H	-0.61223984068788	-7.36484146946745	-0.77408840593150
H	-0.66986522632526	-7.82900199559354	1.68036696639306
H	0.82134663912724	-7.02614136409628	1.23603131434979

TS-Db

69

Coordinates from ORCA-job sp

C	1.52230802151349	-1.72007357297568	1.84474955523352
Cl	-1.17804117594760	-1.85472714351906	-2.35289226729358
Ti	0.12979983646847	-1.03193205807199	-0.13541565268711
C	-1.41911887958860	-1.19318121657846	1.64590051429635
C	-0.72725627675373	0.03813523532978	1.77952938374885
H	-0.06011827852957	0.32538439471243	2.57703503496010
C	-1.12914177080380	0.88906337123923	0.74446089087339
C	-0.63561440652461	2.28559886070168	0.56024821266444
C	-2.03024214484991	0.17279146763958	-0.07213091656365
C	-2.90832282103000	0.67955517974314	-1.16796942306167
C	-2.23914509937366	-1.10128435956074	0.51830381203767
H	-1.36678781542116	-2.03406389684303	2.32108112826975
C	-3.28317016141268	-2.06933361182137	0.06880223333505
C	1.68474416560534	-0.81863332419547	-1.92859319104820
C	2.49296213464387	-0.59745678043511	-0.81124712587632
C	2.01241756632602	0.56286034036317	-0.14174147114215
C	0.97620574626179	1.11201100878487	-0.92703302161849
C	0.74806202808681	0.24192703612309	-1.99983047475165
H	0.01432927030221	0.39696994519473	-2.77204167900964
H	1.78449574541972	-1.62428104541716	-2.63954999656466
C	3.81298564264641	-1.21703085771394	-0.48051048308798
C	2.73974218334573	1.22051837375200	0.99069062486235
C	0.21783274400755	2.37945241378984	-0.70370752865061
C	4.10897098746511	0.57514448596733	1.28221291925551
H	2.13979050069125	1.23194799493150	1.90385588418955
H	2.88764966121010	2.27123597298959	0.72167766499690

```
  C   4.72695568333597     -0.11648879637164      0.06930068126376
  H   4.23991017242250     -1.65516200505309     -1.38492087152546
  H   3.73661290194646     -2.03250248012914      0.24073504689595
  H   4.01758575826350     -0.14993479317579      2.09412439778384
  H   4.78783456895256      1.34481979023178      1.65383530836528
  H   4.91491923229934      0.61847758394993     -0.71909275165062
  H   5.69495914426336     -0.54072574878875      0.34318382756543
  C   -4.28789699367222      0.03044613913152     -1.01317307765138
  H   -2.49625841465240      0.43367458283779     -2.14858300778156
  H   -2.99308092957721      1.76835194652233     -1.11010900956409
  C   -4.20429685407687     -1.49514606657520     -1.02125701137956
  H   -3.87287432888576     -2.34160146527790      0.94918410607944
  H   -2.81034516445383     -2.98771697587460     -0.28487122992805
  H   -4.94475824812218      0.36214221209320     -1.82056942611039
  H   -4.74299463709626      0.37763857193949     -0.07944613319988
  H   -5.20623005665447     -1.91338440711318     -0.90151522461105
  H   -3.83828576742013     -1.81464740403254     -1.99535835207328
  H   -0.42279999795396      2.54887184355017     -1.57066481015190
  H   0.90031149021802      3.23183478970825     -0.64654469184889
  H   -0.05343716642587      2.57179999702569      1.43766065718989
  H   -1.47268600053663      2.98670529137477      0.50758562202673
  C   1.20374538834183     -3.04709917846490      1.57913470385240
  O   2.12104629524246     -3.87705457747676      1.09224595979095
  H   1.08603658760602     -1.31725464385444      2.74615830846350
  H   2.54645488343294     -1.42697297628446      1.68797784760353
  O   0.15962182590527     -3.04206716456564     -0.11392781253902
  H   0.35118460764262     -3.48917552903504      2.08737538751727
  C   1.57418313566061     -4.86715336022493      0.21798792746862
  C   0.81099974534389     -4.03649926983290     -0.83435688108317
  C   -0.09563933390635     -4.94080645743945     -1.67193672488120
  H   1.55613104275508     -3.60175114698018     -1.51557799961442
  C   0.73734534982319     -5.89331206018888      0.97846864337652
  H   2.42898197295952     -5.36856073864291     -0.24159962575095
  C   -0.08306643947061     -6.76338407255298      0.03376351196345
  H   0.04004239727465     -5.40385033957594      1.66278056382422
  H   1.40152934568583     -6.50399681647877      1.59465297816873
  C   -0.95840555936162     -5.88598915587246     -0.85021762349653
  H   0.56146630928922     -5.53801668551402     -2.31584444874618
  H   -0.69871527977363     -4.31360069650237     -2.32389380837499
  H   0.57932634465397     -7.36934909855585     -0.59371190648642
  H   -0.68461350102624     -7.46417569028442      0.61653864855323
  H   -1.56655571258291     -6.50153850557269     -1.51703726447446
  H   -1.65659120142832     -5.31510265620205     -0.22965906219693
```

TS-Dc

```
69
Coordinates from ORCA-job sp
  C   1.33736369476368     -2.85960885977666      0.11049609073398
  Cl  -0.91621583032293     -1.92577990974214     -1.42926906797066
  Ti  0.89778900319884     -0.52164120634372     -0.03214106688862
  C   -0.06968636542656     -0.64625563176842      2.14265400858864
  C   0.54916162477796      0.61551363265132      1.98489794914963
  H   1.42078326720068      0.98319799571049      2.49853784416892
  C   -0.16759799094207      1.34862675384468      1.03350559167764
  C   0.16543349663127      2.73705203784111      0.58913756251767
  C   -1.24802993598403      0.53961912711294      0.59541636248884
  C   -2.42403996965142      0.90715336752668     -0.24933639915188
  C   -1.21323314606960     -0.66601609977327      1.32678210205693
  H   0.20975093137893     -1.41769959913267      2.84490151384068
  C   -2.33241679928251     -1.65492830381988      1.36029435061692
  C   1.54977902335171     -0.50674350703101     -2.33018648079619
  C   2.72262373971677     -0.08199165404890     -1.70390383133288
```

```
  C    2.45772224526856      1.14144655626949     -1.04432298996212
  C    1.12898584650336      1.51223984501553     -1.33582595384432
  C    0.55950216189148      0.48161110150055     -2.10161338376193
  H   -0.44366128304506      0.48362617644291     -2.49522451729068
  H    1.43072118145111     -1.40659798980091     -2.91261117363528
  C    4.09173800533724     -0.65720101168924     -1.79014796177734
  C    3.51581590155132      1.96099023205819     -0.37786181712841
  C    0.42060435032748      2.75918681501911     -0.91628709123574
  C    4.93081200043576      1.37572788635449     -0.54547226587419
  H    3.29708905505401      2.10144653306146      0.68359245425043
  H    3.48637575284101      2.96370351976105     -0.81632971117204
  C    5.10039151352101      0.49112495208453     -1.77704673290563
  H    4.19001736194881     -1.24783017259354     -2.70338751223208
  H    4.28264297111098     -1.33732380561207     -0.95984335010756
  H    5.20234038697311      0.79272920483956      0.33513902642648
  H    5.64925805862733      2.19699658309914     -0.58316019158603
  H    4.97880176885065      1.08817446434881     -2.68639177480235
  H    6.11773430285577      0.09442019058512     -1.80036551681552
  C   -3.67948505285635      0.26875988581043      0.35113819070666
  H   -2.29826485195728      0.55355494081811     -1.27524883510245
  H   -2.53650817248974      1.99309455845831     -0.29435076871115
  C   -3.52621420231770     -1.24407920052162      0.48718594432017
  H   -2.65187120116853     -1.72804588511640      2.40520993340567
  H   -1.98281307281330     -2.64883337395282      1.07593870176249
  H   -4.54226303465227      0.49333303687875     -0.28010832700359
  H   -3.88107167969672      0.71983531061976      1.32842204451895
  H   -4.43942810120944     -1.67161725977080      0.90670147059687
  H   -3.40171061623449     -1.66881670061019     -0.50792335032369
  H   -0.52315744287119      2.82253009942966     -1.45977619347475
  H    0.99597687938180      3.64467322788284     -1.19927841318440
  H    1.05530898451720      3.07008584983363      1.12686929841200
  H   -0.63596309924360      3.43150425056611      0.85752711854876
  C    2.27968116874534     -2.67826460842768      1.11040991623234
  O    2.66384432262996     -0.73106234151029      1.05091270379338
  H    1.67706209678907     -3.16555883998217     -0.87219780418976
  H    0.38162694067384     -3.25165410108740      0.42894158880130
  O    3.56964190294556     -2.95395404802768      0.94707559415181
  H    1.91669848447744     -2.64726408085746      2.13574196043798
  C    3.66143424961746     -0.80128215811430      2.00481817884450
  C    4.47550470231838     -2.07041962049572      1.62986566750717
  C    5.24017151441526     -2.76237980388459      2.74776045709030
  H    5.20034051223120     -1.79482837250348      0.85892558851281
  C    3.16290962515104     -0.77216146816808      3.46438501897441
  H    4.36048215801113      0.04058569360183      1.90758835092269
  C    4.17255474349816     -1.32587661697116      4.46117341015019
  H    2.24272375973073     -1.35053436850706      3.57549568274153
  H    2.90619915398109      0.25634822404587      3.72721392455505
  C    4.53380207710888     -2.75715835310417      4.09382419578080
  H    6.18983263067979     -2.22998754692706      2.86153626314161
  H    5.50256293821868     -3.77341252190700      2.42713903337492
  H    5.07992289176831     -0.71200383636791      4.46800492153713
  H    3.75352152124887     -1.27205859380620      5.46808630866147
  H    5.18531998571944     -3.20293644537310      4.84828988329318
  H    3.62858095880656     -3.37343415594547      4.06371627496800
```

TS-Dd

```
69
Coordinates from ORCA-job sp
  C    1.05718513309597     -2.82745403472595      0.26404312812556
  Cl  -0.94323290060468     -1.83464565094538     -1.49557206926710
  Ti   0.76129672264365     -0.49737418286482      0.05769437206055
  C   -0.36223076481338     -0.46975323968054      2.12455500491102
```

C	0.25575948823785	0.78869947136219	1.89968964756813
H	1.08500153973691	1.21341346576693	2.44486430136580
C	-0.43365604553717	1.43736207418606	0.86337837074321
C	-0.12564081767034	2.78866980456411	0.30354067358322
C	-1.46103758360938	0.57390221753727	0.42580062089194
C	-2.59136941544062	0.85736052847892	-0.50707899904878
C	-1.43937112757655	-0.58332740781426	1.23858008202356
H	-0.11065775066325	-1.19858779438093	2.88090693363260
C	-2.51784350819527	-1.61780542958062	1.24692744832874
C	1.89066964331404	-0.60255015817451	-2.03350259478850
C	2.86419238392555	-0.03244305066487	-1.20176711957093
C	2.37134548234797	1.20192029206506	-0.72758873355307
C	1.12218098228171	1.43977236449130	-1.34622907894520
C	0.82472827858966	0.32741823057633	-2.14309944098807
H	-0.05910998524689	0.22043807421004	-2.74744174714975
H	1.96968999613215	-1.54214442970557	-2.55944783404465
C	4.27023659557616	-0.44543827535079	-0.94724863017080
C	3.19588502794393	2.18263577964637	0.04121188534469
C	0.27183668002367	2.65476387393696	-1.16643371266943
C	4.70007147739607	1.83850490116391	0.04727440236365
H	2.85131679667218	2.27720450010917	1.07439592837654
H	3.04609851153684	3.16999926405431	-0.40584425086907
C	5.13549850305696	0.81736523144296	-1.00224821252709
H	4.59763695795634	-1.18087029155917	-1.68452912763148
H	4.36568773468852	-0.89948440576123	0.04047786173266
H	4.96570699142119	1.45167421430160	1.02897964434309
H	5.27789163997598	2.75592538173597	-0.07871079059063
H	5.07960487285568	1.25191885362538	-2.00494711618749
H	6.18312244642828	0.55848713392305	-0.83194160270631
C	-3.85965494320415	0.20522635492516	0.05243552593913
H	-2.38420424773353	0.46487539720943	-1.50507057164249
H	-2.73737701960601	1.93572521253022	-0.60817690065691
C	-3.67165931457059	-1.29184669114233	0.28614730470261
H	-2.89804566426691	-1.67174389813384	2.27199849724763
H	-2.11184007558031	-2.60575501153795	1.01951822405185
H	-4.69032827257756	0.36467989368580	-0.63876209425538
H	-4.13286030059325	0.70268393615318	0.98898761675888
H	-4.59534846521186	-1.72130099097691	0.68004556576374
H	-3.48182032031268	-1.77032308248595	-0.67336057001949
H	-0.61614628882751	2.57033416570070	-1.79505200715264
H	0.80254534311865	3.54933957915552	-1.50488354721801
H	0.68629691599962	3.23799155917655	0.87674677900478
H	-0.98618621913222	3.45392381176612	0.41388171768578
C	2.21298174085802	-2.74783908042056	1.02505085712123
O	2.19103330360347	-3.06220807995624	2.31425171042437
H	1.18942737924410	-3.15746354814844	-0.75621585742385
H	0.15114673968802	-3.16601173086481	0.75426347959819
O	2.45823747734402	-0.77221058786886	1.40745739962657
H	3.17324995207276	-2.75675165721156	0.51541405483519
C	3.09129044704503	-2.28702633217473	3.10151865296903
C	2.68797276356411	-0.83428777605801	2.77150435992178
C	3.73886943317017	0.15144586592105	3.29909975421251
H	1.77958332711645	-0.63426020963034	3.35616294959950
C	4.55079129459671	-2.63576810467107	2.84243451812121
H	2.85307755361070	-2.51904902161730	4.14233347139769
C	5.46472627989827	-1.65089868404848	3.55971515190045
H	4.78750174601413	-2.59730045758688	1.77576023529020
H	4.73922215461110	-3.66023444943084	3.17183674466808
C	5.18924881859560	-0.24428236365420	3.04968761664624
H	3.58986726353754	0.22652966404075	4.38226319812356
H	3.52570830293673	1.14674625373369	2.90030188121848
H	5.30090282644916	-1.70028850231391	4.64177868047268

H	6.50820842911120	-1.92451402349306	3.39143373062966
H	5.85112062037858	0.47936788970802	3.53140578953350
H	5.42178703257190	-0.21191260624916	1.98195683621716

TS-De
69
Coordinates from ORCA-job sp

C	1.09285157093172	-2.79863810010794	0.31553112751592
Cl	-1.01671149640315	-1.84716103222762	-1.38188664038699
Ti	0.74732046726159	-0.52935012580328	0.07729833692888
C	-0.35047092336274	-0.37103637466470	2.18233459431090
C	0.29365956560289	0.85663399162842	1.87915207437255
H	1.14779790822863	1.28362926374587	2.38319412478292
C	-0.41483553033433	1.47465182089498	0.83579641460511
C	-0.09956344353753	2.79444420861565	0.21123287694083
C	-1.46760085391614	0.61432226280506	0.46397122806991
C	-2.61525959887279	0.88015690267594	-0.45251579849722
C	-1.44562851006137	-0.50843412239171	1.32477427350837
H	-0.11112295294631	-1.04857142432838	2.98699749215051
C	-2.53723129695842	-1.52715562104477	1.39647593595873
C	1.91751041112040	-0.69805794532333	-2.00964340819393
C	2.89902635894669	-0.09588118547088	-1.20507434579094
C	2.39462637464492	1.13820213284147	-0.74730301828208
C	1.14213927709007	1.35618301327192	-1.37234062321650
C	0.85002345706977	0.22500841989344	-2.14485132124179
H	-0.03137365988627	0.10189197214571	-2.75014067398801
H	2.00295589983773	-1.64743064000661	-2.51766968941668
C	4.31670127487188	-0.50201657583924	-0.97461755976299
C	3.21036610328619	2.13255964731461	0.01940105417641
C	0.30043273780615	2.58437668830769	-1.24842685455900
C	4.64630115650101	1.65284712160610	0.26425123377227
H	2.74666257115762	2.39106159468648	0.97465408856083
H	3.22959870417902	3.06342961326306	-0.55835381428361
C	5.17516458967719	0.75752485327432	-0.84985217244540
H	4.66373489241595	-1.12792820491854	-1.79994086533510
H	4.42812548900471	-1.08707516035998	-0.05991922952487
H	4.68221254596452	1.09255942640994	1.19742937627820
H	5.29694884813236	2.51971944549953	0.39393769091005
H	5.18162062441960	1.29810735823608	-1.80177655473697
H	6.20982413614384	0.47803523566957	-0.64031933170418
C	-3.87951355881309	0.25954390151063	0.14955780654751
H	-2.43031887785286	0.45847432760794	-1.44312584010194
H	-2.75496568141353	1.95674670189631	-0.58119904444489
C	-3.70234367329400	-1.22855169965856	0.44055478846347
H	-2.90254128291247	-1.53493448191875	2.42843075402500
H	-2.14593643363727	-2.52806940060302	1.20277654061237
H	-4.71935430797714	0.39943672029118	-0.53489264905969
H	-4.13616826454207	0.79435337259100	1.07029858019811
H	-4.62423357148855	-1.63144254593439	0.86577683610287
H	-3.53344298101497	-1.74875299385944	-0.50086231551450
H	-0.58733106448816	2.47727184183166	-1.87439088225586
H	0.83953026087076	3.45653178635784	-1.62982462162875
H	0.71159439175782	3.26921911686005	0.76460153683493
H	-0.95760430414989	3.46775440345342	0.28485177314008
C	2.43111660204328	-2.78457596071245	0.74452420014638
O	2.91380720607884	-3.19144396200169	1.90811287679830
H	0.98489139311994	-3.20071566838200	-0.68225787188564
H	0.33022241401434	-3.14073987111713	1.01090825430008
O	2.40016584959451	-0.85094655637536	1.37251565455732
H	3.19933093794860	-2.79132336421130	-0.02421878865240
C	2.27816516885745	-2.55238157060541	3.01259419203634
C	2.58849572823777	-1.06774237770532	2.72813343699296

C	4.02556314883108	-0.71367130684555	3.12217284802148
H	1.91586978304098	-0.47206310858044	3.36061627870018
C	2.79786666169173	-3.09747293183646	4.32522017426038
H	1.19904071319602	-2.73321528441574	2.96074833496092
C	4.23735622110869	-2.70377591491278	4.62714867086019
H	2.66107662621716	-4.18141079183860	4.35092583949222
H	2.14779164842588	-2.69207159171576	5.10692650669984
C	4.39887389173634	-1.19378484719284	4.51920844729739
H	4.14674457176998	0.36963090354588	3.05278798734906
H	4.71621731602338	-1.15183349487241	2.39538571374912
H	4.50641286936320	-3.05263881551337	5.62630070858512
H	4.92248569181934	-3.19682119387154	3.93140819758372
H	3.76441124533108	-0.70461138630286	5.26674576840786
H	5.42560696249021	-0.89948041526159	4.74735928534417

D

69

Coordinates from ORCA-job sp

C	-3.33792027719167	-0.71659426096585	3.21231608265594
Cl	1.26352277643053	-0.78050739051969	1.99743661172585
Ti	0.63443013458545	0.59384282598019	0.15065416476306
C	1.22707306585990	-0.25733903490227	-2.00154922659277
C	1.55581651414618	1.10743368650105	-1.88086000426637
H	1.13278515849706	1.90298692478448	-2.47594849656272
C	2.62566592746310	1.22525127702959	-0.95085427738063
C	3.26800611966195	2.50627397481947	-0.52226039974149
C	2.91409319285285	-0.06582929790701	-0.48220309360604
C	4.05579925223958	-0.48856867257961	0.38827666606643
C	2.04046848410123	-0.98342818436404	-1.12363369624484
H	0.46560464037325	-0.66290911631497	-2.65242764835102
C	2.16203603465115	-2.45850786609063	-0.92478980770789
C	-0.26936568170274	1.85748984707579	1.96006999277958
C	-1.10444707044718	2.20295952904626	0.89401903293874
C	-0.30346789921248	2.82431220212189	-0.09398660452798
C	1.01644653577858	2.95217014334075	0.41407623868519
C	1.04272275191160	2.32163165050809	1.66726688515364
H	1.90458081357399	2.22508189362737	2.31247294193001
H	-0.57162669172485	1.33428347183621	2.85443913718624
C	-2.56894660417711	1.96936026215632	0.73332943176070
C	-0.89407007852071	3.42005666217546	-1.33179323665548
C	2.18368870614605	3.56332804736437	-0.29640463136650
C	-2.24213950303089	2.77768737823387	-1.64400478348640
H	-0.21844345718855	3.34999428771727	-2.18583489203238
H	-1.03987841703948	4.49334309219109	-1.16217125582187
C	-3.14615494327078	2.79937856318002	-0.41437431596017
H	-3.08409310565846	2.19839036021112	1.66866775076962
H	-2.74333060448597	0.90884678249419	0.54638674738932
H	-2.08961926845202	1.74382335798406	-1.97040567833701
H	-2.71712646734604	3.30723178037397	-2.47213229589656
H	-3.28344726793813	3.83713773594376	-0.09330739989202
H	-4.13897741143130	2.42253501757320	-0.66820761728884
C	4.12091727898258	-2.00809188287546	0.59341341382335
H	4.02325698478028	0.01803896661570	1.35494933543933
H	4.97547555104799	-0.15039846849376	-0.10033998740806
C	3.62092397785399	-2.79686524722400	-0.61426190815602
H	1.82012861631877	-2.98967340108585	-1.81593015484858
H	1.53646291087581	-2.79415000713025	-0.09220931475094
H	3.52347595989724	-2.28672508903531	1.46228524305155
H	5.15180318024384	-2.28835019695391	0.81926406459875
H	4.24175283106529	-2.58005491347917	-1.48966641624990
H	3.71920338646392	-3.86700497836875	-0.41969719422508
H	2.58263094650741	4.40538250181897	0.27361118665694

H	1.85645841093343	3.97429737182573	-1.25375082260208
H	3.99245488037359	2.85212815985434	-1.26507295576464
H	3.82984587678334	2.34441287627311	0.39994893668928
C	-2.43663375692367	-1.28018776623800	2.41385383130121
O	-2.77101720524958	-1.91570523682216	1.27702728288983
O	-0.87439034521749	-0.34049170506379	-0.16479860736590
C	-1.72221744271916	-2.48662365567760	0.50286302999758
C	-1.33083168261664	-1.55903751805345	-0.65097782245745
C	-2.48478297278135	-1.36968187586809	-1.63067201270644
H	-0.52829501798890	-2.08853527808898	-1.18782594531825
C	-2.20809679254664	-3.82320372869681	-0.04045431824971
H	-0.84545141261562	-2.64534228690346	1.14118989212395
C	-3.35846015808923	-3.66078894780307	-1.02905704193150
H	-2.49204429147682	-4.47324132589136	0.79060414957996
H	-1.36473116036756	-4.31034405001060	-0.53995787815844
C	-2.97381597818687	-2.71490234755291	-2.16232643945082
H	-2.15035741345824	-0.73414643654402	-2.45607173758014
H	-3.30470625963042	-0.83761597297722	-1.13981065638772
H	-3.63755379007008	-4.63718938529469	-1.43112450186268
H	-4.24224423048828	-3.27678131367564	-0.51227835829921
H	-2.18429219678974	-3.17871374389330	-2.76445572208970
H	-3.82086512911413	-2.56246289802611	-2.83489077885100
H	-3.01114258750660	-0.23220250869865	4.12013136573066
H	-4.39604757605497	-0.71350115710104	2.98610578925594
H	-1.37746531098959	-1.25805699448616	2.65671385299068

TS-C$_{cat}$a

69

Coordinates from ORCA-job sp

C	2.58235897958684	-1.35484941123416	1.72871455872792
Cl	-1.48552957917434	-2.81058825509779	-2.33371737477921
Ti	0.80876429981383	-0.76752957554677	0.49905460017122
C	-0.14017175792017	-0.78024563251944	2.68736264533599
C	0.32782824681528	0.51830909435157	2.35217696153469
H	1.14690236124027	1.04188237940062	2.82545940435435
C	-0.50191452672302	1.03212393941606	1.33900867585681
C	-0.43808186676492	2.38347636579564	0.70800102958834
C	-1.42687349243495	0.01754597058710	0.98651759046650
C	-2.56096709983250	0.07160423841745	0.00553709756533
C	-1.24898581747169	-1.06373031600316	1.88487669541491
H	0.25463611348106	-1.41731586075584	3.46466269645402
C	-2.29108465382118	-2.11927194953630	2.06253758076489
C	1.70992882768909	-0.87323718765962	-1.70968222000525
C	2.69876668158583	-0.16030890281095	-1.01593618288702
C	2.10453445964338	1.03400050735313	-0.52331575051243
C	0.78046248130706	1.08985648960782	-0.99917644999819
C	0.51769060040477	-0.11970634027866	-1.68039055996241
H	-0.41351060520264	-0.43441663325096	-2.13310388050409
H	1.82121867036105	-1.83824724842082	-2.18175353945222
C	4.17947700120558	-0.38986540715597	-0.97166831204803
C	2.88445158445226	2.11893070837501	0.15619959288104
C	-0.16645493049501	2.22442143740231	-0.78797758044184
C	4.34400658798257	1.72200706237652	0.38711814119651
H	2.42829951933764	2.41228026906575	1.10404035898153
H	2.84778816257943	3.00877601186999	-0.48201784425756
C	4.91223392455787	0.93970133022270	-0.79090728453344
H	4.49204783121986	-0.87699740276485	-1.89752327278756
H	4.48436027645161	-1.06364183848026	-0.16511640893951
H	4.42450216408716	1.11875470259383	1.29599004959420
H	4.93478839781727	2.62121613749768	0.56807595167317
H	4.82761453020783	1.53732533192140	-1.70381416043240
H	5.97603122495800	0.74869455266074	-0.63966625909259

C	-3.72296533637903	-0.80235332451334	0.47806722939342
H	-2.23787229979675	-0.30120772352528	-0.97000329942292
H	-2.89456231822117	1.10238920647293	-0.13526862558781
C	-3.23275959234726	-2.19310760964297	0.86673497858537
H	-2.86457450380519	-1.82505105963004	2.94966739728464
H	-1.86586398500721	-3.08915665081143	2.30418468227644
H	-4.46158341257113	-0.87430027189516	-0.32236160319995
H	-4.22501887195739	-0.32976294198047	1.32899619321651
H	-4.07674062477152	-2.84144081251165	1.11223921312026
H	-2.71551718058628	-2.62829340652477	0.00656475968408
H	-1.09266462455968	2.02519177157845	-1.32697160118663
H	0.24615764390980	3.14589489066926	-1.20800580892692
H	0.34166651170667	2.97766391288272	1.18429252740734
H	-1.37455656474482	2.92158184401158	0.87719148712723
C	2.60898092600593	-2.55835284686901	0.95561837583435
O	2.57962874497381	-3.78423831920367	1.46545687807750
O	0.90828427780575	-2.66062577251713	0.26615908148869
C	2.12276564232483	-4.64263803819055	0.40778387123266
C	0.73993289573242	-4.04584816375694	0.07260770503382
C	-0.35608530411890	-4.59709283341643	0.96660396976214
H	0.48766112551661	-4.22212260768272	-0.97715921055271
C	2.15534391435409	-6.10529253306852	0.79428929331933
H	2.79596422641927	-4.50256999245482	-0.44843443996647
C	1.00743423855067	-6.55521699419399	1.68532298523214
H	3.12880611078665	-6.33450753483173	1.23416683214162
H	2.11164941638672	-6.66971965593206	-0.14153395673036
C	-0.31775883249773	-6.11470631177877	1.08210919256455
H	-1.31439550147827	-4.25792183523665	0.57347918400275
H	-0.23771367302170	-4.17862712399191	1.96939997502692
H	1.04520079988380	-7.64062282544718	1.79672855365903
H	1.11212982087090	-6.13471144570532	2.68985478682446
H	-0.45572969387615	-6.57863053503105	0.09934636006120
H	-1.15310301590557	-6.45021512521207	1.69988010195819
H	3.13466583041805	-2.50620254780302	0.00160928421981
H	3.43668043189449	-0.70798787807782	1.56484358386188
H	2.37069418115990	-1.49064147157760	2.78394351324954

TS-C_{cat}b
69
Coordinates from ORCA-job sp

C	2.87550637822516	-1.70499698967751	1.22310120970153
Cl	-1.11218950439854	-2.09697080000922	-3.47735052461829
Ti	1.21175665334870	-0.68417229910407	0.15823213242095
C	0.27107540280505	-1.01067743962124	2.31308508939964
C	0.42853684576824	0.37674674800197	2.06020932264627
H	1.09061833174564	1.04165463384280	2.59688995573990
C	-0.48058601298832	0.74170412835716	1.04664304967313
C	-0.72506856163396	2.09085338657203	0.45066263644556
C	-1.14074527113887	-0.43417936486361	0.62350186037464
C	-2.22389929574874	-0.56575862288065	-0.39909985892095
C	-0.71083794987388	-1.50453721397085	1.44157460752465
H	0.78214229180693	-1.56670103650442	3.08550743967538
C	-1.34463025724032	-2.85631973431305	1.37804524528667
C	2.55870974477030	-0.27212254943458	-1.74677394221365
C	3.15634675876490	0.53355993414549	-0.76023998522283
C	2.18941105168148	1.46925746015660	-0.32794668819009
C	1.01510514757516	1.27790368585419	-1.10393361069337
C	1.24307216071364	0.18492422585606	-1.96699550952665
H	0.51549658725754	-0.26761107363367	-2.63706203045988
H	3.05229277746380	-1.06638935873604	-2.27830541430750
C	4.59875849751243	0.59591254276838	-0.35982765549182
C	2.52786687313188	2.58675958352256	0.61280007641799

C	-0.23781067060795	2.08756573775773	-0.99977653331395
C	3.89238277815438	2.37172605074034	1.26300235403683
H	1.77035688968664	2.72209612200420	1.38403140966851
H	2.53933437975286	3.52075230814952	0.03979934161487
C	4.93458180691243	1.96435302292057	0.22955329733307
H	5.22003410351086	0.38475771620357	-1.23248758982769
H	4.84679524186573	-0.17011845498887	0.37730014031956
H	3.81710686510453	1.59364580840278	2.03123501739756
H	4.19537146757525	3.28722990942641	1.77364647177877
H	4.97293066011503	2.71640668793068	-0.56485762144698
H	5.92880687907470	1.93258715220606	0.67822722735407
C	-3.08707250131194	-1.79001482679065	-0.10255439185686
H	-1.80532979863162	-0.68838525059256	-1.40340114515637
H	-2.83236650283825	0.34309231349766	-0.41065003374274
C	-2.21419856932234	-3.01719900411724	0.13160217902441
H	-1.97402354739293	-2.95875586403711	2.26914694109257
H	-0.60934853578438	-3.66146953344532	1.44882352924610
H	-3.75464253044509	-1.96501822734926	-0.94786235664932
H	-3.72221079254412	-1.60815217672320	0.77111385478675
H	-2.83261423737566	-3.91072612277056	0.23757191995447
H	-1.59523067816237	-3.15592491878301	-0.75637483918562
H	-0.99707156759175	1.65204018638727	-1.65114318921045
H	-0.06575887571062	3.10582749411627	-1.35924203915942
H	-0.22597930302907	2.86202245114541	1.03595438794007
H	-1.79249966269062	2.32338464775780	0.49348225463736
C	2.19720370264738	-2.94636870647957	0.97402340454032
O	1.06900109666246	-2.52185742912204	-0.42106822460829
H	2.95161988397251	-1.43608802345338	2.27259942024129
H	3.82932074609981	-1.64489862640605	0.71048106963925
O	2.79051031222008	-3.95740425770459	0.34721050236070
H	1.42378496434654	-3.24733713422600	1.68165893044787
C	1.19265322918267	-3.64353322237069	-1.27044627354782
C	1.77762528271142	-4.71200495766615	-0.33700850274843
C	2.33751754761919	-5.94743061436526	-1.00571231761604
H	1.02077063343281	-5.02029150616477	0.39658353538804
C	2.07359361673436	-3.42238455443049	-2.48856725449594
H	0.19151160787855	-3.92542501563992	-1.60798670687029
C	2.36424828963268	-4.74270807566196	-3.19320159868441
H	3.03034683545717	-2.98920900905805	-2.18935942269203
H	1.56540304005647	-2.72391887338719	-3.15426280576006
C	3.14424357878997	-5.66558128511983	-2.26644520156744
H	1.47482622123725	-6.56608450260366	-1.26937177625466
H	2.91077944949611	-6.52398407592831	-0.27604789513533
H	1.42901743289324	-5.22188964631225	-3.50148081639939
H	2.92865109829975	-4.54968982183780	-4.10730696345781
H	3.37838884505846	-6.61085083738175	-2.76002034140740
H	4.10170063770844	-5.20171290005645	-2.01144875366886

TS-C$_{cat}$C
69
Coordinates from ORCA-job sp

C	0.02355152753125	-2.45600078293083	-0.41093314244240
Cl	-0.97108301258682	-1.44377123387150	-3.75211924896175
Ti	0.44471257554026	-0.48252682843169	0.46195274440259
C	-1.15396397442878	-0.68857677299787	2.19559394485905
C	-0.53395604018323	0.57612017561725	2.26138116839321
H	0.10539961852811	0.93928916314989	3.05559895878823
C	-0.92534255331010	1.31651559921435	1.12563232531123
C	-0.52568915336025	2.72042559535976	0.81010262635352
C	-1.74886569405791	0.48100110948224	0.33014183734075
C	-2.50210032891222	0.79788889807185	-0.92914901410020
C	-1.91737024774947	-0.74522032041953	1.01804395349613

H	-1.09818327111853	-1.46639631740682	2.93578587720448
C	-2.94040030831988	-1.76004563897904	0.62429098560893
C	1.86690297032030	-0.47172933361378	-1.45588461228379
C	2.70528050772658	-0.05847582228480	-0.41076608743066
C	2.16616406655897	1.13520320401742	0.13008627427916
C	1.05588239580387	1.51450166719021	-0.66654022363234
C	0.84635614492431	0.49445645745890	-1.60983648152611
H	0.07296839681347	0.43426362005964	-2.36350253280153
H	1.98658897557333	-1.33711419812681	-2.09088128301516
C	3.98666351923967	-0.66471523843143	0.06281951711918
C	2.84634559454378	1.90292941647965	1.22197382336089
C	0.23107085652344	2.75228854503001	-0.51787273152653
C	3.90594339240946	1.04906434072466	1.91534448524138
H	2.13692524621833	2.29413190799474	1.95432247279241
H	3.32695155908287	2.78205370684827	0.77740080842870
C	4.78866767694884	0.33292956830800	0.89850866931595
H	4.56916320455099	-0.99537909569110	-0.80034050995407
H	3.79782717148852	-1.56136169291102	0.65752298734072
H	3.42012009997534	0.31364496078325	2.56472855906889
H	4.51353160045555	1.68205554945484	2.56421148202562
H	5.25238048427941	1.07482686319815	0.24127292948266
H	5.60401255427801	-0.18889924147700	1.40220096127735
C	-3.76074885096398	-0.06437042701531	-1.03262773879197
H	-1.89485765061731	0.59134067927478	-1.81304563027665
H	-2.75750116403716	1.86016140409099	-0.95657767980185
C	-3.42974413537003	-1.53526405849324	-0.80272669975507
H	-3.78080320376299	-1.64942246279691	1.31877463528662
H	-2.57956114800064	-2.78056977507565	0.76982234302142
H	-4.19922029126726	0.07090726341272	-2.02249109542716
H	-4.50845251521677	0.26973124381856	-0.30570241227810
H	-4.30776017355637	-2.15739423838832	-0.98642305566531
H	-2.66428192600810	-1.83309049342040	-1.52558035504505
H	-0.46982265685457	2.80891137304013	-1.35204253656817
H	0.86248079796448	3.64182554419647	-0.58159150095556
H	0.09431871981275	3.10732559512899	1.61951738621185
H	-1.40750352226201	3.36540572474910	0.76988647597154
C	1.23176254698710	-2.93393446946485	0.20945115921547
O	1.56004926869864	-1.73190471465268	1.48786605890770
H	0.02179831987506	-2.41545711662828	-1.49881650472107
H	-0.86500185595079	-2.93606932493220	-0.01569970360165
O	1.22939282500474	-4.00102728260953	1.01788399683727
H	2.15923937927354	-2.81540503932837	-0.35524877572539
C	2.04983325386222	-2.50877156802340	2.56134890830539
C	2.34813547987040	-3.86519034075596	1.89738873212927
C	2.50634912366394	-5.04500874940543	2.83270189216094
H	3.25907297556417	-3.78154241524276	1.28788847222534
C	1.06812085710589	-2.64066934483015	3.71534508040239
H	2.98651588772863	-2.06478377021229	2.91855643332925
C	1.50868101783287	-3.71974148582900	4.69679809497383
H	0.09051672699972	-2.93149074612188	3.32683181639482
H	0.94869546025301	-1.67354879865234	4.20938600661121
C	1.51879923863296	-5.06917499603677	3.99057305076792
H	3.52042780241482	-4.97891382557562	3.23802034746325
H	2.47626802562919	-5.96830427543109	2.24991321003417
H	2.50388175694595	-3.49636091764139	5.09659136947151
H	0.82671946372428	-3.73624794745679	5.54864262171000
H	1.79252205396103	-5.86869403142030	4.68135571896188
H	0.51111255677451	-5.29706404314005	3.63117835440261

TS-C_{cat}d
69
Coordinates from ORCA-job sp

C	0.42653709372195	-2.38679213374601	-1.31071324663807
Cl	-1.48009118253516	-0.66020821803132	-3.83038923348991
Ti	0.89562050131811	-0.73030606192346	0.08635271052808
C	-0.31396287714217	-1.53021993779626	1.96104066989193
C	0.18897314085425	-0.23800407649068	2.22397080701975
H	0.93321371836394	0.01394630192858	2.96779026878859
C	-0.48866464591090	0.67630899340613	1.38900973758194
C	-0.30106338788538	2.15793617714261	1.34649716377413
C	-1.36273150598236	-0.06563765243977	0.56172331994530
C	-2.38084907111206	0.42699708181101	-0.42011524169742
C	-1.27598549671179	-1.42925911046660	0.94182965804825
H	-0.02971972922018	-2.43740615730693	2.47542265872611
C	-2.24959139626763	-2.46386449228165	0.47296322765914
C	2.05795912025261	-0.09464202349959	-1.88580570438103
C	2.95169215298106	0.16919200790882	-0.84341594563771
C	2.35591569677364	1.12934803862356	0.01186349241993
C	1.12283659224690	1.52362836923231	-0.57340936949208
C	0.92120275492347	0.73050768160555	-1.71669994232041
H	0.05184001078658	0.71410628533293	-2.36646650605477
H	2.22449148003783	-0.77665309370634	-2.70447149047855
C	4.32747678047041	-0.36846508727780	-0.64857617885595
C	3.08804456499831	1.72817483246653	1.17385544886682
C	0.18318734266637	2.56119637390727	-0.04656241986761
C	4.31653028382724	0.89568485117240	1.53545340792555
H	2.44745258987910	1.84314617303213	2.04912976634183
H	3.40297036210013	2.73990109864115	0.89513423281897
C	5.12715520829455	0.52460151628601	0.29841471922769
H	4.82921485160836	-0.44725576528064	-1.61542928173270
H	4.27128724277708	-1.38412012689258	-0.25486681918611
H	3.99743636612320	-0.01555497294289	2.05078258999038
H	4.93548130031789	1.45139467851864	2.24187860556932
H	5.42422930596063	1.43931134365407	-0.22354715378816
H	6.05063241378197	0.01878610962272	0.58818040732441
C	-3.57915527215493	-0.52237192146600	-0.44364409262318
H	-1.97083647954478	0.46236902039731	-1.43390891572603
H	-2.69670588565776	1.43956514883320	-0.15718420832982
C	-3.12022783296849	-1.95723081668263	-0.67824917840117
H	-2.88754888414304	-2.70694148425636	1.32900128656240
H	-1.75605060421410	-3.40188487586917	0.20476025696452
H	-4.25918371786664	-0.21826864548302	-1.24098060189226
H	-4.13908626378814	-0.45189314805797	0.49503797368590
H	-3.97885273740419	-2.62186301185959	-0.78921932257244
H	-2.57331054455382	-1.97650736666441	-1.62532681189523
H	-0.65846942102309	2.65240920230055	-0.73344192995544
H	0.67012376733200	3.53936953512256	-0.02135804646993
H	0.42133626435769	2.45384520221508	2.10733053492109
H	-1.23593111230228	2.66582103068627	1.59718433967710
C	1.07848556138972	-3.24740927712735	-0.36469283673999
O	2.08301363081839	-4.06395903889584	-0.70708226051773
H	0.89839138914666	-2.31705617691301	-2.28135073254659
H	-0.63577741990970	-2.56223482537644	-1.41642313797876
O	2.07897838214479	-2.20003244772309	0.65274743313803
H	0.43063400652991	-3.64474790450624	0.42270023515194
C	2.90441596721838	-4.35697015390969	0.43889668714747
C	2.55324727651787	-3.25089470802554	1.46759249385707
C	3.66790454266909	-2.90231313572948	2.44611345700209
H	1.71362995494127	-3.60581779873697	2.07913460618338
C	4.35156515884469	-4.37943214003675	-0.02136226493573
H	2.62723958330948	-5.33183549954386	0.85247980194297
C	5.31316428833614	-4.28272579256234	1.15667388226281
H	4.53005994041631	-3.54279832550799	-0.70248534974223
H	4.52931910769512	-5.29128710404209	-0.59516502369926

C	5.09358965699657	-2.98052331697989	1.91507612180334
H	3.57826300321131	-3.63286505926644	3.25584509918113
H	3.45305093624176	-1.93289826895978	2.90114217164181
H	5.17033407327812	-5.13222155680208	1.83292541233287
H	6.34022444602377	-4.34503140165150	0.79394647733403
H	5.78251471187502	-2.91185503494374	2.75934281953941
H	5.32650894393826	-2.13465790618500	1.26562926486872

TS-D$_{cat}$a

69

Coordinates from ORCA-job sp

C	1.46085787842628	-1.87428457083818	1.92821577426895
Cl	-1.73991718862509	-2.32179542864799	-3.22676497195533
Ti	0.24145236443277	-0.88573025437614	0.35922262385986
C	-1.18745483285311	-0.70964703315891	2.26924395047989
C	-0.46020776446619	0.49014146188440	2.07557175018726
H	0.29508806322294	0.89259178724308	2.73671983817464
C	-0.92863055366878	1.09912807315846	0.89608650366853
C	-0.48384439981539	2.40798784772097	0.32842071797642
C	-1.88857680716441	0.23158272895518	0.31526766953853
C	-2.73695306749672	0.43965724360004	-0.90426038630707
C	-2.09013027852308	-0.85000705816583	1.20938762101176
H	-1.10742177364276	-1.37401012052614	3.11697592699933
C	-3.27270297897754	-1.76002877912870	1.10336776576684
C	1.65554011689672	-1.19659494097164	-1.54701352878656
C	2.52657455309837	-0.66254905392864	-0.58646399442562
C	2.04030822518440	0.62499145242965	-0.22661647470288
C	0.91720372120595	0.90373557612450	-1.03456500446145
C	0.65129897169655	-0.24573214863693	-1.81136281445994
H	-0.17015728557508	-0.39576794373238	-2.50097407896139
H	1.72340353122228	-2.17396893541811	-2.00039884855954
C	3.87336410171061	-1.16354563155931	-0.16268303106363
C	2.79617410214125	1.56188580339794	0.66623406262949
C	0.13269747273458	2.17509213502659	-1.04937897868975
C	4.04906323379141	0.90906329918105	1.25507321115116
H	2.17495838785412	1.95165651995024	1.47567867829812
H	3.08829910467598	2.43448852743019	0.07169523984259
C	4.76021713391231	0.01273970111277	0.24775855232563
H	4.32410520958695	-1.71445378884799	-0.99074636000640
H	3.82327655205133	-1.87117086922987	0.67005497777719
H	3.78085520501430	0.31905365054625	2.13609758728848
H	4.72170347784632	1.69107293522169	1.61119645524514
H	5.03183077130885	0.59405235984948	-0.63873467032171
H	5.69239239616012	-0.36390327111149	0.67225015432169
C	-4.08084238009624	-0.27412059506906	-0.74985815595264
H	-2.24622832209258	0.03053158687409	-1.79080852156326
H	-2.88671543777821	1.50795944117503	-1.07912764500083
C	-3.89140206986412	-1.71684618092247	-0.28973898536290
H	-4.00714276961442	-1.38806154202175	1.82747861065366
H	-3.05302051125331	-2.77960414992158	1.42461941341848
H	-4.60408366681555	-0.25586828806788	-1.70730917395939
H	-4.71160811696090	0.26422174921573	-0.03464148643061
H	-4.85137289627246	-2.23695951511731	-0.27135551175268
H	-3.25347895663348	-2.22805990775266	-1.01762963587896
H	-0.64811496711219	2.09847328928359	-1.80672405981443
H	0.76757104814292	3.01558464524076	-1.34176146467153
H	0.23645736702689	2.87211723560589	1.00280466475367
H	-1.33065839196948	3.09664815296312	0.26452907763396
C	1.42540007116735	-3.09104528747930	1.17084397789854
O	0.00240560227556	-2.73732721851230	-0.05091821897850
H	0.97360508223251	-1.93253696518124	2.89502935897292
H	2.45315932863280	-1.44494524373735	2.01186244035013

O	0.90714132772070	-4.23991916710237	1.61537258744644
H	2.22838207409458	-3.26299078585871	0.45593228155582
C	-0.81298546916072	-3.85707258258824	0.13630744087005
C	-0.52313694510191	-4.30275305323207	1.56897287213664
C	-0.99628579178444	-5.69844220047076	1.90901207858506
H	-0.94534558645917	-3.58634426066225	2.28592616373994
C	-0.49554889955041	-4.92387283985358	-0.90254200565029
H	-1.86024726151514	-3.56829394434625	0.03689570061737
C	-1.13469080302089	-6.25806477584634	-0.53201724323802
H	0.58796483604735	-5.05457632793389	-0.98612904184427
H	-0.84831825321230	-4.55104635271113	-1.86640467139214
C	-0.65676803147870	-6.72736250070676	0.83689282919518
H	-2.08311722779613	-5.63957726141766	2.02340979950430
H	-0.60466604030987	-5.98920975796960	2.88638934218266
H	-2.22622055828766	-6.16152665396892	-0.52595611199691
H	-0.89955933955816	-7.00294211793908	-1.29482801003105
H	-1.11744581596791	-7.68149132744695	1.10065374241772
H	0.42243412895958	-6.90146457107441	0.80946064347551

TS-D$_{cat}$b

69

Coordinates from ORCA-job sp

C	1.64591054328785	-2.05549552722198	1.71472516091192
Cl	-1.27328787178173	-2.19356323124626	-3.73062264595489
Ti	0.48028990280435	-0.88586192168611	0.24661843823038
C	-0.91137541309753	-0.81155092990348	2.16342131622601
C	-0.35648840139024	0.47283910171877	1.93619642154252
H	0.33117626293799	0.99376201088689	2.58855028834824
C	-0.90524840796716	0.98322427615372	0.74602744840521
C	-0.63204697336368	2.31894806336855	0.13551026928850
C	-1.74029137741609	-0.01968603350761	0.19425153662655
C	-2.58260814309562	0.02813770958850	-1.04361286149528
C	-1.78353662883256	-1.10204816594060	1.10509478318388
H	-0.73416000088313	-1.42719564830038	3.03273619759014
C	-2.70473772678105	-2.26401650862827	0.93789622199685
C	2.04063850041866	-1.03747193592371	-1.55645812878839
C	2.78084177490119	-0.40753315611706	-0.54149624334626
C	2.12102363426615	0.80366479196802	-0.21754405889703
C	1.02559693408488	0.95488937280236	-1.10744580234158
C	0.96119685466418	-0.20221190898618	-1.90806600127140
H	0.20915287386497	-0.43024910989204	-2.65732370788200
H	2.31001656748037	-1.95850404151206	-2.04945009282764
C	4.14249556272396	-0.77159410136743	-0.03855456080794
C	2.70336609943766	1.78988628061412	0.74763876273624
C	0.09831155217808	2.12592450264600	-1.19339905031916
C	3.82187916817815	1.15239402343276	1.56856307432048
H	1.95162264916567	2.21883637836379	1.41120684829921
H	3.10813474573008	2.63183143088433	0.17483997425088
C	4.80485513620244	0.41021456367014	0.67102220981462
H	4.74983654395906	-1.09097386072985	-0.88877024059524
H	4.11855264370455	-1.63214401662306	0.63345318901147
H	3.39133223473497	0.46018955309421	2.29895508040483
H	4.33951794600058	1.92569891066236	2.13861793247006
H	5.20664004994173	1.10667525413647	-0.07098629924286
H	5.65753274340750	0.05300537332149	1.25093441852841
C	-3.79601910318905	-0.88641592156276	-0.88214890890920
H	-2.02525107695118	-0.32404604333640	-1.91772989428852
H	-2.89138245756687	1.05677315668870	-1.24781757094476
C	-3.35406813913576	-2.27855790628358	-0.44515075104045
H	-3.47998815762846	-2.17724112194001	1.70698695140978
H	-2.19436557232820	-3.20922084143205	1.13259489868047
H	-4.32297560794818	-0.94808821110189	-1.83595269699088

H	-4.49874608623987	-0.46121791369091	-0.15787762615588
H	-4.20437918482482	-2.96381696184978	-0.43489838317852
H	-2.64712603015095	-2.65328178861376	-1.18746647435099
H	-0.61916253877322	1.94456210672580	-1.99440558275479
H	0.64851732604446	3.02836265236196	-1.47253898479617
H	-0.03759477315826	2.91836032440907	0.82414138842385
H	-1.56790096309268	2.86317569650500	-0.01458981965086
C	0.84822451776681	-3.22440244053214	1.44933273799363
O	1.33170900114963	-4.36275286412467	0.95915224174346
H	1.53055637606631	-1.69203607883552	2.73181022814858
H	2.68934717870352	-2.18390812622824	1.45449849388095
O	0.01028446034377	-2.70676634351530	-0.18907931974890
H	-0.02513414974748	-3.39641976725871	2.07580144786651
C	1.73889523526897	-4.25183746524194	-0.41097774460407
C	0.46328928642670	-3.71158219188828	-1.06536448843064
C	-0.60720925481510	-4.78089208731929	-1.23877358020862
H	0.67698954714063	-3.27538703995876	-2.04551530177342
C	2.21751195173242	-5.58606441354975	-0.93873056497118
H	2.55307326721161	-3.52050792583685	-0.48601601218147
C	1.10204555428375	-6.61211271375297	-1.09294774067771
H	3.02913822099453	-5.95757474404752	-0.30900911921681
H	2.66018822444323	-5.38769276399441	-1.91960795592072
C	-0.04435752983050	-6.02829459950346	-1.90850261922098
H	-1.40585897043838	-4.34908108257999	-1.84318426455355
H	-1.03375782124746	-5.05330842106505	-0.26856058106751
H	1.50022984083811	-7.50781905297523	-1.57379757530518
H	0.73371570607927	-6.92311551853379	-0.11139192819058
H	0.30777039123966	-5.77975888162296	-2.91549712251017
H	-0.83907864813345	-6.76641420423990	-2.03435565492139

TS-D_{cat}C
69
Coordinates from ORCA-job sp

C	0.60790238555780	-2.13259727535033	0.11305129605952
Cl	-1.79969559613157	-2.37592448233176	-2.41723728870937
Ti	1.24414317515734	-0.01531538685671	0.18368178446929
C	0.46792720379835	0.60871109505628	2.33687463893910
C	0.79631339703080	1.75116102837651	1.57496150135340
H	1.59009556040493	2.45523755354232	1.78778437341220
C	-0.15247149288780	1.86764719915228	0.52895422223187
C	-0.23532767673483	2.93912405238247	-0.51029834593950
C	-1.01099080314256	0.75554010867639	0.60760316433715
C	-2.22733601539910	0.45421138535844	-0.20630503804952
C	-0.64314746417507	-0.01145605515931	1.74480121824706
H	0.98254789558406	0.27332754353995	3.22522256632080
C	-1.51032591396862	-1.10594730567619	2.28711657606651
C	2.20898654639826	-0.71959333684887	-1.86098260678986
C	3.11146068087885	0.23216878090337	-1.37215011286749
C	2.42340715180870	1.46243068851227	-1.24974716243783
C	1.11087216012667	1.28106231041418	-1.76853229474832
C	0.97126164057605	-0.07637999123219	-2.11036588727936
H	0.06697034148512	-0.56438805965244	-2.46426956434153
H	2.43270367785443	-1.76049111659227	-2.04203777790486
C	4.57011438863819	0.06668080957120	-1.08510149267477
C	3.11911737221662	2.71718990126778	-0.81877467823421
C	0.05563175320166	2.33391510639928	-1.88598089587600
C	4.41248343088581	2.38446929419860	-0.07983100816781
H	2.48018589826731	3.34904026985041	-0.20224300747361
H	3.35650267989176	3.30972389925331	-1.70969049795761
C	5.26355525587346	1.41641621653790	-0.89304653075974
H	5.03334311768903	-0.47738471251658	-1.91145376023952
H	4.72262885292989	-0.55696699908514	-0.20525123600417

H	4.17708684311911	1.94870856495407	0.89834752520593
H	4.96728064703422	3.30355092832324	0.11532065664984
H	5.46824569298656	1.86452523609058	-1.86987042668256
H	6.23228308801014	1.26149757301401	-0.41521092997602
C	-3.26556500479162	-0.25074381694670	0.66611812932379
H	-1.98622256972201	-0.20772969212272	-1.04511436715978
H	-2.63526423565175	1.37805075837039	-0.62438607526603
C	-2.65471307229202	-1.47562019375665	1.33671791839865
H	-1.93271709315374	-0.73658925824230	3.22732617466508
H	-0.93505678478396	-1.99445558168990	2.56294777117074
H	-4.10888496691064	-0.55721166328643	0.04503817368141
H	-3.66145769311985	0.43865355822950	1.41934126772634
H	-3.41331572657001	-2.02395651260310	1.89824132682576
H	-2.31092259795008	-2.14282020785543	0.54156304492363
H	-0.84685517775957	1.88023041530444	-2.29744675285332
H	0.36404752380021	3.10743705934977	-2.59386126946750
H	0.46875728323716	3.73677657925710	-0.27720161213908
H	-1.22781040264339	3.39699071952984	-0.49822724472467
C	1.61041797038959	-2.39255065115392	1.10710374929924
O	2.57718881531536	-0.88549912421614	1.27139010547423
H	0.76349884769370	-2.59304510725077	-0.85373349703373
H	-0.40402802826076	-2.29246273168677	0.45142266068925
O	2.64248001140458	-3.19415402760490	0.81039875247959
H	1.26713477860158	-2.41962548486635	2.14405857021565
C	3.66844889743629	-1.40368690821626	1.98885662220169
C	3.91299537639828	-2.77628328592098	1.33597500545927
C	4.56857279721851	-3.81859395003309	2.22418094481695
H	4.54402824784361	-2.64019983021279	0.45302901240891
C	3.38077661195707	-1.42045044185948	3.49192529740079
H	4.54698674937376	-0.77653826362051	1.80969469126016
C	4.27460364441051	-2.39633335865874	4.24611914689622
H	2.34400521374773	-1.71142758192093	3.67691012240573
H	3.49291783486409	-0.40501831623711	3.87746777241780
C	4.10679030867047	-3.79677427189992	3.67316090391519
H	5.64371136620073	-3.61608366123284	2.20093498926917
H	4.43939818995764	-4.80366874208559	1.77092909141004
H	5.32420754722146	-2.09214754480998	4.17467504307808
H	4.01637739493288	-2.37406010517915	5.30614889485954
H	4.68731433632389	-4.52221882407627	4.24581482650907
H	3.05970773164316	-4.10643477486794	3.75278182928240

TS-D$_{cat}$d

69

Coordinates from ORCA-job sp

C	0.57921902070548	-2.21726809682501	0.30059495785416
Cl	-1.47574948471866	-2.43110824083850	-2.65656863005188
Ti	0.98534655223445	-0.06522001126770	0.37006134304966
C	-0.06007985525548	0.36848808609541	2.45611494883949
C	0.37024242147611	1.57956564102065	1.87371189877639
H	1.13069548301545	2.23912446341288	2.26993638319756
C	-0.40013514844954	1.80782127530397	0.71411921031367
C	-0.30726954407682	2.99159058844204	-0.19316017464092
C	-1.26951238024286	0.70142190133683	0.55078045313994
C	-2.34953064049226	0.47883620181007	-0.46491005041291
C	-1.08307145739147	-0.16607370598083	1.65512078727948
H	0.27867094667133	-0.04222443847374	3.39517995989483
C	-2.01768756851981	-1.28650760185236	1.97297984744041
C	1.98348388632964	-0.69198169441347	-1.69997503731534
C	2.94167849501115	0.13285091778347	-1.09194261599884
C	2.35414213299098	1.40675833927332	-0.89929124244669
C	1.06421233934582	1.38844541299873	-1.49348717904847
C	0.82486812427249	0.08009036205335	-1.94697559299242

H	-0.07856006371378	-0.30752210558211	-2.40487840172154
H	2.11827206697606	-1.72155793423176	-1.99872618453612
C	4.36026509936595	-0.18281022601131	-0.74711698586868
C	3.11970835004974	2.56285334183753	-0.33271027136935
C	0.10752585411448	2.53437068403094	-1.59166695689639
C	4.38273542658500	2.08027862583876	0.37761933934290
H	2.50908853993347	3.16451037620898	0.34282931602002
H	3.39811765581911	3.22944712932303	-1.15655740201995
C	5.15943744356302	1.09609823474659	-0.49009457237919
H	4.81019479326110	-0.75045661360266	-1.56513210470375
H	4.40734869627712	-0.82758240163789	0.13309093950288
H	4.11265186645499	1.59980260456136	1.32346732248209
H	5.00672366739583	2.93936486669132	0.62990108414495
H	5.40482583640941	1.57569592190720	-1.44265077431768
H	6.10909926173169	0.84026771510217	-0.01726504769607
C	-3.47324237218699	-0.36266772597943	0.14181863125679
H	-1.97187640788036	-0.06447141292682	-1.33631783106472
H	-2.73423264038034	1.43764695367150	-0.82097360696880
C	-2.91300749753335	-1.62749757291127	0.78450257493236
H	-2.63902930686116	-0.95440958146256	2.81180155491584
H	-1.48584603665413	-2.16722983672219	2.34065109971064
H	-4.18110586257566	-0.62658162554877	-0.64533694573452
H	-4.02674097370063	0.22395221776101	0.88299436302542
H	-3.72493918553161	-2.27466196821513	1.12213650898048
H	-2.35462893194510	-2.17670251444470	0.02092055368871
H	-0.76683175952603	2.20964866317558	-2.15635116990309
H	0.55199944779896	3.35817103176014	-2.15597694098493
H	0.41218795283039	3.70218305150711	0.21436127574580
H	-1.26554528148737	3.51686844451300	-0.22587530403552
C	1.92878296418812	-2.49863709347129	0.72999036183799
O	2.21706858566055	-3.21857631766742	1.82441509199055
H	0.33269301772381	-2.53593711891896	-0.71129393487321
H	-0.16562180602518	-2.56744322573444	1.00763153741966
O	2.41620551782507	-0.87298355114315	1.44084858852043
H	2.68972715735196	-2.63611359764534	-0.03628517781197
C	1.94137737605548	-2.50341092745665	3.02690660375161
C	2.70054102851942	-1.19570635141231	2.78116704940014
C	4.20638014339197	-1.33950942951634	2.97386602124584
H	2.31671162490995	-0.41577885159647	3.44506797177310
C	2.38874871071380	-3.29255930274572	4.23811010478236
H	0.86369878803961	-2.30830853712402	3.10253695066964
C	3.90295443355962	-3.39594772961234	4.37271369047235
H	1.91686113083337	-4.27709551457411	4.22198386756159
H	1.98476331942731	-2.77489042263981	5.11272269707890
C	4.54186741390692	-2.01483226658092	4.29872882618640
H	4.66497171223088	-0.34999349921771	2.91175173747406
H	4.62809593378970	-1.93072685498090	2.15571965461852
H	4.14613386572748	-3.87684194001118	5.32202757931023
H	4.31376559210143	-4.03573643621613	3.58673947156353
H	4.18930735553237	-1.40161874702110	5.13509290795656
H	5.62556717304012	-2.08788002795228	4.40643506864479

References

[1] D. Xu, G. A. Crispino, K. B. Sharpless, *J. Am. Chem. Soc.* **1992**, *114*, 7570–7571.
[2] A. Diaz-Ortiz, E. Diez Barra, A. de la Hoz, P. Prieto, *Synth. Commun.* **1993**, *23*, 1935–1942.
[3] P. C. Zhu, J. Lin, C. U. Pittman, *J. Org. Chem.* **1995**, 60, 5729–5731.
[4] L. Emmanuvel, T. M. A. Shaikh, A. Sudalai, *Org. Lett.* **2005**, *7*, 5071–5074.
[5] J. A. Myhill, C. A. Wolhelmsen, L. Zhang, J. P. Morken, *J. Am. Chem. Soc.* **2018**, *140*, 15181–15185.
[6] H. Mandai, H. Yasuhara, K. Fuji, Y. Shimomura, K. Mitsudo, S. Suga, *J. Org. Chem.* **2017**, *82*, 6846–6856.
[7] P. Saisaha, D. Pijper, R. P. van Summeren, R. Hoen, C. Smit, J. W. de Boer, R. Hage, P. L. Alsters, B. L. Feringa, W. R. Browne, *Org. Biomol. Chem.* **2010**, *8*, 4444–4450.
[8] W. J. Neary, J. G. A. Kennemur, *Macromol. Rapid. Commun.* **2016**, *37*, 975–979.
[9] A. Diouf, T. Darmanin, S. Y. Dieng, F. Guittard *J. Colloid Interface Sci.* **2015**, 453, 42–47.
[10] S. Mirilashvili, N. Chasid-Rubinstein, A. Albeck, *Eur. J. Org. Chem.* **2010**, *24*, 4671–4686.
[11] N. Fujieda, T. Nakamo, Y. Taniguchi, H. Ichihashi, H. Sugimoto, Y. Morimoto, Y. Nishikawa, G. Kurisu, S. Itoh, *J. Am. Chem. Soc.* **2017**, *139*, 5149–5155.
[12] C. M. Plummer, P. Kraft, J. Froese, T. Hudlicky, T. J. Rook, O. A. H. Jones, H. M. Hügel, *Asian J. Org. Chem.* **2015**, *4*, 1075–1084.
[13] L. Maier, P. Khirsariya, O. Hylse, S. K. Adla, L. Cernova, M. Poljak, S. Krajcovicova, E. Weis, S. Drapela, K. Soucek, K. Paruch, *J. Org. Chem.* **2017**, *82*, 3382–3402.
[14] L. Bayeh, P. Q. Le, U. K. Tambar, *Nature,* **2017**, *547*, 196–200.
[15] a) Avogadro: an open-source molecular builder and visualization tool. Version 1.20. http://avogadro.cc/ (accessed Dec10 2021). b) M. D. Hanwell, D. E. Curtis, D. C. Lonie, T. Vandermeersch, E. Zurek, G. R. Hutchison, *J. Cheminf.* **2012**, *4*, 17.
[16] B. Neises, W. Steglich, *Angew. Chem. Int. Ed.* **1978**, *17*, 522–524.
[17] T. Arai, T. Mizukami, A. Yanagisawa, *Org. Lett.* **2007**, *9*, 1145–1147.
[18] D. B. Dess, J. C. Martin, *J. Org. Chem.* **1983**, *48*, 4155–4156.
[19] J. A. Dodge, J. I. Trujillo, M. Presnell, *J. Org. Chem.* **1994**, *59*, 234–236.
[20] S. E. Sen, S. L. A. Roach, *Synthesis* **1995**, *7*, 756–758.
[21] N. Miyaura, T. Ishiyama, H. Sasaki, M. Ishikawa, M. Satoh, A. Suzuki, *J. Am. Chem. Soc.* **1989**, *111*, 314-321.
[22] M. Wächter, P. Rüedi, *Chem. Biodiversity* **2009**, *6*, 283-294.
[23] M. Minato, K. Yamamoto, J. Tsuji, *J. Org. Chem.* **1990**, *55*, 766-768.
[24] a) C. Matt, F. Kölblin, J. Streuff, *Org. Lett.* **2019**, *21*, 6983–6988. b) C. Matt, C. Kern, J. Streuff, *ACS Catal.* **2020**, *10*, 6409–6413.
[25] G. Erker, R. Schlund, C. Krüger, *Organometallics* **1988**, *8*, 2349–2355.
[26] A. Gansäuer, S. Narayan, N. Schiffer-Ndene, H. Bluhm, J. E. Oltra, J. M. Cuerva, A. Rosales, M. Nieger *J. Organomet. Chem.* **2006**, *691,* 2327-233.
[27] G. Erker, M. Aulbach, M. Knickmeier, D. Wingbermühle,, C. Krüger, M. Nolte, S. Werner *J. Am. Chem. Soc.* **1993**, *115*, 4590-4601.
[28] a) F. Neese, *Wiley Interdiscip. Rev.: Comput. Mol. Sci.* **2017**, *8*, e1327; b) F. Neese, F. Wennmohs, U. Becker, C. Riplinger, *J. Chem. Phys.* **2020**, *152*, 224108.
[29] a) S. Grimme, *J. Chem. Theory Comput.* **2019**, *15*, 2847–2862; b) P. Pracht, F. Bohle, S. Grimme, *Phys. Chem. Chem. Phys.* **2020**, *22*, 7169–7192; c) CREST version 2.11, University Bonn, Mulliken Center for Theoretical Chemistry, Bonn, Germany **2021**, https://github.com/grimme-lab/crest/releases; d) xtb version 6.4.0, University of Bonn, Mulliken Center for Theoretical Chemistry, Bonn, Germany **2021**, https://github.com/grimme-lab/xtb.
[30] S. Grimme, J. G. Brandenburg, C. Bannwarth, A. Hansen, *J. Chem. Phys.* **2015**, *143*, 054107.
[31] The calculations utilized the geometrical counterpoise correction gCP and the atom-pairwise dispersion correction with Becke-Johnson damping (D3BJ): a) S. Grimme, J. Antony, S. Ehrlich, H. Krieg, *J. Chem. Phys.* **2010**, *132*, 154104; b) S. Grimme, S. Ehrlich, L. Goerigk, *J. Comput. Chem.* **2011**, *32*, 1456–1465; c) H. Kruse, S. Grimme, *J. Chem. Phys.* **2012**, *136*, 154101.
[32] M. Cossi, N. Rega, G. Scalmani, V. Barone, *J. Comput. Chem.* **2003**, *24*, 669–681.
[33] D. F. Aycock, *Org. Process Res. Dev.* **2007**, *11*, 156–159.

[34] Y. Zhao, D. G. Truhlar, *J. Phys. Chem. A* **2005**, *109*, 5656–5667.
[35] F. Weigend, R. Ahlrichs, *Phys. Chem. Chem. Phys.* **2005**, *7*, 3297–3305.
[36] F. Weigend, *Phys. Chem. Chem. Phys.* **2006**, *8*, 1057–1065.
[37] a) K. Eichkorn, O. Treutler, H. Öhm, M. Häser, R. Ahlrichs, *Chem. Phys. Lett.* **1995**, *240*, 283–290; b) K. Eichkorn, F. Weigend, O. Treutler, R. Ahlrichs, *Theor. Chem. Acc.* **1997**, *97*, 119–124; c) R. Bauernschmitt, M. Häser, O. Treutler, R. Ahlrichs, *Chem. Phys. Lett.* **1997**, *264*, 573–578; d) P. Deglmann, K. May, F. Furche, R. Ahlrichs, *Chem. Phys. Lett.* **2004**, *384*, 103–107; e) C. K. Skylaris, L. Gagliardi, N. C. Handy, G. Ioannou, S. Spencer, A. Willets, *J. Mol. Struct. (THEOCHEM)* **2000**, *501*, 229–239.
[38] F. Neese, F. Wennmohs, A. Hansen, U. Becker, *Chem. Phys.* **2009**, *356*, 98–109.
[39] a) E. Caldeweyher, C. Bannwarth, S. Grimme, *J. Chem.Phys.* **2017**, *147*, 034112; b) E. Caldeweyher, S. Ehlert, A. Hansen, H. Neugebauer, S. Spicher, C. Bannwarth, S. Grimme, *J. Chem. Phys.* **2019**, *150*, 154122.
[40] CYLview 2.0 (Build0001), C. Y. Legault, Université de Sherbrooke, **2020**. https://www.cylview.org (accessed 2021-09-02).
[41] P. C. Wailes, H. Weigold, *J. Organomet. Chem.* **1970**, *24*, 405–411.
[42] E. Yu. Pankratyev, T. V. Tyumkina, L. V. Parfenova, S. L. Khursan, L. M. Khalilov, U. M. Dzhemilev, *Organometallics* **2011**, *30*, 6078–6089.

NMR Spectra

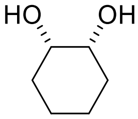

S1

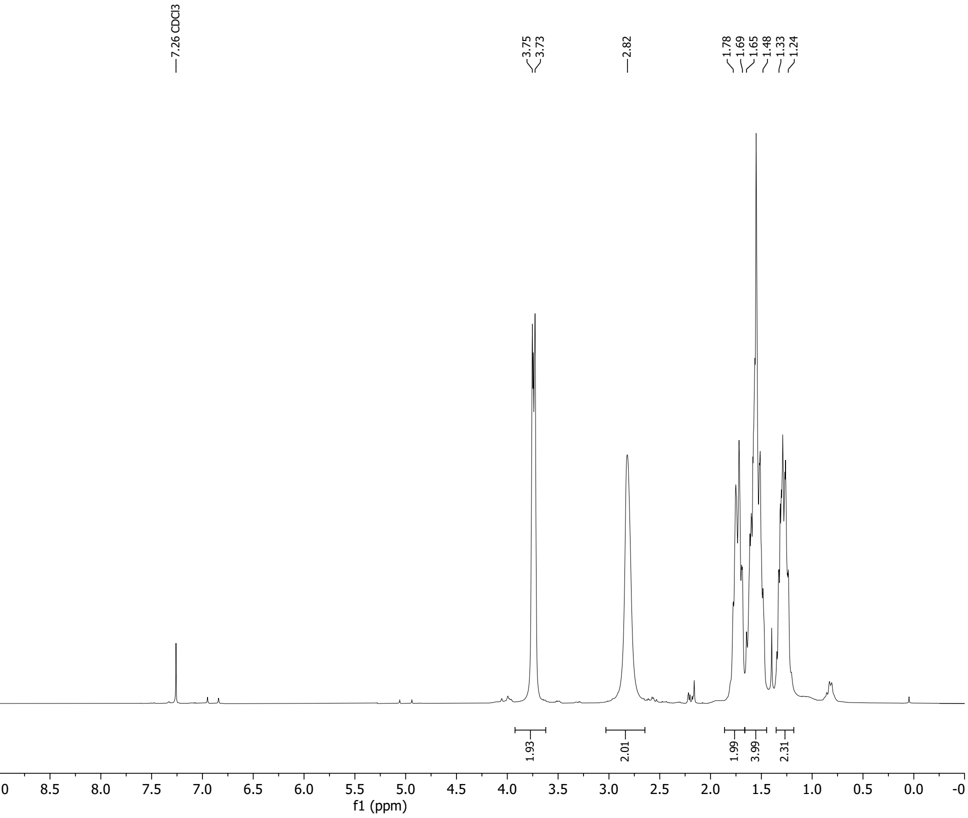

^{1}H NMR (400 MHz, $CDCl_3$) spectrum of compound **S1**.

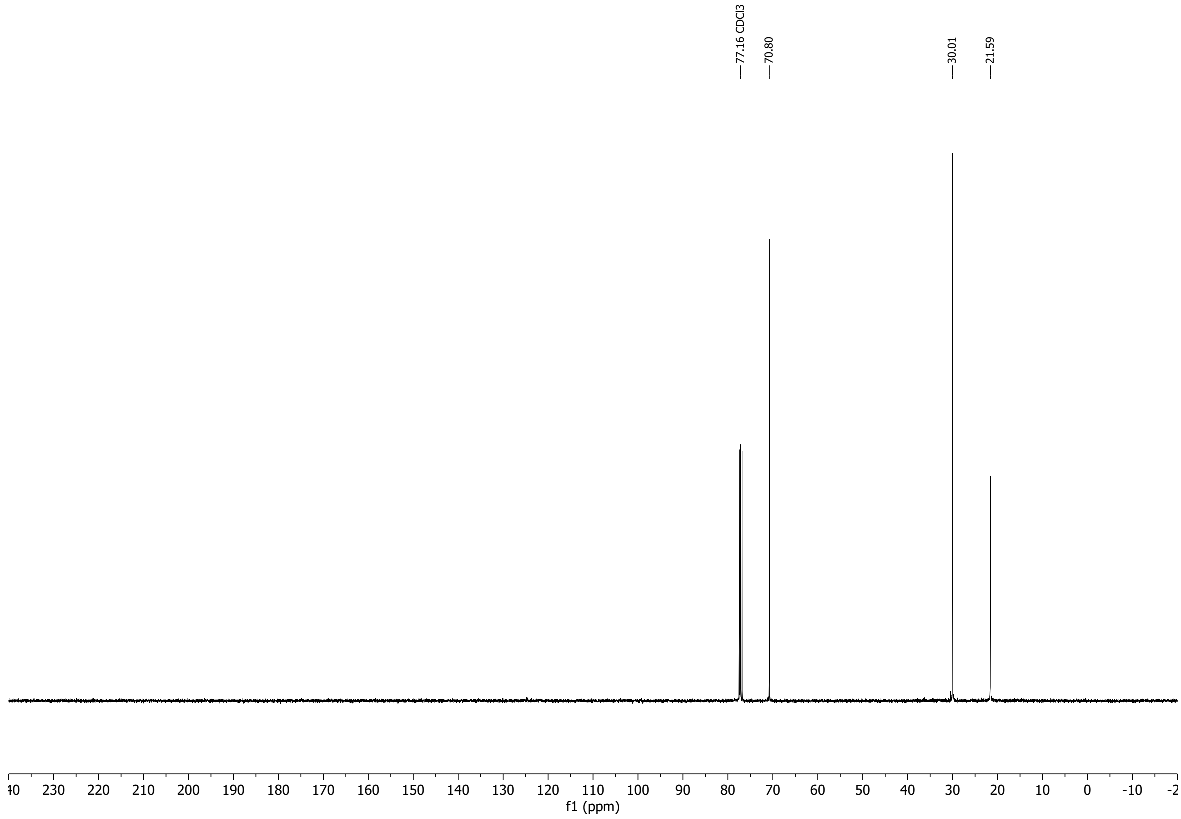

^{13}C NMR (100 MHz, $CDCl_3$) spectrum of compound **S1**.

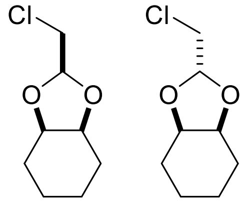

S2

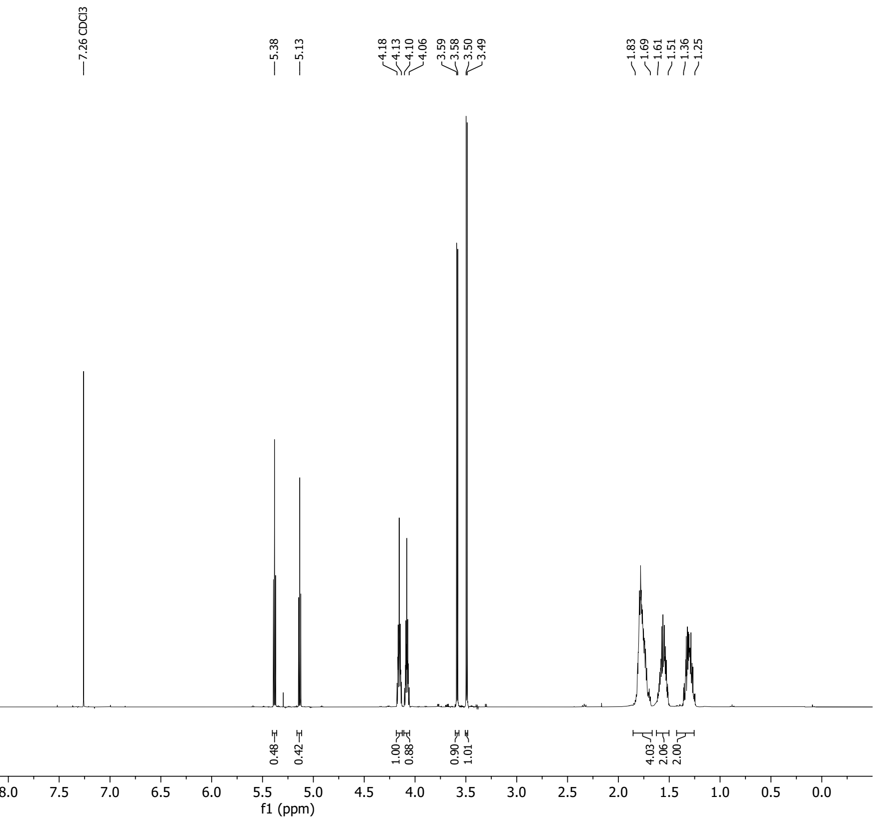

^{1}H NMR (400 MHz, $CDCl_3$) spectrum of compound **S2**.

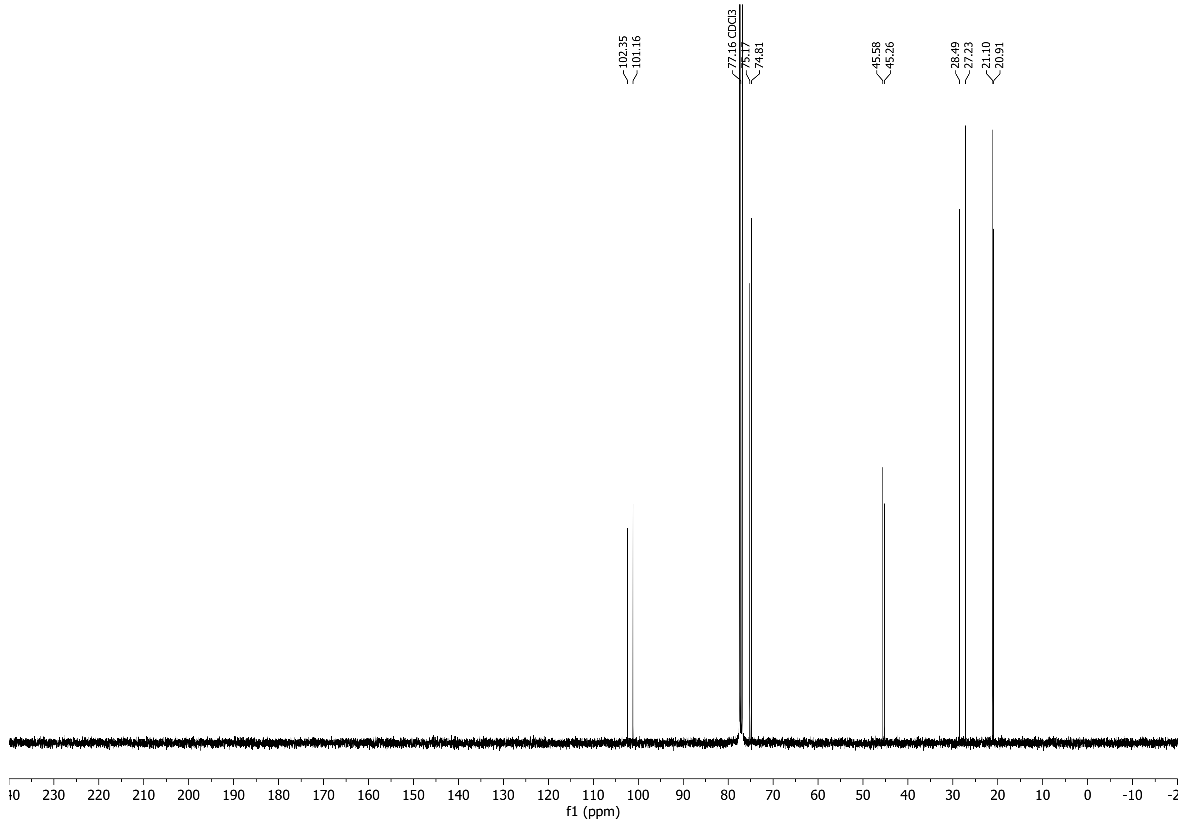

^{13}C NMR (100 MHz, $CDCl_3$) spectrum of compound **S2.**

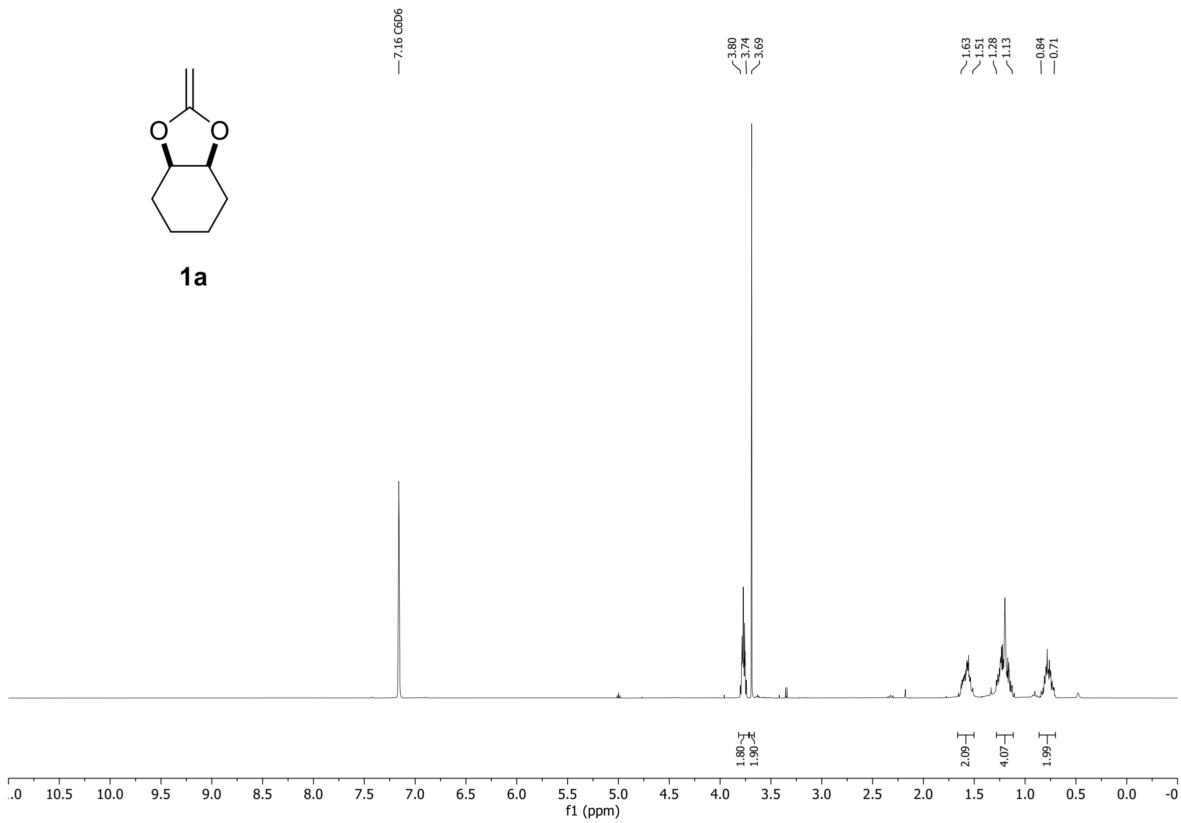

^{1}H NMR (400 MHz, C_6D_6) spectrum of compound **1a**.

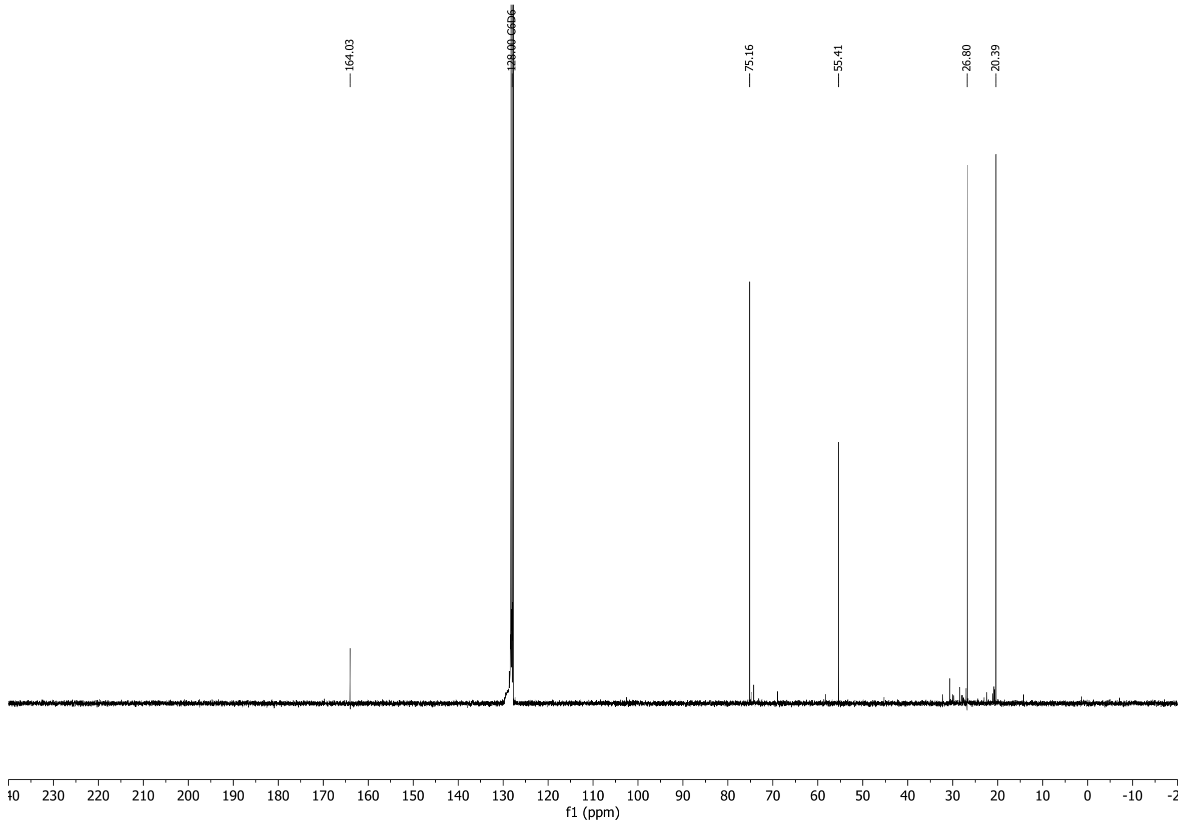

^{13}C NMR (100 MHz, C_6D_6) spectrum of compound **1a.**

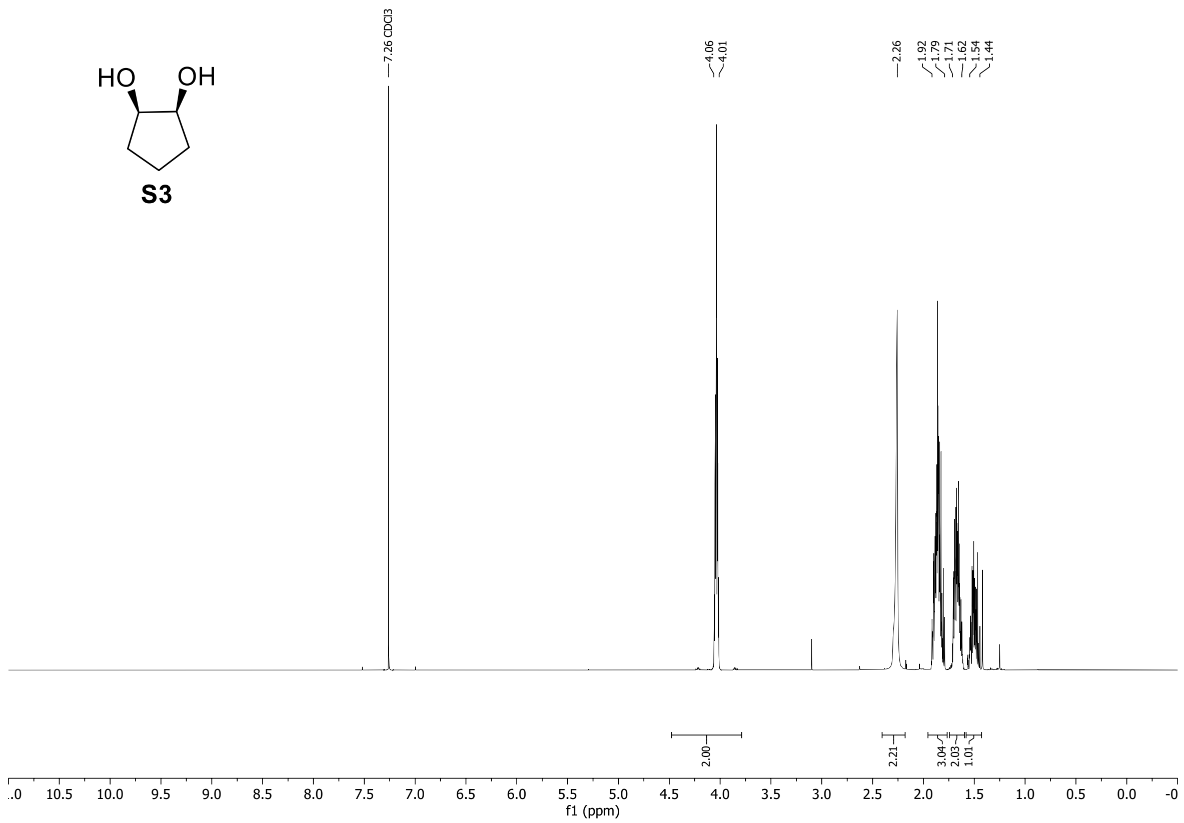

^{1}H NMR (400 MHz, $CDCl_3$) spectrum of compound **S3**.

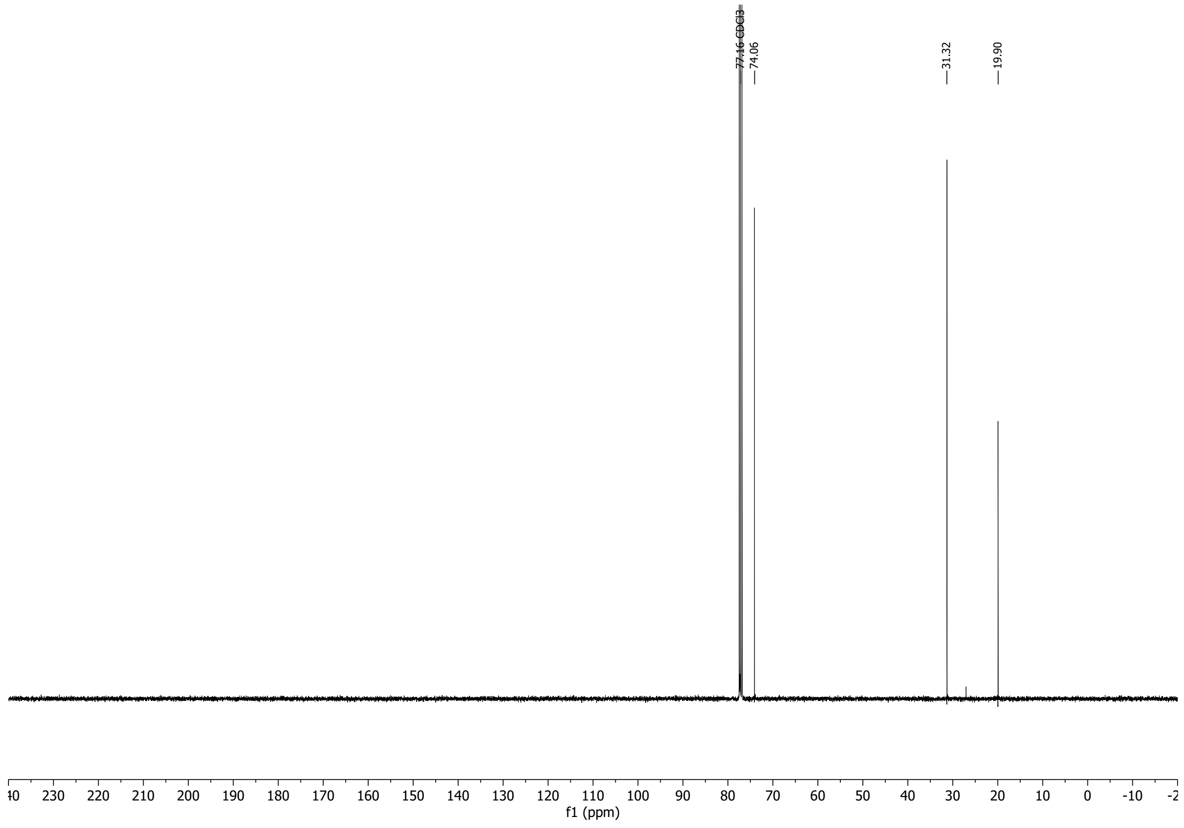

^{13}C NMR (100 MHz, $CDCl_3$) spectrum of compound **S3**.

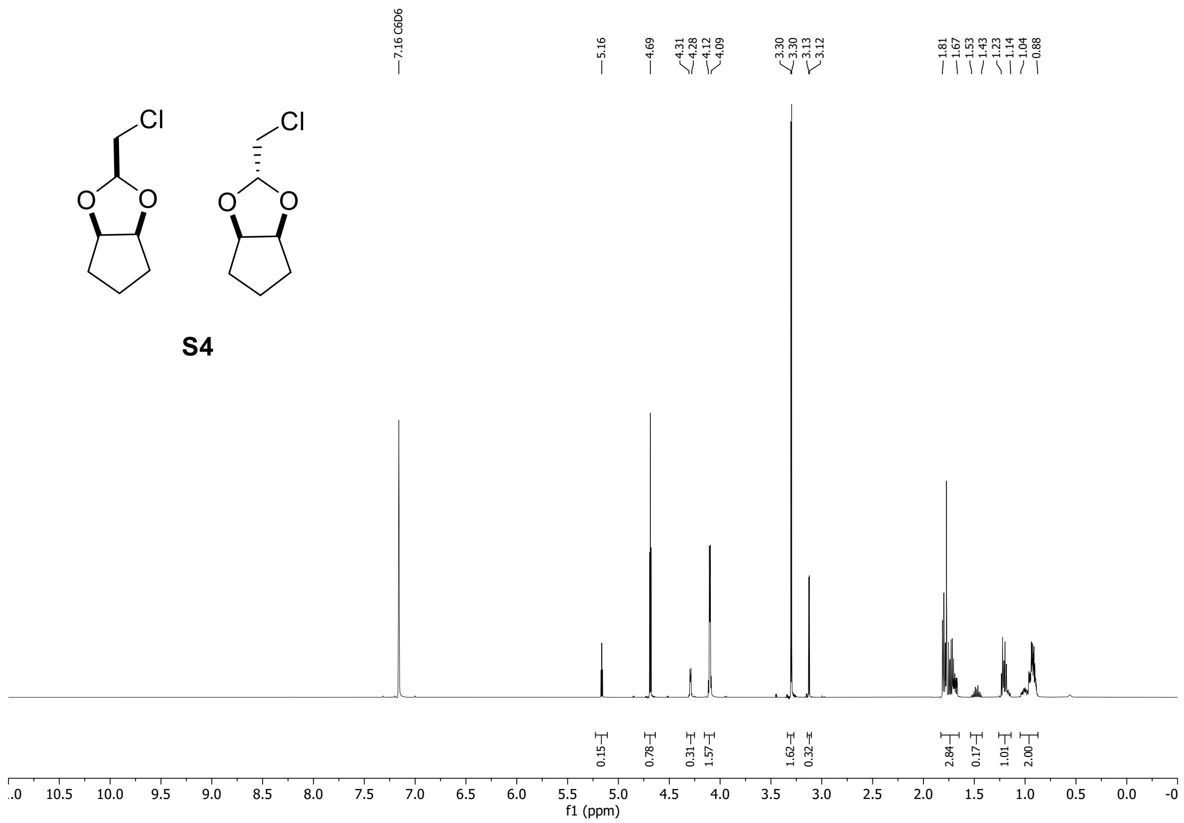

^{1}H NMR (500 MHz, C_6D_6) spectrum of compound **S4**.

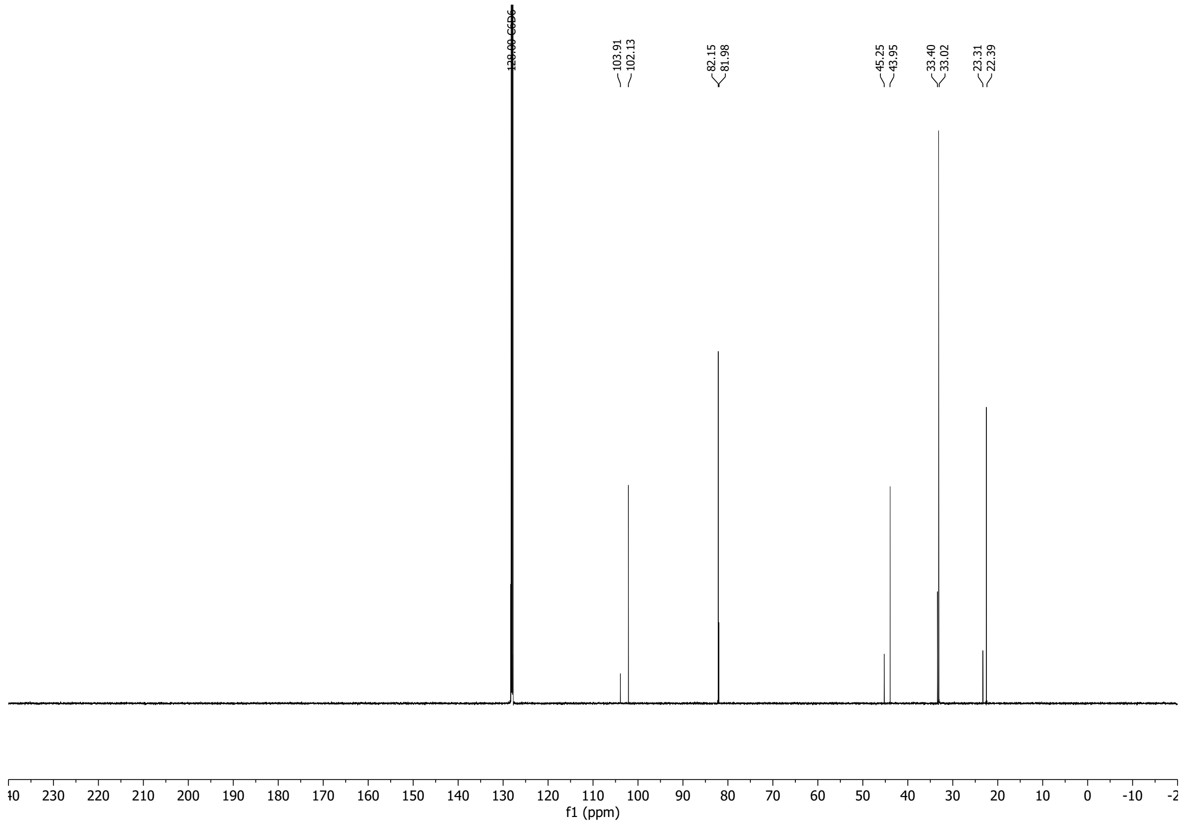

^{13}C NMR (125 MHz, C_6D_6) spectrum of compound **S4**.

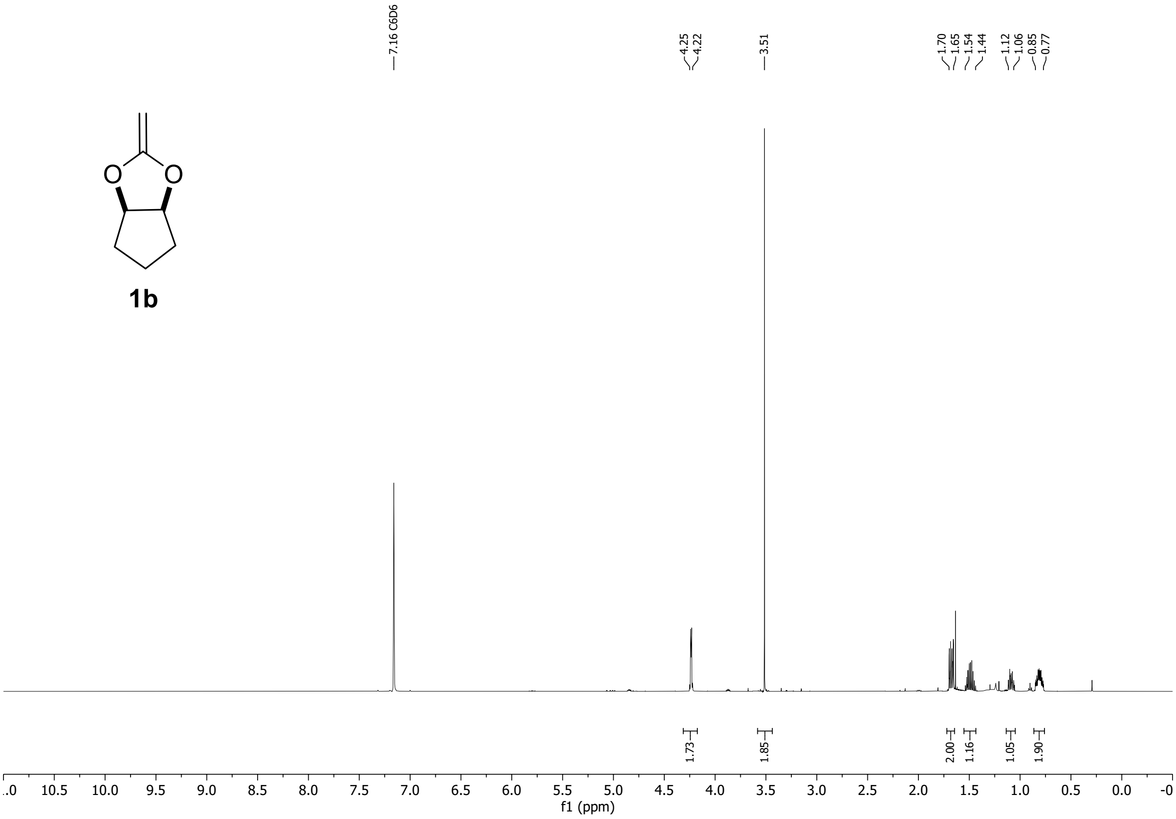

1b

^{1}H NMR (500 MHz, C_6D_6) spectrum of compound **1b**.

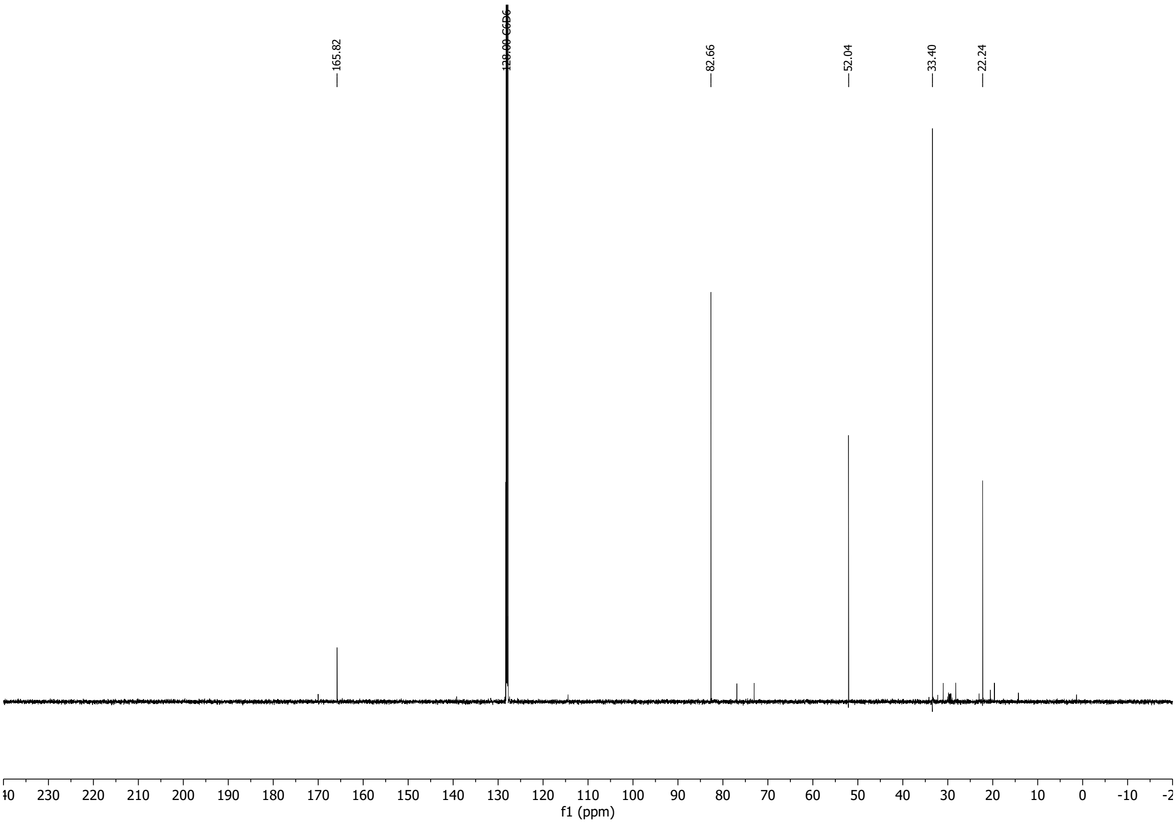

^{13}C NMR (125 MHz, C_6D_6) spectrum of compound **1b**.

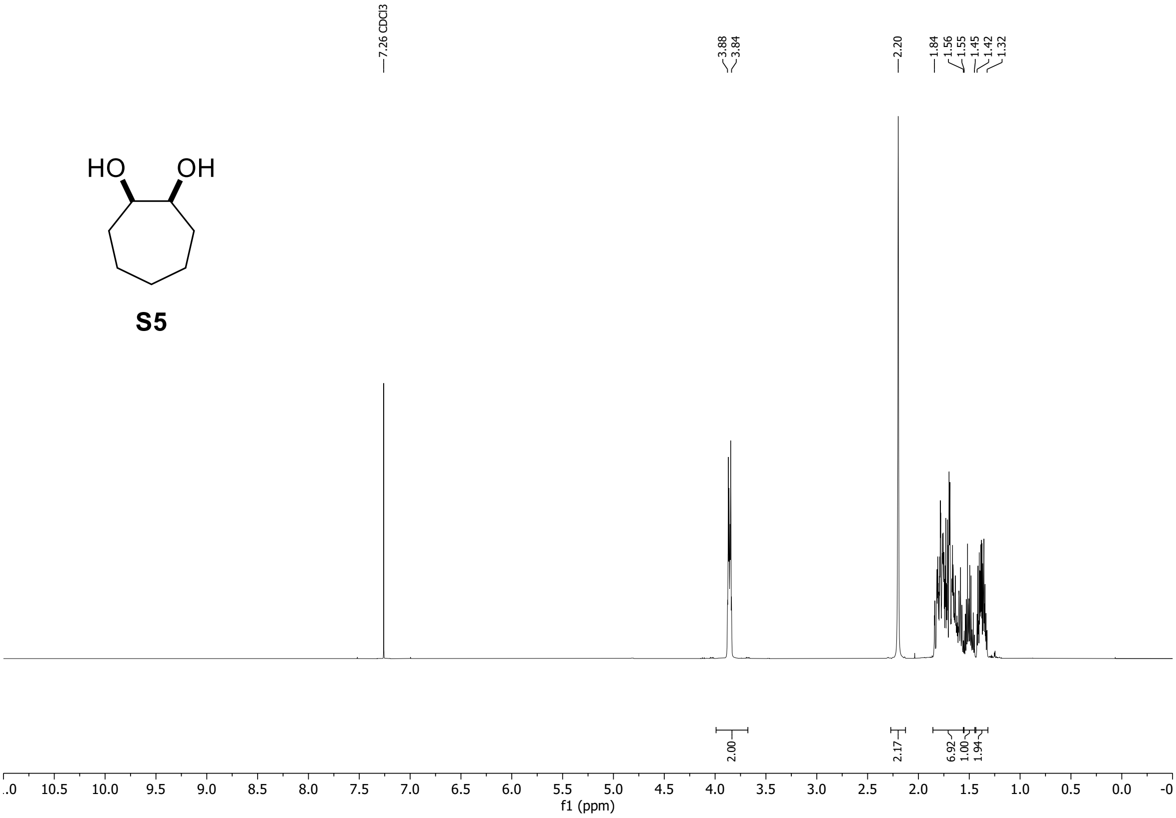

^{1}H NMR (400 MHz, $CDCl_3$) spectrum of compound **S5**.

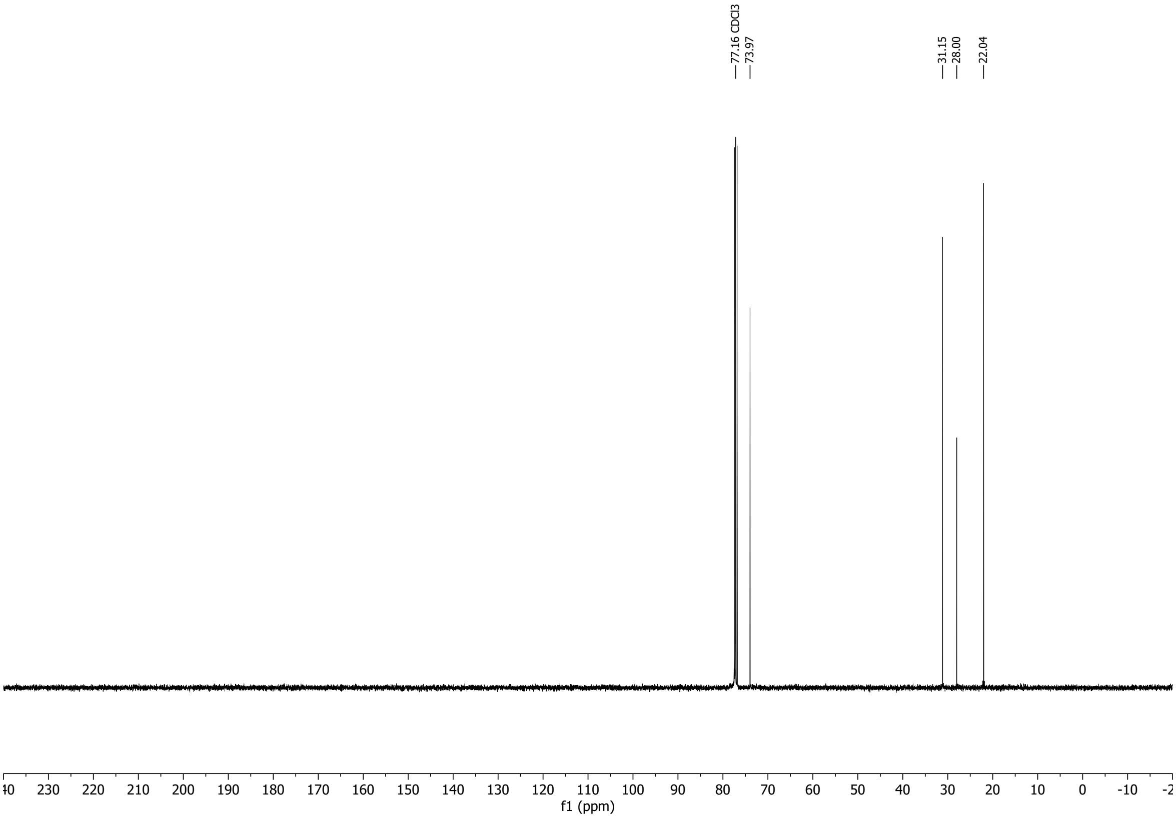

^{13}C NMR (100 MHz, $CDCl_3$) spectrum of compound **S5**.

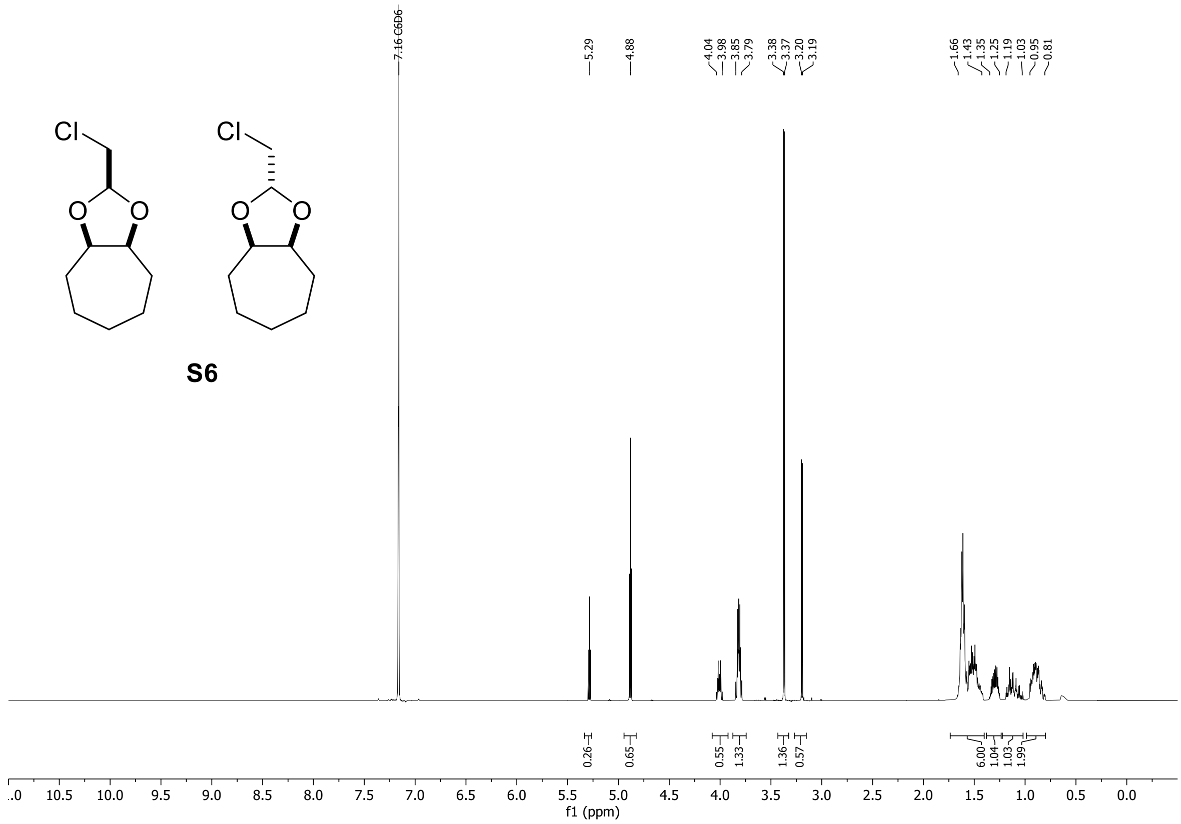

^{1}H NMR (400 MHz, C_6D_6) spectrum of compound **S6**

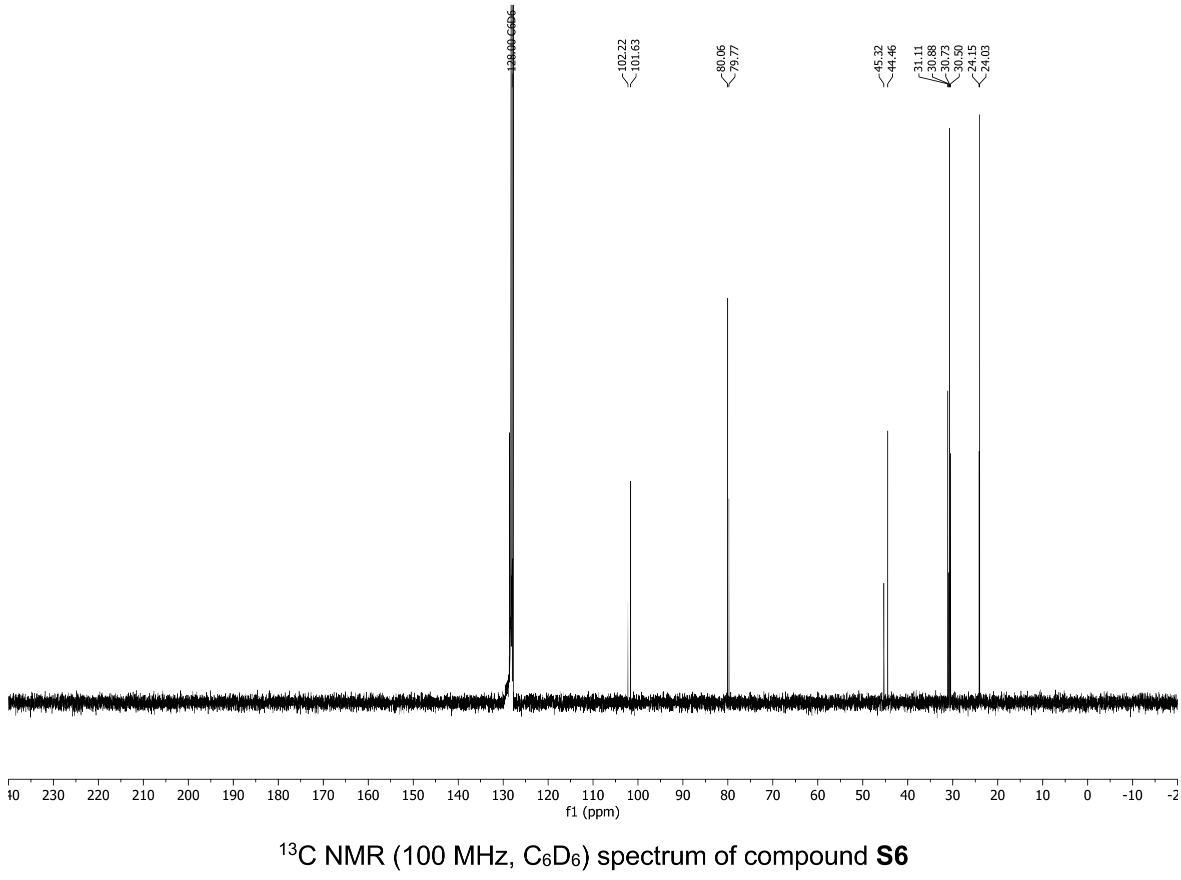

^{13}C NMR (100 MHz, C_6D_6) spectrum of compound **S6**

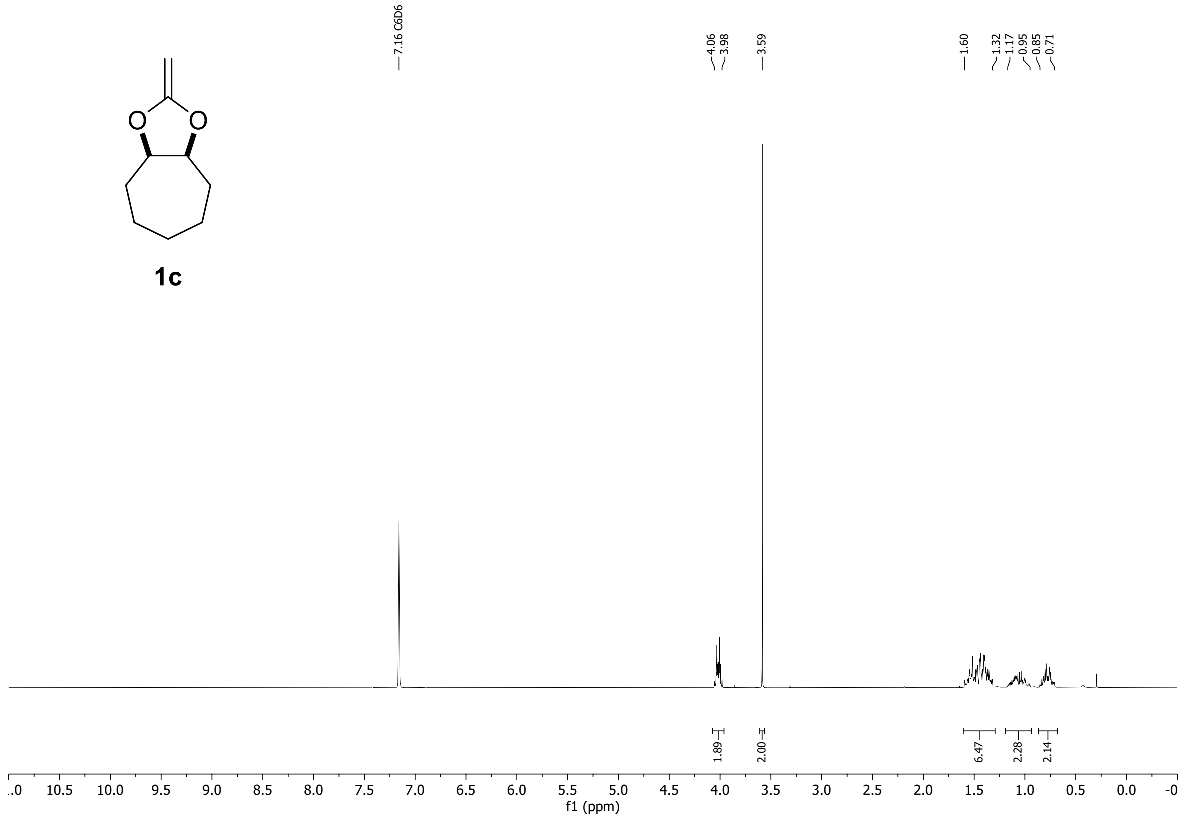

1c

^{1}H NMR (300 MHz, C_6D_6) spectrum of compound **1c**.

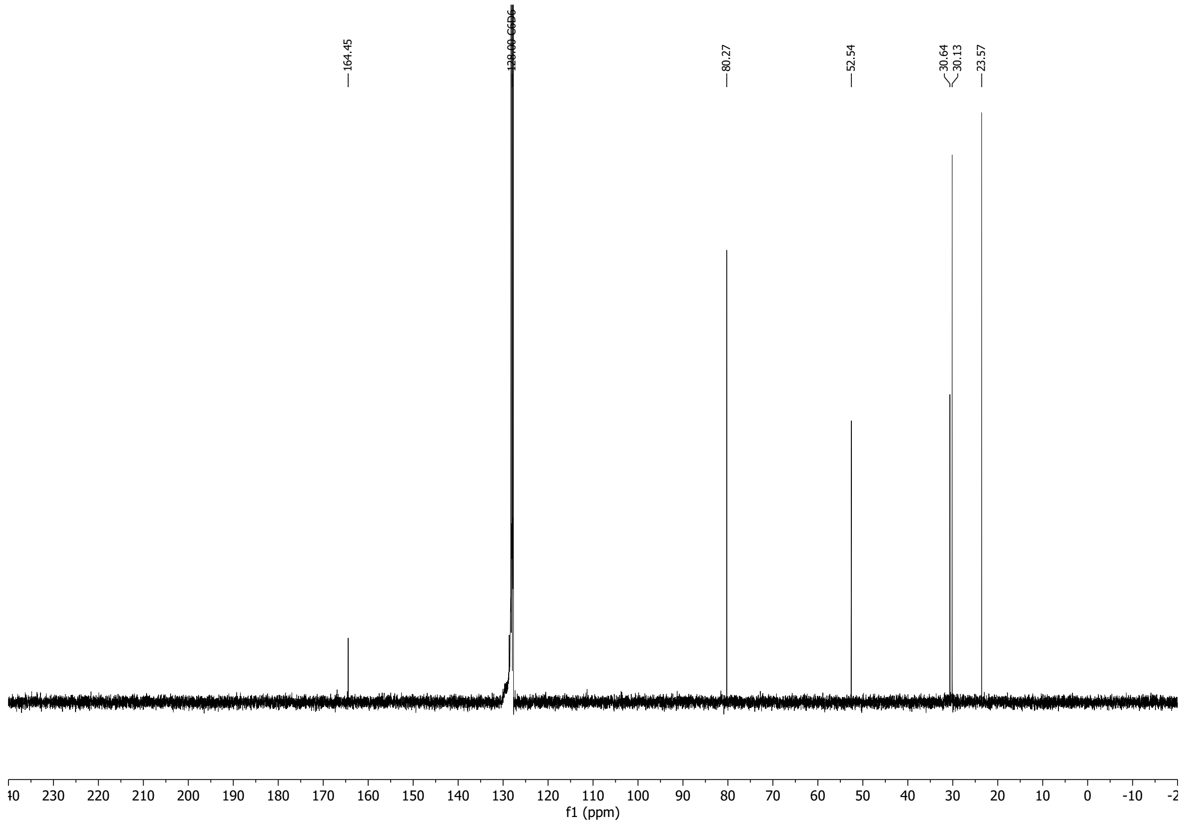

^{13}C NMR (100 MHz, C_6D_6) spectrum of compound **1c**.

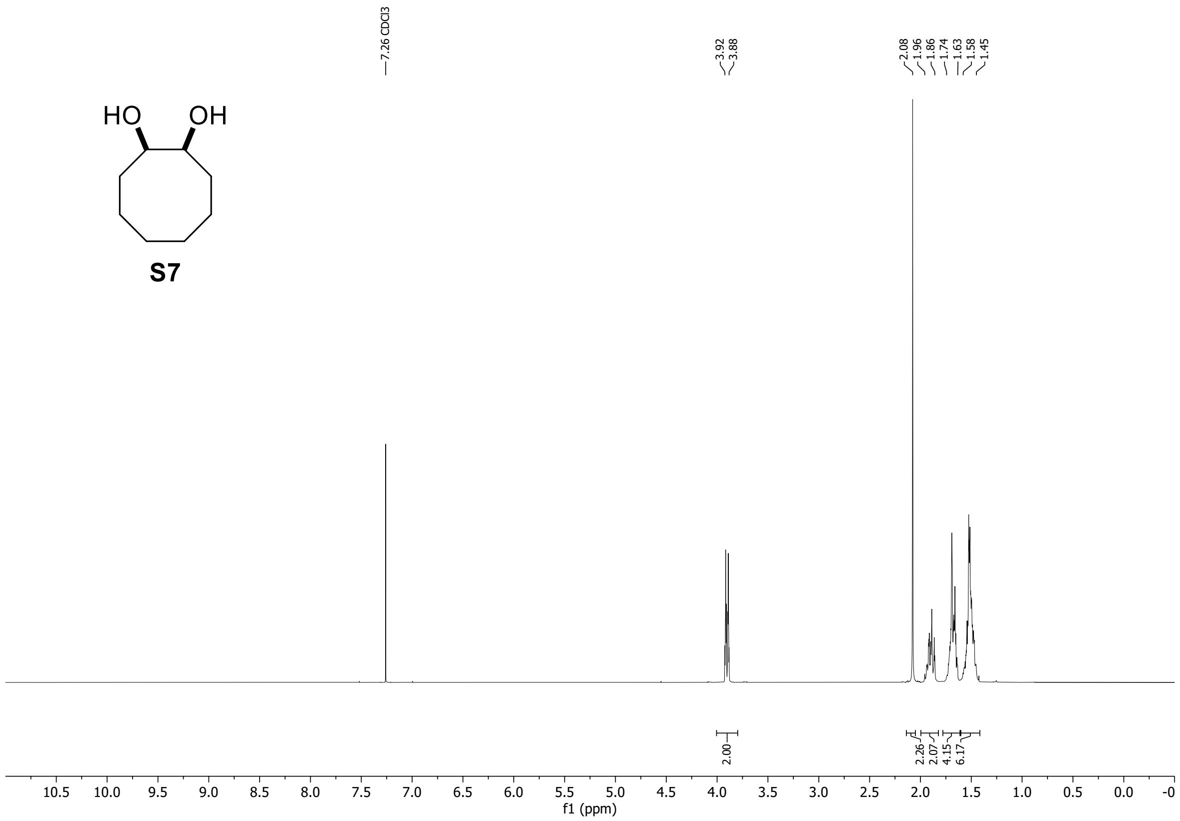

^{1}H NMR (400 MHz, $CDCl_3$) spectrum of compound **S7**.

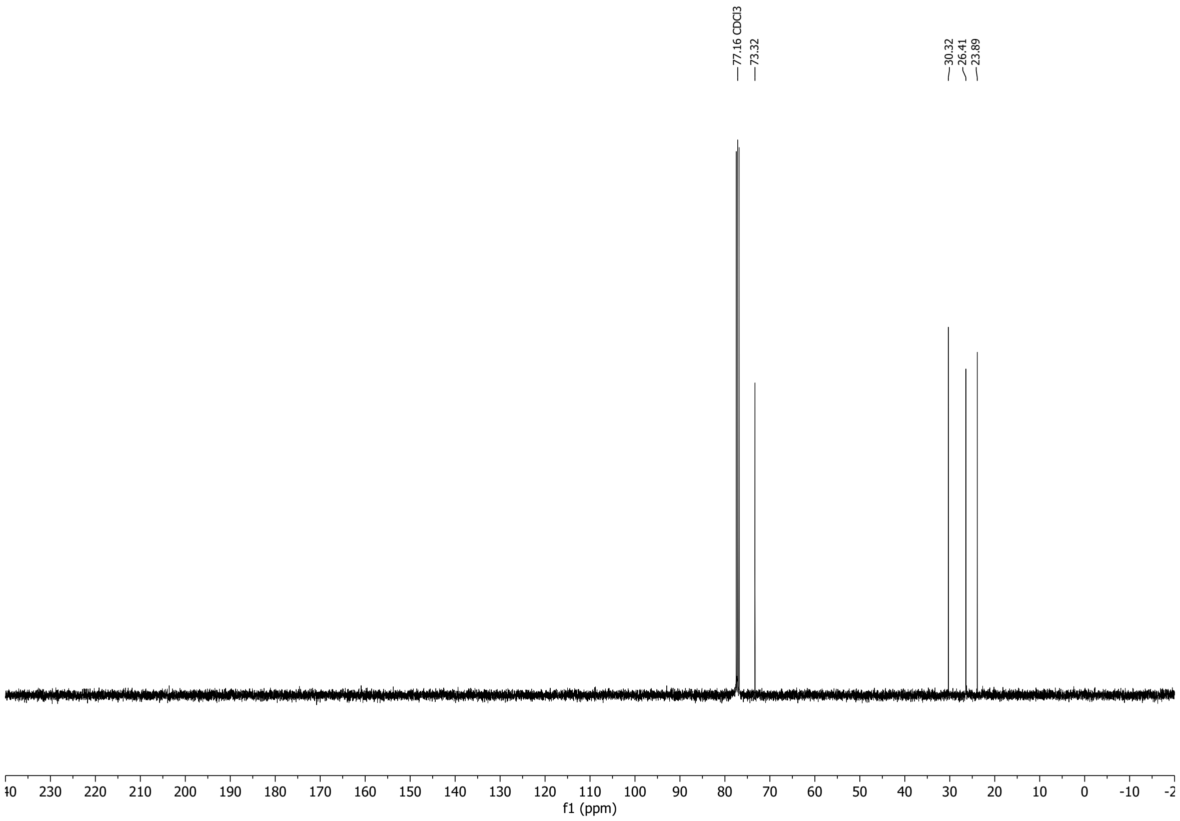

^{13}C NMR (100 MHz, $CDCl_3$) spectrum of compound **S7**.

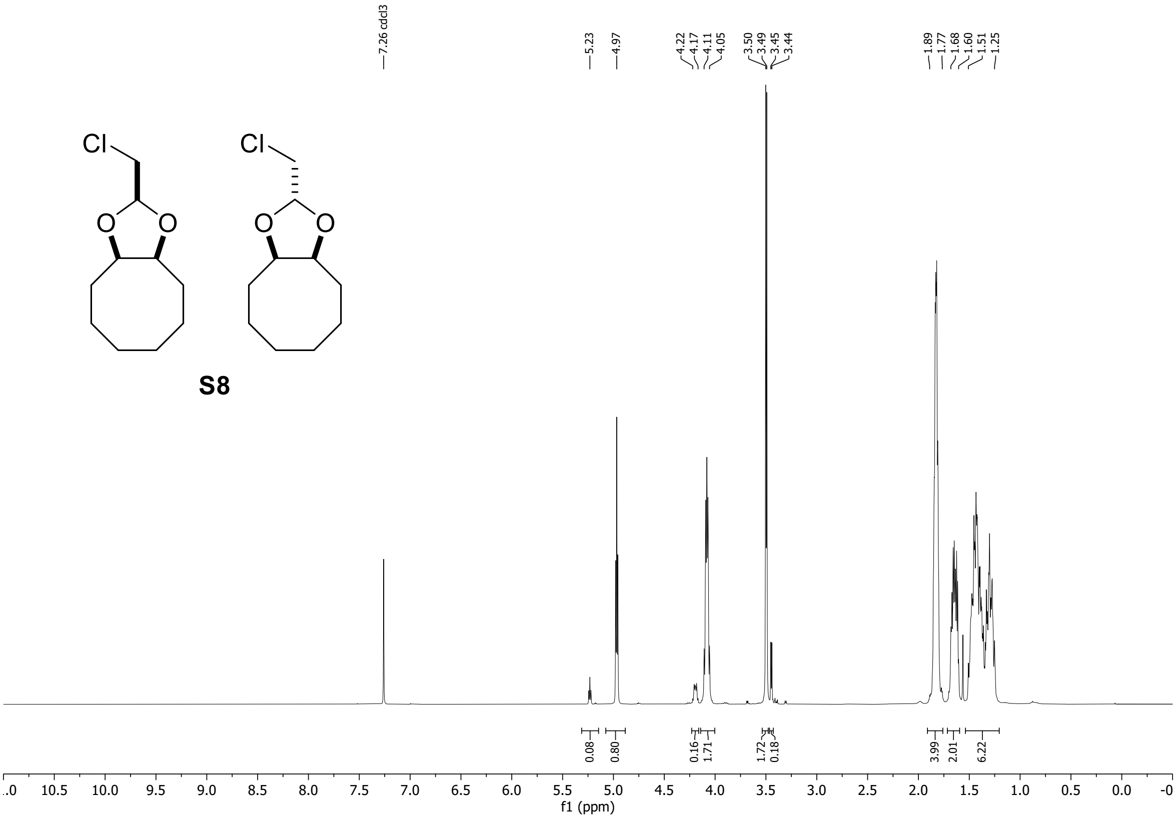

^{1}H NMR (500 MHz, C_6D_6) spectrum of compound **S8**.

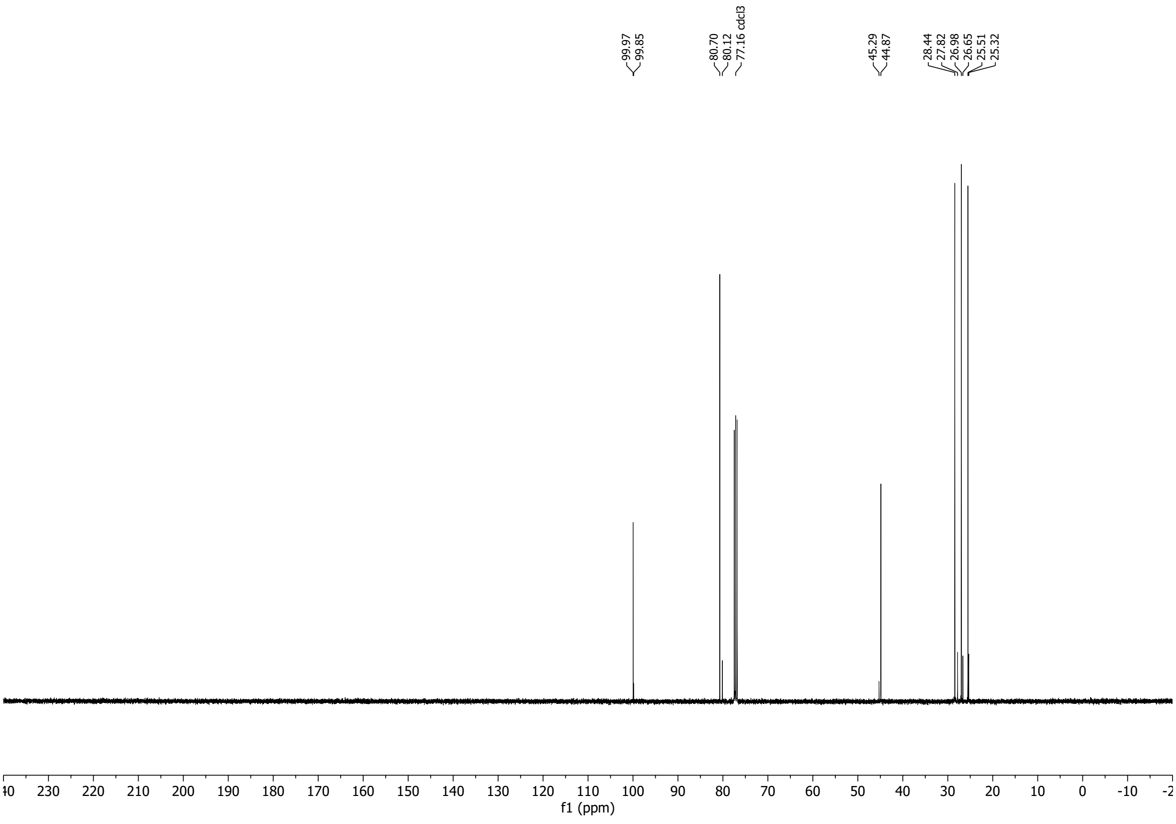

^{13}C NMR (125 MHz, C_6D_6) spectrum of compound **S8**.

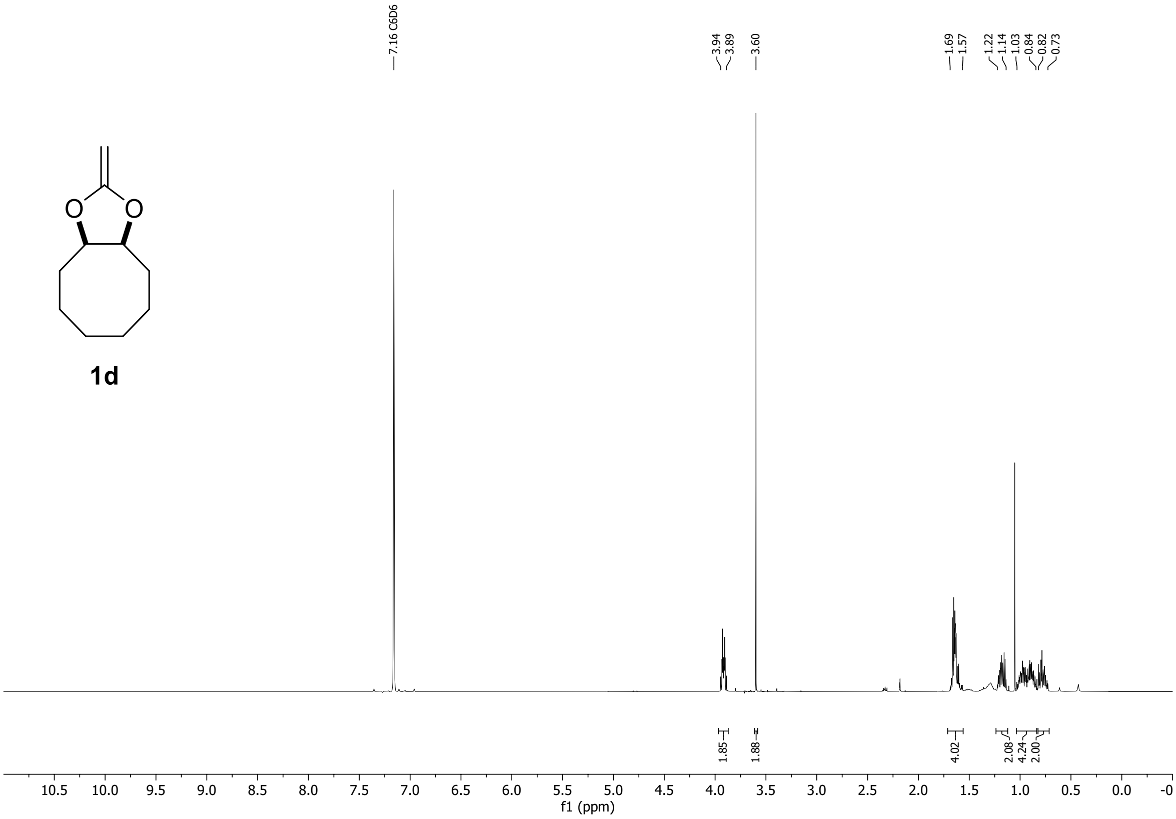

1d

^{1}H NMR (400 MHz, C_6D_6) spectrum of compound **1d**.

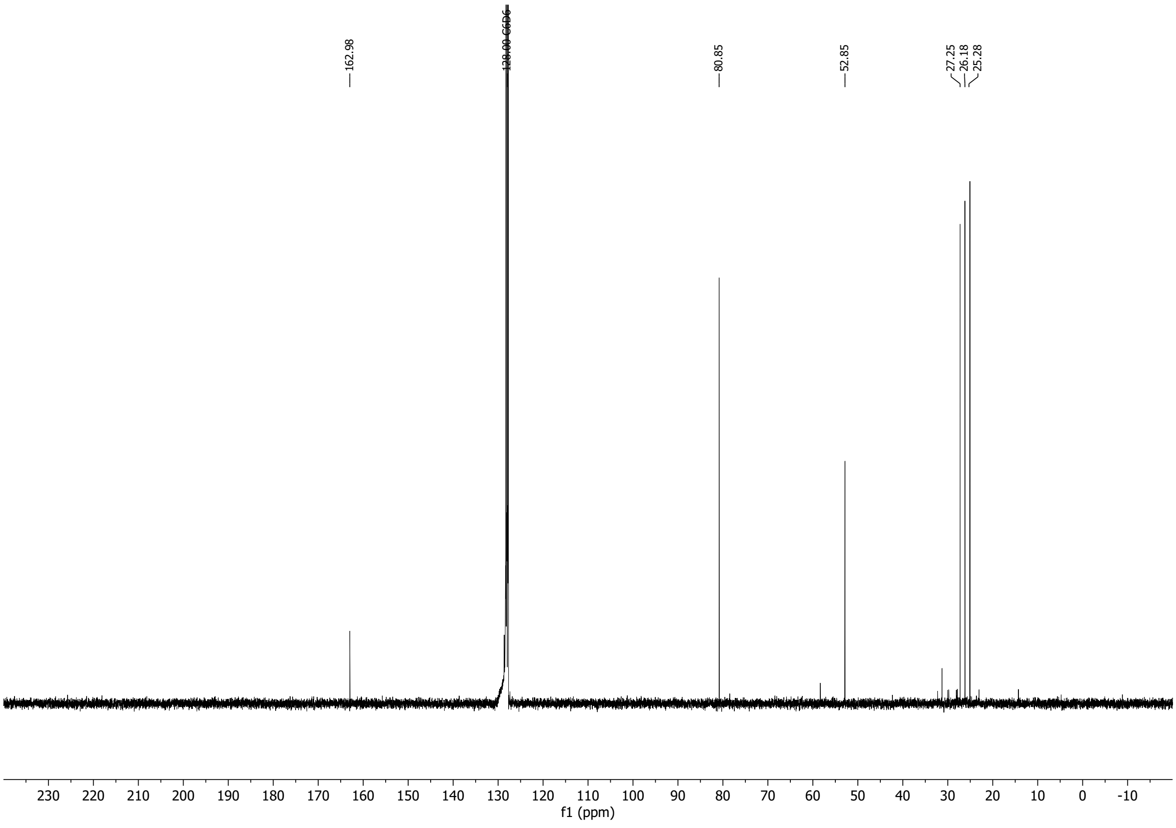

^{13}C NMR (100 MHz, C_6D_6) spectrum of compound **1d**.

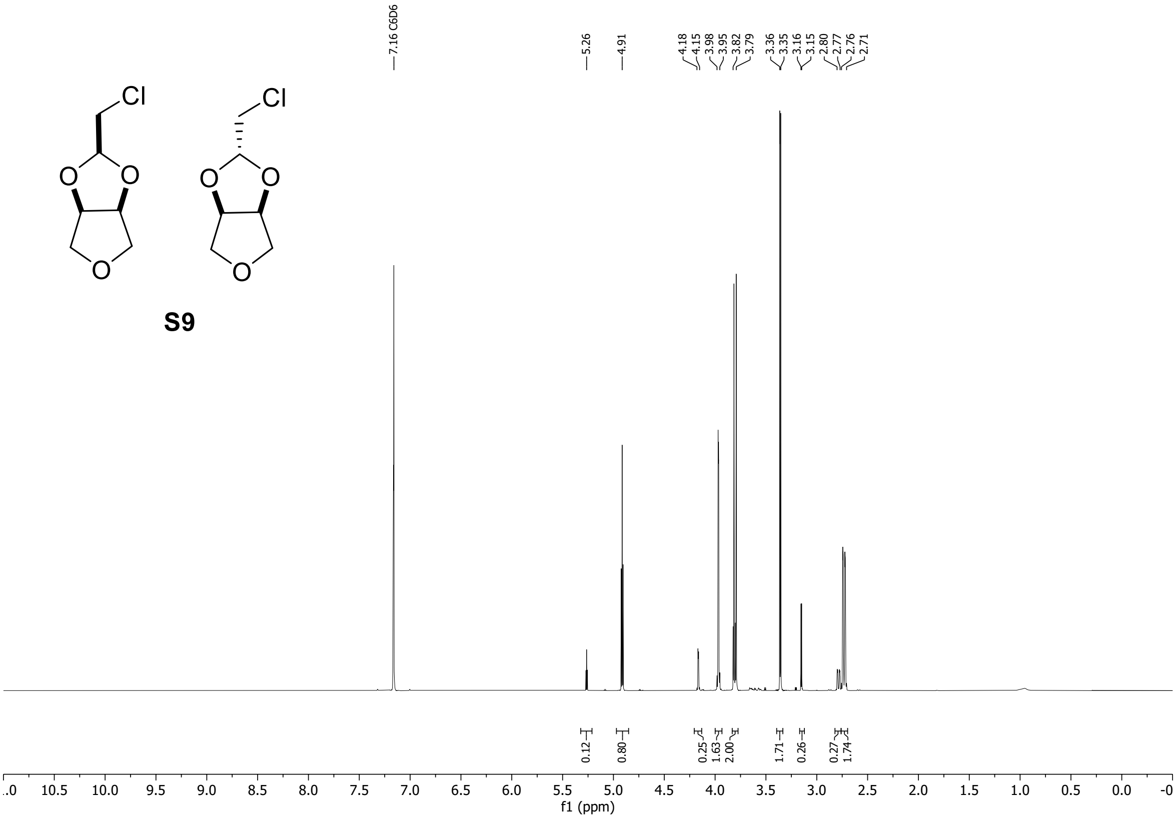

^{1}H NMR (500 MHz, C_6D_6) spectrum of compound **S9**.

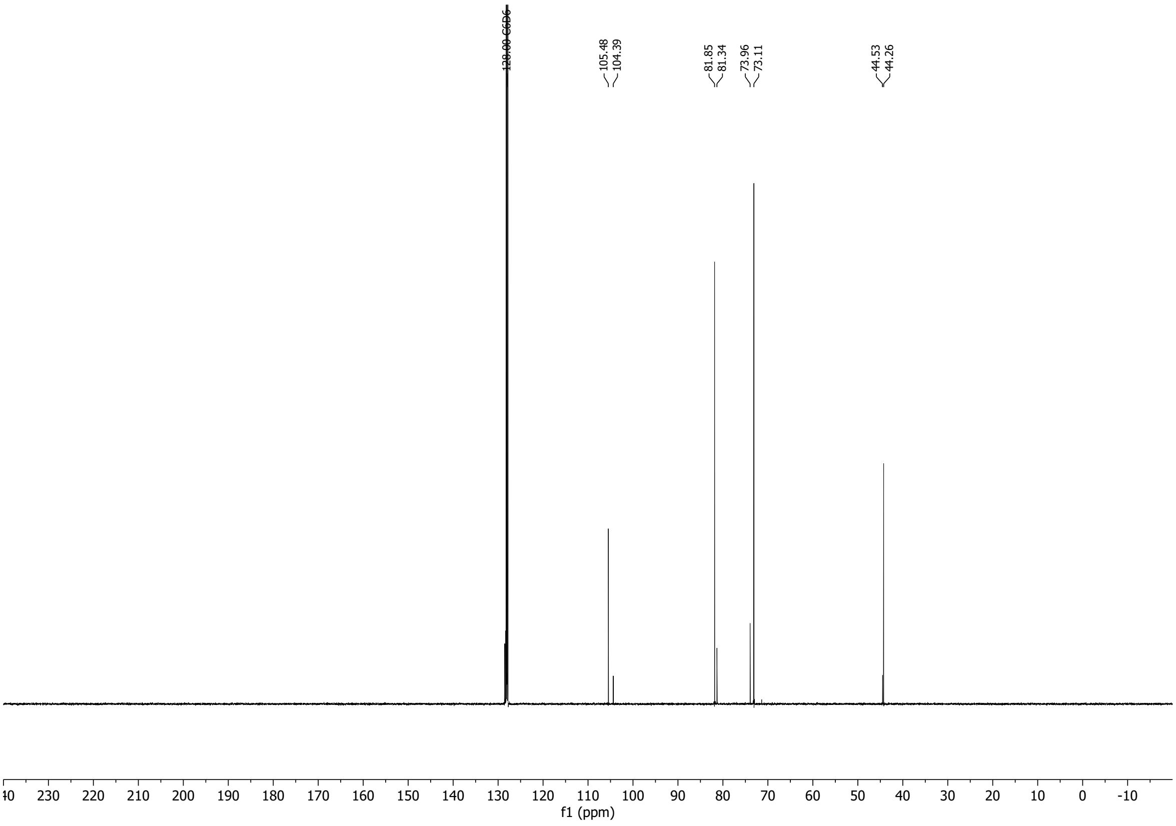

^{13}C NMR (125 MHz, C_6D_6) spectrum of compound **S9**.

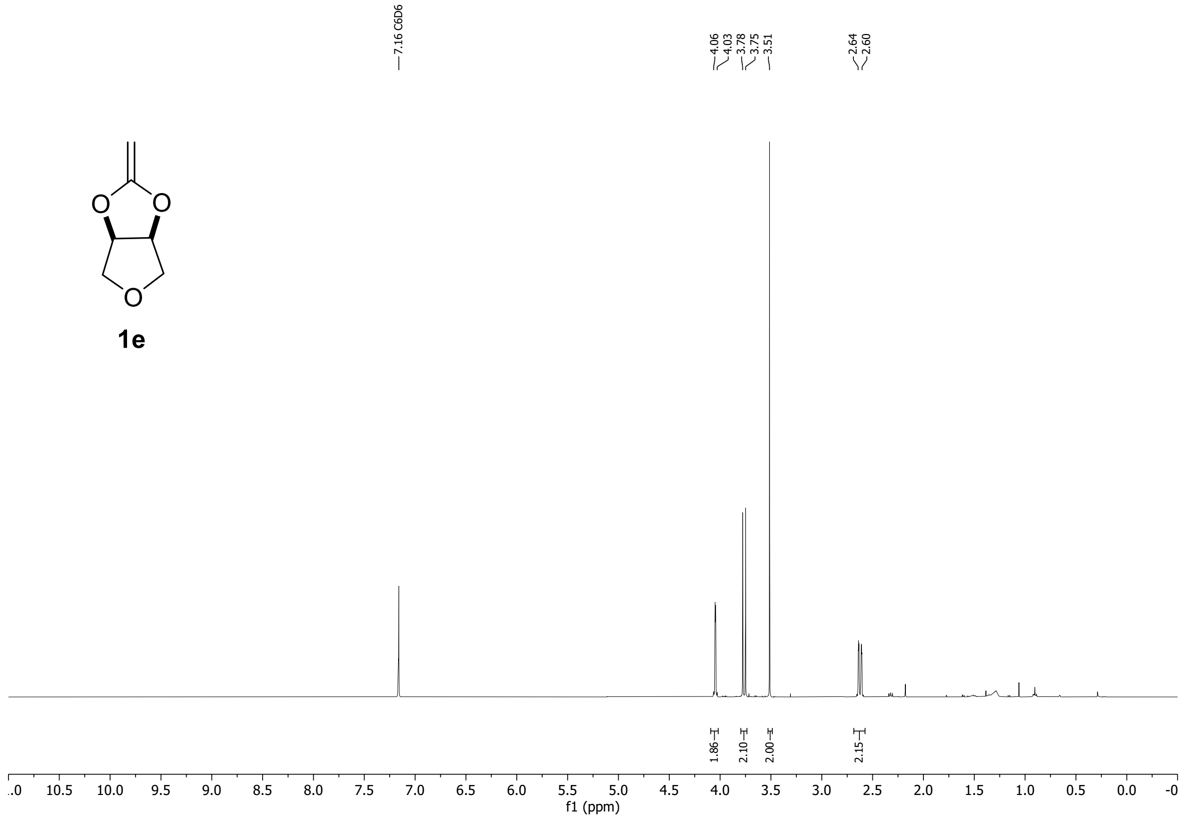

1e

^{1}H NMR (400 MHz, C_6D_6) spectrum of compound **1e**.

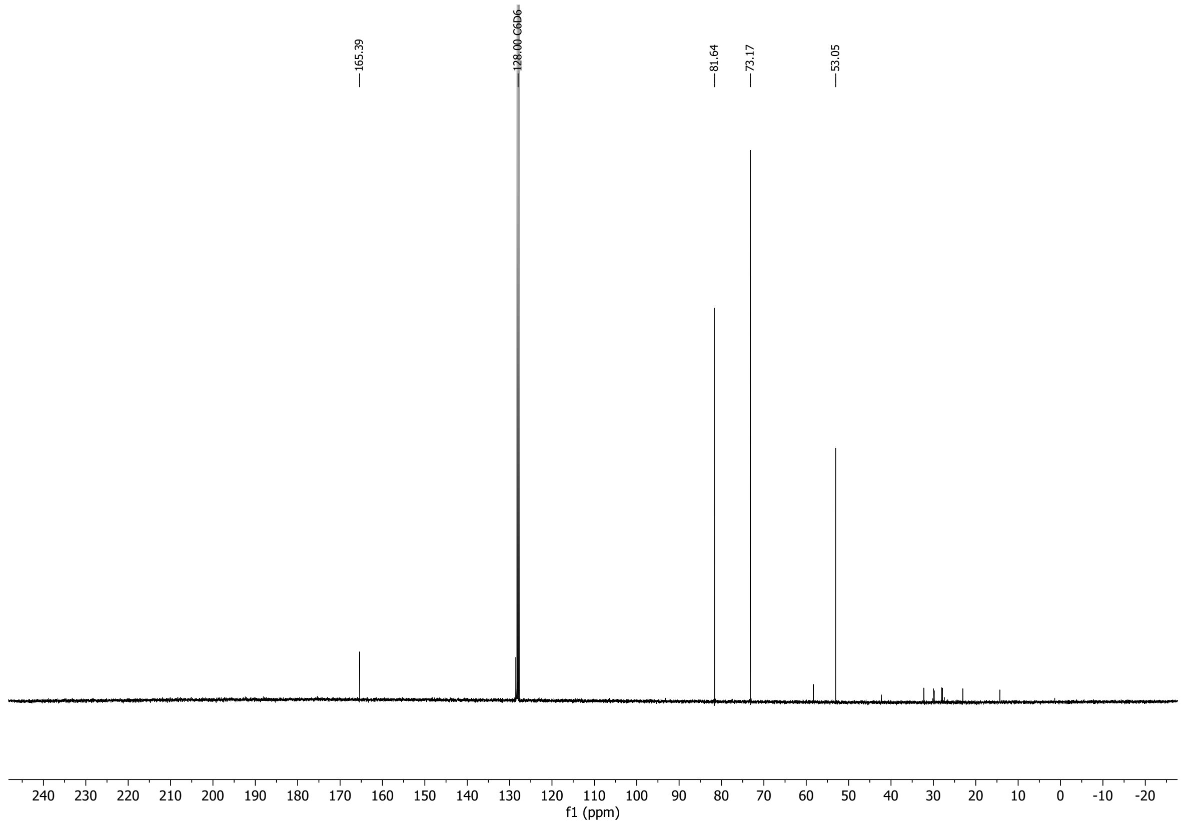

^{13}C NMR (100 MHz, C_6D_6) spectrum of compound **1e**.

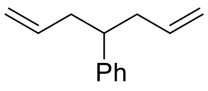

S10

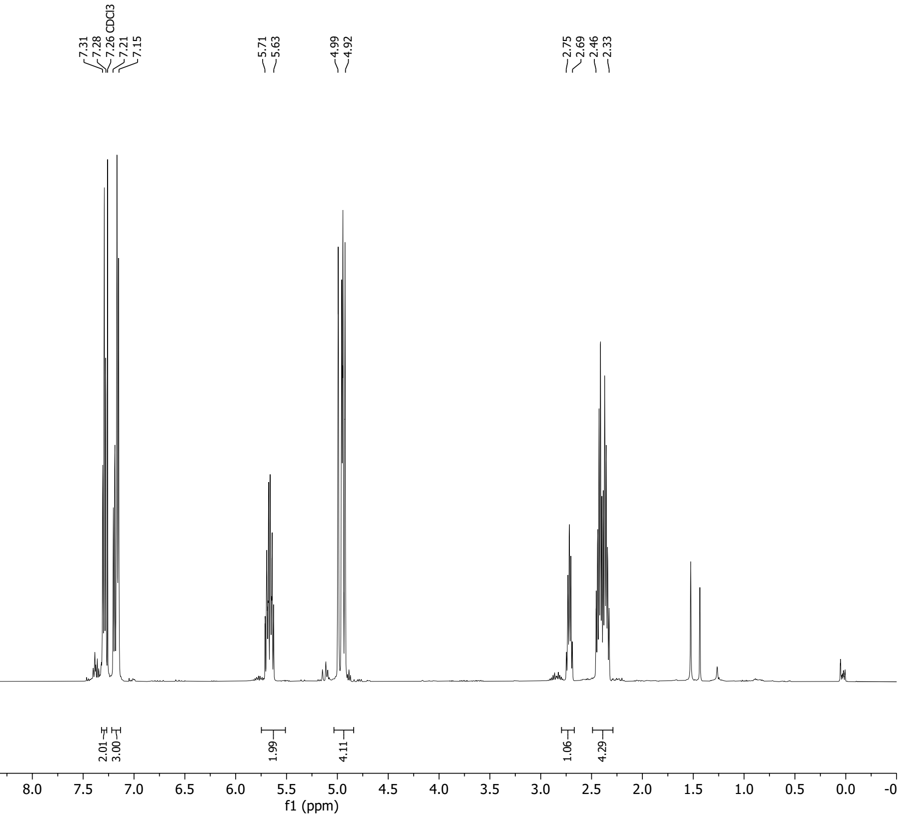

^{1}H NMR (500 MHz, $CDCl_3$) spectrum of compound **S10**.

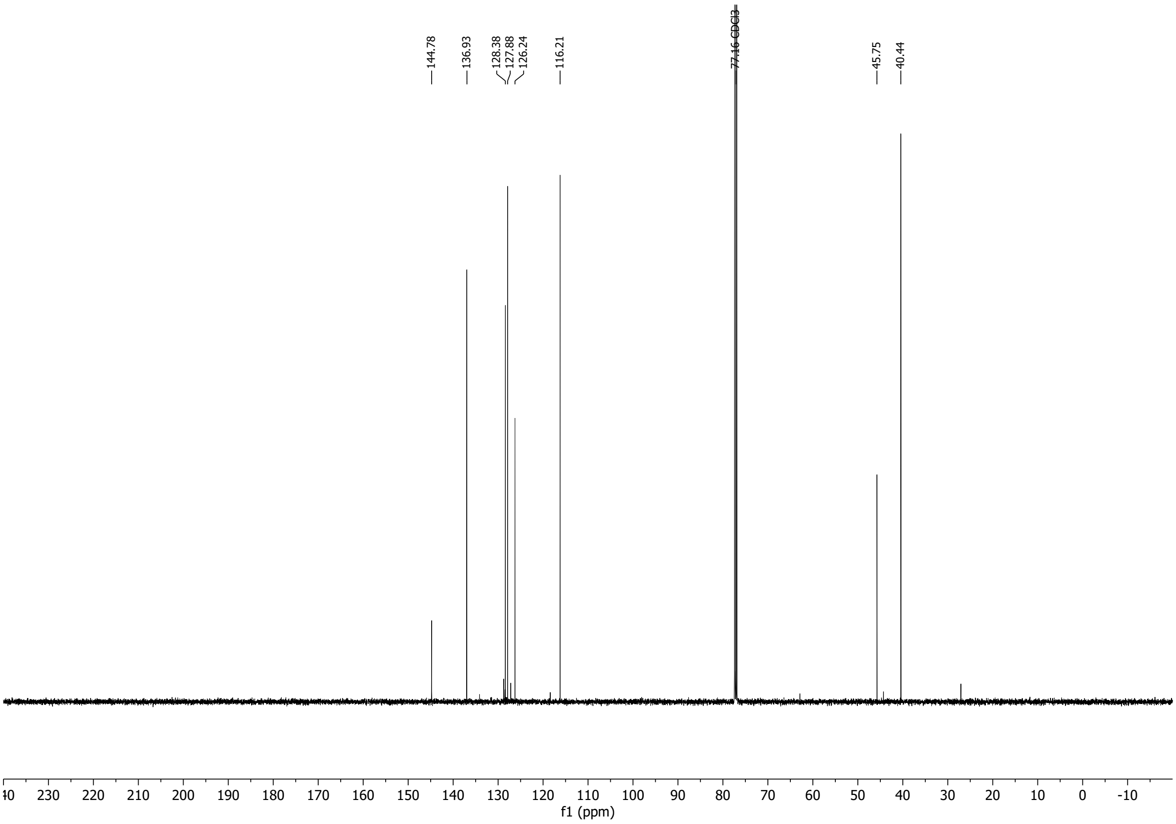

^{13}C NMR (125 MHz, $CDCl_3$) spectrum of compound **S10**.

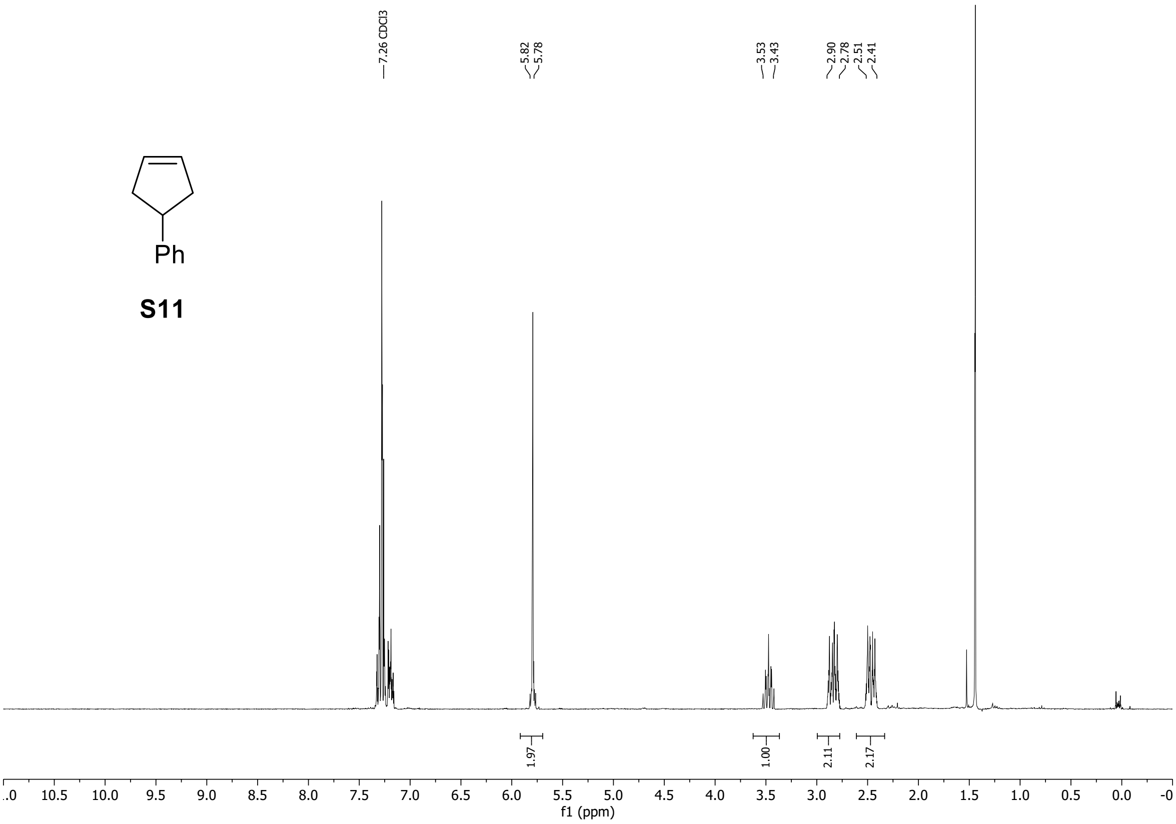

^{1}H NMR (300 MHz, $CDCl_3$) spectrum of compound **S11**.

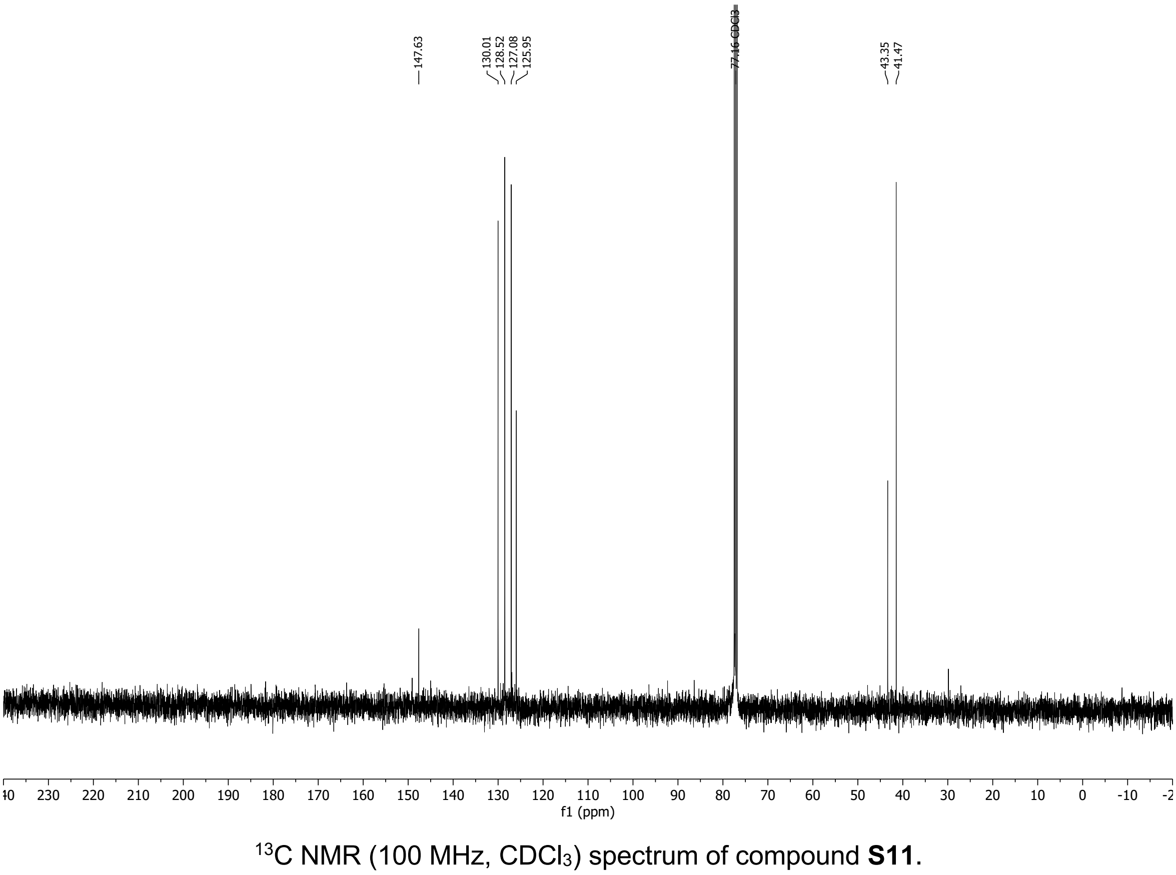

^{13}C NMR (100 MHz, $CDCl_3$) spectrum of compound **S11**.

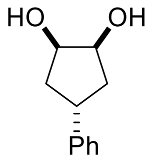

S12

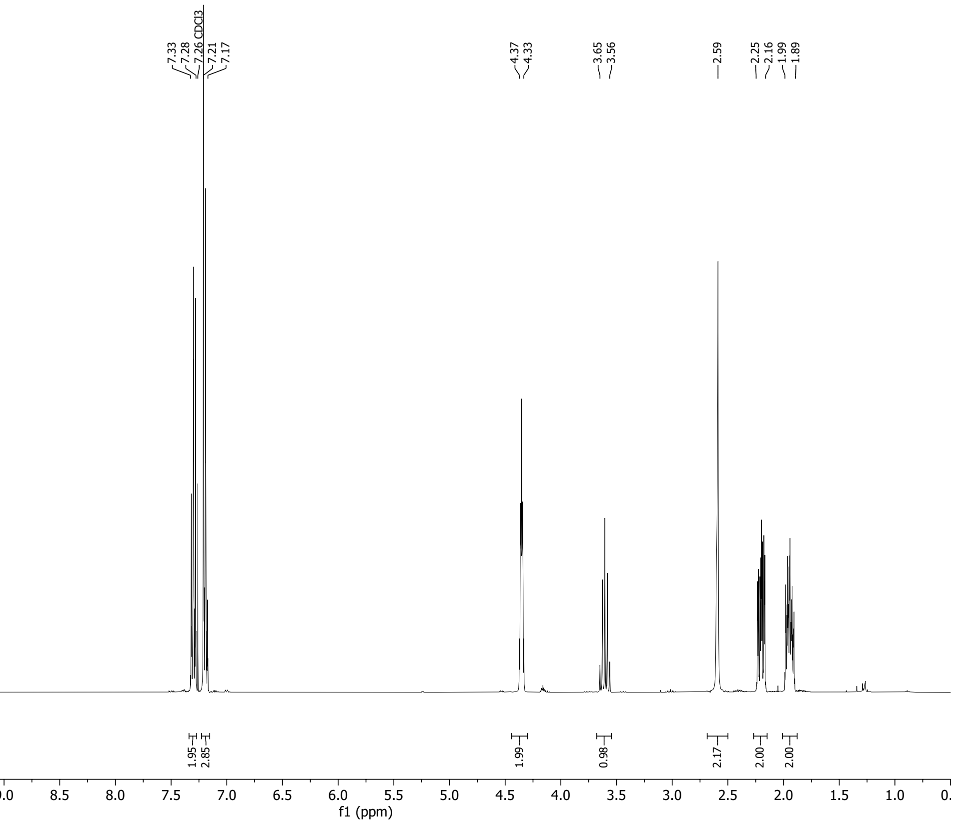

^{1}H NMR (400 MHz, $CDCl_3$) spectrum of compound **S12**.

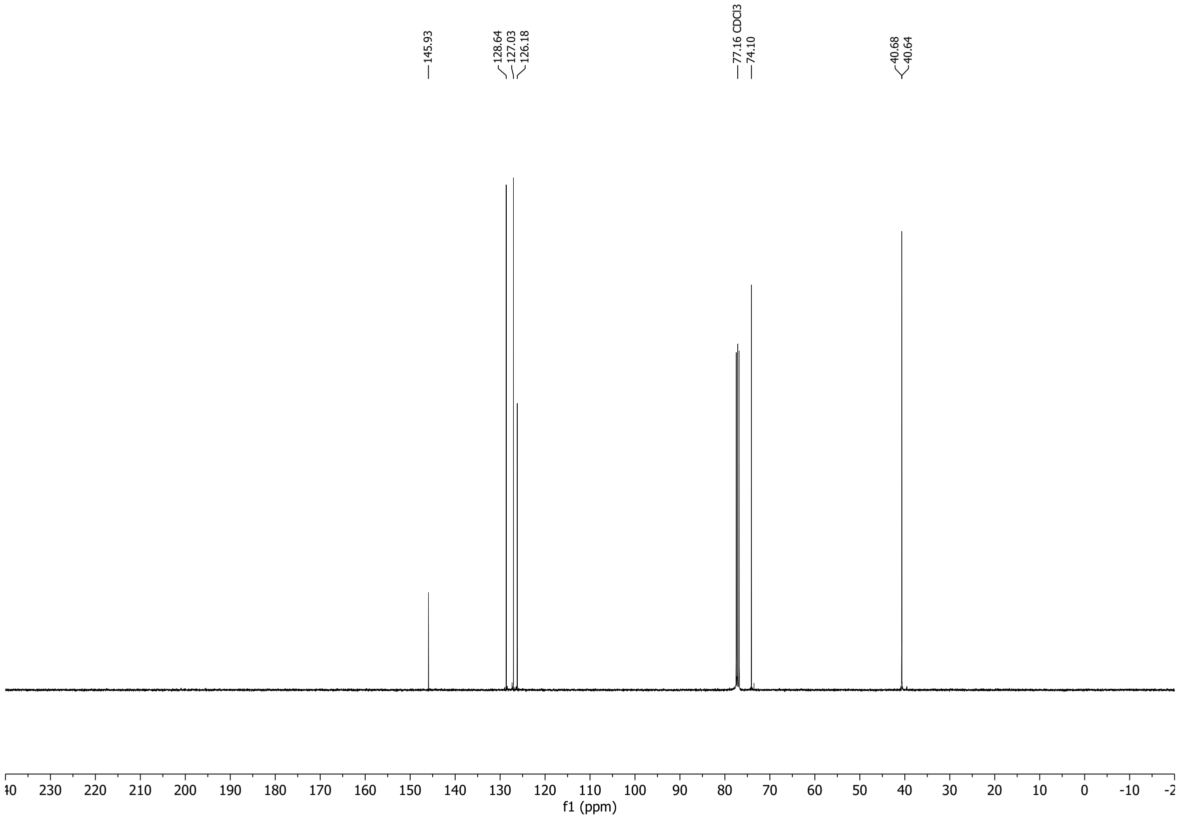

^{13}C NMR (100 MHz, $CDCl_3$) spectrum of compound **S12**.

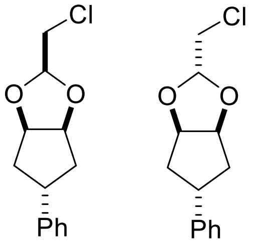

S13

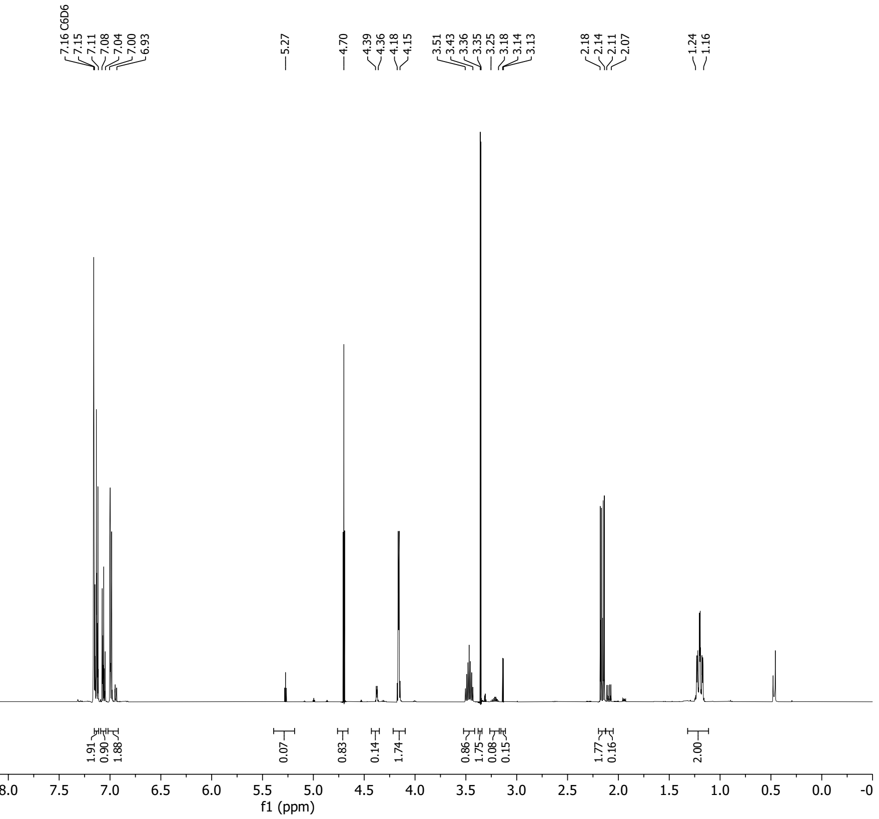

^{1}H NMR (500 MHz, C_6D_6) spectrum of compound **S13**.

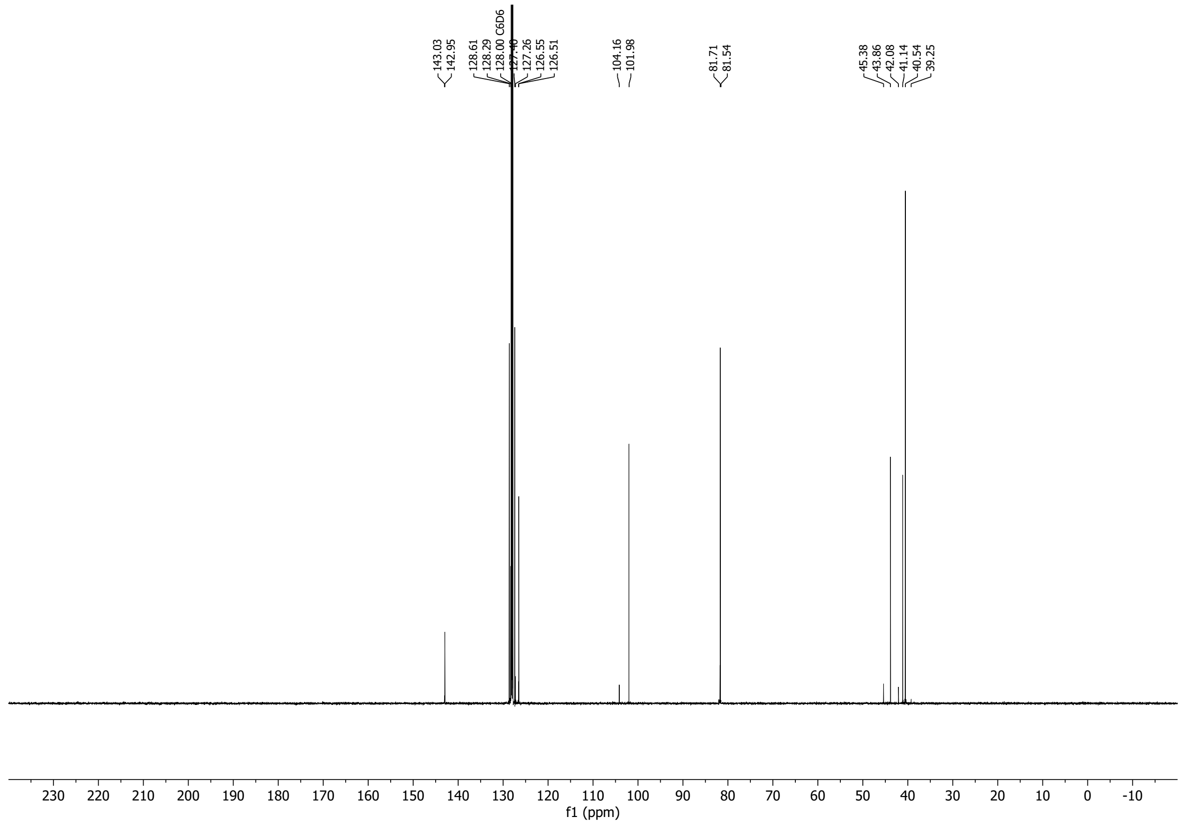

^{13}C NMR (125 MHz, C_6D_6) spectrum of compound **S13**.

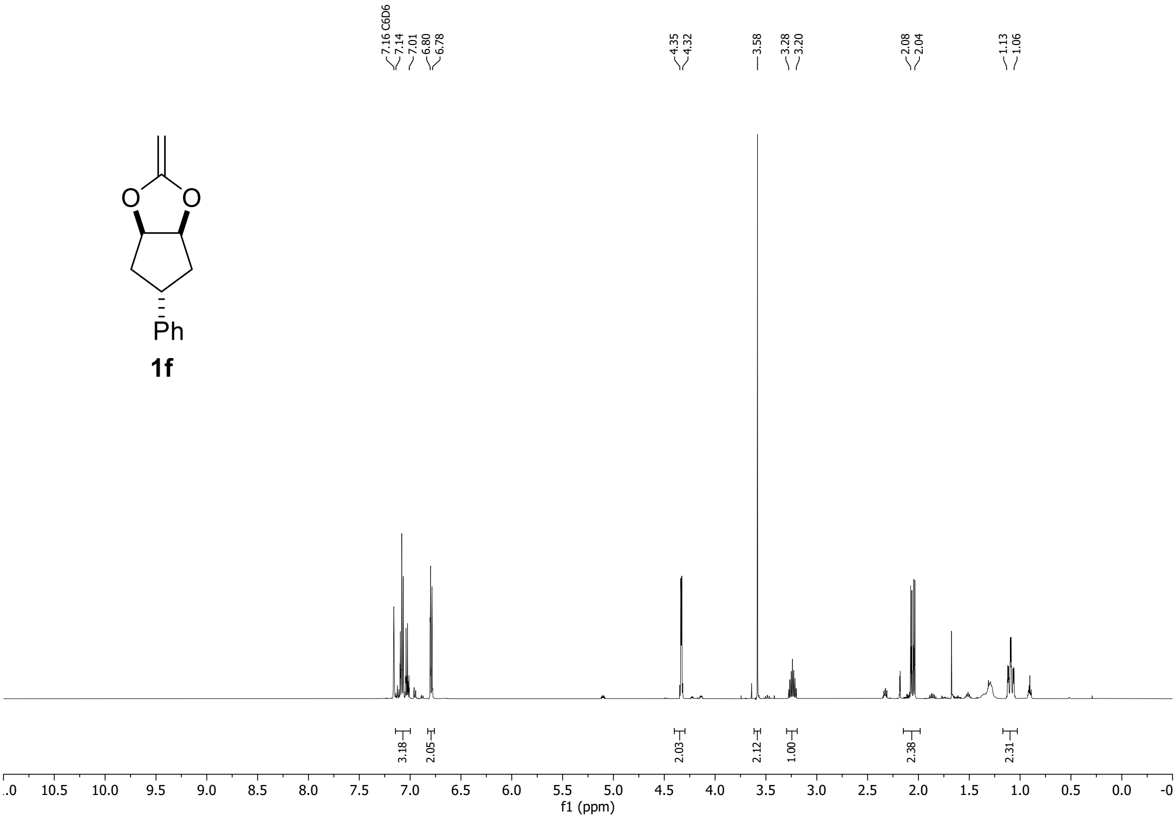

^{1}H NMR (500 MHz, C_6D_6) spectrum of compound **1f**.

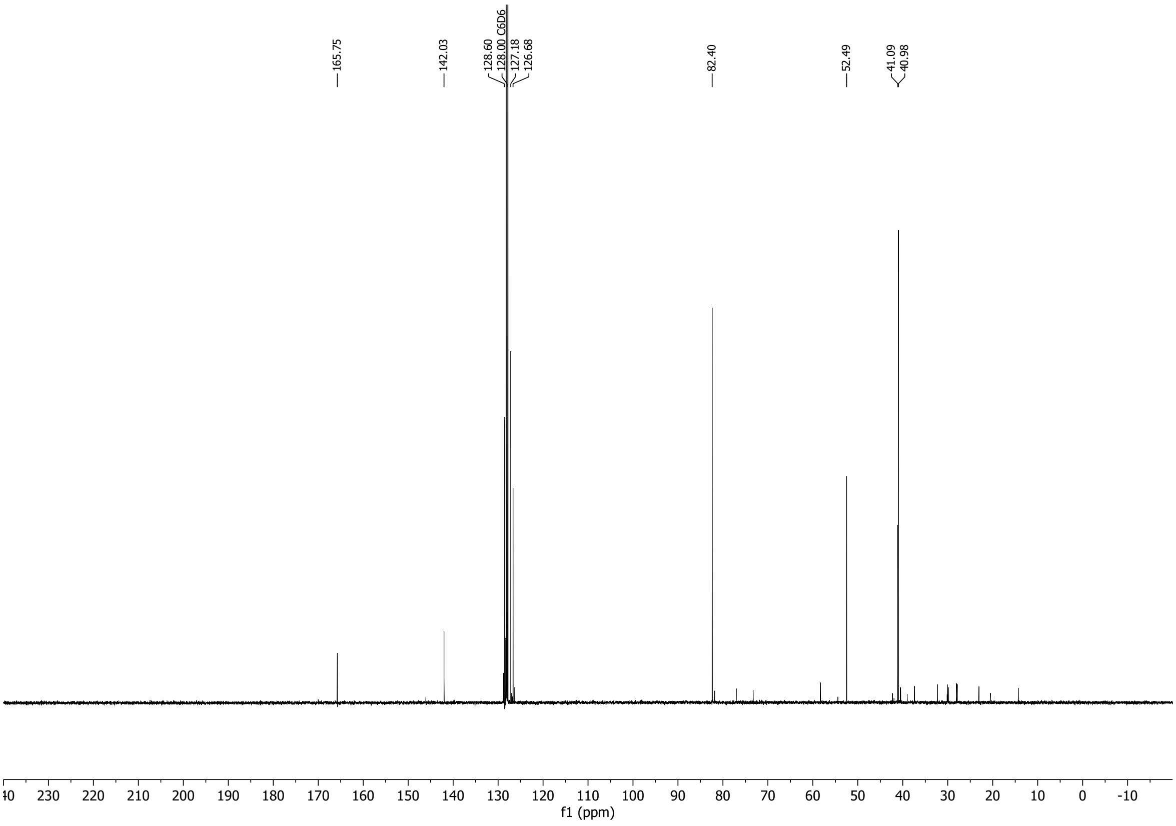

^{13}C NMR (125 MHz, C_6D_6) spectrum of compound **1f**.

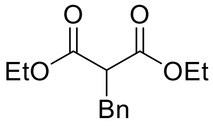

S14

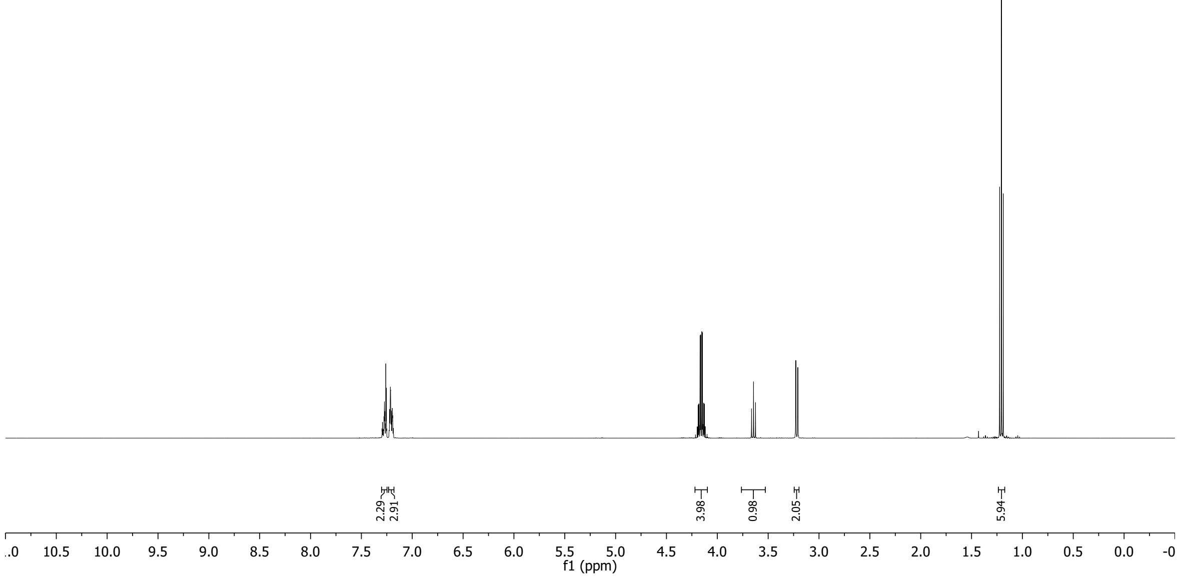

^{1}H NMR (400 MHz, $CDCl_3$) spectrum of compound **S14**.

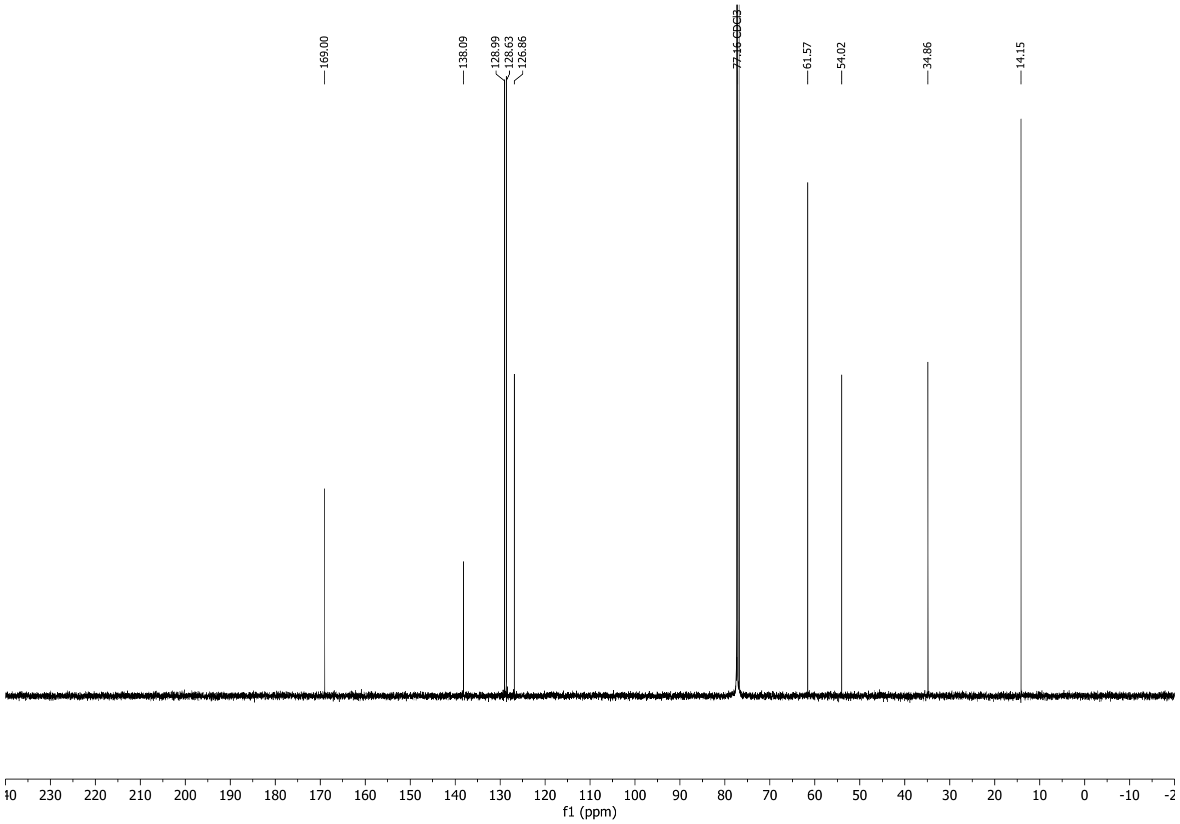

^{13}C NMR (100 MHz, $CDCl_3$) spectrum of compound **S14**.

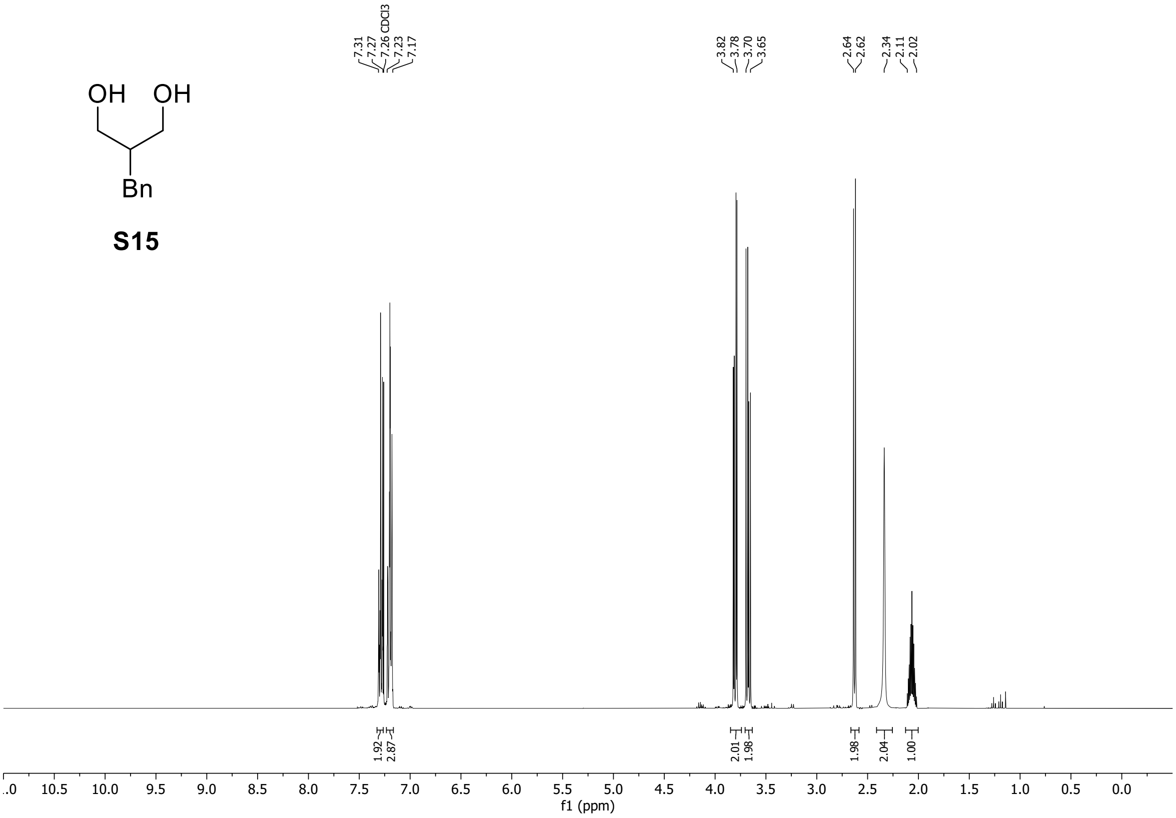

^{1}H NMR (400 MHz, $CDCl_3$) spectrum of compound **S15**.

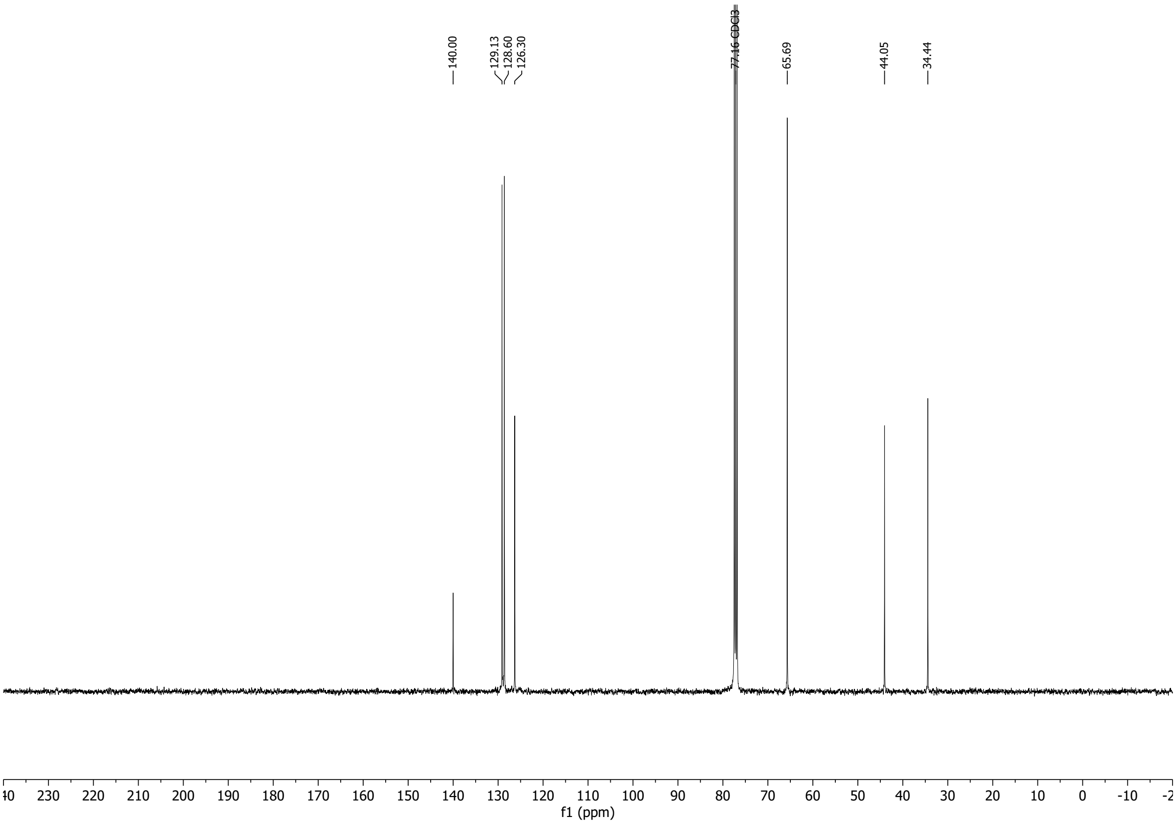

^{13}C NMR (100 MHz, $CDCl_3$) spectrum of compound **S15**.

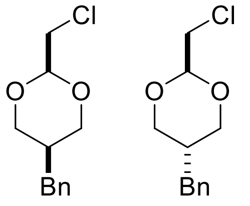

S16

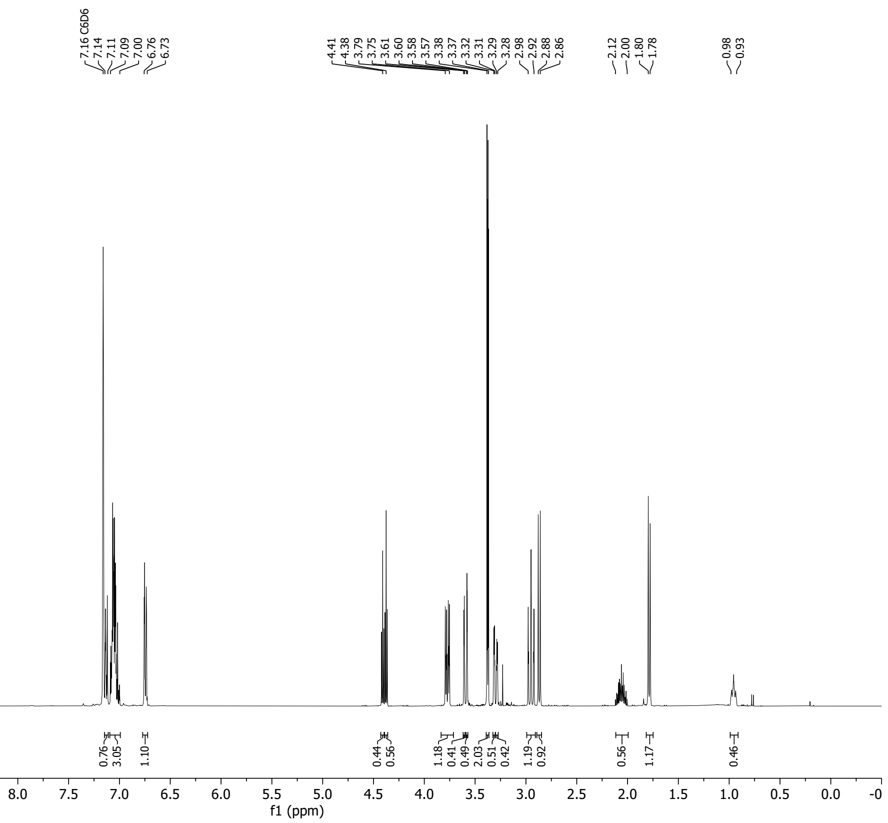

^{1}H NMR (400 MHz, C_6D_6) spectrum of compound **S16**.

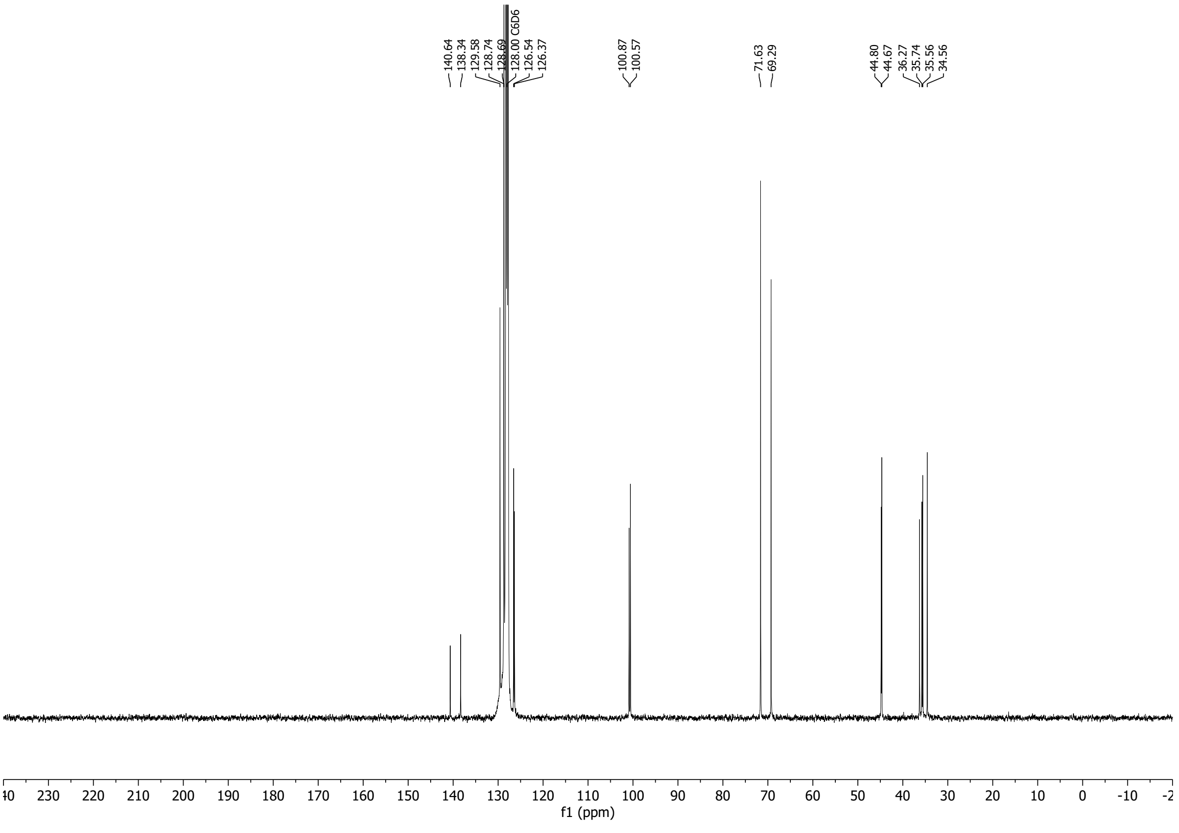

^{13}C NMR (100 MHz, C_6D_6) spectrum of compound **S16**.

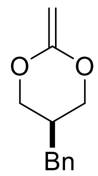

1g

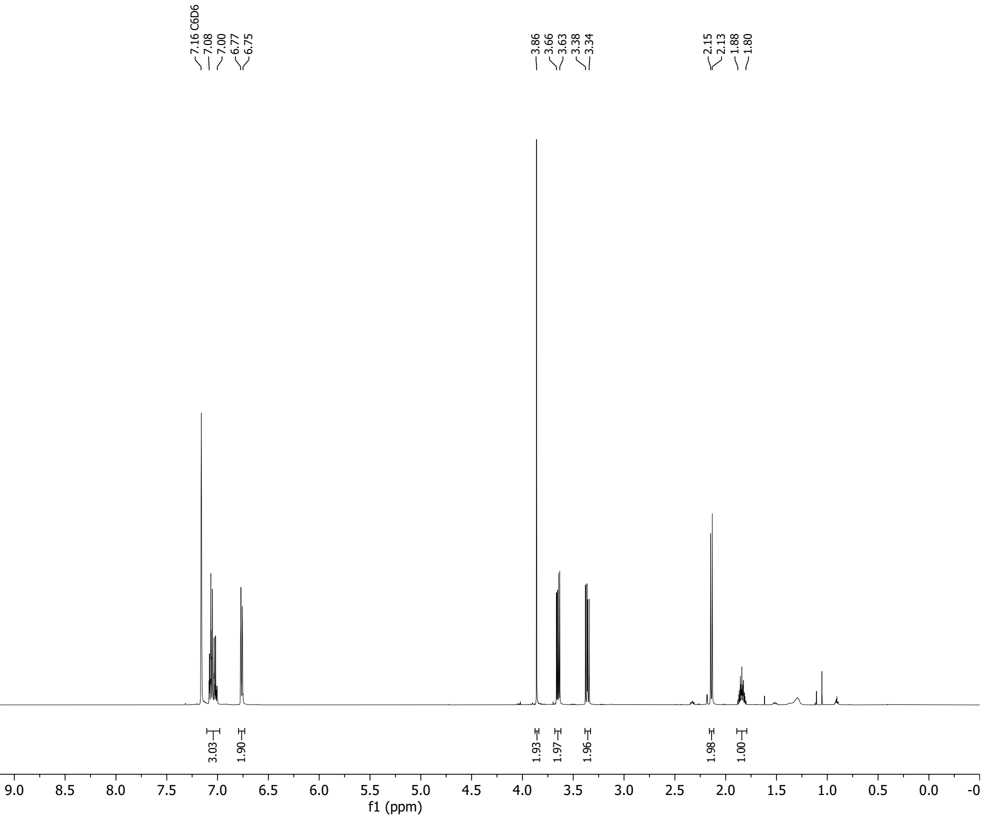

^{1}H NMR (400 MHz, C_6D_6) spectrum of compound **1g**.

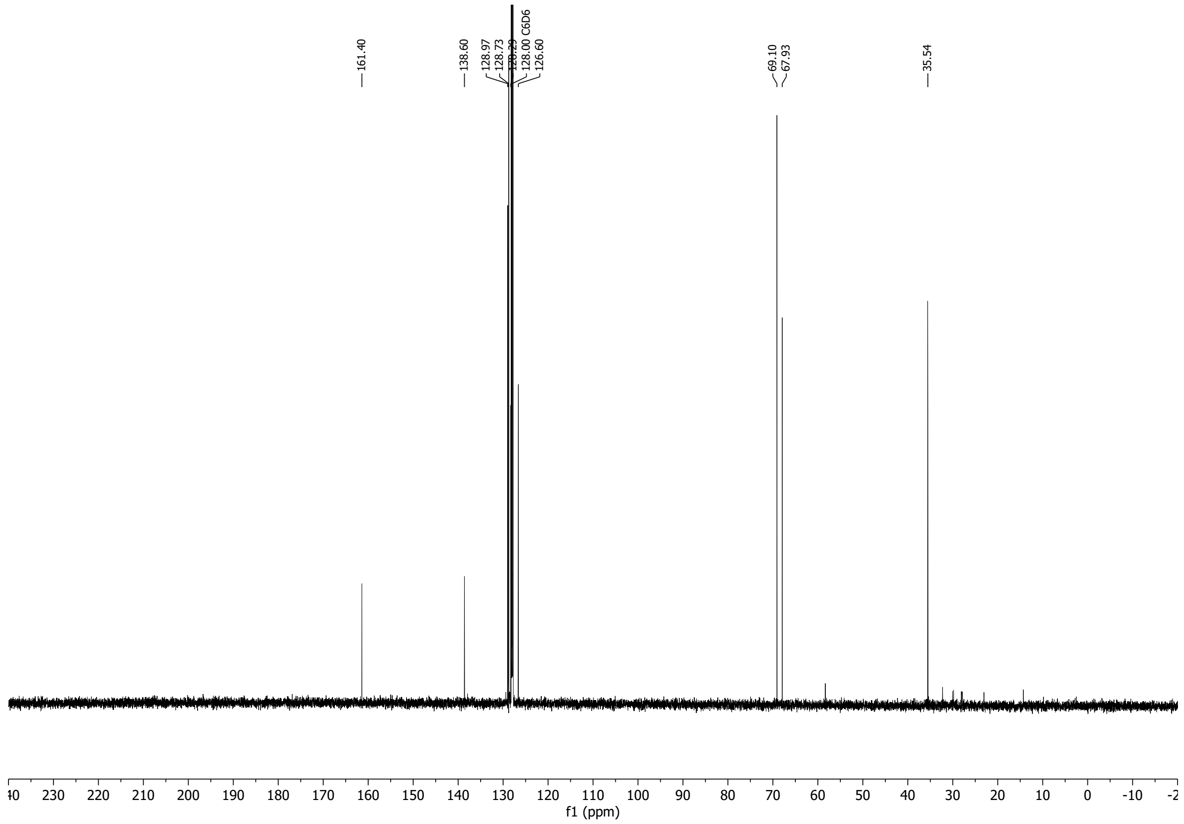

^{13}C NMR (100 MHz, C_6D_6) spectrum of compound **1g**.

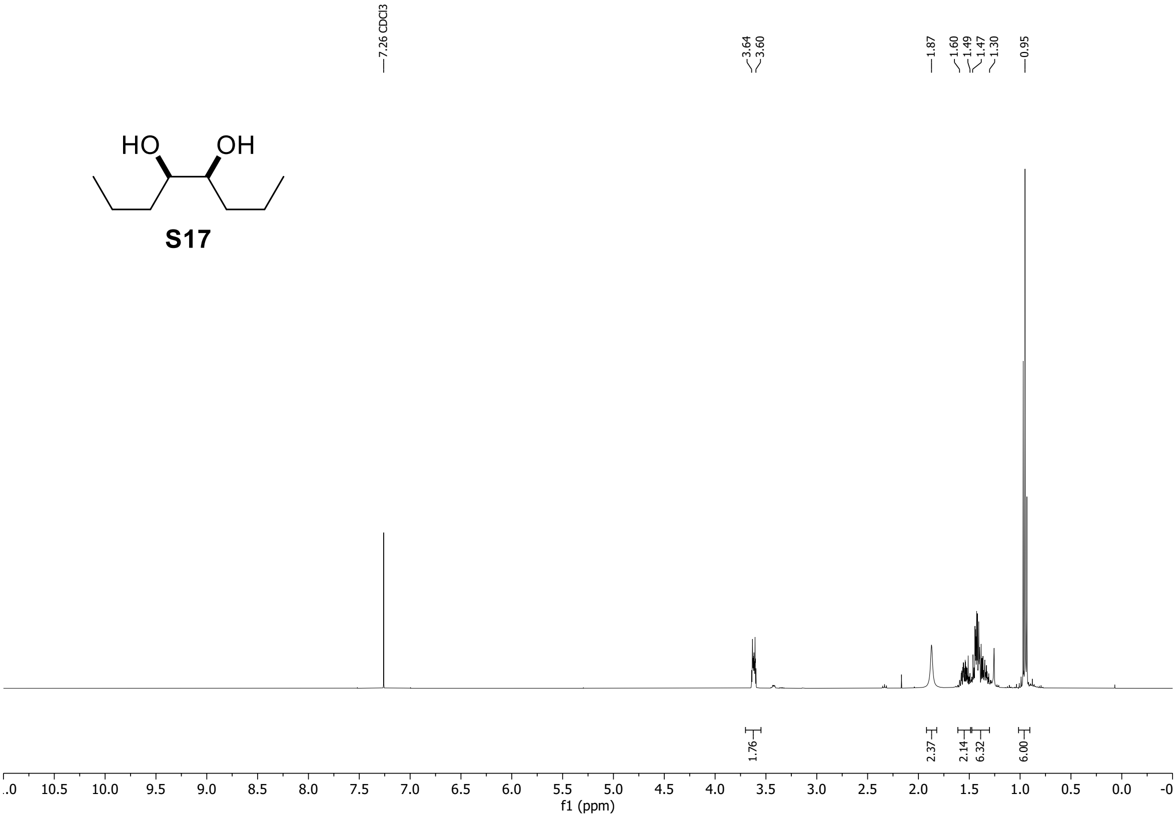

^{1}H NMR (400 MHz, $CDCl_3$) spectrum of compound **S17**.

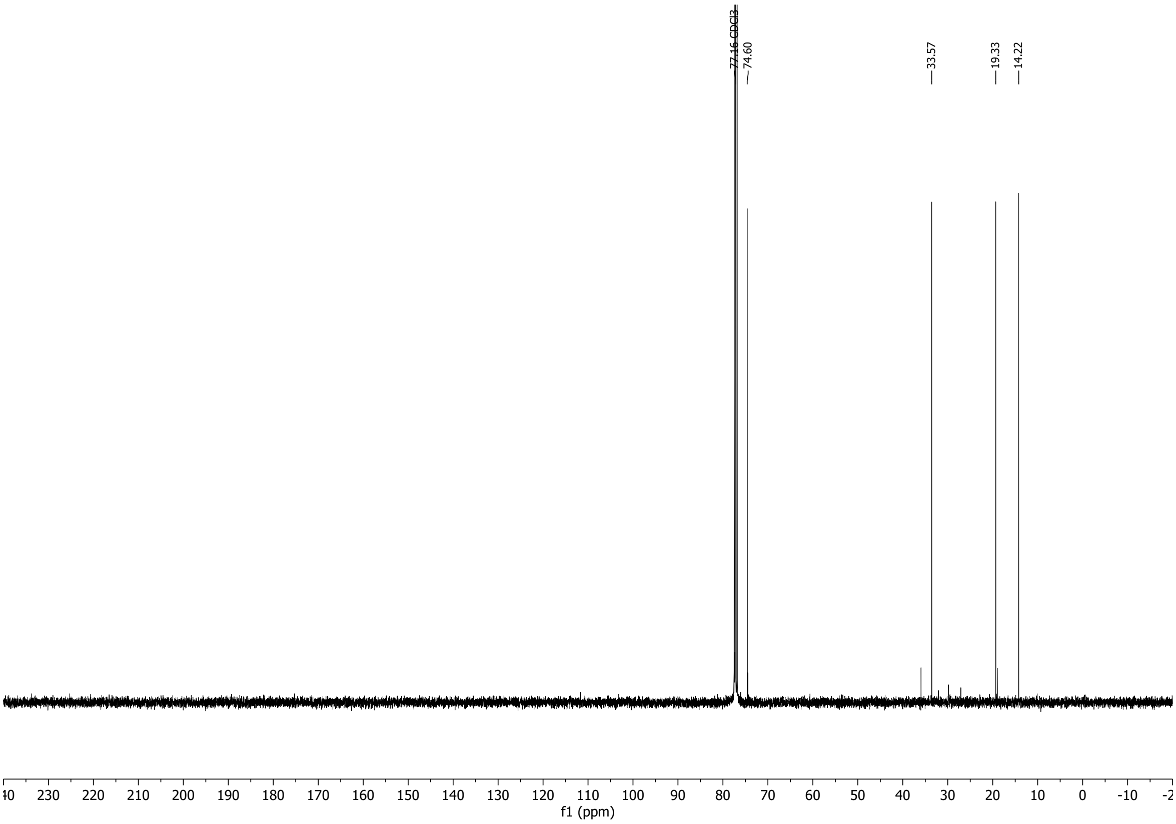

^{13}C NMR (100 MHz, $CDCl_3$) spectrum of compound **S17**.

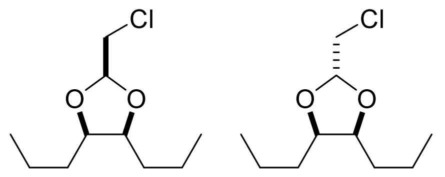

S18

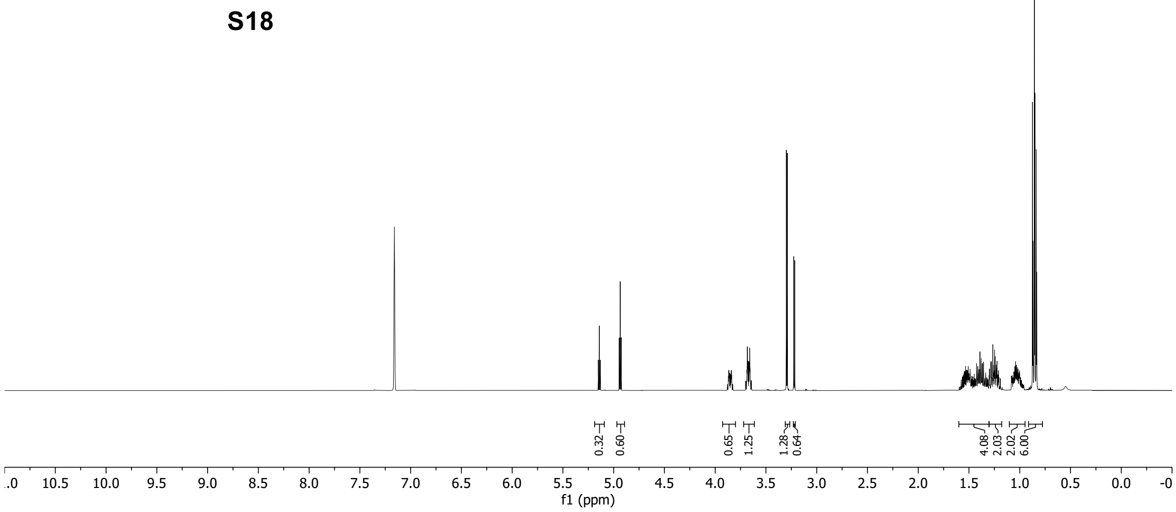

^{1}H NMR (400 MHz, C_6D_6) spectrum of compound **S18**.

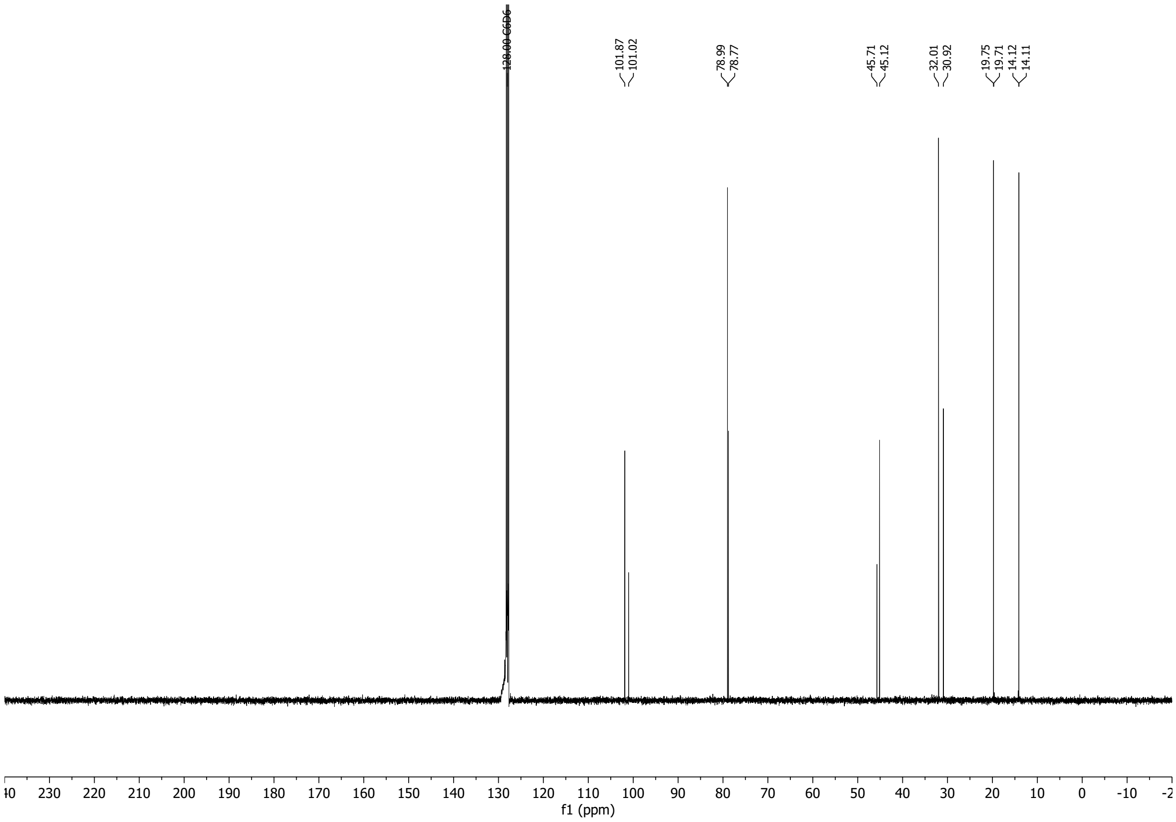

^{13}C NMR (100 MHz, C_6D_6) spectrum of compound **S18**.

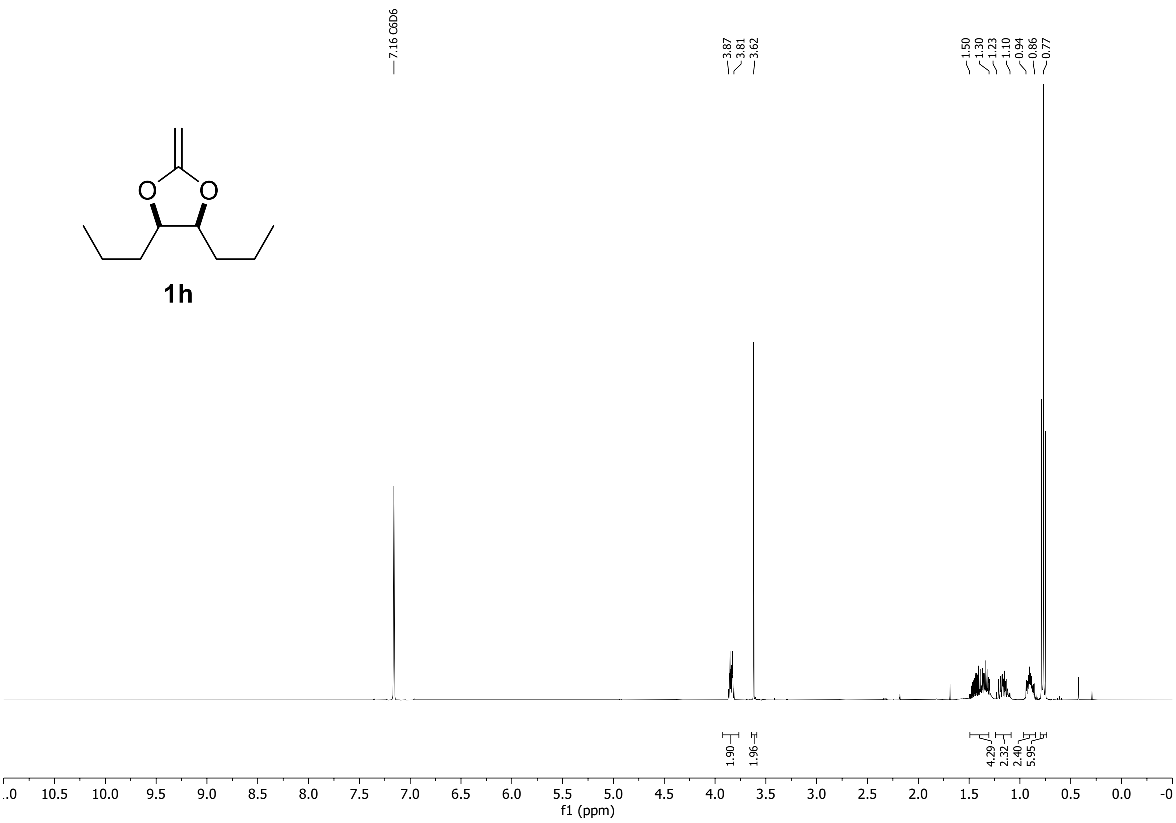

^{1}H NMR (400 MHz, C_6D_6) spectrum of compound **1h**.

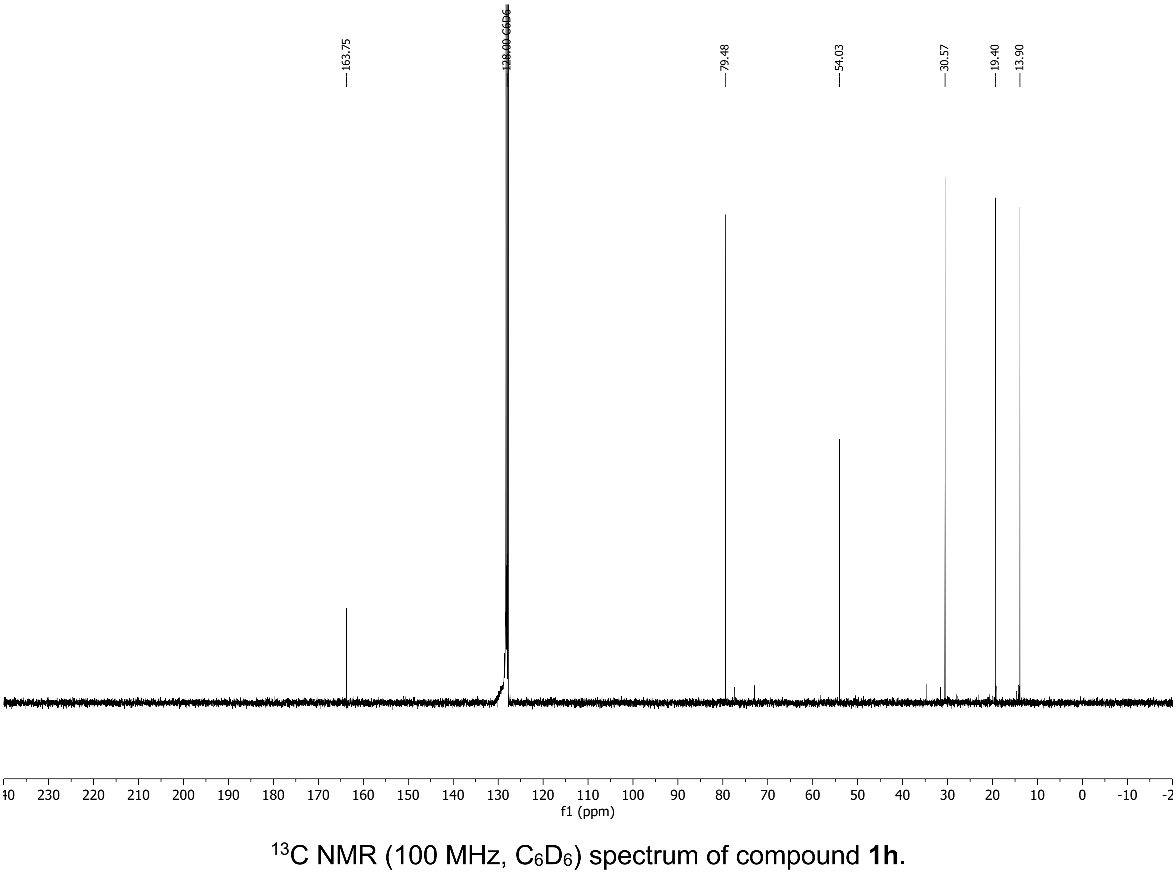

^{13}C NMR (100 MHz, C_6D_6) spectrum of compound **1h**.

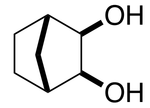

S19

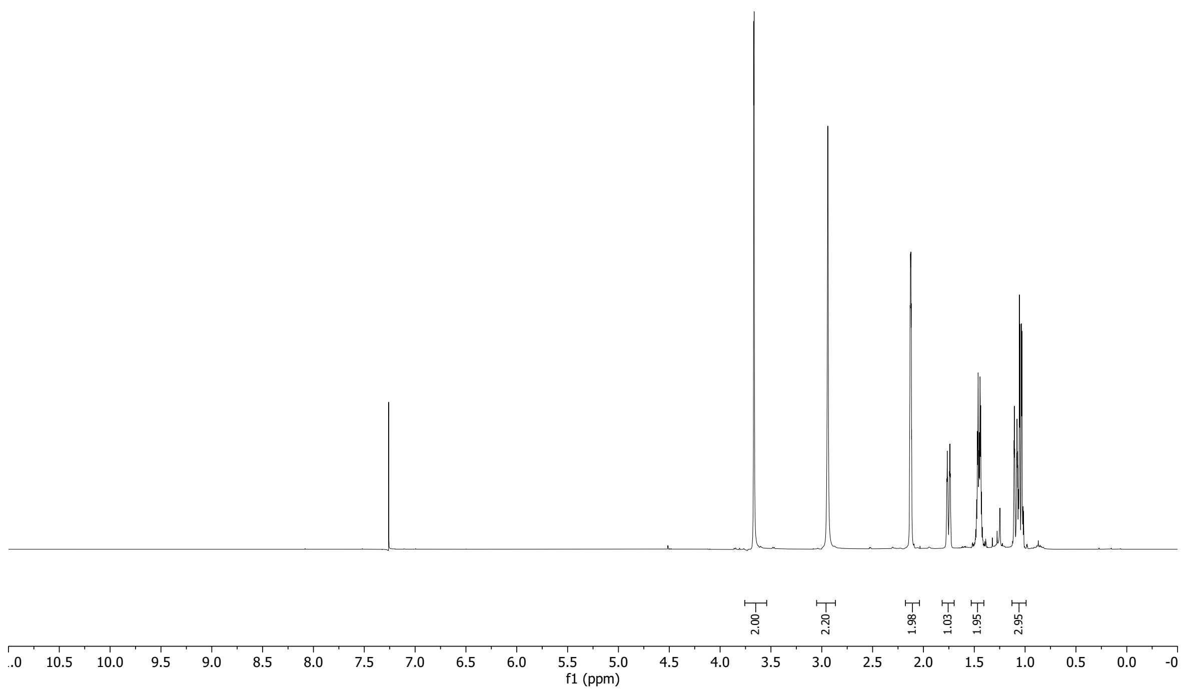

^{1}H NMR (400 MHz, $CDCl_3$) spectrum of compound **S19**.

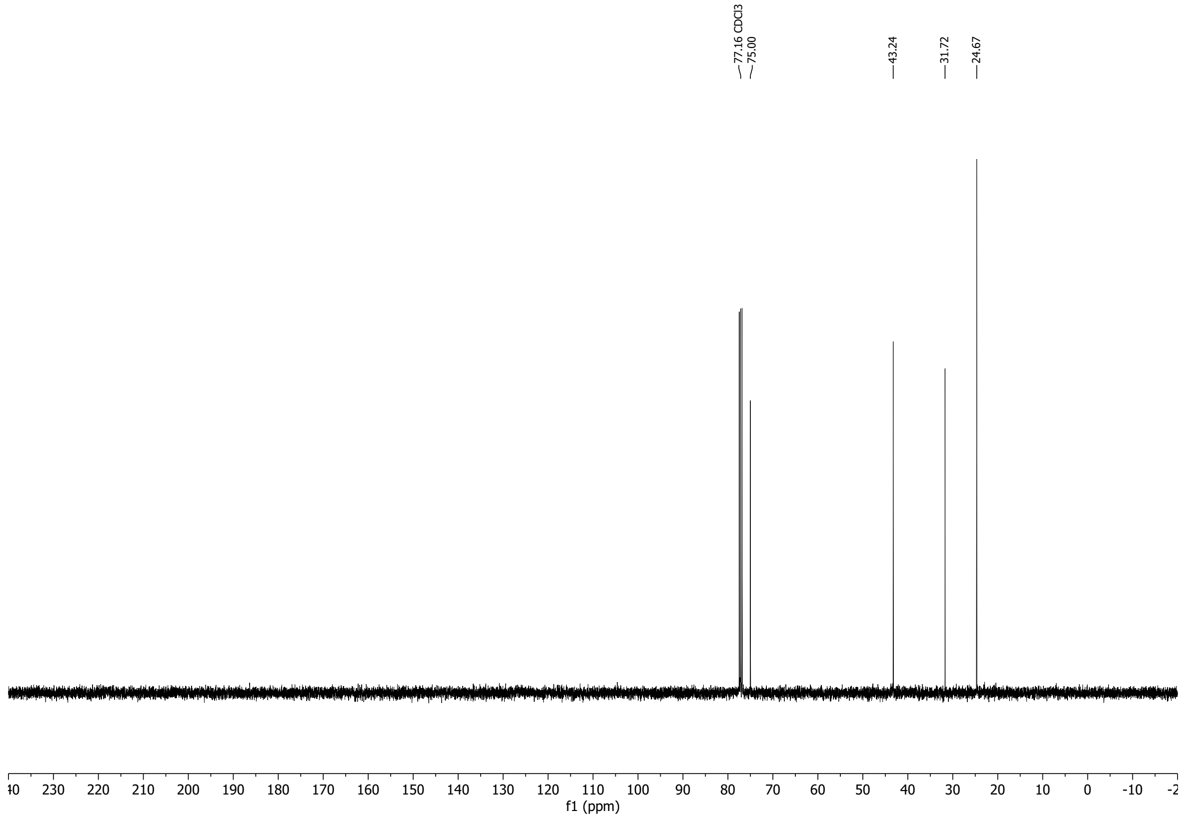

^{13}C NMR (100 MHz, $CDCl_3$) spectrum of compound **S19**.

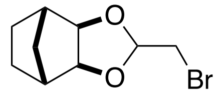

S20

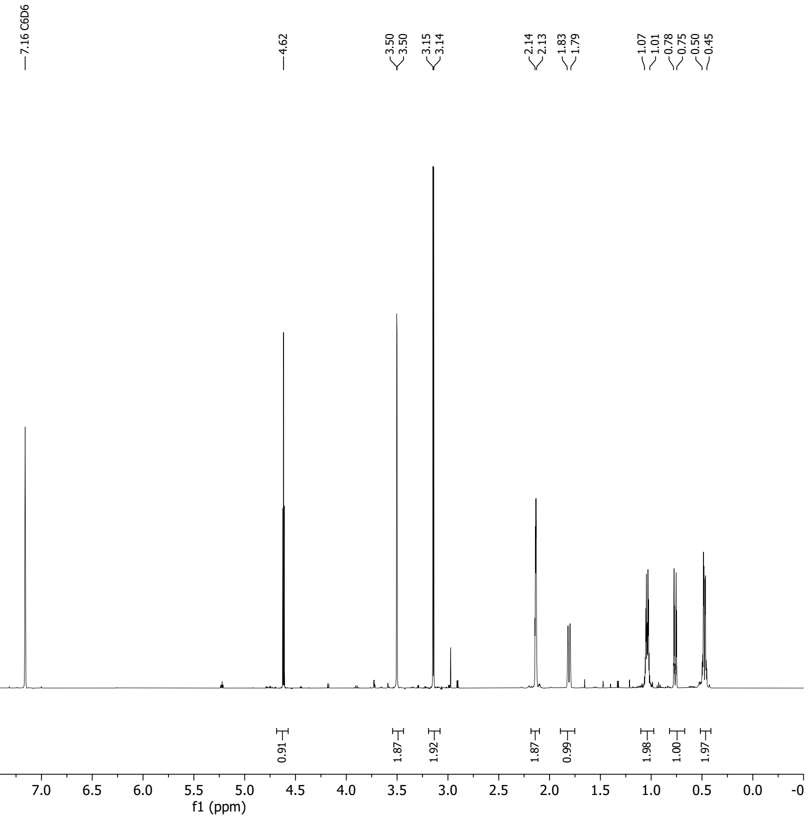

^{1}H NMR (500 MHz, C_6D_6) spectrum of compound **S20**.

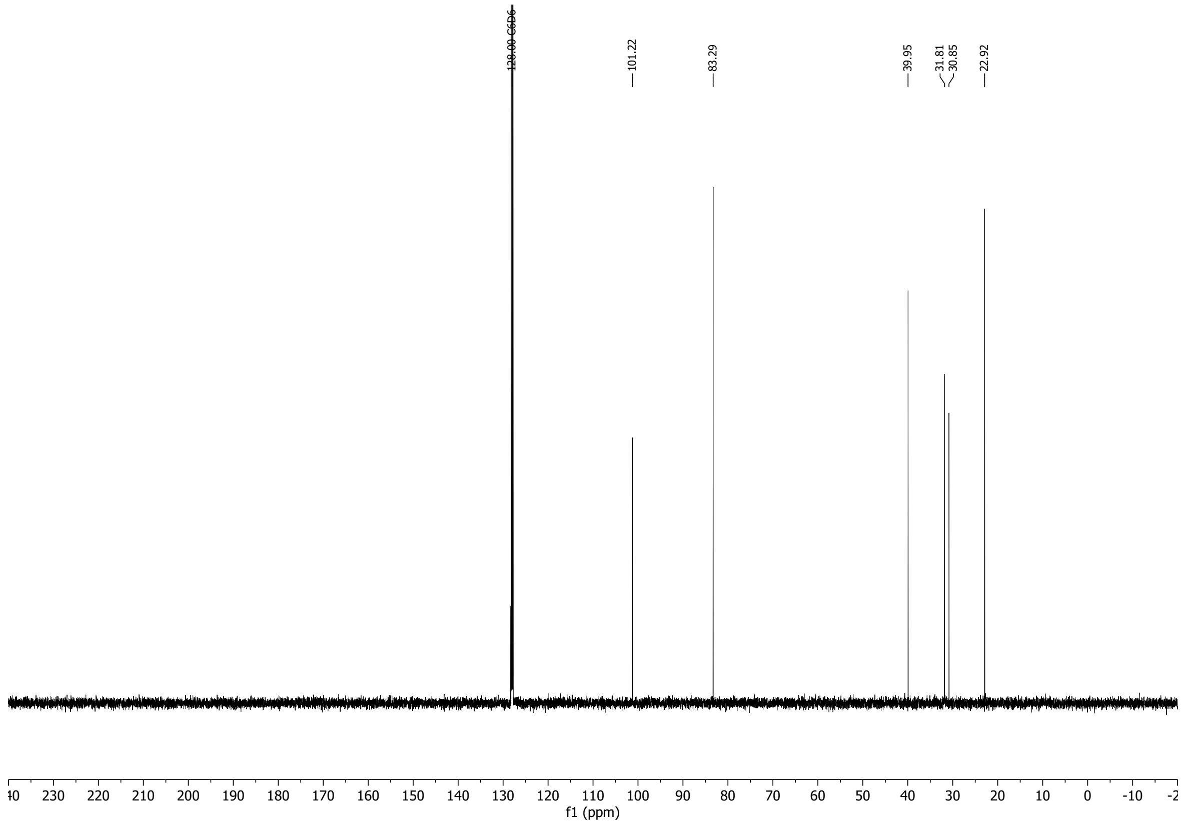

^{13}C NMR (125 MHz, C_6D_6) spectrum of compound **S20**.

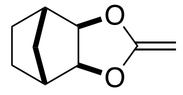

1i

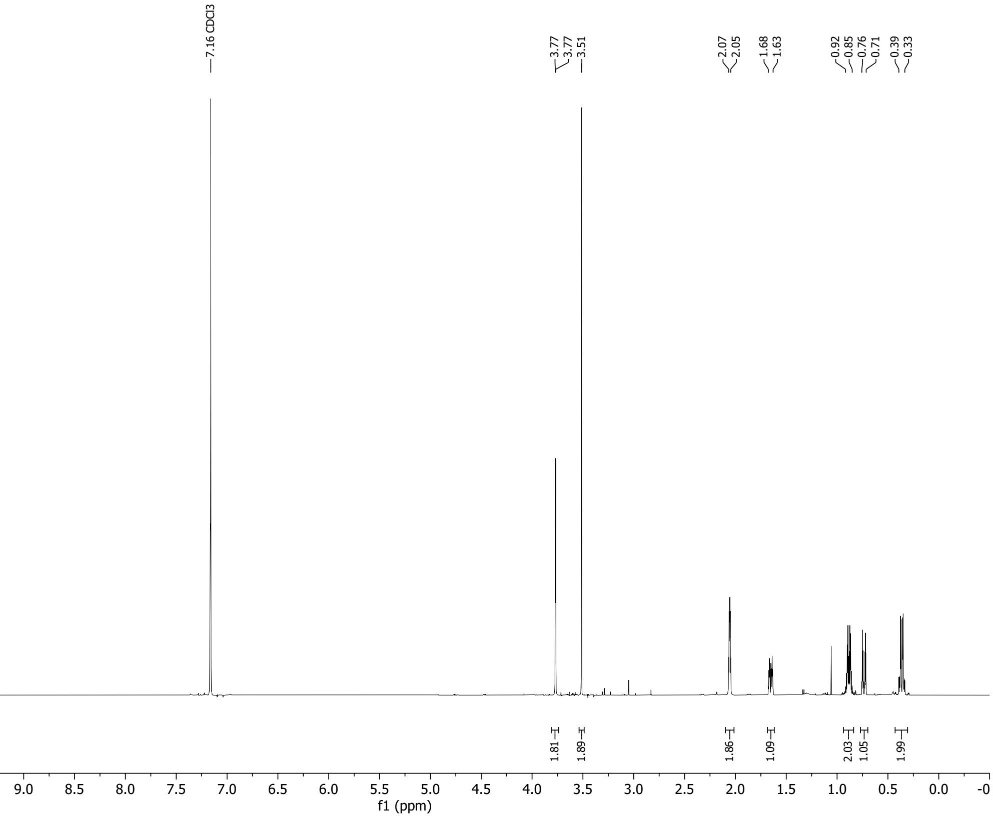

^{1}H NMR (400 MHz, C_6D_6) spectrum of compound **1i**.

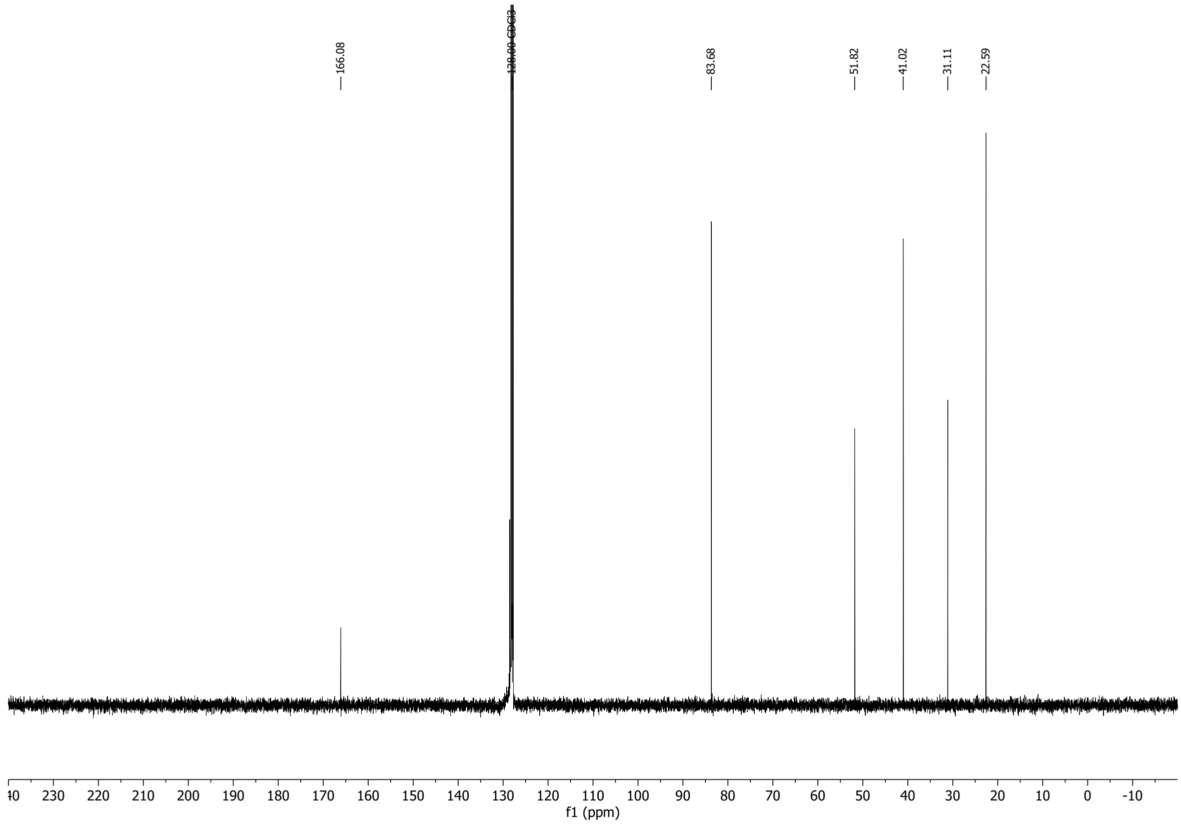

^{13}C NMR (100 MHz, C_6D_6) spectrum of compound **1i**.

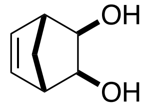

S21

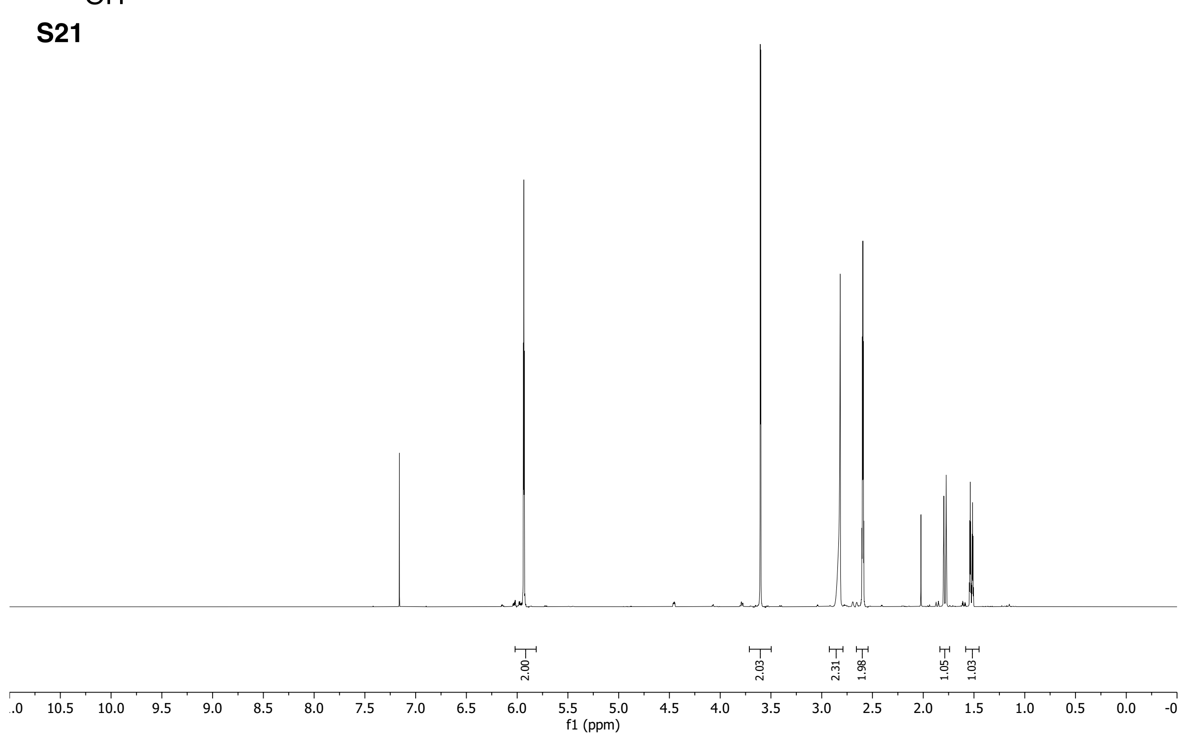

^{1}H NMR (400 MHz, $CDCl_3$) spectrum of compound **S21**.

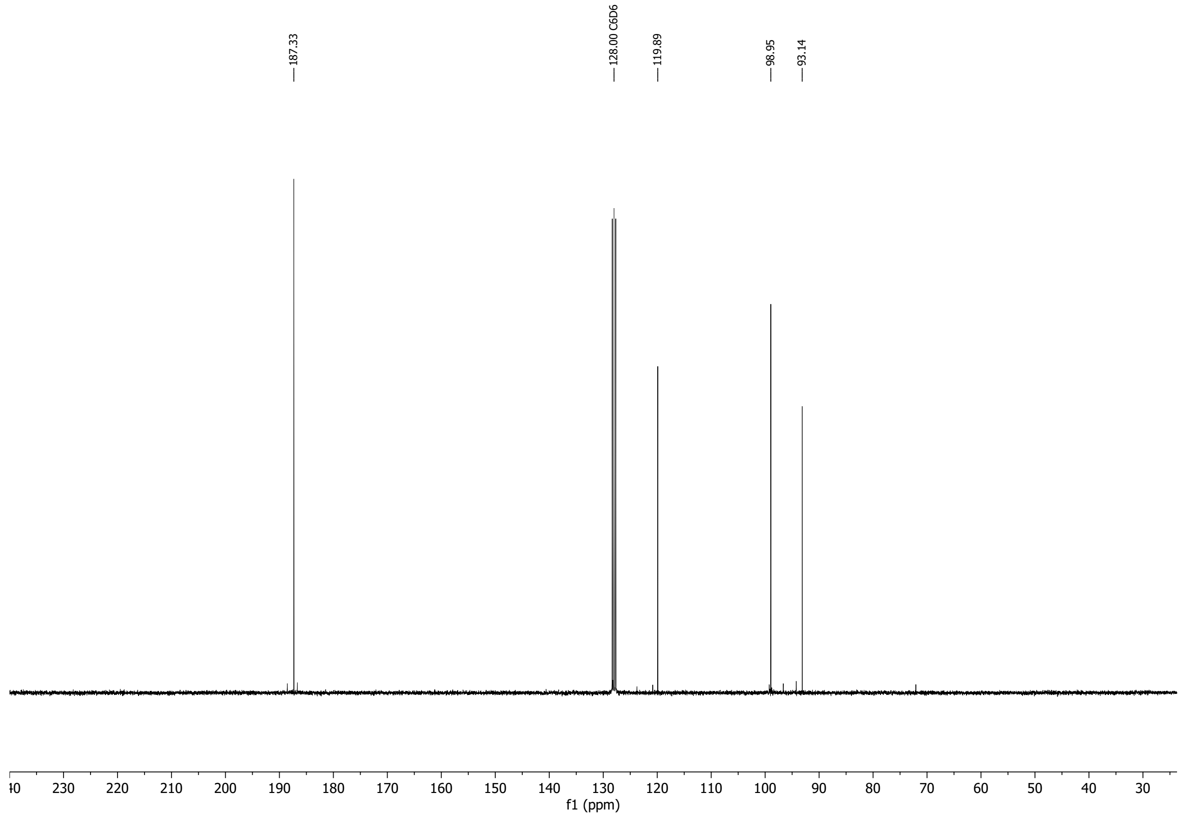

^{13}C NMR (100 MHz, $CDCl_3$) spectrum of compound **S21**.

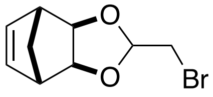

S22

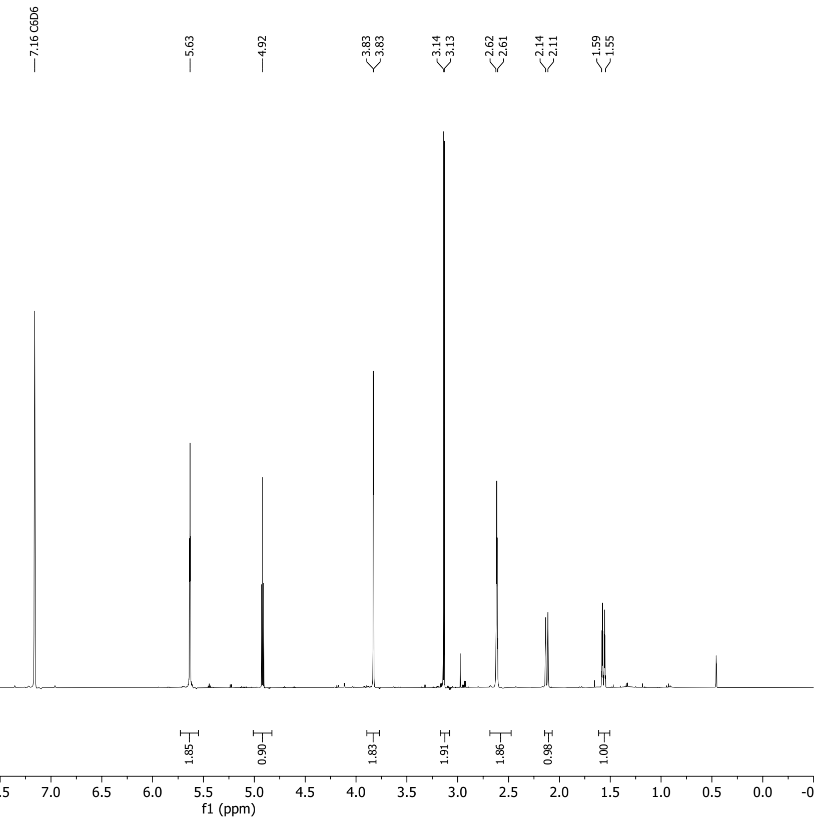

^{1}H NMR (500 MHz, C_6D_6) spectrum of compound **S22**.

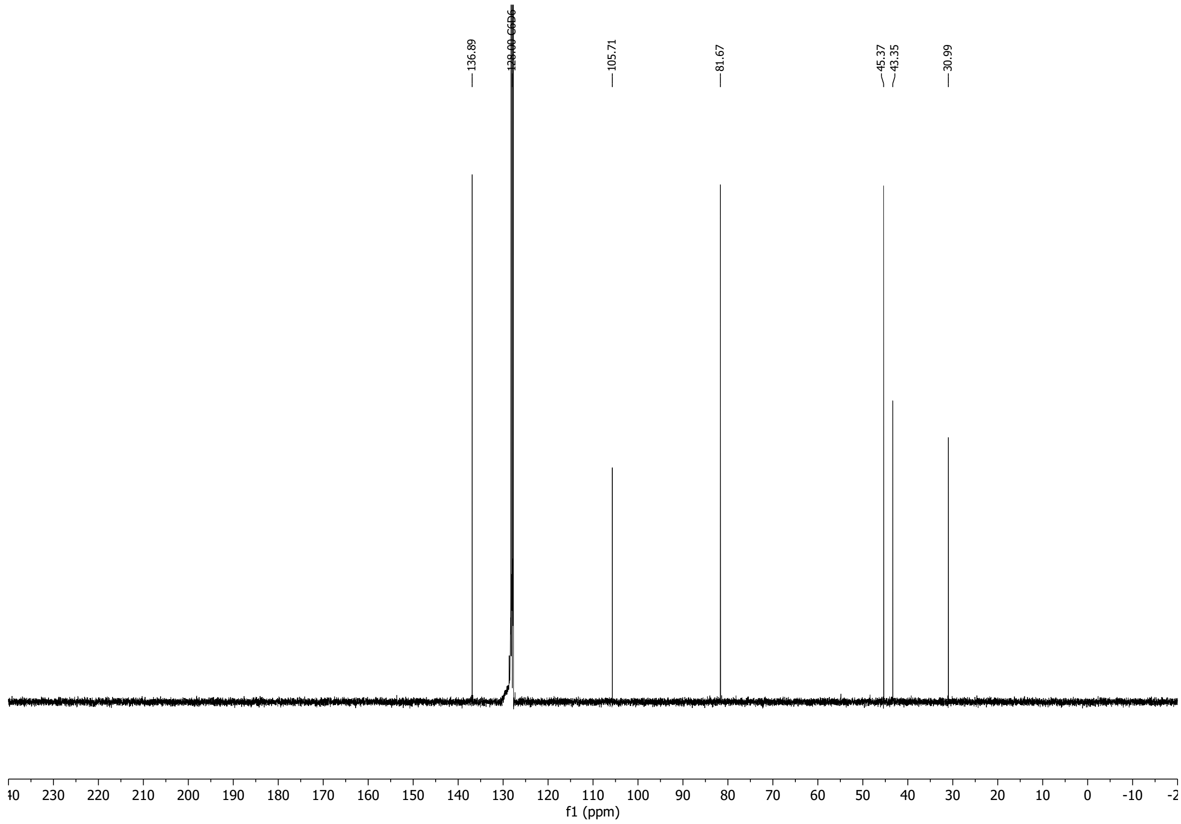

^{13}C NMR (125 MHz, C_6D_6) spectrum of compound **S22**.

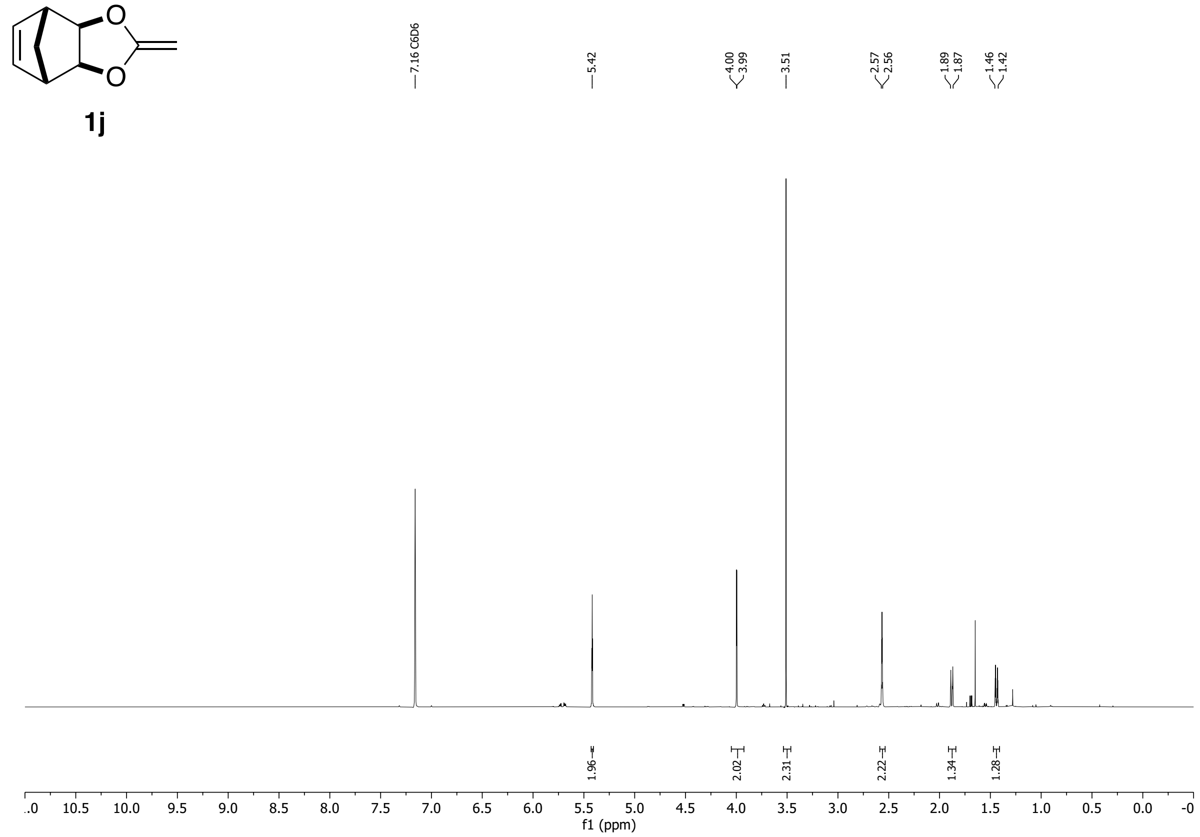

^{1}H NMR (400 MHz, C_6D_6) spectrum of compound **1j**.

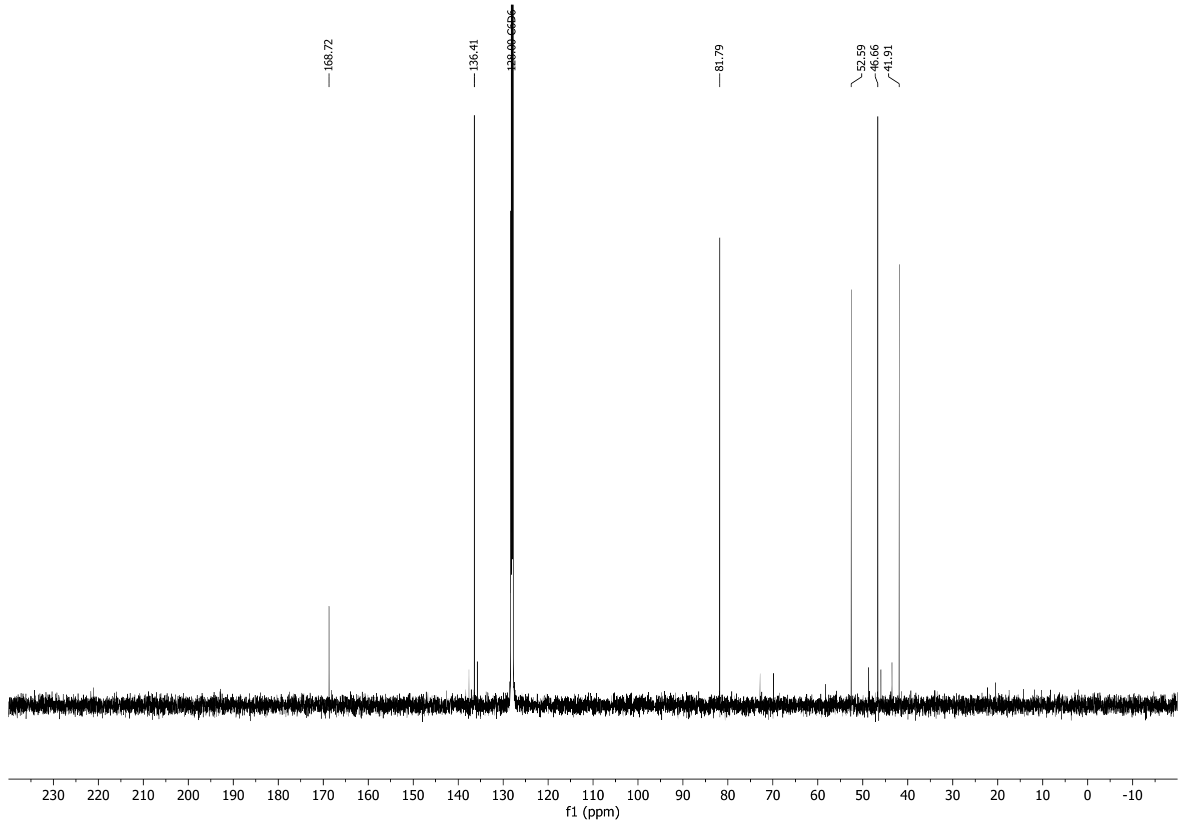

^{13}C NMR (100 MHz, C_6D_6) spectrum of compound **1j**.

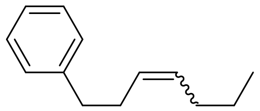

S23

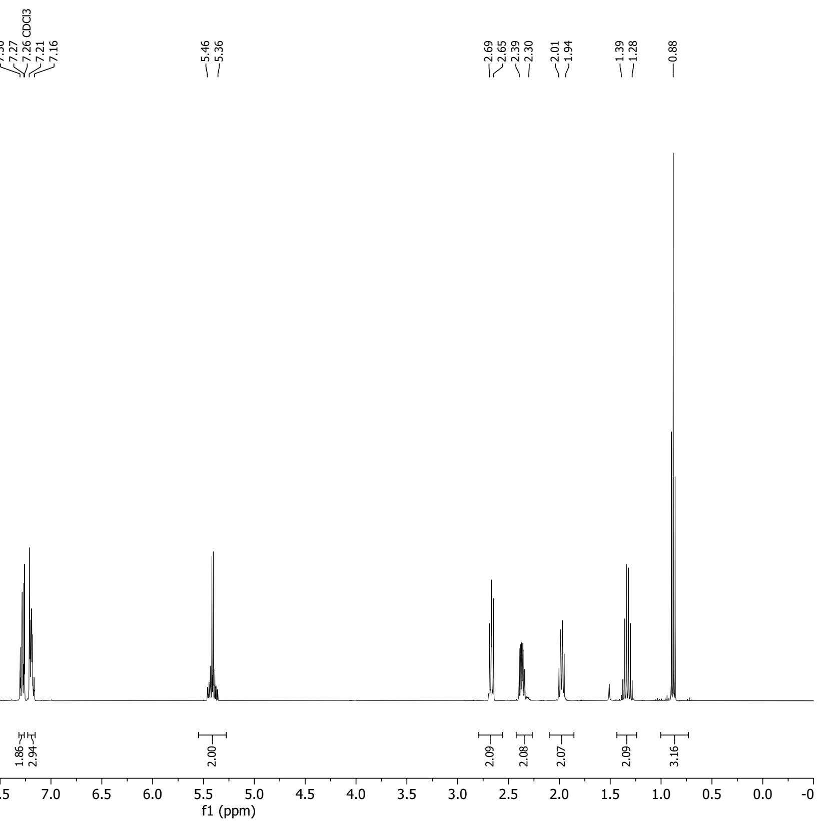

^{1}H NMR (400 MHz, $CDCl_3$) spectrum of compound **S23**.

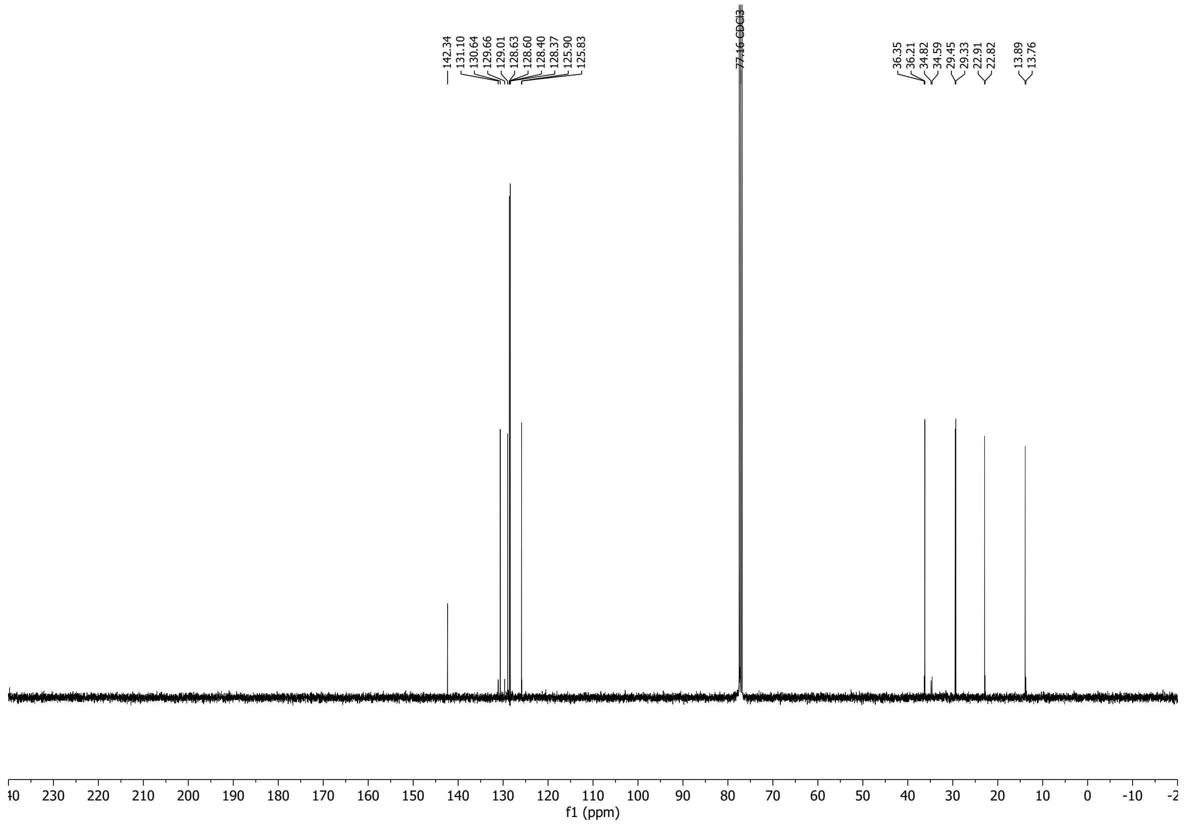

^{13}C NMR (100 MHz, $CDCl_3$) spectrum of compound **S23**.

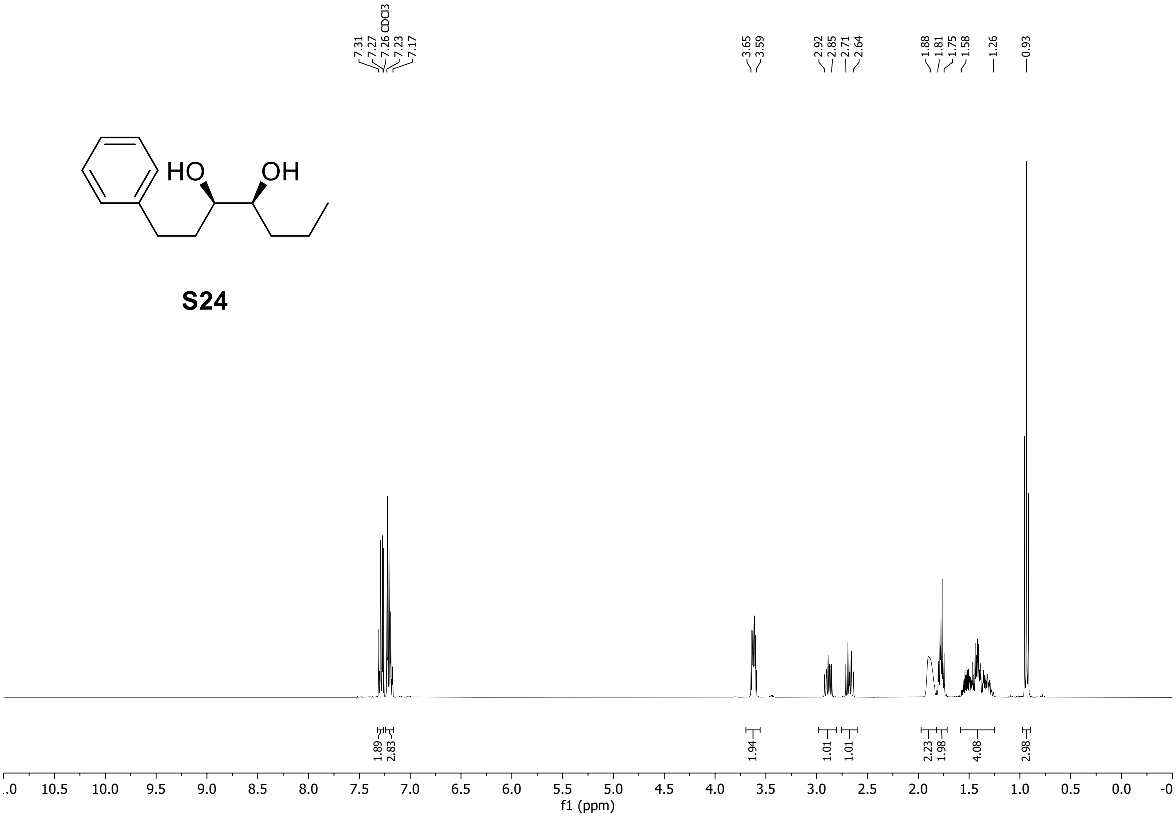

^{1}H NMR (400 MHz, $CDCl_3$) spectrum of compound **S24**.

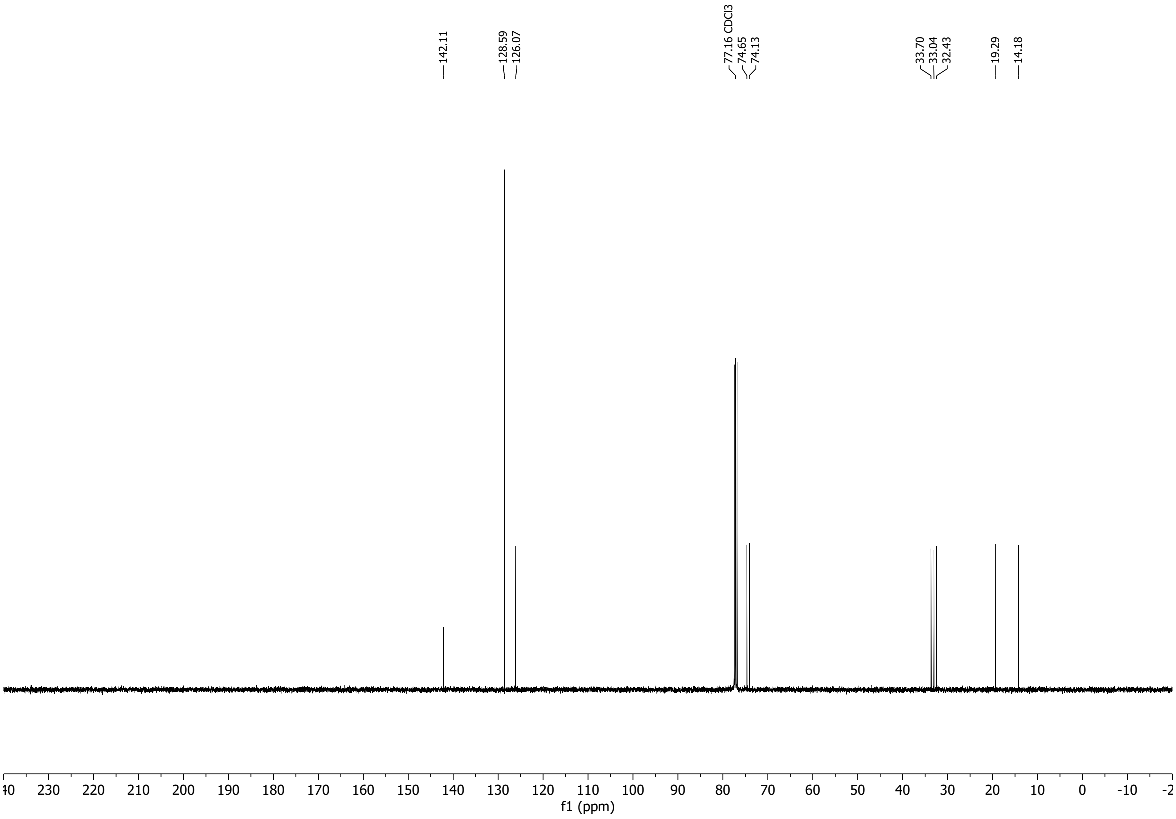

^{13}C NMR (100 MHz, $CDCl_3$) spectrum of compound **S24**.

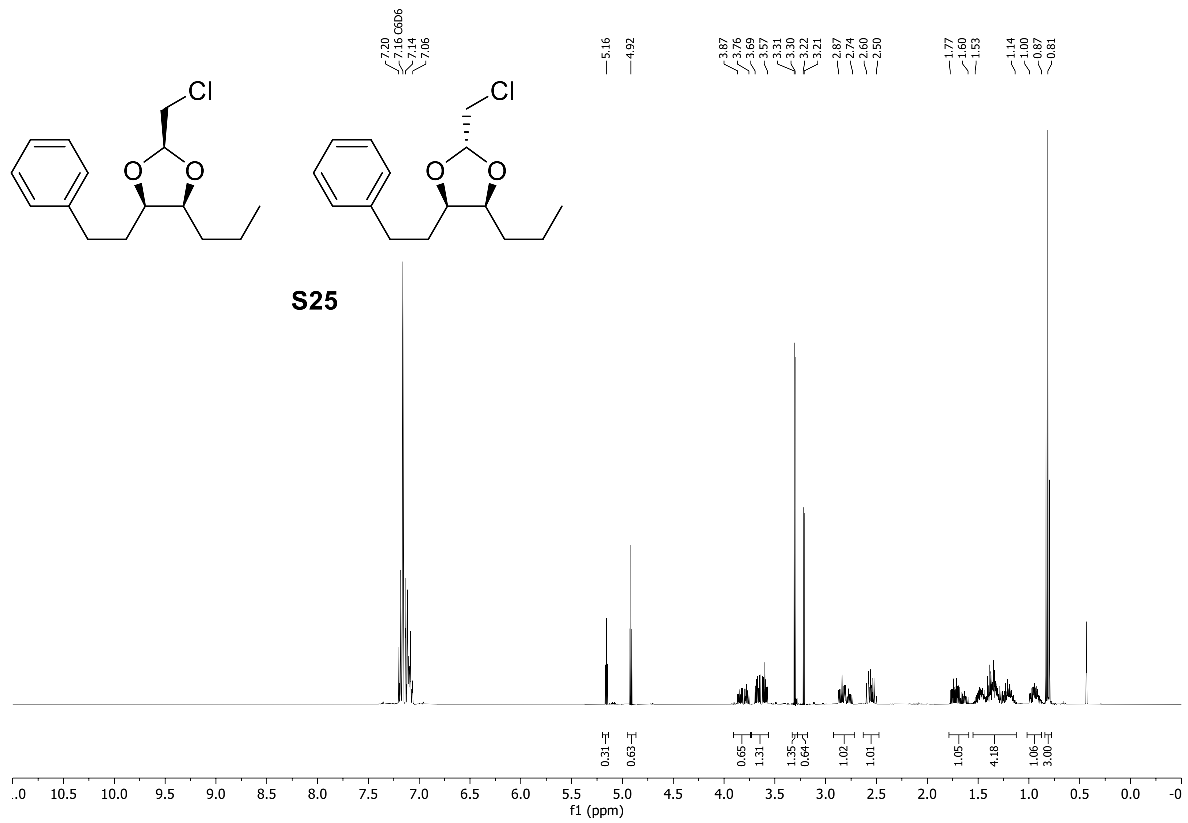

^{1}H NMR (400 MHz, C_6D_6) spectrum of compound **S25**.

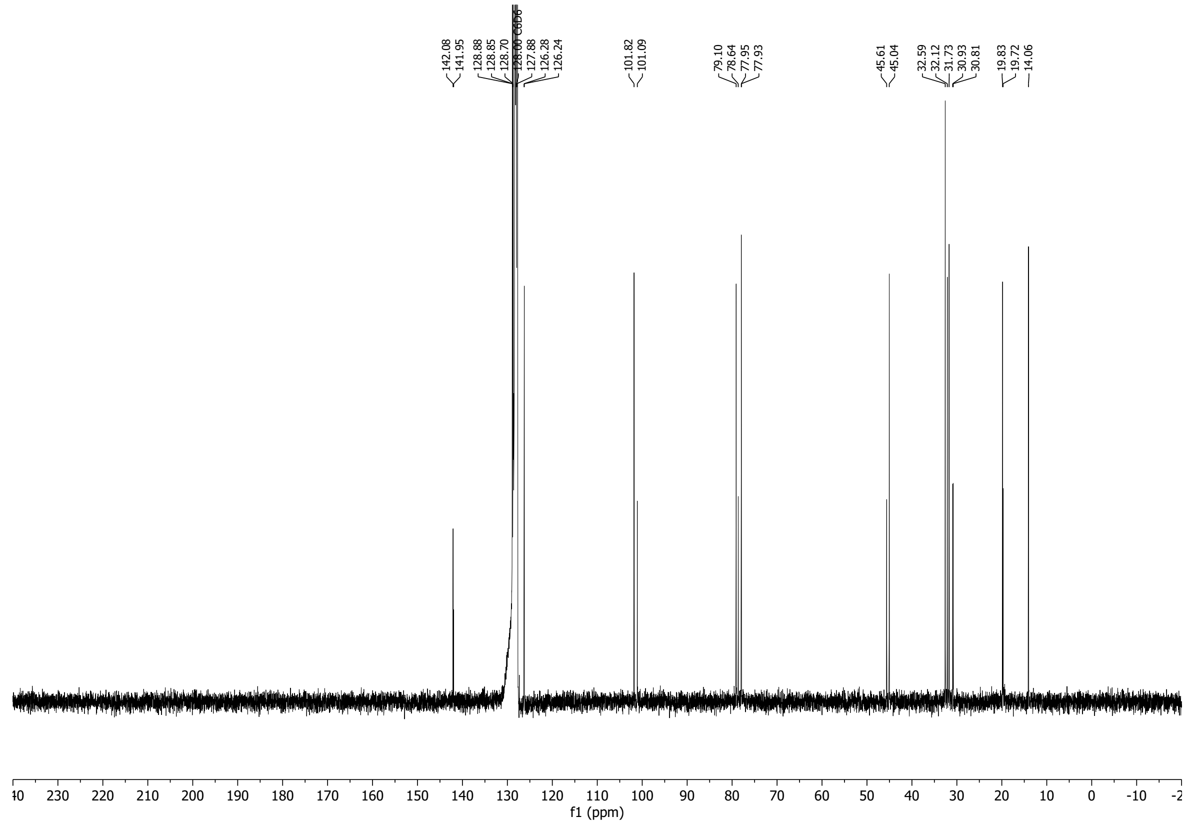

^{13}C NMR (100 MHz, C_6D_6) spectrum of compound **S25**.

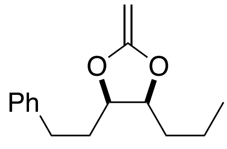

rac-**3**

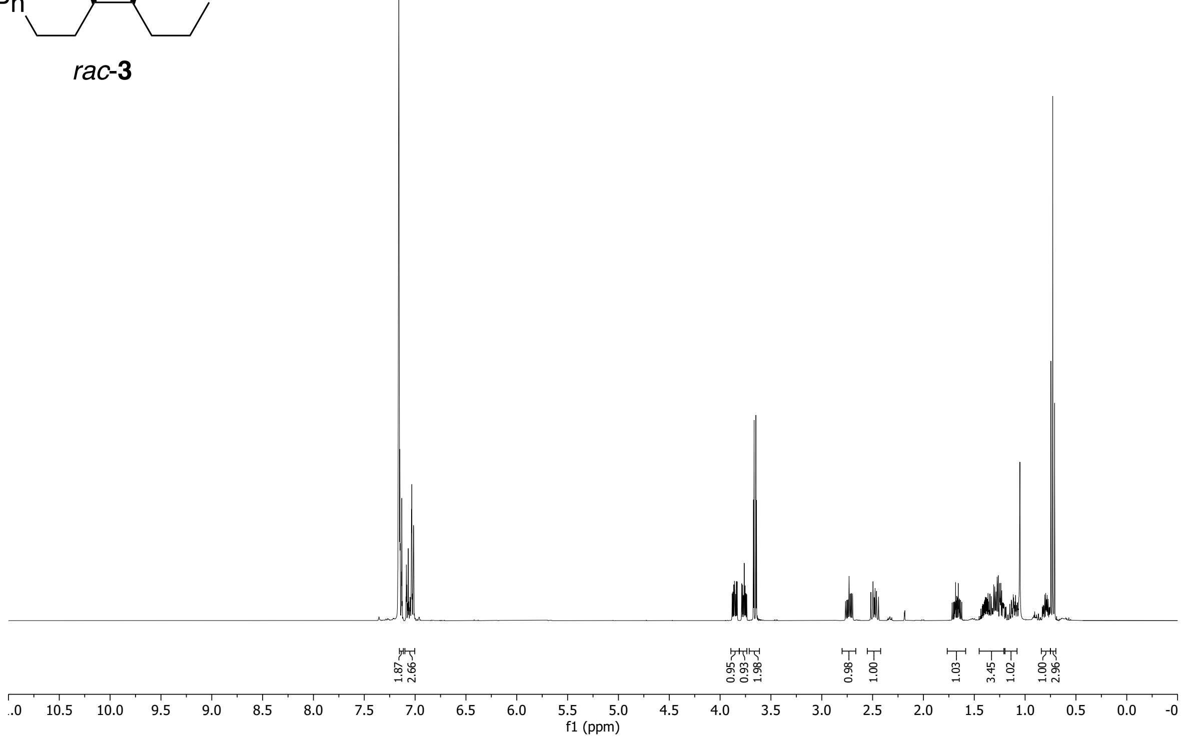

^{1}H NMR (400 MHz, C_6D_6) spectrum of compound *rac*-**3**.

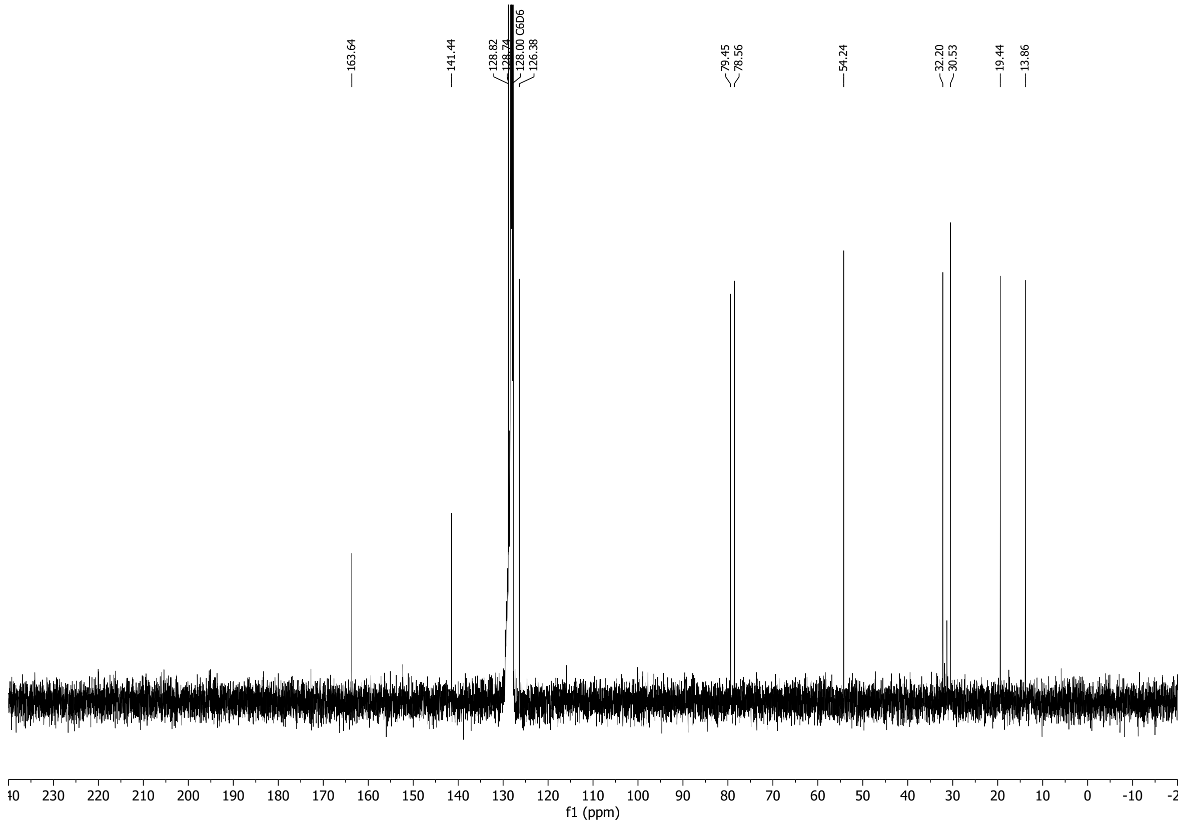

^{13}C NMR (100 MHz, C_6D_6) spectrum of compound *rac*-**3**.

2a

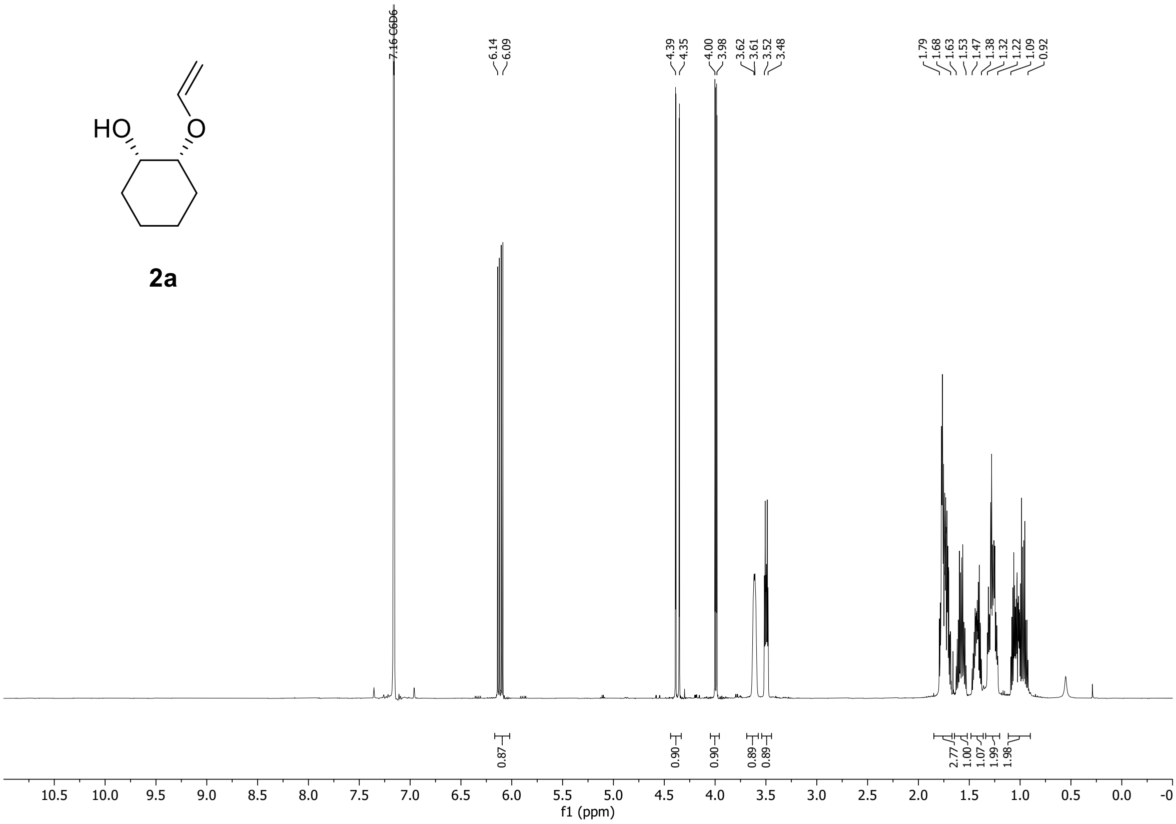

^{1}H NMR (400 MHz, C_6D_6) spectrum of compound **2a**.

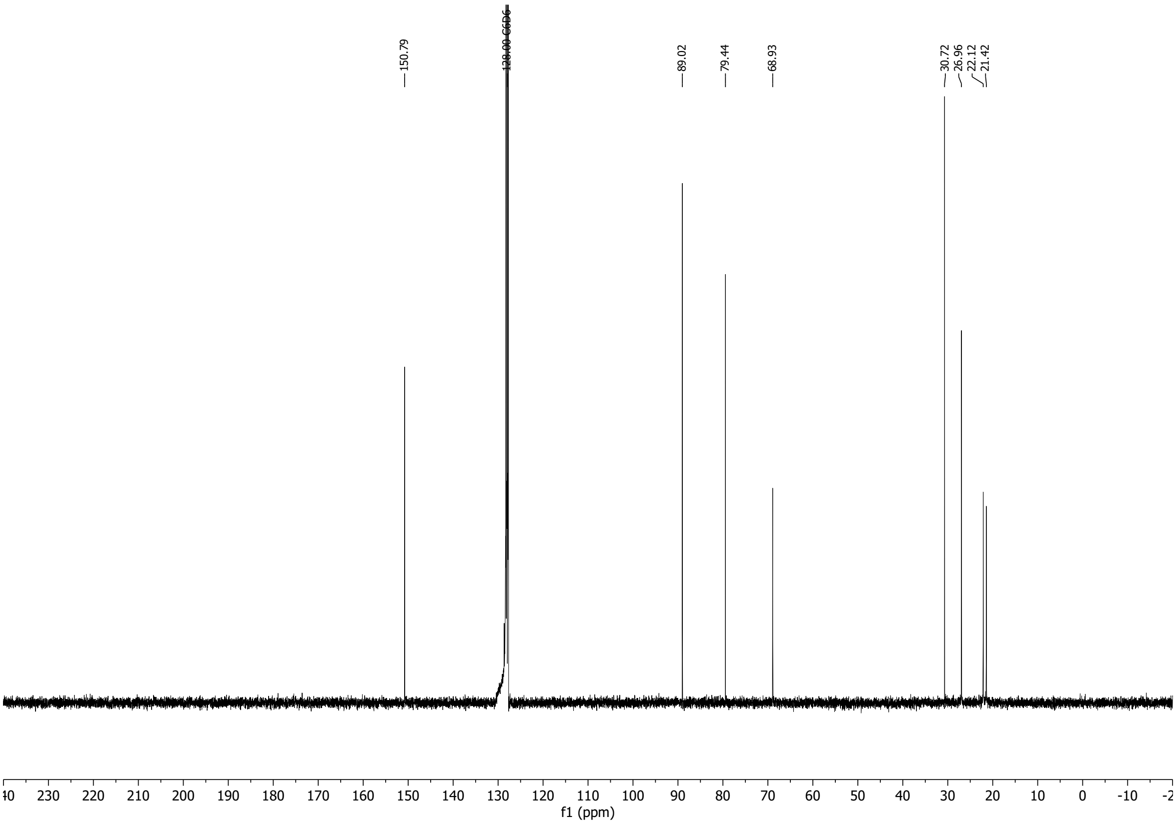

^{13}C NMR (100 MHz, C_6D_6) spectrum of compound **2a**.

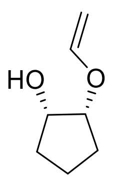

2b

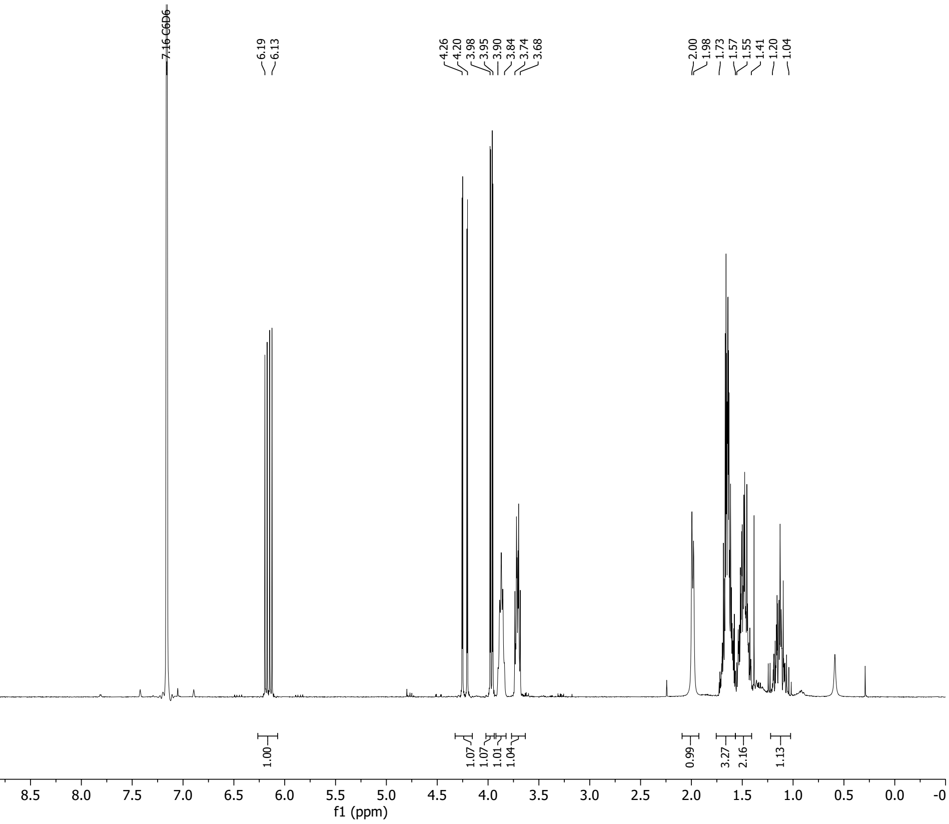

^{1}H NMR (400 MHz, C_6D_6) spectrum of compound **2b**.

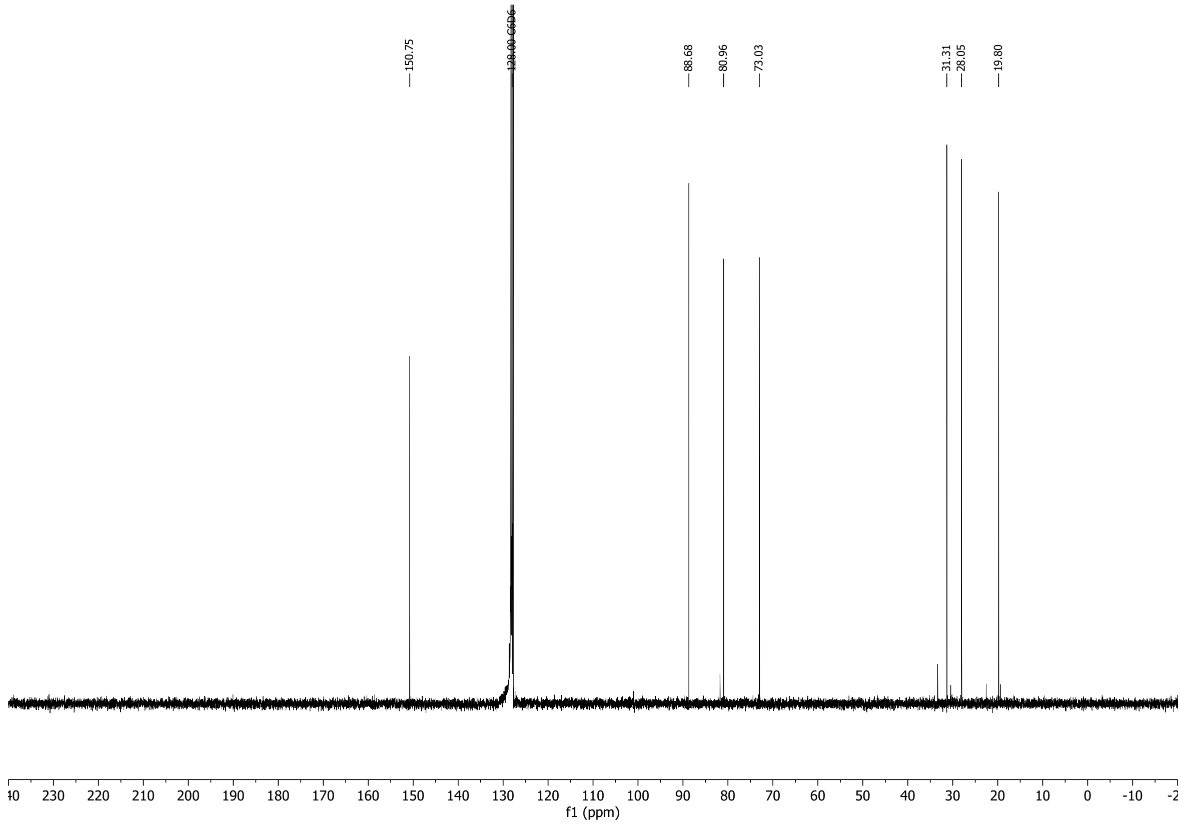

^{13}C NMR (100 MHz, C_6D_6) spectrum of compound **2b**.

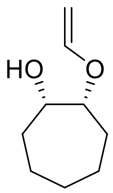

2c

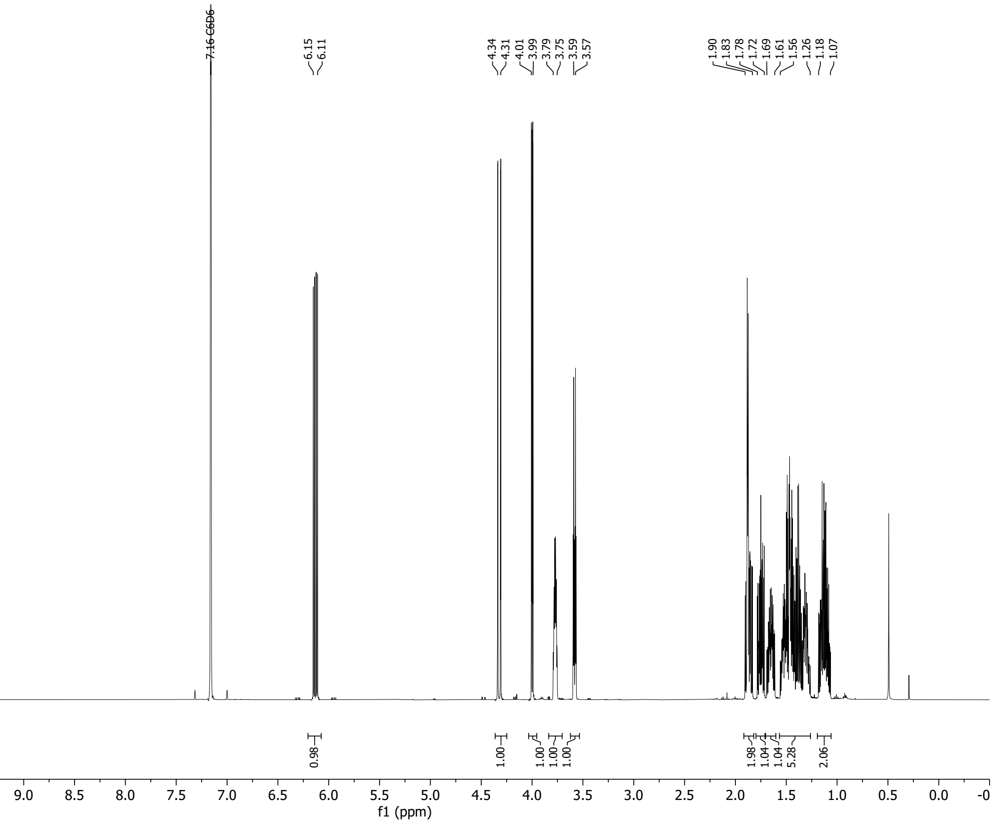

^{1}H NMR (500 MHz, C_6D_6) spectrum of compound **2c**.

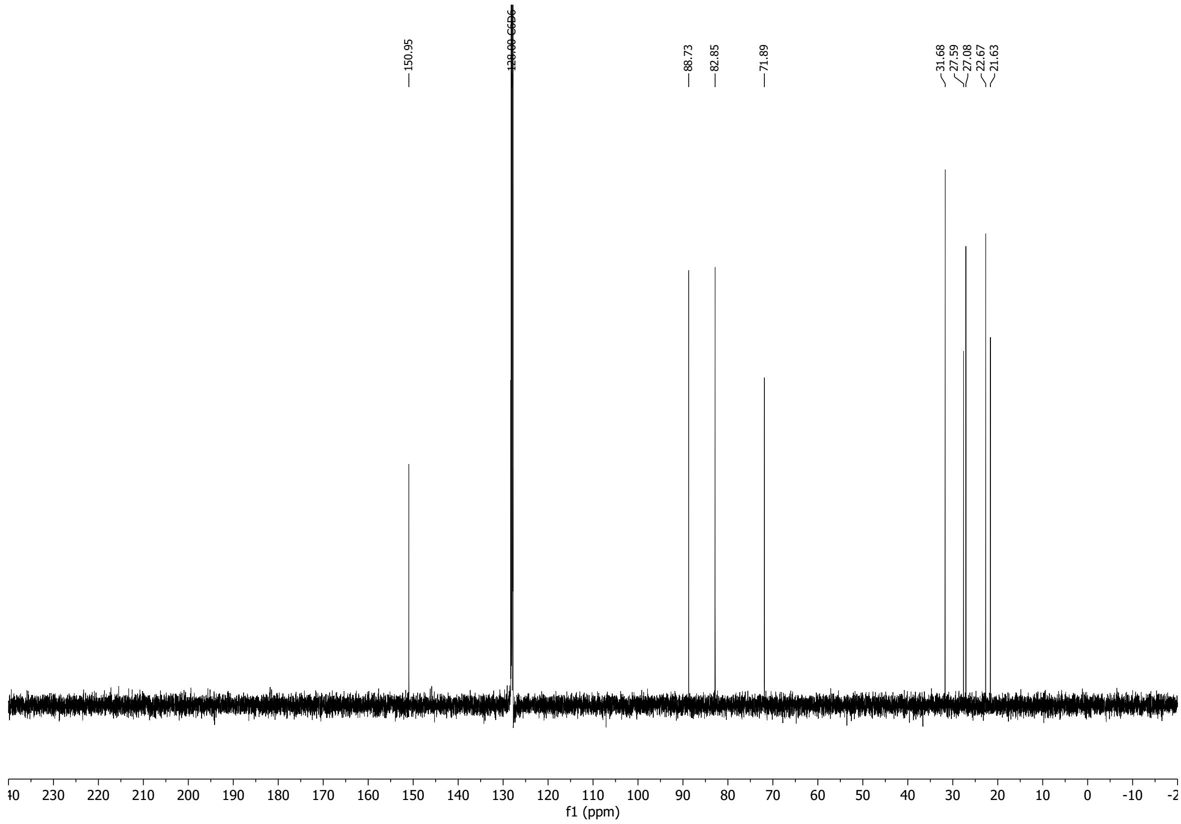

^{13}C NMR (125 MHz, C_6D_6) spectrum of compound **2c**.

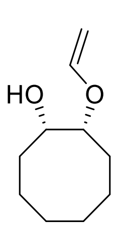

2d

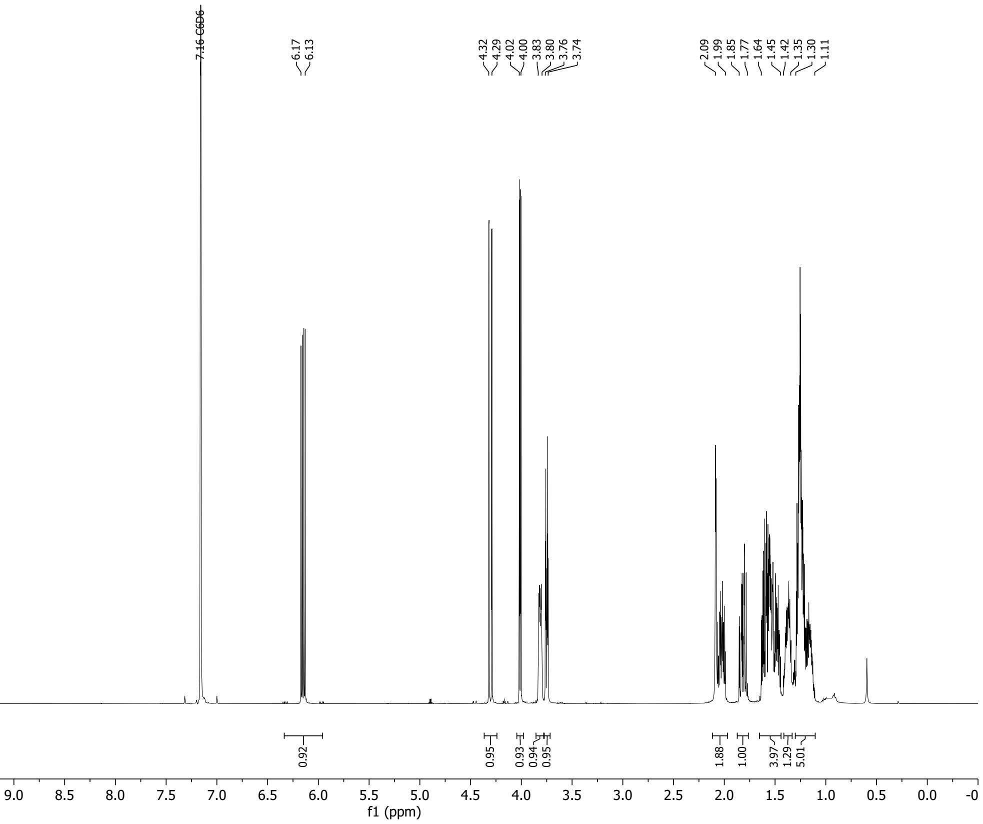

^{1}H NMR (500 MHz, C_6D_6) spectrum of compound **2d**.

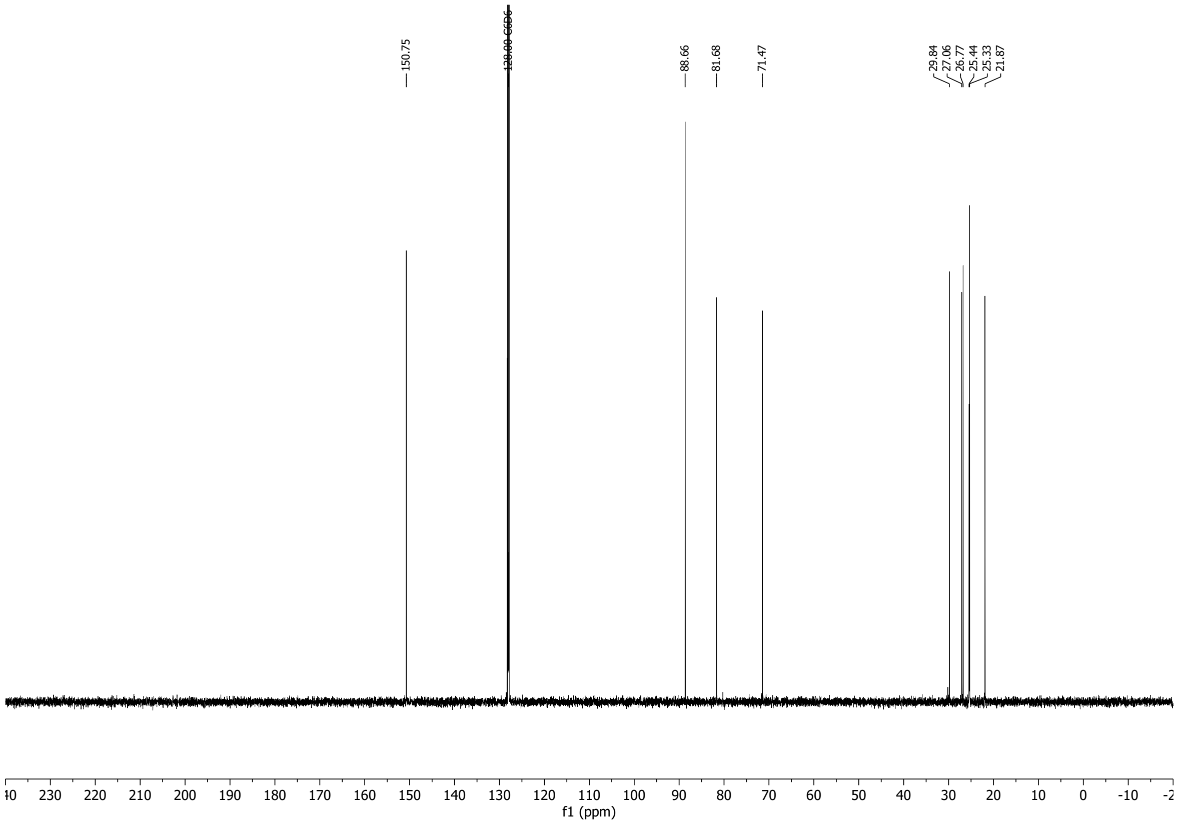

^{13}C NMR (125 MHz, C_6D_6) spectrum of compound **2d**.

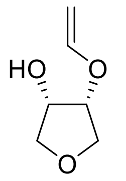

2e

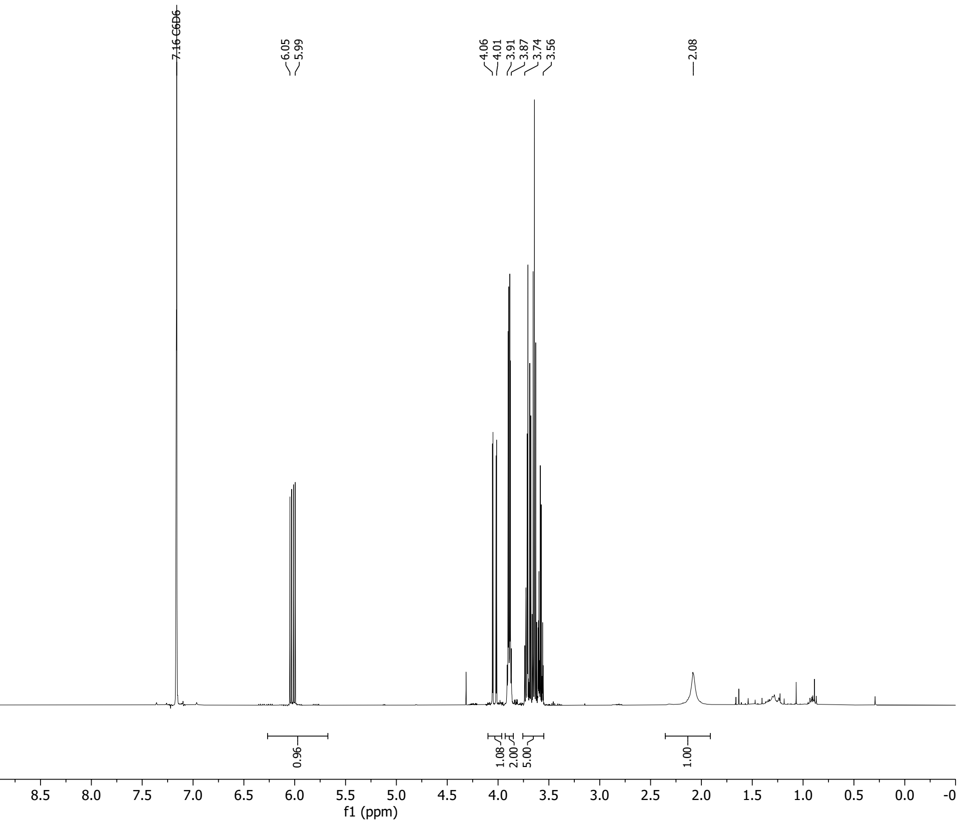

^{1}H NMR (400 MHz, C_6D_6) spectrum of compound **2e**.

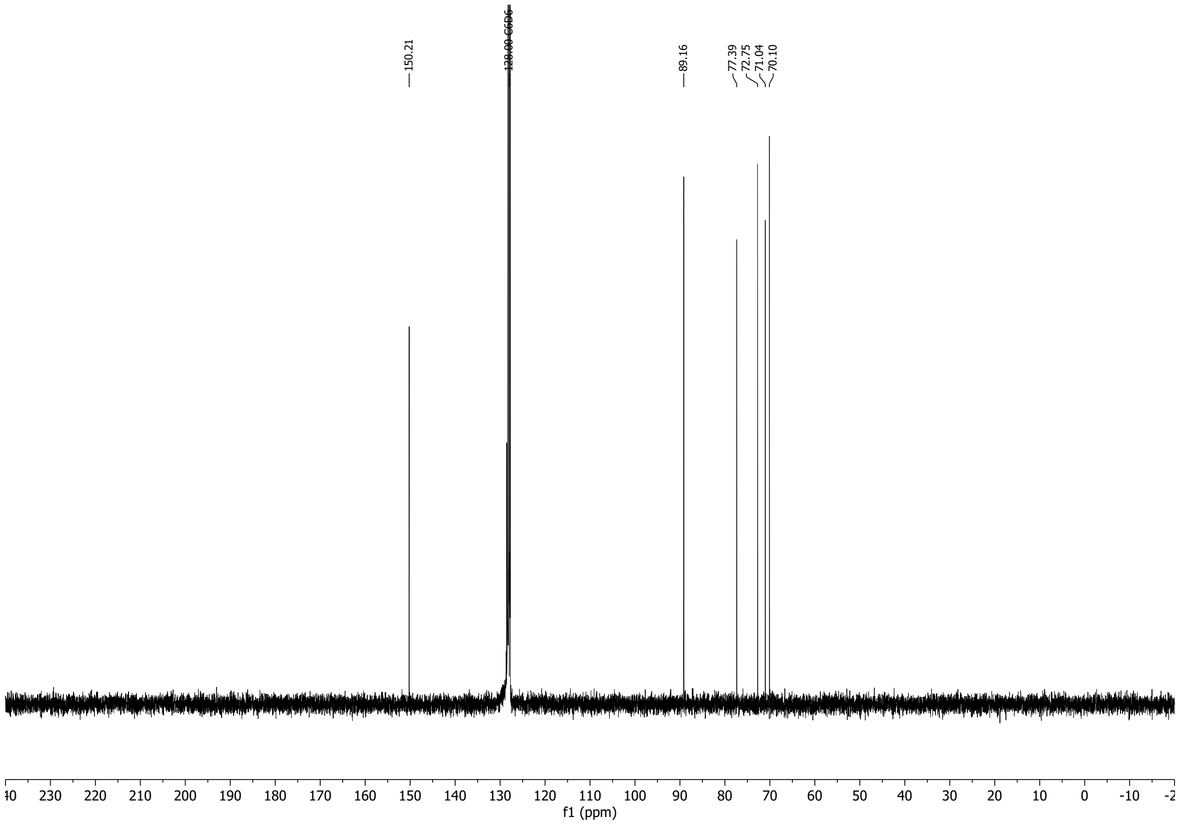

^{13}C NMR (100 MHz, C_6D_6) spectrum of compound **2e**.

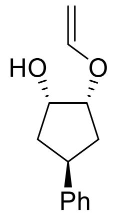

2f

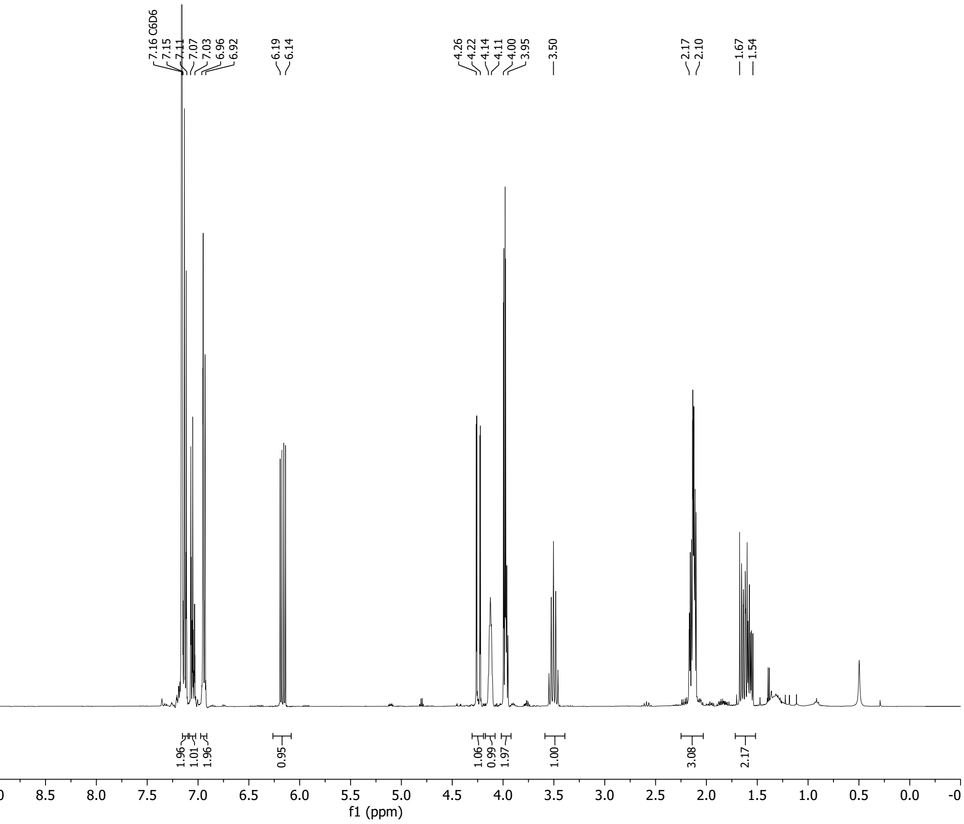

^{1}H NMR (400 MHz, C_6D_6) spectrum of compound **2f**.

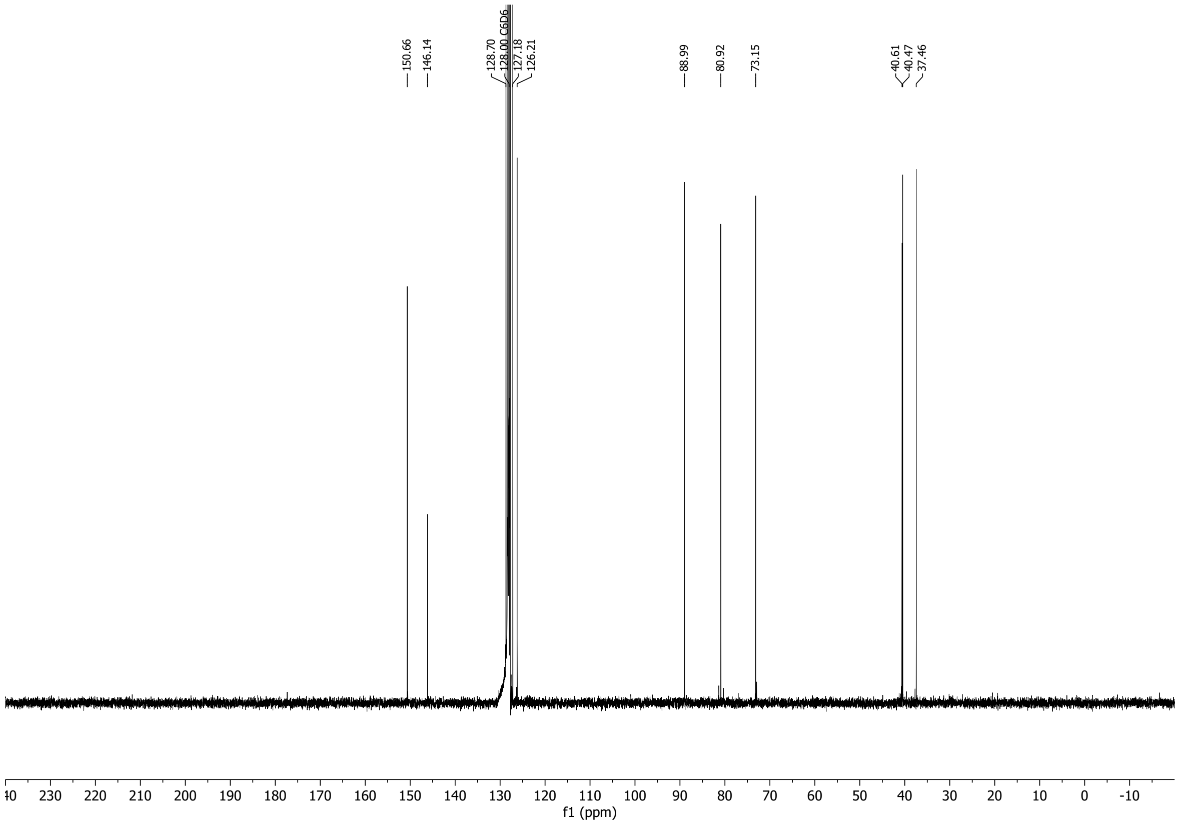

^{13}C NMR (100 MHz, C_6D_6) spectrum of compound **2f**.

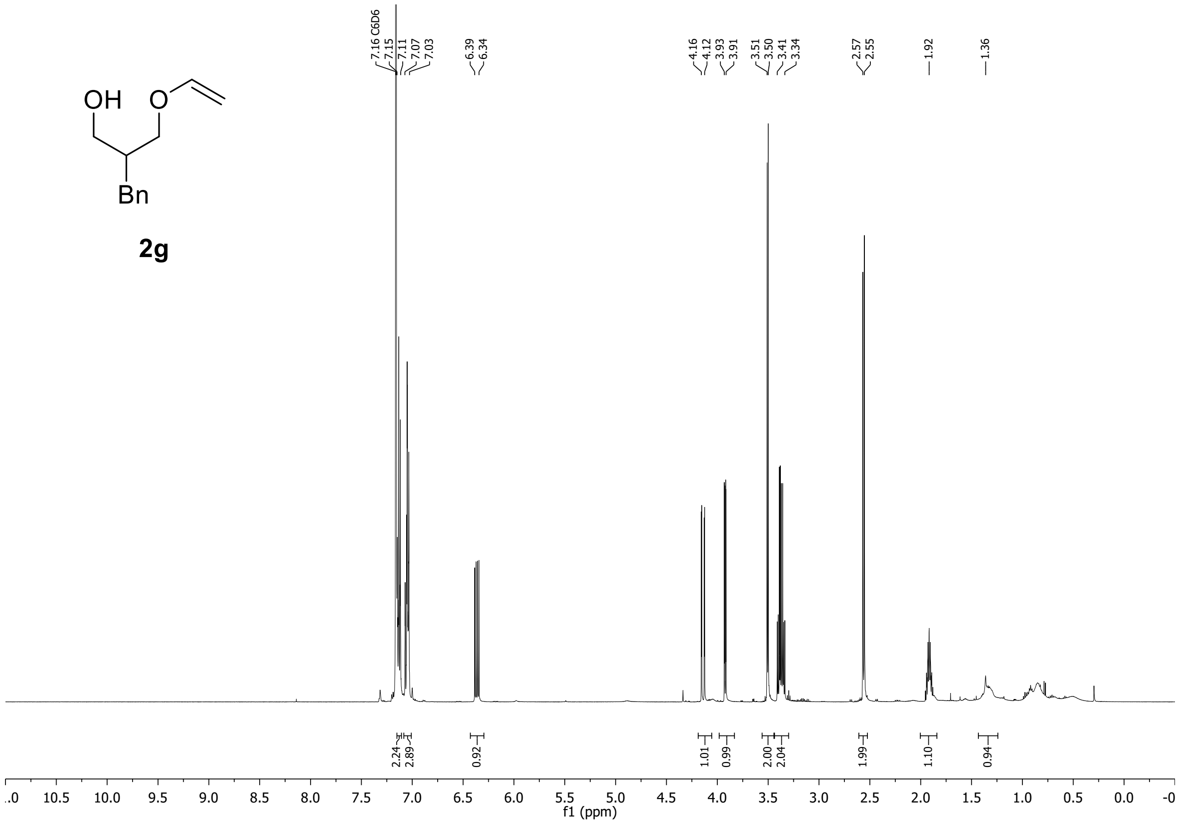

2g

^{1}H NMR (500 MHz, C_6D_6) spectrum of compound **2g**.

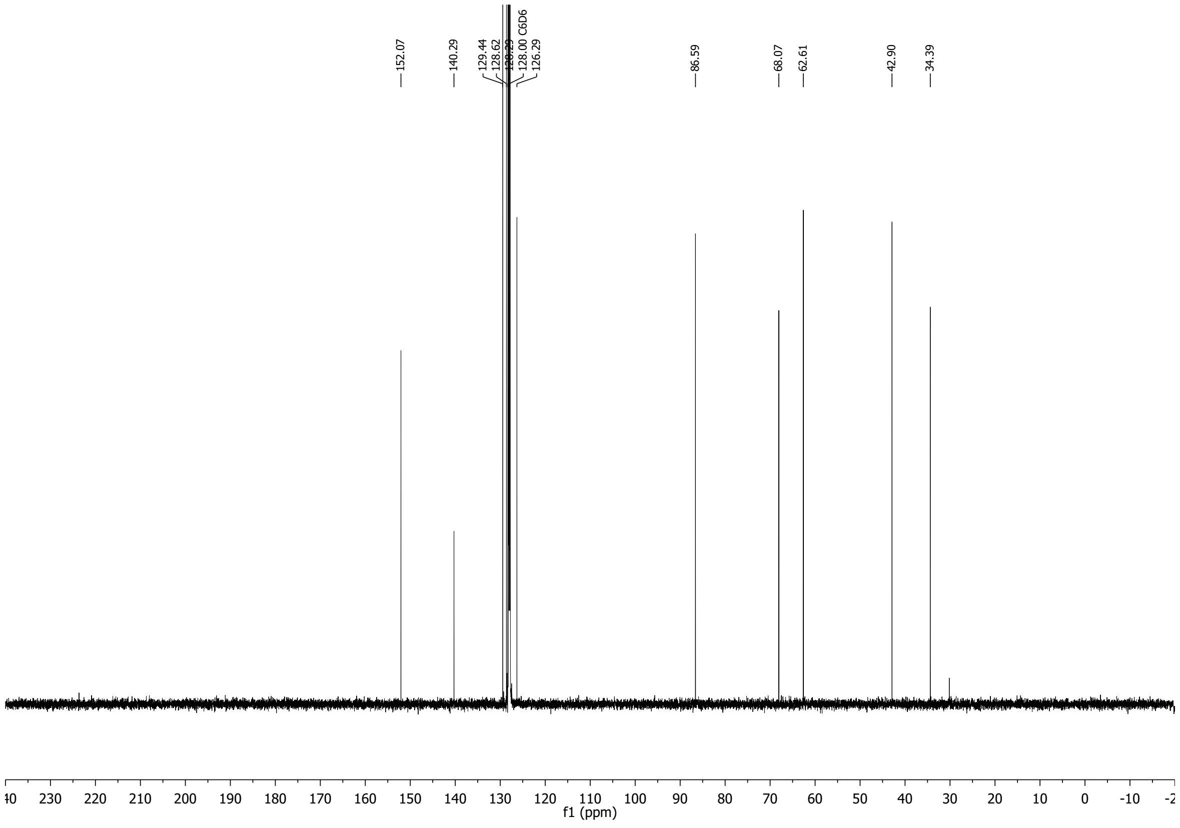

^{13}C NMR (125 MHz, C_6D_6) spectrum of compound **2g**.

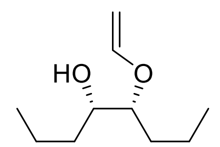

2h

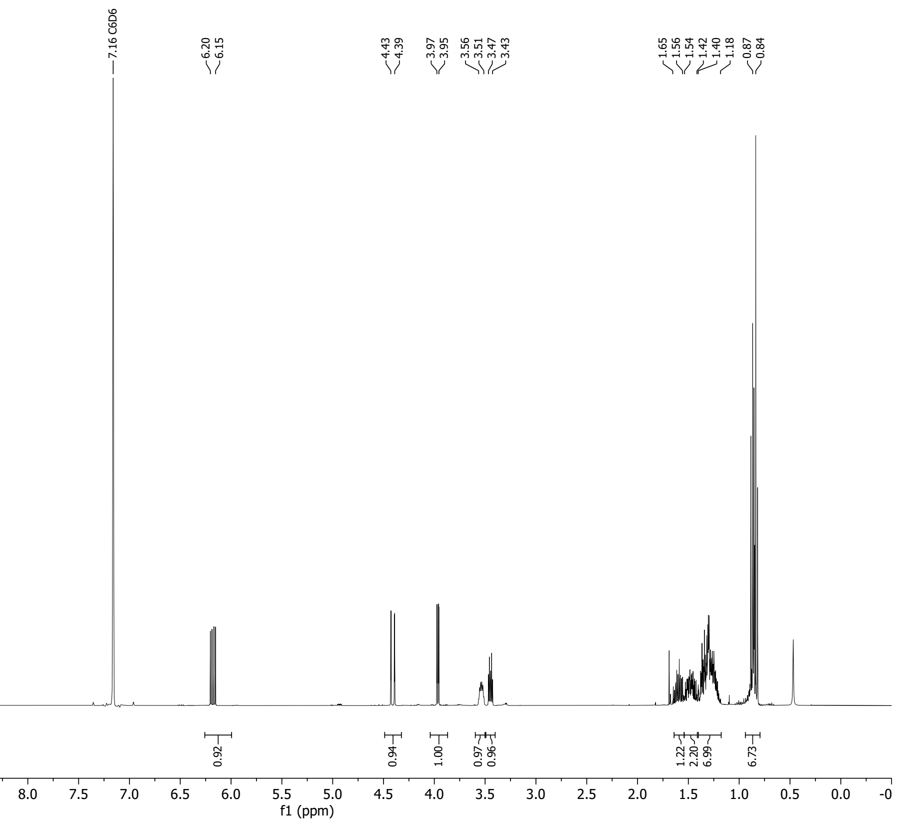

^{1}H NMR (400 MHz, C_6D_6) spectrum of compound **2h**.

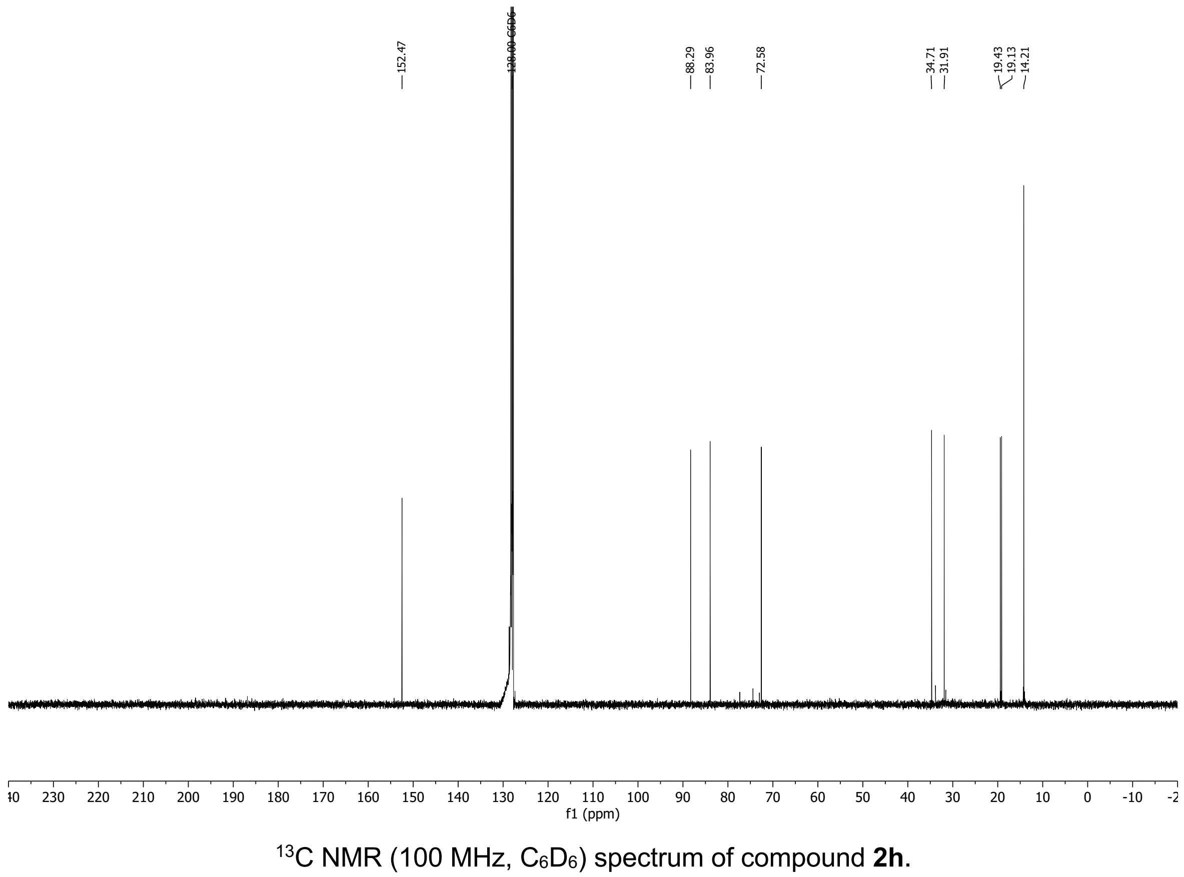

^{13}C NMR (100 MHz, C_6D_6) spectrum of compound **2h**.

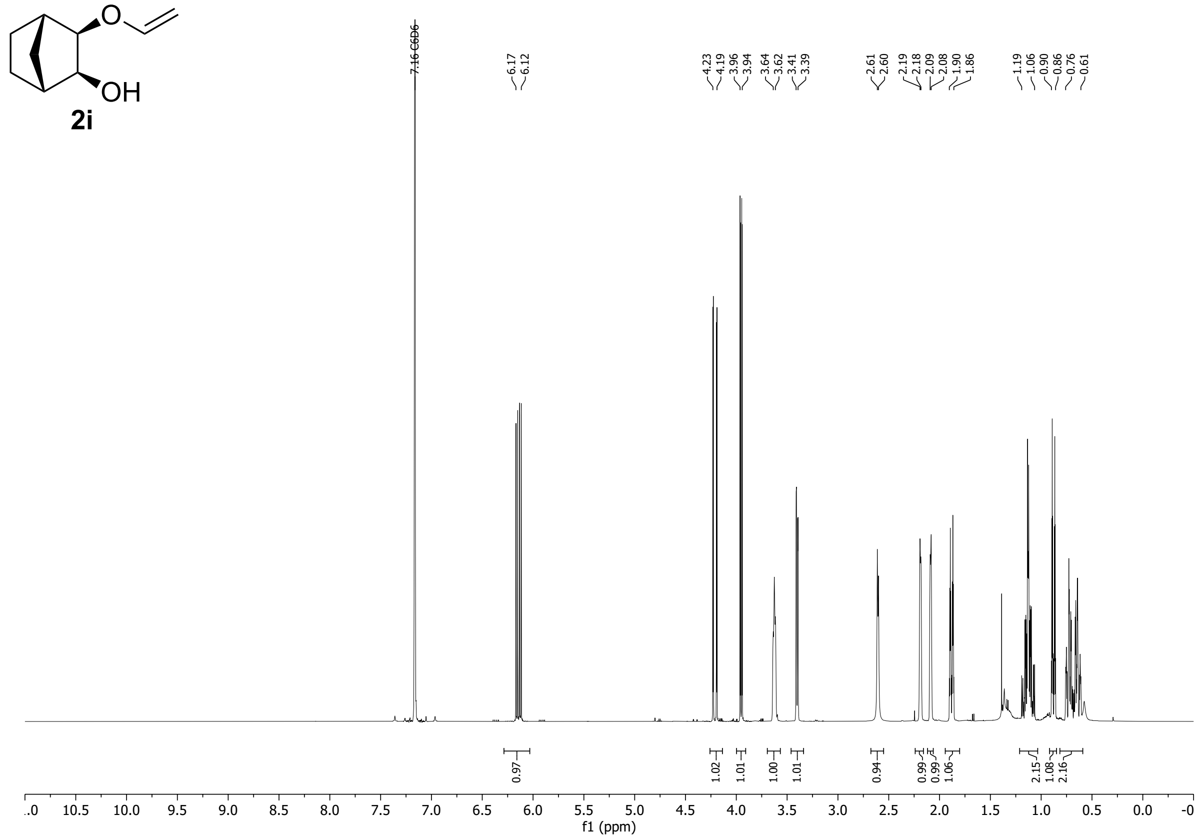

^{1}H NMR (400 MHz, C_6D_6) spectrum of compound **2i**.

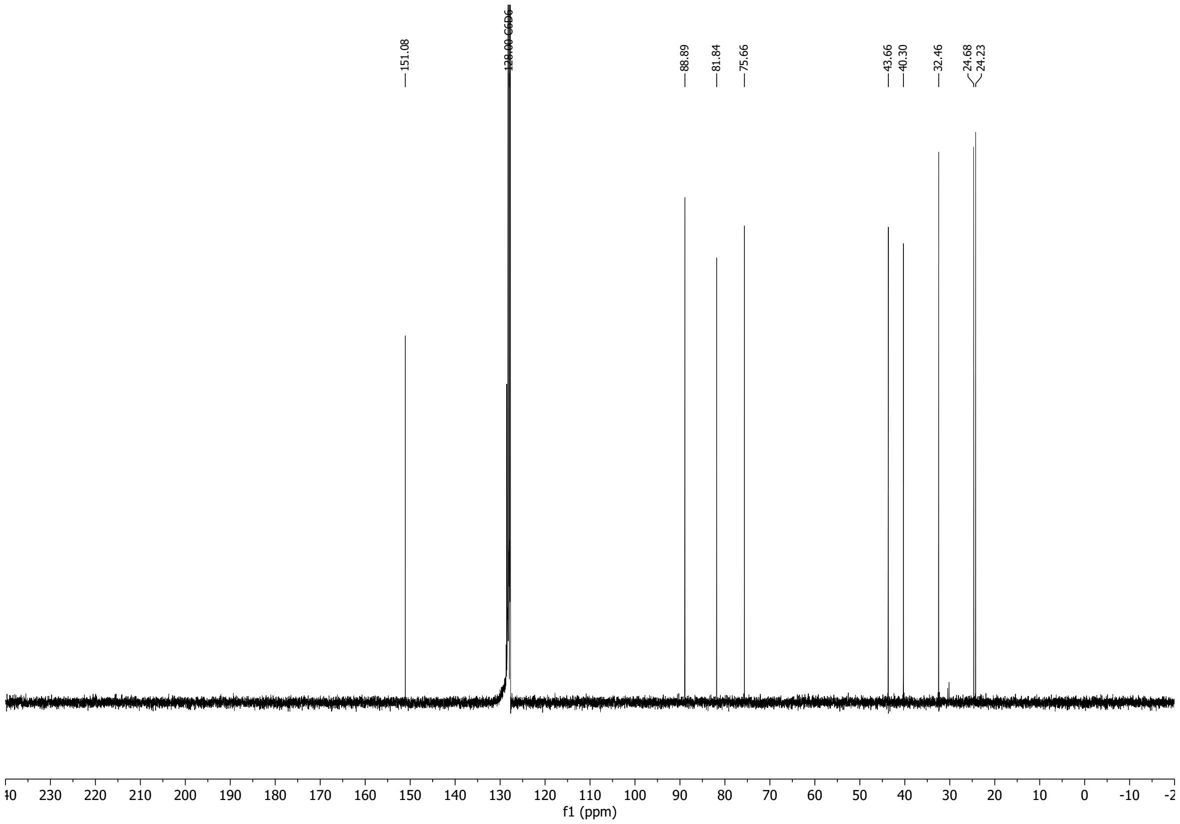

^{13}C NMR (100 MHz, C_6D_6) spectrum of compound **2i**.

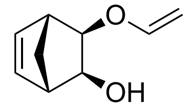

2j
(19:1 mixture with **2i**)

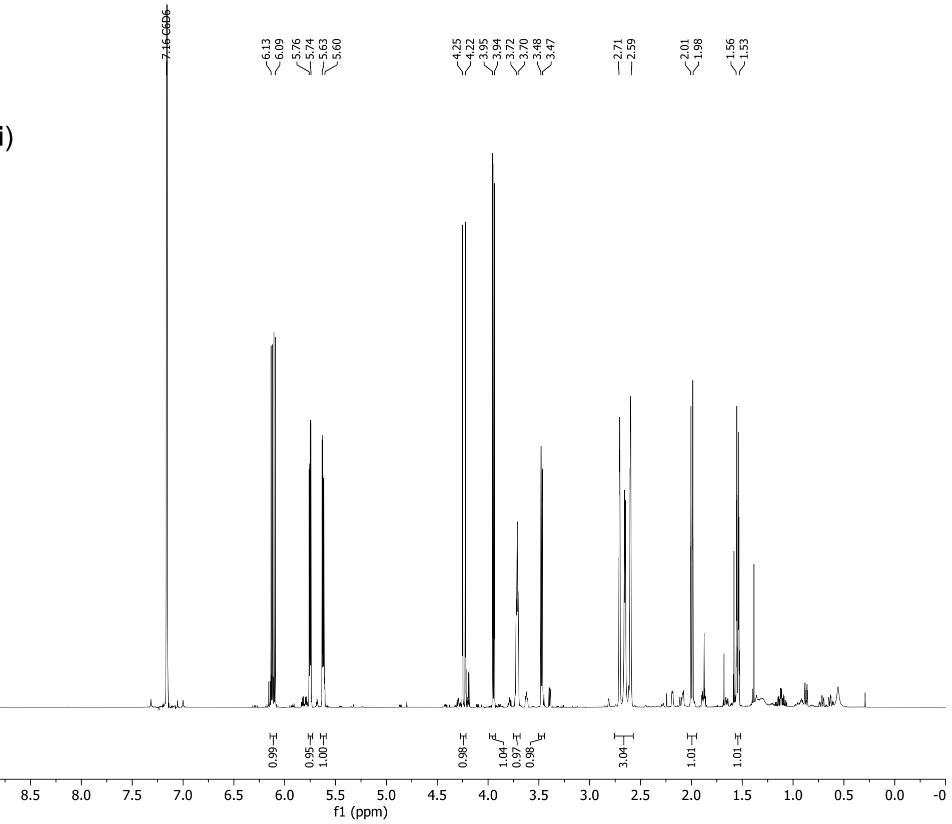

^{1}H NMR (500 MHz, C_6D_6) spectrum of compound **2j**.

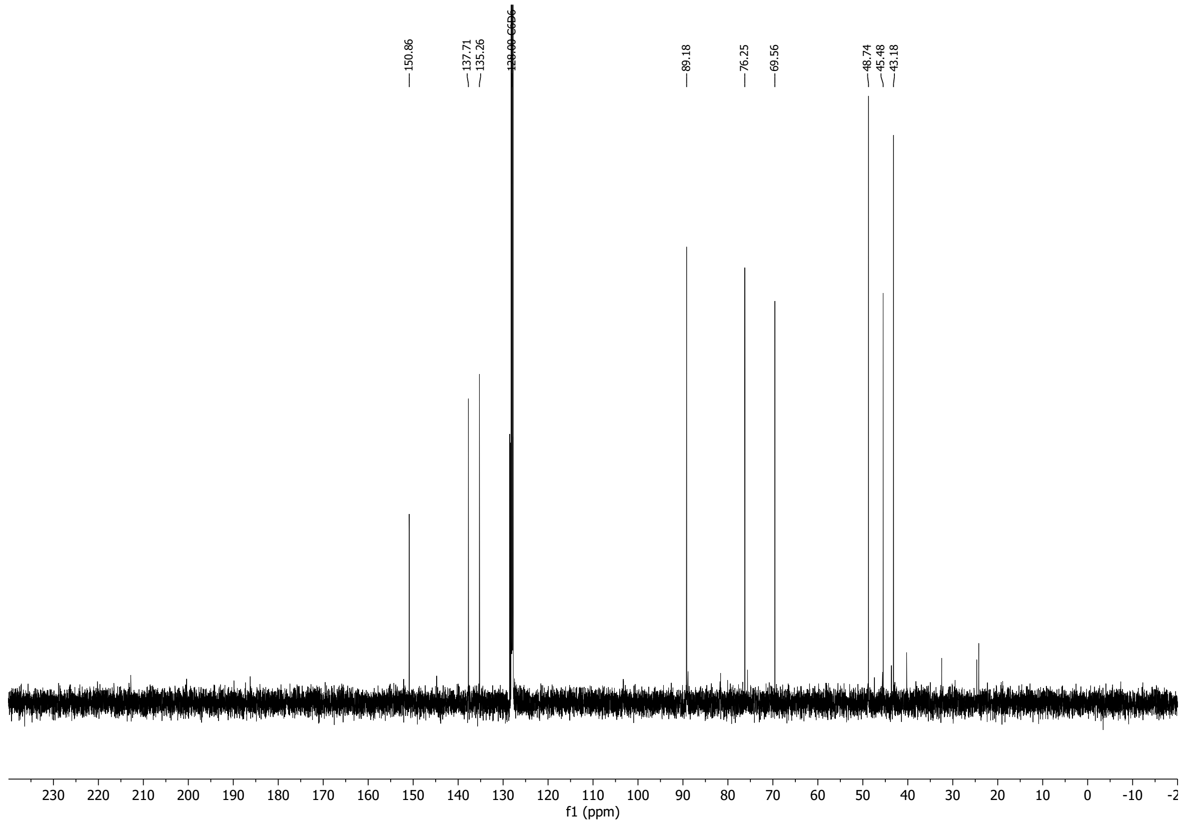

^{13}C NMR (125 MHz, C_6D_6) spectrum of compound **2j**.

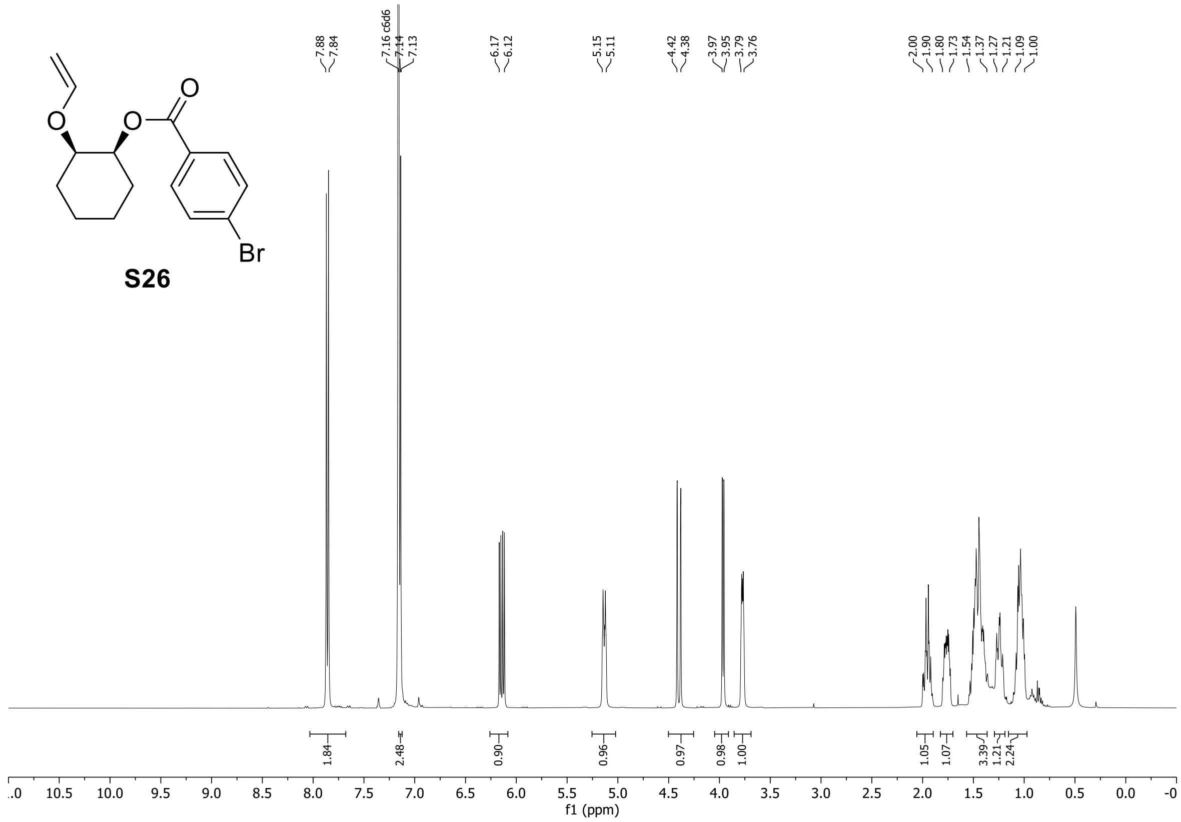

^{1}H NMR (500 MHz, C_6D_6) spectrum of compound **S26**.

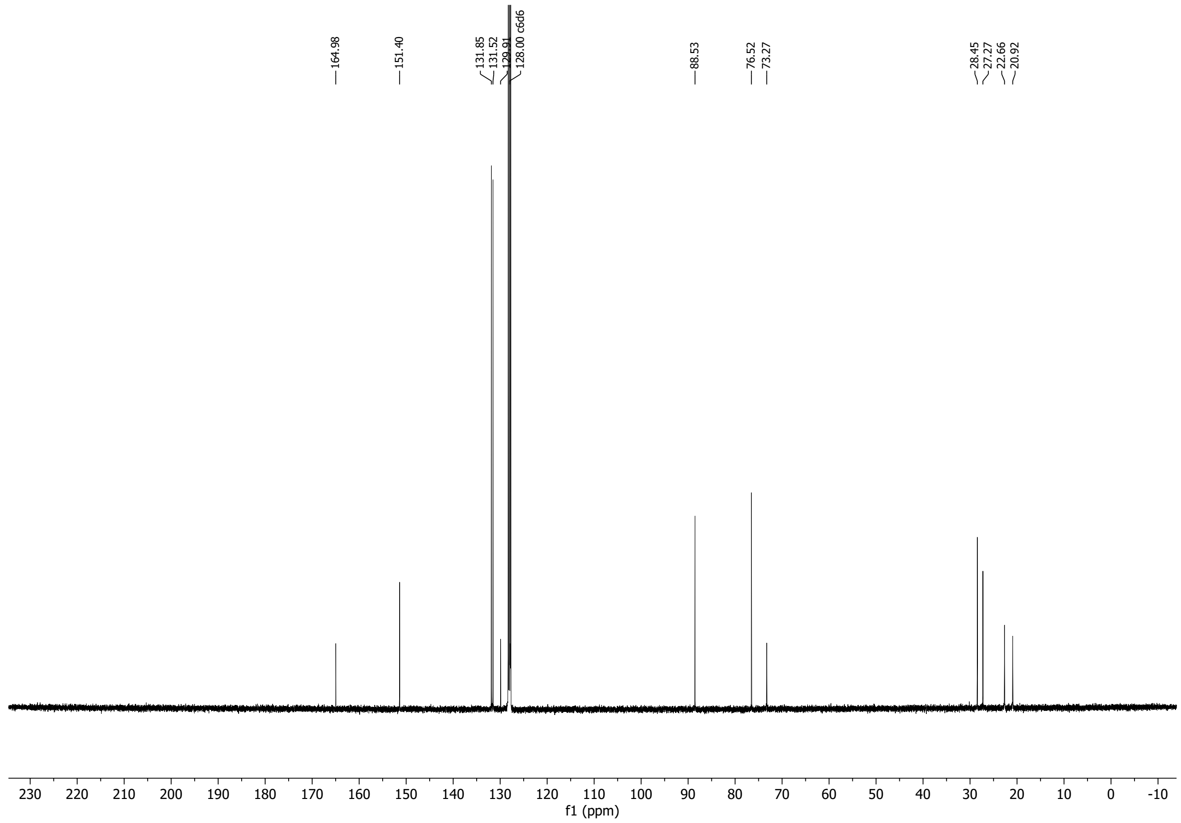

^{13}C NMR (125 MHz, C_6D_6) spectrum of compound **S26**.

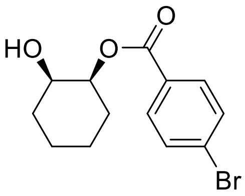

S27

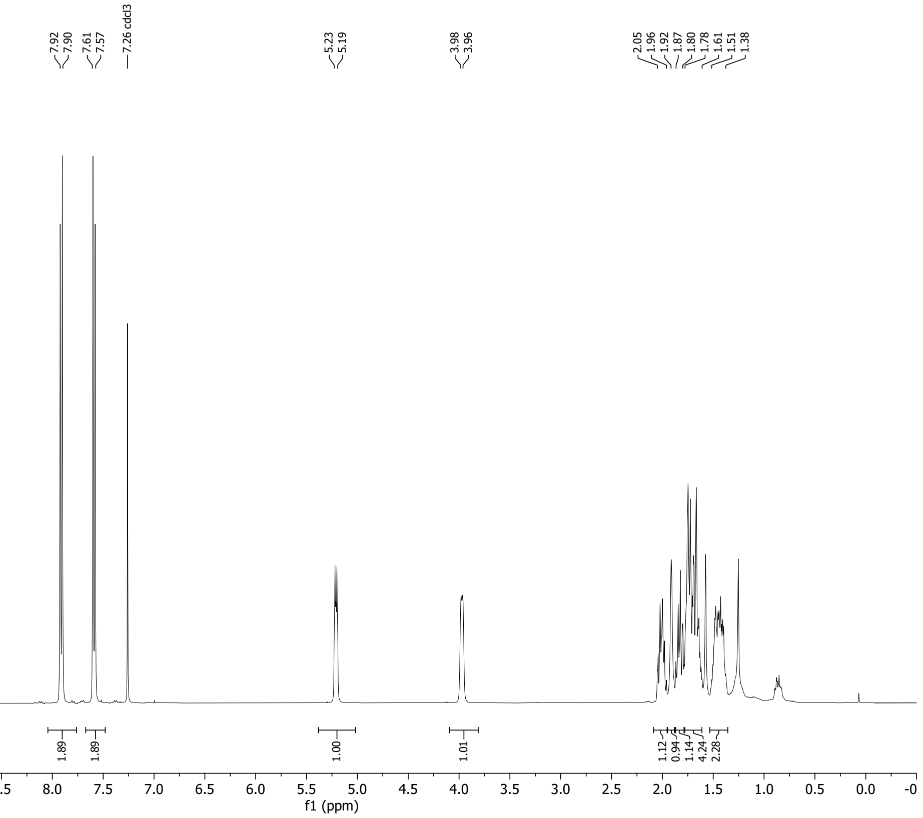

^{1}H NMR (400 MHz, CDCl$_3$) spectrum of compound **S27**.

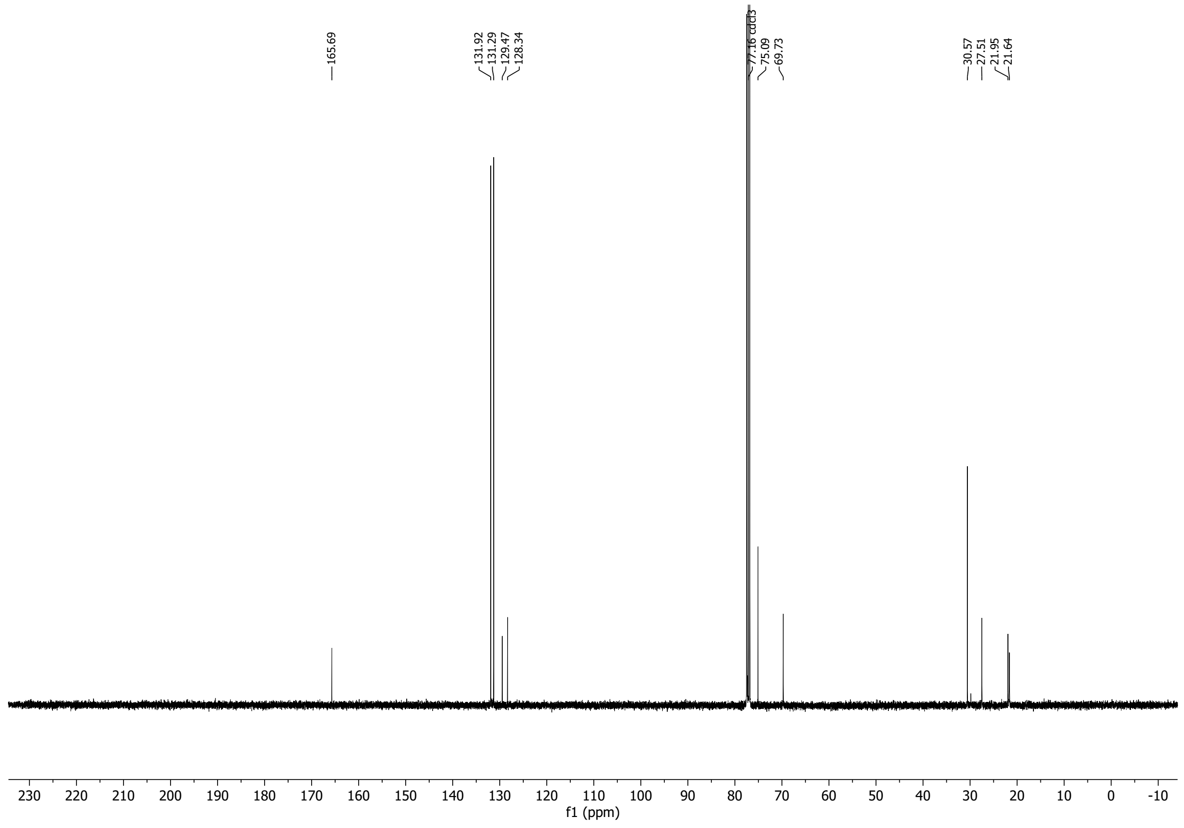

^{13}C NMR (100 MHz, CDCl$_3$) spectrum of compound **S27**.

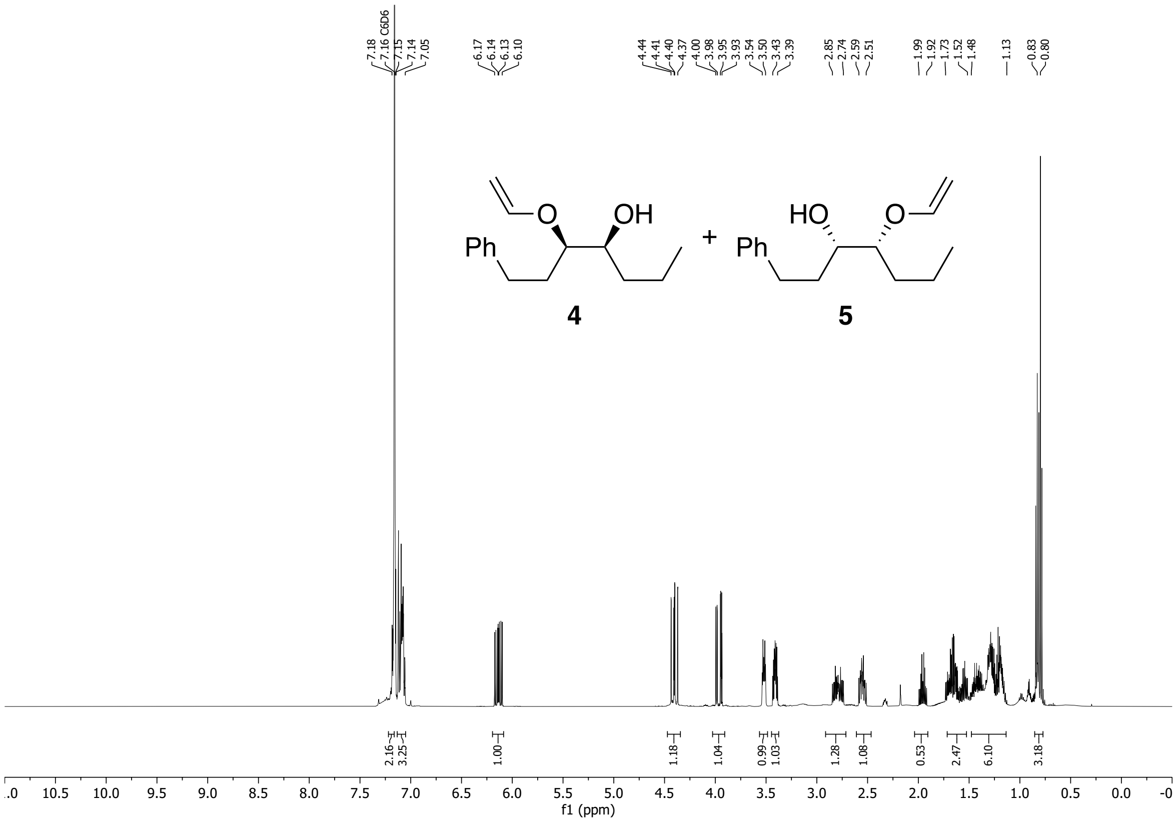

^{1}H NMR (500 MHz, C_6D_6) spectrum of compound **4** and **5**.

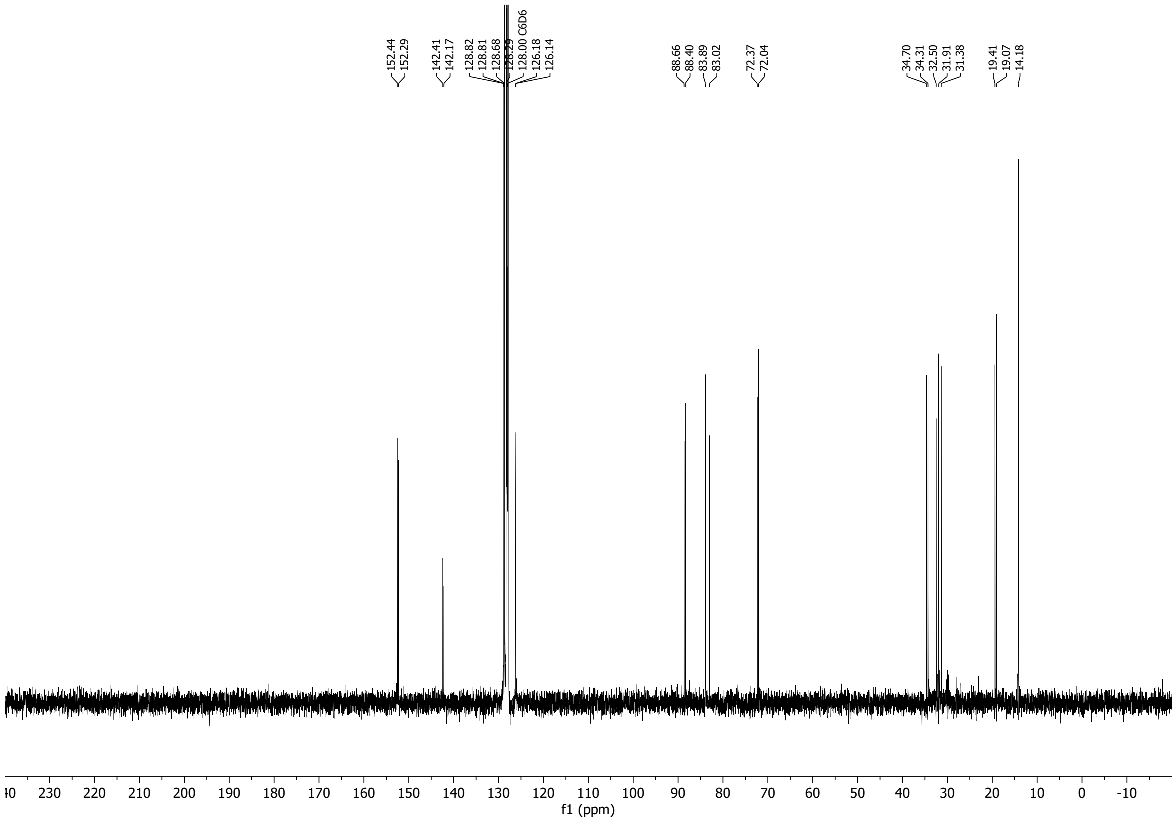

^{13}C NMR (125 MHz, C_6D_6) spectrum of compound **4** and **5**.

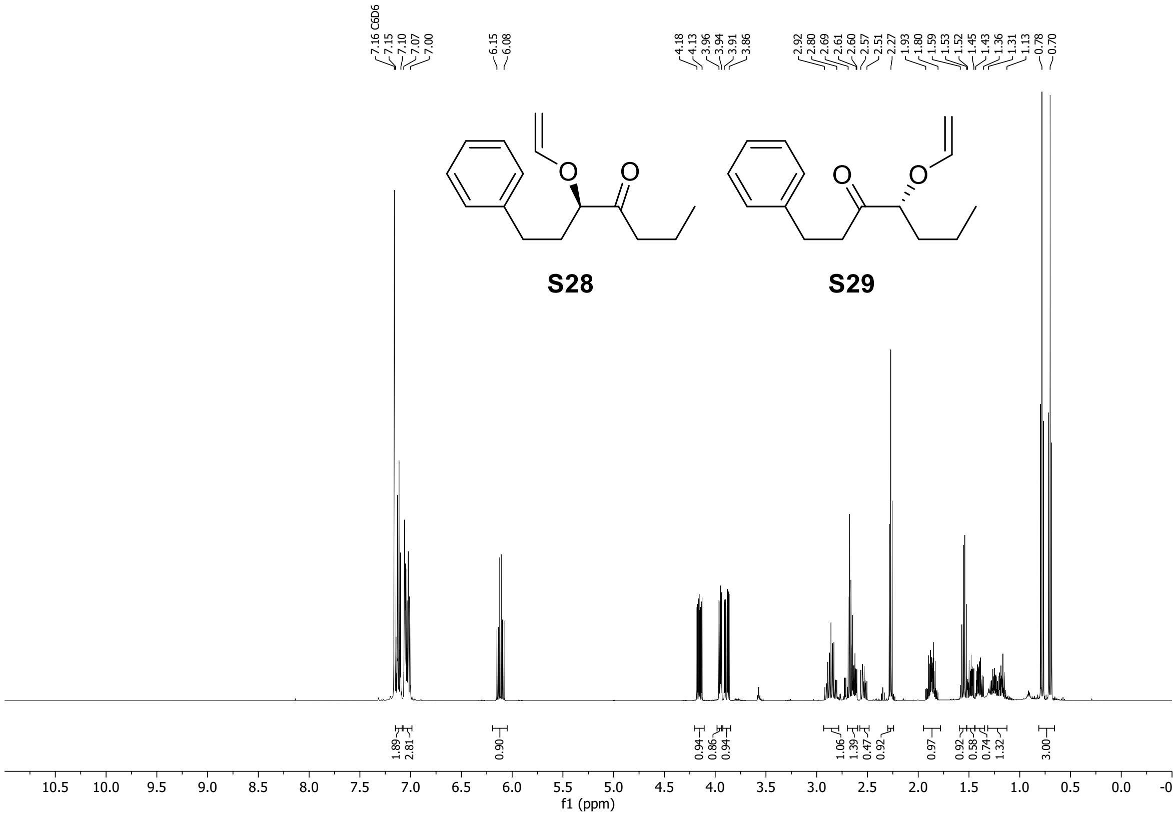

^{1}H NMR (500 MHz, C_6D_6) spectrum of compound **S28** and **S29**.

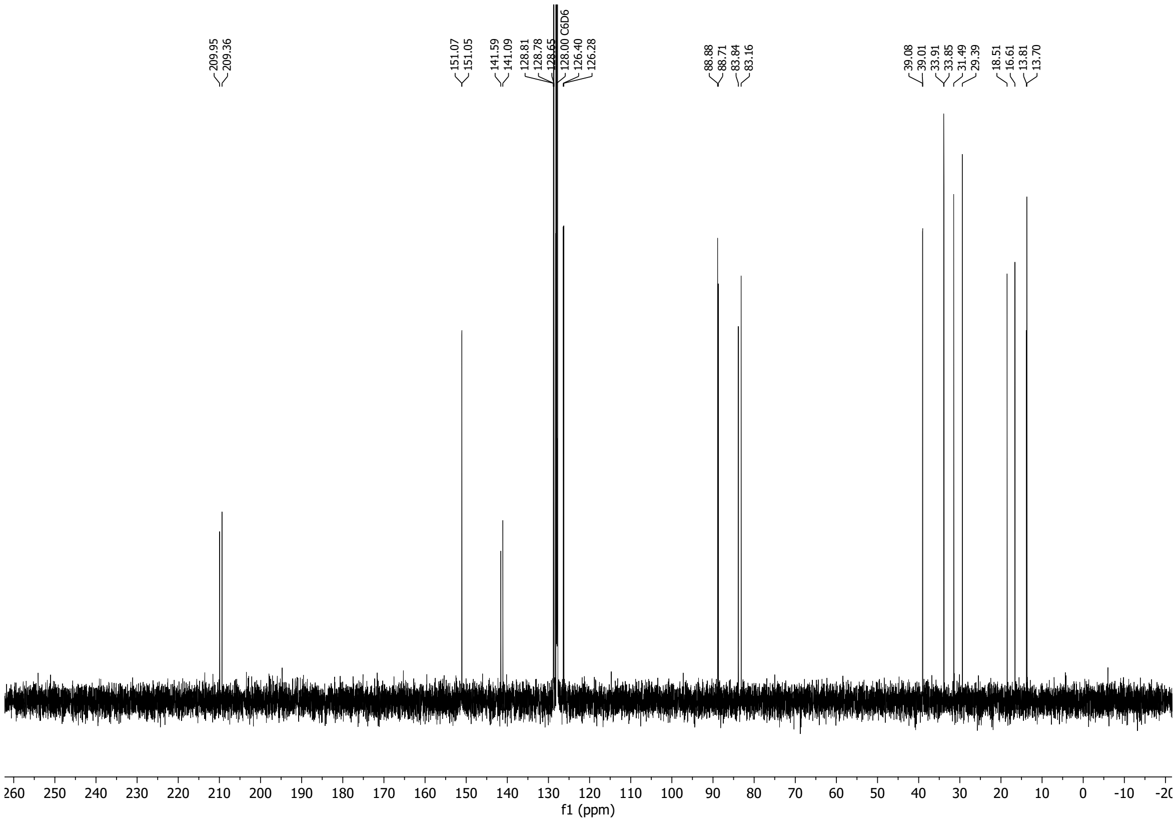

^{13}C NMR (125 MHz, C_6D_6) spectrum of compound **S28** and **S29**.

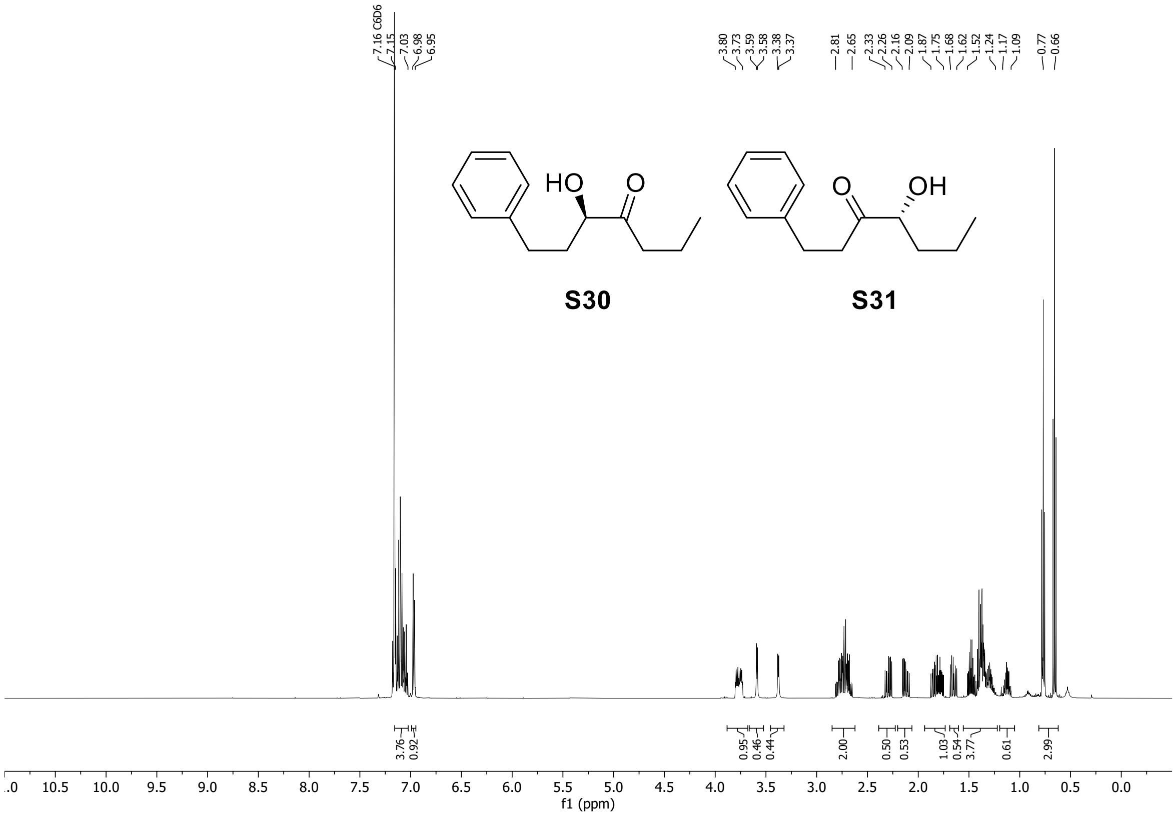

^{1}H NMR (500 MHz, C_6D_6) spectrum of compound **S30** and **S31**.

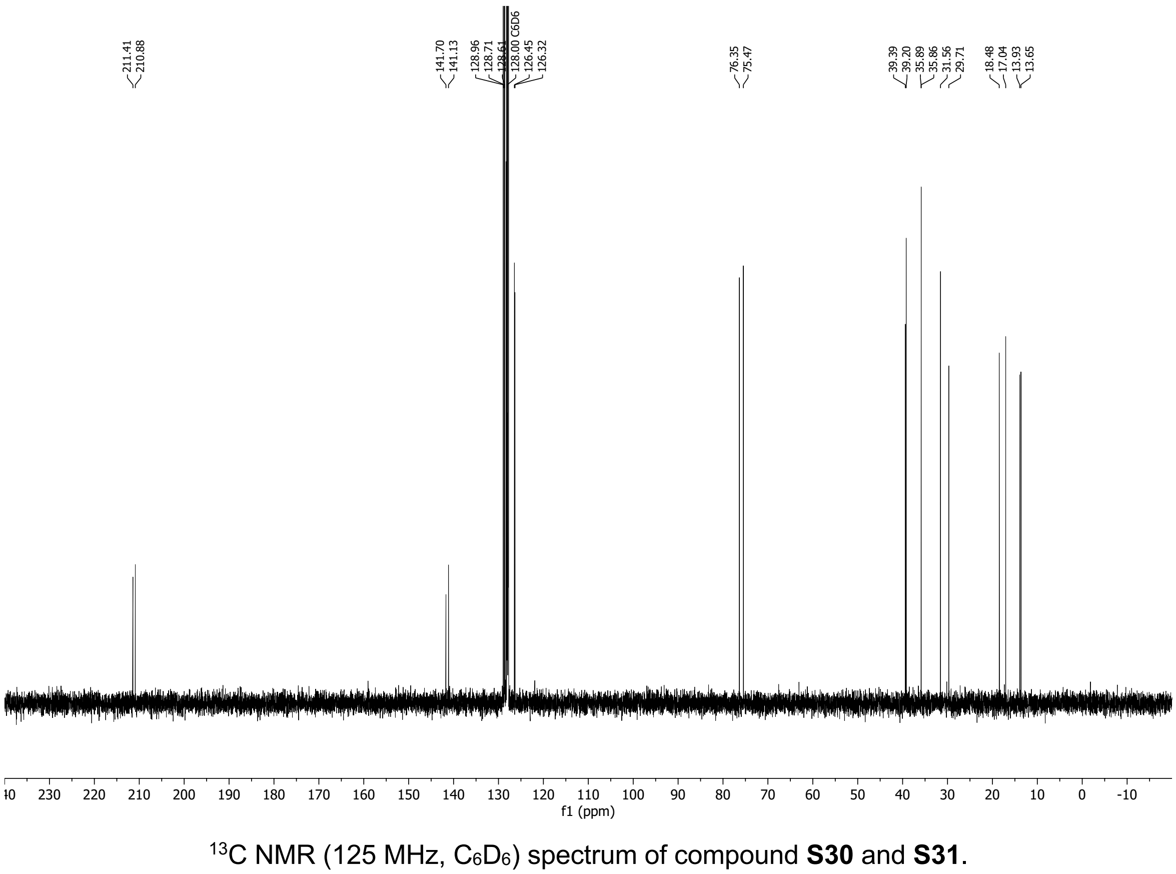

^{13}C NMR (125 MHz, C_6D_6) spectrum of compound **S30** and **S31**.

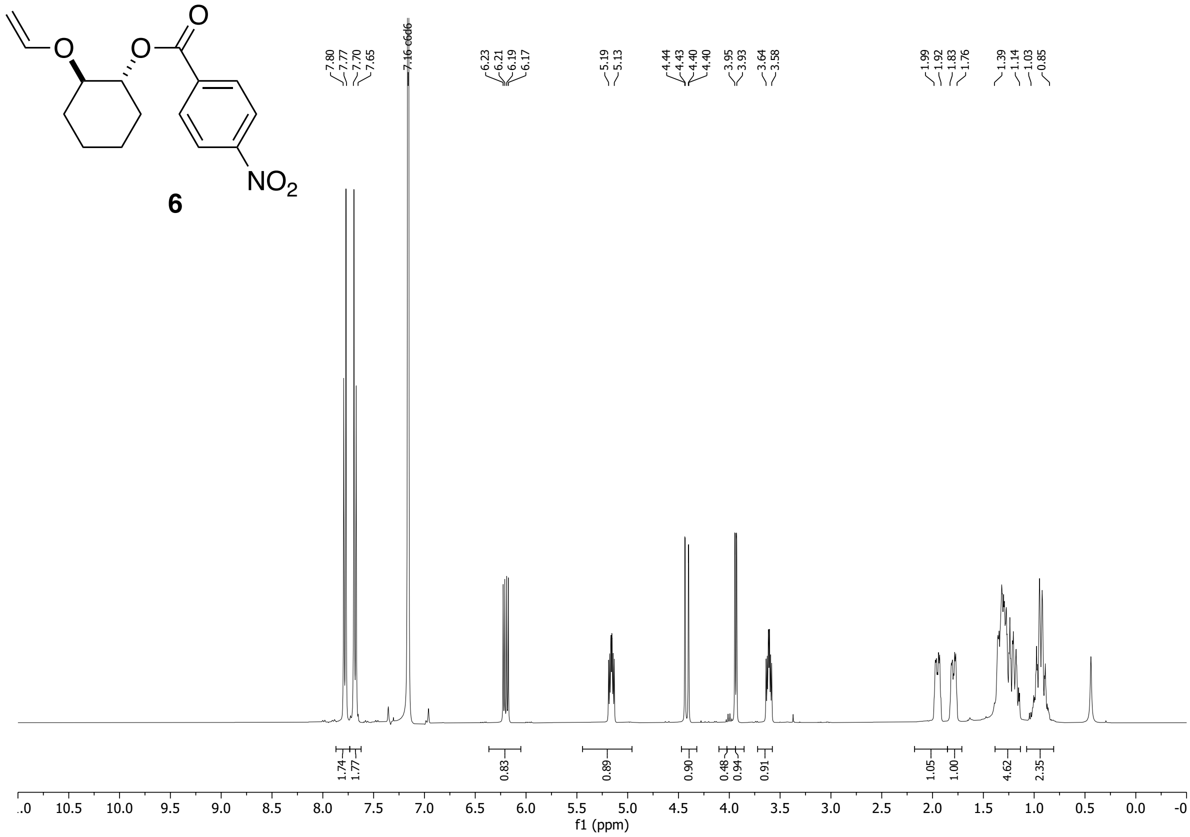

^{1}H NMR (400 MHz, C_6D_6) spectrum of compound **6**.

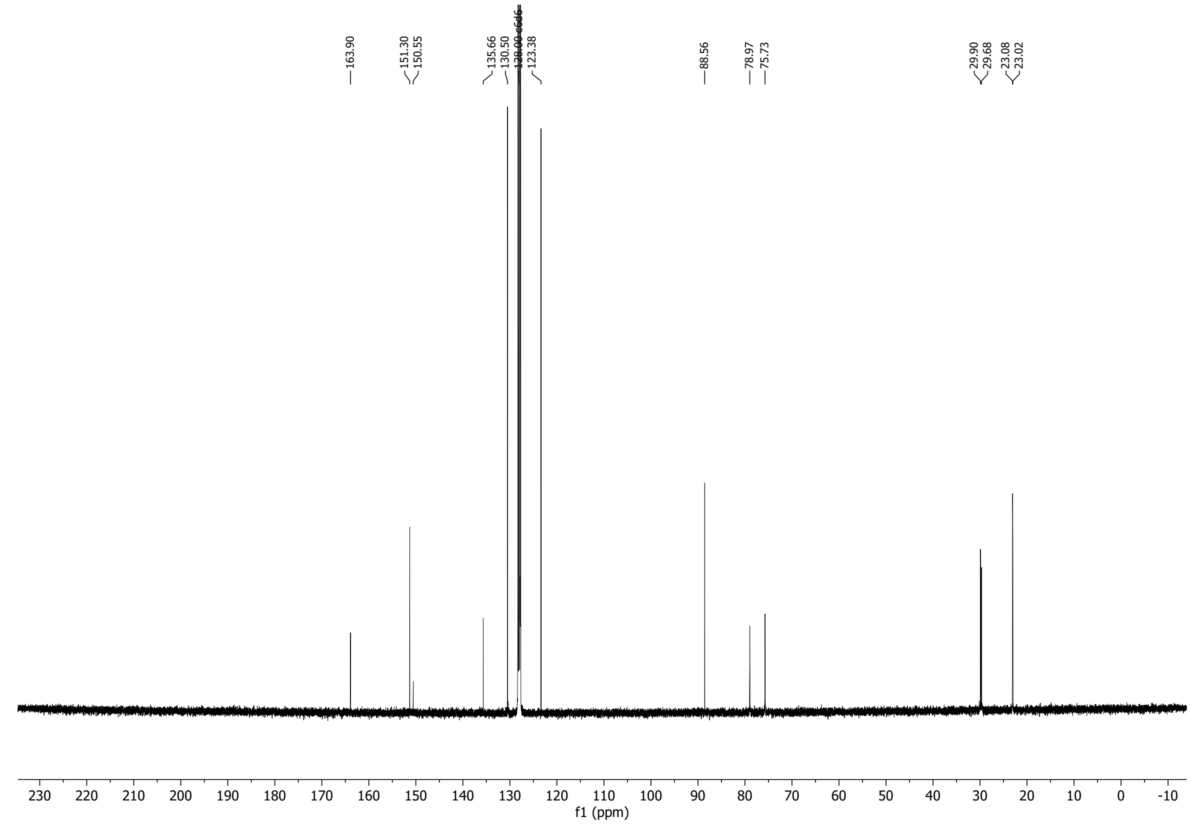

^{13}C NMR (100 MHz, C_6D_6) spectrum of compound **6**.

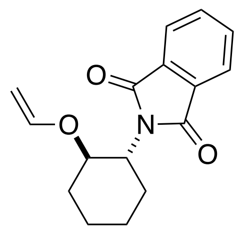

7

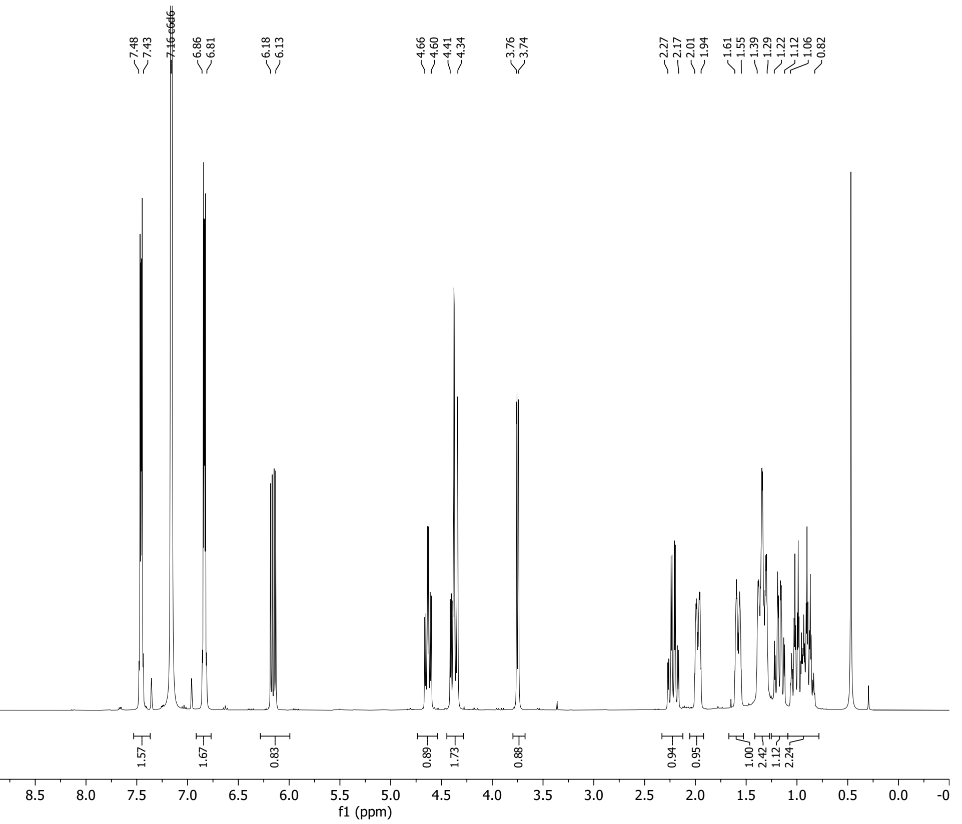

^{1}H NMR (400 MHz, C_6D_6) spectrum of compound **7**.

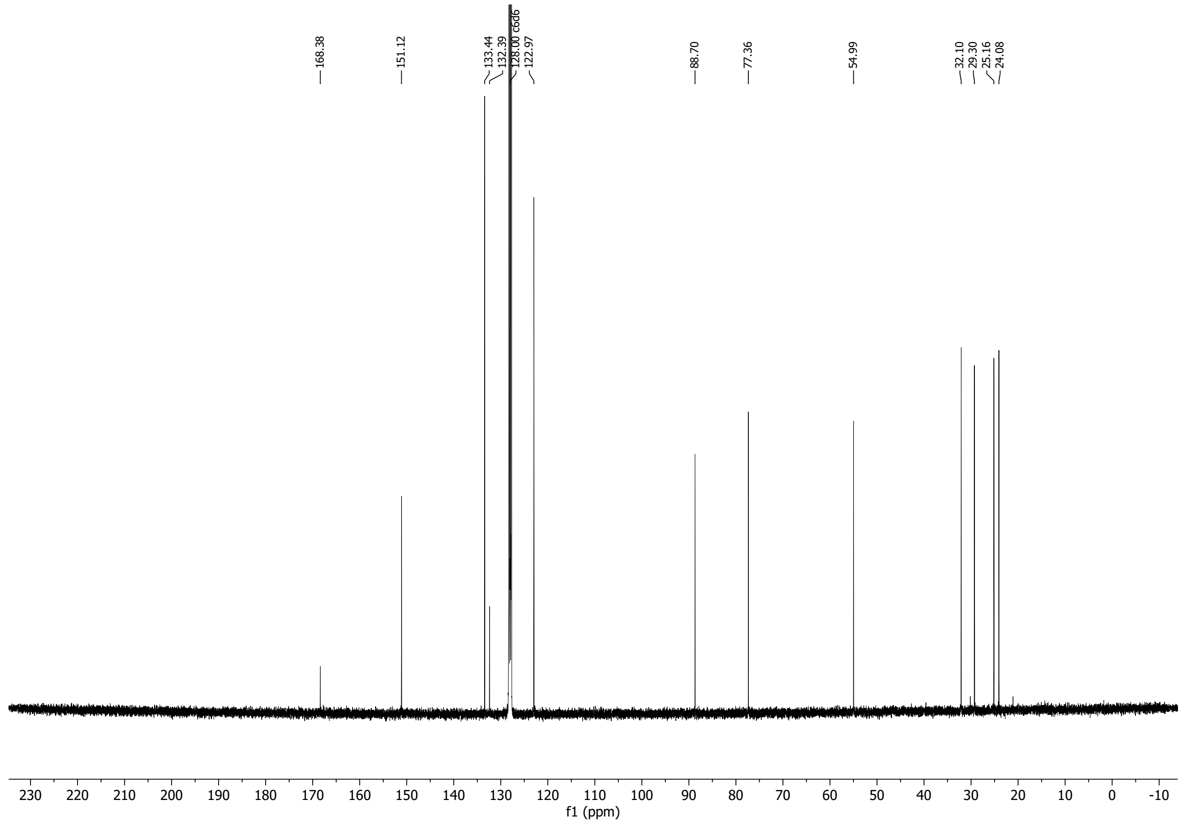

^{13}C NMR (100 MHz, C_6D_6) spectrum of compound **7**.

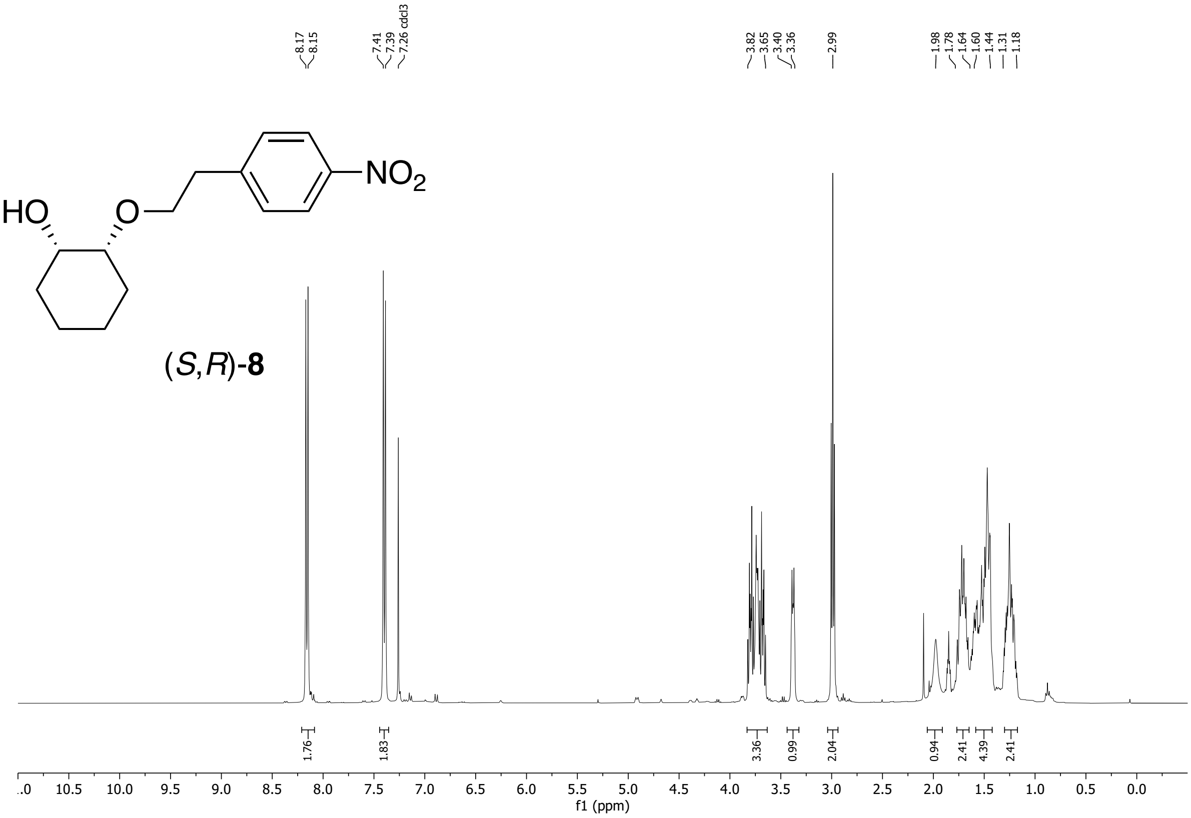

^{1}H NMR (400 MHz, $CDCl_3$) spectrum of compound (*S*,*R*)-**8**.

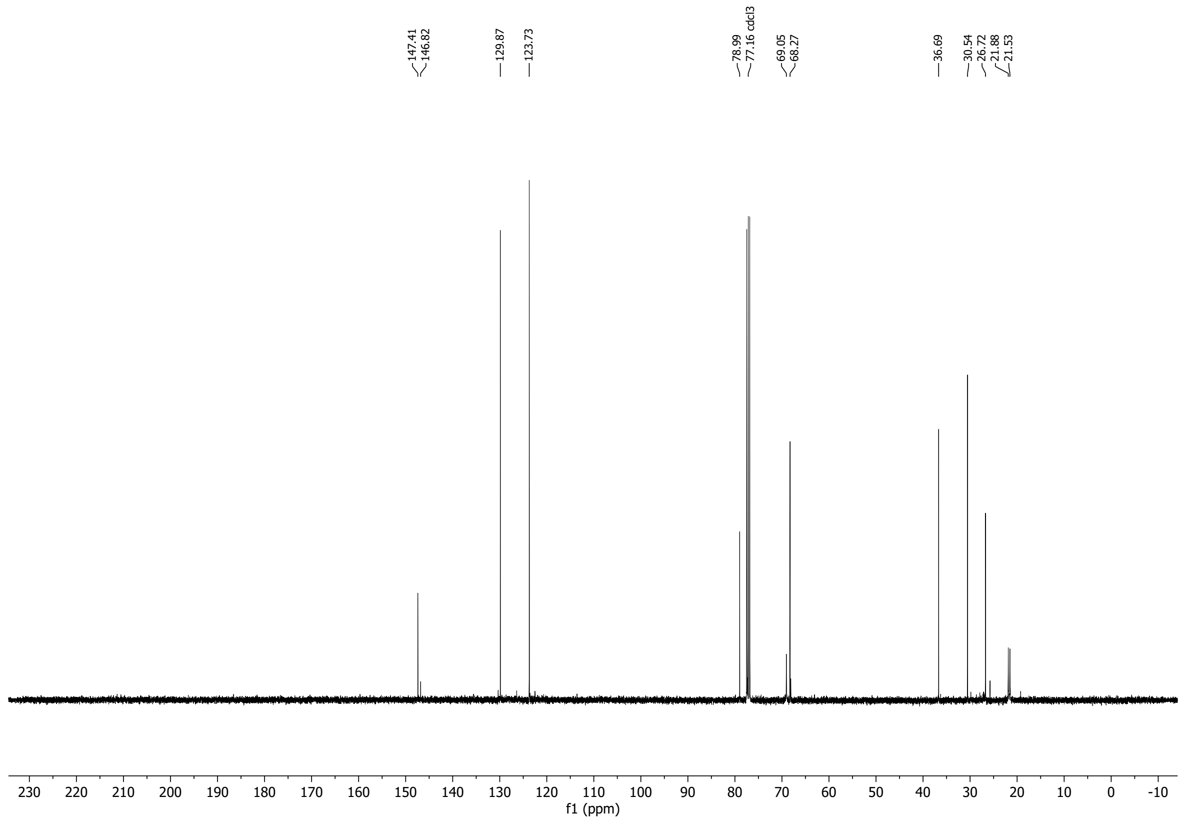

^{13}C NMR (100 MHz, $CDCl_3$) spectrum of compound (*S*,*R*)-**8**.

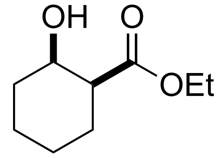

S38

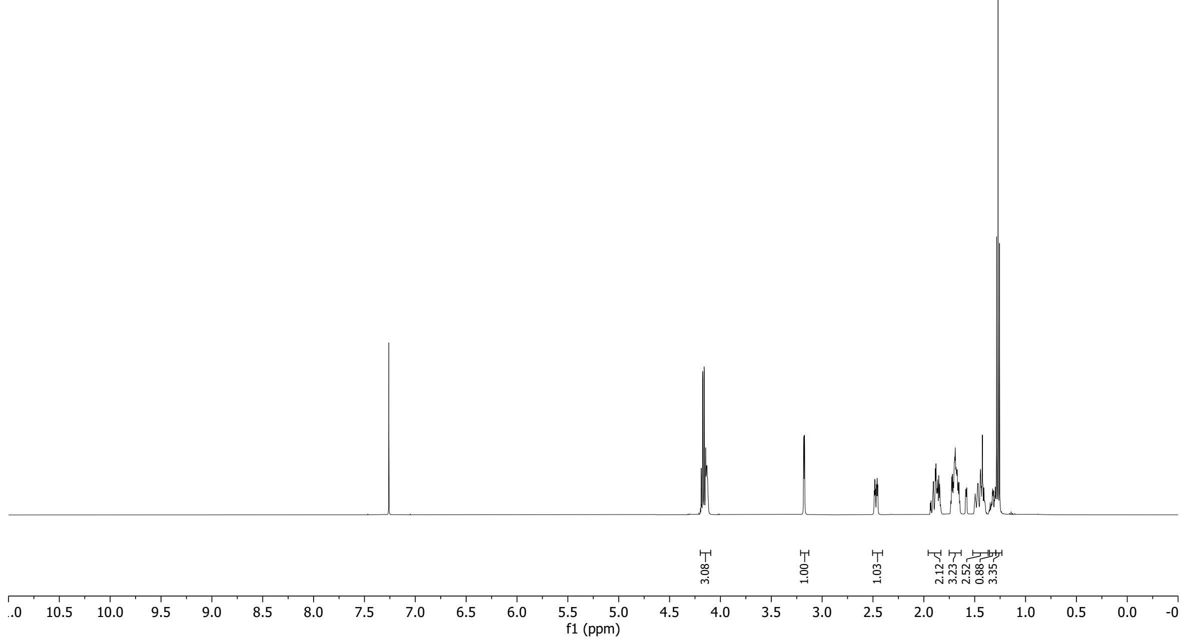

^{1}H NMR (500 MHz, $CDCl_3$) spectrum of compound **S38**.

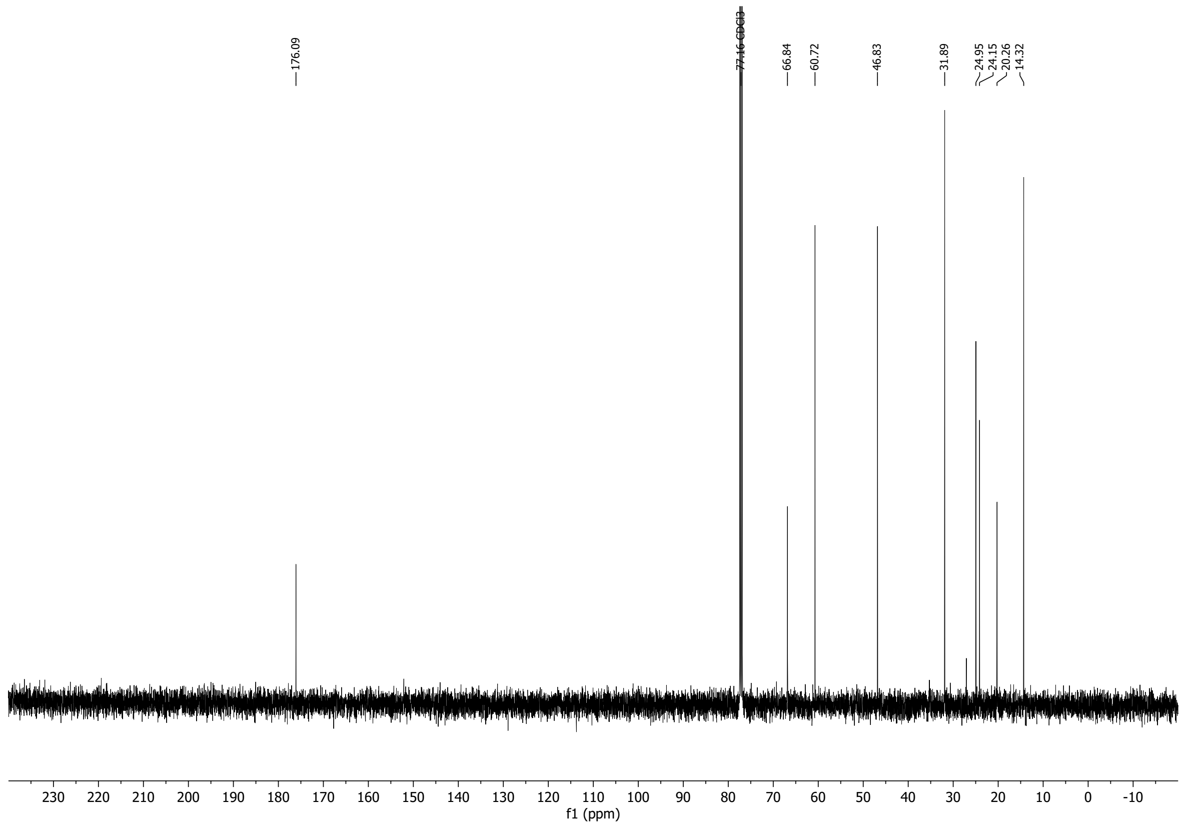

^{13}C NMR (125 MHz, $CDCl_3$) spectrum of compound **S38**.

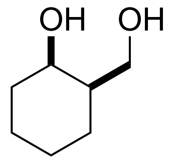

S39

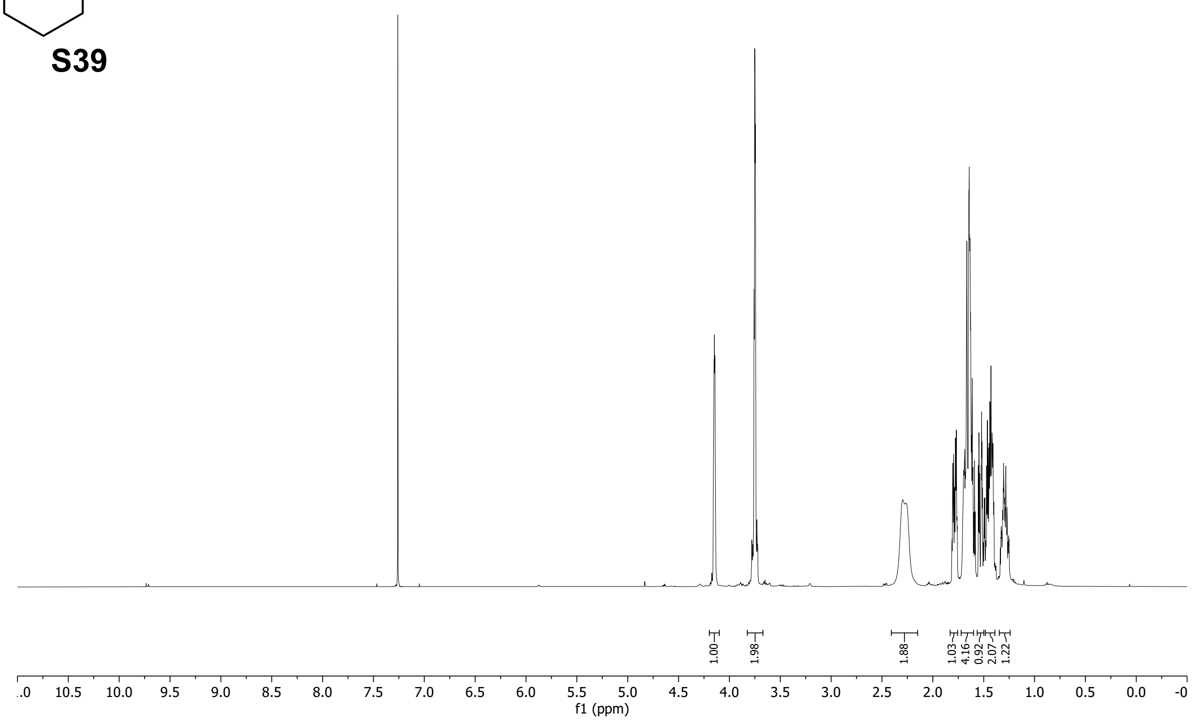

^{1}H NMR (500 MHz, $CDCl_3$) spectrum of compound **S39**.

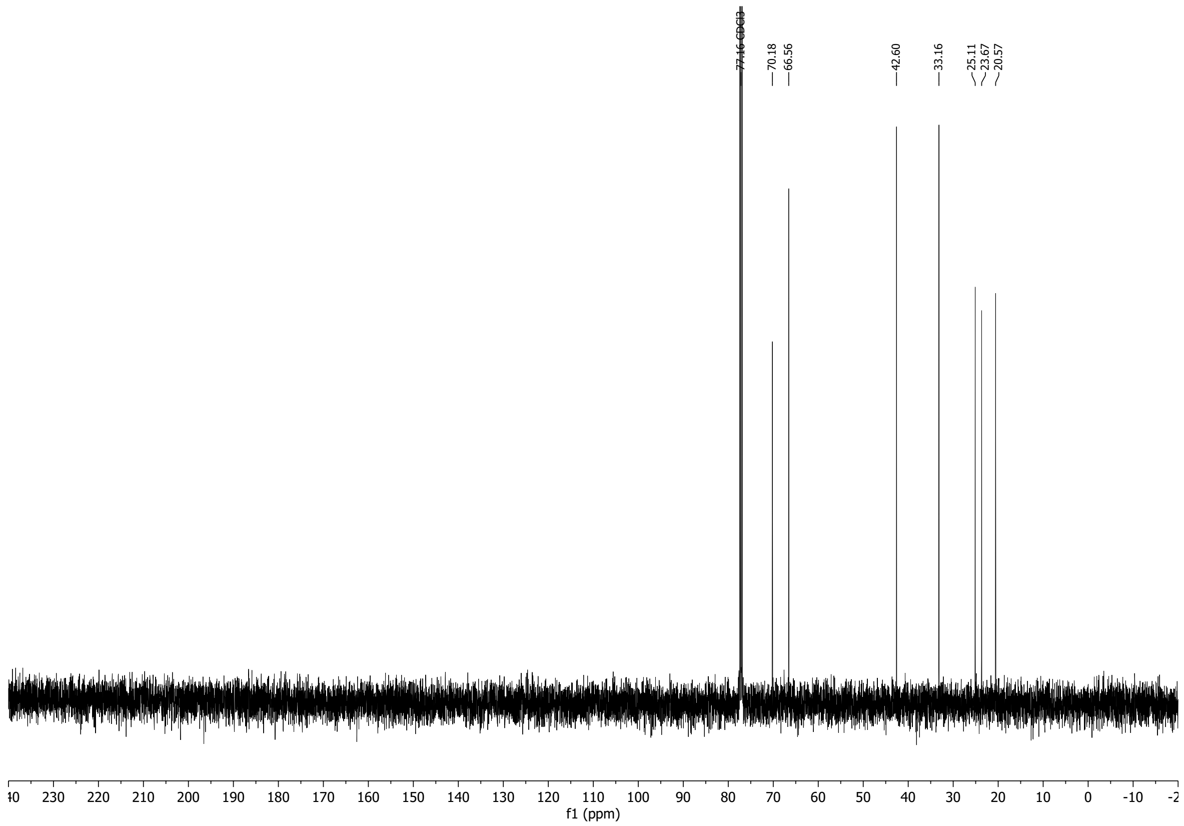

^{13}C NMR (125 MHz, $CDCl_3$) spectrum of compound **S39**.

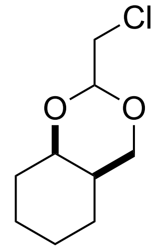

S40

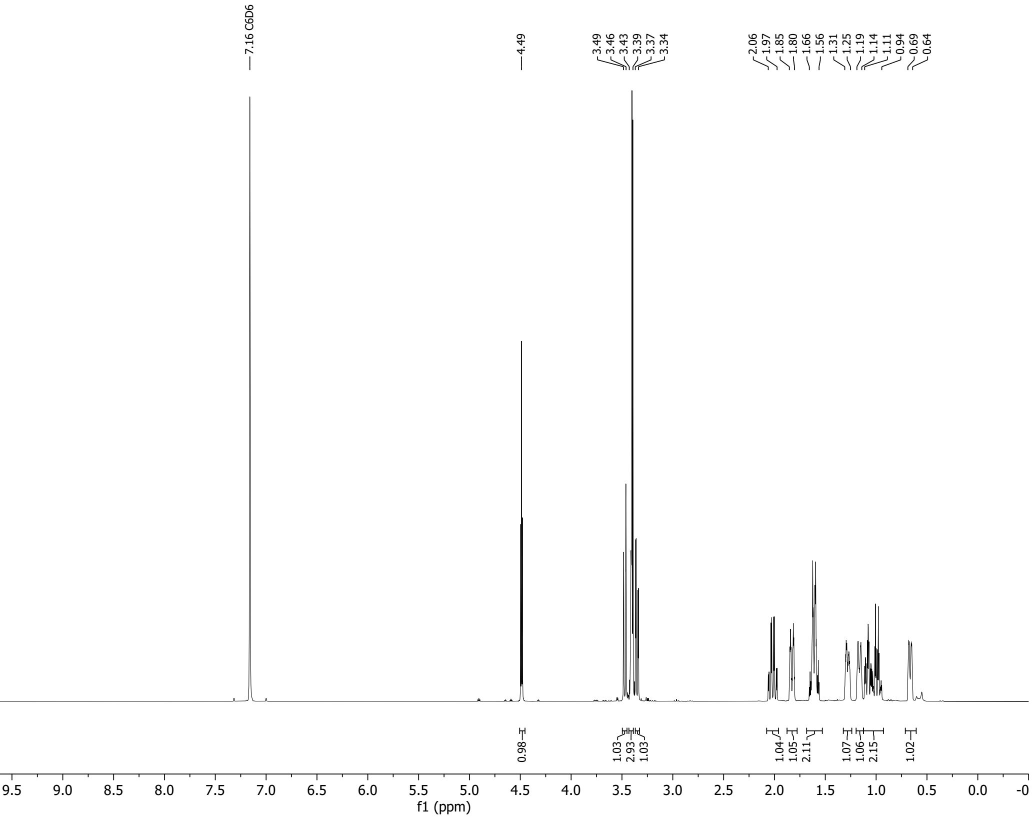

^{1}H NMR (500 MHz, C_6D_6) spectrum of compound **S40**.

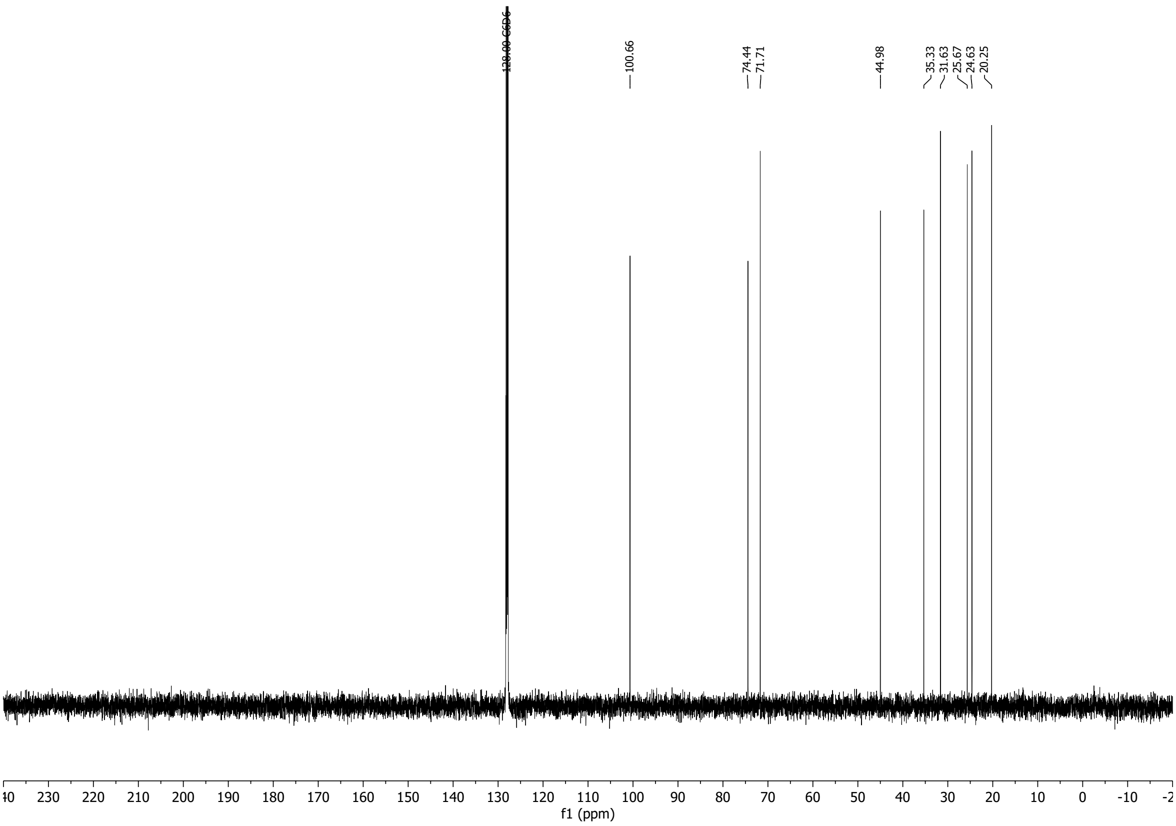

^{13}C NMR (125 MHz, C_6D_6) spectrum of compound **S40**.

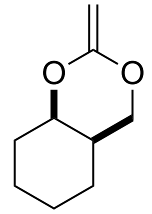

S32

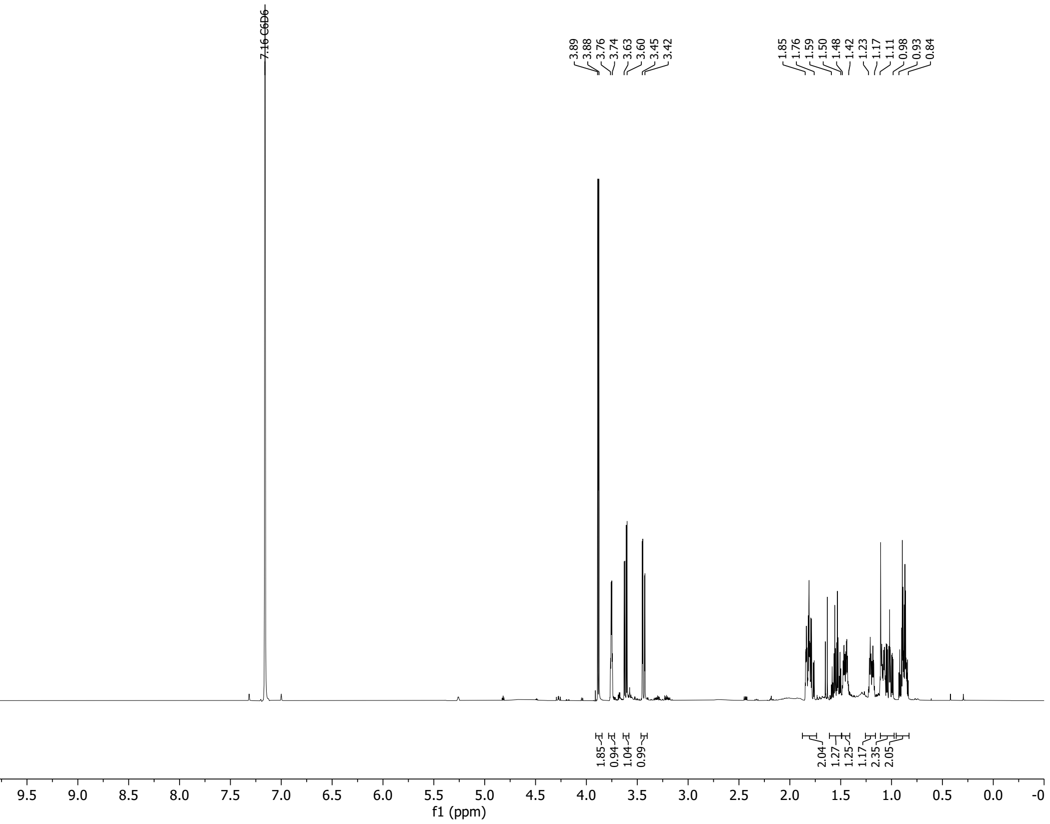

^{1}H NMR (500 MHz, C_6D_6) spectrum of compound **S32**.

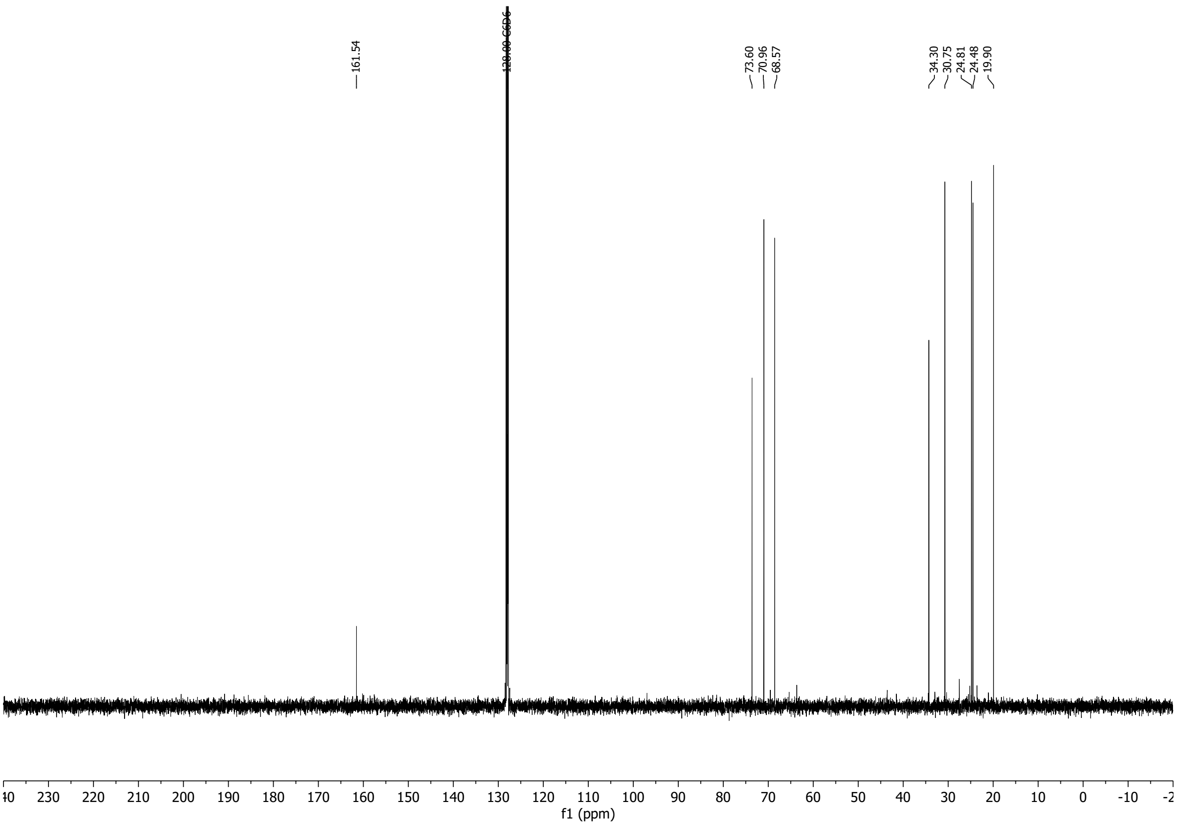

^{13}C NMR (125 MHz, C_6D_6) spectrum of compound **S32**.

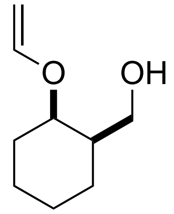

S33

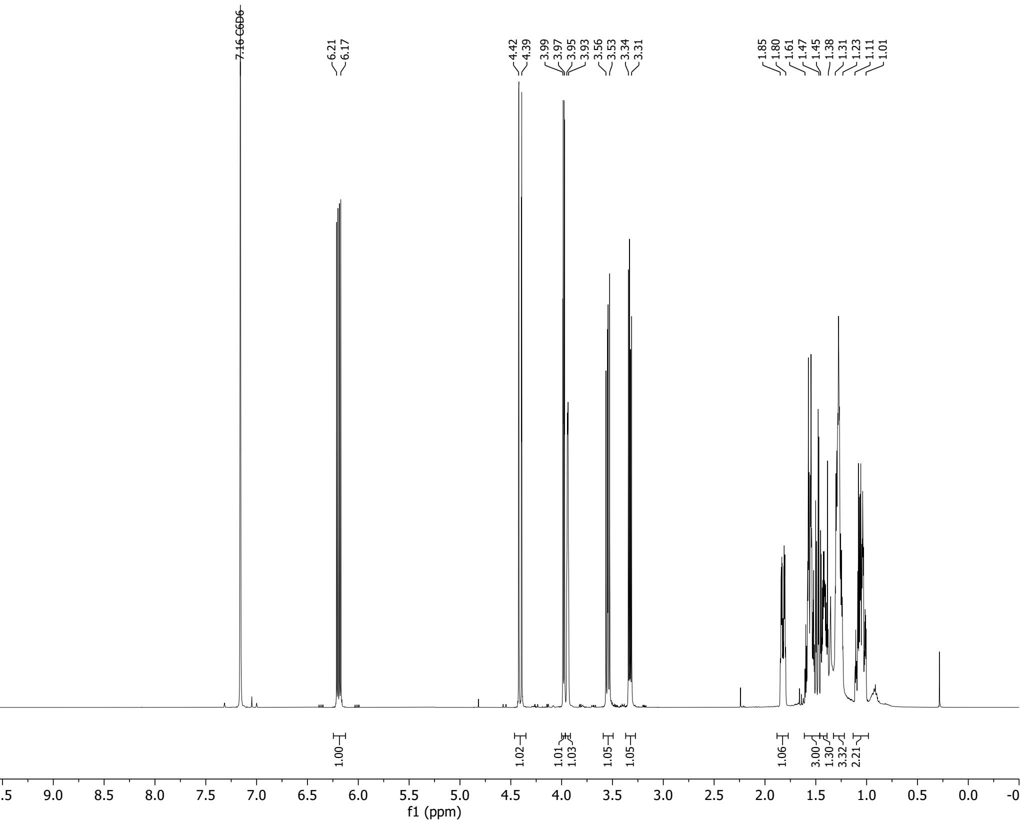

^{1}H NMR (500 MHz, C_6D_6) spectrum of compound **S33**.

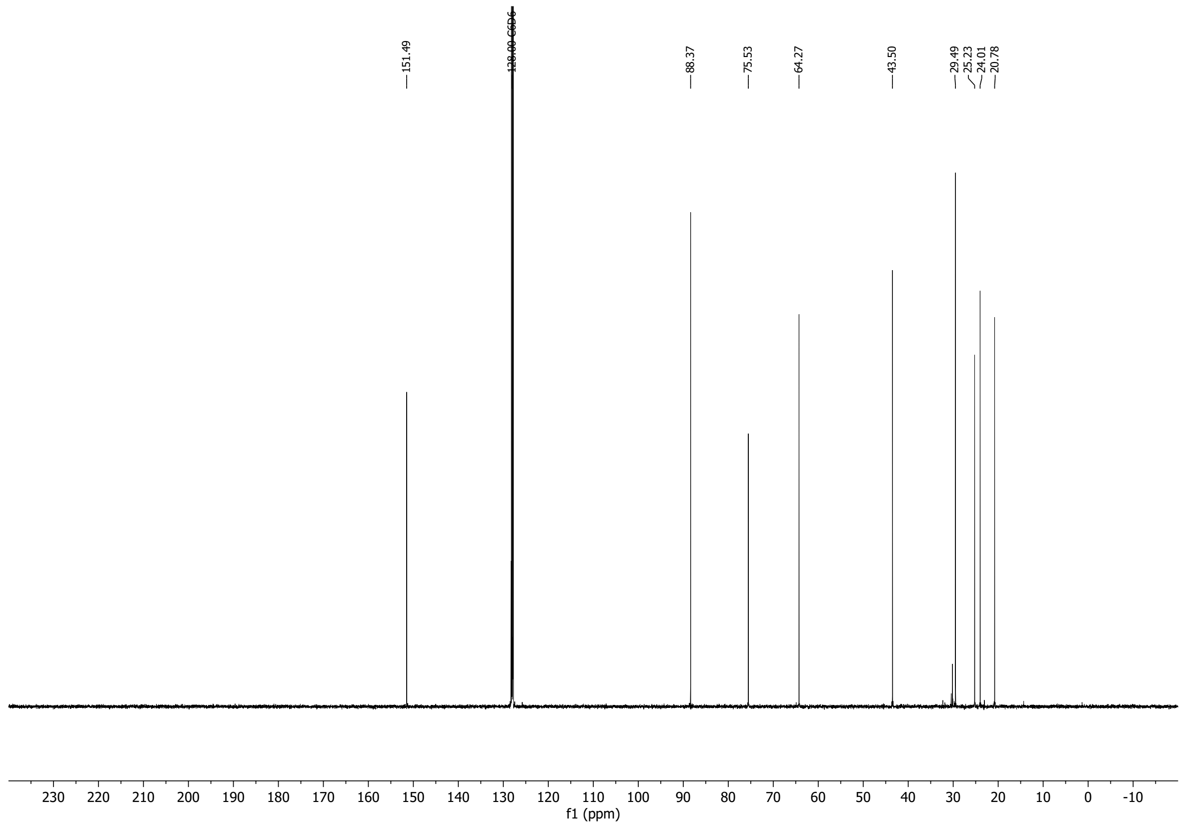

^{13}C NMR (125 MHz, C_6D_6) spectrum of compound **S33**.

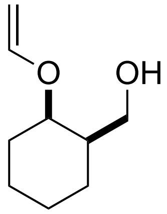

S33

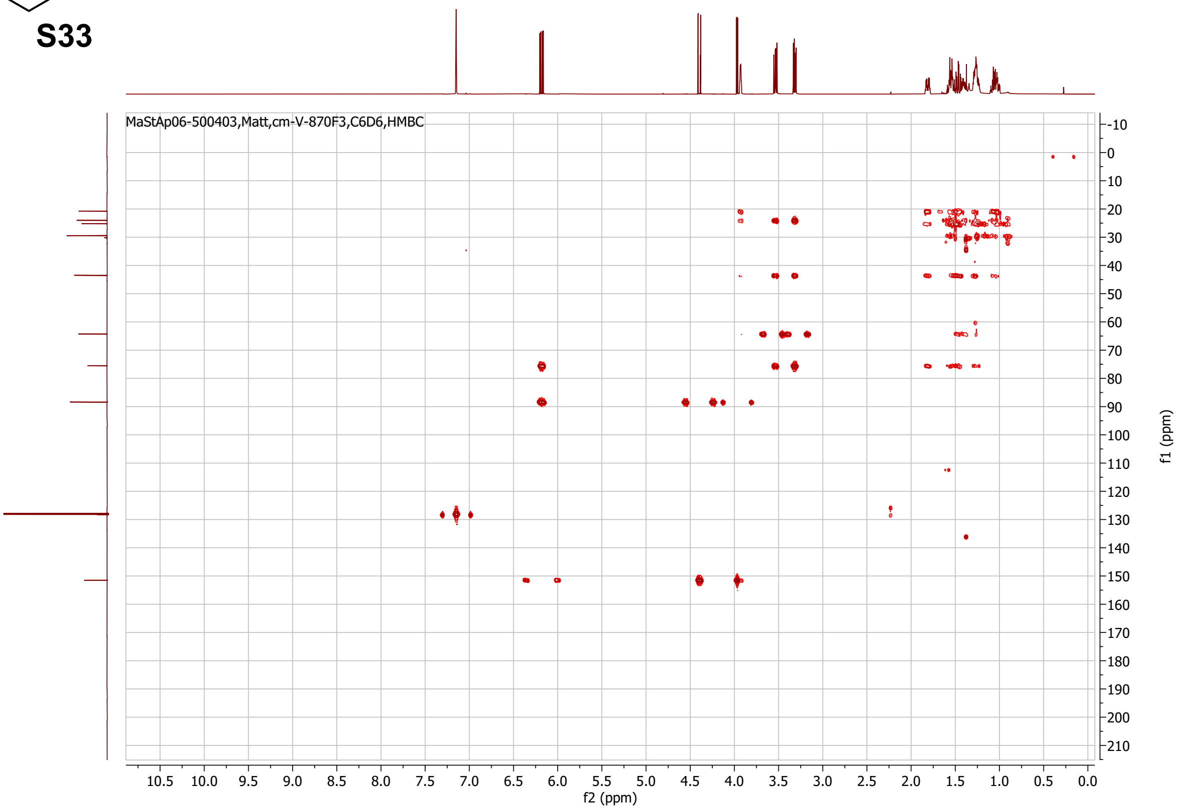

HMBC (coupled) NMR spectrum of compound **S33**.

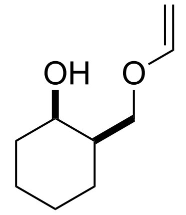

S34

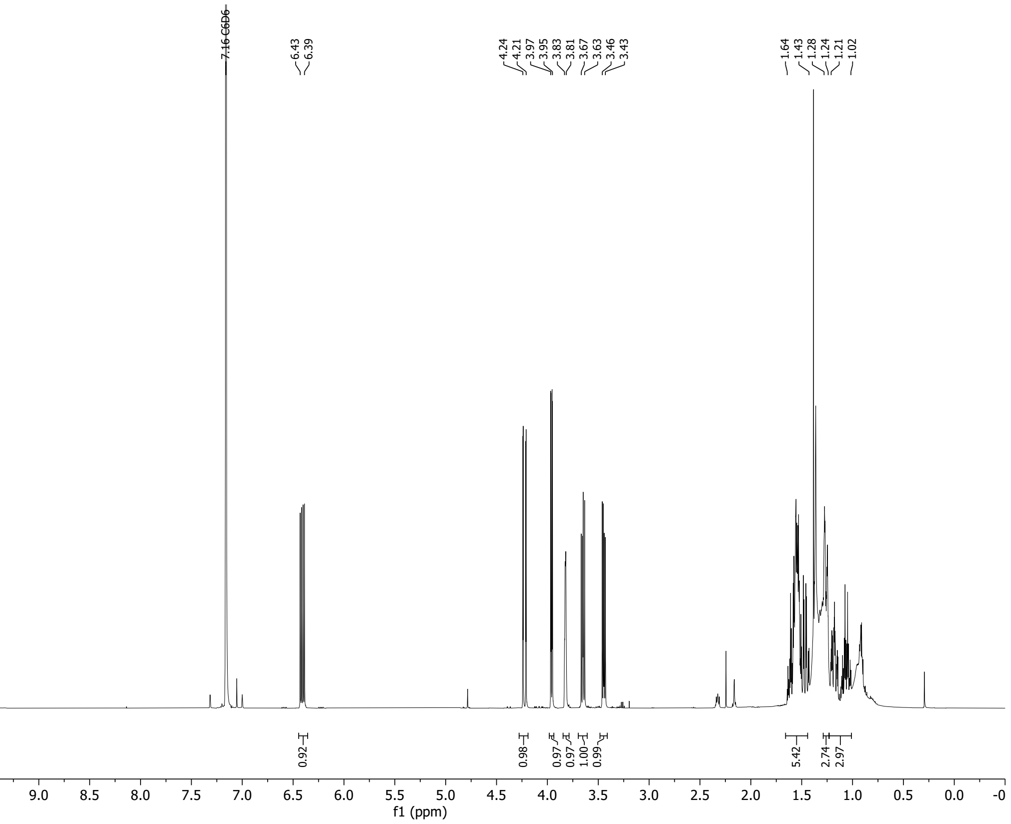

^{1}H NMR (500 MHz, C_6D_6) spectrum of compound **S34**.

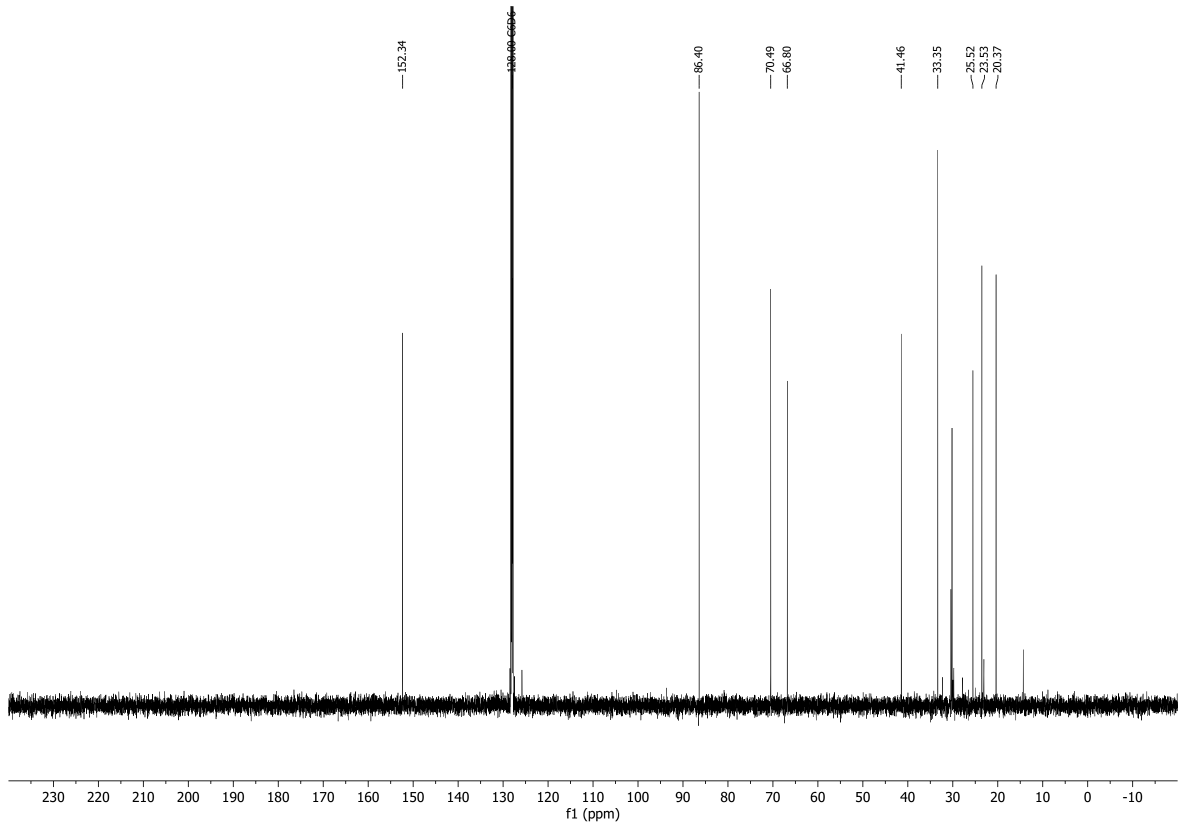

^{13}C NMR (125 MHz, C_6D_6) spectrum of compound **S34**.

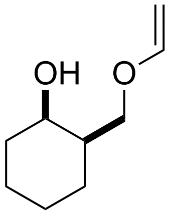

S34

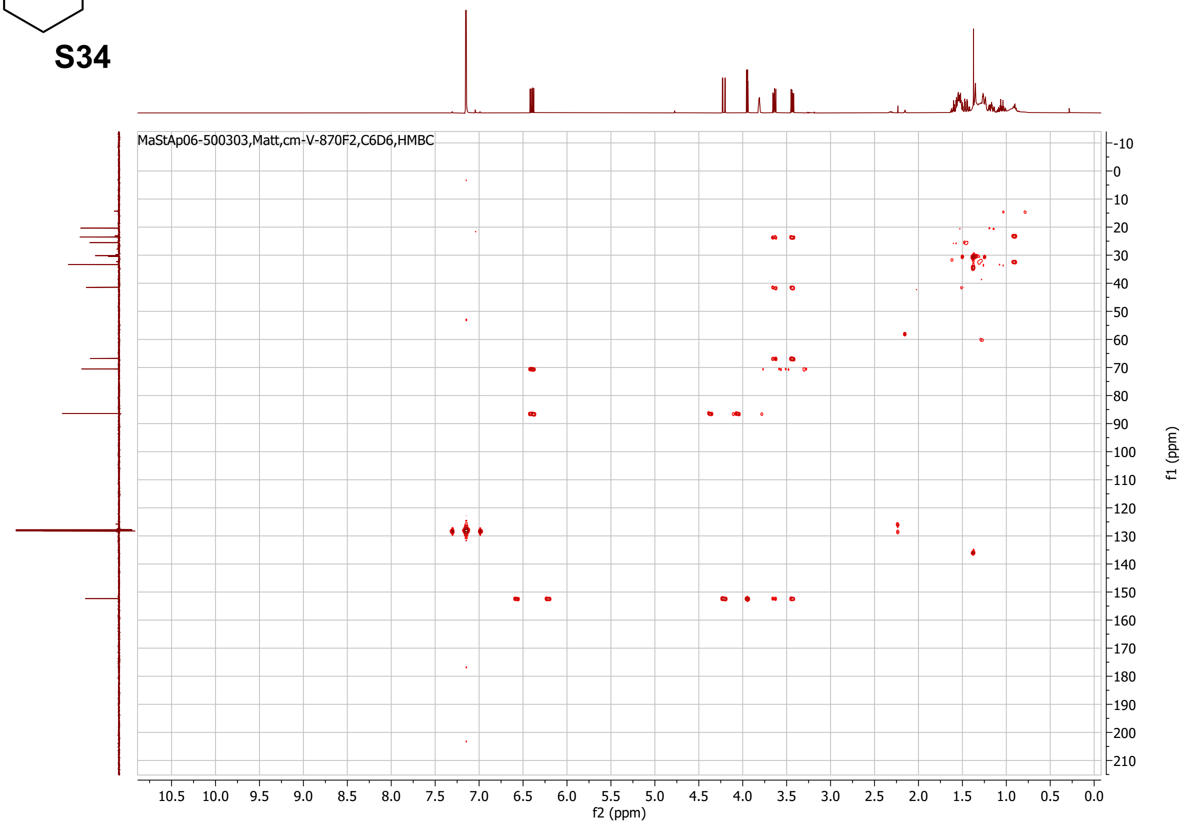

HMBC (coupled) NMR spectrum of compound **S34**.

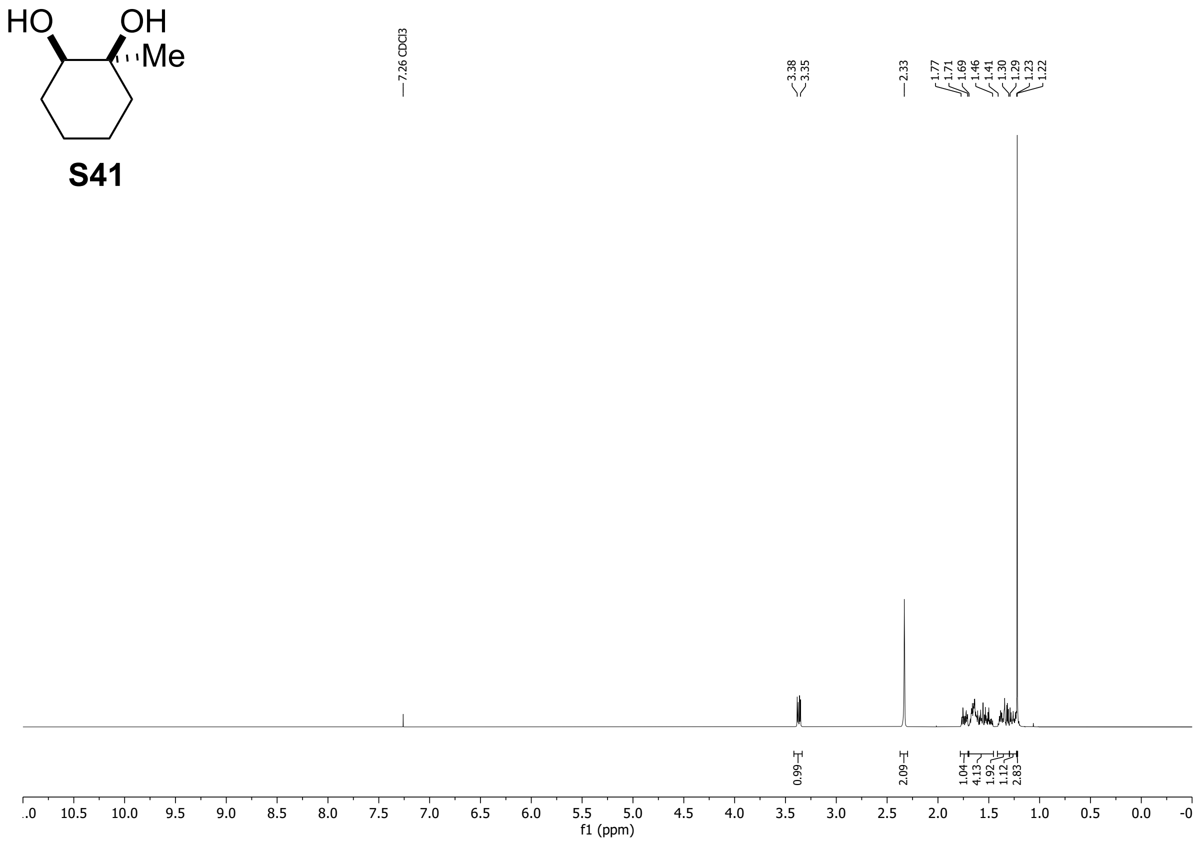

^{1}H NMR (400 MHz, $CDCl_3$) spectrum of compound **S41**.

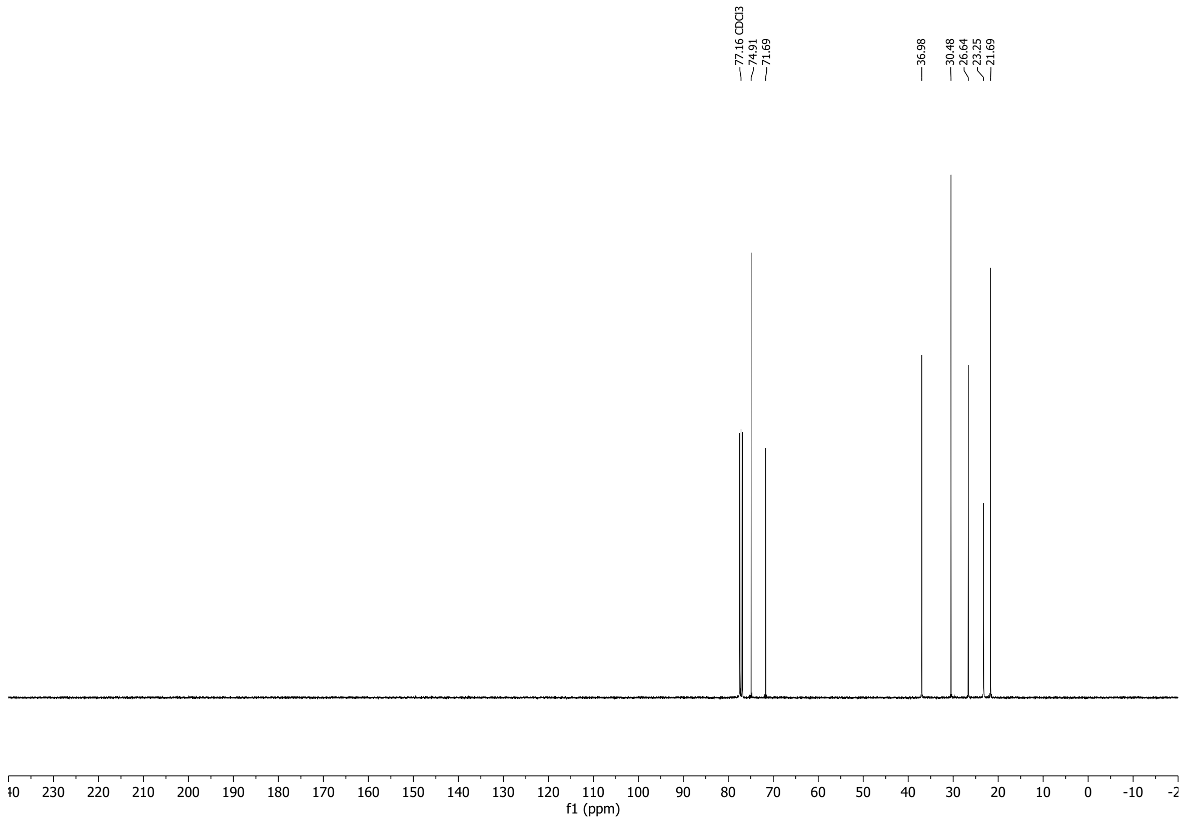

^{13}C NMR (100 MHz, $CDCl_3$) spectrum of compound **S41**.

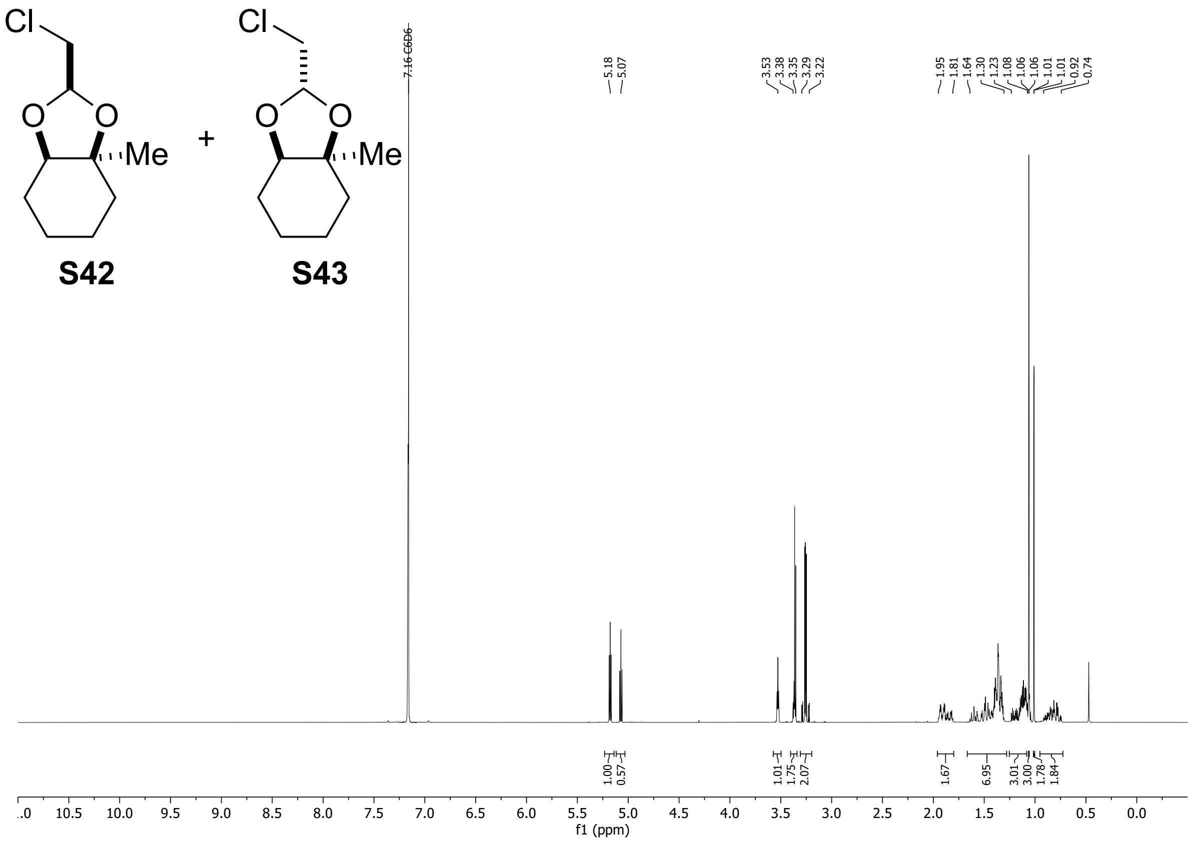

^1H NMR (400 MHz, C_6D_6) spectrum of diastereomers **S42** and **S43**.

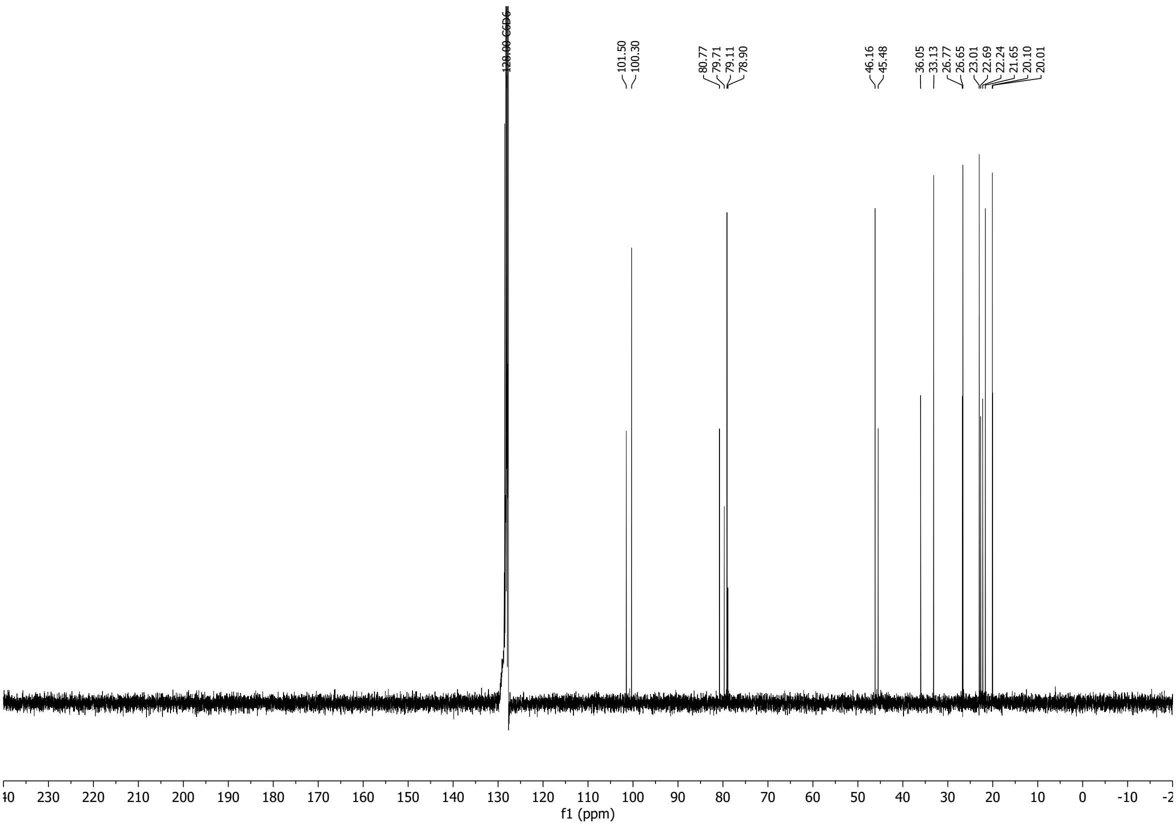

^{13}C NMR (100 MHz, C_6D_6) spectrum of spectrum of diastereomers **S42** and **S43**.

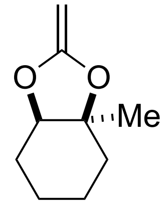

S35

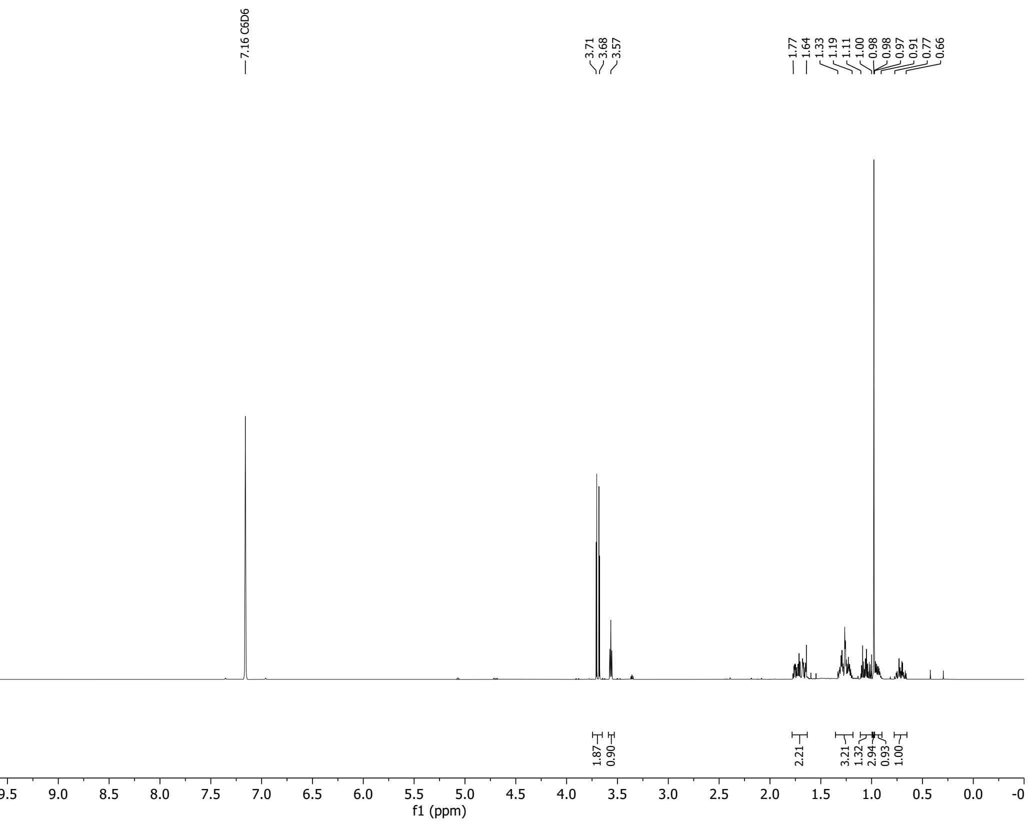

^{1}H NMR (400 MHz, C_6D_6) spectrum of compound **S35**.

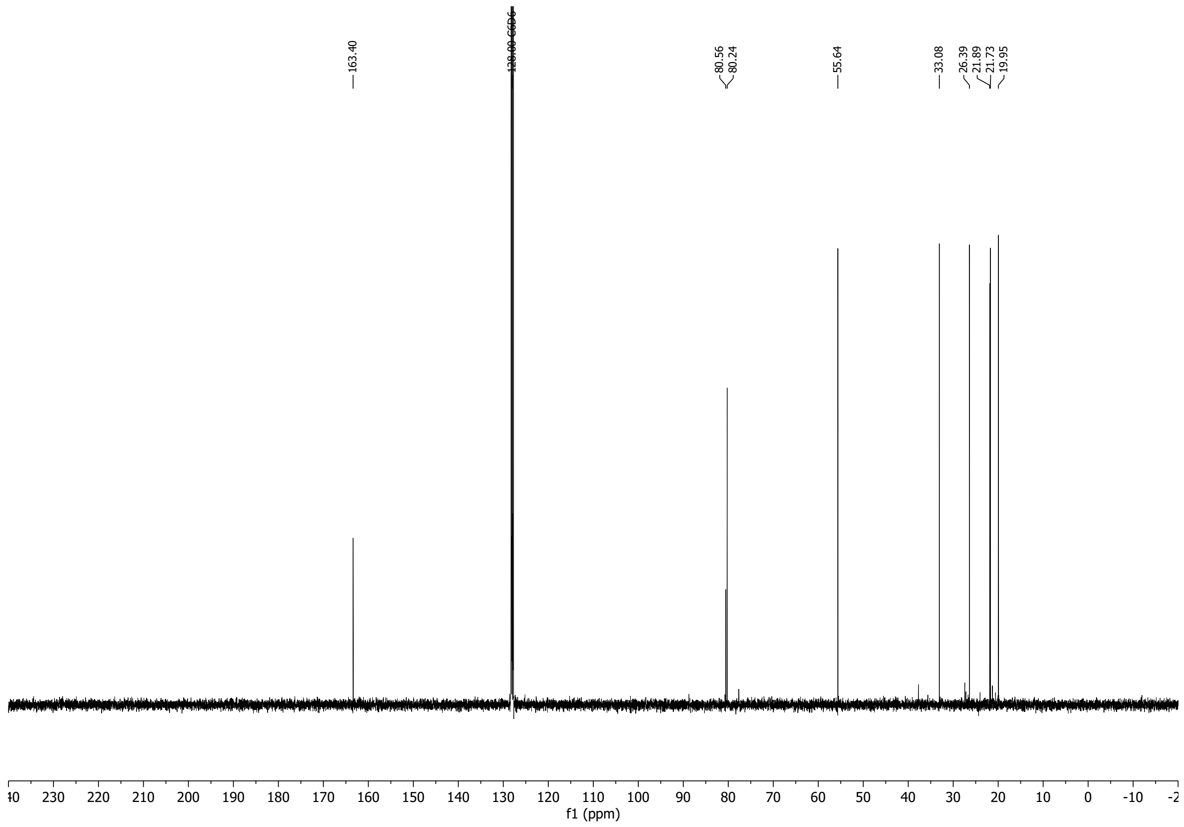

^{13}C NMR (100 MHz, C_6D_6) spectrum of compound **S35**.

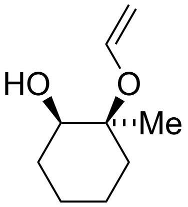

S36

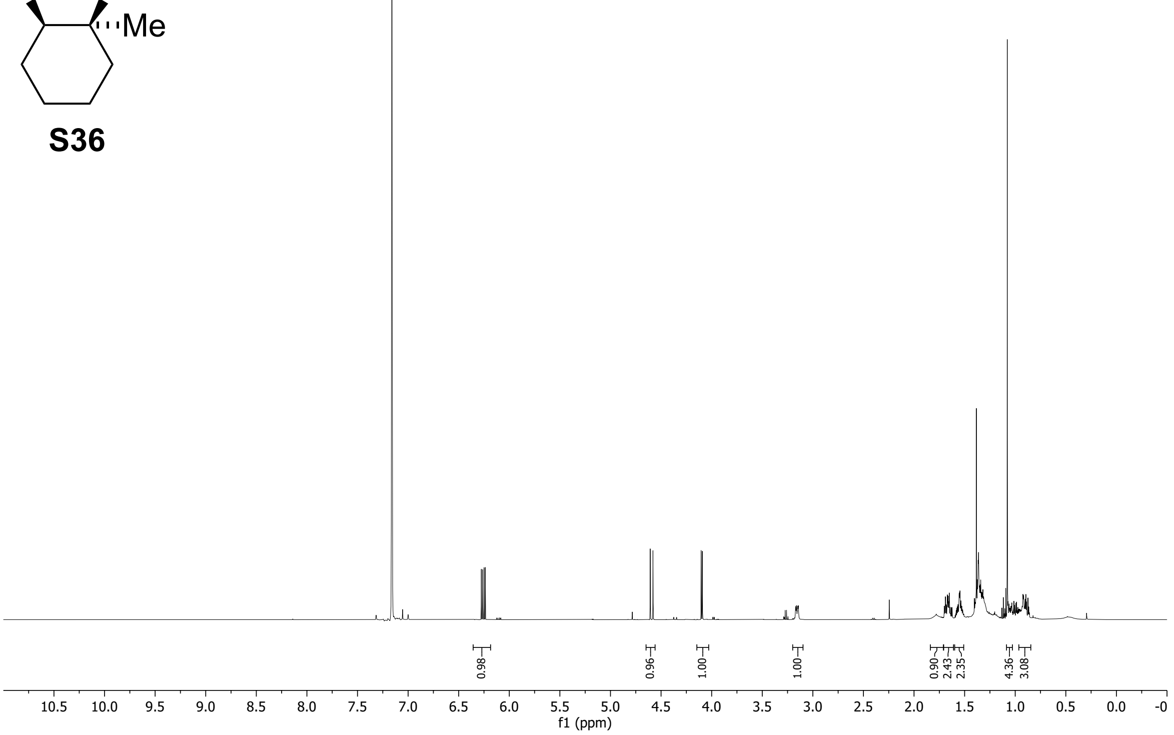

^{1}H NMR (500 MHz, C_6D_6) spectrum of compound **S36**.

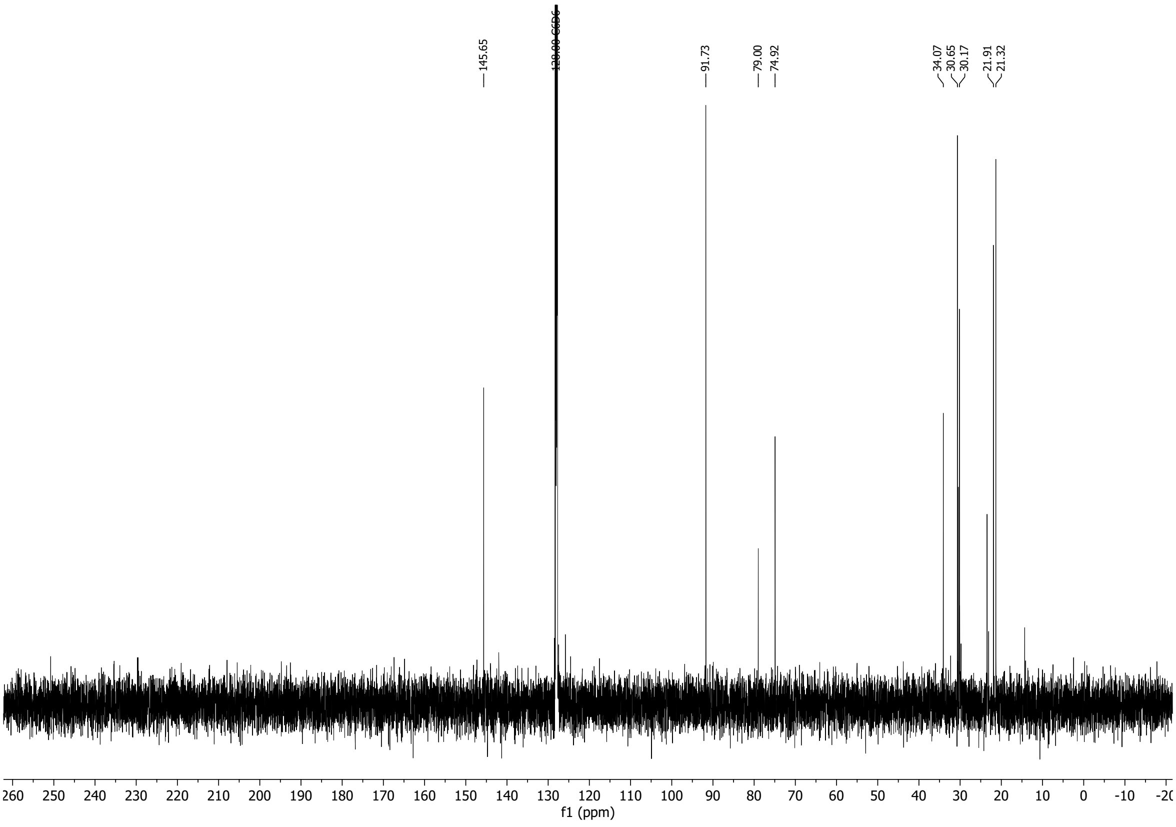

^{13}C NMR (125 MHz, C_6D_6) spectrum of compound **S36**.

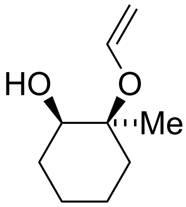

S36

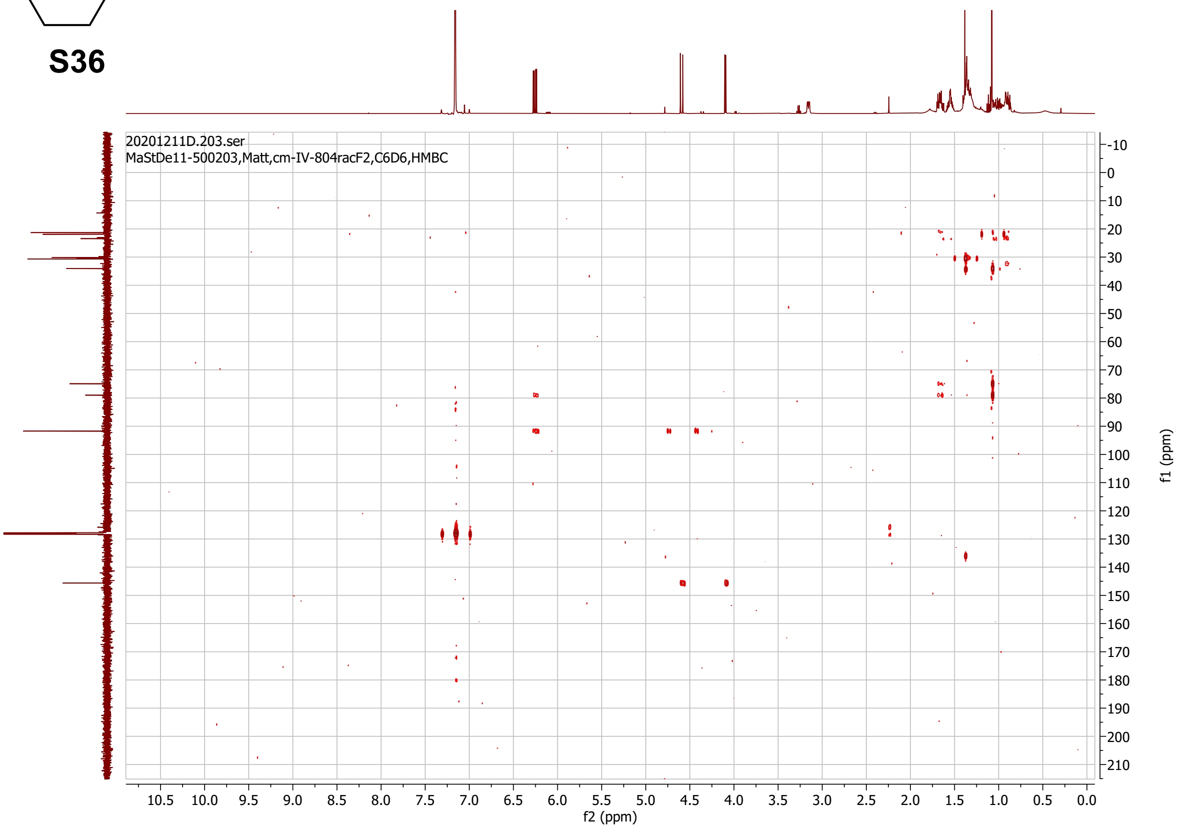

HMBC (coupled) NMR spectrum of compound **S36**.

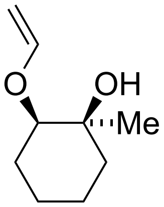

S37

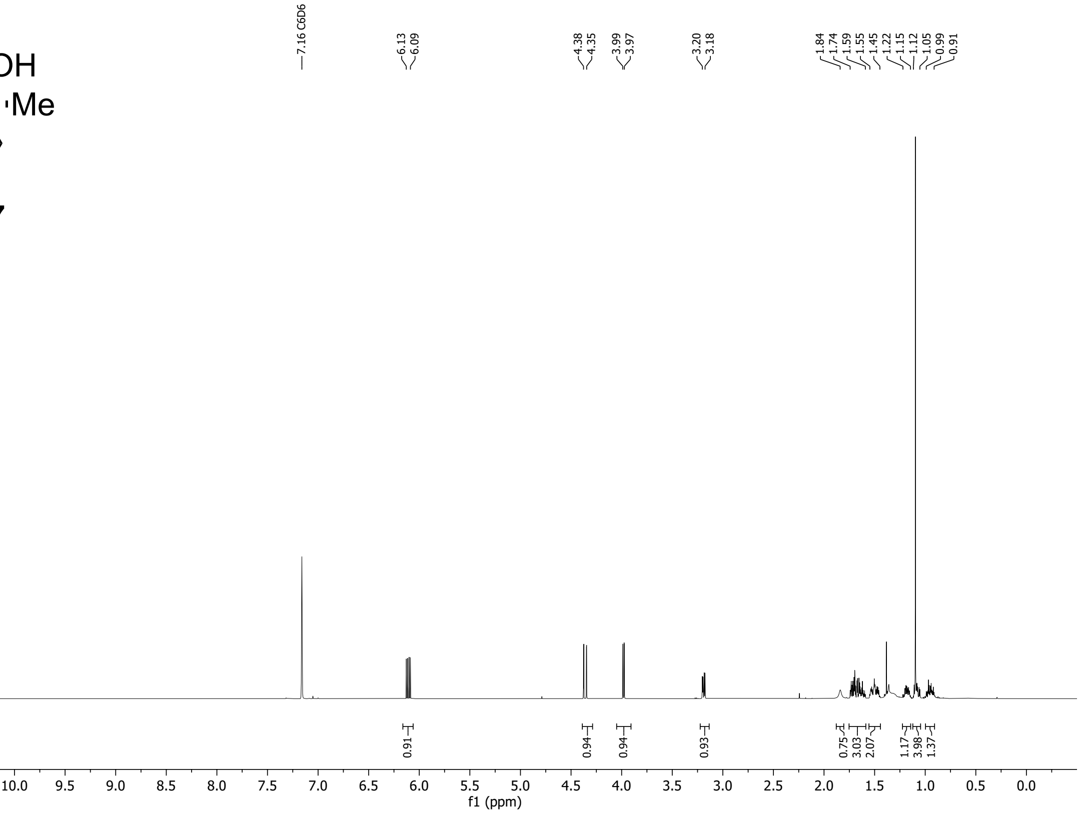

^{1}H NMR (500 MHz, C_6D_6) spectrum of compound **S37**.

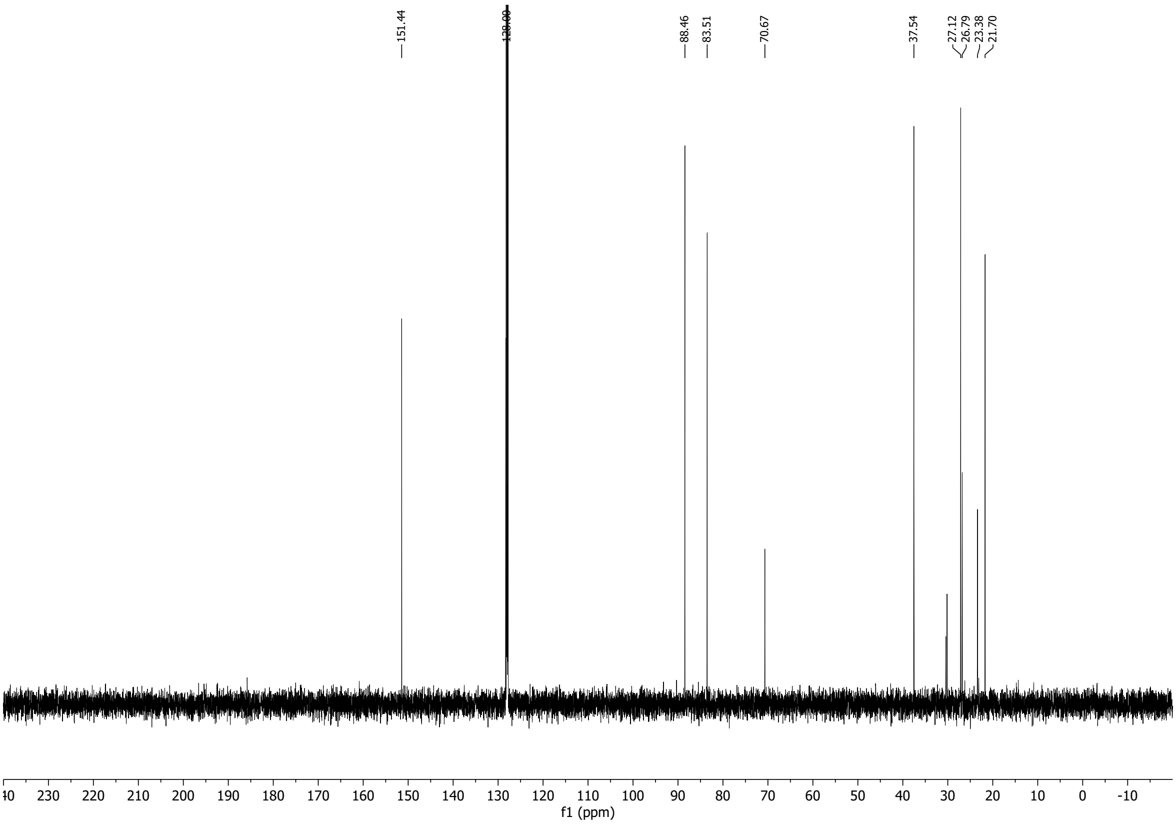

^{13}C NMR (125 MHz, C_6D_6) spectrum of compound **S37**.

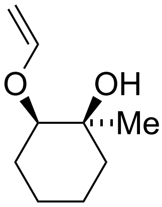

S37

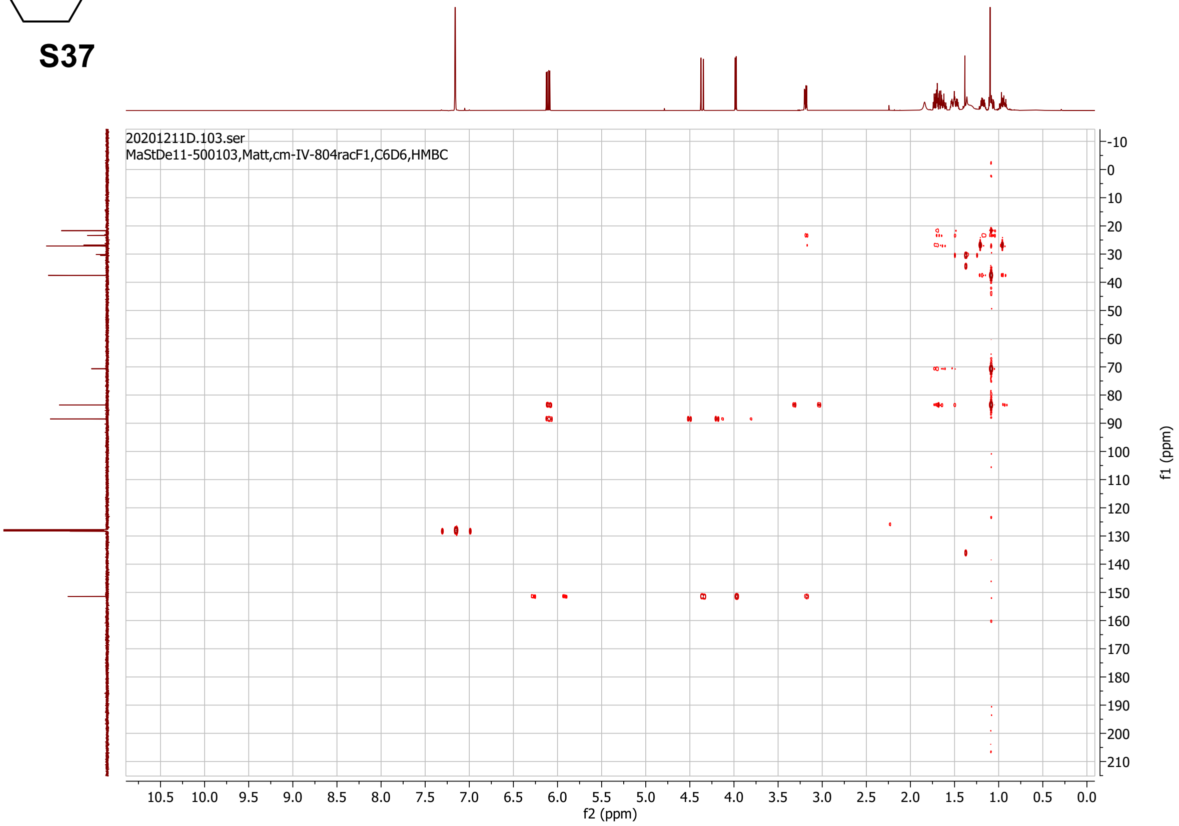

HMBC (coupled) NMR spectrum of compound **S37**.

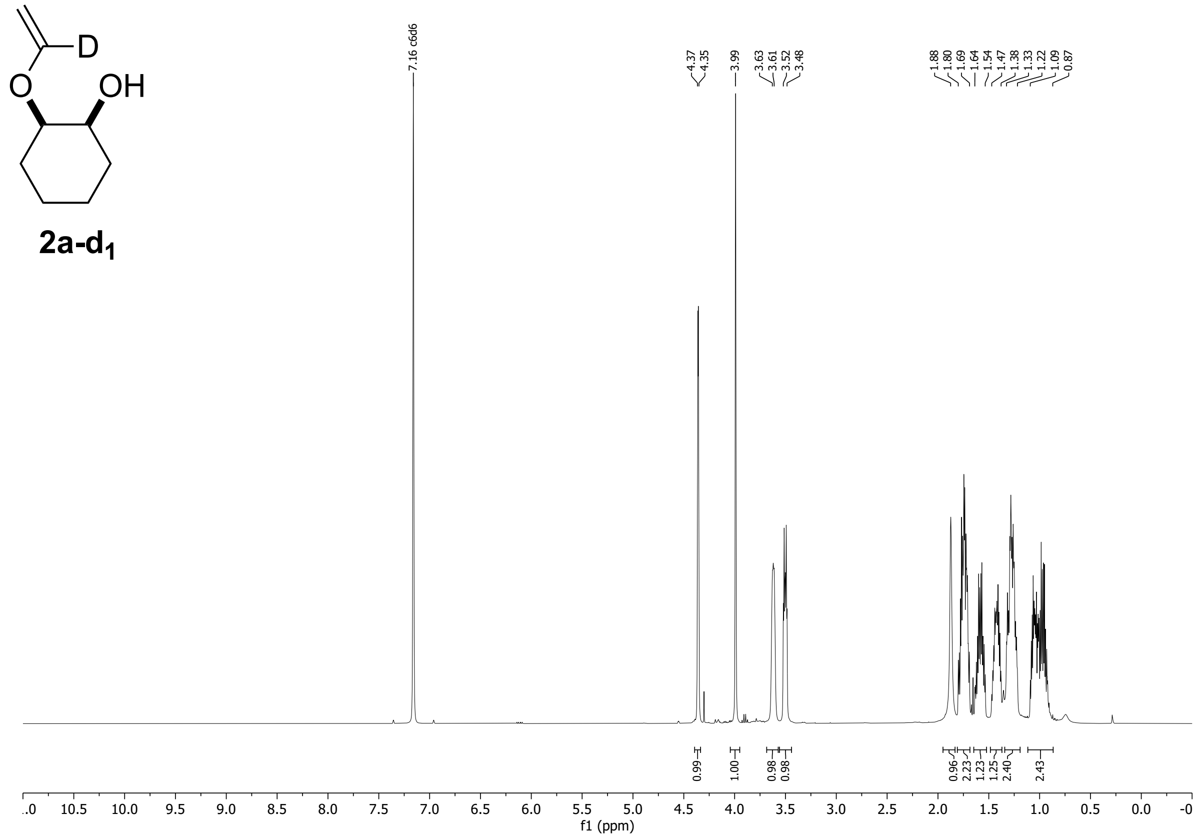

^{1}H NMR (400 MHz, C_6D_6) spectrum of compound **2a-d$_1$**.

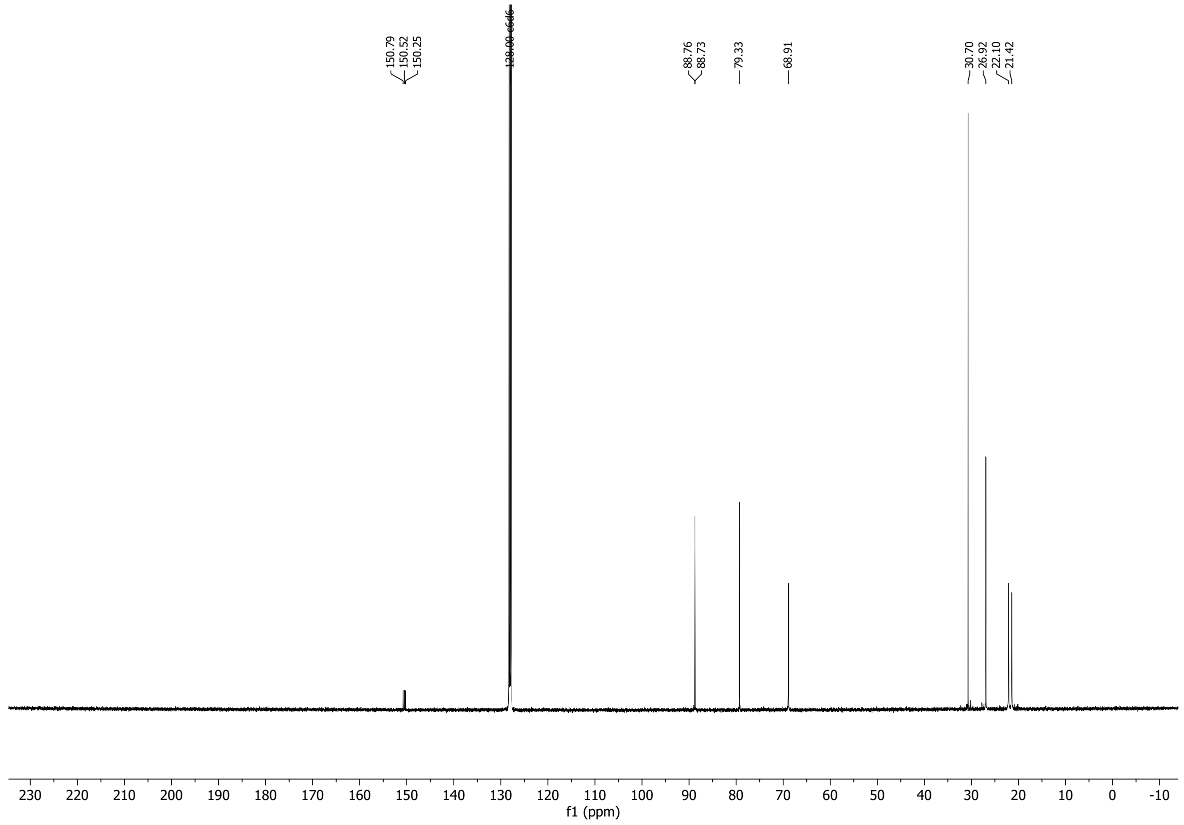

^{13}C NMR (100 MHz, C_6D_6) spectrum of compound **2a-d$_1$**.

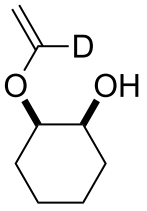

2a-d$_1$

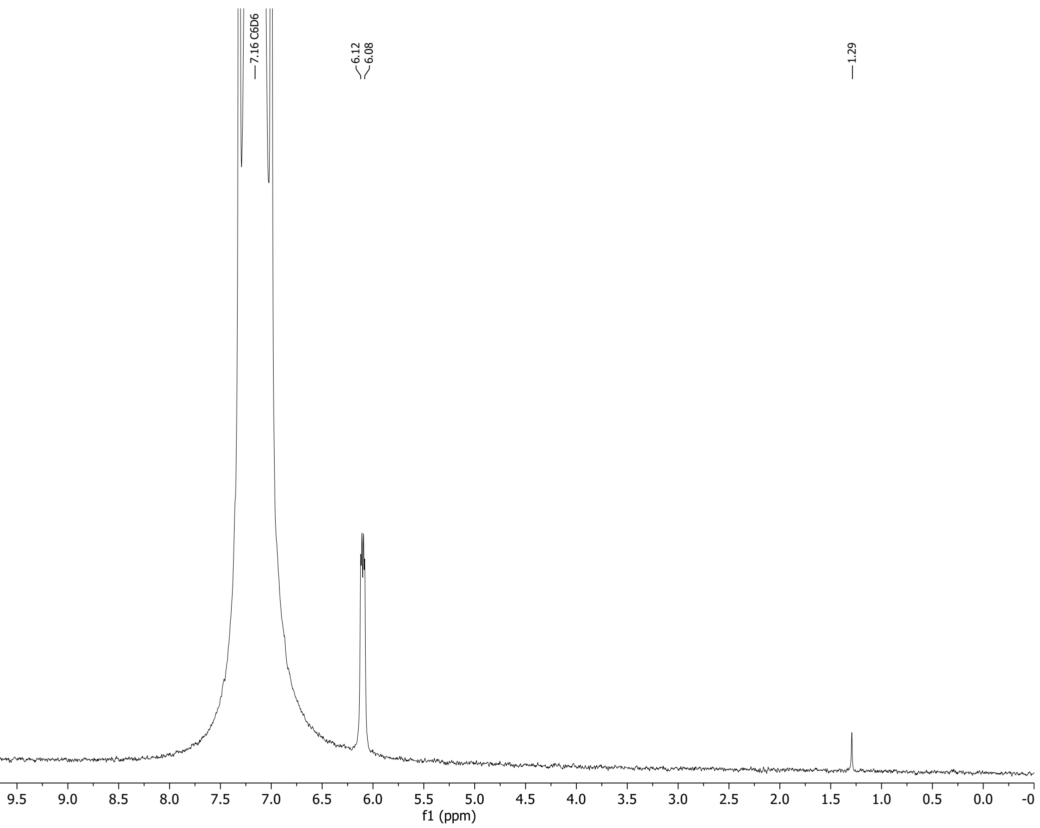

^{2}H NMR (77 MHz, C_6D_6) spectrum of compound **2a-d$_1$**.
Note: A small amount of OH→OD exchange had occurred (1.29 ppm).

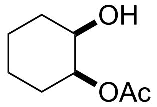

S44

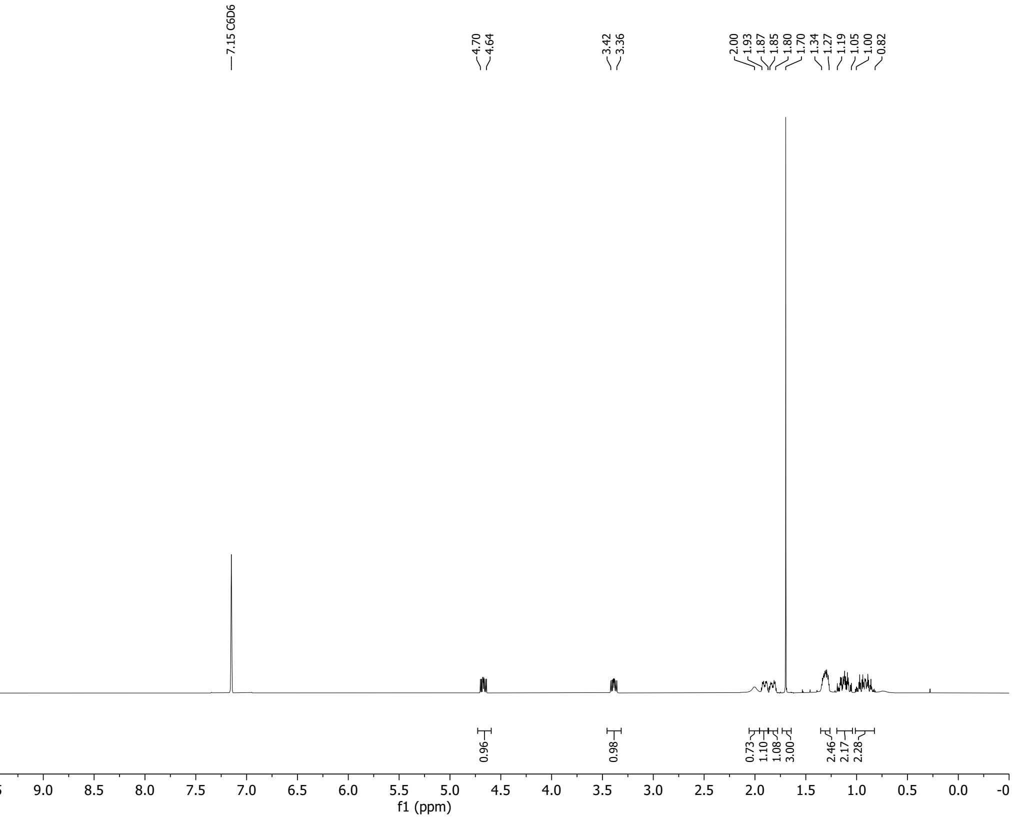

^{1}H NMR (400 MHz, C_6D_6) spectrum of compound **S44**.

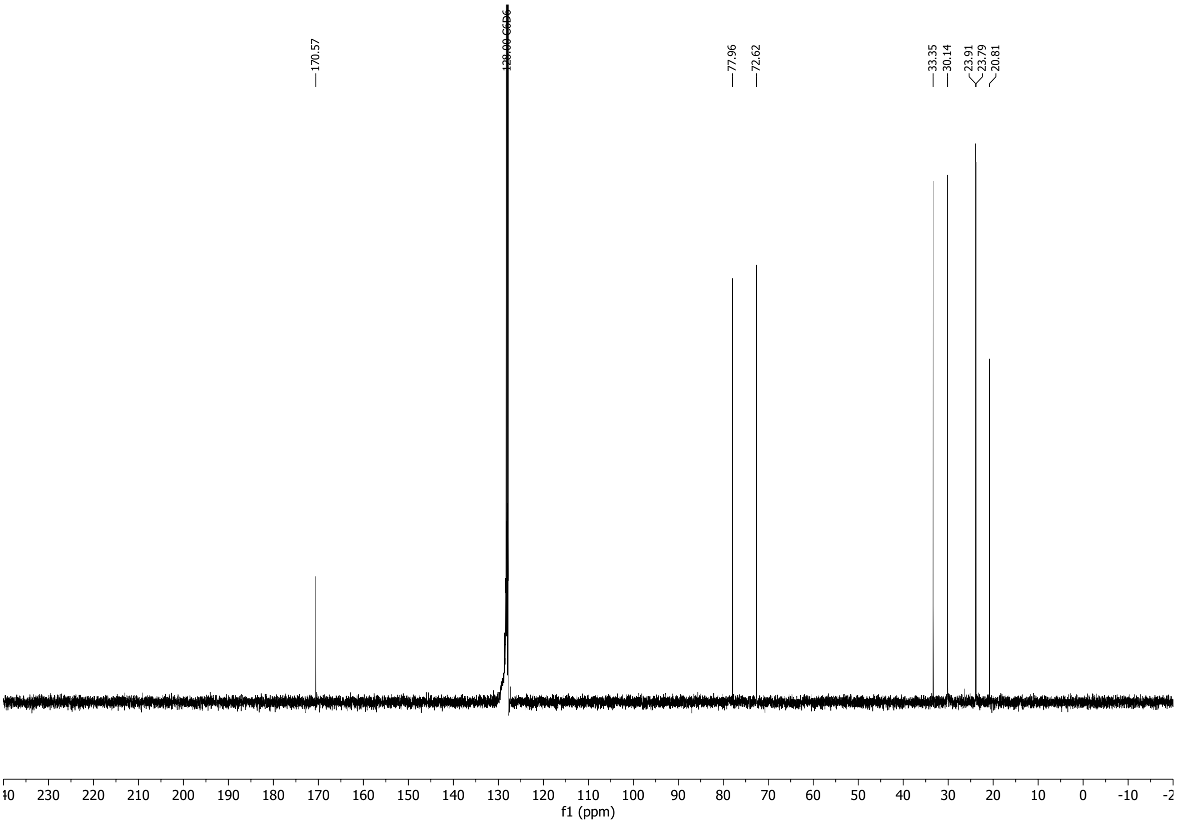

^{13}C NMR (100 MHz, C_6D_6) spectrum of compound **S44**.

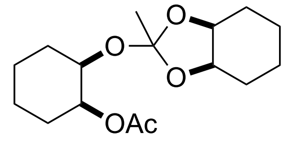

S45

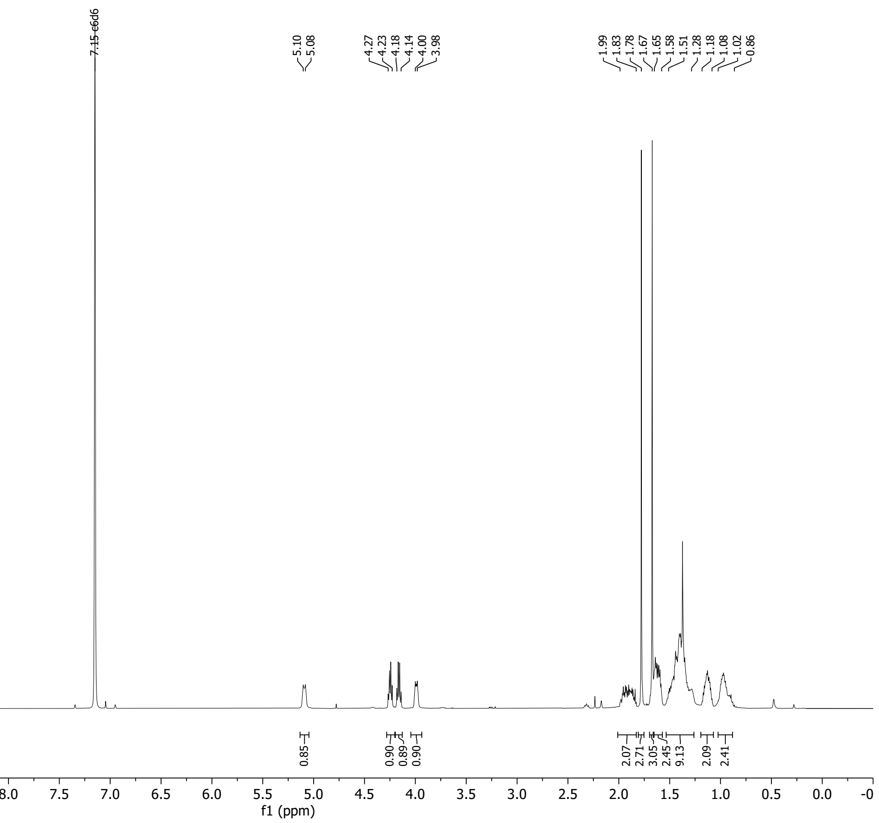

^{1}H NMR (400 MHz, C_6D_6) spectrum of compound **S45**.

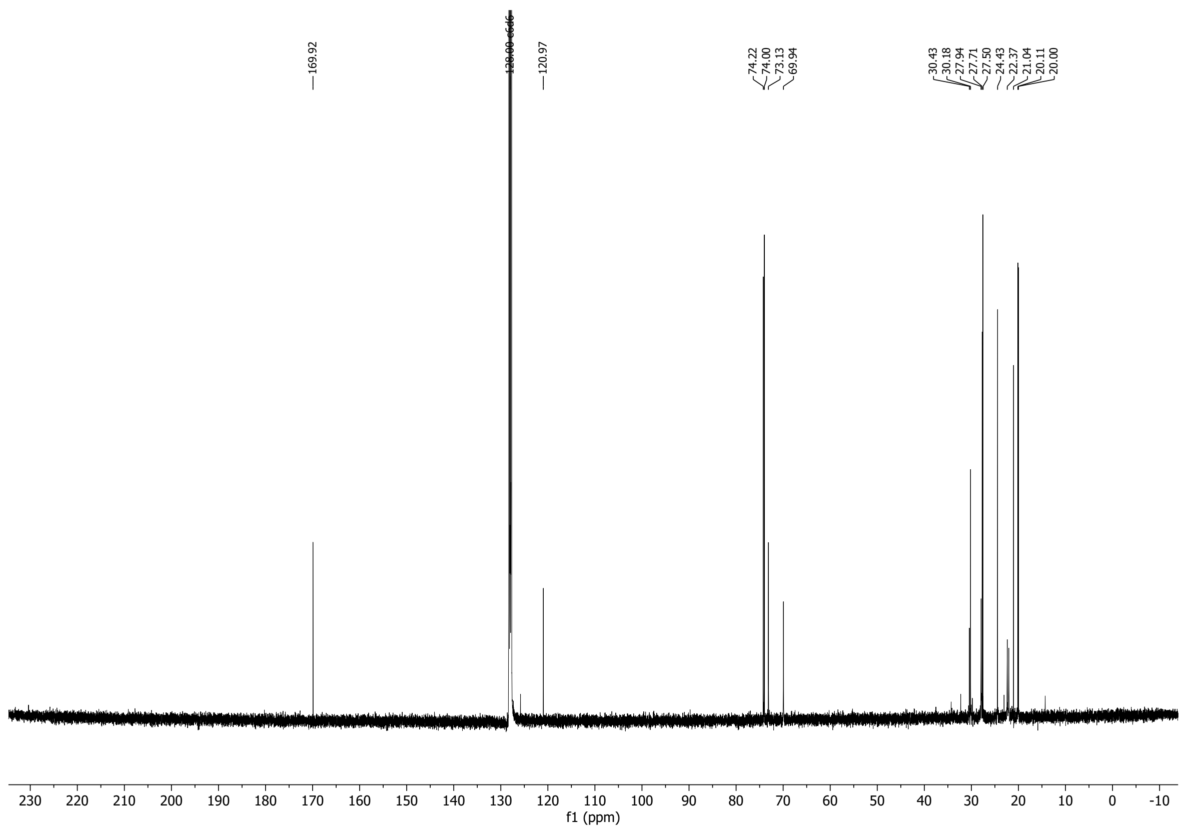

^{13}C NMR (100 MHz, C_6D_6) spectrum of compound **S45**.

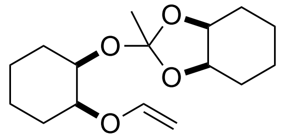

S46

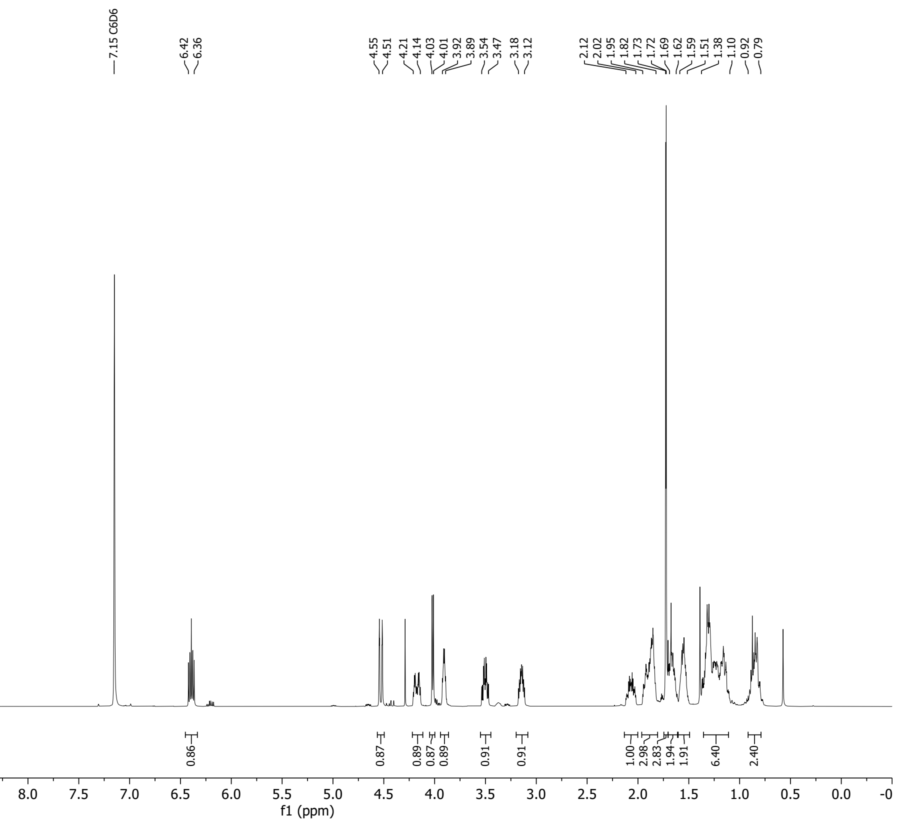

^{1}H NMR (500 MHz, C_6D_6) spectrum of compound **S46**.

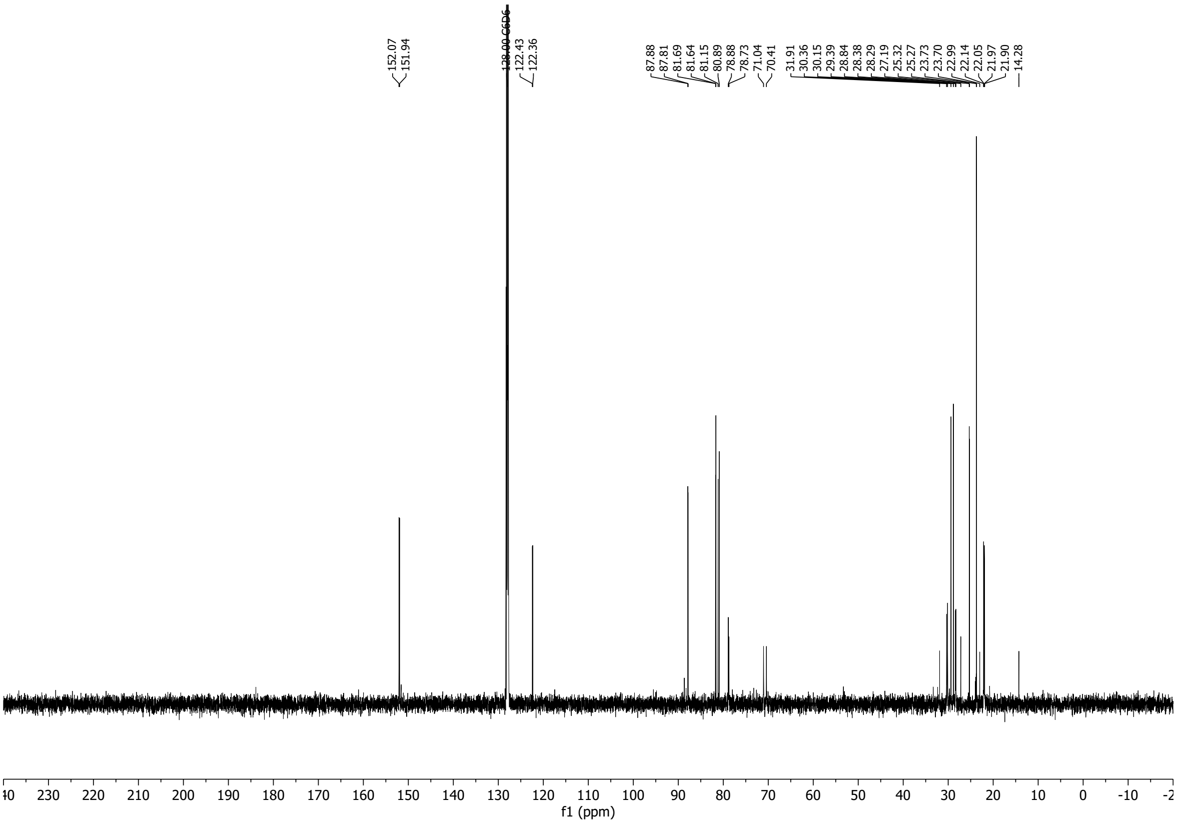

^{13}C NMR (125 MHz, C_6D_6) spectrum of compound **S46**.

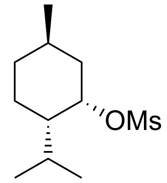

S47

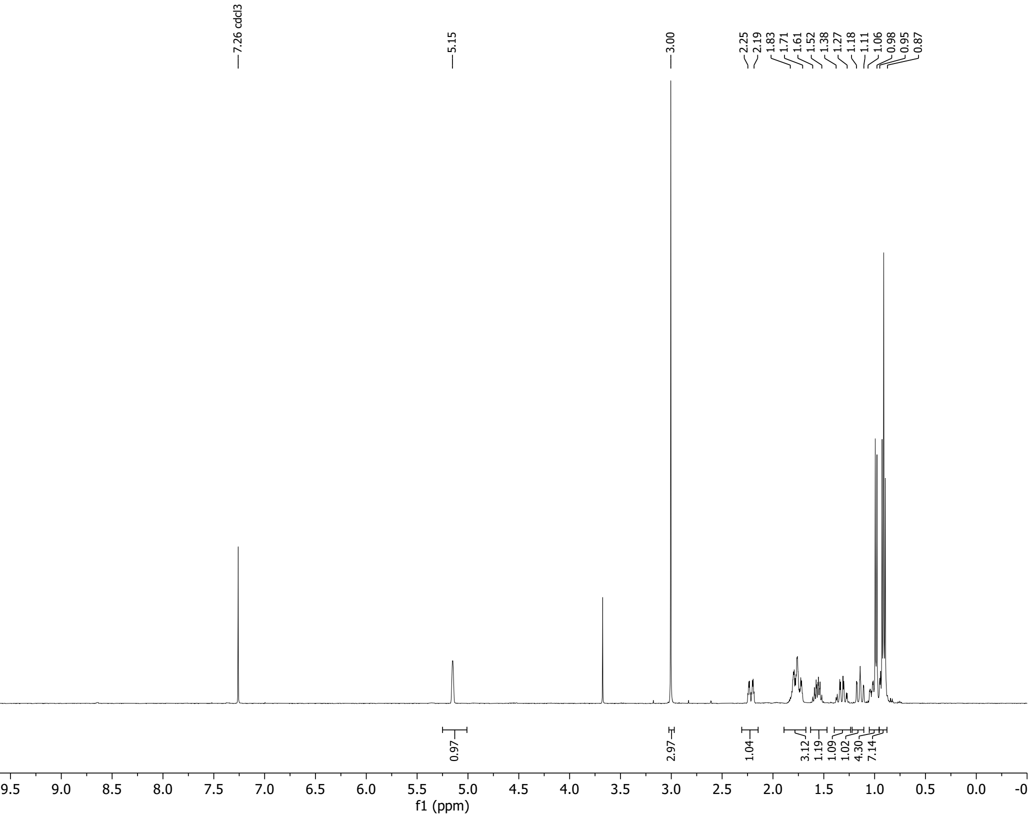

^{1}H NMR (400 MHz, $CDCl_3$) spectrum of compound **S47**.

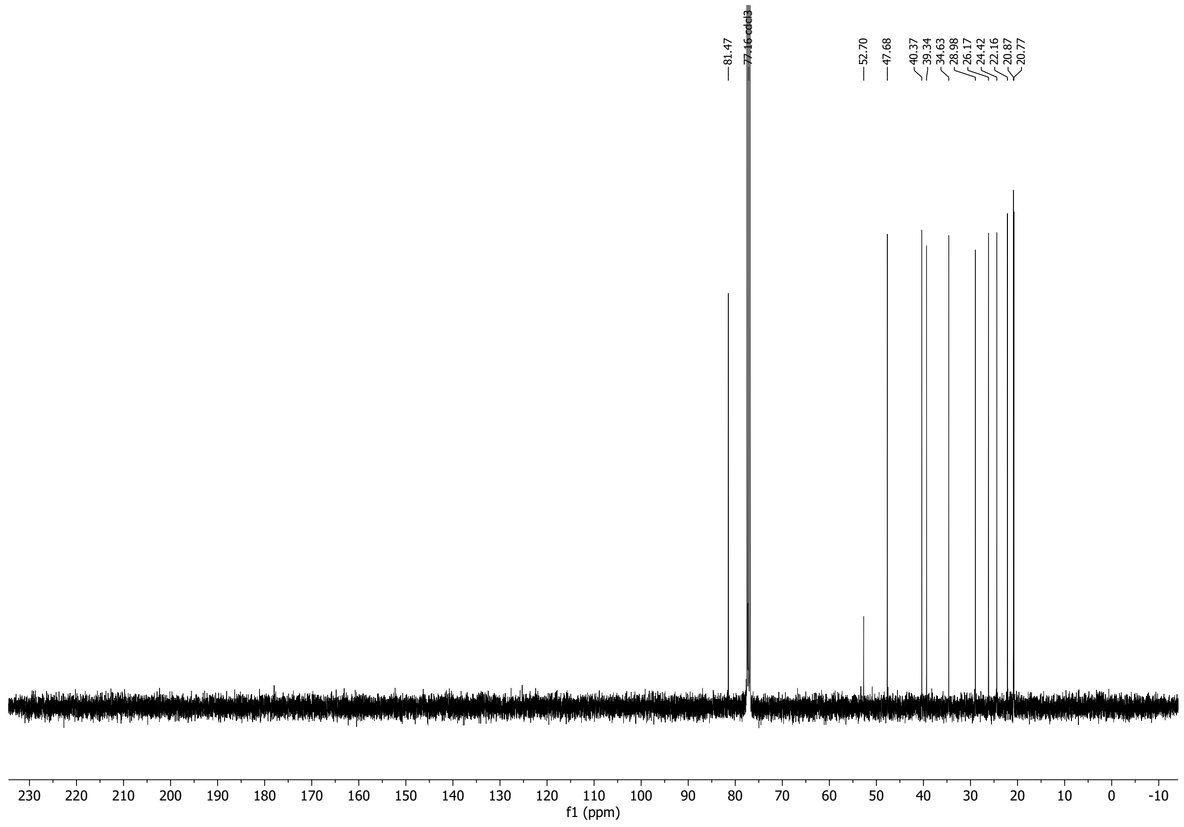

^{13}C NMR (100 MHz, $CDCl_3$) spectrum of compound **S47**.

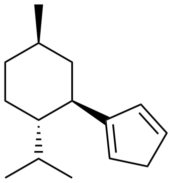

S48

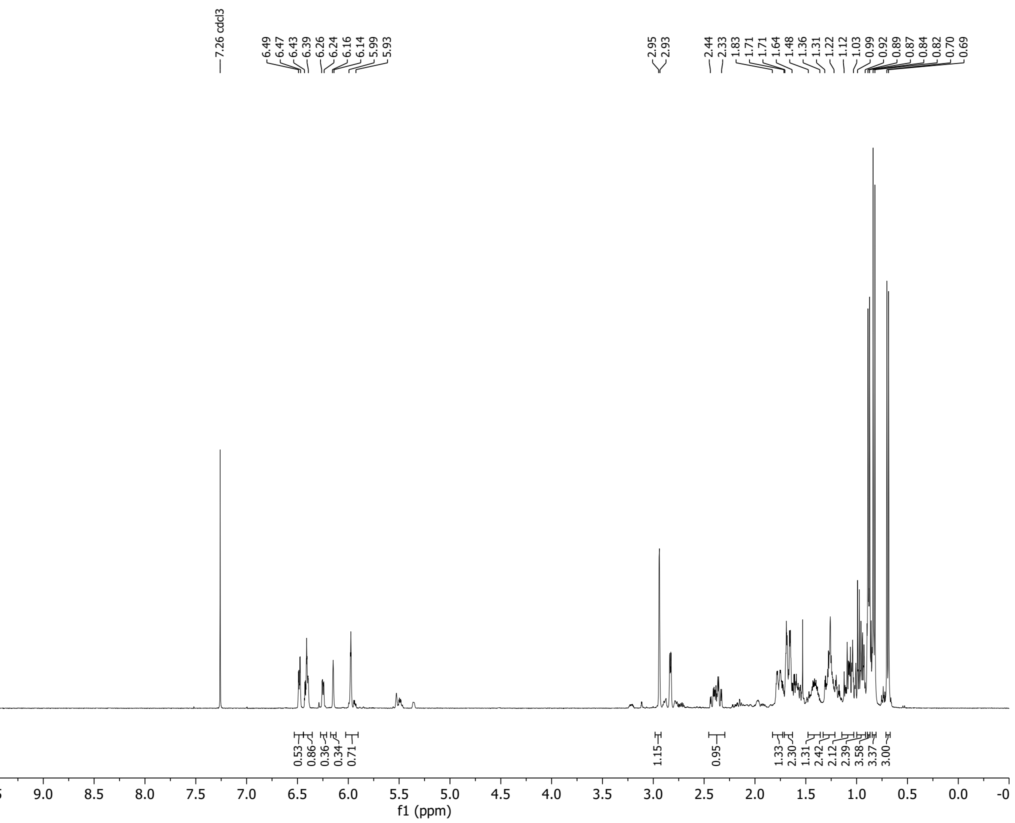

^{1}H NMR (400 MHz, $CDCl_3$) spectrum of compound **S48**.

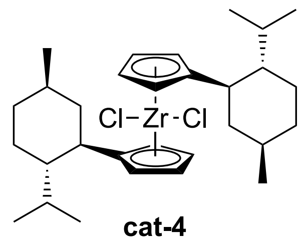

cat-4

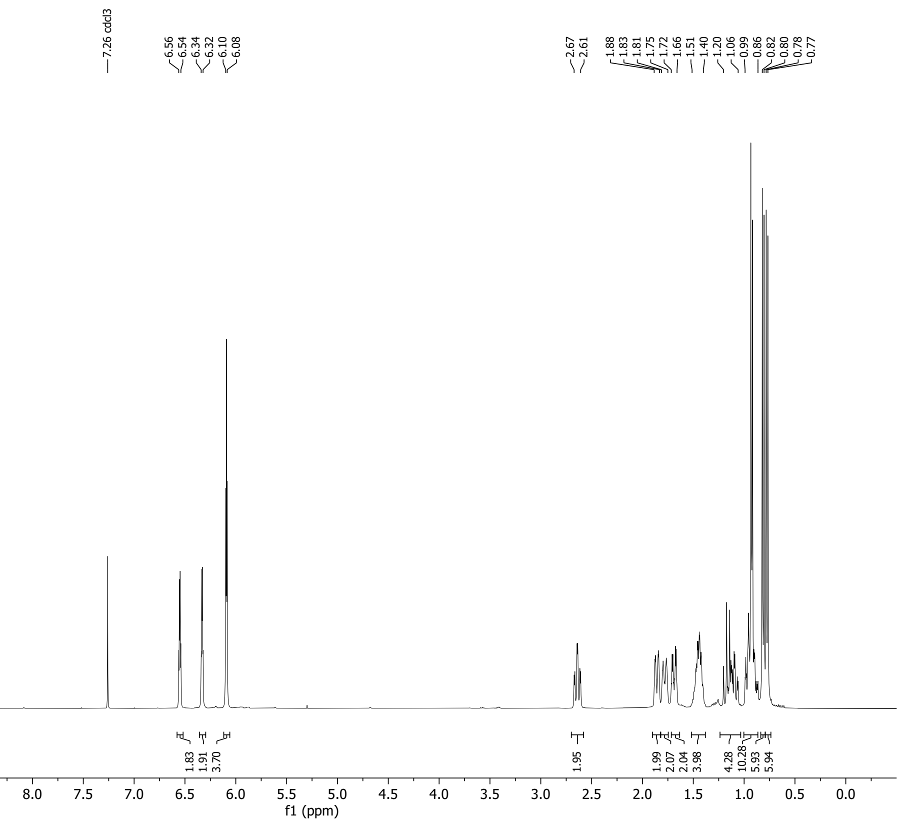

^{1}H NMR (400 MHz, $CDCl_3$) spectrum of **cat-4**.

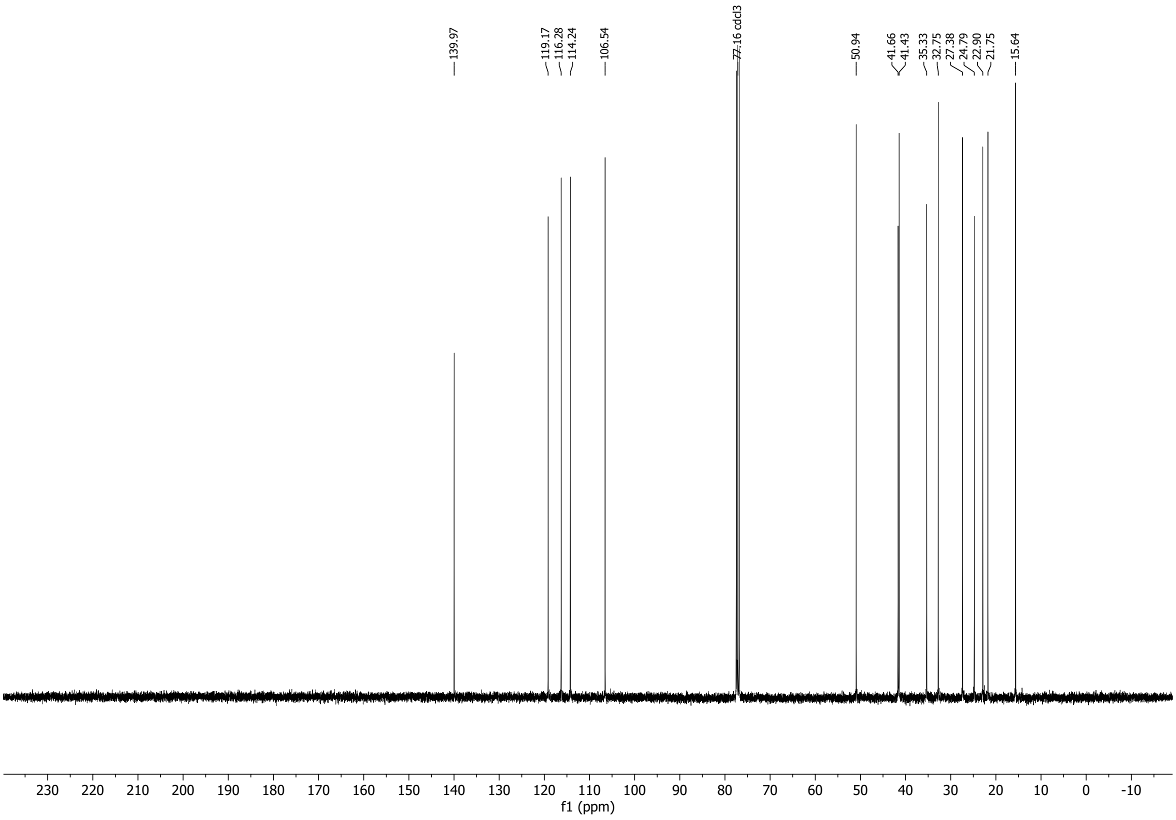

^{13}C NMR (100 MHz, $CDCl_3$) spectrum of **cat-4**.

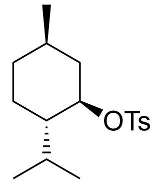

S49

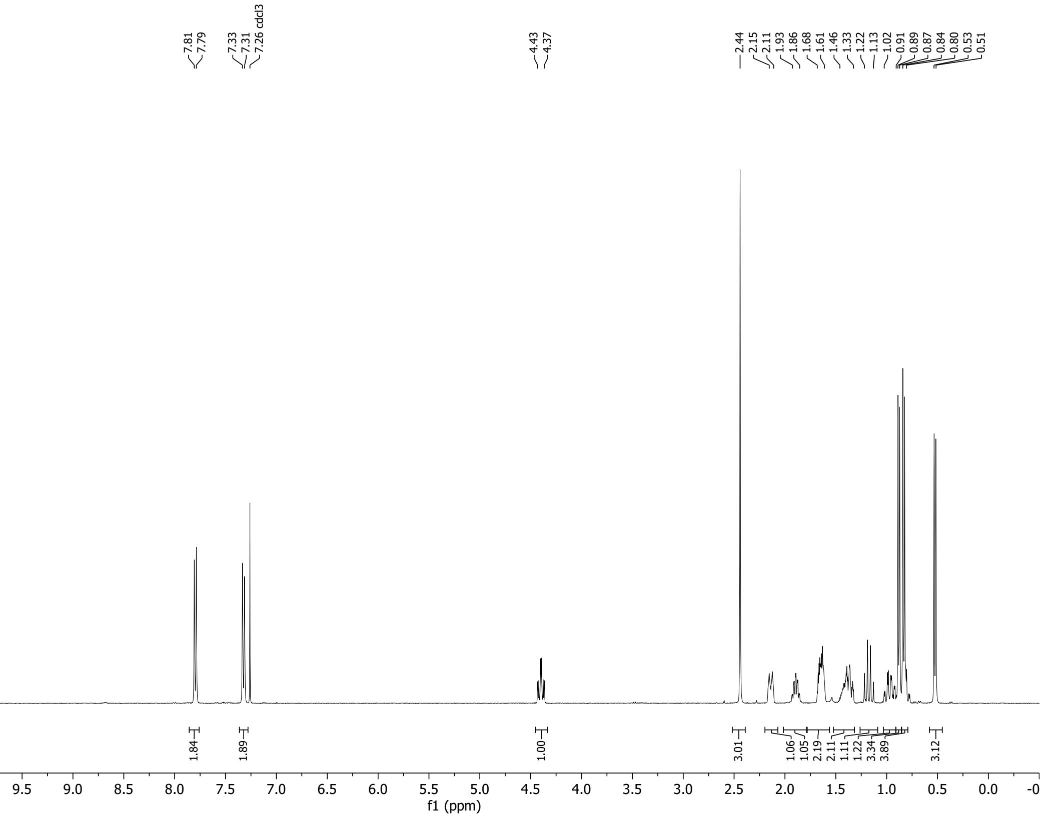

^{1}H NMR (400 MHz, $CDCl_3$) spectrum of compound **S49**.

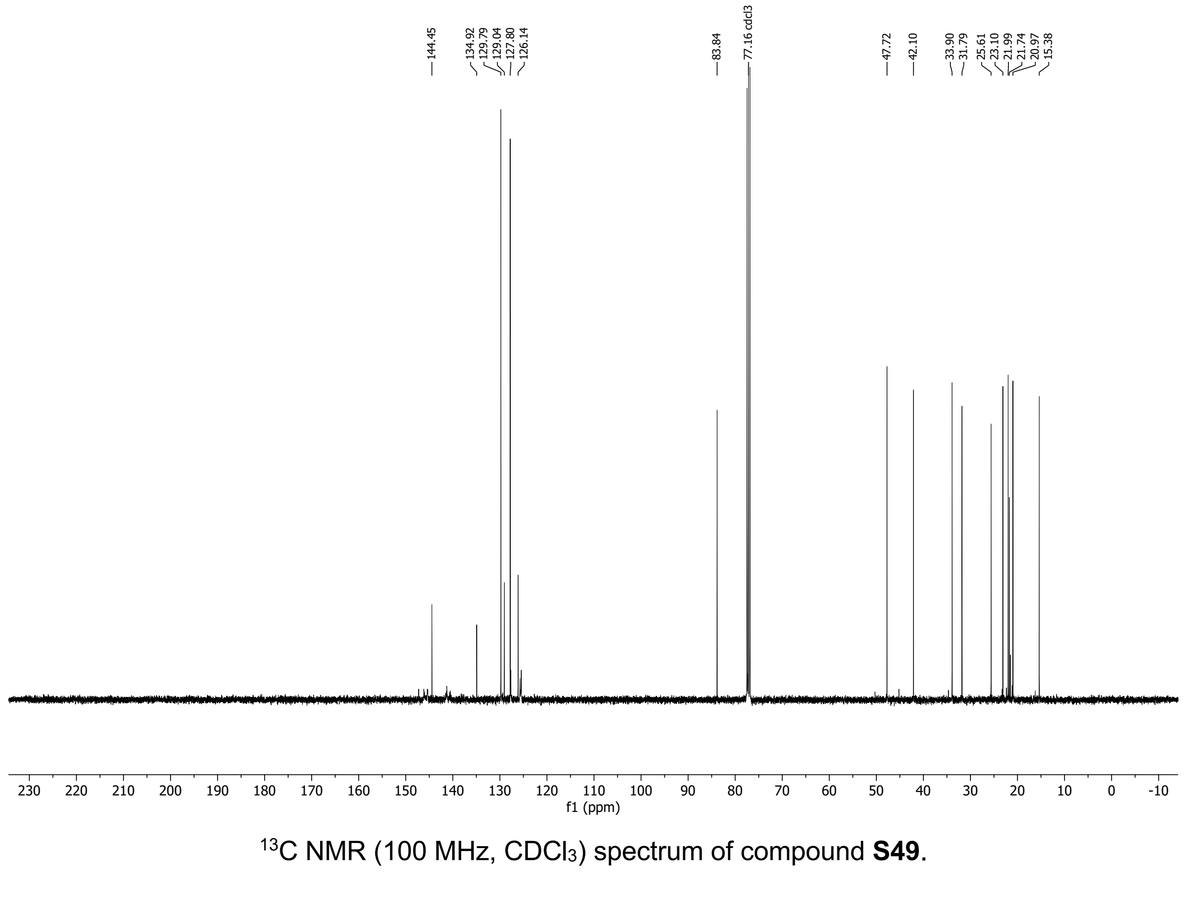

^{13}C NMR (100 MHz, $CDCl_3$) spectrum of compound **S49**.

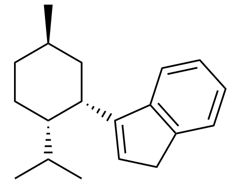

S50

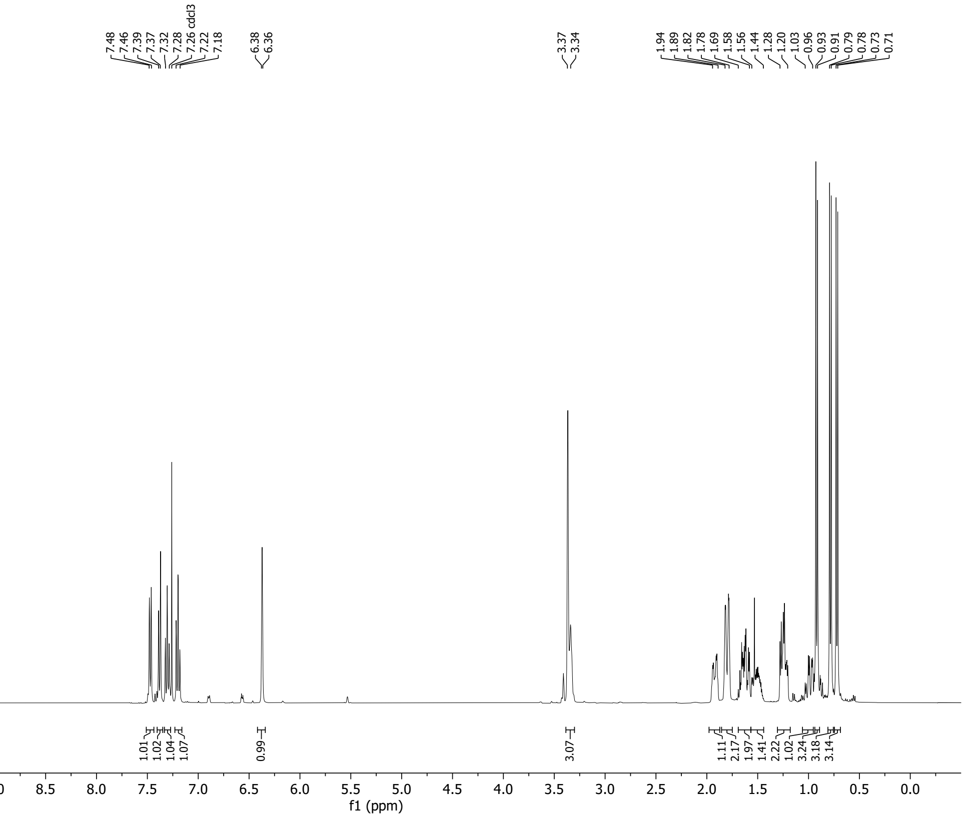

^{1}H NMR (400 MHz, $CDCl_3$) spectrum of compound **S50**.

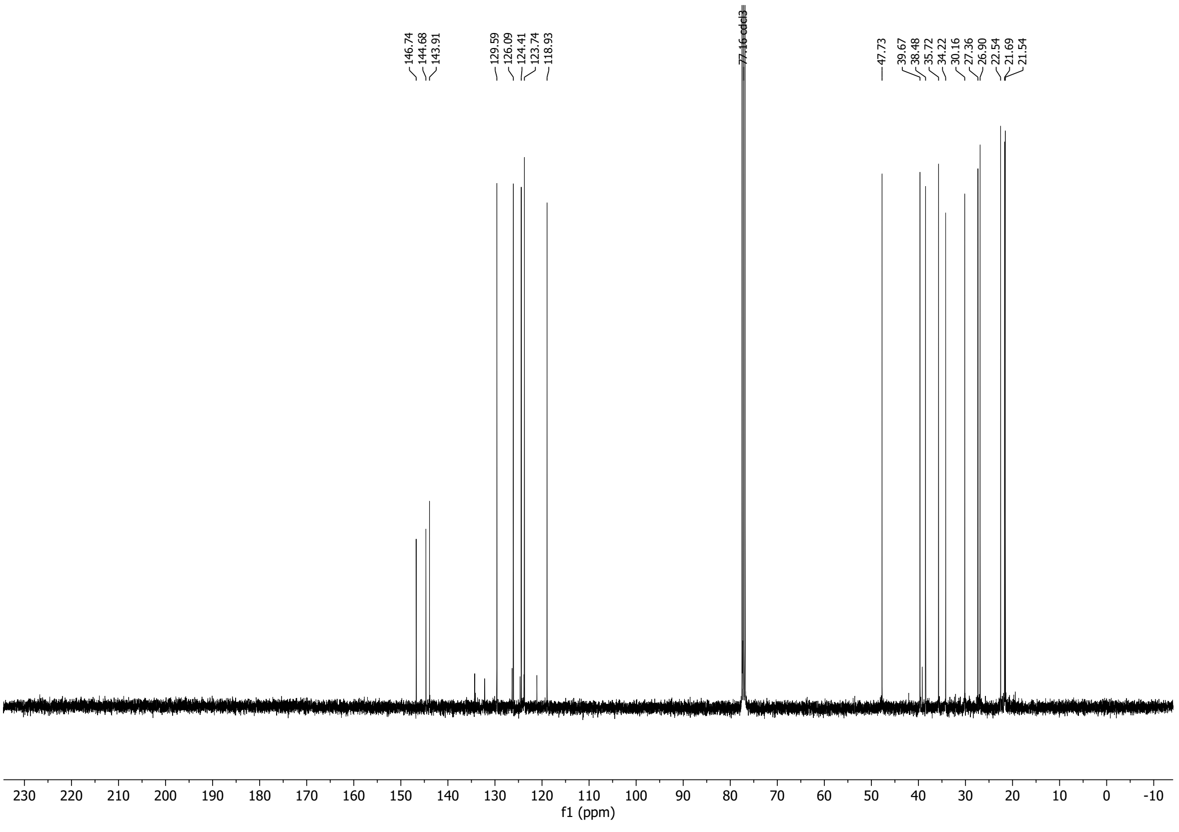

^{13}C NMR (100 MHz, $CDCl_3$) spectrum of compound **S50**.

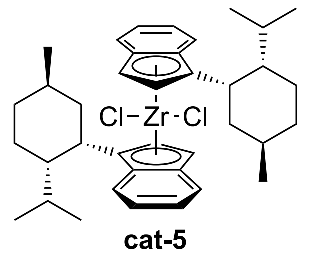

cat-5

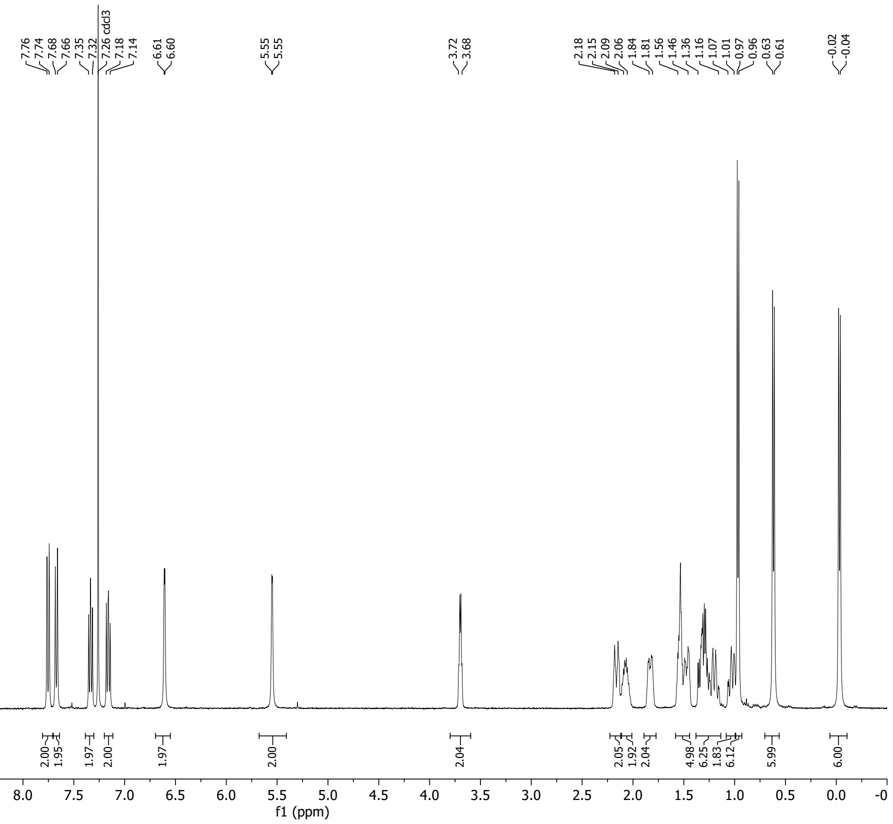

^{1}H NMR (400 MHz, $CDCl_3$) spectrum of **cat-5**.

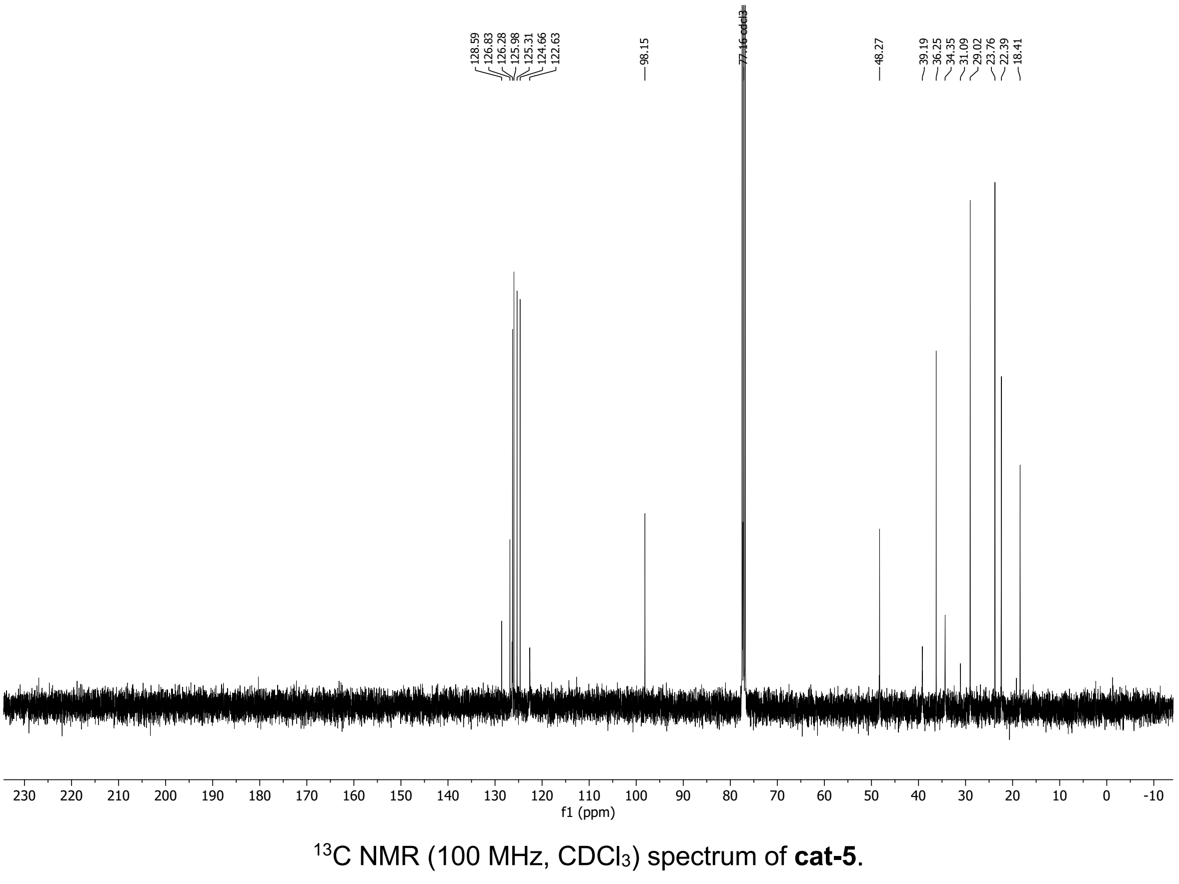

^{13}C NMR (100 MHz, $CDCl_3$) spectrum of **cat-5**.

HPLC and GC reports

```
Data File D:\AK_BREIT\DATA\TIBU\DEF_GC 2019-10-24 11-02-17\CM-III-454.D
Sample Name: CM-III-454

=====================================================================
Acq. Operator   : SYSTEM                          Seq. Line :   2
Acq. Instrument : GC6850_2                         Location : Vial 22
Injection Date  : 10/24/2019 11:11:27 AM                Inj :   1
                                                 Inj Volume : 0.2 µl
Acq. Method     : D:\AK_BREIT\DATA\TIBU\DEF_GC 2019-10-24 11-02-17\RAMPAB60_5CMIN_TO200.M
Last changed    : 10/24/2019 11:02:17 AM by SYSTEM
Analysis Method : D:\AK_BREIT\DATA\TIBU\DEF_GC 2019-10-24 11-02-17\RAMPAB60_5CMIN_TO200.M (
                  Sequence Method)
Last changed    : 8/25/2021 11:00:57 AM by SYSTEM
                  (modified after loading)
Method Info     : Hydroformylierung von Allylalkohol

Additional Info : Peak(s) manually integrated
```

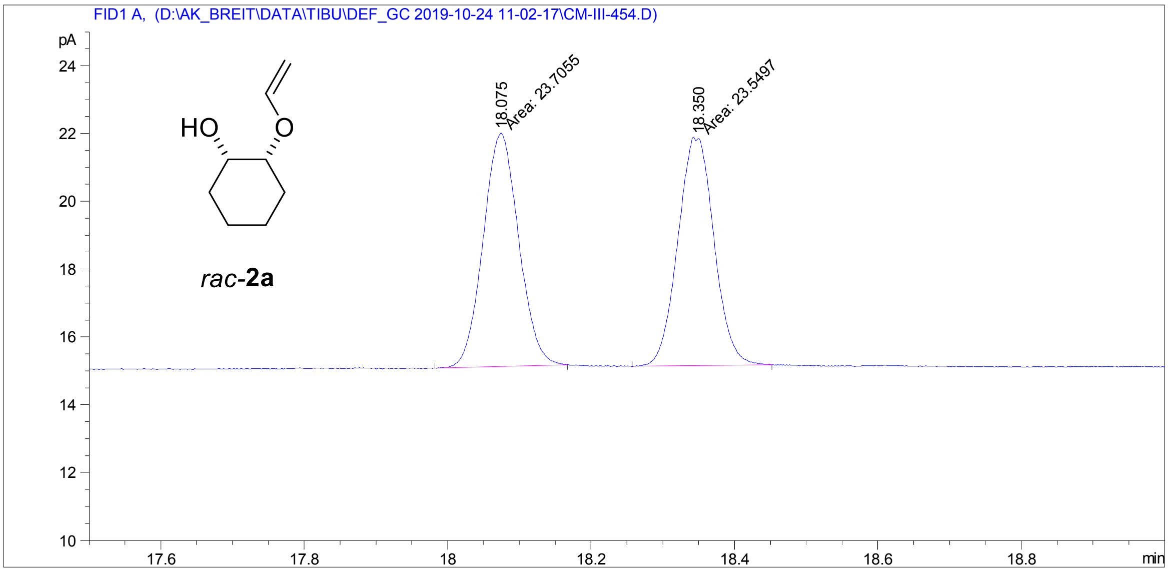

===

Area Percent Report

Peak #	RetTime [min]	Type	Width [min]	Area [pA*s]	Height [pA]	Area %
1	18.075	MM	0.0573	23.70549	6.89026	50.16481
2	18.350	MM	0.0584	23.54972	6.71996	49.83519
Totals :				47.25521	13.61022	

===

*** End of Report ***

GC

GC chromatogram of *rac*-**2a**.

Data File D:\AK_BREIT\DATA\TIBU\DEF_GC 2019-10-24 13-27-32\CM-III-453-B1.D
Sample Name: CM-III-453

===

Acq. Operator : SYSTEM Seq. Line : 2
Acq. Instrument : GC6850_2 Location : Vial 23
Injection Date : 10/24/2019 1:40:10 PM Inj : 1
Inj Volume : 0.2 µl
Acq. Method : D:\AK_BREIT\DATA\TIBU\DEF_GC 2019-10-24 13-27-32\RAMPAB60_5CMIN_TO200.M
Last changed : 10/24/2019 1:27:32 PM by SYSTEM
Analysis Method : D:\AK_BREIT\DATA\TIBU\DEF_GC 2019-10-24 13-27-32\RAMPAB60_5CMIN_TO200.M (Sequence Method)
Last changed : 8/25/2021 10:59:13 AM by SYSTEM
(modified after loading)
Method Info : Hydroformylierung von Allylalkohol

Additional Info : Peak(s) manually integrated

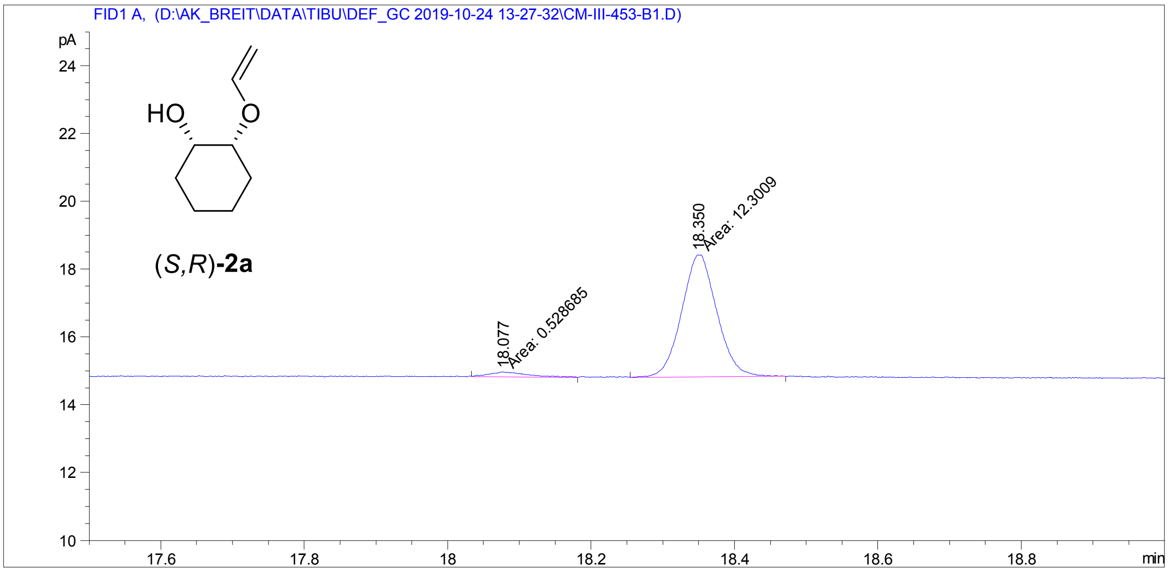

===

Area Percent Report

===

Peak #	RetTime [min]	Type	Width [min]	Area [pA*s]	Height [pA]	Area %
1	18.077	MM	0.0631	5.28685e-1	1.39655e-1	4.12084
2	18.350	MM	0.0567	12.30086	3.61494	95.87916
Totals :				12.82954	3.75459	

===

*** End of Report ***

GC chromatogram of *(S,R)*-**2a**.

SHIMADZU LabSolutions

Analysis Report

<Sample Information>

Sample Name	: cm-886F1		
Sample ID	: 60min 0_3ml		
Data Filename	: cm-886_nhept_iproh99_5_0_5_13.lcd		
Method Filename	: IA_CM-Desym_195nm-Heptane-iProp-99-01-60min-mGr-0_5mL.lcm		
Batch Filename	:		
Vial #	: 1-51	Sample Type	: Unknown
Injection Volume	: 10 uL		
Date Acquired	: 22.07.2021 12:02:45	Acquired by	: System Administrator
Date Processed	: 11.08.2021 14:57:57	Processed by	: System Administrator

<Chromatogram>

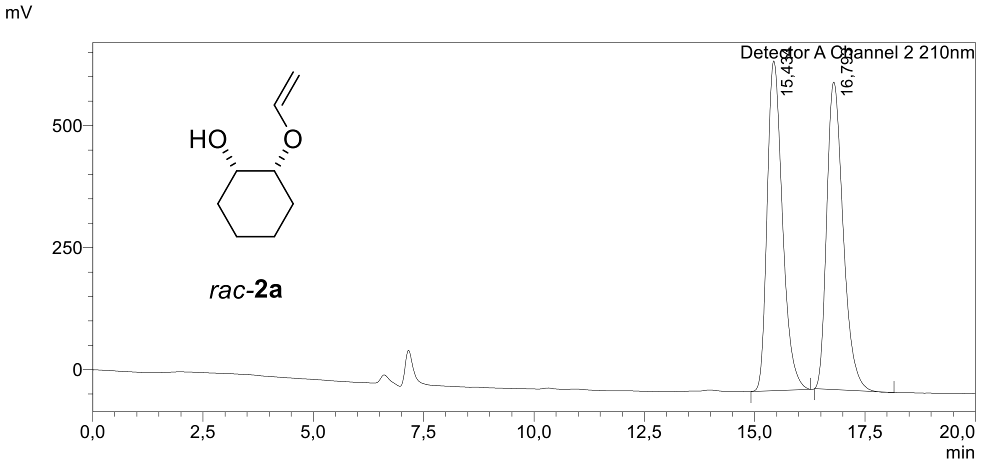

<Peak Table>

Detector A Channel 2 210nm

Peak#	Ret. Time	Area	Height	Conc.	Unit	Mark	Name
1	15,434	16282207	675440	49,941		M	
2	16,793	16320929	629978	50,059		M	
Total		32603136	1305418				

C:\LabSolutions\Data\Wiesler\cm-886_nhept_iproh99_5_0_5_13.lcd

HPLC chromatogram of *rac*-**2a**.

SHIMADZU LabSolutions

Analysis Report

<Sample Information>

Sample Name	: cm-912F1		
Sample ID	: 60min 0_5ml		
Data Filename	: cm-912F1_nhept_iPrOH_98_2_12.lcd		
Method Filename	: IA_CM-Desym_195nm-Heptane-iProp-99-01-60min-mGr-0_5mL.lcm		
Batch Filename	:		
Vial #	: 1-22	Sample Type	: Unknown
Injection Volume	: 10 uL		
Date Acquired	: 17.08.2021 15:48:28	Acquired by	: System Administrator
Date Processed	: 22.08.2021 10:37:58	Processed by	: System Administrator

<Chromatogram>

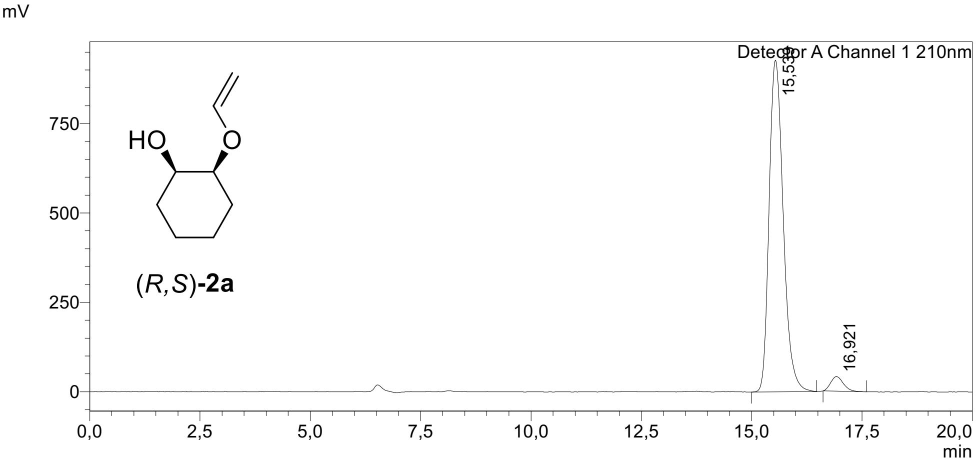

<Peak Table>

Detector A Channel 1 210nm

Peak#	Ret. Time	Area	Height	Conc.	Unit	Mark	Name
1	15,539	20612993	927394	96,192		M	
2	16,921	816055	40841	3,808		M	
Total		21429048	968234				

C:\LabSolutions\Data\Wiesler\cm-912F1_nhept_iPrOH_98_2_12.lcd

HPLC chromatogram of *(R,S)*-**2a**.

```
Data File D:\AK_BREIT\DATA\MD\DEF_GC 2019-12-17 16-26-51\CM-III-503F1.D
Sample Name: cm-III-503F1

    =====================================================================
    Acq. Operator   : SYSTEM                          Seq. Line :   4
    Acq. Instrument : GC6850_2                         Location : Vial 16
    Injection Date  : 12/17/2019 6:30:10 PM                 Inj :   1
                                                    Inj Volume : 0.2 µl
    Acq. Method     : D:\AK_BREIT\DATA\MD\DEF_GC 2019-12-17 16-26-51\RAMPAB60_5CMIN_TO200.M
    Last changed    : 12/17/2019 4:26:51 PM by SYSTEM
    Analysis Method : D:\AK_BREIT\DATA\MD\DEF_GC 2019-12-17 16-26-51\RAMPAB60_5CMIN_TO200.M (
                      Sequence Method)
    Last changed    : 8/25/2021 10:54:50 AM by SYSTEM
                      (modified after loading)
    Method Info     : Hydroformylierung von Allylalkohol

    Additional Info : Peak(s) manually integrated
```

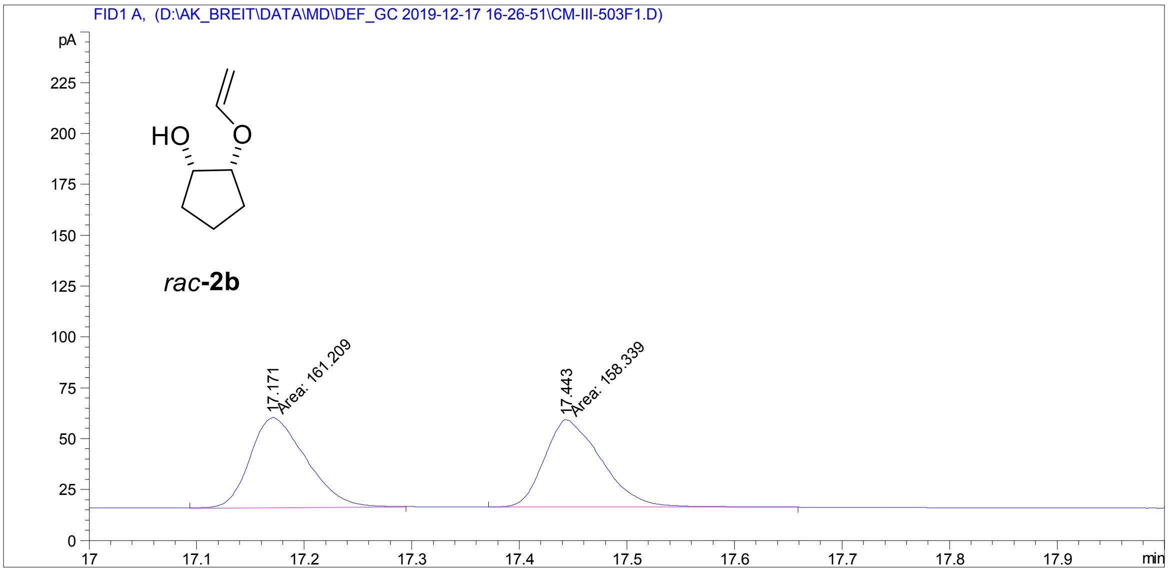

===

Area Percent Report

===

Peak #	RetTime [min]	Type	Width [min]	Area [pA*s]	Height [pA]	Area %
1	17.171	MM	0.0605	161.20923	44.43574	50.44904
2	17.443	MM	0.0614	158.33942	42.99392	49.55096
Totals :				319.54865	87.42966	

===

GC

*** End of Report ***

GC chromatogram of *rac*-**2b**.

```
Data File D:\AK_BREIT\DATA\CM\DEF_GC 2020-01-10 16-32-24\CM-III-515F1.D
Sample Name: cm-III-515F1

    =====================================================================
    Acq. Operator   : SYSTEM                          Seq. Line :   2
    Acq. Instrument : GC6850_2                         Location : Vial 15
    Injection Date  : 1/10/2020 4:46:12 PM                  Inj :   1
                                                     Inj Volume : 0.2 µl
    Acq. Method     : D:\AK_BREIT\DATA\CM\DEF_GC 2020-01-10 16-32-24\RAMPAB60_5CMIN_TO200.M
    Last changed    : 1/10/2020 4:32:24 PM by SYSTEM
    Analysis Method : D:\AK_BREIT\DATA\CM\DEF_GC 2020-01-10 16-32-24\RAMPAB60_5CMIN_TO200.M (
                      Sequence Method)
    Last changed    : 8/25/2021 10:51:02 AM by SYSTEM
                      (modified after loading)
    Method Info     : Hydroformylierung von Allylalkohol

    Additional Info : Peak(s) manually integrated
```

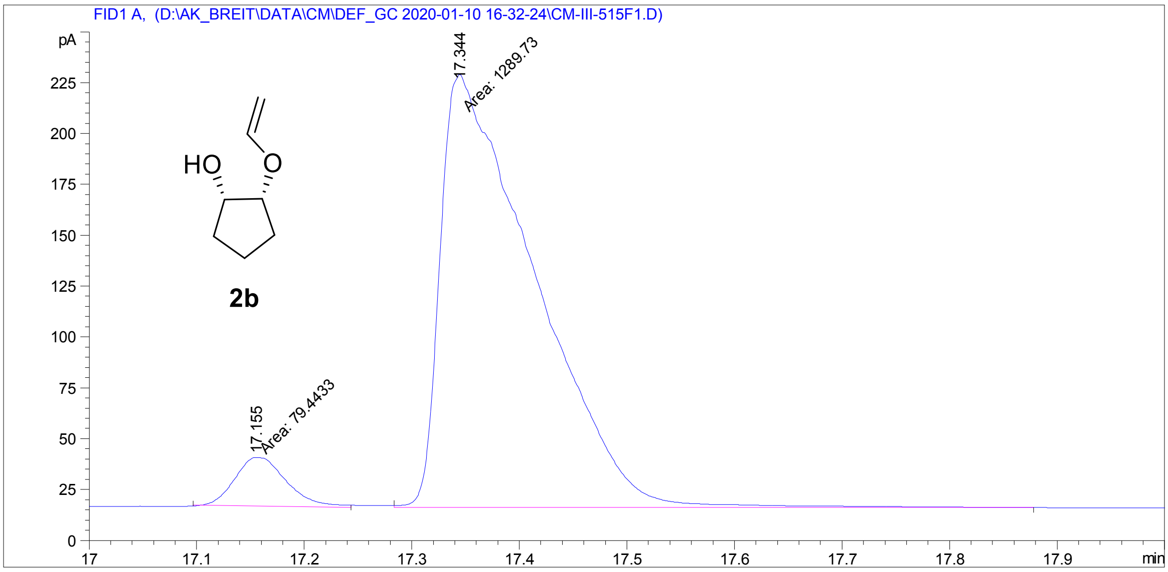


```
    =====================================================================
                             Area Percent Report
    =====================================================================
```

Peak #	RetTime [min]	Type	Width [min]	Area [pA*s]	Height [pA]	Area %
1	17.161	MM	0.0531	55.13348	17.29619	4.93736
2	17.353	MM	0.0977	1061.52661	181.16708	95.06264

Totals : 1116.66009 198.46328

GC chromatogram of **2b**.

SHIMADZU LabSolutions

Analysis Report

<Sample Information>

Sample Name	: CM-739-F1_0_5		
Sample ID	: 60min 0_5ml 01% iPrOH		
Data Filename	: CM-739-F1-Heptan-iPrOH-99-01_60min_0_5mL19.lcd		
Method Filename	: IA_CM-Desym_195nm-Heptane-iProp-99-01-60min-mGr-0_5mL.lcm		
Batch Filename	:		
Vial #	: 1-21	Sample Type	: Unknown
Injection Volume	: 5 uL		
Date Acquired	: 14.10.2020 14:16:41	Acquired by	: System Administrator
Date Processed	: 28.06.2021 16:30:35	Processed by	: System Administrator

<Chromatogram>

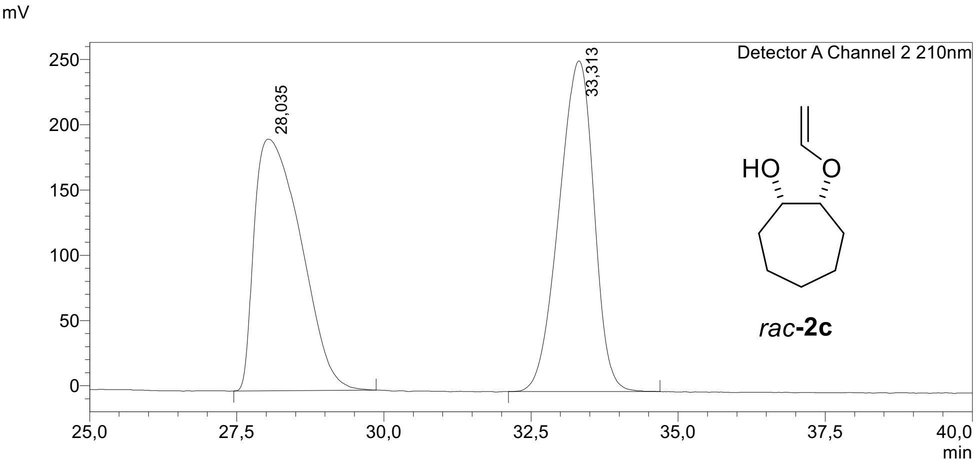

<Peak Table>

Detector A Channel 2 210nm

Peak#	Ret. Time	Area	Height	Conc.	Unit	Mark	Name
1	28,035	10646271	192830	50,377		M	
2	33,313	10487005	253369	49,623		M	
Total		21133277	446199				

C:\LabSolutions\Data\Wiesler\CM-739-F1-Heptan-iPrOH-99-01_60min_0_5mL19.lcd

HPLC chromatogram of *rac*-**2c**.

SHIMADZU LabSolutions

Analysis Report

<Sample Information>

Sample Name	: CM-740-F1_0_5		
Sample ID	: 60min 0_5ml 01% iPrOH		
Data Filename	: CM-740-F1-Heptan-iPrOH-99-01_60min_0_5mL20.lcd		
Method Filename	: IA_CM-Desym_195nm-Heptane-iProp-99-01-60min-mGr-0_5mL.lcm		
Batch Filename	:		
Vial #	: 1-22	Sample Type	: Unknown
Injection Volume	: 5 uL		
Date Acquired	: 14.10.2020 15:17:52	Acquired by	: System Administrator
Date Processed	: 28.06.2021 16:32:20	Processed by	: System Administrator

<Chromatogram>

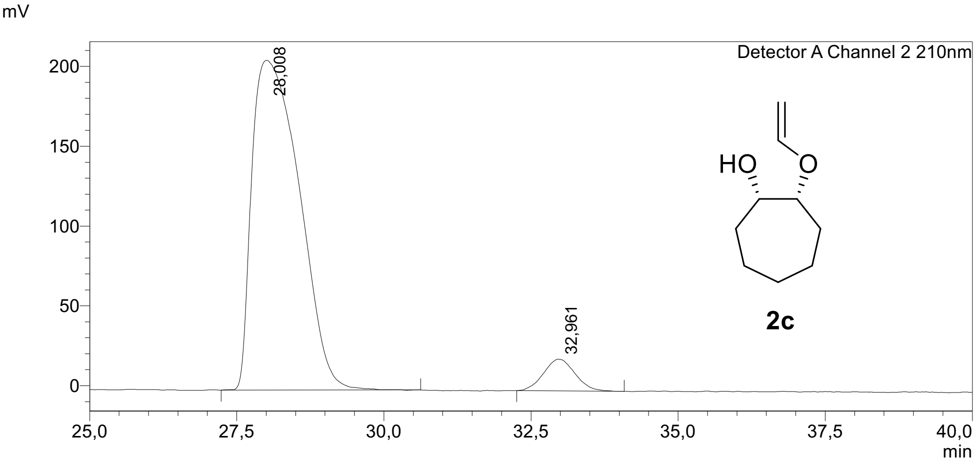

<Peak Table>

Detector A Channel 2 210nm

Peak#	Ret. Time	Area	Height	Conc.	Unit	Mark	Name
1	28,008	11608628	206608	94,033		M	
2	32,961	736698	19841	5,967		M	
Total		12345326	226449				

C:\LabSolutions\Data\Wiesler\CM-740-F1-Heptan-iPrOH-99-01_60min_0_5mL20.lcd

HPLC chromatogram of **2c**.

SHIMADZU LabSolutions **Analysis Report**

<Sample Information>

Sample Name	: cm-883F1		
Sample ID	: 60min 0_5ml		
Data Filename	: cm-883_nhept_iproh98_2_0_7.lcd		
Method Filename	: IA_CM-Desym_195nm-Heptane-iProp-99-01-60min-mGr-0_5mL.lcm		
Batch Filename	:		
Vial #	: 1-61	Sample Type	: Unknown
Injection Volume	: 10 uL		
Date Acquired	: 16.07.2021 11:17:21	Acquired by	: System Administrator
Date Processed	: 16.07.2021 12:23:18	Processed by	: System Administrator

<Chromatogram>

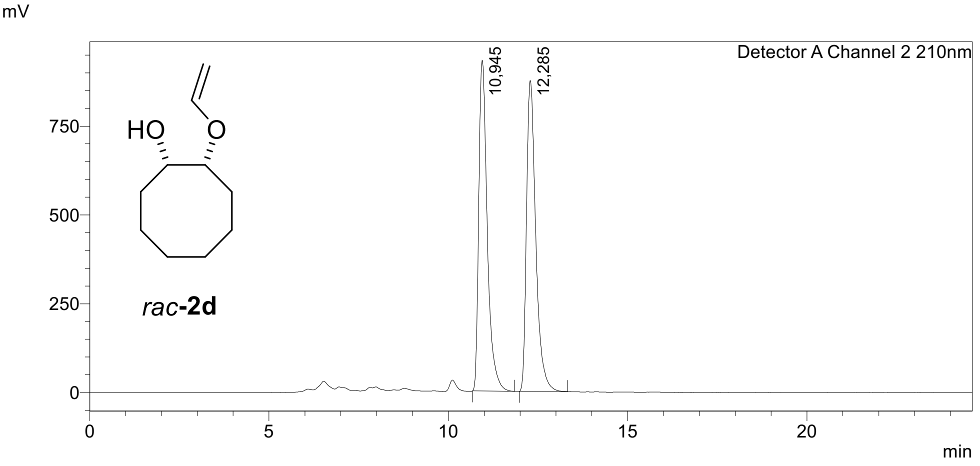

<Peak Table>

Detector A Channel 2 210nm

Peak#	Ret. Time	Area	Height	Conc.	Unit	Mark	Name
1	10,945	15380610	930953	49,434		M	
2	12,285	15733027	874964	50,566		M	
Total		31113637	1805917				

C:\LabSolutions\Data\Wiesler\cm-883_nhept_iproh98_2_0_7.lcd

HPLC chromatogram of *rac*-**2d**.

SHIMADZU LabSolutions

Analysis Report

<Sample Information>

Sample Name	: cm-884F1		
Sample ID	: 60min 0_5ml		
Data Filename	: cm-884_nhept_iproh98_2_0_9.lcd		
Method Filename	: IA_CM-Desym_195nm-Heptane-iProp-99-01-60min-mGr-0_5mL.lcm		
Batch Filename	:		
Vial #	: 1-62	Sample Type	: Unknown
Injection Volume	: 10 uL		
Date Acquired	: 16.07.2021 12:24:21	Acquired by	: System Administrator
Date Processed	: 31.07.2021 13:30:00	Processed by	: System Administrator

<Chromatogram>

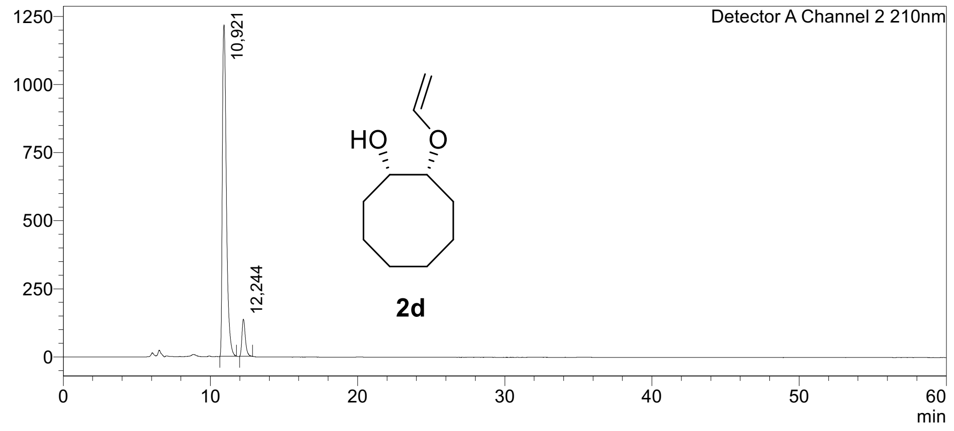

<Peak Table>

Detector A Channel 2 210nm

Peak#	Ret. Time	Area	Height	Conc.	Unit	Mark	Name
1	10,921	22516294	1216698	91,357		M	
2	12,244	2130199	136228	8,643		M	
Total		24646493	1352927				

C:\LabSolutions\Data\Wiesler\cm-884_nhept_iproh98_2_0_9.lcd

HPLC chromatogram of **2d**.

```
Data File D:\AK_BREIT\DATA\CM\DEF_GC 2020-06-10 07-14-43\CM-IV-662F1.D
Sample Name: cm-IV-662F1

    =====================================================================
    Acq. Operator   : SYSTEM                          Seq. Line :    3
    Acq. Instrument : GC6850_2                         Location : Vial 4
    Injection Date  : 6/10/2020 8:38:08 AM                  Inj :    1
                                                     Inj Volume : 0.2 µl
    Acq. Method     : D:\AK_BREIT\DATA\CM\DEF_GC 2020-06-10 07-14-43\RAMPAB80_1CMIN_TO120.M
    Last changed    : 6/10/2020 7:14:43 AM by SYSTEM
    Analysis Method : D:\AK_BREIT\DATA\CM\DEF_GC 2020-06-10 07-14-43\RAMPAB80_1CMIN_TO120.M (
                      Sequence Method)
    Last changed    : 8/25/2021 10:47:27 AM by SYSTEM
                      (modified after loading)
    Method Info     : Hydroformylierung von Allylalkohol

    Additional Info : Peak(s) manually integrated
```

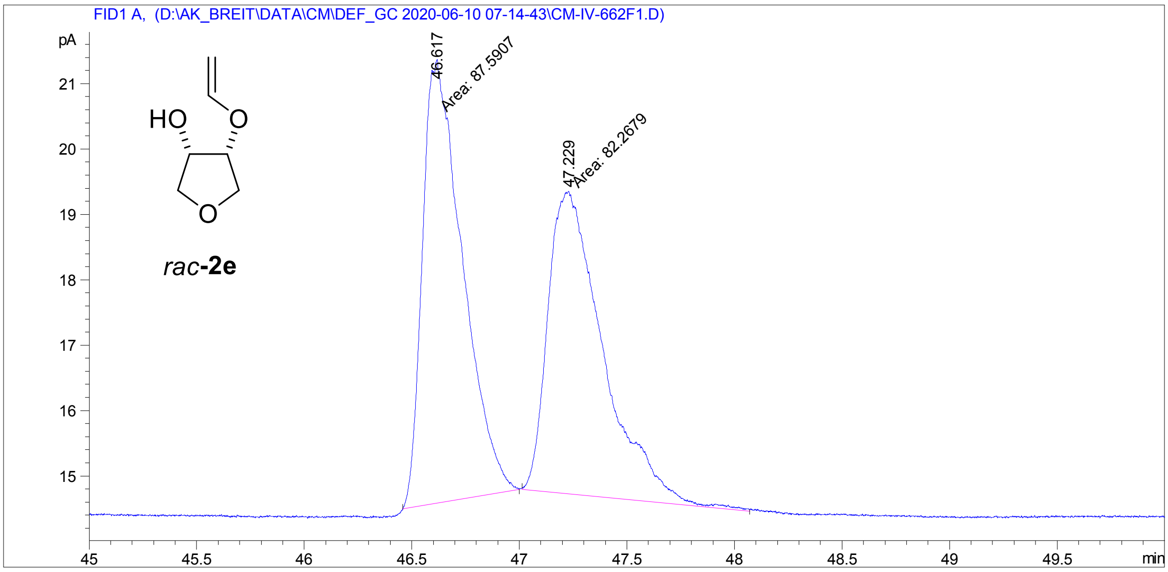

===

Area Percent Report

Peak #	RetTime [min]	Type	Width [min]	Area [pA*s]	Height [pA]	Area %
1	46.617	MM	0.2153	87.59073	6.77909	51.56683
2	47.229	MM	0.2964	82.26794	4.62657	48.43317
Totals :				169.85867	11.40566	

===

*** End of Report ***

GC chromatogram of *rac*-**2e**.

```
Data File D:\AK_BREIT\DATA\CM\DEF_GC 2020-06-10 07-14-43\CM-IV-663F1.D
Sample Name: cm-IV-663F1

    =====================================================================
    Acq. Operator   : SYSTEM                          Seq. Line :   2
    Acq. Instrument : GC6850_2                         Location : Vial 3
    Injection Date  : 6/10/2020 7:41:41 AM                  Inj :   1
                                                     Inj Volume : 0.2 µl
    Acq. Method     : D:\AK_BREIT\DATA\CM\DEF_GC 2020-06-10 07-14-43\RAMPAB80_1CMIN_TO120.M
    Last changed    : 6/10/2020 7:14:43 AM by SYSTEM
    Analysis Method : D:\AK_BREIT\DATA\CM\DEF_GC 2020-06-10 07-14-43\RAMPAB80_1CMIN_TO120.M (
                      Sequence Method)
    Last changed    : 8/25/2021 10:48:17 AM by SYSTEM
                      (modified after loading)
    Method Info     : Hydroformylierung von Allylalkohol

    Additional Info : Peak(s) manually integrated
```

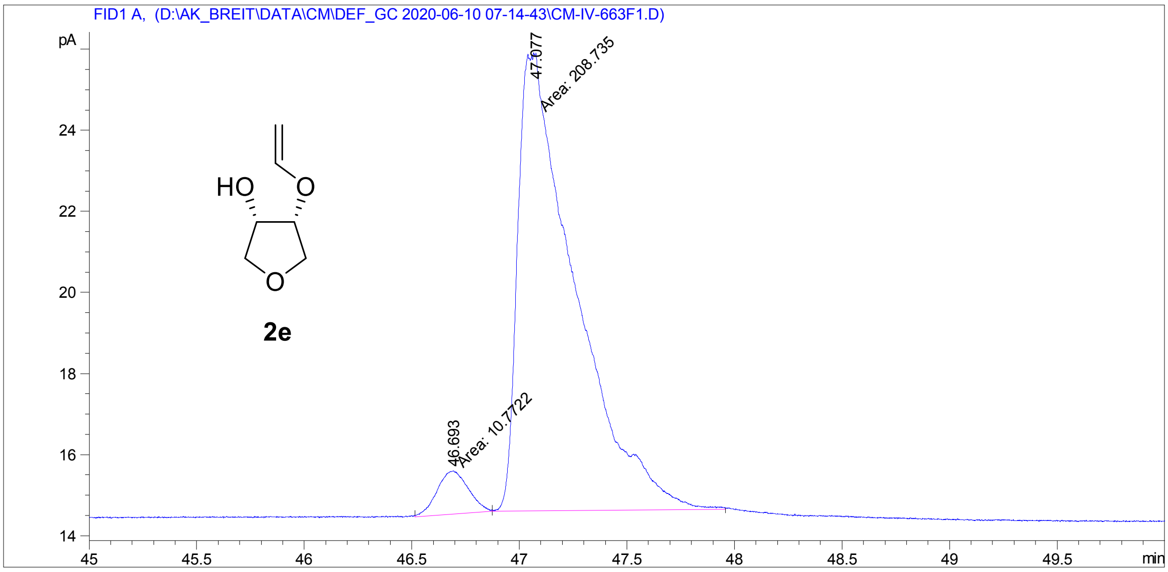

Area Percent Report

Peak #	RetTime [min]	Type	Width [min]	Area [pA*s]	Height [pA]	Area %
1	46.693	MM	0.1681	10.77222	1.06830	4.90747
2	47.077	MM	0.3083	208.73453	11.28299	95.09253
Totals :				219.50675	12.35129	

GC

*** End of Report ***

GC chromatogram of **2e**.

SHIMADZU LabSolutions

Analysis Report

<Sample Information>

Sample Name	: CM-IV-714-F1		
Sample ID	: 60min 0_5ml 02% iPrOH		
Data Filename	: CM-714-F1-Heptan-iPrOH-95-05_60min_0_5mL11.lcd		
Method Filename	: IA_CM-Desym_195nm-Heptane-iProp-98-02-60min-mGr-0_5mL.lcm		
Batch Filename	:		
Vial #	: 1-14	Sample Type	: Unknown
Injection Volume	: 5 uL		
Date Acquired	: 02.09.2020 16:41:06	Acquired by	: System Administrator
Date Processed	: 28.06.2021 16:45:06	Processed by	: System Administrator

<Chromatogram>

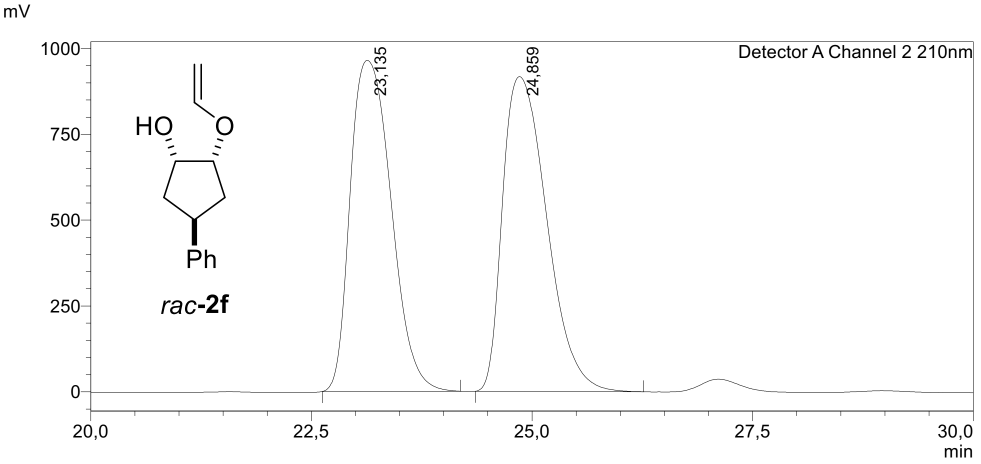

<Peak Table>

Detector A Channel 2 210nm

Peak#	Ret. Time	Area	Height	Conc.	Unit	Mark	Name
1	23,135	31129067	964359	49,343		M	
2	24,859	31958275	916721	50,657		M	
Total		63087343	1881080				

C:\LabSolutions\Data\Wiesler\CM-714-F1-Heptan-iPrOH-95-05_60min_0_5mL11.lcd

HPLC chromatogram of *rac*-**2f**.

SHIMADZU LabSolutions

Analysis Report

<Sample Information>

Sample Name	: CM-IV-715-F1		
Sample ID	: 60min 0_5ml 02% iPrOH		
Data Filename	: CM-715-F1-Heptan-iPrOH-98-02_60min_0_5mL13.lcd		
Method Filename	: IA_CM-Desym_195nm-Heptane-iProp-98-02-60min-mGr-0_5mL.lcm		
Batch Filename	:		
Vial #	: 1-13	Sample Type	: Unknown
Injection Volume	: 5 uL		
Date Acquired	: 02.09.2020 19:36:08	Acquired by	: System Administrator
Date Processed	: 28.06.2021 16:46:03	Processed by	: System Administrator

<Chromatogram>

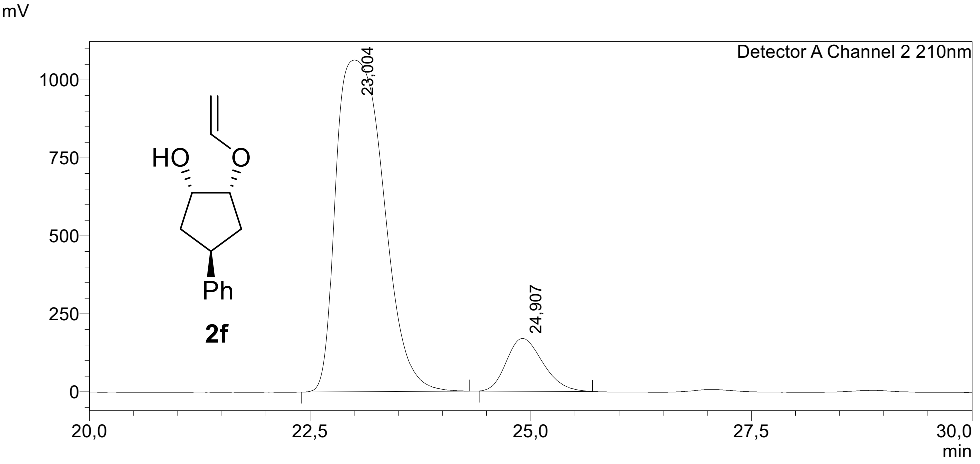

<Peak Table>

Detector A Channel 2 210nm

Peak#	Ret. Time	Area	Height	Conc.	Unit	Mark	Name
1	23,004	40033255	1064057	89,198		M	
2	24,907	4847832	169226	10,802		M	
Total		44881087	1233283				

C:\LabSolutions\Data\Wiesler\CM-715-F1-Heptan-iPrOH-98-02_60min_0_5mL13.lcd

HPLC chromatogram of **2f**.

SHIMADZU LabSolutions

Analysis Report

<Sample Information>

Sample Name	: CM-III-435-F1-kontr		
Sample ID	: 60min 0_7ml 5% iProp		
Data Filename	: CM-III-435-F1-kontr_Heptan-iProp-95-05_60min3.lcd		
Method Filename	: IA_CM-Acetalspaltung-Heptane-iProp-95-05-60min-mGr-0_7mL.lcm		
Batch Filename	:		
Vial #	: 1-10	Sample Type	: Unknown
Injection Volume	: 10 uL		
Date Acquired	: 18.10.2019 17:00:23	Acquired by	: System Administrator
Date Processed	: 18.10.2019 18:13:26	Processed by	: System Administrator

<Chromatogram>

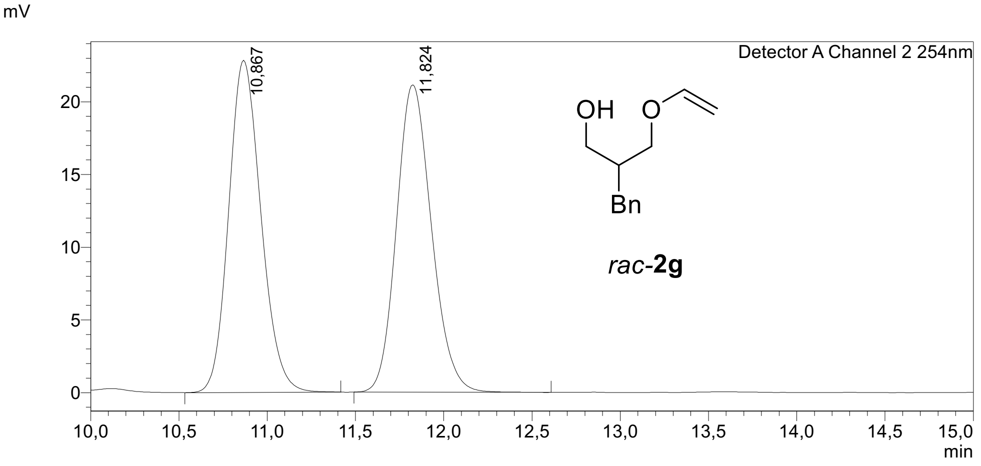

<Peak Table>

Detector A Channel 2 254nm

Peak#	Ret. Time	Area	Height	Conc.	Unit	Mark	Name
1	10,867	290290	22838	49,939		M	
2	11,824	291002	21137	50,061		M	
Total		581292	43974				

C:\LabSolutions\Data\Wiesler\CM-III-435-F1-kontr_Heptan-iProp-95-05_60min3.lcd

HPLC chromatogram of *rac*-**2g**.

SHIMADZU LabSolutions

Analysis Report

<Sample Information>

Sample Name	: CM-III-444-F1-kontr		
Sample ID	: 60min 0_7ml 5% iProp		
Data Filename	: CM-III-444-F1-kontr_Heptan-iProp-95-05_60min2.lcd		
Method Filename	: IA_CM-Acetalspaltung-Heptane-iProp-95-05-60min-mGr-0_7mL.lcm		
Batch Filename	:		
Vial #	: 1-9	Sample Type	: Unknown
Injection Volume	: 10 uL		
Date Acquired	: 18.10.2019 14:40:14	Acquired by	: System Administrator
Date Processed	: 18.10.2019 16:58:45	Processed by	: System Administrator

<Chromatogram>

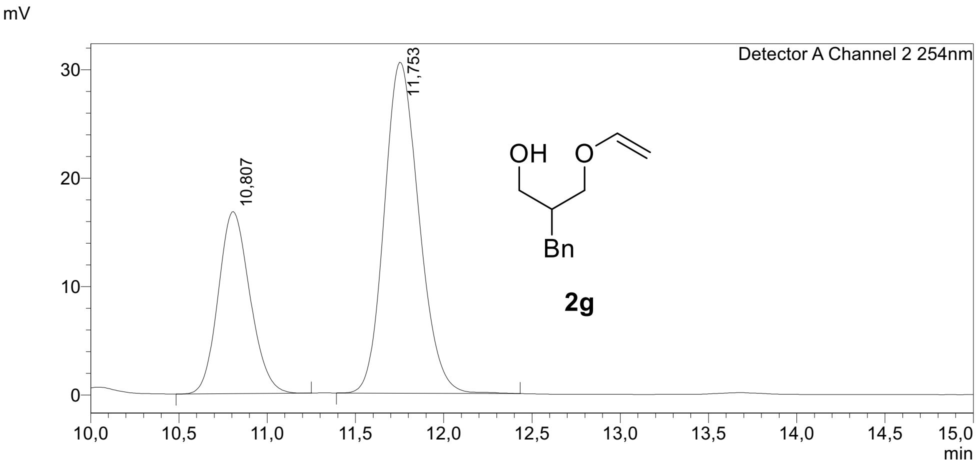

<Peak Table>

Detector A Channel 2 254nm

Peak#	Ret. Time	Area	Height	Conc.	Unit	Mark	Name
1	10,807	210037	16782	33,444		M	
2	11,753	417992	30508	66,556		M	
Total		628029	47290				

C:\LabSolutions\Data\Wiesler\CM-III-444-F1-kontr_Heptan-iProp-95-05_60min2.lcd

HPLC chromatogram of **2g**.

Albert-Ludwigs-Universität Freiburg
Institut für Organische Chemie

Sample ID: ma.cm-IV-700

Method: C:\Enterprise\Methods\HPLC2\5_ChiralPAK AD-3\System2, AD-3, Hep_IPA 99,5_0,5, 0,5ml, 22°C.met

Vial: P1-F3

Injection Volume: 5µL

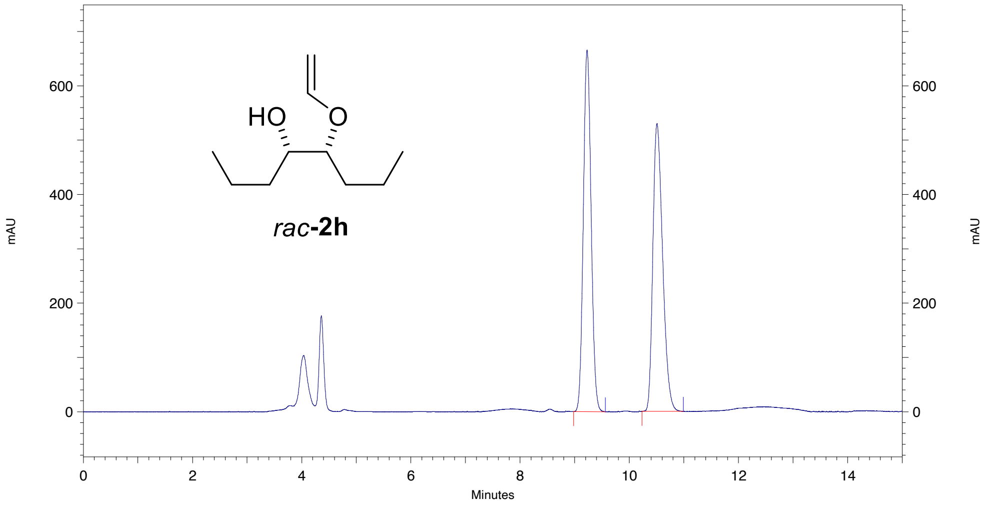

HPLC chromatogram of *rac*-**2h**.

Spectrum Max Plot

Results

Peak Number	Retention Time	Area Percent	Area
1	9,227	50,096	884707336
2	10,507	49,904	881309894
Totals		100,000	1766017230

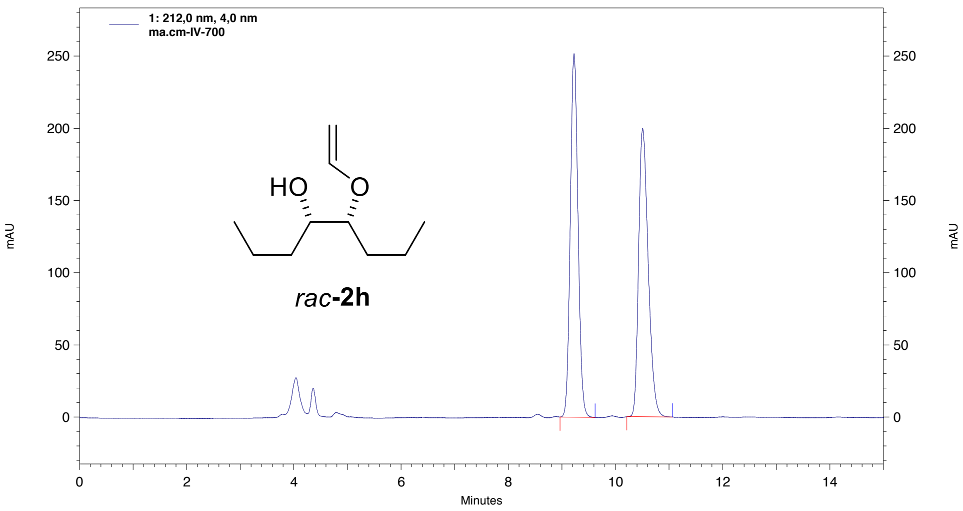

1: 212,0 nm, 4,0 nm

Results

Peak Number	Retention Time	Area Percent	Area
1	9,227	50,231	337865847
2	10,507	49,769	334760306
Totals		100,000	672626153

HPLC chromatogram of *rac*-**2h**.

Albert-Ludwigs-Universität Freiburg
Institut für Organische Chemie

Sample ID: ma.cm-IV-705ee

Method: C:\Enterprise\Methods\HPLC2\5_ChiralPAK AD-3\System2, AD-3, Hep_IPA 99,5_0,5, 0,5ml, 22°C.met

Vial: P1-D1

Injection Volume: 5µL

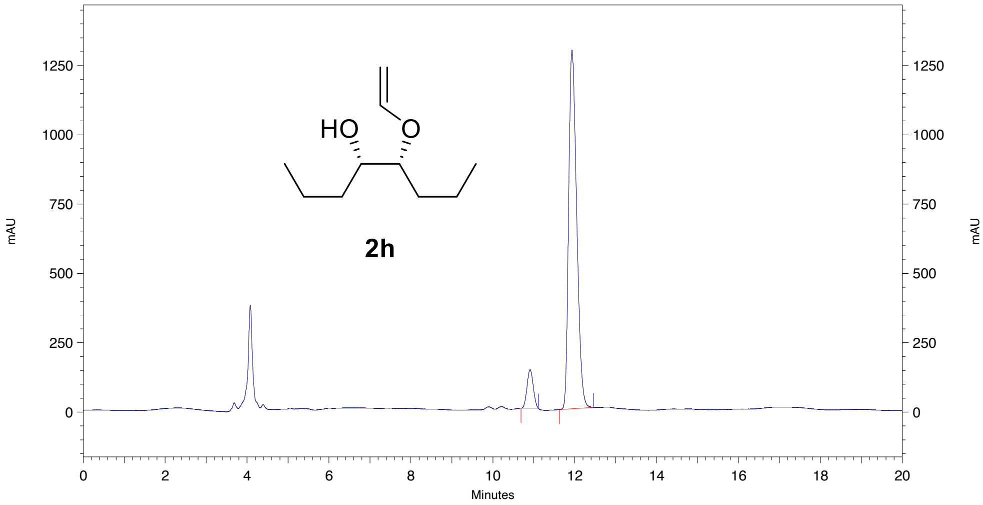

HPLC chromatogram of **2h**.

Spectrum Max Plot
Results

Peak Number	Retention Time	Area Percent	Area
1	10,910	7,703	196817883
2	11,933	92,297	2358197758
Totals		100,000	2555015641

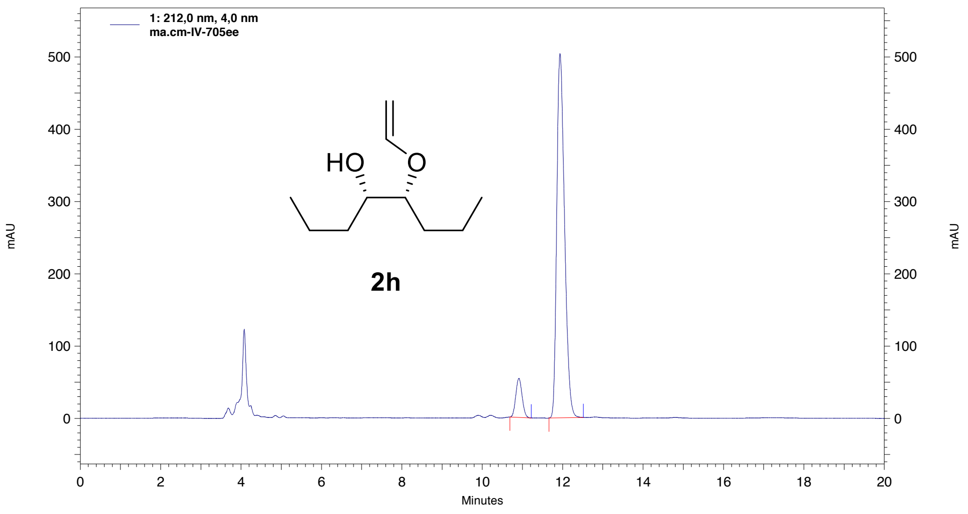

1: 212,0 nm, 4,0 nm
Results

Peak Number	Retention Time	Area Percent	Area
1	10,910	8,069	79794487
2	11,933	91,931	909054159
Totals		100,000	988848646

HPLC chromatogram of **2h**.

SHIMADZU LabSolutions

Analysis Report

<Sample Information>

Sample Name	: cm-IV-762-F1		
Sample ID	: 60min 0_5ml 1% iPrOH		
Data Filename	: CM-IV-762F1-Heptan-iPrOH-99-01_60min_0_5mL28.lcd		
Method Filename	: IA_CM-Desym_195nm-Heptane-iProp-99-01-60min-mGr-0_5mL.lcm		
Batch Filename	:		
Vial #	: 1-3	Sample Type	: Unknown
Injection Volume	: 5 uL		
Date Acquired	: 30.11.2020 09:52:54	Acquired by	: System Administrator
Date Processed	: 25.06.2021 14:28:35	Processed by	: System Administrator

<Chromatogram>

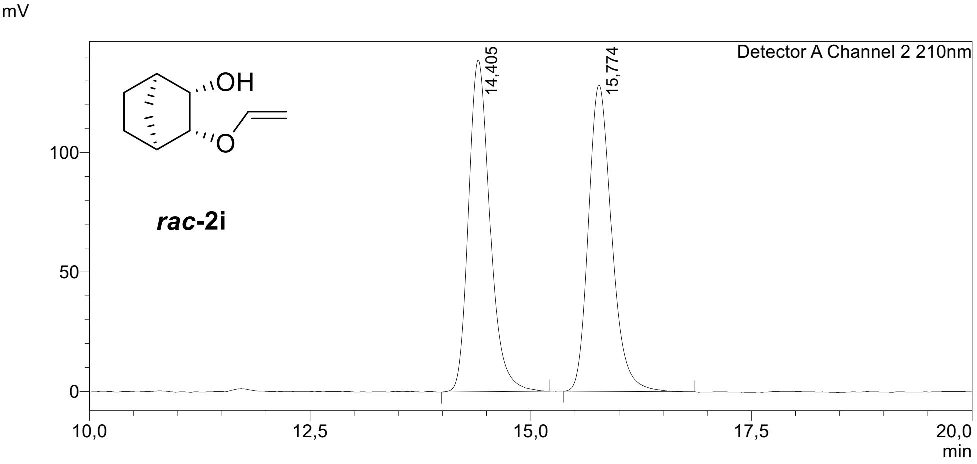

<Peak Table>

Detector A Channel 2 210nm

Peak#	Ret. Time	Area	Height	Conc.	Unit	Mark	Name
1	14,405	2352357	138800	49,997		M	
2	15,774	2352597	128295	50,003		M	
Total		4704954	267094				

C:\LabSolutions\Data\Wiesler\CM-IV-762F1-Heptan-iPrOH-99-01_60min_0_5mL28.lcd

HPLC chromatogram of *rac*-**2i**.

SHIMADZU LabSolutions

Analysis Report

<Sample Information>

Sample Name	: cm-IV-795-F1		
Sample ID	: 60min 0_5ml 1% iPrOH		
Data Filename	: CM-IV-795F1-Heptan-iPrOH-99-01_60min_0_5mL25.lcd		
Method Filename	: IA_CM-Desym_195nm-Heptane-iProp-99-01-60min-mGr-0_5mL.lcm		
Batch Filename	:		
Vial #	: 1-65	Sample Type	: Unknown
Injection Volume	: 5 uL		
Date Acquired	: 27.11.2020 14:54:35	Acquired by	: System Administrator
Date Processed	: 28.06.2021 16:35:42	Processed by	: System Administrator

<Chromatogram>

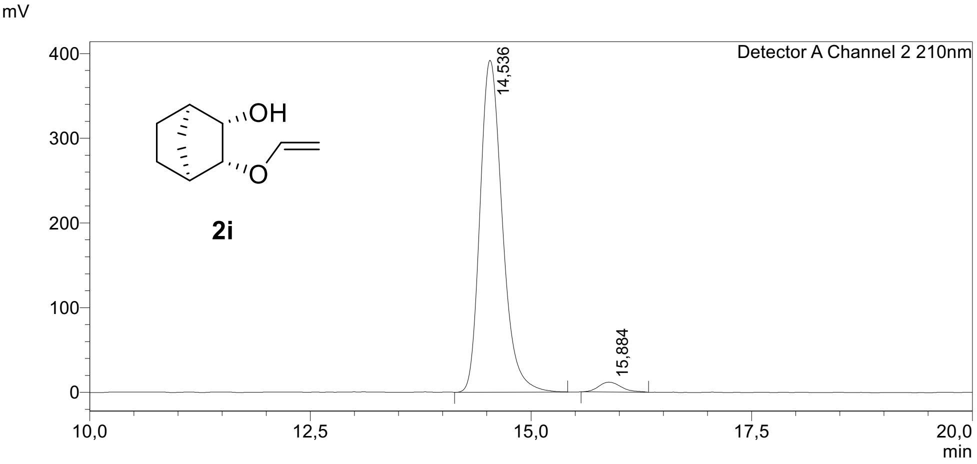

<Peak Table>

Detector A Channel 2 210nm

Peak#	Ret. Time	Area	Height	Conc.	Unit	Mark	Name
1	14,536	6861795	391756	97,066		M	
2	15,884	207392	11611	2,934		M	
Total		7069187	403367				

HPLC chromatogram of **2i**.

SHIMADZU LabSolutions

Analysis Report

<Sample Information>

Sample Name	: cm-IV-791-F1		
Sample ID	: 60min 0_5ml 05% iPrOH		
Data Filename	: CM-IV-791F1-Heptan-iPrOH-9905-005_60min_0_5mL31.lcd		
Method Filename	: IA_CM-Desym_195nm-Heptane-iProp-99-01-60min-mGr-0_5mL.lcm		
Batch Filename	:		
Vial #	: 1-64	Sample Type	: Unknown
Injection Volume	: 3 uL		
Date Acquired	: 30.11.2020 15:51:32	Acquired by	: System Administrator
Date Processed	: 28.06.2021 16:36:51	Processed by	: System Administrator

<Chromatogram>

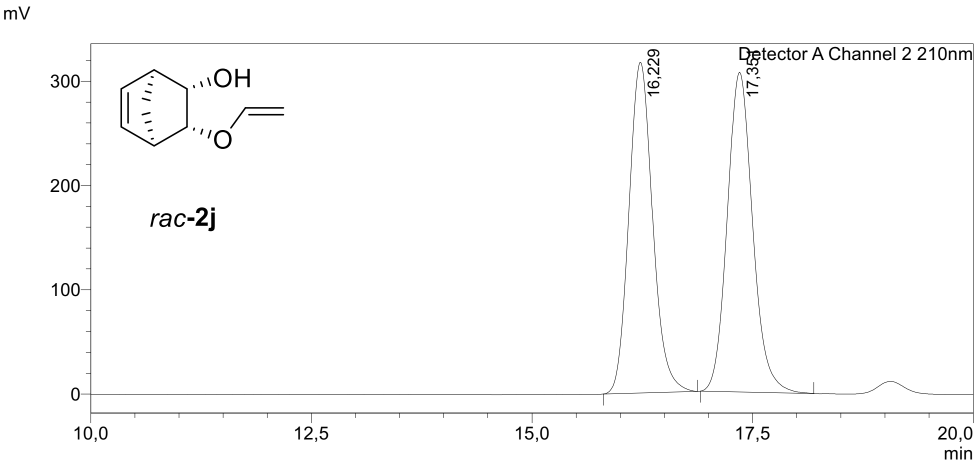

<Peak Table>

Detector A Channel 2 210nm

Peak#	Ret. Time	Area	Height	Conc.	Unit	Mark	Name
1	16,229	5931063	317073	49,148		M	
2	17,351	6136711	306489	50,852		M	
Total		12067774	623562				

C:\LabSolutions\Data\Wiesler\CM-IV-791F1-Heptan-iPrOH-9905-005_60min_0_5mL31.lcd

HPLC chromatogram of *rac*-**2j**.

SHIMADZU LabSolutions

Analysis Report

<Sample Information>

Sample Name	: cm-IV-792-F1		
Sample ID	: 60min 0_5ml 05% iPrOH		
Data Filename	: CM-IV-792F1-Heptan-iPrOH-9905-005_60min_0_5mL32.lcd		
Method Filename	: IA_CM-Desym_195nm-Heptane-iProp-99-01-60min-mGr-0_5mL.lcm		
Batch Filename	:		
Vial #	: 1-63	Sample Type	: Unknown
Injection Volume	: 5 uL		
Date Acquired	: 30.11.2020 16:53:17	Acquired by	: System Administrator
Date Processed	: 28.06.2021 16:39:39	Processed by	: System Administrator

<Chromatogram>

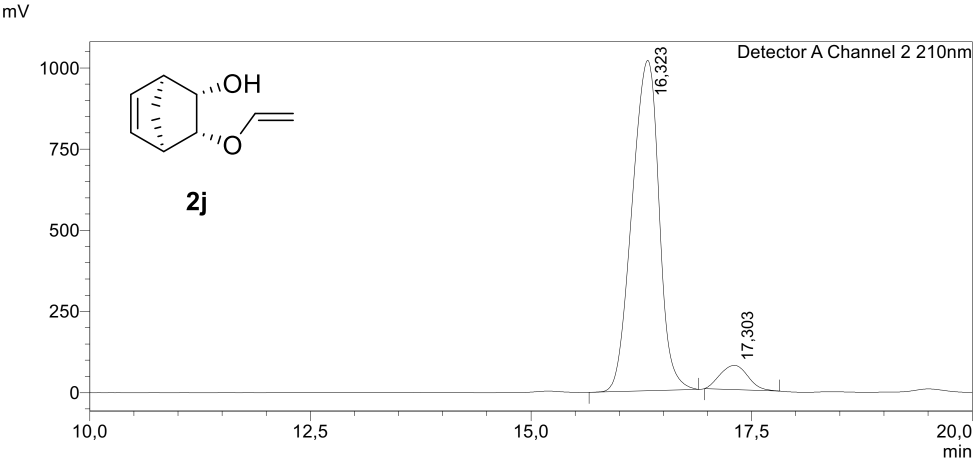

<Peak Table>

Detector A Channel 2 210nm

Peak#	Ret. Time	Area	Height	Conc.	Unit	Mark	Name
1	16,323	21604170	1017627	92,994		M	
2	17,303	1627660	74468	7,006		M	
Total		23231829	1092095				

C:\LabSolutions\Data\Wiesler\CM-IV-792F1-Heptan-iPrOH-9905-005_60min_0_5mL32.lcd

HPLC chromatogram of **2j**.

SHIMADZU LabSolutions

Analysis Report

<Sample Information>

Sample Name	: cm-891F1		
Sample ID	: 60min 1ml		
Data Filename	: cm-891_nhept_iPrOH_99_5_0_5_19.lcd		
Method Filename	: IA_CM-Desym_195nm-Heptane-iProp-99-01-60min-mGr-0_5mL.lcm		
Batch Filename	:		
Vial #	: 1-41	Sample Type	: Unknown
Injection Volume	: 10 uL		
Date Acquired	: 28.07.2021 17:13:45	Acquired by	: System Administrator
Date Processed	: 28.07.2021 17:41:48	Processed by	: System Administrator

<Chromatogram>

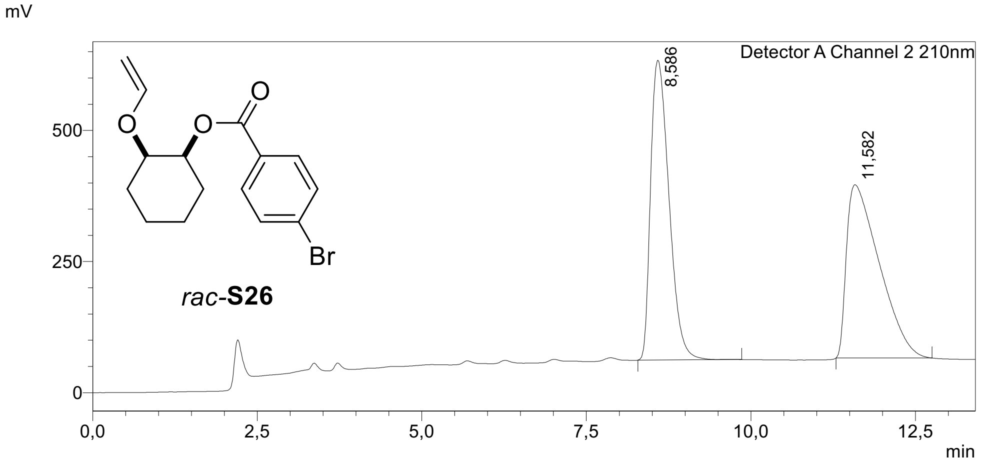

<Peak Table>

Detector A Channel 2 210nm

Peak#	Ret. Time	Area	Height	Conc.	Unit	Mark	Name
1	8,586	11114751	571429	49,285		M	
2	11,582	11437191	330567	50,715		M	
Total		22551943	901996				

C:\LabSolutions\Data\Wiesler\cm-891_nhept_iPrOH_99_5_0_5_19.lcd

HPLC chromatogram of *rac*-**S26**.

SHIMADZU LabSolutions

Analysis Report

<Sample Information>

Sample Name : cm-907F1
Sample ID : 60min 0_5ml
Data Filename : cm-907F1_nhept_iPrOH_99_5_0_541.lcd
Method Filename : IA_CM-Desym_195nm-Heptane-iProp-99-01-60min-mGr-0_5mL.lcm
Batch Filename :
Vial # : 1-63 Sample Type : Unknown
Injection Volume : 10 uL
Date Acquired : 12.08.2021 11:10:42 Acquired by : System Administrator
Date Processed : 12.08.2021 13:58:02 Processed by : System Administrator

<Chromatogram>

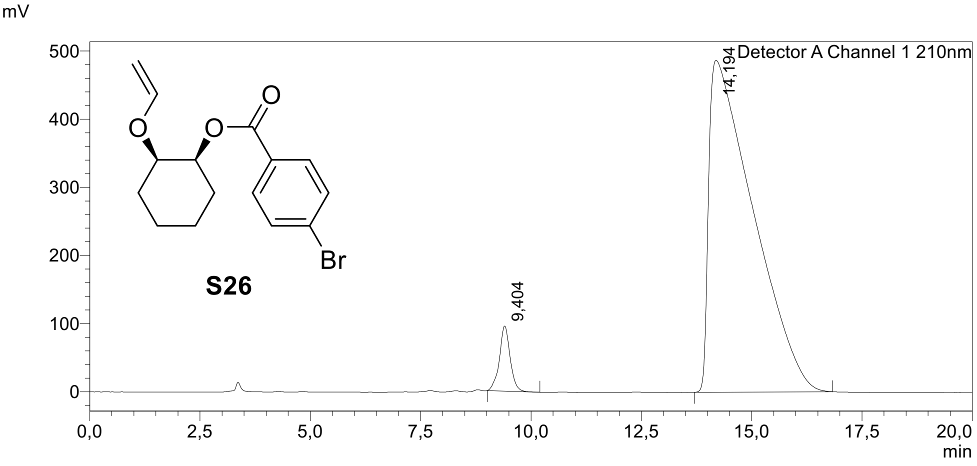

<Peak Table>

Detector A Channel 1 210nm

Peak#	Ret. Time	Area	Height	Conc.	Unit	Mark	Name
1	9,404	1626245	95604	4,556		M	
2	14,194	34069275	486545	95,444		M	
Total		35695521	582149				

C:\LabSolutions\Data\Wiesler\cm-907F1_nhept_iPrOH_99_5_0_541.lcd

HPLC chromatogram of **S26**.

SHIMADZU LabSolutions

Analysis Report

<Sample Information>

Sample Name	: cm-893F1		
Sample ID	: 60min 0_5ml		
Data Filename	: cm-893F1_nhept_iPrOH_95_5.lcd		
Method Filename	: IA_CM-Desym_195nm-Heptane-iProp-99-01-60min-mGr-0_5mL.lcm		
Batch Filename	:		
Vial #	: 1-1	Sample Type	: Unknown
Injection Volume	: 10 uL		
Date Acquired	: 13.08.2021 10:47:48	Acquired by	: System Administrator
Date Processed	: 22.08.2021 10:14:07	Processed by	: System Administrator

<Chromatogram>

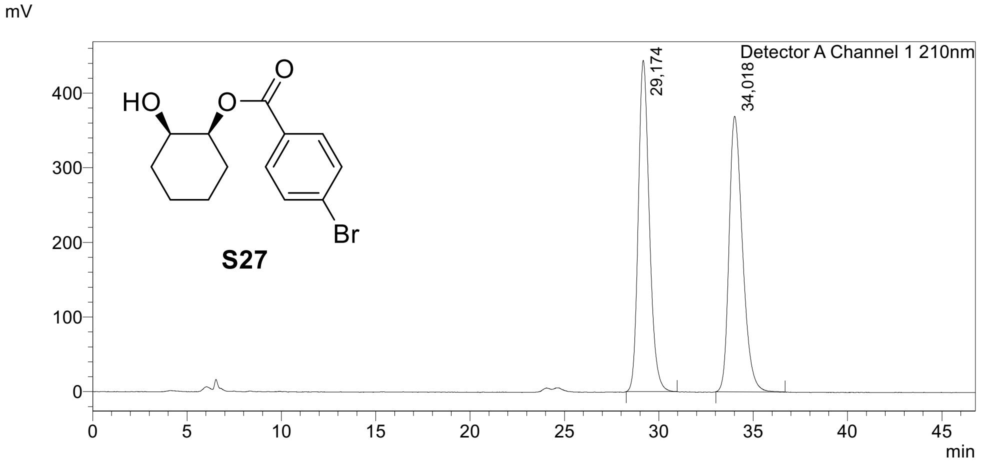

<Peak Table>

Detector A Channel 1 210nm

Peak#	Ret. Time	Area	Height	Conc.	Unit	Mark	Name
1	29,174	18069124	444025	49,665		M	
2	34,018	18313112	369363	50,335		M	
Total		36382236	813387				

C:\LabSolutions\Data\Wiesler\cm-893F1_nhept_iPrOH_95_5.lcd

HPLC chromatogram of *rac*-**S27**.

SHIMADZU LabSolutions

Analysis Report

<Sample Information>

Sample Name	: cm-910F1		
Sample ID	: 60min 0_5ml		
Data Filename	: cm-910F1_nhept_iPrOH_95_510_544.lcd		
Method Filename	: IA_CM-Desym_195nm-Heptane-iProp-99-01-60min-mGr-0_5mL.lcm		
Batch Filename	:		
Vial #	: 1-42	Sample Type	: Unknown
Injection Volume	: 10 uL		
Date Acquired	: 13.08.2021 10:03:29	Acquired by	: System Administrator
Date Processed	: 22.08.2021 10:50:53	Processed by	: System Administrator

<Chromatogram>

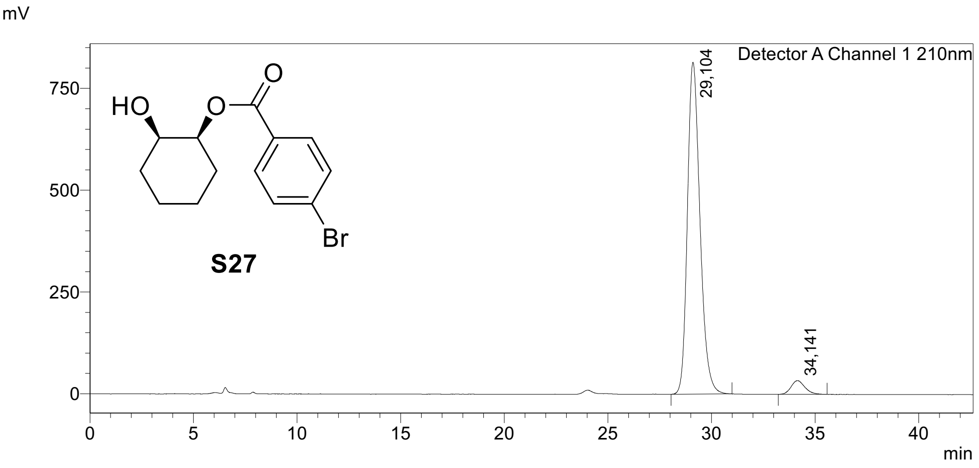

<Peak Table>

Detector A Channel 1 210nm

Peak#	Ret. Time	Area	Height	Conc.	Unit	Mark	Name
1	29,104	34336534	814846	95,644		M	
2	34,141	1563775	33657	4,356		M	
Total		35900308	848503				

C:\LabSolutions\Data\Wiesler\cm-910F1_nhept_iPrOH_95_510_544.lcd

HPLC chromatogram of **S27**.

Albert-Ludwigs-Universität Freiburg
Institut für Organische Chemie

Sample ID: ma.cm-V-833F1

Method: C:\Enterprise\Methods\HPLC2\4_Chiralcel OD-3\System2, OD-3. Hep_EtOH 99,5_0,5, 0,5mL, 22°C.met

Vial: P2-E1

Injection Volume: 5µL

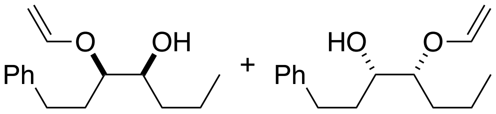

rac-**4** *rac*-**5**

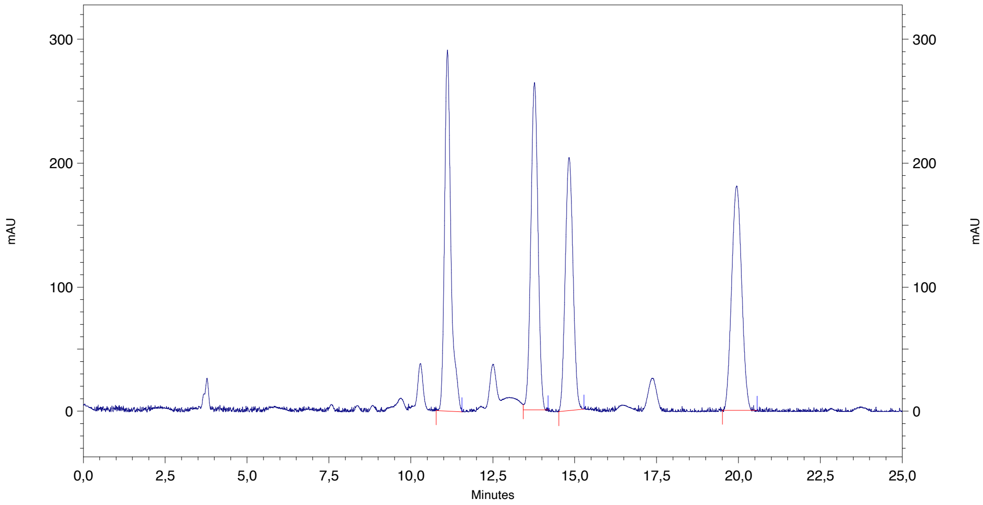

HPLC chromatogram of *rac*-**4** and *rac*-**5**.

Spectrum Max Plot
Results

Peak Number	Retention Time	Area Percent	Area
1	11,113	25,937	527753993
2	13,772	26,408	537335578
3	14,827	22,226	452241060
4	19,942	25,429	517402881
Totals		100,000	2034733512

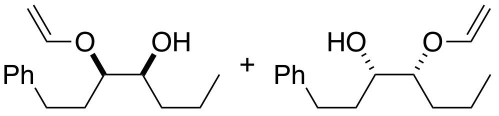

rac-**4** *rac*-**5**

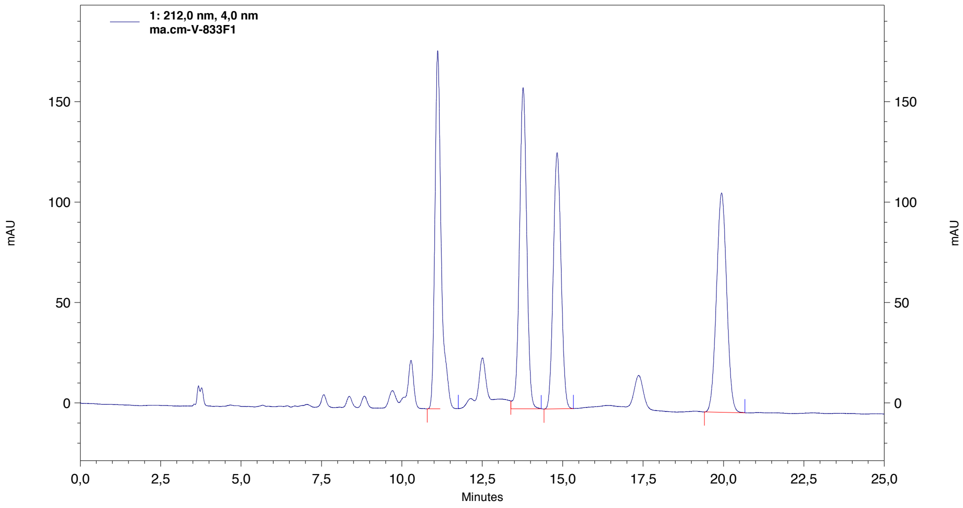

1: 212,0 nm, 4,0 nm
Results

Peak Number	Retention Time	Area Percent	Area
1	11,115	25,676	327361465
2	13,770	26,298	335291129
3	14,827	22,635	288595292
4	19,942	25,392	323741811
Totals		100,000	1274989697

HPLC chromatogram of *rac*-**4** and *rac*-**5**.

Albert-Ludwigs-Universität Freiburg
Institut für Organische Chemie

Sample ID: ma.cm-V-843

Method: C:\Enterprise\Methods\HPLC2\4_Chiralcel OD-3\System2, OD-3. Hep_EtOH 99,5_0,5, 0,5mL, 22°C.met

Vial: P2-E1

Injection Volume: 5µL

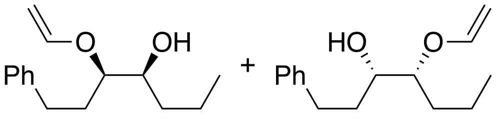

4 **5**

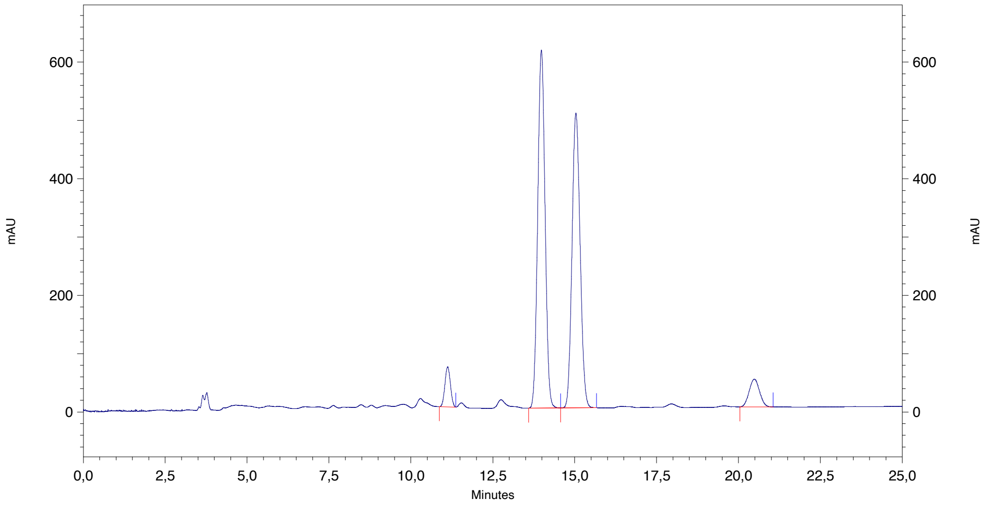

HPLC chromatogram of **4** and **5.**

Spectrum Max Plot
Results

Peak Number	Retention Time	Area Percent	Area
1	11,120	4,035	108832028
2	13,982	47,497	1281079950
3	15,035	43,232	1166040142
4	20,482	5,235	141205573
Totals		100,000	2697157693

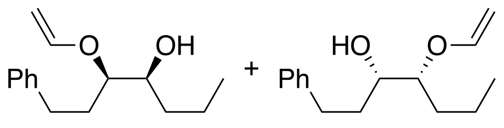

4 5

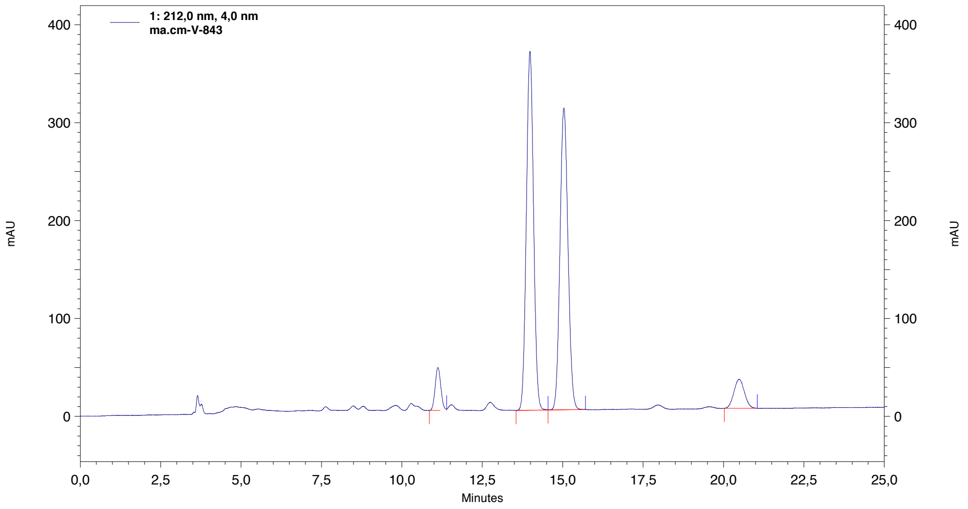

1: 212,0 nm, 4,0 nm
Results

Peak Number	Retention Time	Area Percent	Area
1	11,122	4,415	72520928
2	13,982	46,582	765104661
3	15,037	43,521	714824723
4	20,487	5,482	90039201
Totals		100,000	1642489513

HPLC chromatogram of **4** and **5.**

Albert-Ludwigs-Universität Freiburg
Institut für Organische Chemie

Sample ID: ma.cm-V-836F1

Method: C:\Enterprise\Methods\HPLC2\5_ChiralPAK AD-3\System2, AD-3, Hep_EtOH 99,7_0,3. 0,5ml, 22°C.met

Vial: P2-E3

Injection Volume: 5µL

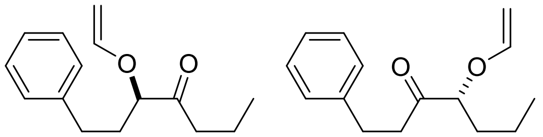

rac-**S28** *rac*-**S29**

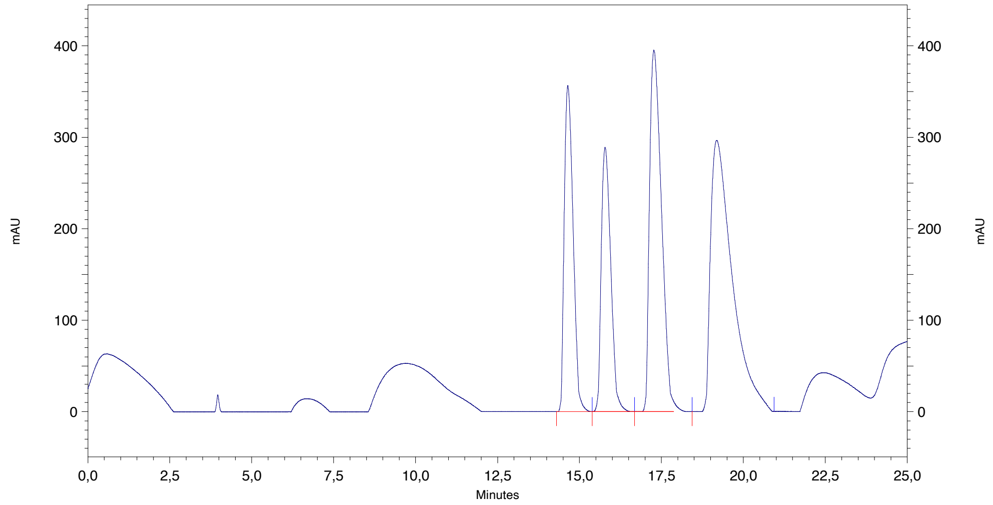

HPLC chromatogram of *rac*-**S28** and *rac*-**S29**.

Spectrum Max Plot
Results

Peak Number	Retention Time	Area Percent	Area
1	14,645	18,527	900468128
2	15,778	16,103	782649194
3	17,268	27,673	1345010164
4	19,187	37,697	1832187905
Totals		100,000	4860315391

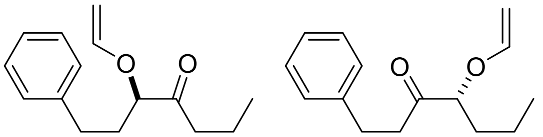

rac-**S28** *rac*-**S29**

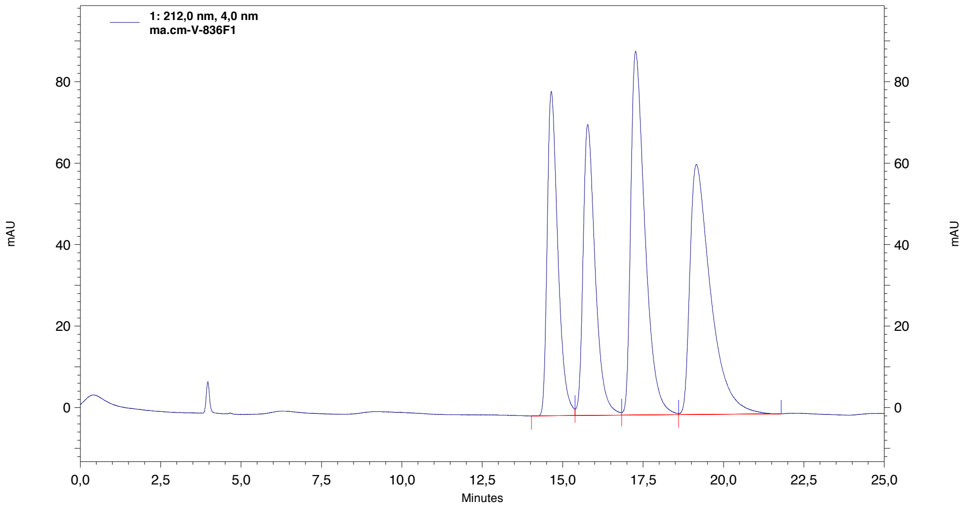

1: 212,0 nm, 4,0 nm
Results

Peak Number	Retention Time	Area Percent	Area
1	14,645	19,975	246682331
2	15,780	20,366	251519227
3	17,268	29,748	367379323
4	19,157	29,912	369404934
Totals		100,000	1234985815

HPLC chromatogram of *rac*-**S28** and *rac*-**S29**.

Albert-Ludwigs-Universität Freiburg
Institut für Organische Chemie

Sample ID: ma.cm-V-845

Method: C:\Enterprise\Methods\HPLC2\5_ChiralPAK AD-3\System2, AD-3, Hep_EtOH 99,7_0,3. 0,5ml, 22°C.met

Vial: P1-E1

Injection Volume: 5µL

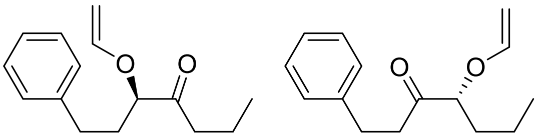

S28 **S29**

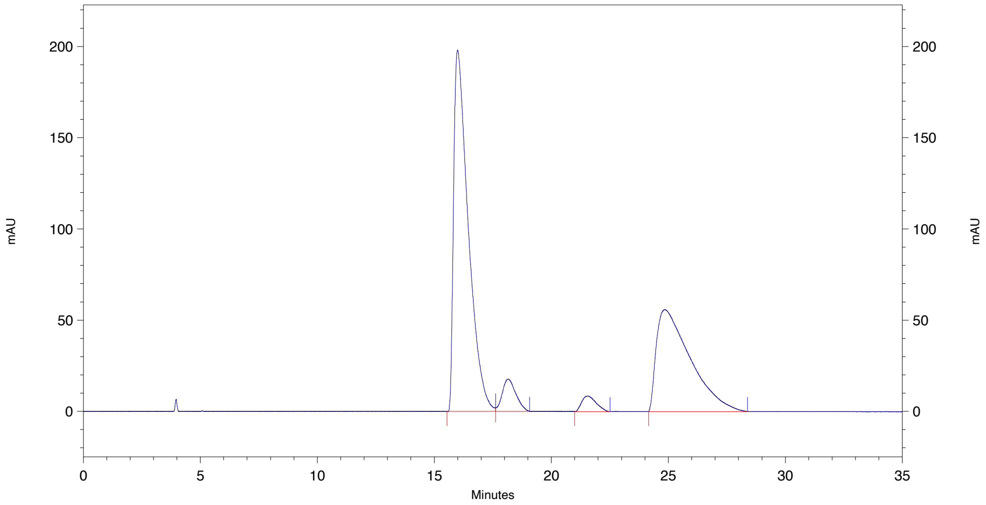

HPLC chromatogram of **S28** and **S29**.

Spectrum Max Plot
Results

Peak Number	Retention Time	Area Percent	Area
1	15,993	56,203	1154566884
2	18,145	4,673	95988586
3	21,543	2,355	48381843
4	24,853	36,769	755344978
Totals		100,000	2054282291

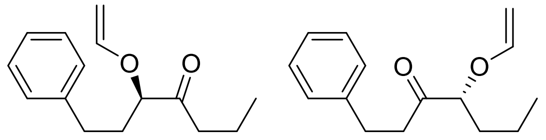

S28 **S29**

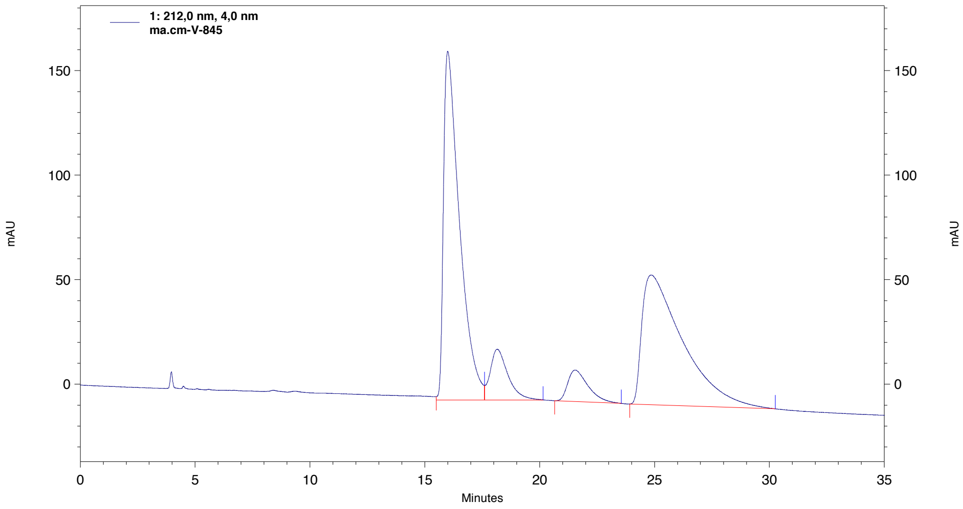

1: 212,0 nm, 4,0 nm
Results

Peak Number	Retention Time	Area Percent	Area
1	15,995	45,054	1090765615
2	18,145	7,217	174735569
3	21,545	5,140	124450930
4	24,855	42,589	1031082381
Totals		100,000	2421034495

HPLC chromatogram of **S28** and **S29**.

Albert-Ludwigs-Universität Freiburg
Institut für Organische Chemie

Sample ID: ma.cm-V-850F1

Method: C:\Enterprise\Methods\HPLC2\5_ChiralPAK AD-3\System2, AD-3, Hep_EtOH 95_5,0,5ml, 22°C.met

Vial: P2-A1

Injection Volume: 5µL

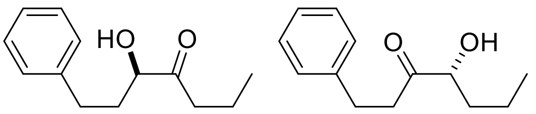

rac-**S30** *rac*-**S31**

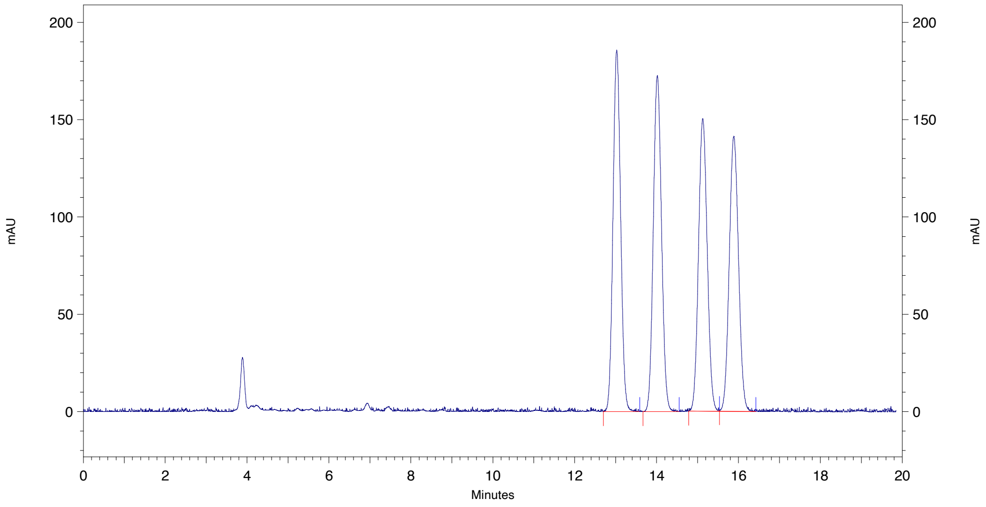

HPLC chromatogram of *rac*-**S30** and *rac*-**S31**.

Spectrum Max Plot
Results

Peak Number	Retention Time	Area Percent	Area
1	13,027	25,916	321538547
2	14,017	25,965	322145016
3	15,127	24,085	298824361
4	15,885	24,033	298179576
Totals		100,000	1240687500

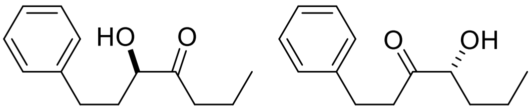

rac-**S30** *rac*-**S31**

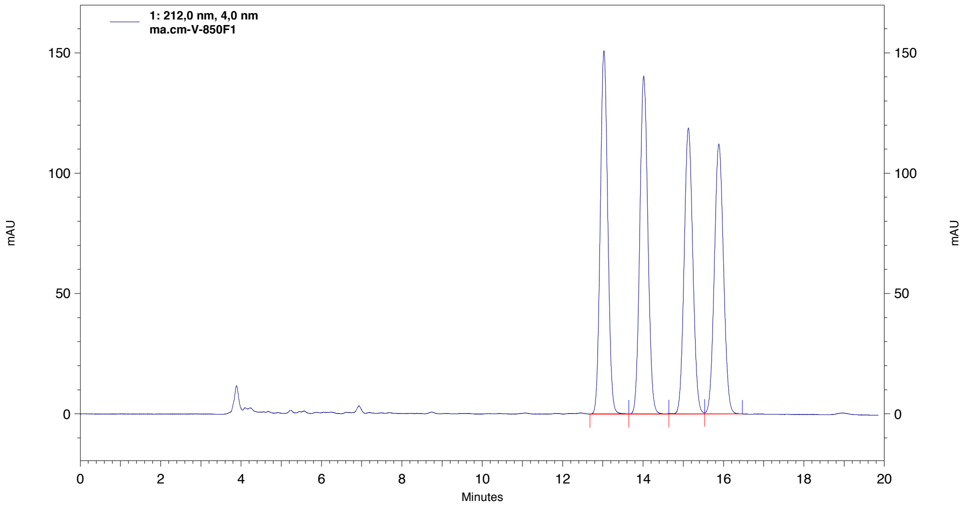

1: 212,0 nm, 4,0 nm
Results

Peak Number	Retention Time	Area Percent	Area
1	13,025	26,139	261384071
2	14,017	26,313	263123169
3	15,127	23,774	237730535
4	15,885	23,775	237741696
Totals		100,000	999979471

HPLC chromatogram of *rac*-**S30** and *rac*-**S31**.

Albert-Ludwigs-Universität Freiburg
Institut für Organische Chemie

Sample ID: ma.cm-V-856F1

Method: C:\Enterprise\Methods\HPLC2\5_ChiralPAK AD-3\System2, AD-3, Hep_EtOH 95_5,0,5ml, 22°C.met

Vial: P2-A2

Injection Volume: 5µL

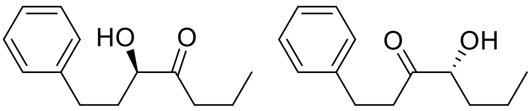

S30 **S31**

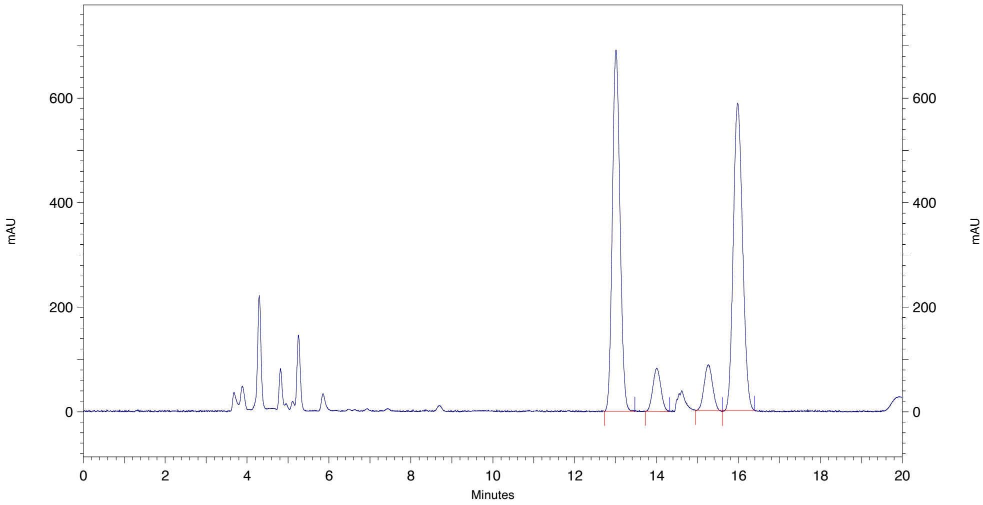

HPLC chromatogram of **S30** and **S31**.

Spectrum Max Plot
Results

Peak Number	Retention Time	Area Percent	Area
1	13,007	43,610	1203556634
2	14,005	5,464	150798203
3	15,260	5,965	164614009
4	15,978	44,962	1240866968
Totals		100,000	2759835814

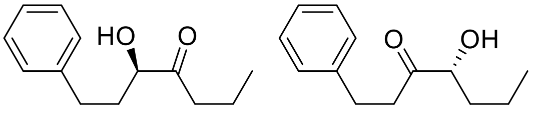

S30 **S31**

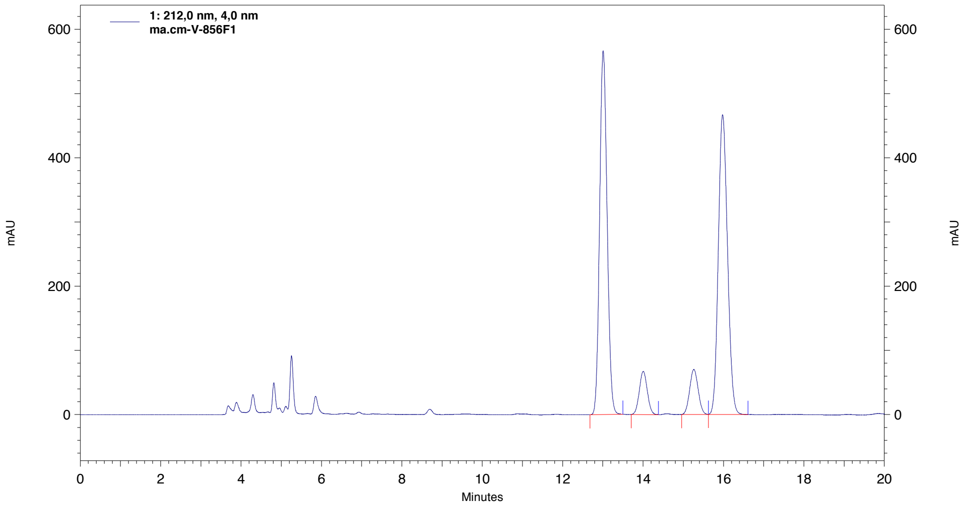

1: 212,0 nm, 4,0 nm
Results

Peak Number	Retention Time	Area Percent	Area
1	13,007	43,896	986514203
2	14,003	5,555	124834192
3	15,262	6,193	139182948
4	15,978	44,356	996864930
Totals		100,000	2247396273

HPLC chromatogram of **S30** and **S31**.

SHIMADZU LabSolutions

Analysis Report

<Sample Information>

Sample Name	: cm-898-2F1		
Sample ID	: 60min 0_5ml		
Data Filename	: cm-898-2F1_nhept_iPrOH_90_10_27.lcd		
Method Filename	: IA_CM-Desym_195nm-Heptane-iProp-99-01-60min-mGr-0_5mL.lcm		
Batch Filename	:		
Vial #	: 1-51	Sample Type	: Unknown
Injection Volume	: 10 uL		
Date Acquired	: 04.08.2021 19:16:25	Acquired by	: System Administrator
Date Processed	: 22.08.2021 10:43:48	Processed by	: System Administrator

<Chromatogram>

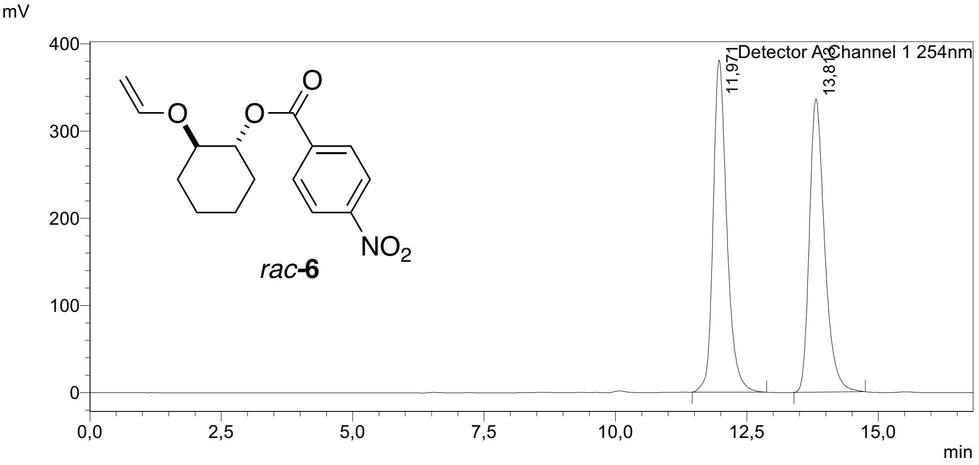

<Peak Table>

Detector A Channel 1 254nm

Peak#	Ret. Time	Area	Height	Conc.	Unit	Mark	Name
1	11,971	6909081	380603	50,445		M	
2	13,813	6787311	336232	49,555		M	
Total		13696391	716836				

C:\LabSolutions\Data\Wiesler\cm-898-2F1_nhept_iPrOH_90_10_27.lcd

HPLC chromatogram of *rac*-**6**.

SHIMADZU LabSolutions

Analysis Report

<Sample Information>

Sample Name	: cm-9082F1		
Sample ID	: 60min 0_5ml		
Data Filename	: cm-9082F1_nhept_iPrOH_90_10_20.lcd		
Method Filename	: IA_CM-Desym_195nm-Heptane-iProp-99-01-60min-mGr-0_5mL.lcm		
Batch Filename	:		
Vial #	: 1-52	Sample Type	: Unknown
Injection Volume	: 10 uL		
Date Acquired	: 22.08.2021 11:24:54	Acquired by	: System Administrator
Date Processed	: 22.08.2021 11:42:42	Processed by	: System Administrator

<Chromatogram>

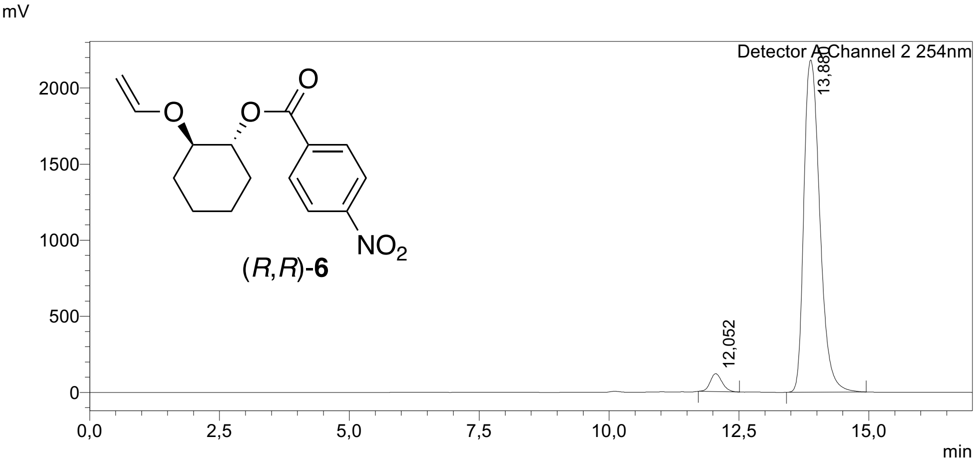

<Peak Table>

Detector A Channel 2 254nm

Peak#	Ret. Time	Area	Height	Conc.	Unit	Mark	Name
1	12,052	1864784	117772	3,841		M	
2	13,880	46679034	2182469	96,159		M	
Total		48543817	2300240				

C:\LabSolutions\Data\Wiesler\cm-9082F1_nhept_iPrOH_90_10_20.lcd

HPLC chromatogram of (*R,R*)-**6**.

SHIMADZU LabSolutions

Analysis Report

<Sample Information>

Sample Name	: cm-913		
Sample ID	: 60min 0_5ml		
Data Filename	: cm-913_nhept_iPrOH_90_10_16.lcd		
Method Filename	: IA_CM-Desym_195nm-Heptane-iProp-99-01-60min-mGr-0_5mL.lcm		
Batch Filename	:		
Vial #	: 1-4	Sample Type	: Unknown
Injection Volume	: 10 uL		
Date Acquired	: 18.08.2021 13:59:42	Acquired by	: System Administrator
Date Processed	: 18.08.2021 14:19:00	Processed by	: System Administrator

<Chromatogram>

mV

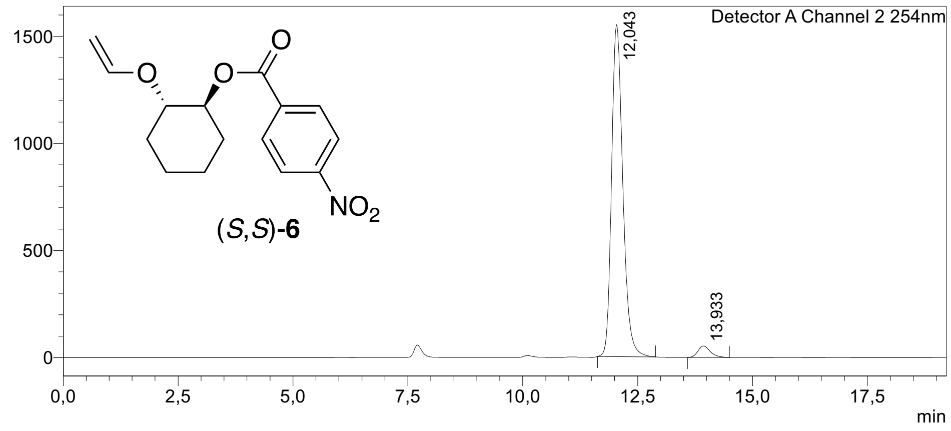

<Peak Table>

Detector A Channel 2 254nm

Peak#	Ret. Time	Area	Height	Conc.	Unit	Mark	Name
1	12,043	26426575	1549484	96,374		M	
2	13,933	994334	53417	3,626		M	
Total		27420909	1602901				

C:\LabSolutions\Data\Wiesler\cm-913_nhept_iPrOH_90_10_16.lcd

HPLC chromatogram of (*S*,*S*)-**6**.

SHIMADZU LabSolutions

Analysis Report

<Sample Information>

Sample Name	: cm-894F1		
Sample ID	: 60min 0_5ml		
Data Filename	: cm-894_nhept_iPrOH_90_10_25.lcd		
Method Filename	: IA_CM-Desym_195nm-Heptane-iProp-99-01-60min-mGr-0_5mL.lcm		
Batch Filename	:		
Vial #	: 1-21	Sample Type	: Unknown
Injection Volume	: 10 uL		
Date Acquired	: 31.07.2021 13:31:21	Acquired by	: System Administrator
Date Processed	: 31.07.2021 13:49:26	Processed by	: System Administrator

<Chromatogram>

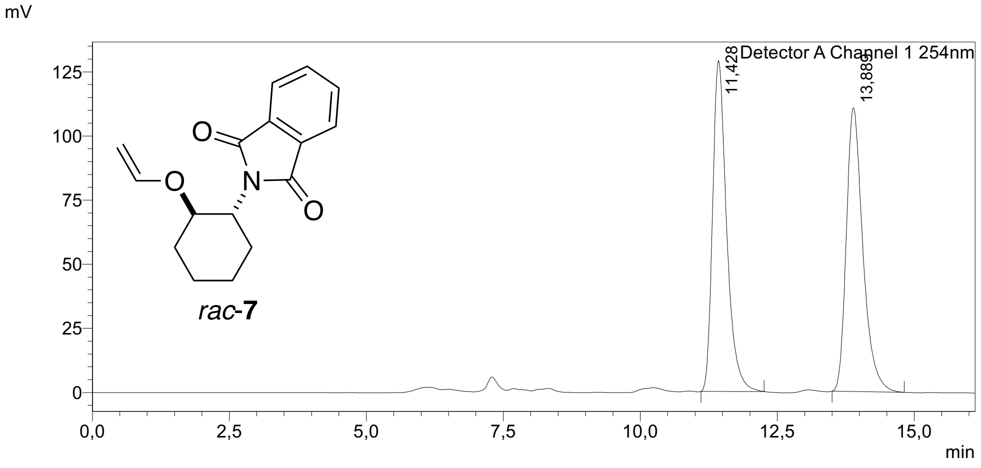

<Peak Table>

Detector A Channel 1 254nm

Peak#	Ret. Time	Area	Height	Conc.	Unit	Mark	Name
1	11,428	2297702	128951	49,687		M	
2	13,889	2326679	110651	50,313		M	
Total		4624381	239602				

C:\LabSolutions\Data\Wiesler\cm-894_nhept_iPrOH_90_10_25.lcd

HPLC chromatogram of *rac*-**7**.

Analysis Report

<Sample Information>

Sample Name	: cm-909-2F1		
Sample ID	: 60min 0_5ml		
Data Filename	: cm-909-2F1_nhept_iPrOH_90_10_8.lcd		
Method Filename	: IA_CM-Desym_195nm-Heptane-iProp-99-01-60min-mGr-0_5mL.lcm		
Batch Filename	:		
Vial #	: 1-64	Sample Type	: Unknown
Injection Volume	: 10 uL		
Date Acquired	: 16.08.2021 11:31:41	Acquired by	: System Administrator
Date Processed	: 16.08.2021 13:23:59	Processed by	: System Administrator

<Chromatogram>

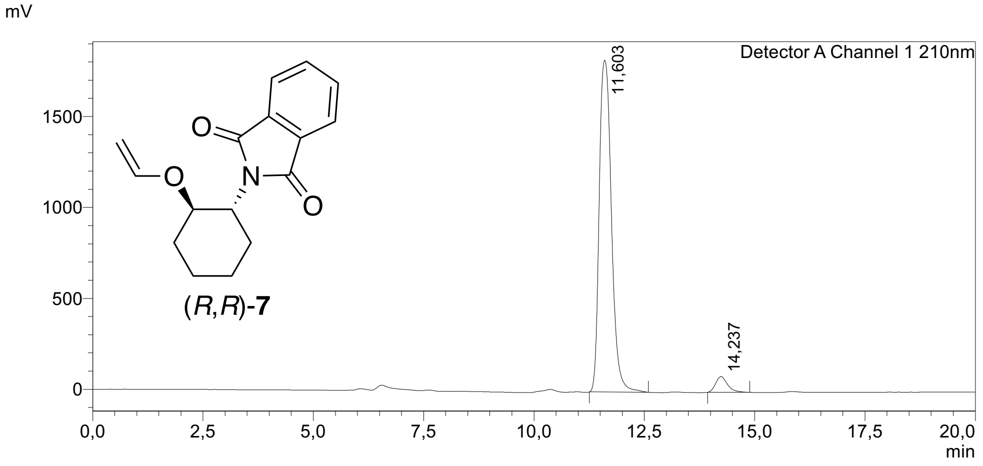

<Peak Table>

Detector A Channel 1 210nm

Peak#	Ret. Time	Area	Height	Conc.	Unit	Mark	Name
1	11,603	35181483	1824103	95,560		M	
2	14,237	1634641	86773	4,440		M	
Total		36816124	1910876				

C:\LabSolutions\Data\Wiesler\cm-909-2F1_nhept_iPrOH_90_10_8.lcd

HPLC chromatogram of (*R,R*)-**7**.

Analysis Report

<Sample Information>

Sample Name	: cm-914		
Sample ID	: 60min 0_5ml		
Data Filename	: cm-914_nhept_iPrOH_90_10_19.lcd		
Method Filename	: IA_CM-Desym_195nm-Heptane-iProp-99-01-60min-mGr-0_5mL.lcm		
Batch Filename	:		
Vial #	: 1-12	Sample Type	: Unknown
Injection Volume	: 10 uL		
Date Acquired	: 18.08.2021 18:02:28	Acquired by	: System Administrator
Date Processed	: 22.08.2021 11:26:52	Processed by	: System Administrator

<Chromatogram>

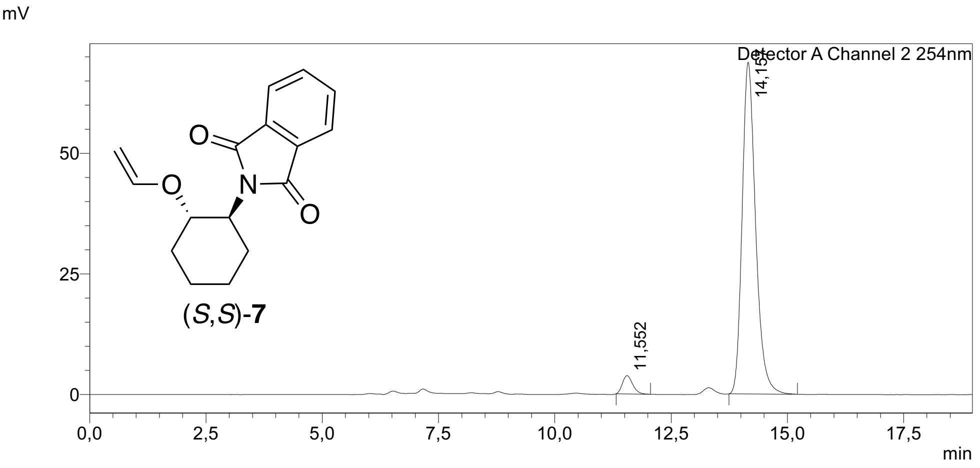

<Peak Table>

Detector A Channel 2 254nm

Peak#	Ret. Time	Area	Height	Conc.	Unit	Mark	Name
1	11,552	58304	3791	4,038		M	
2	14,157	1385750	68760	95,962		M	
Total		1444055	72552				

C:\LabSolutions\Data\Wiesler\cm-914_nhept_iPrOH_90_10_19.lcd

HPLC chromatogram of (*S,S*)-**7**.

SHIMADZU LabSolutions

Analysis Report

<Sample Information>

Sample Name	: cm-1001F1_IA_95_5		
Sample ID	: 60min 0_5ml		
Data Filename	: cm-1001F1_IA_95_5.lcd		
Method Filename	: IA_CM-Desym_195nm-Heptane-iProp-99-01-60min-mGr-0_5mL.lcm		
Batch Filename	:		
Vial #	: 1-38	Sample Type	: Unknown
Injection Volume	: 10 uL		
Date Acquired	: 07.12.2021 16:57:20	Acquired by	: System Administrator
Date Processed	: 07.12.2021 17:50:48	Processed by	: System Administrator

<Chromatogram>

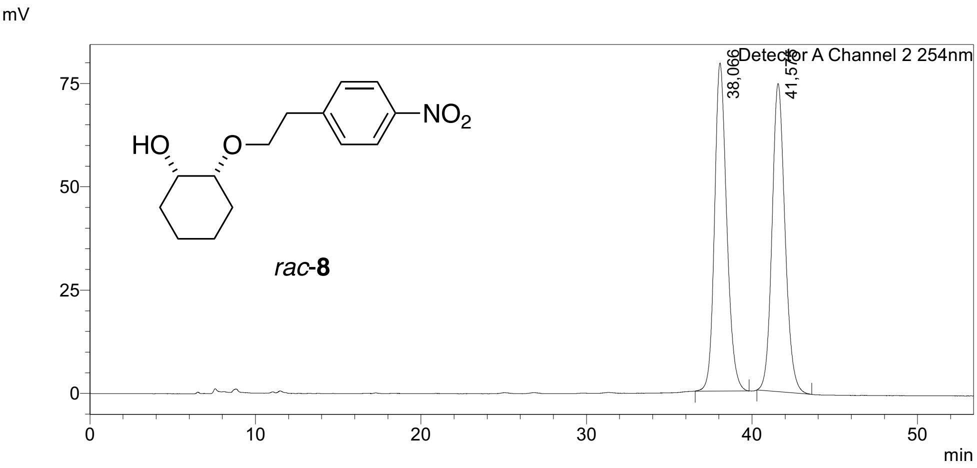

<Peak Table>

Detector A Channel 2 254nm

Peak#	Ret. Time	Area	Height	Conc.	Unit	Mark	Name
1	38,066	4056169	79340	49,965		M	
2	41,575	4061834	74571	50,035		M	
Total		8118003	153911				

C:\LabSolutions\Data\Wiesler\cm-1001F1_IA_95_5.lcd

HPLC chromatogram of *rac*-**8**.

SHIMADZU LabSolutions

Analysis Report

<Sample Information>

Sample Name	: cm-1002F1_IA_95_5		
Sample ID	: 60min 0_5ml		
Data Filename	: cm-1002F1_IA_95_6.lcd		
Method Filename	: IA_CM-Desym_195nm-Heptane-iProp-99-01-60min-mGr-0_5mL.lcm		
Batch Filename	:		
Vial #	: 1-39	Sample Type	: Unknown
Injection Volume	: 10 uL		
Date Acquired	: 07.12.2021 17:51:45	Acquired by	: System Administrator
Date Processed	: 07.12.2021 18:51:47	Processed by	: System Administrator

<Chromatogram>

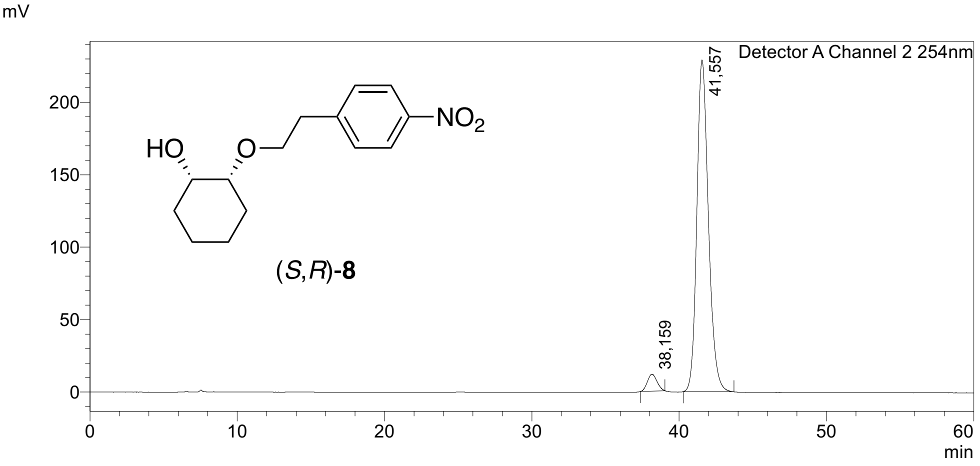

<Peak Table>

Detector A Channel 2 254nm

Peak#	Ret. Time	Area	Height	Conc.	Unit	Mark	Name
1	38,159	531890	11714	4,067		M	
2	41,557	12547475	228962	95,933		M	
Total		13079365	240676				

C:\LabSolutions\Data\Wiesler\cm-1002F1_IA_95_6.lcd

HPLC chromatogram of (*S*,*R*)-**8**.

SHIMADZU LabSolutions

Analysis Report

<Sample Information>

Sample Name	: cm-V-870F3		
Sample ID	: 60min 0_5ml		
Data Filename	: cm-V-870F3 nhept_iproh99_3.lcd		
Method Filename	: IA_CM-Desym_195nm-Heptane-iProp-99-01-60min-mGr-0_5mL.lcm		
Batch Filename	:		
Vial #	: 1-26	Sample Type	: Unknown
Injection Volume	: 10 uL		
Date Acquired	: 06.04.2021 16:31:12	Acquired by	: System Administrator
Date Processed	: 06.04.2021 17:36:11	Processed by	: System Administrator

<Chromatogram>

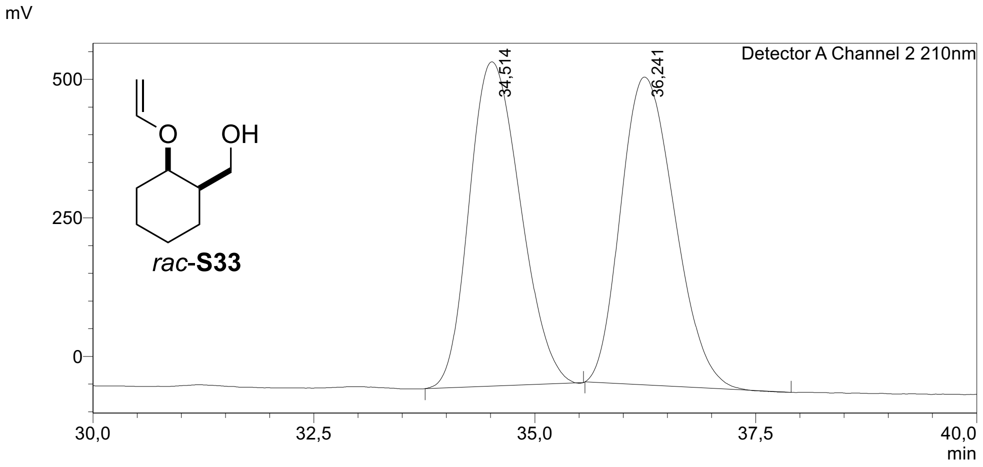

<Peak Table>

Detector A Channel 2 210nm

Peak#	Ret. Time	Area	Height	Conc.	Unit	Mark	Name
1	34,514	23527402	585205	49,948		M	
2	36,241	23576685	555587	50,052		M	
Total		47104087	1140793				

C:\LabSolutions\Data\Wiesler\cm-V-870F3 nhept_iproh99_3.lcd

HPLC chromatogram of *rac*-**S33**.

SHIMADZU LabSolutions

Analysis Report

<Sample Information>

Sample Name	: cm-V-871F2		
Sample ID	: 60min 0_5ml		
Data Filename	: cm-V-871F2 nhept_iproh99_1_0_5.lcd		
Method Filename	: IA_CM-Desym_195nm-Heptane-iProp-99-01-60min-mGr-0_5mL.lcm		
Batch Filename	:		
Vial #	: 1-24	Sample Type	: Unknown
Injection Volume	: 20 uL		
Date Acquired	: 07.04.2021 11:21:34	Acquired by	: System Administrator
Date Processed	: 10.12.2021 16:20:58	Processed by	: System Administrator

<Chromatogram>

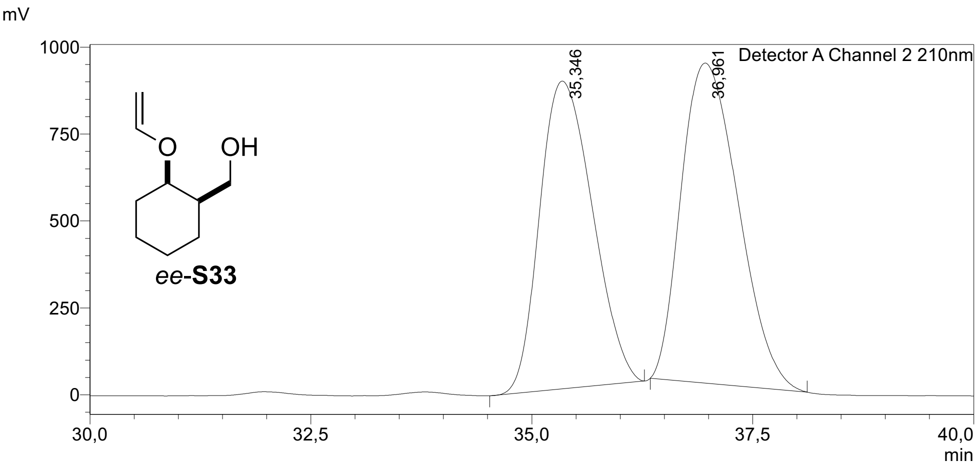

<Peak Table>

Detector A Channel 2 210nm

Peak#	Ret. Time	Area	Height	Conc.	Unit	Mark	Name
1	35,346	36823344	884694	47,038		M	
2	36,961	41461732	920064	52,962		M	
Total		78285076	1804758				

C:\LabSolutions\Data\Wiesler\cm-V-871F2 nhept_iproh99_1_0_5.lcd

HPLC chromatogram of **S33** (*ee*).

Data File D:\AK_BREIT\DATA\CM\DEF_GC 2021-04-06 17-27-09\CM-V-870-F2B.D
Sample Name: cm-V-870-F2

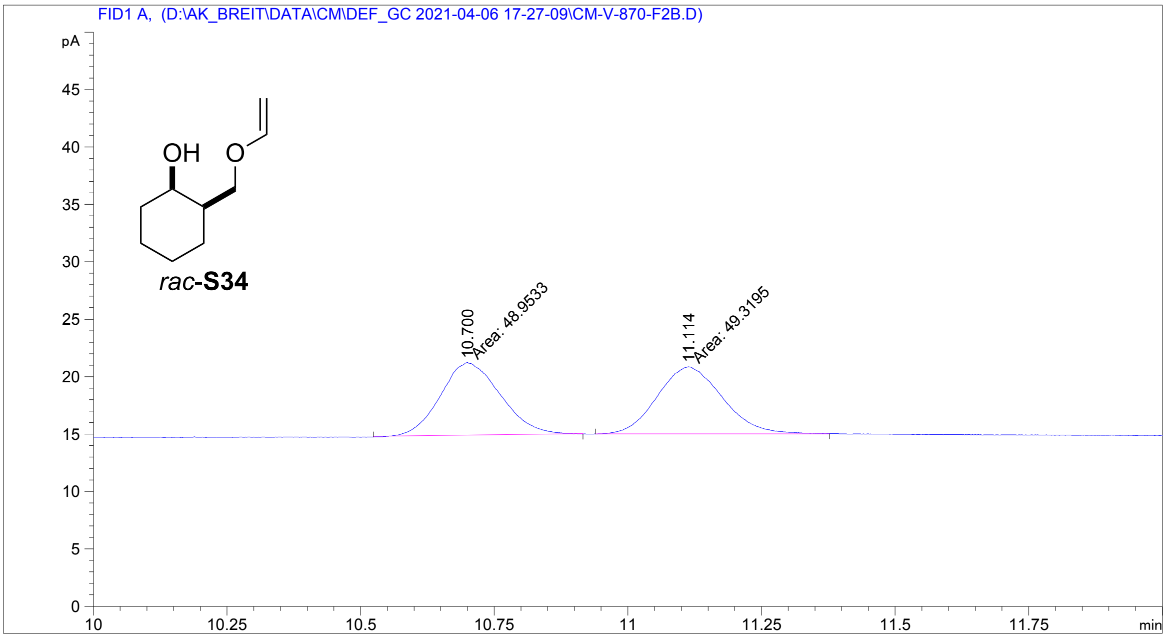

===

Area Percent Report

===

Sorted By : Signal
Multiplier : 1.0000
Dilution : 1.0000
Do not use Multiplier & Dilution Factor with ISTDs

Signal 1: FID1 A,

Peak #	RetTime [min]	Type	Width [min]	Area [pA*s]	Height [pA]	Area %
1	10.700	MM	0.1291	48.95334	6.31740	49.81373
2	11.114	MM	0.1407	49.31945	5.84301	50.18627

Totals : 98.27279 12.16041

===

*** End of Report ***

GC chromatogram of *rac*-**S34**.

Data File D:\AK_BREIT\DATA\CM\DEF_GC 2021-04-07 10-48-12\CM-V-870-F1.D
Sample Name: cm-V-871F1

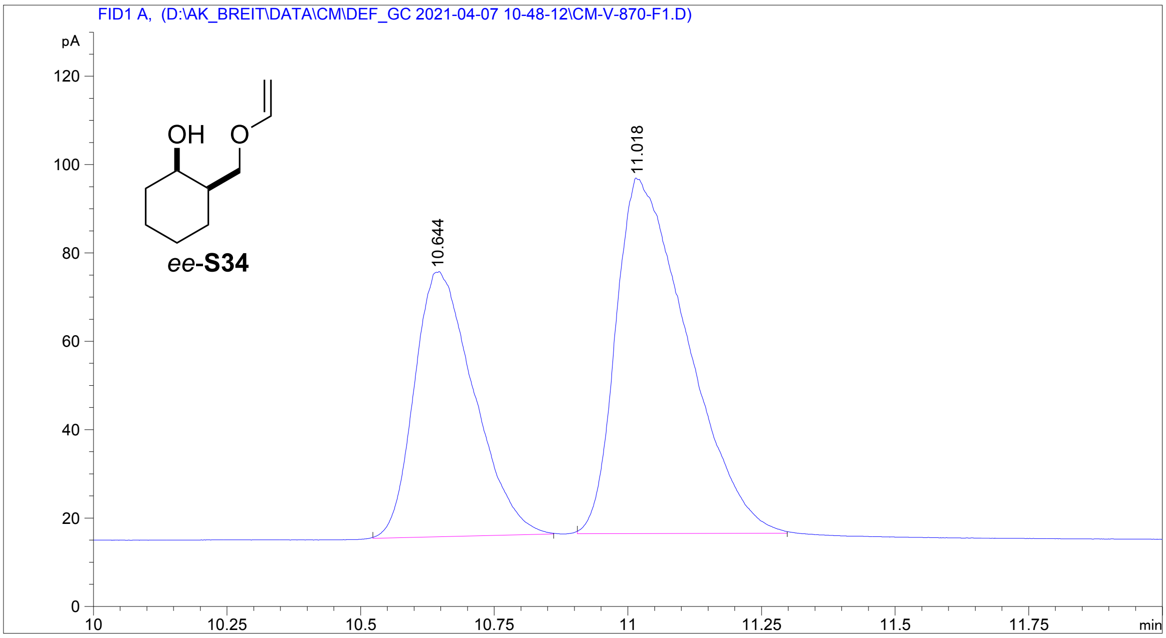

Area Percent Report

Sorted By : Signal
Multiplier : 1.0000
Dilution : 1.0000
Do not use Multiplier & Dilution Factor with ISTDs

Signal 1: FID1 A,

Peak #	RetTime [min]	Type	Width [min]	Area [pA*s]	Height [pA]	Area %
1	10.644	BB	0.1048	465.98608	59.89370	38.33361
2	11.018	BB	0.1190	749.62109	80.22060	61.66639

Totals : 1215.60718 140.11430

*** End of Report ***

GC chromatogram of **S34** (*ee*).

Albert-Ludwigs-Universität Freiburg
Institut für Organische Chemie

Sample ID: ma.cm-IV-804racF2

Method: C:\Enterprise\Methods\HPLC2\4_Chiralcel OD-3\System2, OD-3. Hep_IPA 99_1, 0,5mL,22°C.met

Vial: P1-B5

Injection Volume: 5µL

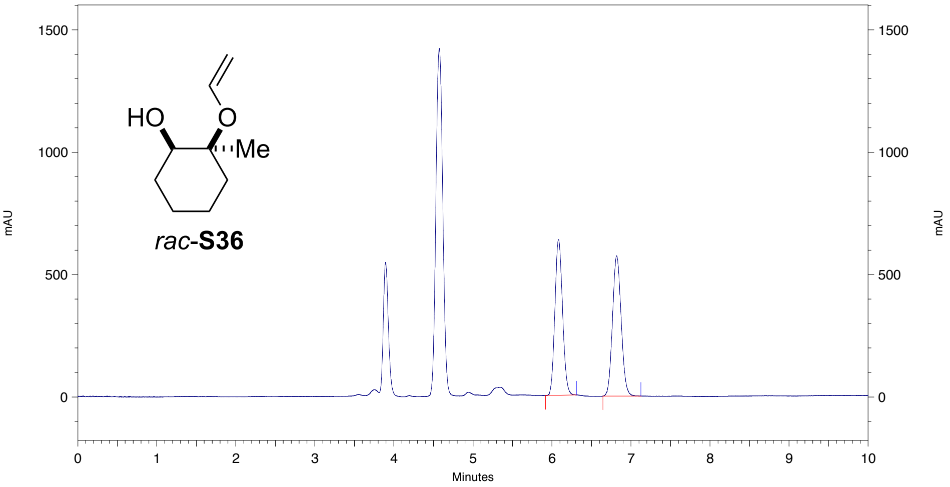

Spectrum Max Plot
Results

Peak Number	Retention Time	Area Percent	Area
1	6,083	49,462	574936217
2	6,818	50,538	587448893
Totals		100,000	1162385110

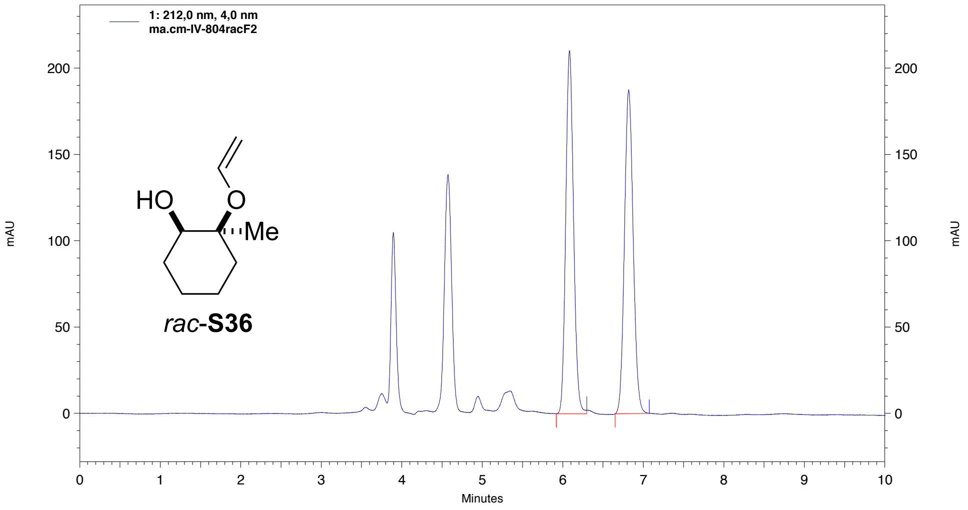

1: 212,0 nm, 4,0 nm
Results

Peak Number	Retention Time	Area Percent	Area
1	6,082	49,985	189912364
2	6,818	50,015	190024727
Totals		100,000	379937091

HPLC chromatogram of *rac*-**S36**.

Albert-Ludwigs-Universität Freiburg
Institut für Organische Chemie

Sample ID: ma.cm-IV-804eeF2

Method: C:\Enterprise\Methods\HPLC2\4_Chiralcel OD-3\System2, OD-3. Hep_IPA 99_1, 0,5mL,22°C.met

Vial: P1-B5

Injection Volume: 5µL

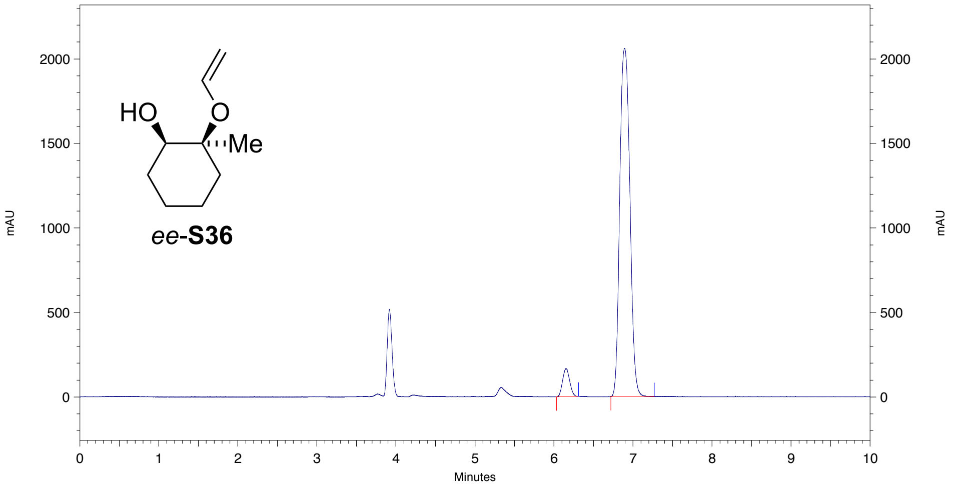

Spectrum Max Plot
Results

Peak Number	Retention Time	Area Percent	Area
1	6,152	5,374	139438019
2	6,894	94,626	2455405809
Totals		100,000	2594843828

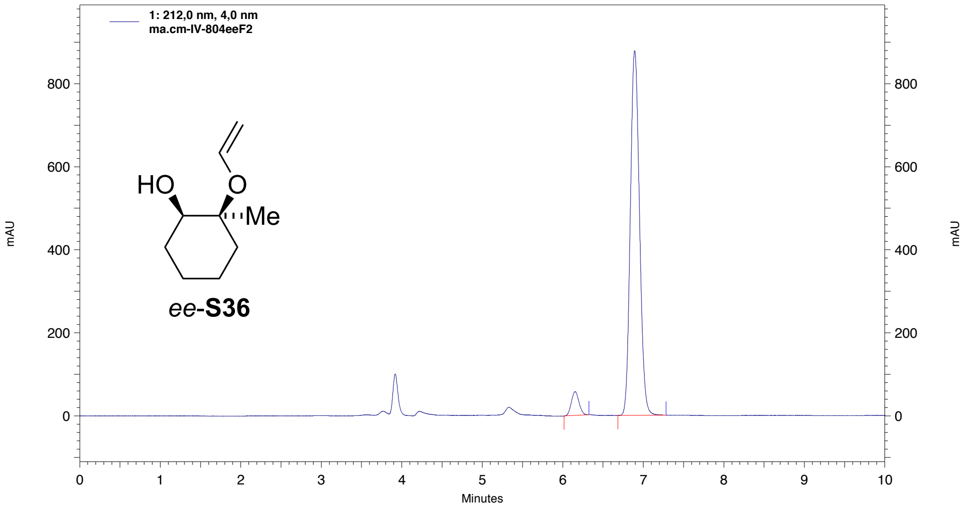

1: 212,0 nm, 4,0 nm
Results

Peak Number	Retention Time	Area Percent	Area
1	6,152	5,186	50879648
2	6,893	94,814	930250091
Totals		100,000	981129739

HPLC chromatogram of **S36** (*ee*).

Albert-Ludwigs-Universität Freiburg
Institut für Organische Chemie

Sample ID: ma.cm-IV-804racF1

Method: C:\Enterprise\Methods\HPLC2\5_ChiralPAK AD-3\System2, AD-3, Hep_IPA 99,5_0,5, 0,5ml, 22°C.met

Vial: P1-B5

Injection Volume: 5µL

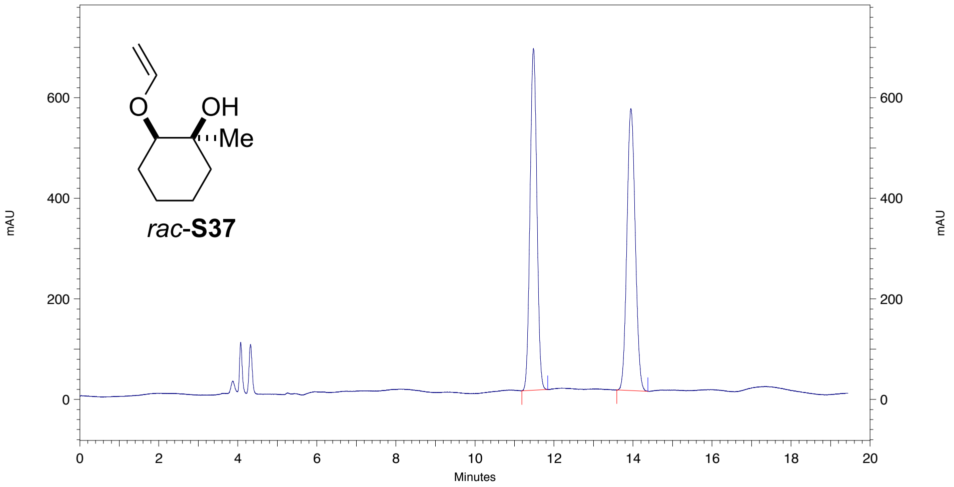

Spectrum Max Plot
Results

Peak Number	Retention Time	Area Percent	Area
1	11,480	49,821	1097046939
2	13,947	50,179	1104919738
Totals		100,000	2201966677

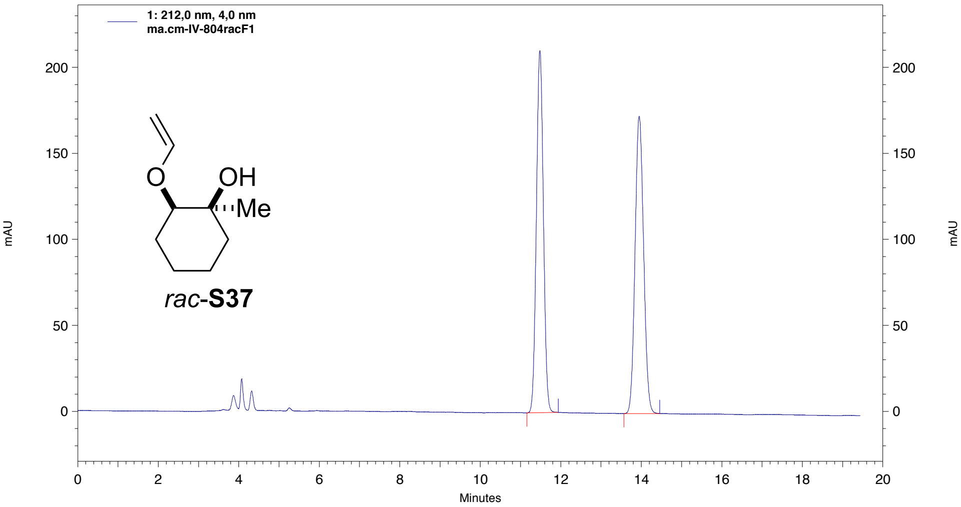

1: 212,0 nm, 4,0 nm
Results

Peak Number	Retention Time	Area Percent	Area
1	11,480	49,872	341011053
2	13,947	50,128	342761930
Totals		100,000	683772983

HPLC chromatogram of *rac*-**S37**.

Albert-Ludwigs-Universität Freiburg
Institut für Organische Chemie

Sample ID: ma.cm-IV-804eeF1

Method: C:\Enterprise\Methods\HPLC2\5_ChiralPAK AD-3\System2, AD-3, Hep_IPA 99,5_0,5, 0,5ml, 22°C.met

Vial: P1-B5

Injection Volume: 5µL

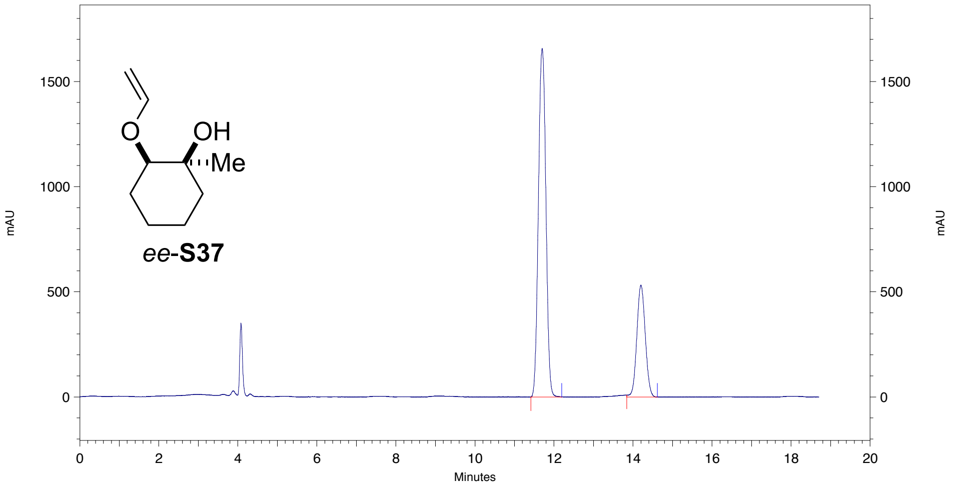

Spectrum Max Plot
Results

Peak Number	Retention Time	Area Percent	Area
1	11,702	73,326	2924524074
2	14,200	26,674	1063873845
Totals		100,000	3988397919

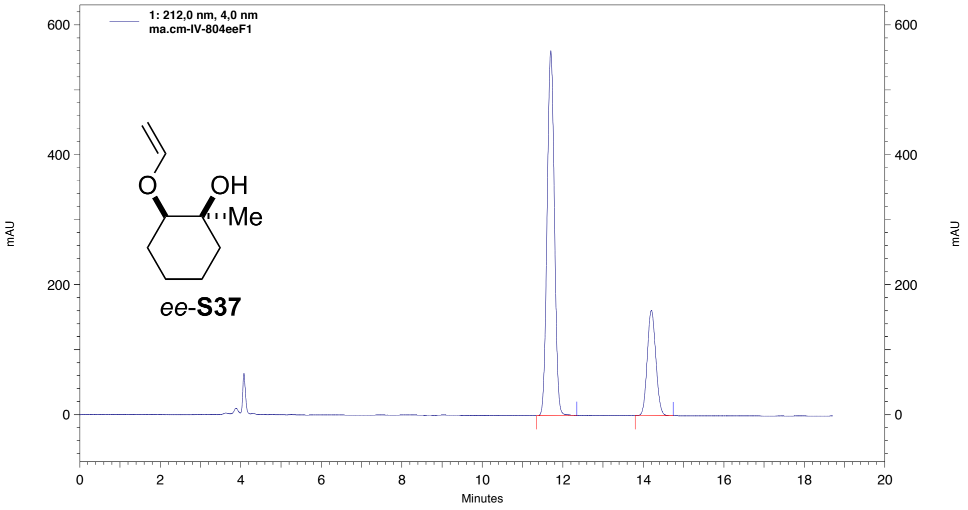

1: 212,0 nm, 4,0 nm
Results

Peak Number	Retention Time	Area Percent	Area
1	11,702	74,713	949088479
2	14,200	25,287	321223430
Totals		100,000	1270311909

HPLC chromatogram of **S37** (*ee*).

X-Ray Analysis Report for Compound S12

Structure Tables

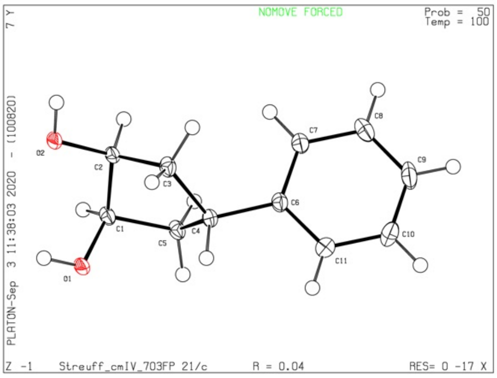

The compound was crystallized from toluene at 23 °C. The data for Streuff_cmIV_703FI were collected from a shock-cooled single crystal at 100(2) K on a Bruker D8 VENTURE dual wavelength Mo/Cu three-circle diffractometer with a microfocus sealed X-ray tube using mirror optics as monochromator and a Bruker PHOTON III detector. The diffractometer was equipped with an Oxford Cryostream 800 low temperature device and used MoK_α radiation (λ = 0.71073 Å). All data were integrated with SAINT and a multi-scan absorption correction using SADABS was applied.[1,2] The structure were solved by direct methods using SHELXT and refined by full-matrix least-squares methods against F^2 by SHELXL-2018/3.[3,4] All non-hydrogen atoms were refined with anisotropic displacement parameters. The hydrogen atoms were refined isotropically on calculated positions using a riding model with their U_{iso} values constrained to 1.5 times the U_{eq} of their pivot atoms for terminal sp^3 carbon atoms and 1.2 times for all other carbon atoms. Crystallographic data (including structure factors) for the structures reported in this paper have been deposited with the Cambridge Crystallographic Data Centre.[5] CCDC 2027182 contain the supplementary crystallographic data for this paper. Copies of the data can be obtained free of charge from the Cambridge Crystallographic Data Centre via www.ccdc.cam.ac.uk/structures. This report and the CIF file were generated using FinalCif.[6]

Table 1. Crystal data and structure refinement for Streuff_cmIV_703FI

CCDC number	2027182
Empirical formula	$C_{11}H_{14}O_2$
Formula weight	178.22
Temperature [K]	100(2)
Crystal system	monoclinic
Space group (number)	$P2_1/c$ (14)
a [Å]	18.811(3)
b [Å]	5.4771(10)
c [Å]	9.2189(16)
α [Å]	90
β [Å]	97.513(6)
γ [Å]	90
Volume [Å^3]	941.7(3)
Z	4
ρ_{calc} [g/cm^3]	1.257
μ [mm^{-1}]	0.085
F(000)	384
Crystal size [mm^3]	0.320×0.170×0.160
Crystal colour	colourless
Crystal shape	block
Radiation	MoK_α (λ=0.71073 Å)
2Θ range [°]	2.18 to 55.84 (0.76 Å)
Index ranges	-24 ≤ h ≤ 24<br>-7 ≤ k ≤ 7<br>-12 ≤ l ≤ 12
Reflections collected	19228
Independent reflections	2235<br>R_{int} = 0.0333<br>R_{sigma} = 0.0164
Completeness to θ = 25.242°	99.7 %
Data / Restraints / Parameters	2235/0/122
Goodness-of-fit on F^2	1.089
Final *R* indexes [$I \geq 2\sigma(I)$]	R_1 = 0.0388<br>wR_2 = 0.1064
Final *R* indexes [all data]	R_1 = 0.0416<br>wR_2 = 0.1091
Largest peak/hole [eÅ^3]	0.40/-0.19

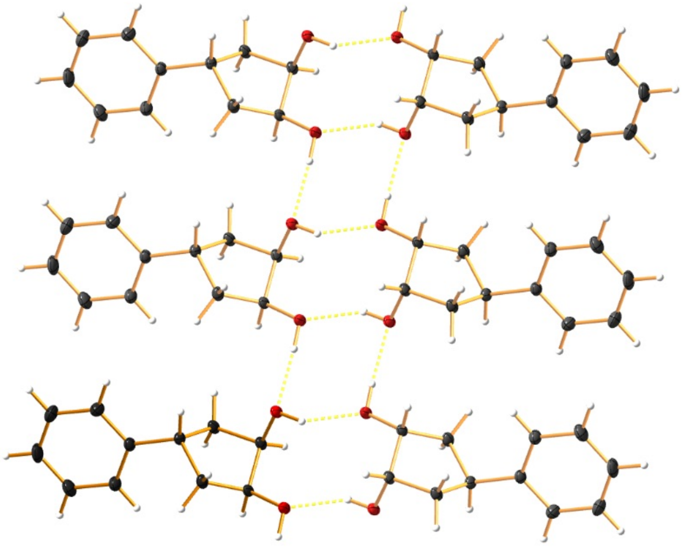

Table 2. Atomic coordinates and U_{eq} [Å^2] for Streuff_cmIV_703FI

Atom	*x*	*y*	*z*	U_{eq}
O1	0.57944(4)	0.24962(14)	0.46210(9)	0.01626(19)
H1A	0.5401(9)	0.298(2)	0.4862(12)	0.024
C1	0.59612(5)	0.3885(2)	0.33887(11)	0.0142(2)
H1	0.558341	0.371884	0.252445	0.017
O2	0.55518(4)	0.75425(15)	0.45443(10)	0.0184(2)
H2A	0.5608(5)	0.911(4)	0.4630(16)	0.028
C2	0.61066(5)	0.65489(19)	0.38138(12)	0.0136(2)
H2	0.614430	0.752413	0.291005	0.016
C5	0.66945(6)	0.3095(2)	0.30412(12)	0.0155(2)
H5A	0.678753	0.375230	0.208357	0.019
H5AB	0.673109	0.129241	0.301947	0.019
C4	0.72292(5)	0.4176(2)	0.43008(11)	0.0143(2)
H4	0.726789	0.300012	0.513729	0.017
C3	0.68416(6)	0.6501(2)	0.47621(12)	0.0160(2)
H3A	0.678290	0.642700	0.581286	0.019
H3AB	0.712011	0.798263	0.458920	0.019
C9	0.93530(6)	0.5140(3)	0.30990(15)	0.0266(3)
H9	0.982021	0.532926	0.282894	0.032
C8	0.88101(6)	0.6728(2)	0.25591(14)	0.0235(3)
H8	0.890455	0.801574	0.192075	0.028
C7	0.81254(6)	0.6438(2)	0.29524(13)	0.0187(2)
H7	0.775536	0.753599	0.257850	0.022
C6	0.79740(6)	0.4556(2)	0.38882(12)	0.0150(2)
C10	0.92133(7)	0.3274(3)	0.40332(16)	0.0279(3)
H10	0.958665	0.219045	0.441028	0.034
C11	0.85274(6)	0.2977(2)	0.44237(14)	0.0218(3)
H11	0.843631	0.168499	0.506162	0.026

U_{eq} is defined as 1/3 of the trace of the orthogonalized U_{ij} tensor.

Table 3. Bond lengths and angles for Streuff_cmIV_703FI

Atom–Atom	Length [Å]
O1–C1	1.4355(13)
O1–H1A	0.843(17)
C1–C5	1.5192(14)
C1–C2	1.5263(15)
C1–H1	1.0000
O2–C2	1.4221(12)
O2–H2A	0.865(19)
C2–C3	1.5363(15)
C2–H2	1.0000
C5–C4	1.5510(15)
C5–H5A	0.9900

C5–H5AB	0.9900
C4–C6	1.5133(14)
C4–C3	1.5543(15)
C4–H4	1.0000
C3–H3A	0.9900
C3–H3AB	0.9900
C9–C10	1.383(2)
C9–C8	1.3838(19)
C9–H9	0.9500
C8–C7	1.3920(15)
C8–H8	0.9500
C7–C6	1.3970(16)
C7–H7	0.9500
C6–C11	1.3933(16)
C10–C11	1.3936(17)
C10–H10	0.9500
C11–H11	0.9500
**Atom–Atom–Atom**	**Angle [°]**
C1–O1–H1A	109.5
O1–C1–C5	108.33(9)
O1–C1–C2	110.71(8)
C5–C1–C2	100.91(8)
O1–C1–H1	112.1
C5–C1–H1	112.1
C2–C1–H1	112.1
C2–O2–H2A	109.5
O2–C2–C1	111.68(8)
O2–C2–C3	113.49(9)
C1–C2–C3	104.34(8)
O2–C2–H2	109.1
C1–C2–H2	109.1
C3–C2–H2	109.1
C1–C5–C4	104.55(8)
C1–C5–H5A	110.8
C4–C5–H5A	110.8
C1–C5–H5AB	110.8
C4–C5–H5AB	110.8
H5A–C5–H5AB	108.9
C6–C4–C5	113.30(8)
C6–C4–C3	116.03(9)
C5–C4–C3	103.66(8)
C6–C4–H4	107.8
C5–C4–H4	107.8
C3–C4–H4	107.8
C2–C3–C4	106.13(8)
C2–C3–H3A	110.5
C4–C3–H3A	110.5
C2–C3–H3AB	110.5
C4–C3–H3AB	110.5
H3A–C3–H3AB	108.7
C10–C9–C8	119.80(11)
C10–C9–H9	120.1
C8–C9–H9	120.1
C9–C8–C7	119.92(12)
C9–C8–H8	120.0
C7–C8–H8	120.0
C8–C7–C6	121.03(11)
C8–C7–H7	119.5
C6–C7–H7	119.5
C11–C6–C7	118.26(10)
C11–C6–C4	119.92(10)
C7–C6–C4	121.80(10)
C9–C10–C11	120.30(12)
C9–C10–H10	119.8
C11–C10–H10	119.8
C6–C11–C10	120.67(12)
C6–C11–H11	119.7
C10–C11–H11	119.7

Table 4. Torsion angles for Streuff_cmIV_703FI

**Atom–Atom–Atom–Atom**	**Torsion Angle [°]**
O1–C1–C2–O2	-50.53(11)
C5–C1–C2–O2	-165.06(9)
O1–C1–C2–C3	72.44(10)
C5–C1–C2–C3	-42.09(10)
O1–C1–C5–C4	-72.14(10)
C2–C1–C5–C4	44.17(10)
C1–C5–C4–C6	-155.71(9)
C1–C5–C4–C3	-29.10(10)
O2–C2–C3–C4	146.18(9)
C1–C2–C3–C4	24.39(10)
C6–C4–C3–C2	127.59(9)
C5–C4–C3–C2	2.73(11)
C10–C9–C8–C7	-0.3(2)
C9–C8–C7–C6	-0.07(19)
C8–C7–C6–C11	0.20(18)
C8–C7–C6–C4	-178.58(11)
C5–C4–C6–C11	-105.48(12)
C3–C4–C6–C11	134.76(11)
C5–C4–C6–C7	73.28(14)
C3–C4–C6–C7	-46.48(14)
C8–C9–C10–C11	0.5(2)
C7–C6–C11–C10	0.01(18)
C4–C6–C11–C10	178.81(11)
C9–C10–C11–C6	-0.4(2)

Table 5. Hydrogen bonds for Streuff_cmIV_703FI

D–H···A [Å]	d(D–H) [Å]	d(H···A) [Å]	d(D···A) [Å]	<(DHA) [°]
O1–H1A···O2#1	0.84	1.96	2.7413(12)	153.5
O2–H2A···O1#2	0.87	1.89	2.7507(12)	173.0

Symmetry transformations used to generate equivalent atoms:
#1: 1-X, 1-Y, 1-Z; #2: +X, 1+Y, +Z;

Bibliography

[1] Bruker, *SAINT*, *V8.40A*, Bruker AXS Inc., Madison, Wisconsin, USA.

[2] Bruker, *SADABS*, *2016/2*, Bruker AXS Inc., Madison, Wisconsin, USA.

[3] G. M. Sheldrick, *Acta Cryst.* **2015**, *A71*, 3–8, doi:10.1107/S2053273314026370.

[4] G. M. Sheldrick, *Acta Cryst.* **2015**, *C71*, 3–8, doi:10.1107/S2053229614024218.

[5] C. R. Groom, I. J. Bruno, M. P. Lightfoot, S. C. Ward, *Acta Cryst.* **2016**, *B72*, 171–179, doi:10.1107/S2052520616003954.

[6] D. Kratzert, *FinalCif*, *V68*, https://www.xs3.uni-freiburg.de/research/finalcif.

X-Ray Analysis Report for Compound S27

Table 1 Crystal data and structure refinement for S27.

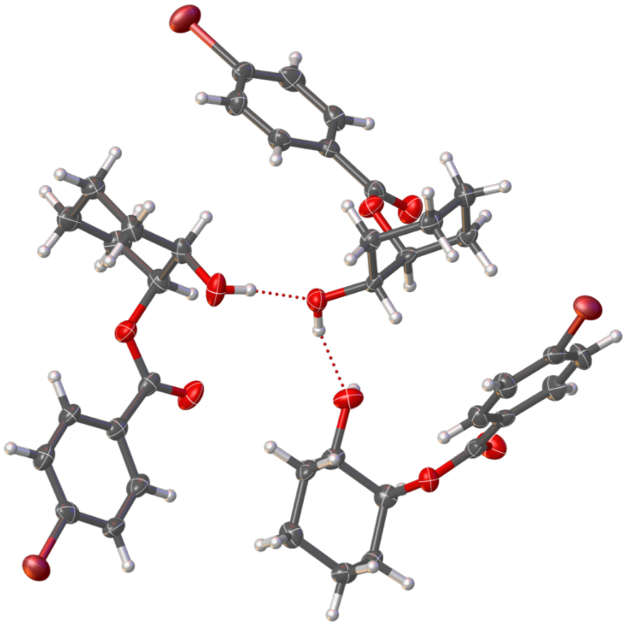

Identification code	AO_JS_CM_910_1_0m (**S27)**
CCDC	2106814
Empirical formula	$C_{13}H_{15}BrO_3$
Formula weight	299.16
Temperature/K	150.15
Crystal system	orthorhombic
Space group	$P2_12_12_1$
a/Å	6.355(2)
b/Å	21.957(8)
c/Å	27.613(10)
α/°	90
β/°	90
γ/°	90
Volume/Å^3	3853(2)
Z	12
ρ_{calc}g/cm^3	1.547
μ/mm^{-1}	3.194
F(000)	1824.0
Crystal size/mm^3	0.2 × 0.18 × 0.04
Radiation	MoKα (λ = 0.71073)
2Θ range for data collection/°	2.37 to 54.336
Index ranges	-8 ≤ h ≤ 8, -28 ≤ k ≤ 28, -35 ≤ l ≤ 35
Reflections collected	70178
Independent reflections	8507 [R_{int} = 0.0711, R_{sigma} = 0.0538]
Data/restraints/parameters	8507/0/463
Goodness-of-fit on F^2	1.034
Final R indexes [I>=2σ (I)]	R_1 = 0.0424, wR_2 = 0.0733
Final R indexes [all data]	R_1 = 0.0645, wR_2 = 0.0789
Largest diff. peak/hole / e Å^{-3}	0.55/-0.53
Flack parameter	0.011(4)

Experimental

Single crystals of $C_{13}H_{15}BrO_3$ **[AO_JS_CM_910_1_0m]** were selected and placed on magnetic mount with a fibre loop, (Fomblin oil) on a 'Bruker APEX-II CCD' diffractometer with a microfocus MoKα radiation. The crystal was kept at 150.15 K during data collection. Using Olex2 [1], the structure was solved with the XT [2] structure solution program using Intrinsic Phasing and refined with the XL [3] refinement package using Least Squares minimisation. The absolute structure was determined by anomalous dispersion giving Flack and Hooft parameters of 0.011(4) and 0.025(4), respectively.

1. Dolomanov, O.V., Bourhis, L.J., Gildea, R.J, Howard, J.A.K. & Puschmann, H. (2009), J. Appl. Cryst. 42, 339-341.
2. Sheldrick, G.M. (2015). Acta Cryst. A71, 3-8.
3. Sheldrick, G.M. (2008). Acta Cryst. A64, 112-122.

Crystal structure determination of [AO_JS_CM_910_1_0m]

Crystal Data for $C_{13}H_{15}BrO_3$ (M =299.16 g/mol): orthorhombic, space group $P2_12_12_1$ (no. 19), a = 6.355(2) Å, b = 21.957(8) Å, c = 27.613(10) Å, V = 3853(2) Å^3, Z = 12, T = 150.15 K, μ(MoKα) = 3.194 mm^{-1}, $Dcalc$ = 1.547 g/cm^3, 70178 reflections measured (2.37° ≤ 2Θ ≤ 54.336°), 8507 unique (R_{int} = 0.0711, R_{sigma} = 0.0538) which were used in all calculations. The final R_1 was 0.0424 (I > 2σ(I)) and wR_2 was 0.0789 (all data).

Table 2 Fractional Atomic Coordinates (×10^4) and Equivalent Isotropic Displacement Parameters (Å^2×10^3) for AO_JS_CM_910_1_0m. U_{eq} is defined as 1/3 of of the trace of the orthogonalised U_{IJ} tensor.

Atom	*x*	*y*	*z*	U(eq)
Br1C	4928.0(10)	4199.2(2)	607.2(2)	44.46(15)
Br1A	-2517.0(11)	10240.7(2)	582.5(2)	48.23(16)
Br1B	3975.7(11)	6980.3(3)	5147.8(2)	53.54(19)
O1B	4183(5)	6903.4(14)	2130.3(12)	29.5(8)
O2C	8706(5)	7095.2(13)	702.7(11)	27.4(8)
O2B	5830(5)	6111.7(15)	2818.5(11)	28.6(8)
O2A	1109(6)	8670.8(14)	2524.7(12)	31.6(8)
O3A	4105(6)	8642.3(18)	2079.5(13)	41.7(9)
O1C	7327(6)	7402.6(16)	1618.5(12)	35.0(8)
O3B	9212(6)	6123.9(16)	3079.3(13)	37.5(9)
O3C	11516(6)	6625.8(16)	1037.8(14)	41.6(9)
O1A	504(6)	7466.0(17)	2249.5(13)	39.9(10)
C8B	6443(8)	6365.3(19)	3636.1(17)	25.2(11)
C7B	7344(9)	6183.3(19)	3152.4(17)	26.9(11)
C3B	2697(8)	5894(2)	2079.3(17)	27.0(11)
C7C	9800(8)	6601(2)	853.8(17)	25.6(11)
C2B	4651(7)	6282.9(19)	2011.8(16)	22.8(11)
C9C	6530(8)	6024(2)	595.3(18)	29.9(11)
C7A	2311(9)	8811(2)	2140.3(17)	28.3(11)
C2C	7541(8)	7861(2)	1256.9(16)	26.7(11)
C8A	1163(8)	9210(2)	1792.2(17)	26.9(11)
C9A	2154(9)	9369(2)	1358.5(19)	34.1(13)
C11C	6474(9)	4942(2)	646.2(17)	31.0(12)
C8C	8618(8)	6027(2)	753.1(16)	26.2(11)
C13A	-903(8)	9396(2)	1870.2(18)	30.4(12)
C1C	9296(8)	7689(2)	903.4(18)	27.2(11)

Table 2 Fractional Atomic Coordinates (×10[4]) and Equivalent Isotropic Displacement Parameters (Å[2]×10[3]) for AO_JS_CM_910_1_0m. U_{eq} is defined as 1/3 of of the trace of the orthogonalised U_{IJ} tensor.

Atom	*x*	*y*	*z*	U(eq)
C11B	4996(10)	6735(2)	4527.8(17)	31.4(12)
C10A	1114(9)	9687(2)	1006.0(19)	35.6(13)
C2A	387(8)	7638(2)	2748.5(17)	28.0(12)
C11A	-955(9)	9851(2)	1084.9(18)	33.4(12)
C10C	5459(8)	5483(2)	537.7(18)	34.3(12)
C1B	6490(8)	6046(2)	2309.7(16)	25.3(11)
C9B	4401(8)	6594(2)	3679.1(17)	27.9(12)
C13B	7729(9)	6319(2)	4036.5(18)	29.8(12)
C3A	-1892(8)	7772(2)	2873.2(19)	31.9(12)
C6C	9469(8)	8149(2)	490.3(17)	30.1(12)
C6A	1599(9)	8412(3)	3362.7(19)	35.5(13)
C4B	3169(8)	5235(2)	1950.2(18)	33.2(12)
C3C	5449(7)	7941(2)	999.1(18)	26.7(11)
C6B	6957(8)	5381(2)	2196.1(18)	29.3(12)
C5C	7383(9)	8244(2)	235.5(17)	31.4(12)
C1A	1814(8)	8182(2)	2846.9(18)	30.2(12)
C10B	3699(9)	6793(2)	4128.7(19)	33.8(12)
C12B	6985(9)	6505(2)	4492.1(19)	37.9(14)
C4A	-2080(8)	7977(2)	3403.9(18)	33.3(12)
C13C	9598(8)	5476(2)	852.5(19)	35.2(13)
C4C	5650(8)	8414(2)	594.2(19)	32.8(12)
C5B	5018(9)	4983(2)	2247.6(19)	35.1(12)
C12A	-1960(8)	9726(2)	1518.4(18)	34.1(13)
C5A	-695(9)	8535(2)	3495.5(19)	37.4(14)
C12C	8550(9)	4933(2)	797(2)	40.0(14)

Table 3 Anisotropic Displacement Parameters (Å[2]×10[3]) for AO_JS_CM_910_1_0m. The Anisotropic displacement factor exponent takes the form: $-2\pi^2[h^2a^{*2}U_{11}+2hka^*b^*U_{12}+\ldots]$.

Atom	U_{11}	U_{22}	U_{33}	U_{23}	U_{13}	U_{12}
Br1C	60.0(4)	30.2(2)	43.1(3)	3.5(2)	1.4(3)	-7.1(3)
Br1A	68.4(4)	37.4(3)	38.8(3)	5.2(3)	-1.9(4)	7.5(3)
Br1B	70.2(5)	58.6(4)	31.8(3)	-9.9(3)	9.6(3)	-7.6(3)
O1B	26(2)	27.0(16)	36(2)	-0.4(15)	3.3(16)	2.4(15)
O2C	25.6(19)	26.1(15)	30.6(19)	-4.6(13)	-4.6(16)	2.2(14)
O2B	20.6(19)	43.9(19)	21.3(17)	1.3(15)	0.4(16)	-1.4(16)
O2A	28(2)	36.0(18)	31.0(19)	2.3(15)	7.2(18)	3.9(16)
O3A	20(2)	65(2)	39(2)	-4.9(18)	6.0(18)	6.5(19)
O1C	32(2)	47(2)	25.8(19)	5.1(15)	-2.1(18)	-10.7(18)
O3B	21(2)	53(2)	38(2)	-2.3(17)	-2.4(17)	8.8(17)
O3C	25(2)	45(2)	55(3)	12.0(18)	-12(2)	-2.7(17)
O1A	32(2)	50(2)	38(2)	-17.8(17)	-6.4(18)	15.5(18)
C8B	27(3)	23(2)	26(3)	4.7(19)	-2(2)	-3(2)
C7B	24(3)	27(2)	30(3)	3(2)	-2(3)	2(2)
C3B	22(3)	33(2)	26(3)	-2(2)	-1(2)	2(2)

Table 3 Anisotropic Displacement Parameters (Å^2×10^3) for AO_JS_CM_910_1_0m. The Anisotropic displacement factor exponent takes the form: $-2\pi^2[h^2a^{*2}U_{11}+2hka^*b^*U_{12}+\ldots]$.

Atom	U_{11}	U_{22}	U_{33}	U_{23}	U_{13}	U_{12}
C7C	20(3)	37(2)	20(2)	3(2)	3(2)	7(2)
C2B	22(3)	29(2)	18(2)	-0.7(18)	3(2)	5(2)
C9C	31(3)	29(2)	30(3)	3(2)	-2(3)	5(2)
C7A	25(3)	35(3)	25(3)	-10(2)	5(3)	-6(2)
C2C	24(3)	32(2)	23(2)	-5.1(19)	-1(2)	-9(2)
C8A	29(3)	26(2)	26(3)	-7(2)	2(2)	-6(2)
C9A	28(3)	36(3)	39(3)	-4(2)	12(3)	-5(2)
C11C	41(3)	28(2)	23(3)	4(2)	5(3)	-2(2)
C8C	27(3)	34(2)	17(2)	5.0(19)	2(2)	-1(2)
C13A	26(3)	38(3)	27(3)	-3(2)	8(2)	0(2)
C1C	18(3)	32(2)	32(3)	-3(2)	-5(2)	-6(2)
C11B	43(3)	28(2)	22(3)	-2.4(18)	4(3)	-2(3)
C10A	43(4)	32(3)	33(3)	1(2)	18(3)	-1(3)
C2A	25(3)	31(2)	28(3)	-4(2)	-8(2)	8(2)
C11A	46(3)	21(2)	33(3)	-1(2)	-1(3)	-5(2)
C10C	31(3)	37(2)	35(3)	2(2)	-7(3)	5(2)
C1B	17(3)	35(2)	24(3)	4(2)	5(2)	1(2)
C9B	26(3)	32(2)	27(3)	4(2)	-7(2)	3(2)
C13B	28(3)	26(2)	36(3)	-2(2)	-8(3)	1(2)
C3A	27(3)	28(2)	40(3)	-3(2)	-5(2)	0(2)
C6C	28(3)	32(2)	30(3)	-5(2)	8(2)	-5(2)
C6A	33(3)	42(3)	31(3)	-1(2)	-6(3)	-8(3)
C4B	38(3)	31(2)	31(3)	-6(2)	1(2)	-4(2)
C3C	18(3)	31(2)	31(3)	-2(2)	8(2)	-2(2)
C6B	21(3)	41(3)	26(3)	4(2)	2(2)	7(2)
C5C	36(3)	32(2)	26(3)	2(2)	0(3)	-6(2)
C1A	21(3)	34(3)	35(3)	1(2)	-3(2)	2(2)
C10B	25(3)	38(3)	38(3)	0(2)	2(3)	6(2)
C12B	53(4)	35(3)	25(3)	0(2)	-14(3)	-1(3)
C4A	32(3)	34(3)	33(3)	1(2)	3(2)	-1(2)
C13C	21(3)	44(3)	40(3)	9(2)	-5(2)	12(2)
C4C	22(3)	33(2)	43(3)	0(2)	-5(3)	-1(2)
C5B	39(3)	29(2)	36(3)	1(2)	5(3)	6(3)
C12A	28(3)	38(3)	36(3)	-8(2)	7(2)	1(2)
C5A	50(4)	33(3)	30(3)	-6(2)	1(3)	-1(2)
C12C	46(4)	28(3)	46(3)	7(2)	-1(3)	14(3)

Table 4 Bond Lengths for AO_JS_CM_910_1_0m.

Atom	Atom	Length/Å	Atom	Atom	Length/Å
Br1C	C11C	1.906(5)	C8A	C9A	1.398(7)
Br1A	C11A	1.908(5)	C8A	C13A	1.392(7)
Br1B	C11B	1.908(5)	C9A	C10A	1.368(7)
O1B	C2B	1.433(5)	C11C	C10C	1.385(7)
O2C	C7C	1.354(5)	C11C	C12C	1.384(7)
O2C	C1C	1.466(5)	C8C	C13C	1.389(6)

Table 4 Bond Lengths for AO_JS_CM_910_1_0m.

Atom	Atom	Length/Å	Atom	Atom	Length/Å
O2B	C7B	1.342(6)	C13A	C12A	1.386(7)
O2B	C1B	1.474(5)	C1C	C6C	1.527(7)
O2A	C7A	1.344(6)	C11B	C10B	1.382(7)
O2A	C1A	1.464(6)	C11B	C12B	1.365(8)
O3A	C7A	1.210(6)	C10A	C11A	1.381(8)
O1C	C2C	1.424(5)	C2A	C3A	1.517(7)
O3B	C7B	1.211(6)	C2A	C1A	1.526(7)
O3C	C7C	1.204(6)	C11A	C12A	1.384(7)
O1A	C2A	1.430(6)	C1B	C6B	1.523(6)
C8B	C7B	1.507(7)	C9B	C10B	1.390(7)
C8B	C9B	1.396(7)	C13B	C12B	1.405(7)
C8B	C13B	1.379(7)	C3A	C4A	1.537(7)
C3B	C2B	1.518(7)	C6C	C5C	1.515(7)
C3B	C4B	1.521(6)	C6A	C1A	1.517(7)
C7C	C8C	1.493(7)	C6A	C5A	1.527(8)
C2B	C1B	1.521(7)	C4B	C5B	1.536(7)
C9C	C8C	1.396(7)	C3C	C4C	1.531(7)
C9C	C10C	1.379(6)	C6B	C5B	1.518(7)
C7A	C8A	1.491(7)	C5C	C4C	1.528(7)
C2C	C1C	1.530(7)	C4A	C5A	1.529(7)
C2C	C3C	1.518(7)	C13C	C12C	1.374(7)

Table 5 Bond Angles for AO_JS_CM_910_1_0m.

Atom	Atom	Atom	Angle/°	Atom	Atom	Atom	Angle/°
C7C	O2C	C1C	117.7(4)	C6C	C1C	C2C	111.5(4)
C7B	O2B	C1B	117.5(4)	C10B	C11B	Br1B	119.1(4)
C7A	O2A	C1A	118.3(4)	C12B	C11B	Br1B	118.9(4)
C9B	C8B	C7B	121.6(5)	C12B	C11B	C10B	121.9(5)
C13B	C8B	C7B	117.7(4)	C9A	C10A	C11A	118.7(5)
C13B	C8B	C9B	120.6(5)	O1A	C2A	C3A	108.7(4)
O2B	C7B	C8B	111.5(4)	O1A	C2A	C1A	110.3(4)
O3B	C7B	O2B	125.2(5)	C3A	C2A	C1A	112.0(4)
O3B	C7B	C8B	123.3(5)	C10A	C11A	Br1A	119.8(4)
C2B	C3B	C4B	110.2(4)	C10A	C11A	C12A	121.6(5)
O2C	C7C	C8C	111.1(4)	C12A	C11A	Br1A	118.5(4)
O3C	C7C	O2C	124.0(5)	C9C	C10C	C11C	119.0(5)
O3C	C7C	C8C	124.9(4)	O2B	C1B	C2B	105.3(4)
O1B	C2B	C3B	109.7(4)	O2B	C1B	C6B	110.2(4)
O1B	C2B	C1B	111.2(4)	C2B	C1B	C6B	111.5(4)
C3B	C2B	C1B	111.7(4)	C10B	C9B	C8B	119.1(5)
C10C	C9C	C8C	120.6(4)	C8B	C13B	C12B	119.8(5)
O2A	C7A	C8A	111.4(4)	C2A	C3A	C4A	110.3(4)
O3A	C7A	O2A	125.1(5)	C5C	C6C	C1C	112.0(4)
O3A	C7A	C8A	123.5(5)	C1A	C6A	C5A	111.7(4)
O1C	C2C	C1C	110.1(4)	C3B	C4B	C5B	111.6(4)
O1C	C2C	C3C	109.1(4)	C2C	C3C	C4C	110.4(4)

Table 5 Bond Angles for AO_JS_CM_910_1_0m.

Atom	Atom	Atom	Angle/°	Atom	Atom	Atom	Angle/°
C3C	C2C	C1C	111.6(4)	C5B	C6B	C1B	112.0(4)
C9A	C8A	C7A	118.6(5)	C6C	C5C	C4C	111.3(4)
C13A	C8A	C7A	122.3(5)	O2A	C1A	C2A	106.5(4)
C13A	C8A	C9A	119.0(5)	O2A	C1A	C6A	107.4(4)
C10A	C9A	C8A	121.3(5)	C6A	C1A	C2A	112.0(4)
C10C	C11C	Br1C	118.8(4)	C11B	C10B	C9B	119.5(5)
C12C	C11C	Br1C	119.8(4)	C11B	C12B	C13B	118.9(5)
C12C	C11C	C10C	121.4(5)	C5A	C4A	C3A	110.3(4)
C9C	C8C	C7C	122.7(4)	C12C	C13C	C8C	121.1(5)
C13C	C8C	C7C	118.3(5)	C5C	C4C	C3C	111.6(4)
C13C	C8C	C9C	118.9(5)	C6B	C5B	C4B	111.3(4)
C12A	C13A	C8A	120.2(5)	C11A	C12A	C13A	119.1(5)
O2C	C1C	C2C	105.9(4)	C6A	C5A	C4A	111.7(4)
O2C	C1C	C6C	108.9(4)	C13C	C12C	C11C	119.0(5)

Table 6 Torsion Angles for AO_JS_CM_910_1_0m.

A	B	C	D	Angle/°	A	B	C	D	Angle/°
Br1C	C11C	C10C	C9C	175.9(4)	C8A	C9A	C10A	C11A	0.1(7)
Br1C	C11C	C12C	C13C	-175.9(4)	C8A	C13A	C12A	C11A	1.9(7)
Br1A	C11A	C12A	C13A	174.4(4)	C9A	C8A	C13A	C12A	1.0(7)
Br1B	C11B	C10B	C9B	-177.6(4)	C9A	C10A	C11A	Br1A	-175.3(4)
Br1B	C11B	C12B	C13B	179.4(4)	C9A	C10A	C11A	C12A	2.9(7)
O1B	C2B	C1B	O2B	58.8(5)	C8C	C9C	C10C	C11C	1.1(8)
O1B	C2B	C1B	C6B	178.4(4)	C8C	C13C	C12C	C11C	-1.0(8)
O2C	C7C	C8C	C9C	-9.4(6)	C13A	C8A	C9A	C10A	-2.0(7)
O2C	C7C	C8C	C13C	175.1(4)	C1C	O2C	C7C	O3C	-19.8(7)
O2C	C1C	C6C	C5C	63.0(5)	C1C	O2C	C7C	C8C	161.4(4)
O2B	C1B	C6B	C5B	63.1(5)	C1C	C2C	C3C	C4C	-56.0(5)
O2A	C7A	C8A	C9A	-176.4(4)	C1C	C6C	C5C	C4C	53.8(5)
O2A	C7A	C8A	C13A	-0.6(6)	C10A	C11A	C12A	C13A	-3.9(7)
O3A	C7A	C8A	C9A	4.0(7)	C2A	C3A	C4A	C5A	57.2(5)
O3A	C7A	C8A	C13A	179.9(5)	C10C	C9C	C8C	C7C	-175.7(5)
O1C	C2C	C1C	O2C	57.8(5)	C10C	C9C	C8C	C13C	-0.2(7)
O1C	C2C	C1C	C6C	176.1(4)	C10C	C11C	C12C	C13C	1.9(8)
O1C	C2C	C3C	C4C	-177.8(4)	C1B	O2B	C7B	O3B	-9.6(6)
O3C	C7C	C8C	C9C	171.8(5)	C1B	O2B	C7B	C8B	168.9(3)
O3C	C7C	C8C	C13C	-3.8(7)	C1B	C6B	C5B	C4B	52.9(6)
O1A	C2A	C3A	C4A	-178.3(4)	C9B	C8B	C7B	O2B	-16.8(6)
O1A	C2A	C1A	O2A	58.1(5)	C9B	C8B	C7B	O3B	161.7(5)
O1A	C2A	C1A	C6A	175.2(4)	C9B	C8B	C13B	C12B	-0.2(7)
C8B	C9B	C10B	C11B	-3.1(7)	C13B	C8B	C7B	O2B	165.3(4)
C8B	C13B	C12B	C11B	-0.6(7)	C13B	C8B	C7B	O3B	-16.1(7)
C7B	O2B	C1B	C2B	-152.8(4)	C13B	C8B	C9B	C10B	2.1(7)
C7B	O2B	C1B	C6B	86.8(5)	C3A	C2A	C1A	O2A	-63.0(5)
C7B	C8B	C9B	C10B	-175.7(4)	C3A	C2A	C1A	C6A	54.1(5)
C7B	C8B	C13B	C12B	177.6(4)	C3A	C4A	C5A	C6A	-56.4(6)

Table 6 Torsion Angles for AO_JS_CM_910_1_0m.

A	B	C	D	Angle/°	A	B	C	D	Angle/°
C3B	C2B	C1B	O2B	-64.0(4)	C6C	C5C	C4C	C3C	-55.3(5)
C3B	C2B	C1B	C6B	55.5(5)	C4B	C3B	C2B	O1B	179.5(4)
C3B	C4B	C5B	C6B	-54.7(6)	C4B	C3B	C2B	C1B	-56.8(5)
C7C	O2C	C1C	C2C	-106.9(4)	C3C	C2C	C1C	O2C	-63.5(4)
C7C	O2C	C1C	C6C	133.1(4)	C3C	C2C	C1C	C6C	54.8(5)
C7C	C8C	C13C	C12C	175.8(5)	C1A	O2A	C7A	O3A	-13.4(7)
C2B	C3B	C4B	C5B	56.3(5)	C1A	O2A	C7A	C8A	167.0(4)
C2B	C1B	C6B	C5B	-53.5(5)	C1A	C2A	C3A	C4A	-56.3(5)
C9C	C8C	C13C	C12C	0.2(8)	C1A	C6A	C5A	C4A	54.1(6)
C7A	O2A	C1A	C2A	-106.7(5)	C10B	C11B	C12B	C13B	-0.4(7)
C7A	O2A	C1A	C6A	133.2(4)	C12B	C11B	C10B	C9B	2.3(7)
C7A	C8A	C9A	C10A	174.0(4)	C5A	C6A	C1A	O2A	64.2(5)
C7A	C8A	C13A	C12A	-174.8(4)	C5A	C6A	C1A	C2A	-52.4(6)
C2C	C1C	C6C	C5C	-53.5(5)	C12C	C11C	C10C	C9C	-1.9(8)
C2C	C3C	C4C	C5C	56.3(5)					

Table 7 Hydrogen Atom Coordinates (Å×10^4) and Isotropic Displacement Parameters (Å^2×10^3) for AO_JS_CM_910_1_0m.

Atom	*x*	*y*	*z*	U(eq)
H1B	5114.11	7131.69	2013.45	44
H1C	8338.12	7425.99	1813.48	52
H1A	1553.64	7239.78	2206.53	60
H3BA	1552.32	6049.55	1869.89	32
H3BB	2220.87	5919.3	2420.11	32
H2B	5059.28	6265.96	1662.28	27
H9C	5841.41	6398.12	527.07	36
H2C	7921.37	8253.46	1418.1	32
H9A	3578.33	9254.33	1307.51	41
H13A	-1589.85	9297.13	2165.45	36
H1CA	10669.26	7657.55	1078.6	33
H10A	1802.31	9792.3	712.3	43
H2A	875.01	7287.14	2950.7	34
H10C	4046.42	5481.02	425.18	41
H1BA	7768.69	6299.7	2247.4	30
H9B	3502.31	6612.9	3404.48	34
H13B	9114.59	6161.37	4004.33	36
H3AA	-2753.63	7402.3	2821.25	38
H3AB	-2432.85	8096.65	2657.71	38
H6CA	9967.72	8543.09	620.96	36
H6CB	10522.16	8002.96	253.06	36
H6AA	2419.9	8792.93	3399.56	43
H6AB	2190.16	8106.53	3588.19	43
H4BA	3511.04	5206.79	1601.12	40
H4BB	1902.98	4983.52	2010.84	40
H3CA	4367.11	8075.27	1234.16	32
H3CB	4993.92	7546.9	860.41	32

Table 7 Hydrogen Atom Coordinates (Å×10^4) and Isotropic Displacement Parameters (Å^2×10^3) for AO_JS_CM_910_1_0m.

Atom	*x*	*y*	*z*	U(eq)
H6BA	7499.71	5348.7	1860.94	35
H6BB	8066.29	5231.52	2418.11	35
H5CA	6983.44	7865.73	63.43	38
H5CB	7532.94	8572	-8.01	38
H1AA	3312.8	8074.83	2776.57	36
H10B	2339.37	6968.02	4161.42	41
H12B	7852.53	6470.77	4771.01	45
H4AA	-3564.34	8078.07	3477.43	40
H4AB	-1641.9	7640.81	3620.36	40
H13C	11016.96	5474.04	960.74	42
H4CA	4292.64	8446.66	419.96	39
H4CB	5968.2	8817.1	737.63	39
H5BA	4608.31	4960.89	2593.07	42
H5BB	5349.96	4564.9	2136.75	42
H12A	-3355.66	9864.75	1573.85	41
H5AA	-1225.48	8881.31	3301.57	45
H5AB	-782.83	8648.79	3841.7	45
H12C	9240.43	4557.9	861.01	48

Refinement model description

Number of restraints - 0, number of constraints - unknown.

Details:

```
1. Fixed Uiso
 At 1.2 times of:
 All C(H) groups, All C(H,H) groups
 At 1.5 times of:
 All O(H) groups
2.a Ternary CH refined with riding coordinates:
 C2B(H2B), C2C(H2C), C1C(H1CA), C2A(H2A), C1B(H1BA), C1A(H1AA)
2.b Secondary CH2 refined with riding coordinates:
 C3B(H3BA,H3BB), C3A(H3AA,H3AB), C6C(H6CA,H6CB), C6A(H6AA,H6AB),
C4B(H4BA,
 H4BB), C3C(H3CA,H3CB), C6B(H6BA,H6BB), C5C(H5CA,H5CB),
C4A(H4AA,H4AB),
 C4C(H4CA,H4CB), C5B(H5BA,H5BB), C5A(H5AA,H5AB)
2.c Aromatic/amide H refined with riding coordinates:
 C9C(H9C), C9A(H9A), C13A(H13A), C10A(H10A), C10C(H10C), C9B(H9B),
C13B(H13B),
 C10B(H10B), C12B(H12B), C13C(H13C), C12A(H12A), C12C(H12C)
2.d Idealised tetrahedral OH refined as rotating group:
 O1B(H1B), O1C(H1C), O1A(H1A)
```
